# Elevated intron retention implicates neuroinflammation in brains of individuals with alcohol use disorder

**DOI:** 10.1093/braincomms/fcag264

**Published:** 2026-07-29

**Authors:** Rudong Li, Jill L Reiter, Season K Wyatt-Johnson, Chuanpeng Dong, Caine S Smith, Nick Green, Hongyu Gao, Sheketha R Hauser, Manav Kapoor, Julia Stevens, R Dayne Mayfield, Alison Goate, Yue Wang, Howard J Edenberg, Richard L Bell, Greg Trevor Sutherland, Randy Brutkiewicz, Yunlong Liu

**Affiliations:** Center for Computational Biology and Bioinformatics (CCBB), Indiana University (IU) School of Medicine, Indianapolis, IN 46202, USA; Department of Medical and Molecular Genetics (MMGE), IU School of Medicine, Indianapolis, IN 46202, USA; Center for Computational Biology and Bioinformatics (CCBB), Indiana University (IU) School of Medicine, Indianapolis, IN 46202, USA; Department of Medical and Molecular Genetics (MMGE), IU School of Medicine, Indianapolis, IN 46202, USA; Department of Microbiology and Immunology, IU School of Medicine, Indianapolis, IN 46202, USA; Stark Neurosciences Research Institute, IU School of Medicine, Indianapolis, IN 46202, USA; Center for Computational Biology and Bioinformatics (CCBB), Indiana University (IU) School of Medicine, Indianapolis, IN 46202, USA; Department of Medical and Molecular Genetics (MMGE), IU School of Medicine, Indianapolis, IN 46202, USA; New South Wales (NSW) Brain Tissue Research Centre, University of Sydney, Sydney, NSW 2006, Australia; Charles Perkins Centre and School of Medical Sciences, University of Sydney, Sydney, NSW 2006, Australia; Center for Computational Biology and Bioinformatics (CCBB), Indiana University (IU) School of Medicine, Indianapolis, IN 46202, USA; Department of Medical and Molecular Genetics (MMGE), IU School of Medicine, Indianapolis, IN 46202, USA; Center for Computational Biology and Bioinformatics (CCBB), Indiana University (IU) School of Medicine, Indianapolis, IN 46202, USA; Department of Medical and Molecular Genetics (MMGE), IU School of Medicine, Indianapolis, IN 46202, USA; Stark Neurosciences Research Institute, IU School of Medicine, Indianapolis, IN 46202, USA; Department of Psychiatry, IU School of Medicine, Indianapolis, IN 46202, USA; Ronald M. Loeb Center for Alzheimer’s Disease, Department of Neuroscience, Icahn School of Medicine at Mount Sinai, New York, NY 10029, USA; Department of Genetics and Genomic Sciences, Icahn School of Medicine at Mount Sinai, New York, NY 10029, USA; New South Wales (NSW) Brain Tissue Research Centre, University of Sydney, Sydney, NSW 2006, Australia; Charles Perkins Centre and School of Medical Sciences, University of Sydney, Sydney, NSW 2006, Australia; Waggoner Center for Alcohol and Addiction Research (WCAAR), University of Texas at Austin, Austin, TX 78712, USA; Department of Neuroscience, University of Texas at Austin, Austin, TX 78712, USA; Ronald M. Loeb Center for Alzheimer’s Disease, Department of Neuroscience, Icahn School of Medicine at Mount Sinai, New York, NY 10029, USA; Department of Genetics and Genomic Sciences, Icahn School of Medicine at Mount Sinai, New York, NY 10029, USA; Department of Medical and Molecular Genetics (MMGE), IU School of Medicine, Indianapolis, IN 46202, USA; Department of Medical and Molecular Genetics (MMGE), IU School of Medicine, Indianapolis, IN 46202, USA; Department of Biochemistry, Molecular Biology, and Pharmacology, IU School of Medicine, Indianapolis, IN 4620, USA; Department of Psychiatry, IU School of Medicine, Indianapolis, IN 46202, USA; New South Wales (NSW) Brain Tissue Research Centre, University of Sydney, Sydney, NSW 2006, Australia; Charles Perkins Centre and School of Medical Sciences, University of Sydney, Sydney, NSW 2006, Australia; Department of Microbiology and Immunology, IU School of Medicine, Indianapolis, IN 46202, USA; Stark Neurosciences Research Institute, IU School of Medicine, Indianapolis, IN 46202, USA; Center for Computational Biology and Bioinformatics (CCBB), Indiana University (IU) School of Medicine, Indianapolis, IN 46202, USA; Department of Medical and Molecular Genetics (MMGE), IU School of Medicine, Indianapolis, IN 46202, USA

**Keywords:** alcohol use disorder, double-stranded RNA, intron retention, neuroinflammation, alcohol-preferring P rats

## Abstract

Intron retention, a form of alternative RNA splicing, can occur as part of normal gene regulation or result from disruption of the splicing machinery. Retained introns can potentially form double-stranded RNA, activating innate immune sensors and inflammation. This mechanism has been implicated in cancer but has not been studied in neuropsychiatric diseases like alcohol use disorder. We systematically analysed transcriptome-wide intron retention events in *post-mortem* brain tissue from 142 individuals (66 with alcohol use disorder and 76 controls), encompassing 320 region-specific samples from the superior frontal cortex, nucleus accumbens, central nucleus and basolateral amygdala. Analyses were adjusted for demographic, technical and biological covariates. Validation was performed in alcohol-preferring (P) rats using long-read sequencing. In complementary experiments, immunofluorescent staining was used to detect double-stranded RNA in rat brain tissue, while single-cell RNA-sequencing was performed to test activation of double-stranded RNA-sensing pathways in human brains. Brains from individuals with alcohol use disorder showed significantly higher total intron retention compared with controls, independent of age, with females showing greater increases than males. A total of 368 introns were positively associated with alcohol use disorder, and these introns were significantly longer and had weaker splice acceptor sites compared with non-associated introns. Genes harbouring these intron retention events were enriched in Purkinje neurons, visual cortex neurons and oligodendrocytes. Computational predictions indicated these long introns could form duplex RNA structures. Increased double-stranded RNA was confirmed experimentally in multiple brain regions of alcohol-consuming rats, where it co-localized primarily with neuronal nuclei and dendrites. In individuals with alcohol use disorder, we found that multiple pathways including double-stranded RNA responses, neuroinflammation, interferon and NF-κB signalling, adaptive immunity and apoptosis were activated. In addition, NeuN-positive neuronal counts significantly decreased in both the prefrontal and visual cortices. Furthermore, single-cell analysis demonstrated upregulation of TICAM1, the target of double-stranded RNA sensor TLR3, in oligodendrocytes, as well as widespread activation of downstream inflammatory pathways across glial and neuronal cell types. These findings provide the first evidence that chronic alcohol consumption promotes an overall increase of intron retention in the brain and is associated with the presence of double-stranded RNA. Furthermore, the double-stranded RNA may contribute to neuronal loss and brain pathology by activating a neuroinflammatory response.

## Teaser

Li *et al*. reported that alcohol use disorder is associated with significantly increased intron retention in the human brain, which was replicated in a rat model. The retained introns may form double-stranded RNA and activate neuroinflammatory pathways. These changes are associated with neuronal loss, suggesting a new mechanism behind alcohol-related brain damage.

## Graphical Abstract

**Graphical Abstract:**
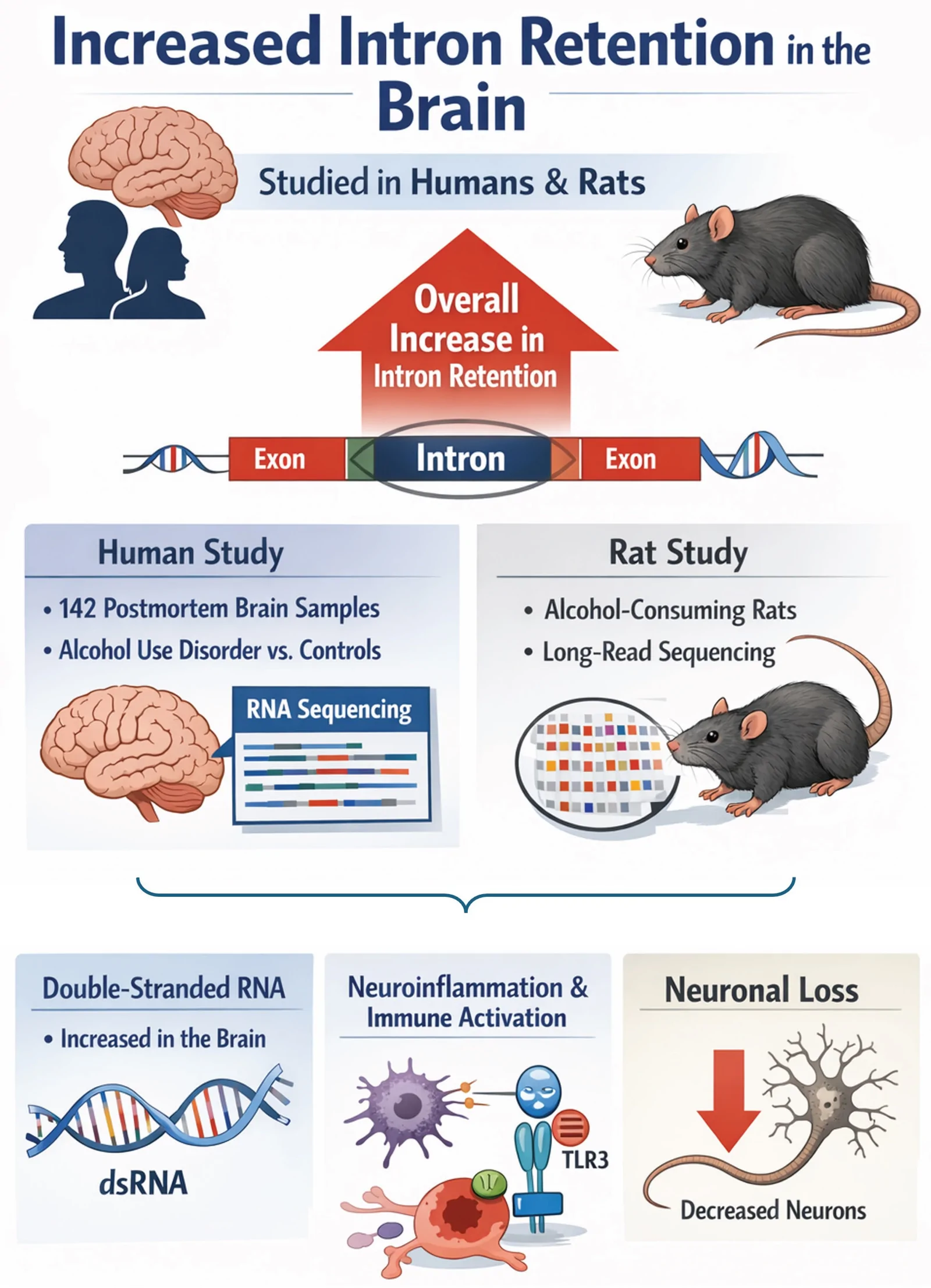

## Introduction

Alcohol use disorder (AUD) is a chronic relapsing medical condition characterized by compulsive alcohol consumption, lack of control over intake and continued use despite harmful consequences. It represents a major global health challenge, ranking among the leading risk factors for premature mortality and disability. Each year, AUD contributes to 2.6 million deaths and accounts for 4.7% of the global burden of disease.^1^ Worldwide, more than 280 million individuals are estimated to be affected, including nearly 30 million in the United States alone.^2,3^ Beyond its direct health burden, AUD is associated with a wide spectrum of comorbidities, including mental and behavioural disorders, liver and cardiovascular diseases, heightened vulnerability to viral infections and an increased risk of cancer.^4,5^

RNA splicing is an essential gene regulatory process in which introns are excised from precursor mRNAs, and exons are ligated to produce mature transcripts. Under certain conditions, however, introns can be aberrantly retained in transcripts due to altered or disrupted splicing machinery. Intron retention (IR) is a distinct mode of alternative splicing that plays critical roles in both normal physiology and disease.^6^ IR has been implicated in aging, cancer, liver disease and various neuropsychiatric disorders, including AUD.^7-9^

IR can result from either regulated splicing programs or aberrant dysregulation of the splicing machinery. Regulated IR is particularly prominent in the brain, where it fine-tunes gene expression and contributes to transcriptomic and proteomic diversity.^10,11^ In contrast, aberrant IR occurs when splicing fidelity is compromised due to factors such as spliceosome dysfunction, transcriptional overload, cellular stress or epigenetic alterations.^12^ Typically, mRNAs with retained introns are targeted for degradation by nuclear exosomes or cytoplasmic non-sense-mediated decay.^13^ However, some mRNAs with aberrantly retained introns escape these surveillance mechanisms and exert pathological effects. For instance, translation of aberrantly retained introns can generate peptides that are not normally present in the cell (i.e. neo-peptides) and displayed on cell-surface major histocompatibility complex molecules, thereby perturbing immune checkpoint pathways, as demonstrated in multiple cancer studies.^14-18^ In addition, retained introns may fold into double-stranded RNA (dsRNA) structures that mimic viral RNA, activating innate immune sensors and triggering antiviral responses, neuroinflammation and apoptosis.^12^ Aberrant IR has been documented in cancer,^12,17,18^ neuropsychiatric disorders,^8^ alcoholic and non-alcoholic liver diseases,^19^ and aging-related conditions.^20,21^ Despite this growing body of evidence, the role of IR in AUD remains poorly understood.

In this study, we systematically examined transcriptome-wide IR events in the brains of individuals with and without AUD. Using RNA-sequencing (RNQ-seq) data from *post-mortem* brain tissue,^22,23^ we identified an overall IR increase in AUD, with AUD-associated introns showing disproportionately greater length. This observation led us to hypothesize that alcohol-induced IR facilitates dsRNA accumulation, which in turn activates neuroinflammatory and apoptotic signalling. We tested this hypothesis by validating dsRNA presence in brain tissue from alcohol-preferring (P) rats,^24,25^ and by demonstrating activation of dsRNA-sensing, immune and apoptotic signalling pathways in brain samples from humans with AUD. Furthermore, genes harbouring human AUD-associated IR were enriched in critical neuronal and glial cell populations, and single-cell analyses revealed upregulation of dsRNA-sensor targets and downstream inflammatory cascades within relevant cell types.

Together, these findings provide new insights into how chronic alcohol exposure disrupts RNA splicing, promotes dsRNA-mediated immune activation and contributes to neuronal injury and neuroimmune dysfunction in AUD.

## Materials and methods

### Human brain RNA-seq datasets

The RNA-seq data included two different sets of individual samples from the New South Wales Brain Tissue Resource Centre (BTRC). The first set consisted of *post-mortem* tissue samples of 60 individuals (30 AUD, all European ancestry; 30 non-AUD controls, 26 European and 4 non-European).^22^ Samples were collected from four brain regions of each individual: the superior frontal cortex (SFC), nucleus accumbens (NAC), central nucleus (CE) and basolateral amygdala (BLA), with two individuals having missing measurements for the NAC. Protocols for RNA sample extraction, library preparation, sequencing and quality control were described previously.^22^ The RNA-seq data were processed at the Waggoner Center for Alcohol and Addiction Research at the University of Texas at Austin. Raw reads in FASTQ files were aligned to the human genome hg38 using STAR version 2.7.3a,^26^ and the RNA sequence quality was assessed using FastQC.^27^ The second set included *post-mortem* tissue samples of 83 individuals, with 1 subject overlapping with the 60 subjects in the first cohort for quality assurance.^23^ Among the 82 independent individuals (36 AUD and 46 non-AUD controls, all European ancestry), frozen samples of the superior frontal gyrus (Brodmann Area 8; also referred to as prefrontal cortex, PFC) were collected. Sample processing, classification and RNA integrity testing, as well as RNA library preparation and sequencing, were performed at the Ronald M. Loeb Center for Alzheimer’s Disease, Icahn School of Medicine at Mount Sinai. Details of the protocols were described in a previous study.^23^ Similar protocols were applied to read alignment and quality assessment. Together, there were a total of 320 (i.e. 60 × 4 − 2 + 82) samples.

### Rat brain RNA-seq samples

Selectively bred alcohol-preferring (P) rats were maintained under a 12-h light–dark cycle in the Indiana Alcohol Research Center Animal Core, which is fully accredited by the Association for the Assessment and Accreditation of Laboratory Animal Care (AAALAC). All research protocols were approved by the Indiana University School of Medicine Institutional Animal Care and Use Committee and were in accordance with the guidelines of the *Guide for the Care and Use of Laboratory Animals* (Institute of Laboratory Animal Resources, Commission on Life Sciences, National Research Council).

Adolescent male P rats from five litters were randomized to either the alcohol-drinking (ALC, *n* = 8) or control groups (CTRL, *n* = 8); each individual rat was considered the experimental unit. Rats were transferred to a vivarium room maintained on a 12 h/12 h dark/light cycle (1000 h/2200 h) and individually housed in hanging stainless steel wire mesh cages. All animals were provided with free access to standard laboratory chow and water. Alcohol drinking began on Post-natal day 31 ± 1 and continued for 10 weeks. The ALC group was provided with continuous free-choice access to three bottles containing water, 15% ethanol or 30% ethanol (available concurrently) to model voluntary chronic excessive alcohol drinking. The amount of alcohol consumed was determined daily, and animals were weighed twice per week. The average weekly alcohol consumption of the rats used in this study ranged from 8 to 12 g of alcohol per kilogram body weight per day (8–12 g/kg/day). These values of ethanol intake corresponded with other published values from peri-adolescent P rats.^24,28,29^ At the end of the 10-week drinking period, animals were euthanized 4 h after removal of the alcohol bottles over a 2-day period, with four rats from each group sacrificed each day. The rats were euthanized by CO_2_ inhalation and ketamine injection before decapitation. Rat heads were cooled in isopentane on dry ice before removing the brains. The NAC shell was manually dissected under a dissecting microscope. Dissected brain tissue was immediately placed in RNAlater solution and stored at 4°C overnight and then at −80°C until nucleic acid isolation.

RNA isolation was performed using the AllPrep DNA/RNA mini kit (Qiagen, Valencia, CA, USA) following the manufacturer’s protocol. All 16 samples were processed in a single run. RNA integrity was assessed using the Experion Automated Electrophoresis station with RNA StdSens microfluidic chips (BioRad). All samples showed an RNA quality indicator >8.3 with an average of 9.2 on a scale of 1–10, where 10 indicates completely intact RNA. Approximately 500 ng total RNA was used for RNA-seq library preparation following the Illumina TruSeq stranded mRNA sample preparation guide, and 100-bp paired-end sequencing was performed on an Illumina HiSeq 2000 platform. RNA library preparation and sequencing were performed at the Greeley Children’s Cancer Research Institute at the University of Texas Health Science Center, San Antonio, TX, USA. The RNA sequence quality was assessed using FastQC,^27^ and raw reads in FASTQ files were aligned to the rat genome rn6 using STAR version 2.7.3a.^26^

### Identification of IR events, quantification of intron content, and statistical analysis

First, annotation of the transcript isoforms *T_i_* (*i* = 1, 2, …) of each gene was extracted from the GTF file (Gencode.v32). Each *T_i_* consists of a set of exons $E_{T_{i}}=\{E_{j}|E_{j}\in T_{i},\;j=1,2,\ldots \}$, and the union of the exon sets was extracted as $U=U_{i}E_{T_{i}}$. Considering all the exons $E_{{\;j}^{\prime }}\in U$, ${\;j}^{\prime }=1,2,\ldots ,|U|$, introns were defined as the interval between adjacent exons in ***U*** (Supplementary Fig. 1). Thereby, a reannotated GTF file was compiled using the introns together with the exons in the union. Next, expression levels of the exons and introns were quantified based on the reannotated GTF file, using uniquely mapped reads from the RNA-seq data via HTseq as previously described.^18^ Identified IR events were further filtered using the following criteria: (i) both the intron region and its two flanking exon regions had read counts >10; and (ii) the transcripts per million (TPM) ratio of intron to flanking exon was >0.05 and <0.5.^17^ These filters allowed the identification of IR events having expression levels comparable with the flanking exons in the mRNA transcripts that were composed of a mixture of normal and aberrant splicing products.

Next, we defined an aggregate metric of intron content (IC) for both the human and rat samples as the actual load of introns retained in the transcriptome. Specifically, it was calculated by the normalized expression level of an intron multiplied by the intron length. For example, for intron *i* in sample *k*


$$IC_{i,k}=[Expression{]}_{i,k}\times [Length{]}_{i}$$

Thus, the total IC of sample *k* is $IC_{k}=\sum_{i}IC_{i,k}=\sum_{i}\left({\left[Expression\right]}_{i,k}\times {\left[Length\right]}_{i}\right)$. The rationale of using this metric by considering the intron length is to characterize reasonably the global effects of IR events. In addition, to leverage the power of brain region–specific sample measurements and appreciate potential brain region–specific IR differences, we adopted a meta-analysis strategy to implement the statistical analysis for IC. Specifically, for the 320 brain region–specific measurements, a generalized linear mixed-effect model was implemented to examine the relationship between the IC and AUD status, adjusting for the covariates, including age, sex, RNA-seq cohort (*coh*), RNA integrity number (RIN, *rin*), *post-mortem interval* (PMI, *pmi*) and brain regions (*br*). The individual difference (*idv*) among the RNA-seq samples was regarded as a random effect following a negative binomial distribution (*b*). In addition, interaction between AUD and age (*AUD* × *age*) as well as interaction between AUD and sex (*AUD* × *sex*) were also examined.


$$\begin{array}{rl} E(\log(IC)) & =\beta_{0}+\beta_{1}\cdot x_{AUD}+\beta_{2}\cdot x_{age}+\beta_{3}\cdot x_{sex}+\beta_{1,2}\cdot x_{AUD}\\ & \;\times x_{age}+\beta_{1,3}\cdot x_{AUD}\times x_{sex}+\;\beta_{4}\cdot x_{coh}+\beta_{5}\cdot x_{rin}\\ & \;+\beta_{6}\cdot x_{\;pmi}+\beta_{7}\cdot br+b\cdot \varepsilon_{idv} \end{array}$$

The effect size *β*_1_ was estimated, along with its standard error and *P*-value.

For the 16 rat samples, IC values were calculated similarly, and we performed a Wilcoxon test between the ALC and CTRL groups.

### Identification of AUD-associated IR events for humans

AUD-associated IR events were defined as those exhibiting significant differences in occurrence between the samples from individuals with and without AUD. In the human samples, we examined the association between the occurrence (1, presence; 0, absence) of each IR event and the AUD phenotype using a generalized estimating equation^30^ considering brain region–specific measurements from the same individual. Binomial distribution with the logit link function was used. Only IR events with sufficient variability, i.e. the number of occurrences was >10 and <310 among the 320 total measurements, were analysed. The generalized estimating equation model was conducted using the R package *geepack*,^31^ and the model was adjusted with the covariates of age, sex, RNA-seq cohort, RIN and PMI. Aberrant IR (aIR) events were regarded as AUD-associated IR events that were not annotated in the non-AUD controls, based on an approach described previously (https://github.com/cpdong/intronneoantigen).^17,18^

### Splicing code analysis

The strength of a splice junction was calculated as a posterior probability that the sequence at each splice site matched the positional weight matrix of the donor or acceptor site. The splice donor site was defined from position −3 to +7 relative to the exon–intron boundary, whereas the splice acceptor site was from position −13 to +1. For each nucleotide *j* = A, T, C, G at every position *i*, the information weight value (*R_iw_*) is


$$R_{iw}(i,j)=\log_{2}\;(\;f_{i,j}/d_{j})=\log_{2}\;f_{i,j}+2$$

where *f_i,j_* is the frequency of observing nucleotide *j* at the position *i* of the splice site sequence. All the *f_i,j_* values were referenced from Itoh *et al.*^32^, and *d_j_* = 0.25 was taken as the background probability of nucleotide *j*. Thereby, the splicing score was the sum of the information weights at each of the positions: $SS=\sum_{i}R_{iw}$. In addition, the presence of canonical splice sites, that is, whether they follow the GT-AG rule at the 5′ and 3′ splice junctions, was also examined. A similar strategy for evaluating the strength of splice junctions has been used previously.^33,34^

### Tissue-cell type and gene ontology enrichment

Cell type enrichments for the genes harbouring the human AUD-associated IR events were predicted by WebCSEA, a public analytic tool built on human single-cell RNA sequencing (scRNA-seq) data from >5.5 million cells from 111 curated tissue panels and 1355 tissue-cell types. A gene list was provided as input, and the output enrichment result was computed based on the cell type–specific expression of the genes in the database, via a permutation-based corrective algorithm designed to eliminate the bias from the number of signature genes among different cell types.^35^ Significant enrichments were identified by a false-discovery rate (FDR) < 0.05, and the subset of genes for each cell type was enumerated.

We also used the R package ClusterProfiler (version 4.0.5)^36^ to perform enrichment analysis for these AUD-associated IR genes, with respect to gene ontology (GO) biological processes and molecular functions. The enrichment significance threshold was FDR < 0.05.

### Gene set enrichment analysis

We used the R package ClusterProfiler (version 4.0.5) to perform gene set enrichment analysis (GSEA) to identify upregulated or downregulated pathways (i.e. gene sets) in the AUD brains of humans as well as alcohol-consuming P rats. In the human brain RNA-seq dataset (4 brain regions, 142 individuals; 320 measured samples altogether), genes having counts-per-million >1 in at least 10 measured samples were considered sufficiently expressed and were thus retained in the analysis, resulting in a total of 21 630 genes. Fold changes (FC) of these genes in AUD versus control were computed together using all 320 samples via the EdgeR package (version 3.34.1),^37^ adjusting for the covariates of age, sex, RNA-seq cohort, PMI, RIN, brain region and individual. Using the ranked FC, enriched (or depleted) gene sets were enumerated, and the enrichment scores and *P*-values were computed. Under FDR < 0.05, upregulated pathways were identified as normalized enrichment score (NES) > 0, and downregulated pathways were indicated by NES < 0. In the rat dataset (NAC, 16 samples), genes having counts-per-million >0.5 in at least 8 samples were retained, resulting in 15 834 genes. Because covariates for the rat samples (e.g. age, sex and cohort) were controlled by study design, the ALC and CTRL groups were directly compared in EdgeR. FC calculation and pathway enrichment criteria were the same as described above.

### Prediction of RNA secondary structure and dsRNA

We used SPOT-RNA, a deep learning method based on RNA sequences, to predict base-pairing for the (non-coding) intron segments retained in the mRNA.^38^ The model predicted whether each retained intron was capable of forming double-stranded (i.e. duplex) regions in the RNA secondary structure. Given the length *L* of the intron, an *L* × *L* base-pairing matrix was generated, and a Perl language-based tool bpRNA (https://github.com/hendrixlab/bpRNA) was used to parse the matrix to transform it into the human-readable secondary structure annotation.^39^ Preferred duplex lengths for common dsRNA sensors were referenced from a review article.^40^

### Immunofluorescence staining of dsRNA in rat brains, microscopy, and semi-quantitative immunofluorescence densitometry analysis

Whole brains were harvested from a separate set of alcohol-drinking and alcohol-naïve P rats than were used for RNA-seq. In this experiment, adolescent male P rats were subjected to a drinking-in-the-dark-multiple-scheduled access (DID-MSA) procedure as described previously.^41^ Briefly, the alcohol-drinking group was provided access to 15% ethanol for two 1-h sessions separated by 3 h of ethanol deprivation during the dark cycle, with the first session initiated at lights out (1000 h). The animals experienced this DID-MSA procedure five consecutive days each week (Monday–Friday) for 4 weeks beginning at Post-natal day 30 ± 1. On Post-natal day 60 ± 1, animals were euthanized 4 h after removal of the alcohol bottles (1500 h) following the first drinking episode of the day. Animals were euthanized by an overdose of isoflurane inhalation before decapitation. Rat heads were cooled in isopentane on dry ice before removing the brains, which were flash-frozen and stored at −80°C until sectioned.

Frozen brains from four rats per group were cut in half, and one hemisphere of each brain was used for histology. As described previously,^42^ brains were post-fixed in 4% paraformaldehyde for 2 h, then cryoprotected with 30% sucrose for 24 h. After cryoprotection, the brains were frozen at −80°C and then subsequently equilibrated at −20°C in a cryostat microtome and sliced into 30-μm sections. Brain sections were again fixed in 4% paraformaldehyde for 30 min and permeabilized with two 5-min washes in 0.1% Triton X-100 in 1X PBS followed by a 30-min incubation in 3% Triton X-100. Non-specific staining was blocked by a 1-h incubation in Mouse BD Fc Block (BD Pharmingen, cat 5553242; rat anti-mouse CD16/CD32) in non-serum immune buffer (1X PBS containing 0.3% bovine serum albumin, 0.1% sodium azide, 0.3% Triton X-100), followed by 1 h in immune buffer with 0.25% goat serum. Brain sections were stained with anti-dsRNA monoclonal (J2; 1:50; 10010200, Axxora, Farmingdale, NY, USA), mouse anti-RBFOX3/NeuN monoclonal (1B7; 1:100; NBP1-92693, Novus Biologicals, Centennial, CO, USA), rabbit anti-GFAP polyclonal (1:100; PA1-10019, ThermoFisher Scientific, Waltham, MA, USA), chicken anti-Map2 polyclonal (1:100; PA1-10005, ThermoFisher Scientific) and rabbit anti-IBA1 polyclonal (1:100; 019-19741, Wako Chemicals, Richmond, VA, USA) diluted in immune buffer with serum for 72 h at 4°C with rocking. Sections were washed three times for 10 min in PBS + 0.1% Triton X-100 and then incubated with either goat anti-mouse IgG2a Alexa fluor 647, goat anti-mouse IgG2b Alexa fluor 488, goat anti-rabbit Alexa fluor 568, goat anti-mouse IgG Alexa fluor 488 or goat anti-chicken IgY Alexa fluor 647 secondary antibody (1:5000; A21141, A21241, A11011, A11001 and A32933, respectively, ThermoFisher Scientific) and DAPI (62248, ThermoFisher Scientific). Sections were washed as mentioned above, and coverslips were mounted with Vectashield Antifade (H-1000, Vector Labs, Newark, CA, USA) and sealed with clear fingernail polish before analysis.

The rat brain dsRNA immunofluorescence signal for six regions (amygdala, cerebral nuclei, cortex, hippocampus, hypothalamus and thalamus) was captured using a Nikon Eclipse Ti Inverted microscope with plan fluor 20×/1.30 and 100×/1.49 objectives. Images were reconstructed with NIS Elements v4.5 software (Nikon Instruments). The ImageJ Fiji software package V1.53 (NIH) was used to analyse the relative mean pixel intensity of the immunostaining signal from each section as previously described.^43^ Images were converted to black and white, and the entire image density was measured. The background, determined by any area where the signal was not located, was measured and subtracted from the image density. For region of interest (ROI) analysis, the density inside each cell boundary was taken using the ImageJ outline tool. Pearson’s correlation coefficient was determined using NIS Elements V4.5 built-in utility. Significant differences between the alcohol-drinking and alcohol-naïve groups were determined for each ROI by the unpaired Student’s *t*-test using GraphPad Prism 8.

### Single-cell RNA-seq data samples and analysis

Single-nucleus RNA-seq data were generated from *post-mortem* caudate nucleus samples of 142 individuals from the New South Wales BTRC. Nuclei were isolated from fresh-frozen tissue and pooled in condition- and sex-balanced batches prior to library preparation and sequencing. The libraries were prepared from the 10× Genomics Chromium Next GEM Single Cell 3′ HT v3.1 platform and were sequenced on an Illumina NovaSeq 6000 sequencer. RNA library preparation and sequencing were performed at the Center for Medical Genomics (CMG) at Indiana University School of Medicine, Indianapolis. Sequencing data were processed using Cell Ranger based on the GRCh38 (hg38) reference genome.

Demultiplexing, barcode-level quality control and cell type annotation were described in a previous study; gene expression counts were aggregated at the sample level for each cell type using a pseudo bulk approach.^44^ Samples with fewer than 50 cells in a given cell type were excluded. Differential expression analysis between samples with and without AUD was performed using DESeq2, adjusting age, sex and ethnicity as covariates. Statistical significance (*P*-value) was corrected using the Benjamini–Hochberg FDR method; genes with FDR < 0.05 were regarded as differentially expressed. For each cell type, genes were ranked by log_2_ fold-change as input for GSEA performed using the ClusterProfiler R package (v4.0.5). Multiple-hypothesis testing for the enrichment was performed separately for each cell type using FDR.

### Neuronal immunohistochemistry of human brain samples

Additional brain samples from the New South Wales BTRC were used to quantify the neurons in the PFC (18 AUD and 19 controls) and visual cortex (18 AUD and 15 controls). Brain tissue sections from these samples were baked at 63°C for 40 min, briefly cooled to room temperature and then deparaffinized in three changes of xylene for 10 min each. Sections were rehydrated in decreasing ethanol concentrations for 3 min each: 100% three times, 95%, 70%, 50% and then distilled water. Heat-induced epitope retrieval was performed using a Decloaking Chamber NxGen, for 30 min at 110°C in a citrate buffer (pH 6) containing 0.05% Tween-20. Sections were cooled to room temperature and then washed briefly in tris-buffered saline (TBS). Endogenous peroxidase activity was blocked using a solution of 3% H_2_O_2_ in methanol for 20 min. Sections were rinsed twice in distilled water and then washed for 5 min in TBS three times. Tissue sections were then blocked in a solution of 10% normal goat serum in TBS for 30 min. The slides were incubated with rabbit polyclonal NeuN antibody (1:500; ABN78, Merck) prepared in TBS with 0.05% Tween-20 (TBS-T) in a humidity chamber overnight at 4°C.

The brain tissue sections were washed three times in TBS-T for 5 min each with agitation. For immuno-detection, Dako EnVision HRP/DAB+ kit (K5007, Agilent, Mulgrave, Victoria, Australia) was used according to the manufacturer’s instructions. Sections were washed for 5 min in TBS-T three times and then dehydrated in increasing ethanol concentrations and then cleared in xylene three times for 10 min each before mounting in distyrene plasticizer and xylene and cover-slipped. Negative controls were performed by omitting the primary antibody. The experimental group (AUD or control) to which each sample belonged was blinded throughout staining and counting. Tissue sections with poor staining quality were excluded from analysis.

#### Neuronal imaging and quantification

Slides were scanned at 20× magnification using an Aperio XT slide scanner (Leica Biosystems), provided by the Sydney Microscopy and Microanalysis core facility located at the University of Sydney. Image files were kept in their original SVS file format and opened in Orbit Image Analysis, an open-source whole-slide image analysis tool.^45^ Orbit uses context-based structure classification with manual attribution of ROI. The mean area sampled was 32 mm^2^. A few NeuN+ cells were selected within each cortical layer to cover the breadth of size and shape diversity of neurons to train the algorithm. Several background ROI were also included in the model, as background staining was not always homogenous within a section. Next, a large ROI was drawn within the grey matter, from the white–grey matter boundary to the cortical surface, and Orbit was run to count NeuN+ features and calculate the ROI area (µm^2^).

#### Statistical analysis

IBM SPSS Statistics 28 was used to perform ANCOVA on NeuN+ cell counts per mm^2^ between the control and AUD subjects in the PFC and visual cortex, respectively, adjusting for potential brain volume effects. GraphPad Prism 9 was used to graph the results.

### Nanopore long-read RNA-seq in rats

Long-read RNA-seq was performed using separate aliquots from the same RNA samples used for short-read sequencing described above. No RNA remained for four animals; thus, only 12 samples were sequenced (4 CTRL and 8 ALC). The quantity and quality of the remaining RNA samples were re-evaluated using an Agilent TapeStation. The samples had RIN of 7.5–9.0. Full-length cDNA libraries were prepared with the Oxford Nanopore Technologies (ONT) cDNA-PCR Barcoding Kit 24 V14 (SQK-PCB114.24). In brief, 500 ng total RNA per sample was used as input. The cDNA RT adapters were first ligated to the 3′ ends of full-length poly(A) RNA. Next, reverse transcription, strand-switching and PCR with barcoded primers were performed. Barcoded cDNAs were pooled at equimolar ratios for attachment of rapid sequencing adapters. The final libraries were sequenced with PromethION flow cells on an ONT PromethION 24. Two PromethION flow cells were utilized with the same 12 samples on each flow cell. Around 10–12 million reads were generated per sample. Library preparation and sequencing were performed at the Indiana University School of Medicine CMG.

Super accuracy base-calling was performed using Dorado v0.9.1 (https://github.com/nanoporetech/dorado) on the generated pod5 files without adapter trimming and model dna_r10.4.1_e8.2_400bps_sup@v5.0.0. The base-called data were further analysed with the Nanopore wf-transcriptomes with parameters ‘direct_rna: False cdna_kit:SQK-PCB114 pychopper_opts “-U –y”’. Rnor 6.0 reference genome (Ensembl release 104) and the corresponding gene annotation was used for the analysis. Analysis results were visualized using the Integrative Genomics Viewer.^46^

## Results

### Elevated IR in the brain is associated with chronic alcohol drinking and AUD

We first systematically identified IR events in brain samples from individuals with and without AUD. Demographic information for the 142 independent subjects included in this study has been reported previously^9,23^ and is summarized in Supplementary Table 1. Across 320 measurements from four brain regions (SFC, NAC, CE and BLA), we identified a total of 21 341 distinct IR events.

**Figure 1:**
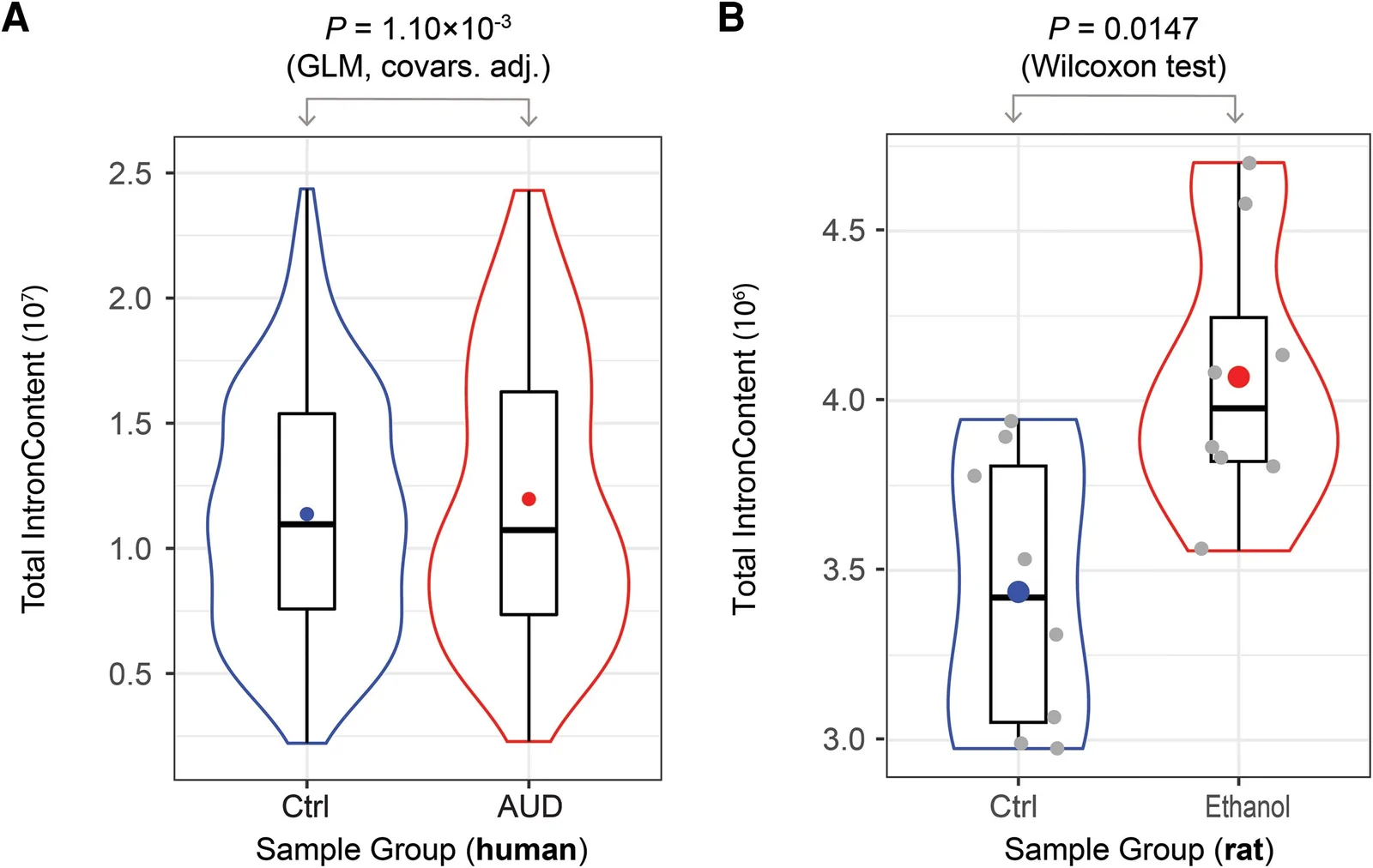
**Elevated IR in AUD brains from human and animal model.** (**A**) Violin with boxplot of the total IC calculated from human brain RNA-seq data for the control (*n* = 165) and AUD (*n* = 155) samples. Mean levels of IC for the control (1.139 × 10^7^) and AUD samples (1.197 × 10^7^) are depicted by round dots. A *P*-value = 0.0254 was obtained from the generalized linear regression model after adjustment for the covariates (covars. adj.) age, sex, RNA-seq cohort, PMI, RIN, brain region and individual difference. Total IntronContent = Sum of all retained intron Length (nt) × Expression (tpm). Metric scale is 10^7^. (**B**) Violin with boxplot of the total IC calculated using RNA-seq data from the selectively bred alcohol-preferring P rat NAC shell. Alcohol-naïve control (*n* = 8) and ethanol-drinking (*n* = 8) male rats were from the same litters. Data points representing the ICs (small dots), along with the mean levels for the control (3.437 × 10^6^) and ethanol (4.070 × 10^6^) groups (large dots), are shown. A *P*-value = 0.0147 was obtained using the Wilcoxon test. IC was calculated as in **A**. The metric scale is 10^6^.

To quantify overall IR levels, we accounted for both intron length and expression by calculating the total IC, i.e. ∑Length×Expression, for each sample. Unlike conventional gene expression analysis, which considers only transcript abundance, this metric captures the cumulative effect of retained introns. We analysed IC within individual brain regions for each study participant, subsequently integrating these findings through a meta-analysis to maximize statistical power. AUD brain samples (*n* = 155 measurements) exhibited significantly higher total IR content than controls (*n* = 165; Fig. 1A) after adjusting for age, sex, sequencing cohort, RIN, PMI, brain region and individual variation (*P* = 1.10 × 10^−3^).

To validate these findings in a controlled animal model, we analysed brain RNA-seq data from selectively bred alcohol-preferring (P) rats. The P rat is a well-established animal model of alcoholism in which chronic voluntary alcohol drinking produces behavioural and neurochemical phenotypes consistent with compulsive drinking.^24,25^ We identified 6132 IR events across all brains from alcohol-drinking (*n* = 8) and alcohol-naïve (*n* = 8) P rats. Total IC was significantly higher in the brains of alcohol-drinking rats compared with their water-drinking littermate controls (*P* = 0.0147; Fig. 1B), confirming that elevated IR is a reproducible feature of chronic alcohol consumption.

In addition, we found that age is also correlated positively with total IC (*P* = 8.02 × 10^−4^, Supplementary Table 2) in the human brain samples, which is consistent with reports of splicing dysregulation during aging.^47-49^ Interestingly, age and AUD acted independently, as their interaction (AUD × age) was non-significant. Furthermore, while sex alone was not associated with IR, a significant AUD × sex interaction was observed (*β*_AUD·sex_ = −0.164, *P* = 0.0437, Supplementary Table 2), indicating that females with AUD displayed higher IR than males. Although this finding was not directly verifiable in the rat cohort as we did not include female P rats in the experiment, it is consistent with evidence from multiple previous studies that alcohol inflicts greater neurobiological harm in females.^50-52^

### AUD-associated aberrant IR events in the human brain

**Figure 2:**
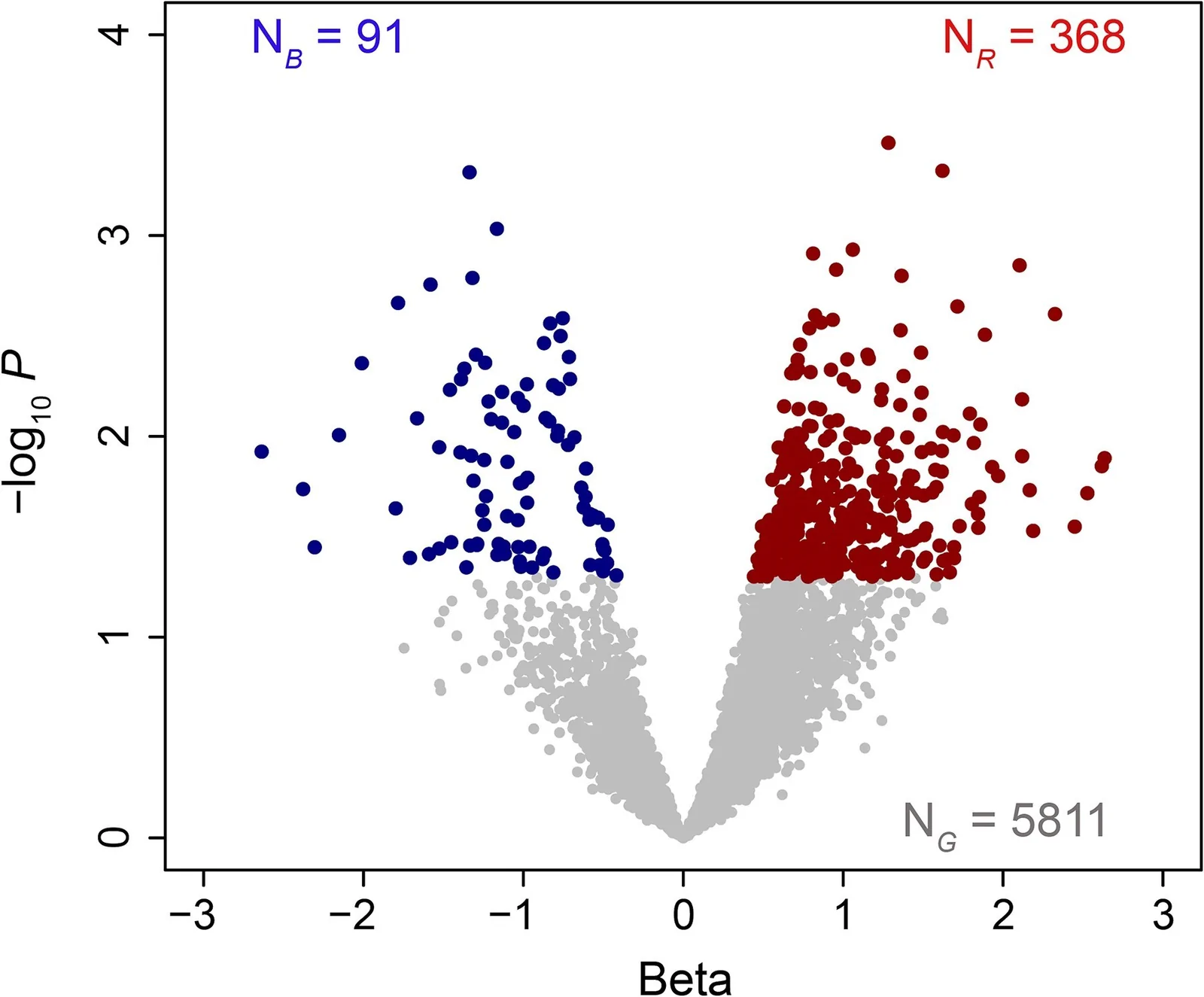
**AUD-associated aIR events.** Volcano plot showing human aIR events with changes in occurrence associated with AUD versus non-AUD. Statistical significance was tested using a generalized estimating equation, adjusting for all covariates. The X-axis shows the effect size (*β),* and the Y-axis shows -log_10_ (*P*-value). Events positively associated with AUD (N_R_) are defined as *β* > 0 and *P*-value < 0.05; events negatively associated with AUD (N_B_) are defined as *β* < 0 and *P*-value < 0.05); and events not significantly associated with AUD (N_G_) are defined as *P*-value ≥0.05.

In addition to the overall IR increase, we next asked whether specific introns were differentially retained in AUD. Because individual brain regions in the human cohorts included no more than 60 individual samples, we integrated data from four regions of two independent RNA-seq cohorts; our analysis accounted for brain region and cohort (nested within sample) as covariates to maximize power. Using a GEE^30^ model to account for repeated brain region measurements within individuals and adjusting for all covariates (mentioned above), we tested 6270 variable IR events (present in ≥10 samples). Of these, 368 were positively associated with AUD (nominal *P*-value < 0.05; Fig. 2). These introns, which we term aIR events, were consistently detected in AUD samples but were absent or rare in controls. An additional 91 events were negatively associated with AUD, yielding 459 total AUD-associated introns (Supplementary Table 3).

Cumulative analysis showed that total IC (∑Length×Expression) derived from these 459 AUD-associated events was significantly greater in AUD samples than controls (*P* = 2.52 × 10^−4^, generalized linear mixed-effect model adjusted for covariates; Supplementary Fig. 2A). Moreover, females exhibited higher aIR content than males (*P* = 3.20 × 10^−3^, Supplementary Fig. 2B), reinforcing a sex-specific vulnerability.^53^ However, no individual aIR event survived multiple-testing correction, suggesting that while overall IR is robustly elevated in AUD, the occurrence of specific introns is more stochastic.

### Replication of IR genes using Nanopore long-read RNA-seq in rats

To validate these predicted IR events in humans, we analysed long-read RNA-seq data from NAC tissue of P rats. We focused on 126 human aIR host genes with rat orthologs (34.2% of the 368 human positively AUD-associated genes) and manually examined their mRNA isoforms, prioritizing those with the most highly expressed introns. For example, in *Ptpn5*, the most highly expressed intron (chr1:103 211 261–103 212 445; Rnor 6.0, Ensembl release 104) was confirmed in long-read alignments, with isoforms containing the entire intron present in both alcohol-drinking and control P rats, though more abundant in the alcohol group (Supplementary Fig. 3A). Similarly, in *Tpm1*, retention of the second-most highly expressed intron in the rat samples (chr8:72 815 589–72 816 613) occurred more frequently in alcohol-drinking rats, reflected by a higher ratio of the intron to its flanking 5′ exon (Supplementary Fig. 3B). Both *Ptpn5* and *Tpm1* have human orthologs (*PTPN5* and *TPM1*) that host AUD-associated aIR events (Supplementary Table 3), supporting cross-species conservation of alcohol-related IR.

### AUD-associated aIR events feature weak splice acceptor sites

**Figure 3:**
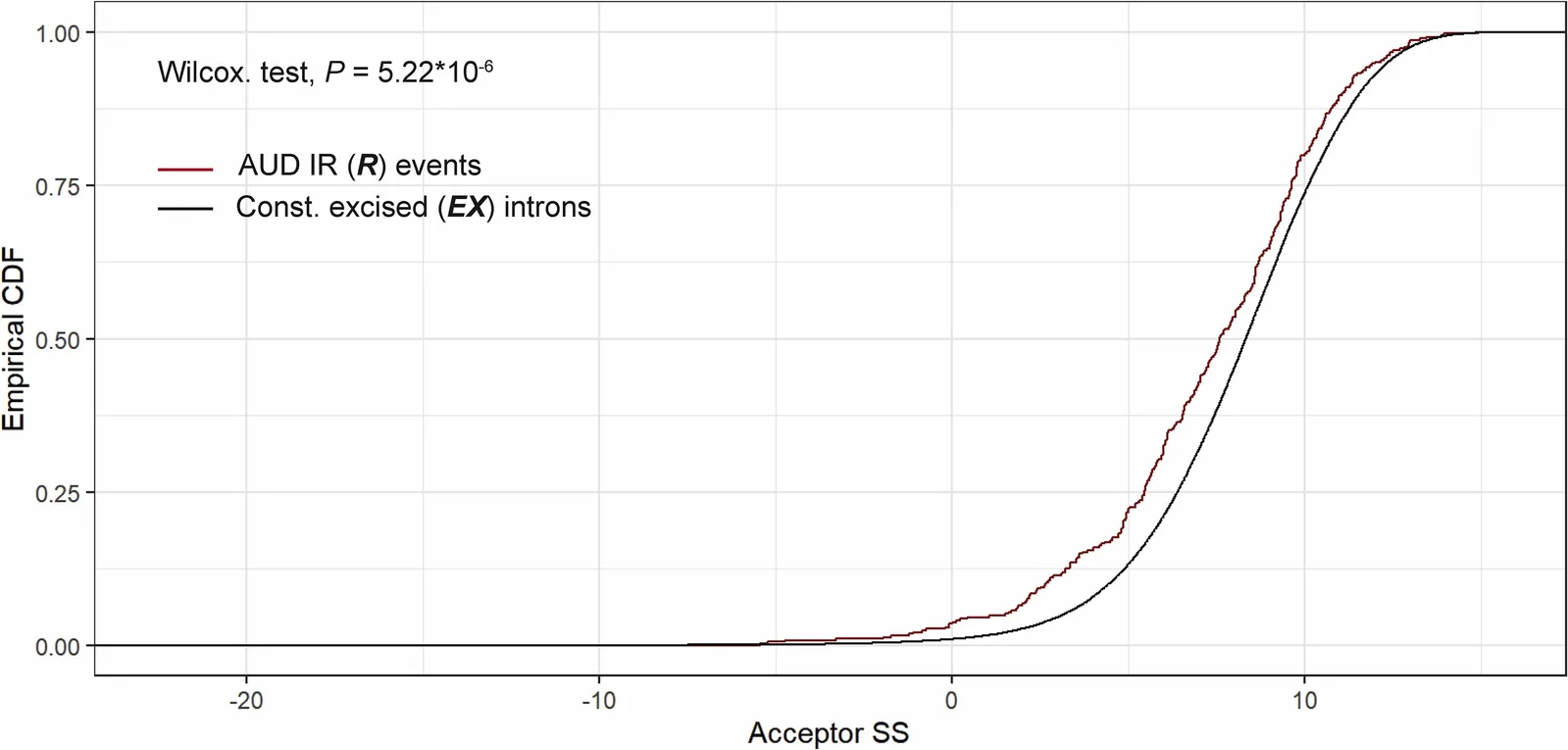
**Splicing code analysis for AUD-associated aIR events.** Empirical cumulative distribution function (CDF) of the 3′-acceptor site splicing scores (SS) of the human AUD-associated aIR events (***R***, red curve; *n* = 368), compared to constitutively excised introns (***EX,*** black curve; *n* = 167 256). The *P*-value was obtained using the Wilcoxon test.

We next investigated whether the human AUD-associated introns displayed reduced splice site strength. Splice site scores at 5′-donor and 3′-acceptor sites were compared between the 368 aIR events and constitutively excised introns. Our analysis revealed that aIR events exhibited significantly weaker 3′-acceptor sites (Fig. 3), whereas no differences were observed at 5′-donor sites (Supplementary Fig. 4). Despite reduced strength, nearly all events (363/368; 98.6%) conformed to the canonical GU-AG splice site motif^54^ (Supplementary Table 3). These results suggest that AUD-associated IR is driven by weakened splice acceptor sites rather than non-canonical splicing.

### Cell type and GO analysis

**Figure 4:**
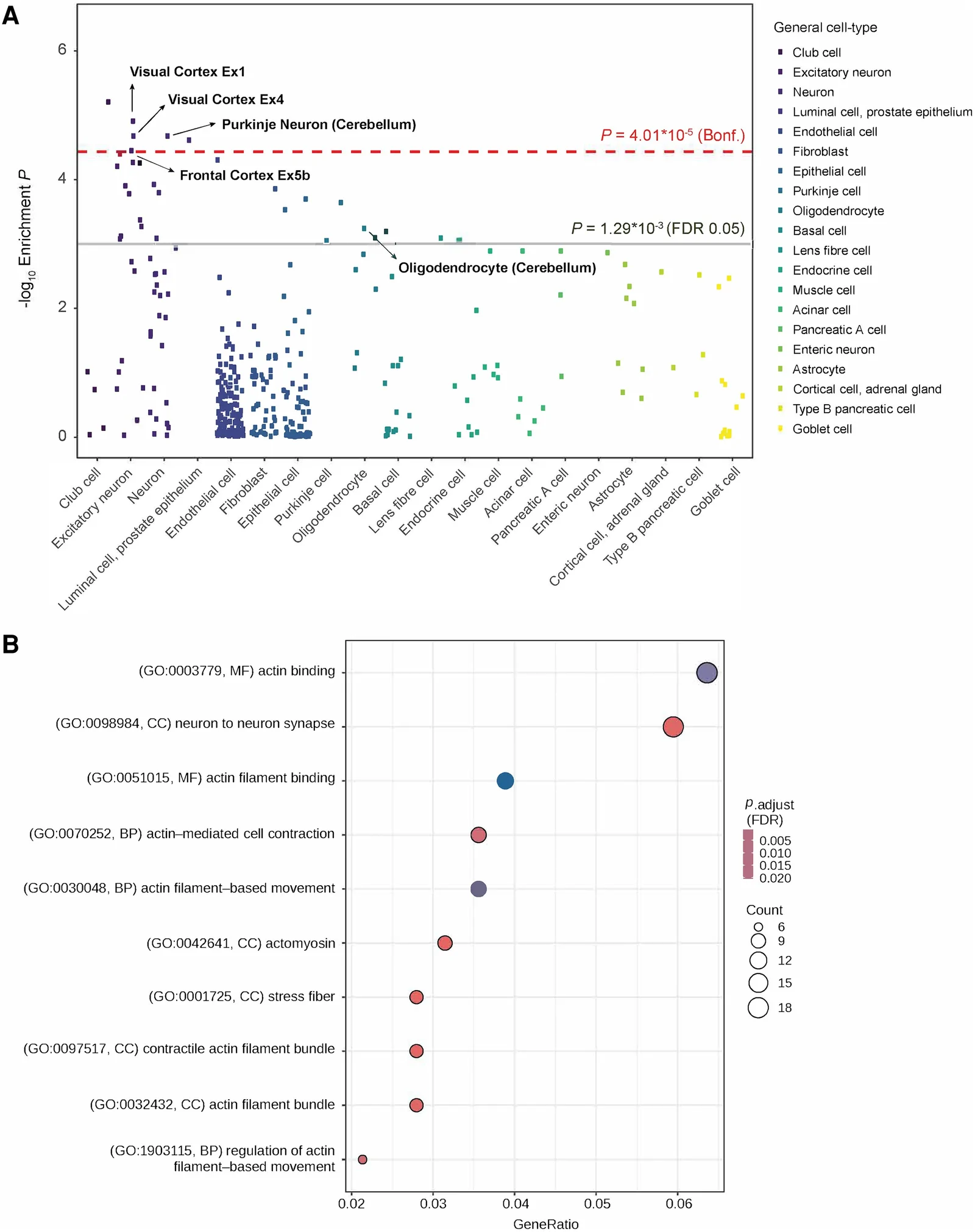
**Cell type enrichment and GO for AUD-associated aIR genes.** (**A**) The genes hosting the human AUD-associated aIR events (*n* = 368) were enriched in multiple neuronal and glial cell types. Enrichment *P*-values were computed using WebCSEA and subject to correction by the FDR and Bonferroni methods, respectively. Solid horizontal line: threshold of significance according to FDR. Dashed horizontal line: threshold of significance according to Bonferroni. (**B**) Enrichment dotplot showing the GO of the human aIR genes. The *P*-value was obtained using ClusterProfiler and adjusted by FDR. Colour gradient corresponds to the *P*-value and dot size corresponds to the gene count. BP: biological process; CC: cellular component; MF: molecular function.

To evaluate whether the human aIR events preferentially affected specific brain cell types, we used WebCSEA.^35^ Genes hosting aIR events were enriched in Purkinje cells, visual cortex neurons and oligodendrocytes (Fig. 4A and Supplementary Table 4). These cell types play critical roles in motor coordination, sensory processing and neuroimmune regulation.^55,56^

GO analysis revealed enrichment of functions related to synaptic activity, axon initial segment organization, ion channel regulation, calcium transport, cell junction assembly and actin filament dynamics (Fig. 4B and Supplementary Table 5). These enrichments suggest that aIR may disrupt neuronal signalling, cell–cell communication and cytoskeletal integrity, thereby contributing to AUD-associated pathology.

### Aberrantly retained introns are associated with greater length and dsRNA

**Figure 5:**
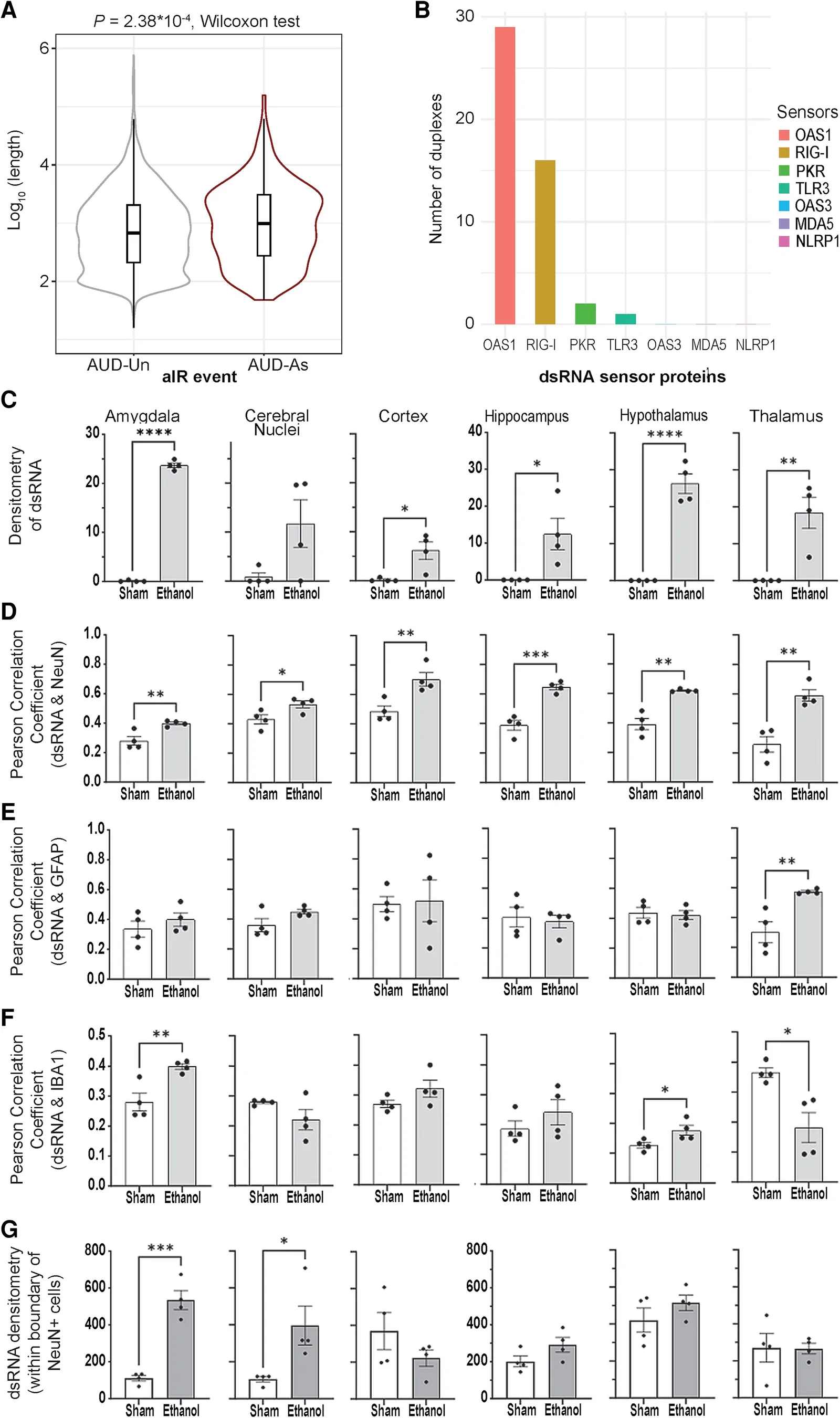
**Association of aIR with dsRNA and co-localization of dsRNA with specific cell types.** (**A**) Violin with boxplot comparing the intron lengths (in 10-base logarithm) between the human AUD-associated aIR events (AUD-As, *n* = 368) and events having no association to AUD (AUD-Un, *n* = 5811). Wilcoxon test (two-sided) was used to calculate the *P*-value. (**B**) Barplot showing the total numbers of duplexes with the preferred sizes for various dsRNA sensor proteins, which were predicted from 288 human AUD-associated retained introns using SPOT-RNA. Preferred duplex length: OAS1 (<20 bp), RIG-I (∼22–500 bp), PKR (≥33 bp), TLR3 (40–50 bp), OAS3 (>50 bp), MDA5 (∼500–1000 bp) and NLRP1 (>500 bp). (**C**) Barplots of dsRNA densitometry measurements are shown based on immunofluorescence microscopy images of the brain sections from alcohol-preferring P rats, with four animals per group. Data points: dsRNA immunostaining signal levels. Sham: control; Ethanol: ethanol-treated. *P*-values were calculated using the unpaired *t*-test. *P < 0.1; **P < 0.01; ****P < 0.0001. (**D–F**) Pearson correlation coefficient (data point) between dsRNA and the respective cell type–specific markers NeuN (neuron nuclei, **D**), GFAP (astrocytes, **E**) and IBA1 (microglia, **F**), based on the brain samples of P rats. (**G**) dsRNA densitometry within the boundary of NeuN+ cells, based on the brain samples of P rats. Data points: dsRNA signal levels (within NeuN+ cells). Sham: control; Ethanol: ethanol-treated. *P*-values were calculated using unpaired *t*-test. *P < 0.1; **P < 0.01; ***P < 0.001; ****P < 0.0001.

The human AUD-associated introns were significantly longer than non-AUD introns (Wilcoxon *P* = 2.38 × 10^−4^, Fig. 5A), consistent with evidence that longer introns are more prone to retention.^57,58^ In addition, long introns have higher potential to form duplex structures. Therefore, we hypothesized that they could generate dsRNA, which might trigger an antiviral response. We used SPOT-RNA,^38^ a deep learning tool, to predict the dsRNA-forming potential for the AUD-associated IR events. We selected 288 of the 368 IR events using the following two criteria: (i) the host gene must be a signature gene for at least one brain cell type (WebCSEA, FDR < 0.05); and (ii) intron lengths must not exceed 10 kb as per the technical limitation of SPOT-RNA. Of these 288 analysable IR events, predictions showed duplexes up to 44 base pairs, within the recognition range of dsRNA sensors such as OAS1, RIG-I, PKR and Toll-like receptor 3 (TLR3), though not for sensors like MDA5, NLRP1 or OAS3^40^ (Fig. 5B). Twenty-one ultra-long introns (>10 kb) could not be processed by SPOT-RNA but may form additional dsRNA structures. Collectively, these results demonstrate that AUD-associated introns are structurally capable of forming dsRNA.

To observe whether dsRNA was present in brain tissue, we performed immunofluorescent staining with a dsRNA-specific antibody^59^ using an animal model, as human samples from the New South Wales BTRC were restricted to RNA-seq data and unavailable for immunohistochemistry analysis. The results revealed significantly elevated dsRNA signals across six brain regions in alcohol-consuming P rats compared to alcohol-naïve controls (Fig. 5C and Supplementary Fig. 5A).

### Co-localization of dsRNA with neuronal cells

We next sought to determine the cell type–specific localization of dsRNA to identify whether it preferentially accumulates in neuronal or glial populations. Strong co-localization was observed between dsRNA and NeuN+ neuronal nuclei across all six brain regions in alcohol-consuming P rats compared with alcohol-naïve controls (Fig. 5D and Supplementary Fig. 5A). In contrast, co-localization with GFAP+ astrocytes and IBA1+ microglia was brain region–specific (Fig. 5E and F; Supplementary Fig. 5A). ROI densitometry revealed significantly elevated dsRNA signal within NeuN+ neurons of the amygdala and cerebral nuclei in alcohol-consuming P rats relative to controls (Fig. 5G). In addition, increased co-occurrence between dsRNA and MAP2+ dendrites was observed in the cerebral nuclei and hypothalamus of alcohol-drinking rats (Supplementary Fig. 5B). These findings indicate that dsRNA predominantly accumulates in neuronal nuclei and dendrites, highlighting neurons as primary sites of alcohol-induced dsRNA formation.

### Conserved dsRNA response and neuroinflammatory signalling across human and rat models

**Figure 6:**
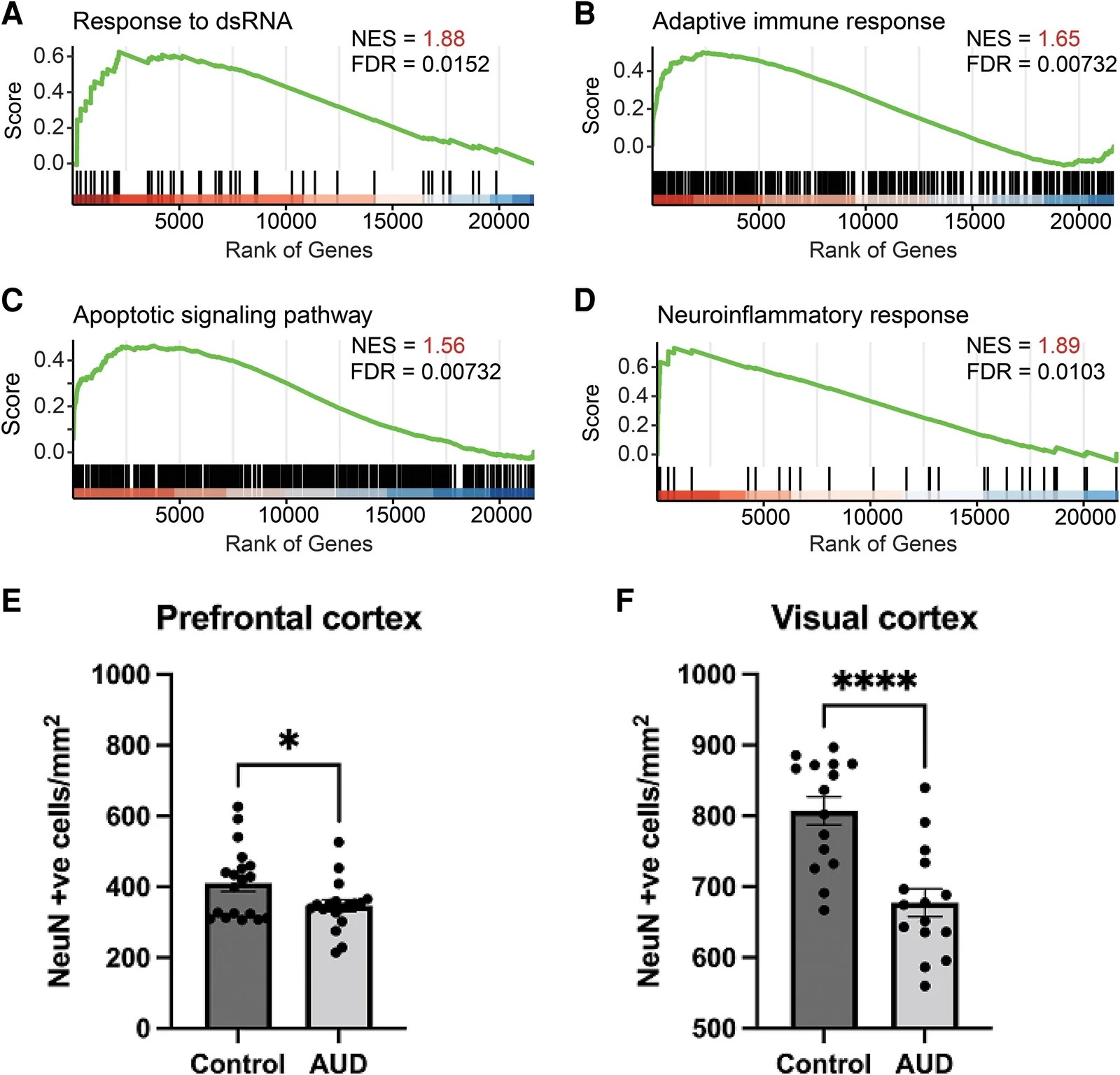
**Pathway activation and neuronal loss in AUD.** GSEA enrichment plots using human brain RNA-seq data from individuals with and without AUD (320 measured samples from 142 individuals, resulting in a total of 21 630 genes). NES > 0 and FDR < 0.05 indicated activation of the dsRNA response (**A**), adaptive immune response (**B**), apoptotic signalling (**C**) and neuroinflammation (**D**) pathways in the AUD brain. (**E** and **F**) Barplots showing that the NeuN+ cell counts per mm^2^ (mean ± SEM) in the PFC (**E**, *P* = 0.0328) and visual cortex (**F**, *P* < 0.0001) in the AUD brains compared to the controls. Data points: NeuN+ cell counts (per mm^2^). The *P*-value was obtained using ANCOVA, adjusting for potential brain volume effects.

Differential expression analysis of the human RNA-seq dataset identified 798 genes across all brain regions that were altered in AUD (549 upregulated and 249 downregulated, FDR < 0.05; Supplementary Table 6). GSEA showed activation of pathways associated with the dsRNA response, adaptive immunity, apoptosis and neuroinflammation (Fig. 6A–D and Supplementary Table 7). Canonical neuroimmune pathways, including JAK-STAT, NIK/NF-κB, TNF, and type I/II interferon (IFN) signalling, were consistently upregulated (Supplementary Table 7). These findings suggest a link between dsRNA accumulation and immune activation in AUD.

Interestingly, as a supplementary analysis, the NAC RNA-seq of the well-controlled P rats indicated that as many as 6428 genes might be differential expressed (3270 upregulated and 3158 downregulated, FDR < 0.05; Supplementary Table 8). The relatively lower number of differentially expressed genes in human samples likely reflects the inherent heterogeneity of *post-mortem* data, whereas the well-controlled nature of the rat experiments allowed for more robust statistical detection. GSEA using the rat samples showed strong upregulation for the negative regulation of gliogenesis and glial cell differentiation. This signals the typical alcohol neurobiology for the reduced development of central nervous system. In addition, synapse assembly, organization and activity were clearly enriched, echoing glial inflammatory signalling that often involves alcohol-induced synaptic changes (Supplementary Table 9).^60,61^ Notably, we observed apparent upregulation of multiple rat dsRNA-sensor and target genes. For example, *Ifit1* is a canonical dsRNA-induced IFN-stimulated gene, which is a downstream target of TLR3, RIG-I and MDA5. Other examples include *Irf7*, which is the ortholog of human *IRF7*, a downstream target of TLR3; *Ifit3*, another dsRNA-induced IFN-stimulated gene, part of the IFIT complex downstream of IRF3/IRF7 activation; *Stat1* and *Mx1*, IFN-response genes induced by dsRNA-triggered interferon (e.g. type I IFN) signalling; and direct dsRNA sensors or sensing regulators such as *Oas1a, Ddx58* (RIG-I), *Ifih1* (MDA5), *Eif2ak2* (PKR) and *Dhx58* (LGP2) (Supplementary Table 8). These findings reaffirmed that the dsRNA-associated mechanism was conserved across human and rat in response to alcohol exposure.

### Neuronal cell loss in the human brain

To investigate the downstream consequences of neuroinflammation, we quantified neuronal density using an independent human sample set from the New South Wales BTRC. Given that chronic neuroinflammation and apoptosis are known drivers of cellular attrition,^62^ we measured NeuN+ cell population in the PFC and visual cortex. Both regions showed significantly reduced neuronal counts compared with controls (Fig. 6E and F), with the greatest loss in the visual cortex where aIR host genes were enriched (Fig. 4A).

### Cell type–specific activation of dsRNA-sensor targets and inflammatory pathways

To elucidate the mechanistic link between dsRNA and neuroinflammation in human brains, we analysed single-cell RNA-seq data from the caudate nucleus of 142 individuals in the New South Wales BTRC cohort, as described in our recent publication.^44^ With a depth of approximately 8000 cells per sample, this high-resolution dataset allowed us to assess whether the downstream targets of dsRNA-sensor proteins and their associated inflammatory pathways were preferentially activated within specific cell populations.

**Figure 7:**
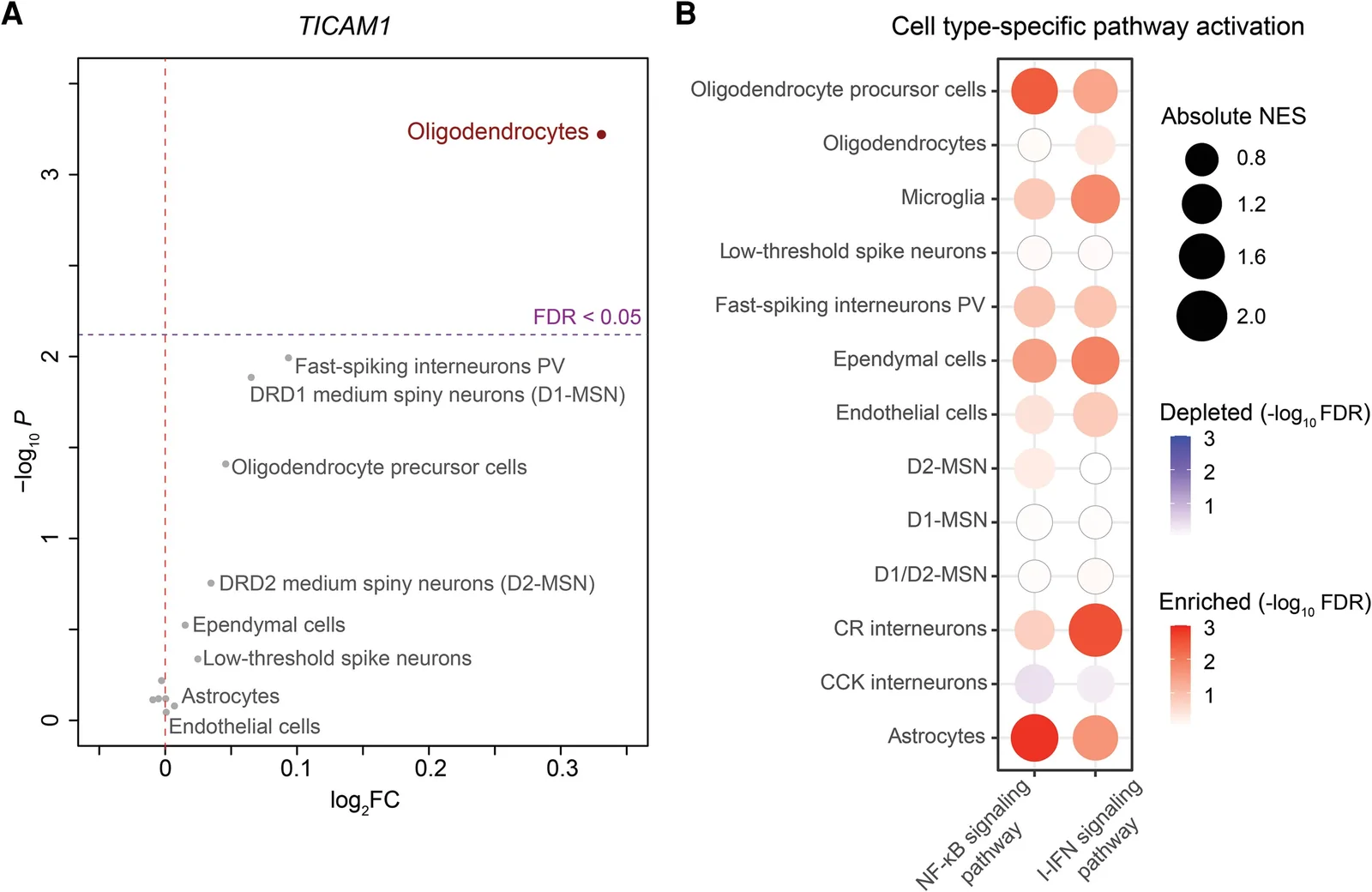
**Cell type–specific gene and pathway activation in AUD caudate.** Gene expression and pathway enrichment plots using human caudate scRNA-seq data from individuals with and without AUD. Statistical tests for differentially expressed genes and enriched pathways were performed using DESeq2 (adjusting for all covariates) and GSEA, respectively, based on 142 sample subjects providing ∼8000 cells each. The exact number of cells and total genes for each cell type were listed in the study by Green *et al*.^44^ The *P*-values were corrected using FDR. Log_2_ FC > 0 and FDR < 0.05 indicated upregulation of *TICAM1* (**A**). NES > 0, e.g. Enriched, and FDR < 0.05 (−log_10_ FDR > 1.30) indicated the activation of the NF-κB and type I IFN (1-IFN) signalling pathways (**B**), which are downstream regulatory targets of *TICAM1*, in the AUD caudate nucleus. PV: parvalbumin; DRD1 or DRD2: dopamine receptor D1 or D2; CR: calretinin; CCK: cholecystokinin.

To identify the molecular transducers of the dsRNA response, we examined the TLR3-TICAM1 axis, a canonical pathway for dsRNA-sensing. TICAM1 (also known as TRIF), the essential adapter protein for TLR3, showed a broad trend towards upregulation across nearly all cell types (Fig. 7A). While the most robust induction was observed in oligodendrocytes, the consistent rightward shift of other populations, including MSNs and interneurons, suggests a widespread activation of this adapter.

Consistent with activated *TICAM1* expression, downstream NF-κB and type I IFN signalling were significantly upregulated across multiple cell types, including astrocytes, microglia and oligodendrocyte-lineage cells (Fig. 7B). Inflammatory mediators (*REL*, *RELB* and *NFKB2)* were highly expressed in oligodendrocytes, astrocytes and dopamine receptor–defined medium spiny neurons (DRD1- and DRD2-MSNs) (Supplementary Fig. 6 and Supplementary Table 10).

Adaptive immune and apoptotic pathways were also consistently activated in astrocytes, microglia, oligodendrocyte-lineage cells, ependymal cells, CR interneurons and DRD1- and DRD2-MSNs (Supplementary Fig. 6). Notably, *OAS1*, another dsRNA sensor, was significantly upregulated specifically in oligodendrocytes (Supplementary Fig. 6), implicating them as a potential initiating cell type.

Together, these results demonstrate that dsRNA-sensing and downstream inflammatory pathways are broadly activated in the AUD brain, with oligodendrocytes appearing to be an initiator of the response. Activation of inflammatory pathways seemingly propagates from oligodendrocytes to neighbouring glial and neuronal populations, which may ultimately contribute to neuronal loss.

## Discussion

In this study, we systematically investigated IR in the *post-mortem* brain transcriptome of individuals with and without AUD. Our key finding is that overall IR is elevated in the brains of those with AUD, independent of age, providing further evidence that AUD is associated with global alteration and dysregulation of RNA splicing.^9,63^ Importantly, higher IR was observed in neuronal and glial populations implicated in AUD, including GABAergic regulatory cells, visual cortex neurons and oligodendrocytes.^64-66^ Immunofluorescent staining confirmed higher dsRNA levels in the brains of alcohol-consuming rats, which co-localized with neuronal markers. At the molecular level, single-cell analysis on *post-mortem* brain tissues demonstrated that dsRNA-sensor targets and downstream inflammatory pathways were activated in multiple cell types from individuals with AUD. Our results provide supporting evidence for a mechanism in which IR produces dsRNA that triggers immune signalling, apoptosis and neuroinflammation, ultimately contributing to neuronal loss in AUD.

RNA splicing is increasingly recognized as a key regulator of gene expression and a driver of complex diseases.^9,17,18,67^ Our analysis expands prior work by systematically examining both alternatively retained and aIR events in the context of AUD. While regulated IR fine-tunes transcriptomes through mechanisms such as non-sense-mediated mRNA decay, nuclear sequestration and turnover of transcripts with alternatively retained introns,^12^ aberrant IR arises from dysregulated splicing and is linked to pathological outcomes. For example, aIR can generate neo-antigens that modulate immune checkpoints in cancer,^12,40^ and spliceosome inhibition has been shown to induce aIR events that form endogenous dsRNAs in breast cancer.^12^ These dsRNAs, once bound by sensor proteins such as the OAS, RIG-1 and TLR families, activate innate antiviral responses, adaptive immunity and apoptosis.^12,40^ Despite these insights, the role of aIR in neuropsychiatric conditions has remained poorly understood, due in part to the limited availability of large-scale, high-depth brain transcriptomic data and computational tools capable of systematically detecting both alternative and aberrant IR events. Thus, our findings extend this knowledge by demonstrating that aIR correlates to dsRNA formation and neuroinflammation in the human AUD brain.

We observed widespread splicing dysregulation in AUD brains, with significantly more aberrantly retained introns than in controls. Elevated dsRNA was validated in brain tissue from a well-characterized rat model of alcohol abuse, further supporting cross-species conservation of this phenomenon. Mechanistically, single-cell analyses highlighted upregulation of *TICAM1*, the canonical regulatory target of TLR3, specifically in oligodendrocytes. This provides strong evidence that the TLR3-TICAM1 dsRNA-sensing pathway is active in AUD. Furthermore, NF-κB, interferon signalling, adaptive immune pathways and apoptotic cascades were consistently upregulated in multiple cell types. Together, these data confirm that dsRNA responses are engaged in the AUD brain and contribute to downstream neuroinflammatory and neuroimmune activity.

AUD-associated introns exhibited significantly weaker splice acceptor sites, although the majority still conformed to canonical GU-AG motifs. This suggests that alcohol-related splicing dysregulation does not arise from non-canonical splice site usage but rather from stochastic dysregulation of weaker, more vulnerable sites. This mechanistic nuance highlights how environmental perturbations such as alcohol exposure may destabilize splicing at specific loci.

Genes containing aIR events were enriched in multiple neuronal and glial cell populations. In particular, visual cortex neurons were enormously affected, consistent with prior studies linking the visual cortex to alcohol-induced plasticity and addiction behaviours.^68-71^ Visual cortical neurons also showed significant loss in AUD, aligning molecular dysregulation with structural pathology. At the functional level, enrichment of actin- and filament-related pathways among aIR host genes suggests a mechanistic basis for alcohol-associated impairments in vision and neurotransmission.

Purkinje cells, key GABAergic neurons critical for motor coordination, learning and balance, also exhibited strong enrichment of aIR host genes. This finding offers a new perspective on how splicing dysregulation may underlie alcohol-related motor and cognitive deficits.

Oligodendrocytes emerged as a particularly important cell type, both through enrichment analyses and single-cell data showing initiation of dsRNA responses. Oligodendrocytes provide myelination and metabolic support but are also increasingly recognized as active contributors to neuroinflammation in neurodegenerative diseases.^72-74^ Our data suggest that aIR-associated molecular actions in oligodendrocytes may propagate inflammatory responses to astrocytes, microglia and neurons, consistent with previous observations that oligodendrocyte signalling states can shape the neuroimmune environment of the brain.^75^ Thus, in addition to astrocytes and microglia, oligodendrocytes play a central role in AUD-induced neuroinflammation.

There are several limitations in this study. The sample size may restrict power to detect individual aIR events that withstand stringent multiple-testing correction. Furthermore, functional validation of specific aIR events is technically challenging due to their length and environmental sensitivity. To mitigate these challenges, we combined *post-mortem* human brain RNA-seq data with a well-established rat model of alcoholism, which provided orthogonal validation and mechanistic insights. In addition, nearly all human samples were of European ancestry. However, the observation of cross-species IR elevation suggests that these mechanisms are unlikely to be ancestry-specific.

In this study, we integrated multiple complementary approaches, including short- and long-read transcriptomics, single-cell analyses and animal models. This multi-modal framework allowed us to explore how chronic alcohol exposure dysregulates splicing and affects neuroimmune signalling. Although the statistical methods in the present study are mainly exploratory, which cannot directly test the causality between alcohol and IR in human *post-mortem* brain data alone, the animal models served as an approach to compare alcohol-exposed and control subjects directly, enabling a well-controlled assessment for the impact of alcohol consumption on IR. The consistency of major observations across these independent systems provides a compelling support for the robustness of the relationship that chronic alcohol exposure induces elevated IR in the brain.

While providing a direct link between IR and dsRNA formation is beyond the scope of the study, evidence for IR-induced dsRNA formation had been demonstrated experimentally using spliceosome inhibitors.^12^ Moreover, RNA secondary structure analysis showed that the aIR events had high potential of forming dsRNA, and single-cell data revealed that downstream pathways mediated by dsRNA were activated in multiple cell types.

Our results align with previous studies showing that IR increases with aging^69,76^ but extend this by demonstrating that AUD independently elevates IR, suggesting a novel insight regarding alcohol exposure and aging at the level of splicing dysregulation. This raises the possibility that chronic alcohol consumption may accelerate aging-related transcriptomic changes. In addition, our findings reinforce sex-dependent vulnerability, as females exhibited higher levels of IR under alcohol exposure. This observation is consistent with prior reports on human subjects that alcohol inflicts greater physiological harm in women,^53^ and thus provides supporting molecular-level evidence from the RNA splicing perspective.

In conclusion, we provide the first comprehensive evidence that aberrant IR in AUD may lead to dsRNA formation, which in turn activates neuroinflammatory, immune and apoptotic pathways across multiple cell types. These processes converge on neuronal loss and neurodegeneration, offering novel mechanistic insights into how chronic alcohol use damages the brain at the molecular level. Our findings highlight RNA splicing as a previously underappreciated contributor of AUD-associated neuropathology and suggest that targeting splicing dysregulation or dsRNA-sensing may represent new therapeutic avenues for AUD.

## Supplementary Material

fcag264_Supplementary_Data

## Data Availability

Human brain sample data are available via the NCBI BioProject database: BLA (PRJNA551909): https://www.ncbi.nlm.nih.gov/bioproject/?term=PRJNA551909, CE (PRJNA551908): https://www.ncbi.nlm.nih.gov/bioproject/?term=PRJNA551908, NAC (PRJNA551775): https://www.ncbi.nlm.nih.gov/bioproject/?term=PRJNA551775, SFC (PRJNA530758): https://www.ncbi.nlm.nih.gov/bioproject/?term=PRJNA530758 and PFC (PRJNA781630): https://www.ncbi.nlm.nih.gov/bioproject/?term=PRJNA781630. The Python tool IR-NeoAg is available via GitHub, https://github.com/cpdong/IntronNeoantigen. WebCSEA is available at https://bioinfo.uth.edu/webcsea/. The R package clusterProfiler is available via Bioconductor, https://bioconductor.org/packages/release/bioc/html/clusterProfiler.html. SPOT-RNA is available via GitHub, https://github.com/jaswindersingh2/SPOT-RNA. The RNA secondary structure tool bpRNA is available via GitHub, https://github.com/hendrixlab/bpRNA. This study did not generate any new code.
