# Supplementary material for: Elevated intron retention implicates neuroinflammation in brains of individuals with alcohol use disorder
Source: Brain Commun. 2026 Jul 29;8(5):fcag264. doi: 10.1093/braincomms/fcag264 (PMC13532580; doi:10.1093/braincomms/fcag264)
Supplement: Supplementary file 1 [file fcag264_supplementary_data.pdf]

Supplementary Materials

Author contributions

Conceptualization, R.L. and Y.L.; Methodology, R.L., S.K.W-J., C.D., J.L.R., N.G., H.G. and R.B.; Investigation, R.L., J.L.R., S.K.W-J., R.L.B. and S.R.H.; Formal Analysis, R.L.; Validation, R.L., J.L.R., N.G., H.G. and S.K.W-J.; Resources, G.T.S., J.S., R.D.M., M.K., A.G., J.L.R., R.L.B., R.B., Y.L. and Y.W.; Data Curation, R.L., C.D., G.T.S., J.S., N.G., H.G., R.D.M., M.K. and A.G.; Writing – Original Draft, R.L., J.L.R., S.K.W-J., R.B. and Y.L.; Writing – Review & Editing, R.L., J.L.R., S.K.W-J., C.D., G.T.S., J.S., N.G., H.G., R.D.M., M.K., A.G., H.J.E., R.L.B., S.R.H., R.B., Y.L. and Y.W.; Visualization, R.L. and S.K.W-J.; Supervision, R.B. and Y.L.; Project Administration, G.T.S., R.D.M., A.G. and Y.L.; Funding Acquisition, S.K.W-J., G.T.S., R.D.M., A.G., H.J.E., J.L.R., R.L.B, R.B. and Y.L.

Supplementary Figure 1

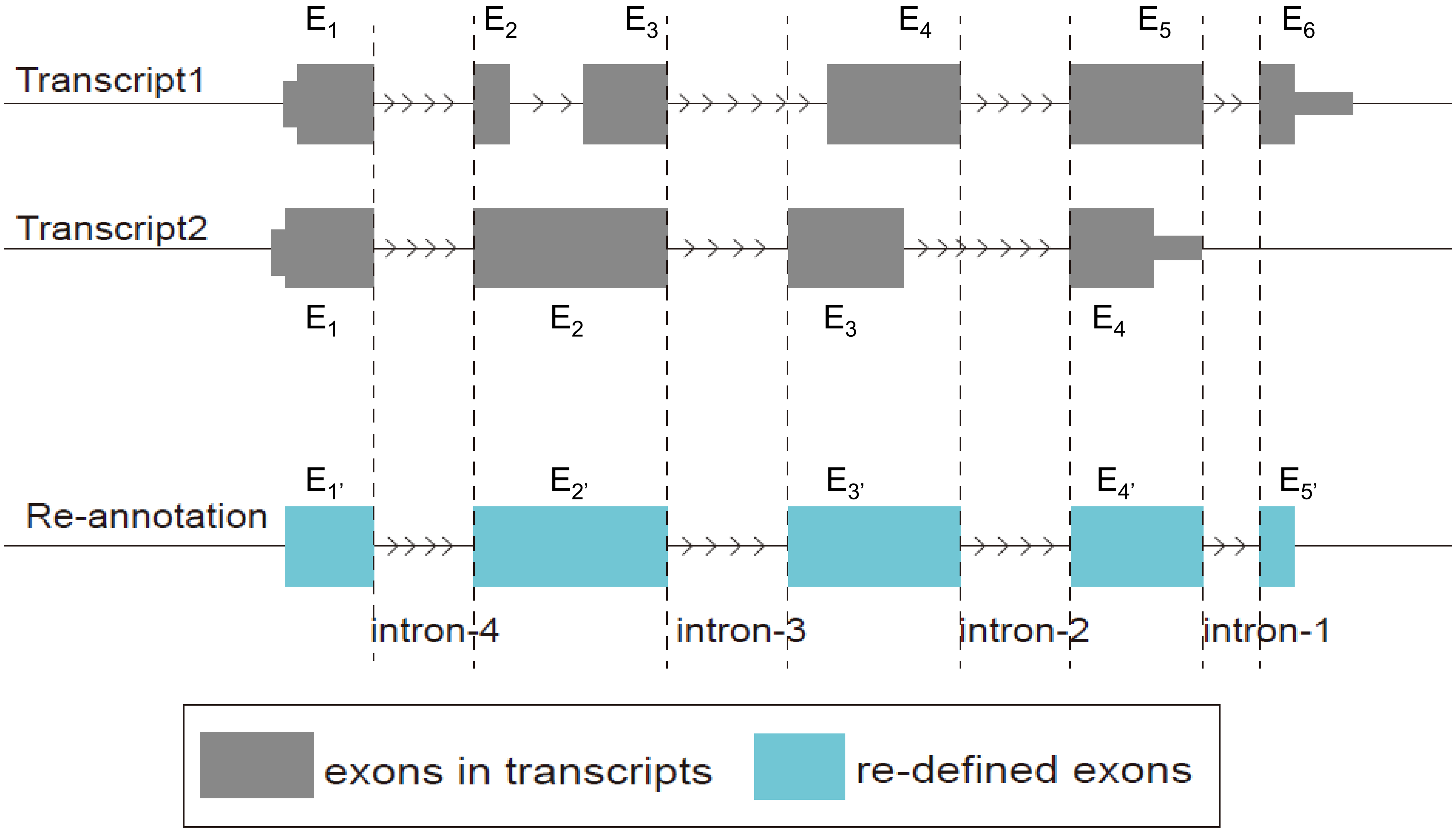

Supplementary Figure 1 – Illustrative diagram of exon and intron re-annotation in the IR identification approach.

The exon sets for a given gene were re-annotated by taking the union of each exon set corresponding to a transcript. The intron regions were defined as the interval between the re-defined exon sets.

Supplementary Figure 2

(A)

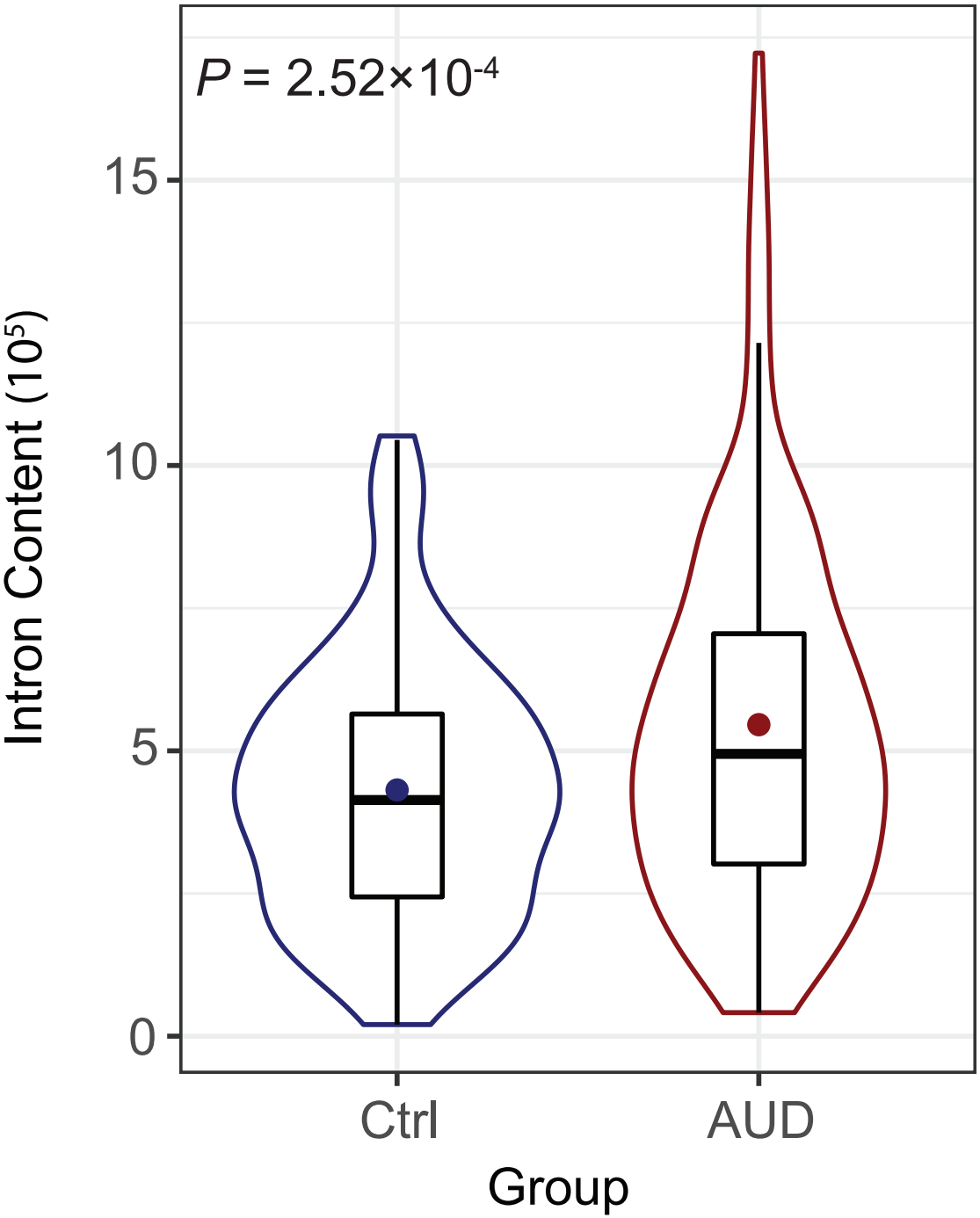

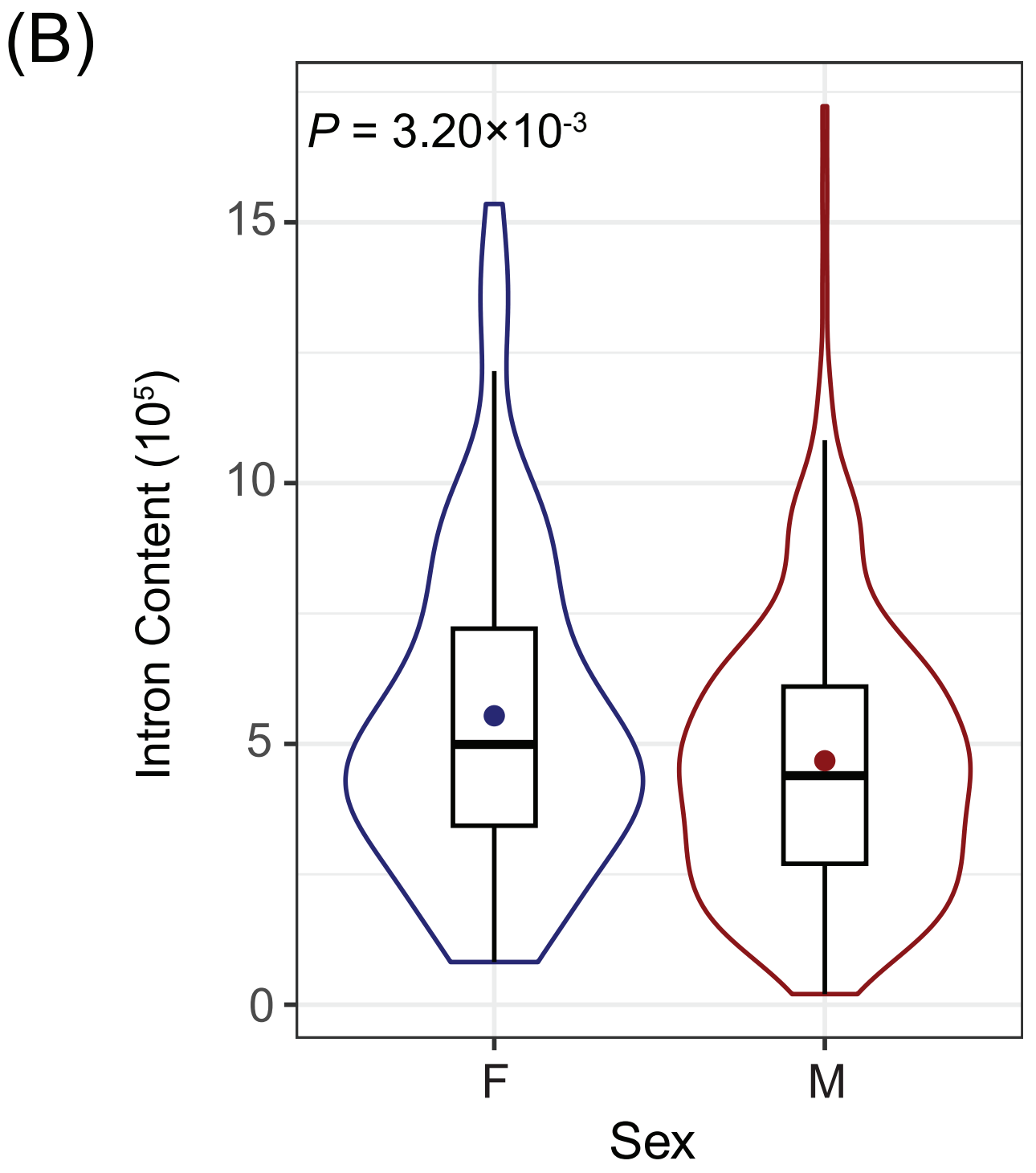

(B)
P = 3.20×10-3
Intron Content (10^5)
15
10
5
0
F
M
Sex

Supplementary Figure 2 – Intron content of AUD-associated IR events.

(A) Violin with boxplot of the intron content calculated based on only the positively (n=368) and negatively (n=91) AUD associated IR events, in the individuals with and without AUD. Mean levels of intron content are shown by the blue (in control group) and red (in AUD group) dots. A *p*-value = 8.5×10^{-5} was obtained from a generalized linear regression model having adjusted for the covariates sex, age, RNA-seq cohort, brain region, and individual difference. Metric scale is 10^5.

(B) Violin with boxplot of the intron content in male and female sample subjects, calculated based on the AUD-associated IR events. Mean levels are shown by the blue (in female group) and red (in male group) dots. A *p*-value = 3.2×10^{-3} was obtained using the generalized linear mixed-effect model described in *Materials and methods*. Metric scale is 10^5.

Supplementary Figure 3

(A) *Ptpn5*

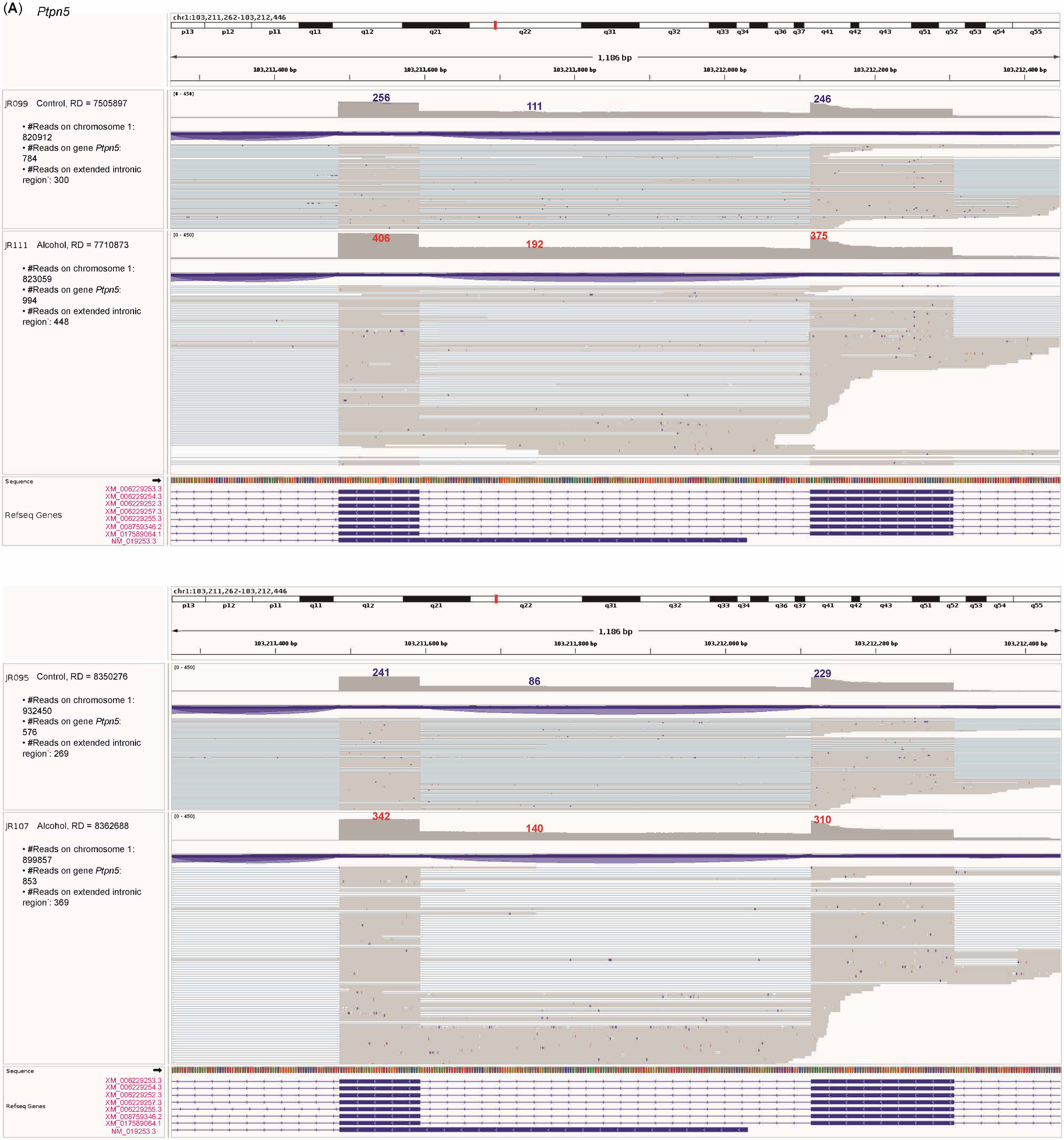

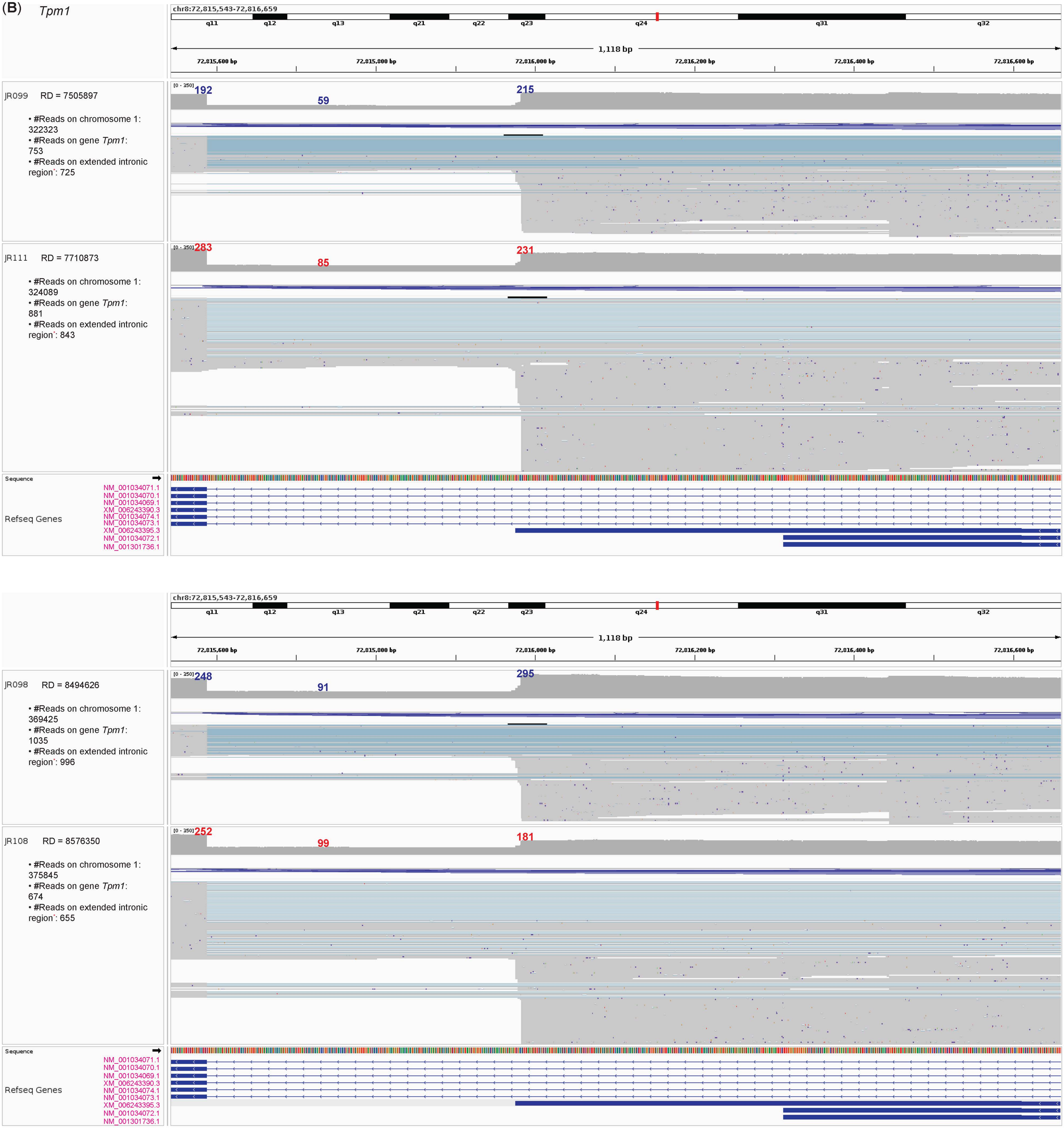

(B)
Tpm1
chr8:72,815,543-72,816,659
q11
q12
q13
q21
q22
q23
q24
q31
q32
1,118 bp
72,815,600 bp
72,815,800 bp
72,816,000 bp
72,816,200 bp
72,816,400 bp
72,816,600 bp
JR099 RD = 7505897
• #Reads on chromosome 1: 322323
• #Reads on gene Tpm1: 753
• #Reads on extended intronic region*: 725
[0 - 250]
192
59
215
JR111 RD = 7710873
• #Reads on chromosome 1: 324089
• #Reads on gene Tpm1: 881
• #Reads on extended intronic region*: 843
[0 - 250]
283
85
231
Sequence
Refseq Genes
NM_001034071.1
NM_001034070.1
NM_001034069.1
XM_006243390.3
NM_001034074.1
NM_001034073.1
XM_006243395.3
NM_001034072.1
NM_001301736.1
JR098 RD = 8494626
• #Reads on chromosome 1: 369425
• #Reads on gene Tpm1: 1035
• #Reads on extended intronic region*: 996
[0 - 250]
248
91
295
JR108 RD = 8576350
• #Reads on chromosome 1: 375845
• #Reads on gene Tpm1: 674
• #Reads on extended intronic region*: 655
[0 - 250]
252
99
181

Supplementary Figure 3 – Nanopore long-read RNA sequencing results of rat nucleus accumbens (example).

IGV track views of the long-read RNA-seq alignment results: intron regions chr1:103,211,261-103,212,445 in gene *Ptpn5* (ENSRNOG00000013981; **A**) and chr8:72,815,589-72,816,613 in gene *Tpm1* (ENSRNOG00000018184; **B**). The tracks include coverage, junction, aligned reads, and reference gene transcripts (top to bottom). The inset numbers indicate the (peak) read counts of the 5' exon, intron (of interest), and 3' exon, respectively, for the IR events. Expression of the retained intron was exemplified by pairs of samples that have similar read depth (RD). Both genes are on the reverse strand (-). The extended intronic region refers to the intron plus the flanking exons, i.e., 5' exon + intron + 3' exon.

Supplementary Figure 4

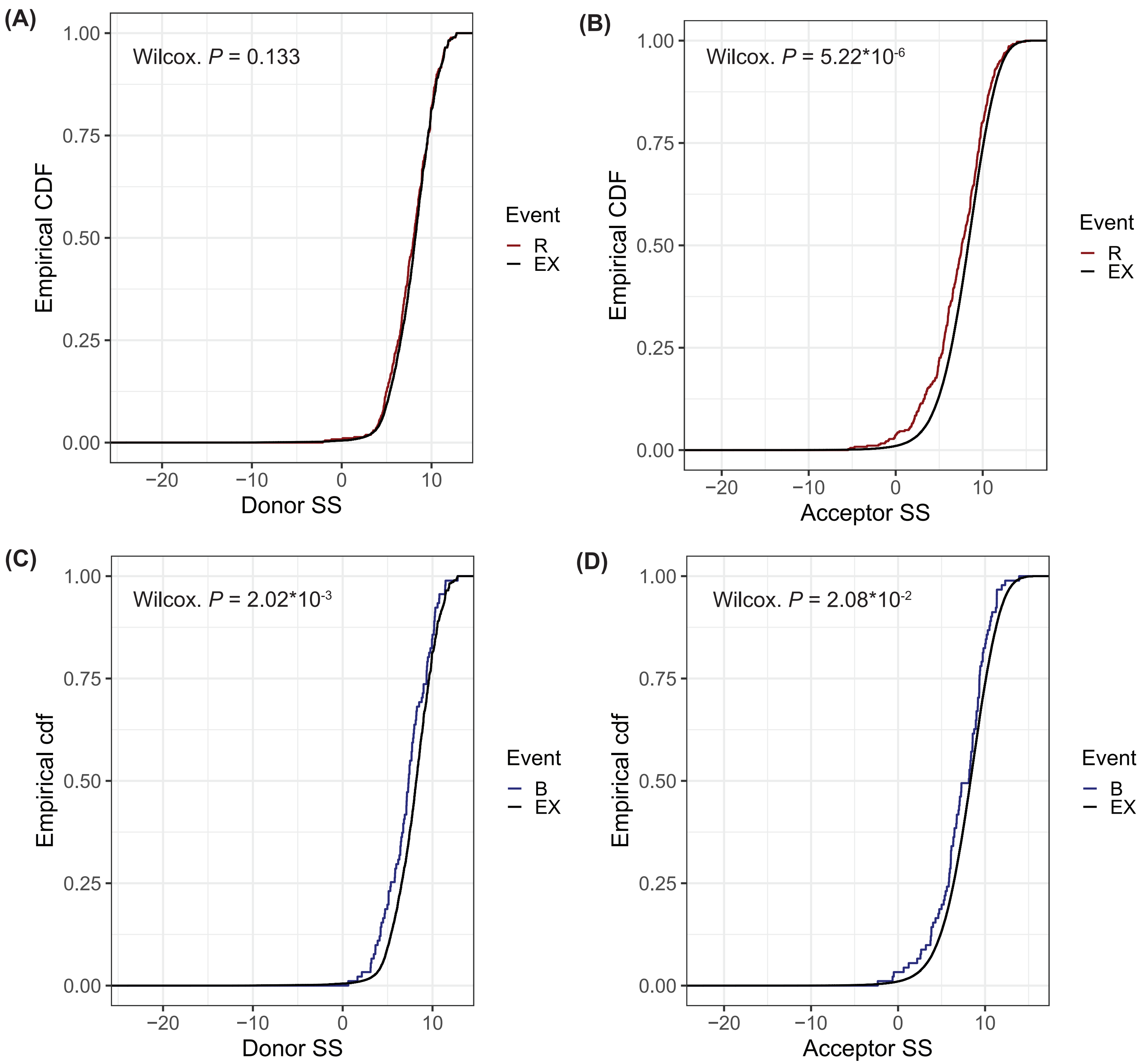

Supplementary Figure 4 – Splicing score analysis for AUD-associated IR events.

(**A** and **B**) Empirical cumulative distribution function (CDF) curves of splicing scores (SS) at the 5'-donor site and 3'-acceptor site of the positively AUD-associated IR events (***R***, red curve; n=368), compared to constitutively excised introns (black curve ***EX***; n=167,256).

(**C** and **D**) CDF curves of SS at the 5'-donor site and 3'-acceptor site of the negatively AUD-associated IR events (***B***, blue curve; n=91), compared to constitutively excised introns (black curve ***EX***; n=167,256).

The p-values were obtained using the Wilcoxon test.

Supplementary Figure 5

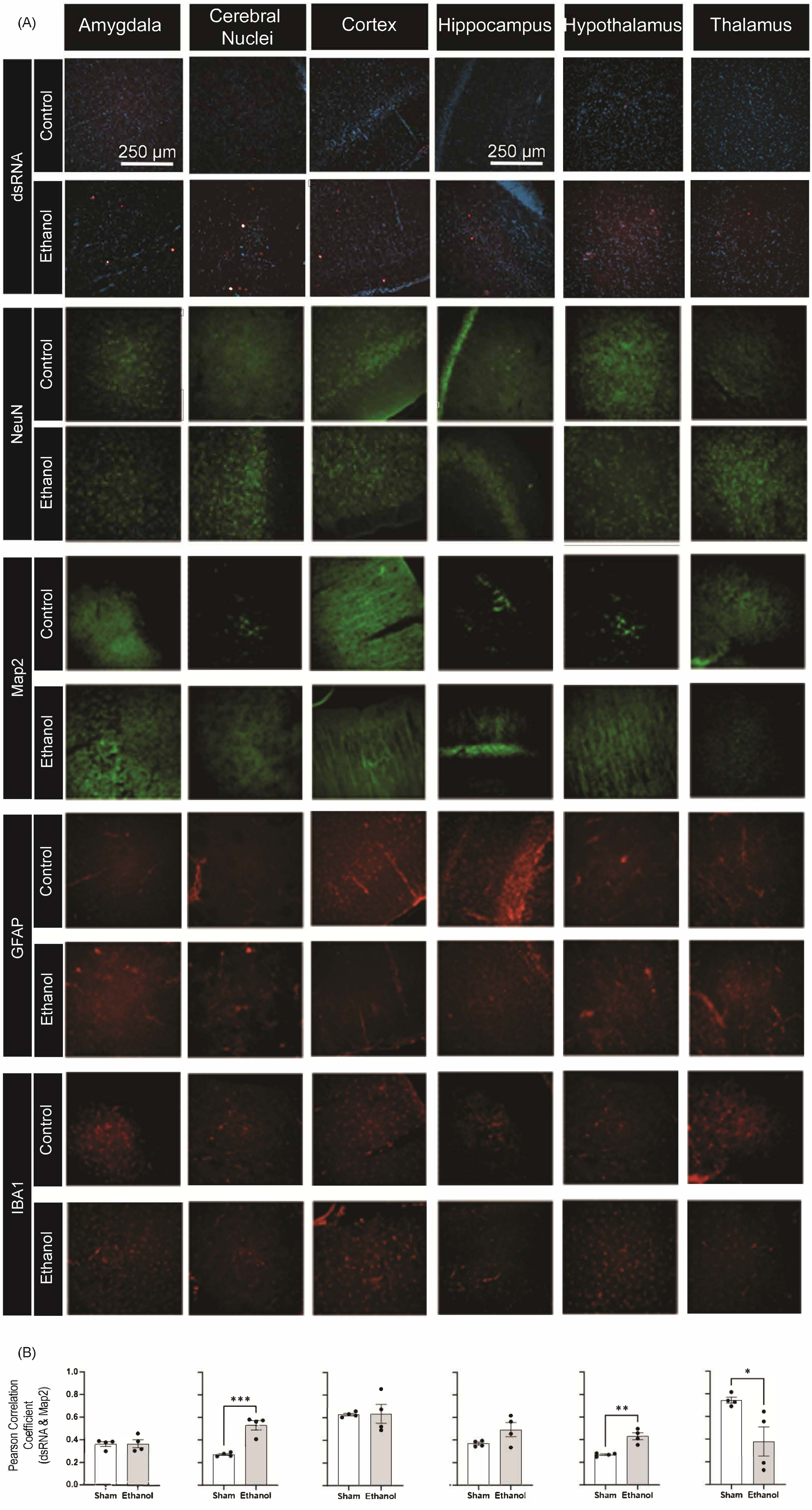

Supplementary Figure 5 – Histology images of six rat brain regions stained for dsRNA and cell-type specific markers.

(**A**) Immunofluorescence staining (histology) of dsRNA and cell type-specific markers (NeuN+ neuron nuclei, GFAP+ astrocytes, IBA1+ microglia, and Map2+ neuron dendrites) in six brain regions: amygdala, cerebral nuclei, cortex, hippocampus, hypothalamus, and thalamus from ethanol-drinking P rats and ethanol-naïve controls. In the dsRNA images, nuclei were stained with DAPI (blue), with the white signal depicting the overlap of dsRNA with DAPI-stained nuclei.

(**B**) Pearson correlation coefficient (data point) between dsRNA and the Map2 marker. *$p<0.1$, **$p<0.01$, and ***$p<0.001$.

Supplementary Figure 6

(**A**)

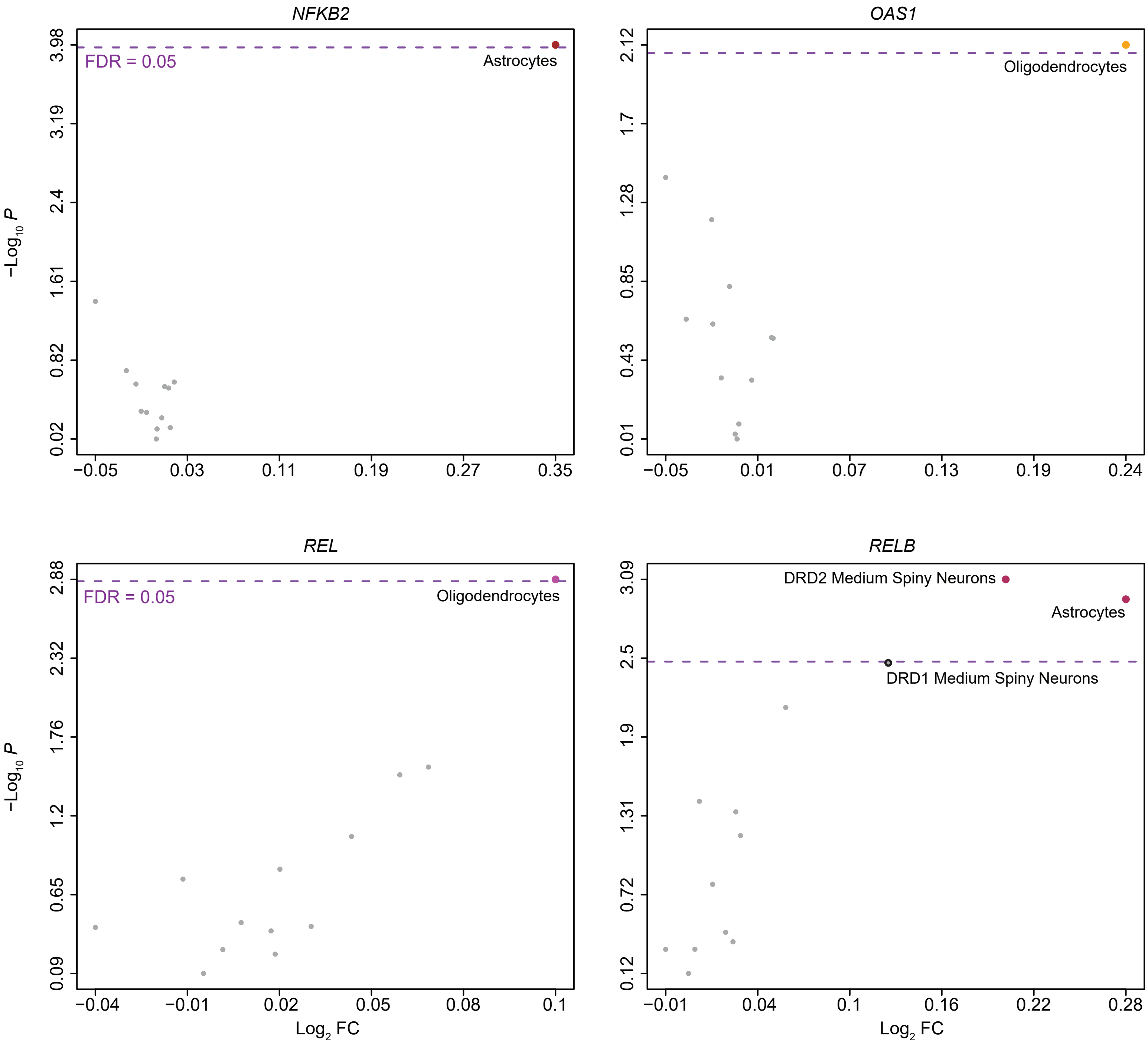

(**B**)

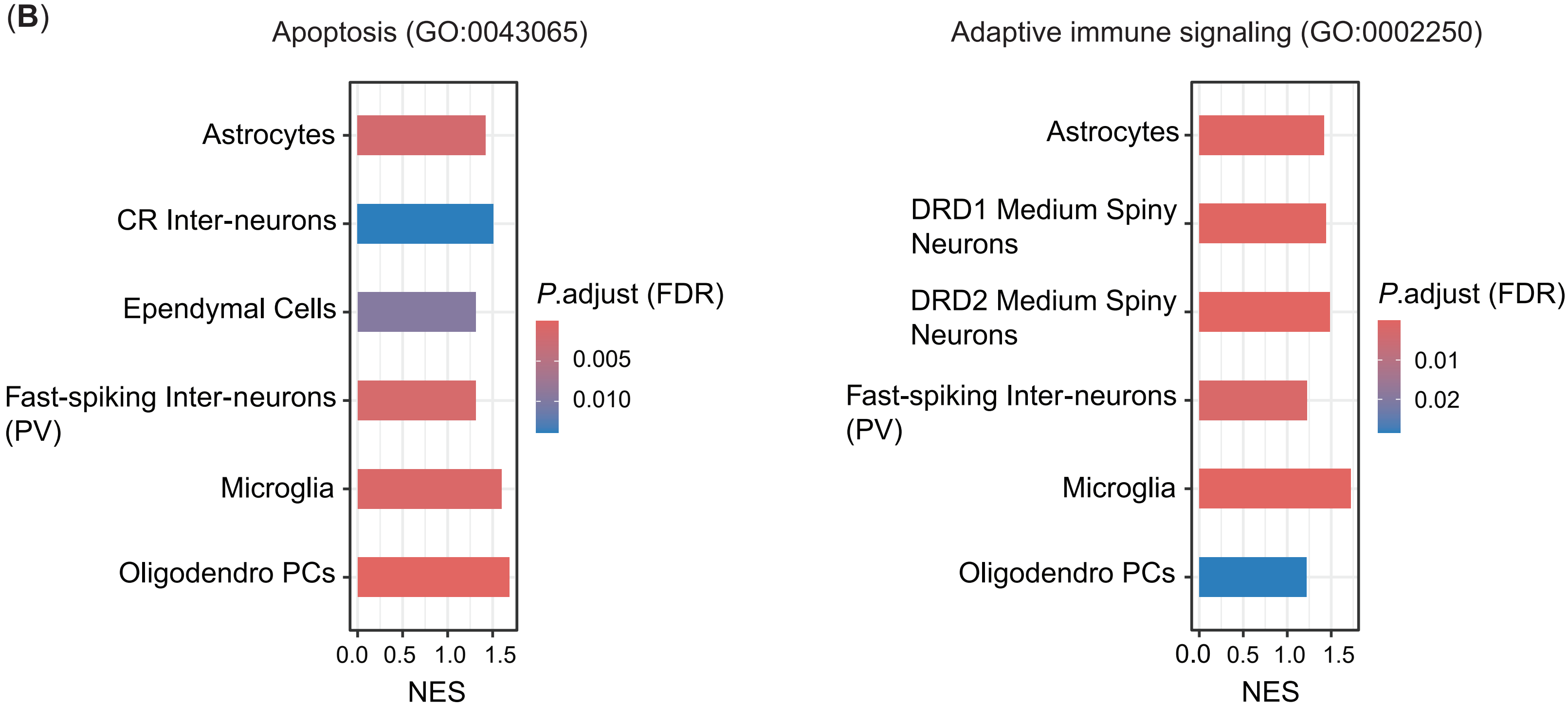
Apoptosis (GO:0043065)
Astrocytes
CR Inter-neurons
Ependymal Cells
Fast-spiking Inter-neurons (PV)
Microglia
Oligodendro PCs
0.0 0.5 1.0 1.5
NES
P.adjust (FDR)
0.005
0.010
Adaptive immune signaling (GO:0002250)
Astrocytes
DRD1 Medium Spiny Neurons
DRD2 Medium Spiny Neurons
Fast-spiking Inter-neurons (PV)
Microglia
Oligodendro PCs
0.0 0.5 1.0 1.5
NES
P.adjust (FDR)
0.01
0.02

Supplementary Figure 6 – Regulation of genes and pathways in specific neural cell types.

(**A**) Important genes related to dsRNA response and inflammation were differentially expressed in specific neuronal and glial cell types, including astrocytes, oligodendrocytes, DRD1 and 2 medium spiny neurons. Statistical tests were performed using DESeq2 (adjusting for all covariates), based on 142 individuals providing a maximum of 8,000 cells each. The *p*-values were further corrected using FDR. X-axis: $\log_2$ of the fold change (FC); Y-axis: $-\log_{10}$ of the *p*-value (*P*) calculated using DESeq2. Dashed purple lines indicate the threshold where FDR = 0.05.

(**B**) Additional dsRNA-related signaling pathways that showing strong enrichment (i.e., activation signal) in multiple neuronal and glial cell types. Apoptosis (left) and adaptive immune signaling (right) showed significant signal of activation in multiple neuronal and glial cell types from AUD donors (compared with non-AUD controls). NES: normalized enrichment score. The statistical significance (*p*-value) of enrichment was computed by GSEA and adjusted by FDR (indicated by different colors).

Supplementary Table 1. Demographic information of alcohol use disorder and control subjects

	**Alcohol use disorder (*N* = 66)**	**Control (*N* = 76)**
Male (%)	51 (77%)	61 (80%)
Caucasian (%)	66 (100%)	72 (95%)
Mean Age (SD) (yrs)	55.57 (11.73)	54.93 (11.92)
Mean PMI (SD) (yrs)*	33.39 (15.63)	26.62 (13.32)
Mean Brain pH (SD)	6.54 (0.23)	6.57 (0.31)
Mean RIN (SD)	6.84 (0.96)	7.0 (1.01)

*P value = 0.0095 (two-sided Wilcoxon test)

Supplementary Table 2. Key covariates for overall intron retention analysis.

	Coefficient [¶]	Standard-error	*P*-value
ALC [1]	0.3903	0.1196	1.097×10^{-3} **
Age [2]	0.005883	0.001755	8.021×10^{-4} ***
ALC × Age	-0.003438	0.002242	0.1252
Sex [3]	0.3636	0.3021	0.2303
ALC × Sex	-0.1642	0.08142	4.369×10^{-2} *
Coh [4]	0.7998	0.05586	1.697×10^{-46} *****
RIN [5]	-0.08546	0.07935	0.2815
PMI [6]	0.001012	0.001547	0.5129
Brain-R2 (NAC) [†]	0.1912	0.05495	5.016×10^{-4} ***
Brain-R3 (CE) [†]	0.3757	0.05516	9.599×10^{-12} *****
Brain-R4 (BLA) [†]	0.3619	0.05547	6.848×10^{-11} *****

Notes:

[1] ALC: alcohol use disorder status, 0/1.
[2] Age: continuous variable ranging from 22 to 82.
[3] Sex: male or female, 0/1.
[4] Coh: RNA sequencing cohort, 0/1.
[5] RIN: RNA integrity number.
[6] PMI: Post-mortem interval.
[†] Brain regions (4), as a categorical variable. Encoded as 3 Boolean variables, with Brain-R1 (SFC) being the default.
[¶] Regression coefficients were estimated on the log-scale.
* indicates the level of statistical significance

Supplementary Table 3

Worksheet 1: IR events positively associated with AUD

chrom	feature	intron_start	intron_end	strand	geneid	donor_start	donor_end	acceptor_start	acceptor_end	donorSEQ	acceptorSEQ	canonical	donor_junc.score	acceptor_junc.score	beta	se	p.value
chr10	intron	12215836	12217360	+	ENSG00000151465	12215833	12215842	12217348	12217361	AGGGTAGGAA	CTTCATTCTTTAGG	TRUE	6.4069	9.7901	1.365170739	0.432235242	0.00158639
chr22	intron	21000788	21001255	-	ENSG00000184436	21001258	21001249	21000800	21000787	CAGGTGGGCC	CTTTTCCCCGCAGT	TRUE	7.1154	9.6532	2.103403149	0.658715956	0.001407118
chr15	intron	101286294	101286907	-	ENSG00000131876	101286910	101286901	101286306	101286293	AAAGTAAGTA	GGTGTCGCTGTAGG	TRUE	9.8483	4.0113	2.5988	0.7605	0.00063273
chr9	intron	111533866	111534260	+	ENSG00000173258	111533863	111533872	111534248	111534261	TGGGTAAAGA	TCTCTATGAGCAGA	TRUE	4.79	4.8951	1.358991431	0.457359842	0.002964614
chr9	intron	6493933	6497197	+	ENSG00000147854	6493930	6493939	6497185	6497198	CAGGTACTAC	TGTTGTTTTTTAGG	TRUE	4.5169	10.1365	1.282846105	0.358463163	0.000345256
chr2	intron	190895726	190900563	+	ENSG00000115419	190895723	190895732	190900551	190900564	AAAGTAAAAG	TTACATTCTACAGA	TRUE	5.4612	8.028	0.786034719	0.300320557	0.008862346
chr1	intron	66667542	66670994	+	ENSG00000118473	66667539	66667548	66670982	66670995	CCGGTAAGAA	TTTCTAATTCTAGG	TRUE	8.9038	9.4376	0.699134921	0.248175228	0.004845914
chr1	intron	246877082	246877157	-	ENSG00000153207	246877160	246877151	246877094	246877081	CAGGTAATTA	TTGTTCTTTTTAGG	TRUE	9.6486	11.4818	1.025850958	0.357751263	0.004137317
chr5	intron	90694702	90696936	+	ENSG00000164199	90694699	90694708	90696924	90696937	AAGGTGAGCA	GATGTTTTTATAGG	TRUE	10.1894	7.5074	0.81211071	0.251275104	0.001229449
chr18	intron	34812114	34829399	+	ENSG00000134769	34812111	34812120	34829387	34829400	CAGGTAGGAG	TTGACTCCCTCAGG	TRUE	8.8577	7.8801	1.1886	0.3739	0.0014773
chr3	intron	14478569	14479084	+	ENSG00000131389	14478566	14478575	14479072	14479085	ATGGTGAGTA	CTGTCTCTTGCAGG	TRUE	9.8658	10.4912	1.299544579	0.557399587	0.019730087
chr10	intron	63215456	63215552	-	ENSG00000171988	63215555	63215546	63215468	63215455	CACGTATGTA	TGGTTATTTTTAGA	TRUE	6.6229	6.9464	0.698220425	0.286019055	0.014639862
chr17	intron	63799626	63800467	+	ENSG00000198231	63799623	63799632	63800455	63800468	CAAGTGAGTT	TGCTTTCCTGCAGG	TRUE	8.6509	10.5559	0.625832672	0.253015567	0.013379878
chr18	intron	45731205	45734273	+	ENSG00000141469	45731202	45731211	45734261	45734274	CAGGTAGGTG	TCTTGCCCCACAGG	TRUE	10.2428	9.7104	1.191166528	0.497932159	0.016746502
chr12	intron	49637825	49641907	+	ENSG00000110844	49637822	49637831	49641895	49641908	AAGGTGAGGG	GCTGTACCCACAGG	TRUE	10.4518	5.9843	1.714349409	0.561271604	0.002255093
chr2	intron	201478192	201478356	+	ENSG00000082146	201478189	201478198	201478344	201478357	CAGGTAAGTG	CTGTTTCTTTTAGA	TRUE	12.7631	9.834	0.73071893	0.250198664	0.003494086
chr6	intron	152269287	152278088	-	ENSG00000131018	152278091	152278082	152269299	152269286	CAGGTGAGGG	TCGTTAAAAGCAGG	TRUE	10.5441	2.6328	1.021665001	0.477998011	0.032566823
chr6	intron	53092548	53092733	+	ENSG00000112146	53092545	53092554	53092721	53092734	ATGGTAAGTT	ATTATTTTCATAGG	TRUE	10.3917	8.5749	0.923744181	0.326463445	0.004661372
chr2	intron	71418640	71422813	+	ENSG00000075292	71418637	71418646	71422801	71422814	AAGGTATGTT	TAAATACTTTTAGC	TRUE	9.8985	2.7866	0.795923184	0.282155552	0.004789411
chr8	intron	58599871	58599969	-	ENSG00000035681	58599972	58599963	58599883	58599870	GAGGTAAGCA	CCGAAACTTTCAGT	TRUE	10.2683	2.1177	0.66669771	0.264511785	0.011719358
chr3	intron	184307526	184307613	+	ENSG00000175166	184307523	184307532	184307601	184307614	GTGGTGAGTA	TTAACATTTCCAGG	TRUE	9.0369	7.576	1.500341883	0.738099442	0.042081862
chr1	intron	39284186	39284332	+	ENSG00000127603	39284183	39284192	39284320	39284333	AAGGTAAAGT	ATTAATTTTATAGG	TRUE	8.0455	7.2028	0.559129261	0.262529689	0.033190164
chr3	intron	194450277	194453705	-	ENSG00000133657	194453708	194453699	194450289	194450276	AAGGTAAGTA	TCTTTAATTTCAGA	TRUE	12.7057	9.7594	1.966	0.6651	0.003118
chr7	intron	78019977	78026067	-	ENSG00000187391	78026070	78026061	78019989	78019976	AGGGTAAGAA	TTCCCGTCCACAGA	TRUE	8.9272	8.044	1.886669674	0.638309223	0.003119301
chr11	intron	107630563	107630699	+	ENSG00000110675	107630560	107630569	107630687	107630700	TCGGTAAGCC	GCTGCTTTCACAGA	TRUE	6.6981	7.5845	0.630695797	0.234240894	0.007091655
chr7	intron	22267013	22291174	-	ENSG00000136237	22291177	22291168	22267025	22267012	GCGGTAAGAC	AATATTTTATTAGG	TRUE	7.3366	4.8537	1.486900689	0.514176763	0.003830314
chr17	intron	7220530	7220603	+	ENSG00000072778	7220527	7220536	7220591	7220604	GCGGTAGGTA	TCTCTCTGCCCAGG	TRUE	6.8474	9.2989	0.620795037	0.254014533	0.014528039
chr3	intron	121115060	121121640	+	ENSG00000145087	121115057	121115066	121121628	121121641	ACTGTAAGTT	ATTGATTTAACAGA	TRUE	6.7082	5.1971	1.152043848	0.399424078	0.003923317
chr18	intron	45730472	45731014	+	ENSG00000141469	45730469	45730478	45731002	45731015	AAGGTATTTA	TCATCCCTTCCAGA	TRUE	7.131	9.3092	1.792452629	0.672680564	0.007707041
chr15	intron	85100199	85109052	+	ENSG00000073417	85100196	85100205	85109040	85109053	CAGGTACGGT	TTTGTTTCTACAGA	TRUE	8.0043	11.5965	1.23704185	0.455416938	0.006601921
chr4	intron	113531300	113537340	-	ENSG00000145349	113537343	113537334	113531312	113531299	TTGGTGAGTA	CTCTTTTCCCTAGG	TRUE	8.6218	10.9004	1.004336809	0.359483619	0.005208754
chr2	intron	53887990	53888720	-	ENSG00000068878	53888723	53888714	53888002	53887989	ATGGTAAATT	GCTTTAATTTTAGT	TRUE	7.4246	5.4766	1.434412208	0.671866431	0.032763761
chr11	intron	11920495	11922723	+	ENSG00000170242	11920492	11920501	11922711	11922724	GAGGTAAATA	ATGTCTTATTAAGA	TRUE	8.9097	3.5133	1.621126495	0.463981011	0.00047593
chr12	intron	89591296	89598589	-	ENSG00000070961	89598592	89598583	89591308	89591295	GGTGTGAGTG	CTGATATTTACAGA	TRUE	5.4342	6.6493	0.988609573	0.424323605	0.019814177
chrX	intron	71538073	71544566	+	ENSG00000147162	71538070	71538079	71544554	71544567	CCTGTGAGTA	TCTGGGTTTCTAGG	TRUE	6.2746	7.0492	0.631382154	0.274498348	0.0214408
chr10	intron	12149727	12155310	+	ENSG00000065665	12149724	12149733	12155298	12155311	AACGTGAGCA	GCTTTTTTCAAAGG	TRUE	6.4396	8.0325	0.7192	0.2581	0.0053302
chr1	intron	31367495	31369384	-	ENSG00000121769	31369387	31369378	31367507	31367494	AAGGTAAGTC	CCTCTTTCCACAGT	TRUE	12.0597	9.1336	0.934920361	0.310793102	0.002628209
chr2	intron	161957241	161958486	+	ENSG00000144290	161957238	161957247	161958474	161958487	AAGGTAACAA	TGTTCTTTTACAGT	TRUE	7.7873	10.3367	1.160313044	0.404292994	0.00410497
chr9	intron	123158124	123158331	-	ENSG00000165209	123158334	123158325	123158136	123158123	CAGGTAGAAG	CTTTGTTCTTTAGC	TRUE	5.8906	9.2511	1.187131025	0.532036472	0.025661507
chr11	intron	118495894	118496260	+	ENSG00000118058	118495891	118495900	118496248	118496261	TGAGTAAGCC	TTTGGATTTCAAGG	TRUE	3.9564	5.8087	0.676962496	0.269219312	0.011918807
chr5	intron	14489070	14492566	+	ENSG00000038382	14489067	14489076	14492554	14492567	TGCGTGAGTG	TGTCCCTCTGCAGA	TRUE	4.3758	9.1719	1.242352366	0.450677942	0.005840038
chrX	intron	21516632	21526866	+	ENSG00000149970	21516629	21516638	21526854	21526867	CAGGTAATGA	TCATTTTAACCAGC	TRUE	8.3374	5.2391	0.836131889	0.334453866	0.012419622
chr5	intron	14397155	14398879	+	ENSG00000038382	14397152	14397161	14398867	14398880	AAGGTAAAGG	TTTCATGTTGTAGG	TRUE	8.3925	8.5065	1.490501541	0.54311567	0.006063016
chr17	intron	64514359	64516722	+	ENSG00000258890	64514356	64514365	64516710	64516723	AAAGTAAGTT	CTGTTCTGAACAGG	TRUE	9.4664	5.9974	1.3602	0.4982	0.0063283
chr1	intron	237727200	237730259	+	ENSG00000198626	237727197	237727206	237730247	237730260	AAGGTCGGAA	GCTTACCTTTCAGG	TRUE	4.6573	9.2007	1.667199717	0.841526896	0.047573295
chr18	intron	686009	688573	-	ENSG00000132199	688576	688567	686021	686008	CAGGTGAGTG	ATGCATTTTCTAGG	TRUE	11.8553	6.5911	1.060228913	0.326746745	0.001175318
chr10	intron	91484656	91485179	+	ENSG00000165338	91484653	91484662	91485167	91485180	TTAGTAGGTT	TTTATCTTTACAGA	TRUE	3.77	11.4008	1.29587354	0.585308983	0.026828844
chr2	intron	44321901	44322730	-	ENSG00000138078	44322733	44322724	44321913	44321900	AAGGTAGGAG	TTAACTCTTCCAGG	TRUE	8.7654	9.1004	1.357303131	0.503161451	0.006985181
chr14	intron	20357150	20357395	+	ENSG00000129484	20357147	20357156	20357383	20357396	GAGGTGAGAC	TTTTGCTTTGCAGG	TRUE	8.9379	12.3966	0.699919326	0.24714238	0.004625076
chr9	intron	89332987	89334521	+	ENSG00000187742	89332984	89332993	89334509	89334522	GAGGTAAAAG	TATCTTTGAGCAGA	TRUE	7.4897	5.4911	0.956376471	0.300861556	0.001478899
chr1	intron	54630887	54634687	+	ENSG00000162390	54630884	54630893	54634675	54634688	GTGGTGAGTA	TGCGTTTCTCCAGG	TRUE	9.0369	9.3023	1.068373494	0.468696742	0.022639986
chr2	intron	113755307	113755453	-	ENSG00000115084	113755456	113755447	113755319	113755306	AAGGTAGATT	TTCTAAAAAAACAGA	TRUE	6.8364	1.9858	0.801828916	0.30655129	0.008905947
chr1	intron	66679753	66681868	+	ENSG00000118473	66679750	66679759	66681856	66681869	CAGGTACGCT	ACTTTAACCACAGG	TRUE	7.707	6.5997	1.056603169	0.47895672	0.027380397
chr18	intron	34818331	34820790	+	ENSG00000134769	34818328	34818337	34820778	34820791	TGGGTAAGGC	TTCTTCTTTCCAGA	TRUE	7.1111	12.9841	0.541204857	0.243257293	0.026093002
chr7	intron	151568843	151570170	-	ENSG00000106617	151570173	151570164	151568855	151568842	AAGGTAAACT	ACTTCTCCCACAGC	TRUE	7.7482	7.7249	0.695957786	0.349169742	0.046241853
chr17	intron	16026703	16027373	+	ENSG00000011295	16026700	16026709	16027361	16027374	GAGGTAGGTA	TGGGTTATTTTAGA	TRUE	9.3565	4.7884	0.6994	0.2623	0.0076671
chr18	intron	58341170	58341677	+	ENSG00000049759	58341167	58341176	58341665	58341678	CAGGTACGAA	GCCTCCAAAATAGC	TRUE	8.3123	0.1105	1.478699059	0.555846211	0.007807884
chr8	intron	100264196	100264670	-	ENSG00000034677	100264673	100264664	100264208	100264195	TAGGTAAGAA	AATTTCTCTTTAGG	TRUE	10.0766	8.7158	0.596505916	0.235644446	0.01136143
chr5	intron	90802883	90805283	+	ENSG00000164199	90802880	90802889	90805271	90805284	CAGGTAATTG	GATTTTCCCTCAGG	TRUE	9.6137	9.0646	1.693648244	0.806002294	0.035615116
chr15	intron	44566010	44566216	-	ENSG00000104133	44566219	44566210	44566022	44566009	AAGGTAACCC	CCTTGTTCCTCAGG	TRUE	6.9179	10.1792	0.964388553	0.365451848	0.008317708
chr2	intron	152637675	152639957	+	ENSG00000157827	152637672	152637681	152639945	152639958	AAGGTATATG	TTGATTTACCCAGC	TRUE	7.2784	5.5342	0.823537329	0.366852269	0.024776186
chr9	intron	92253454	92256679	-	ENSG00000196305	92256682	92256673	92253466	92253453	CAGGTGAGTC	GTCTTACTTTTAGC	TRUE	11.2442	7.0446	1.845071972	0.84261002	0.028545242
chr4	intron	78930326	78935120	-	ENSG00000163291	78935123	78935114	78930338	78930325	CAGGTAAGTC	TTATTTTTCACAGG	TRUE	12.152	11.7259	1.126741268	0.48323263	0.019717814
chr18	intron	34827677	34829040	+	ENSG00000134769	34827674	34827683	34829028	34829041	GAGGTAAGTT	TATATCATTTCAGC	TRUE	11.4949	6.516	0.573585768	0.286544332	0.045313259
chr7	intron	137623555	137645471	-	ENSG00000157680	137645474	137645465	137623567	137623554	AAGGTAAGAG	TCTAATATCCCAGG	TRUE	11.2857	6.9245	1.858335867	0.708597939	0.008727361
chr11	intron	124630486	124630744	+	ENSG00000154144	124630483	124630492	124630732	124630745	TATGTAAGTT	CTCTCCTGTTTAGG	TRUE	7.9733	8.2717	1.504408102	0.645190662	0.019715089
chr10	intron	20897030	20897124	-	ENSG00000078114	20897127	20897118	20897042	20897029	CAGGTGAGTG	TTTTTTCAAATAGG	TRUE	11.8553	8.5926	2.635097879	1.059082351	0.012842929
chr1	intron	200850235	200852540	+	ENSG00000118200	200850232	200850241	200852528	200852541	AAGGTGTAGT	AATTCGTTCTTAGG	TRUE	4.7124	6.1026	0.863035615	0.39403463	0.028505878
chr10	intron	68394137	68394327	-	ENSG00000204130	68394330	68394321	68394149	68394136	CAAGTAAGTG	TTATTTACTTCAGG	TRUE	9.9057	10.2991	0.502243723	0.238333846	0.035090562
chr10	intron	20817693	20819423	-	ENSG00000078114	20819426	20819417	20817705	20817692	GCGGCAAGTC	TTATCCTTCTTAGG	FALSE	2.6702	9.6296	1.04537027	0.404051048	0.009675288
chr7	intron	43445571	43450827	+	ENSG00000002746	43445568	43445577	43450815	43450828	AAGGTTAGAC	ATCTTGTCCGTAGG	TRUE	6.5374	6.9774	1.275642377	0.493324767	0.009715146
chr14	intron	50977354	50979386	-	ENSG00000100505	50979389	50979380	50977366	50977353	CAGGTAAGCA	TCTCATTCCCCAGG	TRUE	11.1895	9.8743	1.0726	0.4152	0.0097902
chr11	intron	57737669	57737912	+	ENSG00000213593	57737666	57737675	57737900	57737913	CCAGTAAGTT	CTCTGTGTTTCAGT	TRUE	7.0496	7.3039	0.674806945	0.261537569	0.009875655
chr8	intron	100261756	100264033	-	ENSG00000034677	100264036	100264027	100261768	100261755	TAGGTAAGTC	TATTTGCATTTAGA	TRUE	10.8157	6.1046	1.690299302	0.655310898	0.009897632
chr2	intron	27368140	27368371	-	ENSG00000115211	27368374	27368365	27368152	27368139	GAGGTAGGAT	GCCATCCCATCAGC	TRUE	7.5895	3.1031	0.699569735	0.319844518	0.028726603
chr12	intron	56178535	56178809	-	ENSG00000139613	56178812	56178803	56178547	56178534	AAGGTGGAGC	TCCATACTCGTAGG	TRUE	4.3533	5.9859	0.741358123	0.287541435	0.009929771
chr1	intron	112649470	112653597	+	ENSG00000116489	112649467	112649476	112653585	112653598	ACAGTAAGTA	TAAAAAAAAAACAGT	TRUE	7.3392	-5.4692	1.401070837	0.544826736	0.010123278
chr9	intron	87638082	87639353	+	ENSG00000196730	87638079	87638088	87639341	87639354	AAGGTACGTT	TCTTTTTTTCAAGC	TRUE	9.2232	9.5737	1.077440928	0.419352643	0.010190547
chr4	intron	159343191	159343280	+	ENSG00000109756	159343188	159343197	159343268	159343281	AAGGTAAACA	CTGTCAATTTCAGG	TRUE	8.1301	7.4888	0.705372114	0.340975423	0.038575278
chr6	intron	43019166	43019287	+	ENSG00000124702	43019163	43019172	43019275	43019288	TTAGTAAGTA	TTCTCCCTTAAAGG	TRUE	6.6722	9.0119	1.289921767	0.624451799	0.038858073
chr18	intron	50275982	50276377	-	ENSG00000141644	50276380	50276371	50275994	50275981	AAGGTAAGCA	CCATTCCTGGCAGC	TRUE	11.0972	4.9437	0.823530083	0.306460272	0.007204673
chr10	intron	78021683	78021859	-	ENSG00000148606	78021862	78021853	78021695	78021682	AGGGTAGGTT	TTTGGCTATTTAGG	TRUE	7.4101	7.0969	0.695775596	0.305038491	0.022551784
chr7	intron	134935766	134941091	+	ENSG00000122786	134935763	134935772	134941079	134941092	GAGGTAAATA	TACTGTTGGTTAGA	TRUE	8.9097	2.2017	1.816994725	0.712785222	0.010798659
chr7	intron	106269313	106272529	-	ENSG00000105835	106272532	106272523	106269325	106269312	GAGGTAAATT	TTTGTTACTACAGA	TRUE	8.5278	9.6132	1.37749733	0.49080317	0.005006447
chr11	intron	6396216	6400988	-	ENSG00000166313	6400991	6400982	6396228	6396215	TTGGTATGTG	CCATATGTTCTAGG	TRUE	7.0694	5.4724	0.956280741	0.457600424	0.036638249
chr1	intron	8015385	8015494	-	ENSG00000116285	8015497	8015488	8015397	8015384	AAAGTAAGTA	CCTCTCCTTTCAGC	TRUE	9.8483	10.1439	0.533529741	0.249052999	0.032174677
chr16	intron	82149404	82151423	-	ENSG00000135698	82151426	82151417	82149416	82149403	GAGGTATTAA	GTGTATTTTTCAGA	TRUE	4.917	7.9478	1.254541608	0.521735359	0.016192149
chr3	intron	394873	398226	+	ENSG00000134121	394870	394879	398214	398227	GGAGTAAGTA	GTACATTTCTTAGG	TRUE	6.626	5.6675	1.015228022	0.401585515	0.011469821
chr1	intron	160091939	160092281	-	ENSG00000162729	160092284	160092275	160091951	160091938	ATGGTGAGTG	CTCTCTCCCCCAGC	TRUE	9.8309	9.09	1.549497172	0.613031138	0.011484529
chr8	intron	61676320	61680967	-	ENSG00000198363	61680970	61680961	61676332	61676319	TAGGCAAGTA	TATTCTTATGTAGG	FALSE	5.4102	8.3179	0.732135477	0.290175894	0.011633366
chr10	intron	93758360	93777378	+	ENSG00000108231	93758357	93758366	93777366	93777379	ATTGTAAGGC	TTTTTCTGGGCAGG	TRUE	5.7099	8.5738	1.6171	0.6424	0.011821
chr2	intron	227536994	227550058	+	ENSG00000173744	227536991	227537000	227550046	227550059	CAGGTAAGAA	GGCTTTCCATAAGG	TRUE	11.4129	3.1232	0.949036609	0.453341929	0.036311374
chr4	intron	73146874	73147240	-	ENSG00000132466	73147243	73147234	73146886	73146873	CAGGTAGGCA	TTGACTACTACAGG	TRUE	8.6692	7.0311	0.721384942	0.268975223	0.007318883
chr19	intron	40532772	40534079	+	ENSG00000160460	40532769	40532778	40534067	40534080	CGGGTGAGGA	CCACTTGGGGCAGG	TRUE	8.1856	1.5904	1.264141697	0.503154914	0.011990276
chr4	intron	119023582	119037111	+	ENSG00000172403	119023579	119023588	119037099	119037112	AAGGTACAAC	TGCTCTGTGACAGG	TRUE	4.6069	5.9469	0.5913	0.2355	0.012037
chr2	intron	29167541	29174372	+	ENSG00000115295	29167538	29167547	29174360	29174373	CTGGTAAGAC	CTTTCATATTTAGG	TRUE	8.8348	8.2769	0.824197056	0.27257306	0.002496497
chr11	intron	62677635	62677780	-	ENSG00000162191	62677783	62677774	62677647	62677634	AAGGTAGGTG	CTTTGCCCTTTAGT	TRUE	10.1505	7.9021	0.963793463	0.451654004	0.032849339
chr2	intron	135753568	135754158	+	ENSG00000144224	135753565	135753574	135754146	135754159	TCTGTATCCT	TTAAACTGTACAGA	TRUE	-2.1029	3.6148	0.659354771	0.263210851	0.012243615
chr10	intron	95410778	95414493	-	ENSG00000095637	95414496	95414487	95410790	95410777	ACCGTAAGCT	TTGTTCGTTTCAGG	TRUE	4.4564	10.3248	0.757746087	0.302779016	0.012327177

chr16	intron	11897882	11897993	-	ENSG00000103342	11897996	11897987	11897894	11897881	AAGGTAATGA	ATCTCTTGTCTAGG	TRUE	8.2451	7.4036	0.923590246	0.426371922	0.030298883
chr9	intron	97662145	97662953	+	ENSG00000136937	97662142	97662151	97662941	97662954	AAAGTATGTA	TCATTATCAAAAGG	TRUE	7.423	3.2066	0.66077128	0.311926622	0.034145025
chr5	intron	95919546	95943001	-	ENSG00000118985	95943004	95942995	95919558	95919545	GGGGTGAGTA	CCCCTCATTTTAGC	TRUE	8.5756	6.4855	0.902393219	0.410562972	0.027953305
chr10	intron	63200678	63206594	-	ENSG00000171988	63206597	63206588	63200690	63200677	ATAGTAAGTC	ACTGATTTTACAGC	TRUE	7.2702	6.0018	1.843017162	0.818500868	0.024341333
chr2	intron	238183741	238184019	-	ENSG00000132323	238184022	238184013	238183753	238183740	CCAGTAAGTA	TGTTTCCCCCCAGG	TRUE	7.4315	10.6713	0.715183569	0.276366324	0.009658821
chr4	intron	159343405	159344035	+	ENSG00000109756	159343402	159343411	159344023	159344036	TCGGTGAGTA	CTCTCACTTACAGG	TRUE	8.0448	9.9809	0.641189485	0.256898357	0.012564232
chr7	intron	137619941	137623482	-	ENSG00000157680	137623485	137623476	137619953	137619940	GCGGTAAGAT	TTTTTCTCTTCAGT	TRUE	7.6007	12.4217	0.522286859	0.236740371	0.027372881
chr4	intron	168925079	168925232	+	ENSG00000129116	168925076	168925085	168925220	168925233	ACAGTGAGTG	TTTCTATTTGTAGT	TRUE	6.3965	9.0396	0.789013774	0.264908357	0.002897193
chr14	intron	96322255	96322539	-	ENSG00000066739	96322542	96322533	96322267	96322254	CAGGTAAAAG	TATTATGTTTTAGA	TRUE	8.4109	6.7459	0.619794406	0.291116163	0.03325196
chr16	intron	21597301	21612644	+	ENSG00000197006	21597298	21597307	21612632	21612645	AAGGTCAGCT	TTTTTTTTTTTAGT	TRUE	6.5723	12.484	0.667	0.2693	0.01324
chr2	intron	206562294	206565019	+	ENSG00000114948	206562291	206562300	206565007	206565020	CAAGTATGTA	TTTATTGGACCAGA	TRUE	7.5153	4.4694	0.899694887	0.437171979	0.039591241
chr13	intron	110634777	110635467	+	ENSG00000213995	110634774	110634783	110635455	110635468	GTGGTGAGTC	TGTGTTGTCATAGC	TRUE	8.3909	4.9385	0.737455415	0.298359036	0.013447056
chr2	intron	97167606	97167694	+	ENSG00000135976	97167603	97167612	97167682	97167695	ACGGTAAACA	AAAACCCATTCAGC	TRUE	5.621	-0.0846	1.33428075	0.534483423	0.012546346
chr21	intron	46442574	46443809	+	ENSG00000160299	46442571	46442580	46443797	46443810	CAGGTGGGAC	AATTCTGGGGAAGG	TRUE	7.3388	-1.7484	1.036788639	0.420376273	0.013650367
chr1	intron	227035608	227040130	-	ENSG00000143776	227040133	227040124	227035620	227035607	AAGGTAAGAA	TTCCCTCTAAAAGT	TRUE	11.3206	4.1805	0.808685549	0.347145604	0.019831089
chr2	intron	218265683	218265830	-	ENSG00000127837	218265833	218265824	218265695	218265682	AAGGTGAGTG	CTCTGGCTTTCAGG	TRUE	11.763	8.8406	0.745415909	0.303204622	0.0139535
chr15	intron	63675094	63677844	-	ENSG00000103657	63677847	63677838	63675106	63675093	CAGGTATGAT	TTTGGTCGCCTAGT	TRUE	8.6057	3.8372	0.931659102	0.379031049	0.013971237
chr10	intron	95357106	95357630	-	ENSG00000095637	95357633	95357624	95357118	95357105	AAGGTAATTA	GCATGCTTACCAGT	TRUE	9.5563	2.7686	1.246213906	0.507618597	0.014087668
chr11	intron	47478953	47482694	-	ENSG00000149187	47482697	47482688	47478965	47478952	GCAGTGAGTC	GTGTAATGTCTAGC	TRUE	4.9565	1.0346	1.931635157	0.787833174	0.014213072
chr10	intron	119823903	119825951	+	ENSG00000198825	119823900	119823909	119825939	119825952	GAGGTGAGTA	TTCTTATTTATAGC	TRUE	10.969	9.3657	1.805092283	0.786521808	0.021731322
chr10	intron	24635104	24666984	-	ENSG00000107863	24666987	24666978	24635116	24635103	GAGGTATGCT	TTCTCTGTAACAGG	TRUE	7.4611	8.6117	1.45138433	0.619700642	0.019176948
chr2	intron	71427415	71428546	+	ENSG00000075292	71427412	71427421	71428534	71428547	CAGGTAGATA	TTTCTTTTTAAAGC	TRUE	7.3106	9.3681	0.87173921	0.356538651	0.014484966
chr15	intron	101285802	101286213	-	ENSG00000131876	101286216	101286207	101285814	101285801	AACGTAAGAC	TCCCCCTCTTAAGT	TRUE	6.9248	5.4461	0.589528975	0.267715064	0.027659925
chr17	intron	31357091	31357268	+	ENSG00000196712	31357088	31357097	31357256	31357269	CTAGTAAGGA	TGCATCTTGGCAGG	TRUE	6.6973	5.6813	2.119569407	0.849078666	0.012549044
chr2	intron	61222664	61223059	-	ENSG00000115464	61223062	61223053	61222676	61222663	CATGTAAGTG	CTTTCTGTTTTAGA	TRUE	9.6566	0.7609	0.92906255	0.473604419	0.049799202
chr18	intron	34838172	34838744	+	ENSG00000134769	34838169	34838178	34838732	34838745	AGGGTAAGTT	TGCCCTGTTTCAGG	TRUE	9.9304	8.2972	0.690909959	0.319750666	0.030712552
chr2	intron	101286178	101288972	-	ENSG00000163162	101288975	101288966	101286190	101286177	CAAGTATGTT	TTAATCTTTTTAGG	TRUE	7.1334	8.7881	1.082266127	0.444716419	0.014949111
chr18	intron	34851929	34858284	+	ENSG00000134769	34851926	34851935	34858272	34858285	CAGGTGAGAC	CCTCTCTCCCAAGA	TRUE	9.8591	6.5989	0.667450755	0.338174136	0.048417055
chr11	intron	83633326	83786689	-	ENSG00000150672	83786692	83786683	83633338	83633325	AAGGTCAGTC	TTTTCATTCCCAGA	TRUE	7.9167	10.9569	0.9306	0.3829	0.015071
chr10	intron	95322154	95322745	-	ENSG00000095637	95322748	95322739	95322166	95322153	GTGGTAAGAT	GGCCACCTCACAGG	TRUE	8.1777	4.7196	0.611537635	0.251840676	0.015170965
chr1	intron	182818690	182829602	+	ENSG00000135838	182818687	182818696	182829590	182829603	GAGGTAAGTC	TTTTTCTATACAGA	TRUE	11.2308	11.369	0.6212	0.2558	0.015176
chr12	intron	98782138	98798933	-	ENSG00000185046	98798936	98798927	98782150	98782137	CGGGTAAGTC	GAAAATCCACCAGG	TRUE	9.7586	0.0064	0.9647	0.3974	0.015193
chr13	intron	35805780	35808223	-	ENSG00000133083	35808226	35808217	35805792	35805779	AAGGTAGGTG	TTCCCCTTCACAGG	TRUE	10.1505	11.056	1.015303632	0.419424404	0.015490353
chr1	intron	145995032	145995143	-	ENSG00000265972	145995146	145995137	145995044	145995031	ATGGTGAGAT	CCCTTCTATATAGG	TRUE	8.0988	8.2334	0.916030169	0.347867371	0.008456605
chr12	intron	2803241	2805167	+	ENSG00000004478	2803238	2803247	2805155	2805168	CAGGTGGAGA	ACCCTATTTACAGA	TRUE	5.0916	7.2605	2.119040825	0.779254943	0.006541698
chr5	intron	10397345	10400783	+	ENSG00000145495	10397342	10397351	10400771	10400784	TTGGTAAGTT	TCCCCCTTTTTAGC	TRUE	9.1477	8.5852	0.718247559	0.253671066	0.004634284
chr19	intron	17627598	17627862	-	ENSG00000130477	17627865	17627856	17627610	17627597	GAGGTGAGGC	TCTCCCTCCTTAGC	TRUE	9.0118	8.189	1.415156999	0.585774879	0.015697588
chr3	intron	50114100	50114179	+	ENSG00000003756	50114097	50114106	50114167	50114180	AAGGTAATAG	CTCTGTTTCCCAGG	TRUE	8.1363	10.5306	1.969311375	0.815476924	0.015738674
chrX	intron	136207191	136207791	+	ENSG00000022267	136207188	136207197	136207779	136207792	CAGGTACTGC	CTTCCCTCTGCAGG	TRUE	4.5908	11.3528	0.542404115	0.25038844	0.03029204
chr10	intron	95422074	95422189	-	ENSG00000095637	95422192	95422183	95422086	95422073	CAGGTATGGC	CTCTATTTCTCAGA	TRUE	8.4155	9.5712	1.436602956	0.595294017	0.015810266
chr2	intron	206560119	206561127	+	ENSG00000114948	206560116	206560125	206561115	206561128	CTCGTACGTA	GCTTTTTCTTAAGG	TRUE	4.0155	8.589	0.838171434	0.347723935	0.01593282
chr20	intron	25289931	25290480	+	ENSG00000100994	25289928	25289937	25290468	25290481	CGGGTACTTC	CCTCTCCTTCCAGG	TRUE	3.5086	11.7256	1.60267421	0.760151501	0.034999726
chr1	intron	88961288	88961380	-	ENSG00000137944	88961383	88961374	88961300	88961287	AAGGTAAGCA	TTATTATGTGTAGG	TRUE	11.0972	6.3546	0.557660212	0.232504431	0.016462699
chr2	intron	53895077	53895582	-	ENSG00000068878	53895585	53895576	53895089	53895076	AAAGTAAGTA	TCTTACTATATAGG	TRUE	9.8483	7.9296	1.650536711	0.807536321	0.040961809
chr1	intron	110016731	110017494	+	ENSG00000168710	110016728	110016737	110017482	110017495	GAGGTAAATA	TTTTGTTCTGCAGG	TRUE	8.9097	12.3305	0.710450069	0.296480525	0.016562476
chr3	intron	33604210	33606590	-	ENSG00000163539	33606593	33606584	33604222	33604209	CAAGTAAGGT	TTATACTTTGCAGT	TRUE	8.2475	7.6432	0.779691671	0.397764097	0.049974038
chr7	intron	108178113	108180222	-	ENSG00000091129	108180225	108180216	108178125	108178112	GAGGTATGGA	TTCTCACTGTAAGT	TRUE	8.1403	2.8707	0.9389	0.3952	0.017526
chr9	intron	97658744	97660945	+	ENSG00000136937	97658741	97658750	97660933	97660946	GCAGTAAGTA	ATTCTGTGTTTAGA	TRUE	6.5103	5.4605	1.582668588	0.668446704	0.01789996
chr11	intron	10604546	10606741	-	ENSG00000072952	10606744	10606735	10604558	10604545	GAGGTAAGTG	TTGTTCCCAACAGA	TRUE	11.8419	7.822	0.680480329	0.287407508	0.017901472
chr3	intron	98518996	98521652	-	ENSG00000080822	98521655	98521646	98519008	98518995	TGGGTATGTG	GTTCTTGTTCCAGA	TRUE	6.6081	9.4054	0.7236	0.3061	0.018071
chr3	intron	15257114	15258830	-	ENSG00000131370	15258833	15258824	15257126	15257113	CTGGTAAGTC	TCTCTCAACTTAGT	TRUE	10.2199	4.7531	0.869941551	0.368401121	0.018206195
chr12	intron	98799006	98800996	-	ENSG00000185046	98800999	98800990	98799018	98799005	TTTGTAAGTT	TTTTGTATTTTAGT	TRUE	6.0412	8.438	2.166750839	0.920226908	0.018543491
chr2	intron	99402347	99402643	-	ENSG00000135945	99402646	99402637	99402359	99402346	CAGGTTGGCT	TCCCCCTTCCTAGA	TRUE	4.1501	8.4571	0.67620158	0.317413407	0.033142747
chr11	intron	767374	770313	-	ENSG00000177225	770316	770307	767386	767373	AGTGTAAGCA	CTCTCTTTGCCAGG	TRUE	5.5973	9.7646	1.040000945	0.44248859	0.018756006
chr11	intron	18732703	18733234	-	ENSG00000110786	18733237	18733228	18732715	18732702	GAGGTAGGGA	TGGGCCCCTGCAGA	TRUE	8.0453	4.9494	0.613869033	0.261250464	0.018786844
chrX	intron	101248291	101248513	+	ENSG00000102385	101248288	101248297	101248501	101248514	TAGGTAAGGG	TTTTTAAACCTAGT	TRUE	10.1156	5.0043	1.218577612	0.518618889	0.01879061
chr7	intron	74189919	74190246	+	ENSG00000106682	74189916	74189925	74190234	74190247	GAGGTGATTG	TGTGTATCCTCAGG	TRUE	7.7847	7.5349	0.607667185	0.286245822	0.033763424
chr8	intron	38836288	38838469	+	ENSG00000147526	38836285	38836294	38838457	38838470	GAGGTAAAAG	TATTGTACTCTAGA	TRUE	7.4897	6.54	1.557	0.664	0.019039
chr16	intron	88717212	88717536	-	ENSG00000103335	88717539	88717530	88717224	88717211	GAGGTAAATT	CGTTCCCCTCCAGA	TRUE	8.5278	9.1473	0.858130808	0.365977277	0.019039062
chr1	intron	215611922	215618885	+	ENSG00000136636	215611919	215611928	215618873	215618886	AAGGTAACAC	TTTTCTTTTGCAGA	TRUE	7.1413	13.4416	1.3773	0.5874	0.01905
chr15	intron	101285802	101286907	-	ENSG00000131876	101286910	101286901	101285814	101285801	AAAGTAAGTA	TCCCCCTCTTAAGT	TRUE	9.8483	5.4461	0.6669	0.2845	0.01906
chr2	intron	112647465	112647652	+	ENSG00000144136	112647462	112647471	112647640	112647653	TTGGTATGTT	TGTTCTATTCCAGT	TRUE	6.7224	8.5651	0.623355228	0.275796935	0.023809067
chr15	intron	92953574	92955422	+	ENSG00000173575	92953571	92953580	92955410	92955423	ACGGTGTGTA	TTTTTTCTTATAGA	TRUE	6.8635	12.8169	0.698892416	0.312089515	0.025130077
chr2	intron	99403107	99403694	-	ENSG00000135945	99403697	99403688	99403119	99403106	CTGGTGAGCC	CATCTGCTCTTAGT	TRUE	7.7036	4.8424	2.52727261	1.079247699	0.01919622
chrX	intron	21591195	21594973	+	ENSG00000149970	21591192	21591201	21594961	21594974	GAGGTAAGAT	CTTGGCTCTTCAGG	TRUE	10.1098	9.2246	0.804970396	0.344477352	0.019450232
chr6	intron	169704089	169705132	-	ENSG00000130024	169705135	169705126	169704101	169704088	CAGGTAAAGA	ATTTTTTTCCTAGG	TRUE	8.5197	11.2612	0.899996966	0.38679867	0.019976763
chr18	intron	12351211	12351305	-	ENSG00000141385	12351308	12351299	12351223	12351210	TTGGTGAGAT	ATGATTTCTAAAGG	TRUE	6.8548	4.2252	1.118127937	0.480792922	0.020040353
chr14	intron	103728984	103729090	+	ENSG00000100711	103728981	103728990	103729078	103729091	GAGGTAAGAG	TTATTTGATGTAGA	TRUE	10.4568	5.7315	1.491679544	0.593667702	0.011982793
chrX	intron	68193953	68194464	-	ENSG00000079482	68194467	68194458	68193965	68193952	AAAGTGAGTC	TTGCTTGAAACAGT	TRUE	8.2945	2.7155	1.850814881	0.795988038	0.020062377
chr17	intron	44904555	44908063	-	ENSG00000131095	44908066	44908057	44904567	44904554	GAGGTAAGGA	TGTCTTCTCACAGG	TRUE	10.5656	10.7067	1.0396	0.4471	0.02007
chr6	intron	110892204	110892298	+	ENSG00000123505	110892201	110892210	110892286	110892299	TTGGTATGTT	AATTTTTATTTAGG	TRUE	6.7224	7.3575	0.859223428	0.399185603	0.031362643
chr5	intron	178612546	178612795	-	ENSG00000113240	178612798	178612789	178612558	178612545	ATGGTAAGTT	TGTGTCTCTCTAGA	TRUE	10.3917	7.9608	1.082124634	0.48064726	0.024360821
chr5	intron	91373839	91374113	-	ENSG00000113369	91374116	91374107	91373851	91373838	AAGGTAATTT	CTTTTTCCTTCAGC	TRUE	9.1744	11.8859	1.129800821	0.439085293	0.010079841
chr10	intron	38115291	38117389	+	ENSG00000075407	38115288	38115297	38117377	38117390	TGGGTGAGTT	CTCTGTATTTCAGA	TRUE	7.7786	8.6815	0.66197192	0.285892822	0.020588053
chr7	intron	1436857	1437534	-	ENSG00000164877	1437537	1437528	1436869	1436856	CCGGTGCGTC	GCCATCCCCACAGA	TRUE	5.6345	5.2989	1.623956499	0.62636126	0.009523037
chr10	intron	32286843	32286925	-	ENSG00000120616	32286928	32286919	32286855	32286842	GCTGTAAGTT	TTTTTTTTTTAAGC	TRUE	5.8793	10.5406	1.403161178	0.587677269	0.016956988
chr12	intron	101770192	101770405	-	ENSG00000111670	101770408	101770399	101770204	101770191	CAGGTACAGG	TTGTTGTTAAAAGG	TRUE	5.3842	4.8498	0.941434253	0.407513319	0.020877494
chr5	intron	146878196	147035168	-	ENSG00000156475	147035171	147035162	146878208	146878195	ACTGTGAGTC	GTCTCCTCCTCAGG	TRUE	5.5363	9.5425	0.675506186	0.239826039	0.004852727
chr10	intron	68032870	68033964	-	ENSG00000148634	68033967	68033958	68032882	68032869	ATGGTAGGTT	TTGGAATTTTCAGA	TRUE	7.8714	5.7758	1.325809359	0.669366338	0.047625689
chr1	intron	61088681	61132694	+	ENSG00000162599	61088678	61088687	61132682	61132695	CAGGTAAGTG	GTTTCTTTTTCAGG	TRUE	12.7631	12.6857	1.262112698	0.54938412	0.021600359
chr5	intron	113592179	113593302	+	ENSG00000047188	113592176	113592185	113593290	113593303	CAGGTATACA	CTGATTTATCTAGG	TRUE	5.7971	6.1246	0.764353705	0.363057914	0.035263315
chr2	intron	231457125	231457285	-	ENSG00000115053	231457288	231457279	231457137	231457124	TAGGTACTTA	TCTCTATTTGCAGG	TRUE	5.2117	11.1541	0.699446933	0.304538221	0.021633265
chr9	intron	97669707	97671085	+	ENSG00000136937	97669704	97669713	97671073	97671086	CAGGTATGTG	TGTGTGTCCACAGC	TRUE	10.3378	5.5318	0.7955	0.3465	0.021706
chr7	intron	81962496	81964055	-	ENSG00000153956	81964058	81964049	81962508	81962495	CTGGTAAGTC	TTGAAAAATTCAGG	TRUE	10.2199	1.842	1.095122708	0.477434311	0.021803929
chr11	intron	43330059	43330507	+	ENSG00000166181	43330056	43330065	43330495	43330508	GAGGTAAGAA	TTTCCCTTTGTAGA	TRUE	10.4917	10.9138	0.738256551	0.326951409	0.023945608
chr13	intron	114243990	114244889	+	ENSG00000130177	114243987	114243996	114244877	114244890	TGTGTAAGTA	CTTTAACTTTCAGA	TRUE	5.9618	9.0145	1.272536764	0.555394573	0.021950109
chr9	intron	77370936	77371025	+	ENSG00000197969	77370933	77370942	77371013	77371026	AAGGCAAGTA	TTTTTCTTTCCAGG	FALSE	6.6542	14.9048	1.365820955	0.597700381	0.022305414
chr2	intron	190931638	190953564	+	ENSG00000115419	190931635	190931644	190953552	190953565	AGGGTAAGCA	CTTTTCTTCACAGG	TRUE	8.7038	12.9436	1.2756	0.5583	0.022314
chr17	intron	2672758	2674059	+	ENSG00000007168	2672755	2672764	2674047	2674060	TGGGTAAGTA	TTTATATTGACAGC	TRUE	9.0683	6.1001	1.14286947	0.501137722	0.022575108
chr8	intron	39016195	39017218	+	ENSG00000168615	39016192	39016201	39017206	39017219	CAGGTAAGCA	ATGCAACATTCAGA	TRUE	11.1895	1.8608	0.793065219	0.343733496	0.021043139
chr11	intron	68165949	68166981	-	ENSG00000110066	68166984	68166975	68165961	68165948	CAAGTAAGTA	TCTTCCACTACAGA	TRUE	9.9406	9.6369	0.6813	0.2994	0.022888
chr2	intron	170848795	170849285	+	ENSG00000128683	170848792	170848801	170849273	170849286	ATGGTACCCA	TCTGTCCCCACAGG	TRUE	2.5312	9.5603	0.765634487	0.332147832	0.021160886
chr12	intron	112228812	112229697	-	ENSG00000173064	112229700	112229691	112228824	112228811	CAGGTACCAA	TGTCTGTCTCCAGG	TRUE	4.779	9.5889	1.141586552	0.503362643	0.023334041
chr4	intron	122893226	122897623	-	ENSG00000170917	122897626	122897617	122893238	122893225	TTGGTGTGTA	TTTTTTTACGTAGG	TRUE	6.1965	10.516	0.864200297	0.381134448	0.023363231
chr5	intron	14479351	14479918	+	ENSG00000038382	14479348	14479357	14479906	14479919	GAGGTAAGAC	CTTAAAACTGTAGG	TRUE	9.8457	3.6898	0.597527421	0.263925558	0.023574125
chr2	intron	229805884	229807707	-	ENSG00000153827	229807710	229807701	229805896	229805883	GAGGTAGTAG	TCTTTGCTCTCAGG	TRUE	4.7871	11.0645	0.819039999	0.3619988	0.023663509
chr16	intron	56499908	56500853	-	ENSG00000125124	56500856	56500847	56499920	56499907	CAGGTGACCC	TGATTGGCCATAGC	TRUE	6.1024	1.7999	0.793354074	0.382296968	0.037965239
chr15	intron	87925665	87929190	-	ENSG00000140538	87929193	87929184	87925677	87925664	AGGGTAAAGT	CTTTTTTGTTCAGT	TRUE	5.6521	9.8067	0.9842	0.4359	0.023952
chr12	intron	105177263	105188272	-	ENSG00000136044	105188275	105188266	105177275	105177262	GAGGTACGTT	TTTAAAATTTCAGG	TRUE	8.3943	6.7454	1.380898781	0.612136886	0.024079025
chr17	intron	50737027	50737286	+	ENSG00000108848	50737024	50737033	50737274	50737287	TTGGTAAGTG	AAATTTTAAAAAGA	TRUE	9.4947	-1.1211	1.049301786	0.465414167	0.024161282

chr4	intron	158240419	158241508	+	ENSG00000164124	158240416	158240425	158241496	158241509	CAGGTAAGAA	CTTTGTATTTCAGG	TRUE	11.4129	10.6022	0.862345943	0.287591683	0.002713051
chr15	intron	45364017	45364796	-	ENSG00000171766	45364799	45364790	45364029	45364016	ACGGTATGTT	TTGCTGTTTTTAGA	TRUE	7.3894	8.1759	0.874245413	0.384390133	0.022943745
chr5	intron	83512397	83519348	+	ENSG00000038427	83512394	83512403	83519336	83519349	AACGTAAGTG	ATTATTTTCTAGC	TRUE	8.921	8.0372	1.0042	0.4462	0.024395
chr19	intron	21098312	21098811	+	ENSG00000160352	21098309	21098318	21098799	21098812	TGGGTGAGAA	ACTTTTAATAAAGC	TRUE	6.7754	2.6656	0.855079891	0.318898567	0.007332494
chr2	intron	151870862	151872416	-	ENSG00000182389	151872419	151872410	151870874	151870861	CAGGTGAGGG	TATTGTCTTTTAGC	TRUE	10.5441	7.8527	0.66797088	0.297505386	0.024752754
chr3	intron	124674615	124678189	+	ENSG00000160145	124674612	124674621	124678177	124678190	CAAGTAAGTG	TTTGGTACAACAGG	TRUE	9.9057	5.8326	1.136656743	0.506465821	0.024813677
chr19	intron	35133765	35134032	-	ENSG00000153902	35134035	35134026	35133777	35133764	GCTGTGAGTG	GCCCCCTTGCCAGC	TRUE	5.3185	4.5525	1.38216311	0.616182211	0.024889865
chr5	intron	58457380	58457487	-	ENSG00000145632	58457490	58457481	58457392	58457379	AATGTAAGTA	TCTTTGCACACAGG	TRUE	9.5992	8.2866	0.85918006	0.415474471	0.038644809
chr10	intron	73379024	73379878	-	ENSG00000138279	73379881	73379872	73379036	73379023	TCTGTAAGAT	CTCTAACTTGCAGT	TRUE	4.0791	6.2881	1.15962194	0.517242126	0.024965734
chr8	intron	25368251	25368570	+	ENSG00000147459	25368248	25368257	25368558	25368571	TGGGTGAGTG	ACTTTCATTTCAGG	TRUE	8.1256	9.8675	0.917254893	0.355911427	0.009960433
chr16	intron	1760016	1760379	+	ENSG00000138834	1760013	1760022	1760367	1760380	CAAGTAAGAG	TGCTTTTTCAAAGA	TRUE	8.5206	6.8772	0.639867945	0.286097753	0.025316677
chr5	intron	95906783	95913770	-	ENSG00000118985	95913773	95913764	95906795	95906782	TAGGTTTGTA	CACTGGACTTTAGG	TRUE	4.8992	3.3289	0.894738257	0.44392355	0.043849848
chr3	intron	124633954	124637207	+	ENSG00000160145	124633951	124633960	124637195	124637208	ATGGTAGAAC	CGATGTCTTGCAGT	TRUE	3.2551	4.794	0.878575378	0.393547798	0.025585304
chr12	intron	57500491	57516434	+	ENSG00000166986	57500488	57500497	57516422	57516435	AGTGTGGCAA	CCCTTTATCTTAGG	TRUE	-1.1407	8.6769	0.9993	0.4483	0.025797
chr1	intron	236731315	236734432	+	ENSG00000077522	236731312	236731321	236734420	236734433	AAGGTGAGAT	TTTCTCCACACAGA	TRUE	10.0309	9.0622	0.611872832	0.28557648	0.032146169
chr1	intron	237590002	237590639	+	ENSG00000198626	237589999	237590008	237590627	237590640	GAGGTTTGCT	CTTCGTTTGCTAGG	TRUE	3.3239	7.5591	0.508606709	0.245255297	0.0380993
chr16	intron	3532842	3533098	+	ENSG00000103351	3532839	3532848	3533086	3533099	AATGTAAGTC	TCGCTTTCCAAAGA	TRUE	8.9532	5.3886	0.8173	0.3671	0.026001
chr4	intron	88043458	88046641	+	ENSG00000118762	88043455	88043464	88046629	88046642	CAGGTGTGTA	TATTTACATGCAGG	TRUE	9.4649	7.5929	1.226973777	0.551306074	0.02604257
chr2	intron	98616534	98618145	+	ENSG00000115446	98616531	98616540	98618133	98618146	GTGGTAAGTA	TTTTTTAAATCAGT	TRUE	9.9447	6.5445	0.559542346	0.257037655	0.029488879
chr10	intron	104005692	104005911	+	ENSG00000065613	104005689	104005698	104005899	104005912	TAGGTGAGTA	TAAATTTTATCAGA	TRUE	10.5539	4.7124	1.278444456	0.543057762	0.018564656
chr19	intron	19799451	19806030	-	ENSG00000081665	19806033	19806024	19799463	19799450	CAGGTAGGTG	TTTGACCTAACAGG	TRUE	10.2428	7.1668	2.617417067	1.065985464	0.014072924
chr14	intron	88618332	88618649	-	ENSG00000165521	88618652	88618643	88618344	88618331	CAGGTACAGT	TCTGATGAAAAAGG	TRUE	5.0372	-0.7583	1.0838	0.4929	0.027899
chr2	intron	170836884	170844044	+	ENSG00000128683	170836881	170836890	170844032	170844045	CATGTAAGTC	ACCACCTTTCCAGG	TRUE	9.0455	7.4724	1.7281	0.7864	0.027991
chr18	intron	34848384	34851830	+	ENSG00000134769	34848381	34848390	34851818	34851831	TCTGTAAGTA	ACATATTTTACAGC	TRUE	5.8461	5.5985	0.55260788	0.257837329	0.032093644
chr10	intron	3143502	3144291	-	ENSG00000107959	3144294	3144285	3143514	3143501	ATGGTAAGAA	CCGTGTCCCTCAGG	TRUE	9.3885	7.2685	1.093	0.4977	0.028078
chr11	intron	128981562	128981828	-	ENSG00000134909	128981831	128981822	128981574	128981561	AAGGTAATTT	GTTTACCTCACAGA	TRUE	9.1744	8.1265	0.492322761	0.224193838	0.028094148
chrX	intron	68206674	68210152	-	ENSG00000079482	68210155	68210146	68206686	68206673	AATGTGTGTG	CTTGTCCTTGTAGG	TRUE	6.2312	9.7896	1.105958202	0.503660001	0.028103195
chr1	intron	8014397	8015494	-	ENSG00000116285	8015497	8015488	8014409	8014396	AAAGTAAGTA	TCGTTCATTCCAGG	TRUE	9.8483	9.6174	0.8605	0.392	0.028141
chr2	intron	239969763	240005209	-	ENSG00000130414	240005212	240005203	239969775	239969762	ACTGTAAGTG	TGTGTTGGTTTAGG	TRUE	7.0552	4.8379	2.4483	1.1155	0.028172
chr1	intron	236734519	236735634	+	ENSG00000077522	236734516	236734525	236735622	236735635	AAGGTGAGGA	TCTCCCCCTTCAGA	TRUE	10.4867	10.477	0.521304528	0.256386237	0.042024432
chr12	intron	57533023	57533237	-	ENSG00000175203	57533240	57533231	57533035	57533022	CAGGTCAGGT	CCTTTCCCACCAGA	TRUE	6.9619	8.6662	1.079054719	0.492633197	0.028496569
chr15	intron	63061789	63062214	+	ENSG00000140416	63061786	63061795	63062202	63062215	AAGGTAGGCC	TTTCTAATTACAGT	TRUE	7.9309	8.3759	1.016349821	0.464215517	0.028568348
chr10	intron	26755742	26759061	-	ENSG00000136754	26759064	26759055	26755754	26755741	CAAGTAAGCT	TTGTTGCTACCAGT	TRUE	7.9502	5.9355	0.632731136	0.289233997	0.028698035
chr5	intron	59038972	59586328	-	ENSG00000113448	59586331	59586322	59038984	59038971	ATGGTGAGTA	TGTCTCTTCGCAGA	TRUE	9.8658	9.6279	1.5186	0.6945	0.028772
chr3	intron	9448007	9448387	+	ENSG00000168137	9448004	9448013	9448375	9448388	AAGGTAAGTC	TTTTTTACCTTAGT	TRUE	12.0597	9.0015	1.21682137	0.556550337	0.028789018
chr1	intron	181776229	181779442	+	ENSG00000198216	181776226	181776235	181779430	181779443	ATGGTGCGTA	GCTTCCTTTACAGC	TRUE	6.7652	9.3246	0.555942996	0.254471386	0.028911035
chr14	intron	67562036	67562136	+	ENSG00000054690	67562033	67562042	67562124	67562137	AAGGTAGGCA	ACCCGAATCACAGC	TRUE	8.5769	1.4986	0.666843026	0.261266909	0.010700082
chr14	intron	95193920	95194535	-	ENSG00000165959	95194538	95194529	95193932	95193919	TCGGTTAGTA	AATTTCACTGTAGG	TRUE	4.8154	6.2272	1.066413081	0.385207158	0.005632976
chr10	intron	80155913	80157966	-	ENSG00000122359	80157969	80157960	80155925	80155912	AGGGTATGTA	TTTCTGTGGCCAGG	TRUE	7.887	6.5311	1.297	0.5947	0.029193
chr7	intron	100205385	100211010	+	ENSG00000066923	100205382	100205391	100210998	100211011	AAGGTAGGGT	ATCTGCTTGGCAGG	TRUE	8.4923	6.1071	0.863378347	0.396725268	0.029535633
chr4	intron	113346023	113348275	+	ENSG00000145362	113346020	113346029	113348263	113348276	AAGGTATCGT	TTGGGGCGGAAAGG	TRUE	5.054	-3.2893	0.908896173	0.458559752	0.047471738
chr11	intron	10606773	10609727	-	ENSG00000072952	10609730	10609721	10606785	10606772	AAGGTAAAAT	TTCTTTTTCACAGT	TRUE	7.9716	11.3032	2.188338864	1.005721279	0.029563488
chr14	intron	49789344	49789495	-	ENSG00000165525	49789498	49789489	49789356	49789343	GGGGTAAATA	CTATGAATTAAAGT	TRUE	6.5163	0.4172	1.219654088	0.614893323	0.047309215
chrX	intron	21440782	21516484	+	ENSG00000149970	21440779	21440788	21516472	21516485	CAGGTATTAG	TGCTGGTTACCAGG	TRUE	5.8033	5.3957	0.6209	0.2858	0.029813
chr2	intron	151869236	151876425	-	ENSG00000182389	151876428	151876419	151869248	151869235	AGGGTACGTT	CACCTCTGCAAAGG	TRUE	6.8298	2.3612	0.5556	0.2562	0.030126
chr2	intron	71364253	71365428	+	ENSG00000075292	71364250	71364259	71365416	71365429	CAGGTACACT	ATCTTTTTACTAGA	TRUE	4.7399	7.697	0.522615926	0.259833108	0.044288255
chr14	intron	50983422	50985955	-	ENSG00000100505	50985958	50985949	50983434	50983421	CGGGTGAGCT	TATCTCTTTATAGG	TRUE	7.5064	10.3571	0.716989857	0.331125259	0.030363716
chr5	intron	143057748	143057857	+	ENSG00000145819	143057745	143057754	143057845	143057858	AAAGTAGGTT	ACTATTGCATCAGG	TRUE	6.9461	4.5317	0.99803887	0.462090691	0.030785575
chr2	intron	238188258	238189852	-	ENSG00000132323	238189855	238189846	238188270	238188257	AAGGTTTTCA	CTCTTTTCCTTAGC	TRUE	1.3853	9.3187	0.809191741	0.375148163	0.031006255
chr17	intron	81694854	81694923	+	ENSG00000185359	81694851	81694860	81694911	81694924	GAGGTAAGGC	TCATTGCCTGCAGC	TRUE	9.9196	6.5866	1.506816381	0.698664837	0.031028386
chr10	intron	68345912	68346006	-	ENSG00000204130	68346009	68346000	68345924	68345911	AAGGTAAGGG	TTCTGTTTAATAGC	TRUE	11.3596	6.8055	0.519737585	0.241853992	0.031636578
chr2	intron	97158168	97158587	+	ENSG00000135976	97158165	97158174	97158575	97158588	ATTGTAAGCT	GTTTTTACTGTAGT	TRUE	5.6767	7.3094	0.557827883	0.260264017	0.032087779
chr12	intron	108538204	108538933	-	ENSG00000075856	108538936	108538927	108538216	108538203	CTAGTAAGAT	TTCTTTATCATAGG	TRUE	6.2415	10.1699	1.469595091	0.682564504	0.031314797
chr12	intron	49643017	49643222	+	ENSG00000110844	49643014	49643023	49643210	49643223	TCAGTGAGTA	CTGATCTGCCCAGG	TRUE	5.1874	5.378	1.155071926	0.536864244	0.031435507
chr11	intron	46778314	46778459	-	ENSG00000175216	46778462	46778453	46778326	46778313	CAGGTAGGTT	TTCTGTTTTTCAGT	TRUE	9.8958	10.4041	0.99169653	0.461441728	0.031624394
chr6	intron	34879737	34879966	-	ENSG00000064995	34879969	34879960	34879749	34879736	AAGGTGAGTA	TTCCTTTCCTCAGC	TRUE	11.7979	10.3609	0.666718068	0.31026575	0.031645395
chr12	intron	98735635	98751354	-	ENSG00000185046	98751357	98751348	98735647	98735634	GTGGTAAGTA	CTCTTTTTTACAGG	TRUE	9.9447	13.2737	0.6325	0.2945	0.031753
chr5	intron	79640966	79641104	+	ENSG00000164329	79640963	79640972	79641092	79641105	CACGTATGTT	TCTGTTTCTTTAGA	TRUE	6.241	9.8751	1.122581702	0.522895223	0.031804602
chr8	intron	491671	492458	-	ENSG00000180190	492461	492452	491683	491670	CTGGTGAGCA	TTTTTGTTTACAGA	TRUE	8.3496	12.2882	0.8298	0.3882	0.032565
chr10	intron	68376973	68379423	-	ENSG00000204130	68379426	68379417	68376985	68376972	AAGGTACAGC	TGAATTACACCAGA	TRUE	4.6808	2.1908	1.3451	0.6298	0.032697
chr15	intron	25356057	25356690	-	ENSG00000114062	25356693	25356684	25356069	25356056	CCAGTAAGTT	TTCTGGTTGCTAGG	TRUE	7.0496	6.0863	1.405995171	0.660230308	0.033208568
chr2	intron	127738055	127763061	-	ENSG00000136709	127763064	127763055	127738067	127738054	GAGGTACGTG	CTTGTTTTGATAGA	TRUE	8.7413	7.2806	0.684820209	0.321683358	0.033265491
chr4	intron	36114288	36117060	-	ENSG00000047365	36117063	36117054	36114300	36114287	CAGGTAAGTT	ACTCTCTTACAGA	TRUE	10.0227	10.0479	0.715317511	0.28058753	0.010792206
chr18	intron	36167223	36170062	+	ENSG00000134759	36167220	36167229	36170050	36170063	AAGGTAATTA	CTGTCTGTATTAGG	TRUE	9.5563	5.646	0.929710599	0.437567658	0.033609632
chr1	intron	181779540	181781426	+	ENSG00000198216	181779537	181779546	181781414	181781427	AAGGTTTGCC	CCTCCATGAGCAGT	TRUE	3.8887	2.1786	0.82262626	0.387455554	0.033741284
chr14	intron	96334502	96341521	-	ENSG00000066739	96341524	96341515	96334514	96334501	AAGGTCAGTG	TTTTTCCCTTAAGT	TRUE	8.5278	8.5845	0.476323054	0.235927881	0.043493977
chr1	intron	109231022	109231630	+	ENSG00000031698	109231019	109231028	109231618	109231631	AAGGTAAGAG	TCTGCTCCTCTAGG	TRUE	11.2857	9.3783	0.728820634	0.312109524	0.019535907
chr10	intron	95416186	95421960	-	ENSG00000095637	95421963	95421954	95416198	95416185	CAGGTAGGCA	AATATTGCTAAAGG	TRUE	8.6692	2.0302	0.489641154	0.232650029	0.035323931
chr14	intron	20474949	20475061	+	ENSG00000198805	20474946	20474955	20475049	20475062	AAGGTATGTA	ACCTGTGTCCTAGG	TRUE	10.2804	4.9456	1.584876126	0.804010836	0.048699587
chr17	intron	4075179	4075262	-	ENSG00000074755	4075265	4075256	4075191	4075178	AAGGTAAGAC	TATGCCTTCACAGG	TRUE	10.6746	8.498	0.776232784	0.370131024	0.035977309
chr2	intron	121728357	121728476	-	ENSG00000155438	121728479	121728470	121728369	121728356	CAGGTAAAAT	AATTTATTTTAAGG	TRUE	8.0639	5.5881	0.650327092	0.310133312	0.035999988
chr1	intron	66690317	66694438	+	ENSG00000118473	66690314	66690323	66694426	66694439	TTGGTAAAAA	CTTTGGTTCGTAGT	TRUE	5.1774	6.0383	0.732590548	0.349820814	0.036243237
chr9	intron	123073678	123074284	+	ENSG00000011454	123073675	123073684	123074272	123074285	CAGGTAAAAA	TTTGCTATTTCAGG	TRUE	8.4458	10.6585	0.619868915	0.296051866	0.03627915
chr1	intron	66671020	66671943	+	ENSG00000118473	66671017	66671026	66671931	66671944	CCAGTAAGTA	TCCTGTGCATCAGG	TRUE	7.4315	6.0473	1.30856562	0.625892381	0.036553185
chr5	intron	87394485	87395043	-	ENSG00000134480	87395046	87395037	87394497	87394484	GAGGTATGTT	TTTTCCTTCTTAGG	TRUE	9.0696	12.2457	0.9818	0.4702	0.036819
chr7	intron	82951491	82951855	-	ENSG00000186472	82951858	82951849	82951503	82951490	CAGGTCAGTA	CCCTGTATTTCAGG	TRUE	8.655	8.5921	0.990825795	0.450798749	0.027953826
chr2	intron	53887468	53887857	-	ENSG00000068878	53887860	53887851	53887480	53887467	AAGGTAGGTA	TTTTATTTTGCAGT	TRUE	10.1854	10.8003	0.872763907	0.41849429	0.037025224
chr12	intron	119718862	119720477	-	ENSG00000122966	119720480	119720471	119718874	119718861	AAGGTGAGTC	TTTCCATTGCTAGG	TRUE	11.1519	7.7001	0.790631328	0.379125007	0.037032041
chr12	intron	119712696	119713202	-	ENSG00000122966	119713205	119713196	119712708	119712695	AAGGTAAATT	TCTTTGCACCAAGC	TRUE	9.3567	3.5333	0.755721245	0.362554216	0.03712048
chr1	intron	204984143	204985909	+	ENSG00000163531	204984140	204984149	204985897	204985910	CGAGTGAGGA	CTCTGTCCCGAAGG	TRUE	5.3282	5.6408	0.549586819	0.263766627	0.037195644
chr17	intron	59838398	59839767	+	ENSG00000062716	59838395	59838404	59839755	59839768	CAGGTAAGAC	TTTATTTCTACAGG	TRUE	10.7669	12.1524	1.04580873	0.485006428	0.031061958
chr10	intron	68383915	68384050	-	ENSG00000204130	68384053	68384044	68383927	68383914	GAGGTAAGTA	GTTTTCTTCCTAGG	TRUE	11.8768	10.848	0.900748031	0.432839523	0.037432006
chr8	intron	61653661	61680967	-	ENSG00000198363	61680970	61680961	61653673	61653660	TAGGCAAGTA	TAAAAAAATGCAGG	FALSE	5.4102	-1.2316	0.6187	0.2974	0.037497
chr17	intron	44903560	44904441	-	ENSG00000131095	44904444	44904435	44903572	44903559	GAGGTAGCGT	CTCCCCTTTCTAGA	TRUE	4.1301	9.3989	1.0781	0.5183	0.037516
chr3	intron	4717400	4725545	+	ENSG00000150995	4717397	4717406	4725533	4725546	GAGGTCCTAA	TACTCCCAATTAGC	TRUE	0.0987	2.4884	1.284223423	0.617438837	0.037532866
chr9	intron	33303269	33307193	+	ENSG00000086102	33303266	33303275	33307181	33307194	GTGGTAAGTT	TGATATGTTCTAGG	TRUE	9.5628	5.0163	1.277739133	0.615206803	0.03780837
chr11	intron	120429518	120429711	+	ENSG00000196914	120429515	120429524	120429699	120429712	CAGGTATTAA	TATAACTTTTCAGT	TRUE	5.8382	5.9408	1.112696867	0.535880375	0.037857858
chrX	intron	101886732	101897867	-	ENSG00000166432	101897870	101897861	101886744	101886731	TGGGTAAGGT	TTTGTTTTTTCAGC	TRUE	7.3752	11.6513	0.705516779	0.306683648	0.021421562
chr14	intron	74821138	74824255	+	ENSG00000119596	74821135	74821144	74824243	74824256	CTGGTAGGAG	GTGTACGATTTAGG	TRUE	6.9256	2.3895	0.6634	0.3202	0.038282
chr19	intron	7542068	7542560	+	ENSG00000032444	7542065	7542074	7542548	7542561	CAGGTTTGGA	TGCCTGCCTGCAGG	TRUE	4.9243	7.5267	1.207449427	0.58384996	0.03863234
chr4	intron	42455420	42455524	-	ENSG00000124406	42455527	42455518	42455432	42455419	GTGGTAAGTT	TTGTGCGCTTTAGA	TRUE	9.5628	5.7515	0.855753679	0.414217444	0.038832939
chr7	intron	134934078	134935687	+	ENSG00000122786	134934075	134934084	134935675	134935688	CAGGTACAGT	TGAAAATAAAAAGG	TRUE	5.0372	-4.745	1.077778557	0.522215418	0.039031166
chr1	intron	12348974	12349163	+	ENSG00000048707	12348971	12348980	12349151	12349164	ACAGTATGTT	ATTTCTGTGGCAGA	TRUE	4.532	6.0123	0.75067619	0.363957417	0.039156495
chr14	intron	66996219	67023632	+	ENSG00000171723	66996216	66996225	67023620	67023633	GAGGTATATT	GCTCTTTCTACAGG	TRUE	6.1025	10.74	0.757842245	0.367444369	0.039163203
chr2	intron	27055254	27055681	+	ENSG00000084693	27055251	27055260	27055669	27055682	CCGGTAAGTG	TGTTTTCCTACAGC	TRUE	10.254	10.2512	1.193610001	0.554016553	0.031203621
chr1	intron	71065753	71066775	-	ENSG00000132485	71066778	71066769	71065765	71065752	AAGGTTTGGG	TCTATTCCATAAGC	TRUE	4.7971	3.5025	1.157954447	0.562728086	0.039614014
chr1	intron	31736391	31736572	-	ENSG00000121753	31736575	31736566	31736403	31736390	GGGGTGAGCA	CCTGCTCCCCCAGG	TRUE	6.9671	8.7146	0.764154258	0.371455309	0.039667924
chr2	intron	113733404	113734585	-	ENSG00000115084	113734588	113734579	113733416	113733403	AAGGTAAGCA	TATCTTAATAAAGA	TRUE	11.0972	3.353	0.485286645	0.247343727	0.049763307
chr8	intron	23441724	23442006	-	ENSG00000197217	23442009	23442000	23441736	23441723	ATGGTAAGTG	CTGTTCTTTCTAGA	TRUE	10.7387	9.7469	0.855502642	0.416137016	0.039799906
chr3	intron	51356494	51356961	+	ENSG00000088538	51356491	51356500	51356949	51356962	CTGGTGAGAC	TGTGTGCCCCTAGG	TRUE	7.927	5.7855	1.207991503	0.587684822	0.039829857

chr2	intron	206561213	206562202	+	ENSG00000114948	206561210	206561219	206562190	206562203	GAGGTAAATT	CTCTGAAAAACAGT	TRUE	8.5278	0.2118	0.482912073	0.234975794	0.039863874
chr1	intron	23056039	23057483	+	ENSG00000004487	23056036	23056045	23057471	23057484	AGGGTAAGAA	ATTTCTGAAACAGG	TRUE	8.9272	5.0016	1.405537111	0.683989692	0.039887719
chr2	intron	97179934	97181597	+	ENSG00000135976	97179931	97179940	97181585	97181598	CAGGTAATTT	TTTTGCTTTTCAGT	TRUE	9.2667	10.9661	0.772395417	0.376201164	0.040058548
chr11	intron	45946004	45946075	-	ENSG00000135365	45946078	45946069	45946016	45946003	AAGGTATGTA	TCTTGGTATATAGG	TRUE	10.2804	6.5439	1.518167866	0.739931415	0.040192179
chr6	intron	134982563	134985340	-	ENSG00000112339	134985343	134985334	134982575	134982562	AAGGTAAGTG	GTATGTTTTGCAGA	TRUE	12.6708	7.4249	1.221471757	0.595494222	0.040248453
chr6	intron	126008473	126011252	+	ENSG00000066651	126008470	126008479	126011240	126011253	TGGGTGAGTA	TCTTTCCCTTCAGG	TRUE	8.1605	12.9367	1.693853043	0.82659628	0.040443514
chr2	intron	209978709	209982178	+	ENSG00000144406	209978706	209978715	209982166	209982179	AAAGTAGGTC	TTGCCCCTTTTAGA	TRUE	6.682	8.5084	0.960130818	0.46919328	0.040722545
chr17	intron	48930613	48931642	-	ENSG00000159210	48931645	48931636	48930625	48930612	CTGGTATGCA	CTTTTCCCTCCAGG	TRUE	6.8321	12.9266	0.86552639	0.423080147	0.040778605
chr5	intron	132706460	132708906	-	ENSG00000131437	132708909	132708900	132706472	132706459	CTAGTGAGTG	TTCTTATTGCAAGA	TRUE	7.0658	5.1837	0.46512959	0.227535655	0.040933271
chr18	intron	3656114	3729134	-	ENSG00000170579	3729137	3729128	3656126	3656113	CGGGTGAGTG	GTTTTTATTCAAGG	TRUE	9.4619	8.0601	1.169	0.5725	0.041159
chr11	intron	47413687	47414424	+	ENSG00000165915	47413684	47413693	47414412	47414425	AAGGTGAGGG	CCCCTCCCTGCAGA	TRUE	10.4518	8.7523	0.9866	0.4834	0.041243
chr14	intron	70584131	70584816	-	ENSG00000133997	70584819	70584810	70584143	70584130	TCAGTGAGTA	TCTTTTTGTTCAGT	TRUE	5.1874	9.757	0.7747	0.3798	0.041375
chr6	intron	108876351	108879927	+	ENSG00000118690	108876348	108876357	108879915	108879928	AGGGTCAGTA	CATCTCCTTCTAGC	TRUE	6.1693	7.5562	0.561511364	0.275483479	0.041522339
chr2	intron	222624776	222628836	-	ENSG00000116120	222628839	222628830	222624788	222624775	CCAGTAAGTG	CTTTCTATTTAAGG	TRUE	7.3966	8.6004	1.627234074	0.798989193	0.041688551
chr1	intron	240330750	240333886	+	ENSG00000155816	240330747	240330756	240333874	240333887	AGTGTAAGTA	TCTTTCTGCCTAGG	TRUE	7.2058	9.1141	1.144090274	0.561842641	0.041718435
chr2	intron	43794644	43795886	+	ENSG00000138036	43794641	43794650	43795874	43795887	CCTGTGAGTT	ATATGTTTATTAGC	TRUE	5.8927	3.3572	1.1995	0.5891	0.041733
chr2	intron	99394586	99394718	+	ENSG00000158417	99394583	99394592	99394706	99394719	TCAGTAAGTA	TTTAACCTTTTAGG	TRUE	6.0952	8.6263	1.185568618	0.582879513	0.041953033
chr4	intron	86101218	86101893	-	ENSG00000109339	86101896	86101887	86101230	86101217	AGGGTAAGAA	TCCTTACTGTTAGG	TRUE	8.9272	6.8781	0.745555819	0.367072903	0.042246497
chr2	intron	113735859	113742691	-	ENSG00000115084	113742694	113742685	113735871	113735858	GTGGTAGGTC	TCTTTTTCTTTAGT	TRUE	6.7784	10.9698	1.1779	0.58	0.042258
chr7	intron	137597908	137599822	-	ENSG00000157680	137599825	137599816	137597920	137597907	TGCGTAAGTC	ATTTCCTCTACAGG	TRUE	4.6725	11.1383	0.892965204	0.439810189	0.042321763
chr16	intron	19542761	19542877	+	ENSG00000103540	19542758	19542767	19542865	19542878	CAAGTGGGTA	TTCTTTCTCCTAGG	TRUE	6.5125	11.8512	0.547614376	0.254370982	0.031333001
chr5	intron	177301988	177302127	-	ENSG00000169228	177302130	177302121	177302000	177301987	TGGGTGAGTG	TTTTTCCTGCCAGA	TRUE	8.1256	10.5936	1.389279587	0.685353027	0.042652117
chr2	intron	127057602	127059010	-	ENSG00000136717	127059013	127059004	127057614	127057601	CAGGTAGGAG	CGCCGACCCACAGC	TRUE	8.8577	2.3969	0.739072638	0.364853657	0.042798745
chr18	intron	57607334	57607443	-	ENSG00000134440	57607446	57607437	57607346	57607333	GAAGTAAGTA	TGCATTTGAACAGG	TRUE	9.0194	3.9946	1.321418872	0.652738442	0.042926623
chr5	intron	162149314	162151729	+	ENSG00000113327	162149311	162149320	162151717	162151730	CCTGTATGTA	TACAAACCCAAAGC	TRUE	4.7571	-1.9729	0.7939	0.3924	0.043049
chr22	intron	32857361	32858016	+	ENSG00000100234	32857358	32857367	32858004	32858017	CAGGTAATGG	TTTCCTCCTGTAGG	TRUE	8.3025	11.1517	0.652395066	0.322560672	0.043119571
chrX	intron	24562118	24562442	-	ENSG00000102230	24562445	24562436	24562130	24562117	CCTGTGAGCA	ACTCTCCGTACAGC	TRUE	4.6661	5.6174	0.4989	0.2468	0.043198
chr1	intron	237732150	237733704	+	ENSG00000198626	237732147	237732156	237733692	237733705	ATGGTATGGT	TTGCTTTCCCCAGC	TRUE	6.6552	9.0071	0.517049329	0.255939871	0.043362808
chr1	intron	66684188	66689147	+	ENSG00000118473	66684185	66684194	66689135	66689148	CAGGTCTGTC	GTTTGTTTTTCAGG	TRUE	5.5837	11.364	1.315459818	0.6515039	0.043475504
chr1	intron	11998879	11998987	+	ENSG00000116688	11998876	11998885	11998975	11998988	ACGGTAACTC	ACCCTTTATCTAGG	TRUE	6.0173	6.7358	0.983207755	0.487126917	0.043551588
chr2	intron	218592403	218592615	+	ENSG00000144580	218592400	218592409	218592603	218592616	TTGGTGAGTT	TTGGGGGAAACAGG	TRUE	8.2399	-0.8514	1.010194861	0.500532773	0.043566356
chr19	intron	32464785	32468730	+	ENSG00000178904	32464782	32464791	32468718	32468731	AAGGTAAGAC	TTTTTAATTTCAGT	TRUE	10.6746	9.5484	1.181257441	0.585405224	0.043607329
chr4	intron	55424644	55452238	+	ENSG00000134851	55424641	55424650	55452226	55452239	CTGGTAAGTC	TTCCATCTTGCAGG	TRUE	10.2199	10.2184	0.6555	0.3255	0.044017
chr18	intron	34838838	34848295	+	ENSG00000134769	34838835	34838844	34848283	34848296	ATGGTTAGTG	TTGTTCTTAATAGC	TRUE	6.6015	7.0332	0.59991015	0.297900278	0.04403104
chr13	intron	35793480	35805698	-	ENSG00000133083	35805701	35805692	35793492	35793479	AATGTAAGTG	TCTTTTTCTCCAGG	TRUE	9.5643	13.9316	1.354612935	0.673274456	0.044222328
chr19	intron	1425961	1428841	+	ENSG00000071626	1425958	1425967	1428829	1428842	AAAGTGTGTA	TCGGTTTGTTTAGG	TRUE	6.5152	5.9972	0.706095175	0.351249999	0.044406283
chr3	intron	37121537	37121634	-	ENSG00000093167	37121637	37121628	37121549	37121536	CTGGTTTGTA	TCATTTTTATCAGG	TRUE	4.3034	9.4468	1.616109199	0.664379842	0.014994665
chr21	intron	33582056	33582164	-	ENSG00000159147	33582167	33582158	33582068	33582055	GGAGTATGTG	AGCAATTTCCTAGG	TRUE	4.1658	3.1988	1.089141603	0.542499562	0.044681995
chr1	intron	1312369	1312439	-	ENSG00000127054	1312442	1312433	1312381	1312368	AGCGTGAGTG	CCCTGCCCCACAGA	TRUE	5.6198	6.9323	0.802221967	0.399610529	0.044695431
chr6	intron	111696457	111699514	-	ENSG00000010810	111699517	111699508	111696469	111696456	TTGGTAAGGC	GGGATGTTTGTAGG	TRUE	7.5724	2.6152	1.162230737	0.580043077	0.045102498
chr1	intron	150578492	150578842	-	ENSG00000143384	150578845	150578836	150578504	150578491	AAGGTAAGGG	TTTTCTTTCTCAGG	TRUE	11.3596	13.8725	0.679806045	0.339533446	0.045265702
chr6	intron	143857445	143857751	+	ENSG00000135521	143857442	143857451	143857739	143857752	ACAGTATGTG	ACCTTTACTTTAGG	TRUE	4.879	7.6126	0.84946886	0.424942809	0.04560627
chr11	intron	6609846	6609935	+	ENSG00000166333	6609843	6609852	6609923	6609936	ATGGTGAGGC	TTCTCTTCCTCAGA	TRUE	7.9086	11.3447	0.536180947	0.268420965	0.045766824
chr10	intron	114448027	114451623	-	ENSG00000099204	114451626	114451617	114448039	114448026	ATGGTAAAAC	GATTCCCATCTAGA	TRUE	5.7754	4.8121	0.5613	0.281	0.045771
chr12	intron	121420335	121420576	+	ENSG00000170633	121420332	121420341	121420564	121420577	CGGGTGAGGC	TCTGTGGTTTCAGA	TRUE	7.5396	7.197	0.568401235	0.285176111	0.046244076
chr6	intron	99408272	99409172	-	ENSG00000132424	99409175	99409166	99408284	99408271	TTGGTAGCTA	CCACGTGAAACAGA	TRUE	3.476	-0.2127	0.771488312	0.387653273	0.046574371
chr10	intron	93777451	93777545	+	ENSG00000108231	93777448	93777457	93777533	93777546	TTTGTAAGTA	TAACTTATTGCAGA	TRUE	6.4231	6.333	0.528825972	0.265882181	0.046706885
chr9	intron	83971357	83971671	-	ENSG00000165119	83971674	83971665	83971369	83971356	ATGGTAAGAA	TTATCTCTAACAGG	TRUE	9.3885	8.8277	0.929049145	0.448003187	0.038102009
chr2	intron	98832513	98832861	-	ENSG00000196872	98832864	98832855	98832525	98832512	CAGGTAAATG	TATTCCAATCTAGT	TRUE	9.796	4.3033	0.917158722	0.461560487	0.046913266
chr1	intron	145994168	145994280	-	ENSG00000265972	145994283	145994274	145994180	145994167	AAGGTGAGCC	TTTTCTTTTCTAGC	TRUE	9.5434	11.9775	0.859201769	0.432638694	0.047038117
chr17	intron	7223876	7223967	+	ENSG00000072778	7223873	7223882	7223955	7223968	AAGGTACAGG	GGCCCTGTGCTAGG	TRUE	5.2919	2.0929	0.529928428	0.266895319	0.047085634
chr2	intron	115753298	115762571	+	ENSG00000175497	115753295	115753304	115762559	115762572	AAAGTGAGTA	TGTTTTGTTTCAGA	TRUE	8.9405	10.2245	0.834785516	0.421003903	0.047384737
chr11	intron	18733373	18733555	-	ENSG00000110786	18733558	18733549	18733385	18733372	CGGGTACGTA	TGGCTGGCCGCAGG	TRUE	7.304	3.6115	0.530750084	0.267718496	0.047424073
chr13	intron	39039289	39039563	+	ENSG00000188811	39039286	39039295	39039551	39039564	CAAGTAAGTA	TGTATACCTTCAGA	TRUE	9.9406	6.8338	0.813633853	0.411666569	0.048105081
chr11	intron	47168044	47168122	-	ENSG00000149182	47168125	47168116	47168056	47168043	AAAGTAAGGC	ACTGTCTCCCTAGG	TRUE	7.8911	6.9932	0.506097217	0.256070158	0.048109422
chr10	intron	91487779	91491199	+	ENSG00000165338	91487776	91487785	91491187	91491200	AGGGTAAGTC	CTTATTTAATCAGC	TRUE	9.6663	5.4901	1.405586245	0.711221585	0.048120964
chr18	intron	58341798	58342905	+	ENSG00000049759	58341795	58341804	58342893	58342906	GAGGTGAGAC	TGTTATTCTTTAGG	TRUE	8.9379	9.3279	0.650177806	0.29407183	0.027039372
chr4	intron	75790239	75790642	+	ENSG00000138768	75790236	75790245	75790630	75790643	AAGGTAGAAA	TTAAATGCAACAGA	TRUE	5.8332	1.676	0.958359001	0.485339116	0.048311598
chr2	intron	219300260	219300942	-	ENSG00000054356	219300945	219300936	219300272	219300259	AAAGTGAGTC	CTCTTCTGAACAGA	TRUE	8.2945	6.4737	1.122780238	0.56878392	0.048381587
chr3	intron	197116084	197119409	-	ENSG00000075711	197119412	197119403	197116096	197116083	AAGGTAAGGA	TTTTAATTTTTAGG	TRUE	11.3945	10.1175	0.798933952	0.322691648	0.013292191
chr1	intron	204957827	204962107	+	ENSG00000163531	204957824	204957833	204962095	204962108	CCAGTAAGTG	TTGTTTGTTACAGA	TRUE	7.3966	9.7766	1.576918695	0.646289155	0.014688951
chr2	intron	113729506	113731583	-	ENSG00000115084	113731586	113731577	113729518	113729505	TAGGTAAGTA	TCCATTTTTACAGG	TRUE	11.4617	10.7494	1.280641206	0.650126818	0.048857523
chr9	intron	37780881	37781985	-	ENSG00000107371	37781988	37781979	37780893	37780880	AAAGTAAGTC	CTCTTCCCACCAGG	TRUE	9.2023	9.288	0.654097203	0.266673644	0.014174899
chr15	intron	43873223	43874118	-	ENSG00000171877	43874121	43874112	43873235	43873222	CAGGTGAATA	CAAATCTGAGCAGA	TRUE	8.9231	-0.097	0.5146	0.2617	0.049225
chr1	intron	51814814	51816311	-	ENSG00000078618	51816314	51816305	51814826	51814813	AAAGCAAGTA	TCCCCACCTGTAGA	FALSE	3.7968	6.2858	0.63747981	0.322778428	0.048270814
chr8	intron	102299548	102299999	-	ENSG00000104517	102300002	102299993	102299560	102299547	CTGGTAAGAG	TGTTTACTGAAAGA	TRUE	9.4459	3.3407	0.523389526	0.266830024	0.049819662
chr12	intron	48921418	48931412	+	ENSG00000139537	48921415	48921424	48931400	48931413	CAGGTGATAA	TATCCACCCACAGG	TRUE	7.3557	7.1919	0.4419	0.2253	0.049881
chr12	intron	13211541	13216443	+	ENSG00000134531	13211538	13211547	13216431	13216444	GTGGTCCACA	AAACTGTGGGAAGA	TRUE	-1.8745	-5.2276	0.9521	0.4856	0.049925
chr15	intron	63723356	63725291	-	ENSG00000103657	63725294	63725285	63723368	63723355	TGGGTAATGT	GTATTTTTTTTAGA	TRUE	4.2258	8.9071	1.182040078	0.602956675	0.049948317

<u>Worksheet 2</u>: IR events negatively associated with AUD

chrom	feature	intron_start	intron_end	strand	geneid	donor_start	donor_end	acceptor_start	acceptor_end	donorSEQ	acceptorSEQ	canonical	donor_junc.score	acceptor_junc.score	beta	se	p.value
chr12	intron	9109406	9109866	-	ENSG00000175899	9109869	9109860	9109418	9109405	TTGGTATGGA	CACCAATCAACAGT	TRUE	5.7931	0.6427	-1.336973624	0.383141801	0.000483924
chr10	intron	13858913	14024353	-	ENSG00000151474	14024356	14024347	13858925	13858912	TTTGTAAGTA	TCTTCCCTCTTAGA	TRUE	6.4231	10.0072	-1.1656	0.3519	0.0009258
chr3	intron	196062646	196062853	-	ENSG00000072274	196062856	196062847	196062658	196062645	GAGGTATTCT	CCTTCTCTTCTAGG	TRUE	4.3117	11.3408	-0.871137184	0.297727173	0.003433974
chr1	intron	1046958	1047326	+	ENSG00000188157	1046955	1046964	1047314	1047327	CCGGTGAGTG	ACCTCCCCCACAGC	TRUE	9.3462	6.0809	-1.783924445	0.581737302	0.002165472
chr7	intron	143267734	143268090	+	ENSG00000197448	143267731	143267740	143268078	143268091	GGAGTGAGCA	CTGCTTTCTCCAGG	TRUE	4.1097	10.6833	-1.318806638	0.418515011	0.001626249
chr17	intron	44906165	44908063	-	ENSG00000131095	44908066	44908057	44906177	44906164	GAGGTAAGGA	TGTGTCATACCAGG	TRUE	10.5656	5.9525	-0.5902	0.2111	0.0051772
chr2	intron	171844509	171855833	-	ENSG00000115840	171855836	171855827	171844521	171844508	TTGGTAAGGG	TTTCCCTTTTAAGA	TRUE	8.1835	9.1814	-0.832068447	0.277767371	0.002739426
chr1	intron	154273599	154273749	+	ENSG00000143575	154273596	154273605	154273737	154273750	CTGGTGTGTG	TTTCCCATCCCAGC	TRUE	7.4979	8.7459	-2.15340103	0.834383087	0.009856306
chr11	intron	68081458	68097018	-	ENSG00000110721	68097021	68097012	68081470	68081457	ATGGTGAGTT	TTCTCATTCATAGA	TRUE	9.4839	8.5564	-0.814006038	0.293627232	0.005567146
chr17	intron	64503572	64503802	-	ENSG00000108654	64503805	64503796	64503584	64503571	ATTGTAAGTA	TCTCTTTCCTTAGT	TRUE	7.6671	9.1767	-1.798826679	0.79035514	0.022847647
chr19	intron	9338583	9339192	+	ENSG00000188321	9338580	9338589	9339180	9339193	CAGGTAAGTA	TGTGATGGTTTAGG	TRUE	12.798	2.5737	-0.715168856	0.24865725	0.004025927
chr1	intron	207071574	207078953	+	ENSG00000123836	207071571	207071580	207078941	207078954	ACTGTAAGTA	CTCTGGCTCGTAGG	TRUE	7.0901	6.3574	-1.2179	0.4629	0.0085143
chr19	intron	10803669	10805915	+	ENSG00000079805	10803666	10803675	10805903	10805916	CTGGTGGGTA	TCTTTGGTTTCAGT	TRUE	7.4378	8.2917	-1.244814211	0.501852427	0.01312208
chr6	intron	167010894	167013507	+	ENSG00000213066	167010891	167010900	167013495	167013508	AAGGTGAGTT	TTCTCATGCTCAGA	TRUE	11.416	7.008	-0.720532654	0.283514317	0.011039909
chr12	intron	47748543	47748818	-	ENSG00000079337	47748821	47748812	47748555	47748542	CCGGTAATAC	CCATTTCTGCTAGG	TRUE	5.1084	6.4859	-1.39021255	0.497462885	0.005196307
chr17	intron	44321232	44321433	-	ENSG00000013306	44321436	44321427	44321244	44321231	GCCGTGAGCA	AATCCCCACACAGT	TRUE	3.1016	2.6472	-0.975081341	0.405085303	0.016079711
chr13	intron	95859676	95860787	-	ENSG00000102595	95860790	95860781	95859688	95859675	AAGGTAGGTA	TTCTTTTATACAGA	TRUE	10.1854	10.807	-1.312140735	0.547952564	0.016637386
chr5	intron	150245202	150247771	-	ENSG00000070808	150247774	150247765	150245214	150245201	CCGGTAGGTG	TTCTCCTCCACAGG	TRUE	7.7337	11.3499	-0.838181951	0.3181048	0.008415614
chrY	intron	19716474	19716595	-	ENSG00000012817	19716598	19716589	19716486	19716473	TGGGTGAGTG	AACGCACTTGCAGC	TRUE	8.1256	2.1943	-0.611217995	0.262766025	0.020013634
chr3	intron	196069569	196071395	-	ENSG00000072274	196071398	196071389	196069581	196069568	ACTGTAAGTA	GTCTTTGTTCTAGG	TRUE	7.0901	8.9353	-2.637146465	1.048761344	0.011918968
chr11	intron	105966062	105971913	+	ENSG00000152578	105966059	105966068	105971901	105971914	AAGGTTAGCC	TTTCCACGTGAAGA	TRUE	6.314	3.7596	-1.217919229	0.449209694	0.006703032
chr4	intron	67662256	67663138	-	ENSG00000033178	67663141	67663132	67662268	67662255	TGGGTAATGT	TTTTGAGTTGTAGA	TRUE	4.2258	6.7532	-1.123347076	0.534029173	0.035419353
chr16	intron	53470186	53470382	+	ENSG00000103479	53470183	53470192	53470370	53470383	AAGGTAAGGA	TCTCTGTCACTAGG	TRUE	11.3945	7.281	-2.010562109	0.704590859	0.004323819
chr2	intron	192057776	192179667	-	ENSG00000144339	192179670	192179661	192057788	192057775	GAGGTAGGAG	TTCTGTCCTACAGT	TRUE	7.9365	9.0118	-1.3315	0.5574	0.016898
chr6	intron	136651092	136656306	-	ENSG00000197442	136656309	136656300	136651104	136651091	CCAGTGAGTA	GTATCTTTTCAAGG	TRUE	6.5237	6.6863	-0.581861055	0.28856664	0.043759874
chr11	intron	121550089	121550584	+	ENSG00000137642	121550086	121550095	121550572	121550585	AGGGTATGTA	GGTGGGGTGACAGC	TRUE	7.887	-2.3332	-0.767677167	0.260117431	0.003164686
chr1	intron	236886368	236889180	+	ENSG00000116984	236886365	236886374	236889168	236889181	CAGGTCTGTT	TTCTCTCCTGTAGT	TRUE	5.8478	9.0139	-0.753952784	0.250177767	0.002581099
chr14	intron	65077429	65077562	-	ENSG00000125952	65077565	65077556	65077441	65077428	TGTGTAAGTA	CCTACAACCACAGG	TRUE	5.9618	4.7334	-2.377813714	1.00793012	0.018319036
chr1	intron	160084881	160087485	+	ENSG00000162728	160084878	160084887	160087473	160087486	CGGGTGCGAG	CCCCTCCTCCCAGG	TRUE	4.9762	9.7305	-1.162885179	0.563178943	0.038936332
chr2	intron	178601915	178602001	-	ENSG00000155657	178602004	178601995	178601927	178601914	AAGGTAGGAA	CCTTTTCTAATAGG	TRUE	8.8003	9.3345	-1.021409441	0.428590384	0.017163646
chr1	intron	200973579	200974713	-	ENSG00000116852	200974716	200974707	200973591	200973578	CAGGTGGGTG	TCCTCCACCCCAGT	TRUE	9.335	6.7354	-1.1177	0.4767	0.019037
chr3	intron	197068538	197069218	-	ENSG00000075711	197069221	197069212	197068550	197068537	ACCGTAAGTG	TCTAATATGAAAGT	TRUE	6.4119	-0.6342	-1.01534812	0.505577771	0.044612203
chr12	intron	52056160	52056493	+	ENSG00000123358	52056157	52056166	52056481	52056494	AAGGTGTGTG	CATTCCTTTGCAGT	TRUE	9.3377	8.5759	-1.035572619	0.465658783	0.026156033
chr11	intron	121577039	121577280	+	ENSG00000137642	121577036	121577045	121577268	121577281	GAGGCAAGCA	GTCTCACCTGCAGA	FALSE	4.2168	7.1008	-0.608297317	0.248783512	0.014481723
chr17	intron	17815330	17815859	-	ENSG00000072310	17815862	17815853	17815342	17815329	CAGGTGCTCT	CTCCTCTCCACAGT	TRUE	3.6498	8.3967	-1.451707677	0.683615666	0.033705877
chr17	intron	17819726	17820089	-	ENSG00000072310	17820092	17820083	17819738	17819725	CAGGTAAGGG	TTTATTTCTGCAGG	TRUE	11.4519	11.9186	-1.233319317	0.529603728	0.01987185
chr1	intron	26030596	26031131	+	ENSG00000158008	26030593	26030602	26031119	26031132	GAGGTGAGGG	TCTCTCATTTTAGG	TRUE	9.6229	10.0874	-1.057067153	0.407722744	0.009524998
chr1	intron	154344786	154344970	+	ENSG00000143515	154344783	154344792	154344958	154344971	CTGGTAGGCA	CTCTGTGCTCCAGG	TRUE	6.7371	8.5728	-1.132947362	0.4123946	0.006009895
chr3	intron	123949133	123955842	-	ENSG00000175455	123955845	123955836	123949145	123949132	CATGTAAGCC	TCTTCCACATTAGC	TRUE	7.437	5.4632	-0.97812962	0.35234502	0.005502268
chr4	intron	67663216	67663884	-	ENSG00000033178	67663887	67663878	67663228	67663215	GAGGTAAATA	ATGTTCTCAATAGG	TRUE	8.9097	5.8609	-1.100158625	0.444883065	0.013401549
chr2	intron	47480872	47482778	+	ENSG00000095002	47480869	47480878	47482766	47482779	GAGGTTTGTC	ACATGTGTTTCAGC	TRUE	4.6683	3.8287	-1.133795944	0.431224216	0.008557362
chr9	intron	129010694	129012235	-	ENSG00000148341	129012238	129012229	129010706	129010693	GCGGTAAGCG	GCCGTCTCCACAGC	TRUE	7.7243	5.2506	-0.584142756	0.259495321	0.024380952
chr15	intron	83122879	83124671	+	ENSG00000136404	83122876	83122885	83124659	83124672	AAGGTGATTT	AACTCTATTTTAGG	TRUE	8.2666	6.0366	-1.664974254	0.629148152	0.008135486
chr12	intron	123152626	123160780	-	ENSG00000051825	123160783	123160774	123152638	123152625	CAGGTGAAAT	TTTATTTCCATAGA	TRUE	7.1561	9.3568	-0.5635	0.2511	0.02482
chr2	intron	200856986	200857717	-	ENSG00000013441	200857720	200857711	200856998	200856985	ATTGTAAGTT	TCTTTATTCTCAGT	TRUE	7.2852	9.7325	-1.101340728	0.491286374	0.0249776
chr16	intron	20800039	20800301	-	ENSG00000196678	20800304	20800295	20800051	20800038	AAGGTAATGG	TTTTATTGGTTAGC	TRUE	8.2102	4.6286	-0.778886238	0.282242177	0.005786544
chr11	intron	103154607	103154694	+	ENSG00000187240	103154604	103154613	103154682	103154695	TCGGTTTGAT	TGGTATTTTATAGG	TRUE	0.6231	7.2665	-0.638119665	0.269791502	0.018018748
chr5	intron	81092962	81094295	+	ENSG00000113319	81092959	81092968	81094283	81094296	AAGGTACTGG	GGTTTGTTTCCAGA	TRUE	5.1096	8.2004	-0.976515529	0.424408535	0.021398093
chrX	intron	14918783	14921379	+	ENSG00000130150	14918780	14918789	14921367	14921380	CAAGTAAGTA	TTTTCTGTTTCAGT	TRUE	9.9406	10.586	-0.4728	0.2146	0.027564
chr13	intron	24884246	24884322	-	ENSG00000151849	24884325	24884316	24884258	24884245	GTGGTATGTT	TGTGTCTTTACAGA	TRUE	7.1375	9.442	-1.239221487	0.434034385	0.004302019
chr11	intron	134368782	134369804	+	ENSG00000149328	134368779	134368788	134369792	134369805	ATGGCAAGTA	CGGTGTCTTGCAGA	FALSE	4.7221	6.1312	-1.034757095	0.37986343	0.006449182
chr13	intron	111205374	111209871	+	ENSG00000102606	111205371	111205380	111209859	111209872	CAGGTAAGAC	ATGCTCTTTGCAGA	TRUE	10.7669	8.1369	-1.296617012	0.449533191	0.003922061
chr3	intron	4795188	4799354	+	ENSG00000150995	4795185	4795194	4799342	4799355	GAGGTACCCA	CATTCCCAATCAGC	TRUE	3.6344	3.8198	-1.006984065	0.421831804	0.016978656
chr7	intron	95412462	95416241	-	ENSG00000105854	95416244	95416235	95412474	95412461	GTGGTGAGTG	CTAAAATTCCCAGG	TRUE	9.002	4.3033	-0.6469	0.3	0.031056
chr11	intron	123606799	123608658	+	ENSG00000023171	123606796	123606805	123608646	123608659	AAGGTGGGCA	CTCTTCTGTGCAGG	TRUE	7.6691	9.5932	-1.0907	0.5065	0.031291
chr11	intron	105910546	105918711	+	ENSG00000152578	105910543	105910552	105918699	105918712	ATGGTAAGTG	TCTCTCATTATAGG	TRUE	10.7387	9.8159	-0.5909	0.2747	0.031492
chr5	intron	58458529	58458724	-	ENSG00000145632	58458727	58458718	58458541	58458528	AGGGTAAGTG	CTCCTCTTCTTAGT	TRUE	10.2774	8.1895	-1.244612787	0.564882739	0.027572782
chr4	intron	41613105	41613465	+	ENSG00000064042	41613102	41613111	41613453	41613466	AAGGTTTTGG	TCTTTTTTGACAGG	TRUE	1.6477	11.3472	-0.945814329	0.471845206	0.045016388
chr2	intron	209704035	209704449	+	ENSG00000078018	209704032	209704041	209704437	209704450	GAGGTTTGAG	CTTTCATGGCTAGA	TRUE	3.8943	4.0203	-0.861315621	0.325320225	0.008106701
chr4	intron	55871234	55871848	+	ENSG00000090989	55871231	55871240	55871836	55871849	CAGGTATGTT	CGTTCTGTGTCAGG	TRUE	9.9908	6.464	-1.333128518	0.632309159	0.035000794
chr1	intron	65622982	65636190	+	ENSG00000116678	65622979	65622988	65636178	65636191	AAGGTTGCTT	TCTTTTCTTTTAGC	TRUE	2.133	11.3575	-0.9624	0.4576	0.035458
chr15	intron	47761432	47761660	+	ENSG00000137872	47761429	47761438	47761648	47761661	AGGGTAAGTA	TTTTCTTTTGTAGT	TRUE	10.3123	11.2377	-1.032102208	0.491104969	0.035589043
chr3	intron	196058366	196058573	-	ENSG00000072274	196058576	196058567	196058378	196058365	AGTGTAAGTT	TTTGTCTTTGCAGT	TRUE	6.8239	10.3107	-2.305214344	1.097491365	0.035690187
chr12	intron	21470347	21470968	-	ENSG00000004700	21470971	21470962	21470359	21470346	AGGGTAAATG	TTTTTTTTTGTAGG	TRUE	7.3103	13.9145	-0.788219319	0.305898943	0.009973951
chr15	intron	74840317	74840497	-	ENSG00000140474	74840500	74840491	74840329	74840316	ATGGTGAGAC	CTCCCTTCCACAGA	TRUE	7.8347	9.3148	-0.533031947	0.23850114	0.025422332
chr12	intron	914122	914430	-	ENSG00000002016	914433	914424	914134	914121	TCAGTAAATA	TTGACCTCTGTAGA	TRUE	3.1281	6.3361	-0.505739872	0.239169446	0.03446685
chr1	intron	204537498	204549265	+	ENSG00000198625	204537495	204537504	204549253	204549266	AAGGTAAAAT	CATACCTGAAAAGG	TRUE	7.9716	-0.4939	-1.637	0.7846	0.03694
chr4	intron	122247358	122249484	+	ENSG00000138688	122247355	122247364	122249472	122249485	CAGGTAGGAT	CTTTTGATAATAGA	TRUE	8.5107	5.8261	-0.493301362	0.23652447	0.0370126
chr7	intron	64522501	64543706	-	ENSG00000173041	64543709	64543700	64522513	64522500	CAGGTAGGTG	TTATGTCTTTCAGT	TRUE	10.2428	8.3773	-0.7234	0.3495	0.038458
chr19	intron	9341185	9341616	+	ENSG00000188321	9341182	9341191	9341604	9341617	ATGGTAAGAT	CCATGTGTAACAGA	TRUE	9.0066	3.1947	-0.501249086	0.239179633	0.03610869
chr4	intron	157143960	157152717	+	ENSG00000109738	157143957	157143966	157152705	157152718	TGGGTAAGGT	TCTGTTTCTGTAGG	TRUE	7.3752	10.1875	-1.116410727	0.539548095	0.038531181
chr2	intron	43800918	43801638	+	ENSG00000138036	43800915	43800924	43801626	43801639	AAGGTACTGC	CTTCTCCACGTAGC	TRUE	4.4985	6.0614	-1.459207321	0.529596604	0.005863503
chr16	intron	89737906	89737975	+	ENSG00000158805	89737903	89737912	89737963	89737976	GCAGTAAGTG	CTCTGTCCCCCAGG	TRUE	6.4754	9.4098	-1.5901	0.7687	0.038583
chr2	intron	121418730	121425138	-	ENSG00000074054	121425141	121425132	121418742	121418729	TAGGTAAGGA	TGCTGCACGCTAGC	TRUE	10.1505	1.221	-0.812527956	0.410233147	0.047630627
chr16	intron	76553908	76558489	+	ENSG00000152910	76553905	76553914	76558477	76558490	GAGGTAAATA	TTTCTCTCTCTAGG	TRUE	7.3423	12.3109	-0.586614659	0.263415996	0.025950512
chr1	intron	53218924	53220260	-	ENSG00000162384	53220263	53220254	53218936	53218923	AAGGTGCGGC	TTTTTCCCCAAAGA	TRUE	6.7401	8.539	-1.022212639	0.502575109	0.041956638
chr10	intron	73848083	73848525	-	ENSG00000148660	73848528	73848519	73848095	73848082	GCGGTAAGCC	TTCTGTCTCGTAGG	TRUE	7.1132	9.343	-1.256531645	0.554155249	0.023361393
chr9	intron	131510063	131510259	+	ENSG00000130714	131510060	131510069	131510247	131510260	GGGGTACTTG	GAATCTCTGGCAGG	TRUE	3.1985	3.7216	-0.476529834	0.235186332	0.042746346
chr17	intron	61984866	61984999	-	ENSG00000108510	61985002	61984993	61984878	61984865	TAAGTAAGCT	TTATTAAATATAGG	TRUE	6.6139	5.0044	-1.291615678	0.611881744	0.03478168
chr11	intron	9145114	9145669	-	ENSG00000184014	9145672	9145663	9145126	9145113	GAGGTATGTG	TATTCTCTAATAGT	TRUE	9.4166	6.073	-0.522497069	0.259255238	0.043865152
chr1	intron	36287271	36289059	+	ENSG00000054118	36287268	36287277	36289047	36289060	GAGGCAAGTA	TTTTTATAAATAGG	FALSE	5.8253	7.0682	-0.867831234	0.418835158	0.038264375
chr1	intron	156414754	156416768	-	ENSG00000125462	156416771	156416762	156414766	156414753	AAGGTGAGAC	CTCTTCCCTTTAGC	TRUE	9.7668	9.1561	-0.998514025	0.37053021	0.007042564
chr17	intron	8149311	8149461	-	ENSG00000179094	8149464	8149455	8149323	8149310	CAGGTGAGGT	CTGTCTGTCCCAGG	TRUE	10.1971	8.3155	-1.356378393	0.676411043	0.044935514
chr14	intron	74908933	74909100	-	ENSG00000198208	74909103	74909094	74908945	74908932	CAGGCAAGAA	CTTGTTCCCACAGA	FALSE	5.3614	9.3333	-1.525694492	0.728442481	0.036218918
chr4	intron	185399029	185399569	+	ENSG00000186352	185399026	185399035	185399557	185399570	TGAGTGAGTA	ATCCTGTTTTCAGT	TRUE	5.3031	7.2309	-0.879887468	0.43033199	0.040887275
chr3	intron	36990436	36992877	-	ENSG00000178567	36992880	36992871	36990448	36990435	GCGGTATGTG	TTTTTACTGTGAGA	TRUE	6.9075	10.5064	-0.5007	0.252	0.046974
chr12	intron	78177314	78177619	+	ENSG00000067798	78177311	78177320	78177607	78177620	AGCGTAAGTT	TATGTACATACAGG	TRUE	6.1806	5.6139	-1.288806014	0.608974277	0.03431459
chr10	intron	27028946	27029285	-	ENSG00000107890	27029288	27029279	27028958	27028945	AAAGTAAAAT	ATATACATTTCAGG	TRUE	5.1142	5.8943	-1.393605695	0.554849956	0.012015789
chr17	intron	76089321	76090328	-	ENSG00000182473	76090331	76090322	76089333	76089320	CCGGTGAGTA	TGCCATCCCCCAGG	TRUE	9.3811	6.9441	-1.154544986	0.54557857	0.034329107
chr1	intron	206458410	206458622	+	ENSG00000266028	206458407	206458416	206458610	206458623	CAAGTAAGTA	TTTGTGATTGAAGG	TRUE	9.9406	5.3525	-1.709354788	0.83352996	0.040292091
chr2	intron	50620184	50621204	-	ENSG00000179915	50621207	50621198	50620196	50620183	TCGGTAGATA	CTTTGTTATCTAGG	TRUE	3.4652	8.9335	-0.418115671	0.21263114	0.049253619

ReadMe

chrom	chromosome number
feature	GTF feature (all introns)
intron_start	nucleotide position of intron start
intron_end	nucleotide position of intron end
strand	"+" or "-"
geneid	Ensembl Gene ID
donor_start	nucleotide position of the 5'-donor splice site start
donor_end	nucleotide position of the 5'-donor splice site end
acceptor_start	nucleotide position of the 3'-acceptor splice site start
acceptor_end	nucleotide position of the 3'-acceptor splice site end
donorSEQ	nucleotide sequence of the 5'-donor splice site
acceptorSEQ	nucleotide sequence of the 3'-acceptor splice site
canonical	canonical splice site (TRUE or FALSE)
donor_junc-score	the splice junction score at the donor site
acceptor_junc-score	the splice junction score at the acceptor site
beta	association coefficient with the AUD phenotype (>0, positively associated; <0, negatively associated)
se	standard error
p-value	statistical significance of the association with AUD

Supplementary Table 4

Tissue_cell_type_name	Developmental_stag	Organ_system	Tissue	Specific_cell_type	General_cell_type	Cell_Ontology_ID	General_tissue_name	Uber_anatomy_ontology_I	input_list_raw_p	input_list_permutated_p	input_list_rank_p	input_list_combined_p	Input_List_Combined_Fdr	input_list_combined_log10p	delabel
TS_Prostate_club cell of prostate epithelium	Adult	Reproductive system	TS_Prostate	club cell of prostate epithelium	Club cell	CL:0000158	Prostate gland	UBERON:0002367	4.59803E-36	0.000535222	0.000738007	6.21899E-06	0.006586544	5.206279963	TS_Prostate_club cell of prostate epithelium
Lake_2017_VisualCortex_Ex1	Adult	Nervous system	Lake_2017_VisualCortex	Ex1	Excitatory neuron	CL:0008030	Visual cortex	UBERON:0000411	1.68805E-32	0.000370538	0.002214022	1.23168E-05	0.006586544	4.909503374	Lake_2017_VisualCortex_Ex1
Fetal_HCA_Cerebellum_10k_Purkinje neurons	Fetal	Nervous system	Fetal_HCA_Cerebellum_10k	Purkinje neurons	Neuron	CL:0000540	Cerebellum	UBERON:0002037	7.58408E-30	0.000329367	0.004428044	2.10573E-05	0.006586544	4.676596726	Fetal_HCA_Cerebellum_10k_Purkinje neurons
Lake_2017_VisualCortex_Ex4	Adult	Nervous system	Lake_2017_VisualCortex	Ex4	Excitatory neuron	CL:0008030	Visual cortex	UBERON:0000411	2.67949E-28	0.000247025	0.005904059	2.10573E-05	0.006586544	4.676596726	Lake_2017_VisualCortex_Ex4
TS_Prostate_luminal cell of prostate epithelium	Adult	Reproductive system	TS_Prostate	luminal cell of prostate epithelium	Luminal cell of prostate epithelium	CL:0002340	Prostate gland	UBERON:0002367	8.7835E-32	0.000576392	0.00295203	2.43046E-05	0.006586544	4.61431167	TS_Prostate_luminal cell of prostate epithelium
Lake_2017_FrontalCortex_Ex5b	Adult	Nervous system	Lake_2017_FrontalCortex	Ex5b	Excitatory neuron	CL:0008030	Frontal cortex	UBERON:0001870	2.17562E-28	0.000494051	0.005166052	3.5422E-05	0.007450071	4.450726645	NA
Lake_2017_VisualCortex_Ex5b	Adult	Nervous system	Lake_2017_VisualCortex	Ex5b	Excitatory neuron	CL:0008030	Visual cortex	UBERON:0000411	2.55E-24	0.000329367	0.008856089	4.00928E-05	0.007450071	4.39693347	NA
Voigt_2019_retina_2_Vein cell 2	Adult	Sensory system	Voigt_2019_retina_2	Vein cell 2	Endothelial cell	CL:0000115	Retina	UBERON:0000966	3.01387E-10	4.11709E-05	0.089298893	4.96827E-05	0.007450071	4.303794385	NA
Lake_2017_FrontalCortex_Ex4	Adult	Nervous system	Lake_2017_FrontalCortex	Ex4	Excitatory neuron	CL:0008030	Frontal cortex	UBERON:0001870	2.29274E-21	0.000288196	0.01402214	5.42278E-05	0.007450071	4.265777693	NA
Lake_2017_VisualCortex_Ex3a	Adult	Nervous system	Lake_2017_VisualCortex	Ex3a	Excitatory neuron	CL:0008030	Visual cortex	UBERON:0000411	1.49494E-23	0.000370538	0.011070111	5.49821E-05	0.007450071	4.259778891	NA
Lake_2017_FrontalCortex_Ex1	Adult	Nervous system	Lake_2017_FrontalCortex	Ex1	Excitatory neuron	CL:0008030	Frontal cortex	UBERON:0001870	2.15204E-24	0.000576392	0.008118081	6.21041E-05	0.007650101	4.206879423	NA
Fetal_HCA_Cerebellum_10k_SLC24A4_PEX5L positive cells	Fetal	Nervous system	Fetal_HCA_Cerebellum_10k	SLC24A4_PEX5L positive cells	Neuron	CL:0000540	Cerebellum	UBERON:0002037	4.7496E-19	0.000411709	0.022878229	0.000118425	0.013074545	3.926556615	NA
Lake_2017_VisualCortex_Ex6b	Adult	Nervous system	Lake_2017_VisualCortex	Ex6b	Excitatory neuron	CL:0008030	Visual cortex	UBERON:0000411	3.81423E-19	0.00045288	0.022140221	0.000125438	0.013074545	3.901569365	NA
TS_Pancreas_fibroblast	Adult	Digestive system	TS_Pancreas	fibroblast	Fibroblast	CL:0000057	Pancreas	UBERON:0001264	9.42129E-28	0.001688006	0.006642066	0.000139011	0.013454237	3.856952171	NA
Fetal_HCA_Cerebrum_10k_SKOR2_NPSR1 positive cells	Fetal	Nervous system	Fetal_HCA_Cerebrum_10k	SKOR2_NPSR1 positive cells	Neuron	CL:0000540	Cerebral hemisphere	UBERON:0001869	1.20388E-17	0.000411709	0.031734317	0.000159992	0.014126863	3.795902283	NA
Lake_2017_VisualCortex_Ex3b	Adult	Nervous system	Lake_2017_VisualCortex	Ex3b	Excitatory neuron	CL:0008030	Visual cortex	UBERON:0000411	7.43891E-20	0.000741076	0.018450185	0.000166812	0.014126863	3.777773571	NA
TS_Salivary_Gland_duct epithelial cell	Adult	Digestive system	TS_Salivary_Gland	duct epithelial cell	Epithelial cell	CL:0000066	Saliva-secreting gland	UBERON:0001044	2.7445E-34	0.011280823	0.001476015	0.000199859	0.01592993	3.699276501	NA
Lake_2017_CerebellarHemisphere_Purk2	Adult	Nervous system	Lake_2017_CerebellarHemisphere	Purk2	Purkinje cell	CL:0000121	Cerebellar hemisphere	UBERON:0002245	8.59786E-18	0.000617563	0.03099631	0.000227096	0.017095246	3.643791425	NA
TS_Prostate_hillock cell of prostate epithelium	Adult	Reproductive system	TS_Prostate	hillock cell of prostate epithelium	Epithelial cell	CL:0000066	Prostate gland	UBERON:0002367	6.52309E-21	0.001729177	0.014760148	0.000295452	0.02107036	3.529513731	NA
Lake_2017_FrontalCortex_Ex2	Adult	Nervous system	Lake_2017_FrontalCortex	Ex2	Excitatory neuron	CL:0008030	Frontal cortex	UBERON:0001870	1.67581E-14	0.000741076	0.050922509	0.000422088	0.028596461	3.374597002	NA
Lake_2017_VisualCortex_Ex8	Adult	Nervous system	Lake_2017_VisualCortex	Ex8	Excitatory neuron	CL:0008030	Visual cortex	UBERON:0000411	1.86936E-11	0.000658734	0.074538745	0.000536265	0.034601874	3.270620376	NA
Lake_2017_CerebellarHemisphere_Oli	Adult	Nervous system	Lake_2017_CerebellarHemisphere	Oli	Oligodendrocyte	CL:0000128	Cerebellar hemisphere	UBERON:0002245	2.77231E-07	0.000370538	0.143173432	0.000575301	0.035433287	3.240105172	NA
TS_Tongue_basal cell	Adult	Digestive system	TS_Tongue	basal cell	Basal cell	CL:0000646	Tongue	UBERON:0001723	4.44046E-30	0.016344843	0.003690037	0.000646313	0.038076241	3.189557395	NA
Lake_2017_FrontalCortex_Ex8	Adult	Nervous system	Lake_2017_FrontalCortex	Ex8	Excitatory neuron	CL:0008030	Frontal cortex	UBERON:0001870	2.3503E-10	0.000823418	0.087084871	0.000756004	0.038967863	3.121476186	NA
AdultCerebellum_Oligodendrocyte	Adult	Nervous system	AdultCerebellum	Oligodendrocyte	Oligodendrocyte	CL:0000128	Cerebellum	UBERON:0002037	4.67602E-06	0.00045288	0.171217712	0.000811444	0.038967863	3.090741471	NA
Fetal_HCA_Eye_Lens fibre cells	Fetal	Sensory system	Fetal_HCA_Eye	Lens fibre cells	Lens fibre cell	CL:0011004	Eye	UBERON:0000970	6.11997E-06	0.00045288	0.172693727	0.000817768	0.038967863	3.087369978	NA
Fetal_HCA_Cerebrum_10k_Limbic system neurons	Fetal	Nervous system	Fetal_HCA_Cerebrum_10k	Limbic system neurons	Neuron	CL:0000540	Cerebral hemisphere	UBERON:0001869	1.20346E-15	0.001770348	0.044280443	0.000819492	0.038967863	3.086455531	NA
Lake_2017_VisualCortex_Ex5a	Adult	Nervous system	Lake_2017_VisualCortex	Ex5a	Excitatory neuron	CL:0008030	Visual cortex	UBERON:0000411	4.46642E-17	0.002346741	0.033948339	0.000831546	0.038967863	3.080113923	NA
FetalPancreas_Fetal endocrine cell	Fetal	Digestive system	FetalPancreas	Fetal endocrine cell	Endocrine cell	CL:0000163	Pancreas	UBERON:0001264	1.59806E-06	0.000535222	0.157195572	0.000873576	0.038967863	3.058699302	NA
Fetal_HCA_Pancreas_Islet endocrine cells	Fetal	Digestive system	Fetal_HCA_Pancreas	Islet endocrine cells	Endocrine cell	CL:0000163	Pancreas	UBERON:0001264	8.14525E-10	0.00090576	0.094464945	0.000886964	0.038967863	3.052094178	NA
Lake_2017_CerebellarHemisphere_Purk1	Adult	Nervous system	Lake_2017_CerebellarHemisphere	Purk1	Purkinje cell	CL:0000121	Cerebellar hemisphere	UBERON:0002245	8.83183E-16	0.001976203	0.043542435	0.000891516	0.038967863	3.04987101	NA
FetalPancreas_Fetal neuron	Fetal	Digestive system	FetalPancreas	Fetal neuron	Neuron	CL:0000540	Pancreas	UBERON:0001264	6.46797E-08	0.000864589	0.132103321	0.001150994	0.048737391	2.938927044	NA
van_Zyl_eye_2020_CiliaryMuscle	Adult	Sensory system	van_Zyl_eye_2020	CiliaryMuscle	Muscle cell	CL:0000187	Eye	UBERON:0000970	9.33302E-06	0.000699905	0.185239852	0.001290107	0.050208723	2.889374109	NA
FetalPancreas_Fetal acinar cell	Fetal	Digestive system	FetalPancreas	Fetal acinar cell	Acinar cell	CL:0000622	Pancreas	UBERON:0001264	1.15038E-05	0.000699905	0.18597786	0.00129473	0.050208723	2.887820863	NA
Enge_2017_Pancreas_pancreatic A cell	Adult	Digestive system	Enge_2017_Pancreas	pancreatic A cell	Pancreatic A cell	CL:0000171	Pancreas	UBERON:0001264	5.75828E-14	0.002387912	0.054612546	0.001296904	0.050208723	2.887092078	NA
Fetal_HCA_Intestine_ENS neurons	Fetal	Digestive system	Fetal_HCA_Intestine	ENS neurons	Enteric neuron	CL:0007011	Intestine	UBERON:0000160	6.59173E-11	0.001646836	0.084132841	0.001369493	0.0515462	2.863440144	NA
AdultTemporalLobe_Oligodendrocyte	Adult	Nervous system	AdultTemporalLobe	Oligodendrocyte	Oligodendrocyte	CL:0000128	Temporal lobe	UBERON:0001871	3.93746E-06	0.000864589	0.170479705	0.00144777	0.053019694	2.839300356	NA
Fetal_HCA_Lung_Visceral neurons	Fetal	Respiratory system	Fetal_HCA_Lung	Visceral neurons	Neuron	CL:0000540	Lung	UBERON:0002048	5.5133E-12	0.002470253	0.070110701	0.00167322	0.059663499	2.776446977	NA
Lake_2017_FrontalCortex_Ex6b	Adult	Nervous system	Lake_2017_FrontalCortex	Ex6b	Excitatory neuron	CL:0008030	Frontal cortex	UBERON:0001870	9.2185E-09	0.001729177	0.114391144	0.001884711	0.065481611	2.72475533	NA
Lake_2017_CerebellarHemisphere_Ast	Adult	Nervous system	Lake_2017_CerebellarHemisphere	Ast	Astrocyte	CL:0000127	Cerebellar hemisphere	UBERON:0002245	2.82778E-09	0.002182057	0.101107011	0.002078046	0.069639754	2.682344918	NA
Braga_2020_lung_Transformed epithelium	Adult	Respiratory system	Braga_2020_lung	Transformed epithelium	Epithelial cell	CL:0000066	Lung	UBERON:0002048	0.000194011	0.001029272	0.217712177	0.002107181	0.069639754	2.676298211	NA
Lake_2017_FrontalCortex_Oli	Adult	Nervous system	Lake_2017_FrontalCortex	Oli	Oligodendrocyte	CL:0000128	Frontal cortex	UBERON:0001870	7.99708E-07	0.001811519	0.150553506	0.002511037	0.081010847	2.600146833	NA
Lake_2017_VisualCortex_Ex3d	Adult	Nervous system	Lake_2017_VisualCortex	Ex3d	Excitatory neuron	CL:0008030	Visual cortex	UBERON:0000411	9.59622E-09	0.002511424	0.115129151	0.002645211	0.081953077	2.5775397	NA
FetalIntestine_Fetal neuron	Fetal	Digestive system	FetalIntestine	Fetal neuron	Neuron	CL:0000540	Intestine	UBERON:0000160	5.77807E-06	0.001729177	0.17195572	0.002711945	0.081953077	2.566719113	NA
Fetal_HCA_Adrenal_Adrenocortical cells	Fetal	Endocrine system	Fetal_HCA_Adrenal	Adrenocortical cells	Cortical cell of adrenal gland	CL:0002097	Adrenal gland	UBERON:0002369	2.54879E-06	0.001770348	0.168634686	0.002721689	0.081953077	2.565161519	NA
Fetal_HCA_Heart_SATB2_LRRC7 positive cells	Fetal	Cardiovascular system	Fetal_HCA_Heart	SATB2_LRRC7 positive cells	Neuron	CL:0000540	Heart	UBERON:0000948	2.56894E-10	0.003664209	0.087822878	0.002909592	0.085353359	2.536167952	NA
Fetal_HCA_Pancreas_ENS neurons	Fetal	Digestive system	Fetal_HCA_Pancreas	ENS neurons	Neuron	CL:0000540	Pancreas	UBERON:0001264	9.20165E-08	0.002470253	0.132841328	0.002960596	0.085353359	2.528620821	NA
Enge_2017_Pancreas_type B pancreatic cell	Adult	Digestive system	Enge_2017_Pancreas	type B pancreatic cell	Type B pancreatic cell	CL:0000169	Pancreas	UBERON:0001264	5.85023E-09	0.002964304	0.113653137	0.003030679	0.085553545	2.518460049	NA
TS_Salivary_Gland_basal cell	Adult	Digestive system	TS_Salivary_Gland	basal cell	Basal cell	CL:0000646	Saliva-secreting gland	UBERON:0001044	2.46857E-25	0.048828673	0.007380074	0.003217435	0.088971914	2.492490282	NA
TS_Uterus_endothelial cell	Adult	Reproductive system	TS_Uterus	endothelial cell	Endothelial cell	CL:0000115	Uterus	UBERON:0000995	7.56099E-24	0.03894766	0.009594096	0.003322706	0.090045333	2.478508085	NA
TS_Large_Intestine_large intestine goblet cell	Adult	Digestive system	TS_Large_Intestine	large intestine goblet cell	Goblet cell	CL:0000160	Large intestine	UBERON:0000059	3.87132E-16	0.009798671	0.039483395	0.003426787	0.091045032	2.465112868	NA
Voigt_2019_retina_2_Schwann cell	Adult	Sensory system	Voigt_2019_retina_2	Schwann cell	Schwann cell	CL:0002573	Retina	UBERON:0000966	0.000117497	0.002264399	0.21402214	0.004183398	0.109009699	2.378470813	NA
Fetal_HCA_Eye_Amacrine cells	Fetal	Sensory system	Fetal_HCA_Eye	Amacrine cells	Neuron	CL:0000540	Eye	UBERON:0000970	2.7053E-07	0.003581867	0.142066421	0.00436774	0.111665816	2.35974318	NA
Lake_2017_FrontalCortex_Ast	Adult	Nervous system	Lake_2017_FrontalCortex	Ast	Astrocyte	CL:0000127	Frontal cortex	UBERON:0001870	7.67189E-07	0.003581867	0.149815498	0.004577481	0.114231013	2.339373445	NA
TS_Small_Intestine_small intestine goblet cell	Adult	Digestive system	TS_Small_Intestine	small intestine goblet cell	Goblet cell	CL:0000160	Small intestine	UBERON:0002108	8.53726E-19	0.023055697	0.023616236	0.004636683	0.114231013	2.333792577	NA
Fetal_HCA_Eye_Ganglion cells	Fetal	Sensory system	Fetal_HCA_Eye	Ganglion cells	Retinal ganglion cell	CL:0000740	Eye	UBERON:0000970	2.7053E-07	0.004075918	0.142066421	0.004895367	0.118450402	2.310214728	NA
Lake_2017_VisualCortex_Oli	Adult	Nervous system	Lake_2017_VisualCortex	Oli	Oligodendrocyte	CL:0000128	Visual cortex	UBERON:0000411	6.26279E-06	0.003417184	0.176383764	0.005071438	0.119841143	2.29486892	NA
TS_Large_Intestine_monocyte	Adult	Digestive system	TS_Large_Intestine	monocyte	Monocyte	CL:0000576	Large intestine	UBERON:0000059	2.43548E-22	0.051710636	0.011808118	0.005129732	0.119841143	2.289905359	NA
Segerstolpe_2016_PancreaticIslets_pancreatic PP cell	Adult	Digestive system	Segerstolpe_2016_PancreaticIslets	pancreatic PP cell	Pancreatic PP cell	CL:0002275	Pancreas	UBERON:0001264	1.31303E-06	0.004281772	0.155719557	0.005542798	0.126688904	2.256270946	NA
Fetal_HCA_Heart_Visceral neurons	Fetal	Cardiovascular system	Fetal_HCA_Heart	Visceral neurons	Neuron	CL:0000540	Heart	UBERON:0000948	2.46358E-06	0.004034747	0.167527675	0.005609841	0.126688904	2.251049467	NA
Fetal_HCA_Spleen_Vascular endothelial cells	Fetal	Lymphatic system	Fetal_HCA_Spleen	Vascular endothelial cells	Endothelial cell	CL:0000115	Spleen	UBERON:0002106	0.000124672	0.0032525	0.214760148	0.005774257	0.128264229	2.238503906	NA
Fetal_HCA_Adrenal_Chromaffin cells	Fetal	Endocrine system	Fetal_HCA_Adrenal	Chromaffin cells	Neuron	CL:0000540	Adrenal gland	UBERON:0002369	1.05074E-07	0.005311045	0.13800738	0.006023797	0.130181795	2.220129681	NA
TS_Vasculature_smooth muscle cell	Adult	Cardiovascular system	TS_Vasculature	smooth muscle cell	Smooth muscle cell	CL:0000192	Vasculature	UBERON:0002049	1.26384E-21	0.059450763	0.012546125	0.006116898	0.130181795	2.213468768	NA
TS_Pancreas_pancreatic alpha cell	Adult	Digestive system	TS_Pancreas	pancreatic alpha cell	Pancreatic A cell	CL:0000171	Pancreas	UBERON:0001264	3.20462E-09	0.007451931	0.101845018	0.006210867	0.130181795	2.206847791	NA
Fetal_HCA_Heart_Cardiomyocytes	Fetal	Cardiovascular system	Fetal_HCA_Heart	Cardiomyocytes	Fetal cardiomyocyte	CL:0002495	Heart	UBERON:0000948	0.000108836	0.003623038	0.213284133	0.006309836	0.130181795	2.199981911	NA
Fetal_HCA_Eye_PDE11A_FAM19A2 positive cells	Fetal	Sensory system	Fetal_HCA_Eye	PDE11A_FAM19A2 positive cells	Neuron	CL:0000540	Eye	UBERON:0000970	4.11663E-05	0.003828893	0.20295203	0.006340958	0.130181795	2.197845104	NA
Fetal_HCA_Stomach_Neuroendocrine cells	Fetal	Digestive system	Fetal_HCA_Stomach	Neuroendocrine cells	Epithelial cell	CL:0000066	Stomach	UBERON:0000945	4.68611E-05	0.003952405	0.204428044	0.006561602	0.132700313	2.18299014	NA
TS_Blood_alpha-beta t cell	Adult	Lymphatic system	TS_Blood	alpha-beta t cell	T cell	CL:0000084	Blood	UBERON:0000178	3.62904E-20	0.049487408	0.016605166	0.006659499	0.132700313	2.176558436	NA
AdultCerebellum_Astrocyte	Adult	Nervous system	AdultCerebellum	Astrocyte	Astrocyte	CL:0000127	Cerebellum	UBERON:0002037	0.000181504	0.003993576	0.21697417	0.006976254	0.136997446	2.156377732	NA
TS_Spleen_type i nk t cell	Adult	Lymphatic system	TS_Spleen	type i nk t cell	Natural killer cell	CL:0000623	Spleen	UBERON:0002106	1.78307E-19	0.045617344	0.019926199	0.007274739	0.140818169	2.138182561	NA
TS_Eye_retinal bipolar neuron	Adult	Sensory system	TS_Eye	retinal bipolar neuron	Bipolar neuron	CL:0000103	Eye	UBERON:0000970	1.99914E-17	0.028366742	0.033210332	0.00750587	0.143245833	2.12459895	NA
Fetal_HCA_Heart_Schwann cells	Fetal	Cardiovascular system	Fetal_HCA_Heart	Schwann cells	Schwann cell	CL:0002573	Heart	UBERON:0000948	0.000256215	0.004734652	0.224354244	0.008335791	0.15687496	2.079053169	NA
Lake_2017_VisualCortex_Ast	Adult	Nervous system	Lake_2017_VisualCortex	Ast	Astrocyte	CL:0000127	Visual cortex	UBERON:0000411	9.45876E-08	0.008110665	0.133579336	0.008480595	0.157413781	2.071573684	NA
TS_Bladder_smooth muscle cell	Adult	Urinary system	TS_Bladder	smooth muscle cell	Smooth muscle cell	CL:0000192	Bladder organ	UBERON:0018707	1.75861E-19	0.066820352	0.019188192	0.009820343	0.179818444	2.007873339	NA
Fetal_HCA_Cerebrum_10k_Inhibitory neurons	Fetal	Nervous system	Fetal_HCA_Cerebrum_10k	Inhibitory neurons	Inhibitory neuron	CL:0008029	Cerebral hemisphere	UBERON:0001869	3.62414E-08	0.010045697	0.131365314	0.010069483	0.180694968	1.996992829	NA
Fetal_HCA_Eye_Bipolar cells	Fetal	Sensory system	Fetal_HCA_Eye	Bipolar cells	Bipolar neuron	CL:0000103	Eye	UBERON:0000970	0.000545138	0.005475728	0.242804428	0.010134921	0.180694968	1.994179644	NA
Fetal_HCA_Stomach_ENS neurons	Fetal	Digestive system	Fetal_HCA_Stomach	ENS neurons	ENS neuron	CL:0000540	Stomach	UBERON:0000945	1.81633E-05	0.007328418	0.193357934	0.010711456	0.18687322	1.970151479	NA
TS_Large_Intestine_intestinal enteroendocrine cell	Adult	Digestive system	TS_Large_Intestine	intestinal enteroendocrine cell	Endocrine cell	CL:0000163	Large intestine	UBERON:0000059	3.87132E-16	0.036065698	0.039483395	0.010757278	0.18687322	1.968297624	NA
TS_Thymus_fast muscle cell	Adult	Lymphatic system	TS_Thymus	fast muscle cell	Cell of skeletal muscle	CL:0000188	Thymus	UBERON:0002370	1.0407E-07	0.010786773	0.135055351	0.010971982	0.188190332	1.959714895	NA
TS_Salivary_Gland_myoepithelial cell	Adult	Digestive system	TS_Salivary_Gland	myoepithelial cell	Epithelial cell	CL:0000066	Saliva-secreting gland	UBERON:0001044	1.22376E-13	0.026349368	0.057195572	0.011299372	0.191383107	1.946945707	NA
TS_Blood_cd4-positive, alpha-beta memory t cell	Adult	Lymphatic system	TS_Blood	cd4-positive, alpha-beta memory t cell	T cell	CL:0000084	Blood	UBERON:0000178	1.81137E-19	0.075425067	0.02103321	0.011812993	0.197612415	1.927640051	NA
TS_Blood_type i nk t cell	Adult	Lymphatic system	TS_Blood	type i nk t cell	T cell	CL:0000084	Blood	UBERON:0000178	3.62904E-20	0.102597854	0.016605166	0.012564417	0.207619322	1.900857673	NA
TS_Blood_classical monocyte	Adult	Lymphatic system	TS_Blood	classical monocyte	Monocyte	CL:0000576	Blood	UBERON:0000178	8.49517E-24	0.170859186	0.010332103	0.012956524	0.210939844	1.887511484	NA
Fetal_HCA_Adrenal_Sympathoblasts	Fetal	Endocrine system	Fetal_HCA_Adrenal	Sympathoblasts	Neuron	CL:0000540	Adrenal gland	UBERON:0002369	2.54879E-06	0.010580918	0.168634686	0.013076714	0.210939844	1.883501389	NA
TS_Salivary_Gland_pericyte cell	Adult	Digestive system	TS_Salivary_Gland	pericyte cell	Pericyte cell	CL:0000669	Saliva-secreting gland	UBERON:0001044	4.09721E-18	0.06719089	0.027306273	0.013395121	0.213281015	1.873053371	NA
TS_Uterus_myometrial cell	Adult	Reproductive system	TS_Uterus	myometrial cell	Myometrial cell	CL:0002366	Uterus	UBERON:0000995	1.04562E-09	0.019432659	0.095571956	0.013536655	0.213281015	1.868488645	NA
FetalStomach_Fetal neuron	Fetal	Digestive system	FetalStomach	Fetal neuron	Neuron	CL:0000540	Stomach	UBERON:0000945	0.000273891	0.008481203	0.226568266	0.013940255	0.217115469	1.855729275	NA
Fetal_HCA_Kidney_Metanephric cells	Fetal	Urinary system	Fetal_HCA_Kidney	Metanephric cells	Epithelial cell	CL:0000066	Kidney	UBERON:0002113	2.60429E-06	0.012845317	0.169741697	0.015542343	0.239316765	1.808483499	NA
Fetal_HCA_Eye_Horizontal cells	Fetal	Sensory system	Fetal_HCA_Eye	Horizontal cells	Retina horizontal cell	CL:0000745	Eye	UBERON:0000970	0.000239318	0.009922184	0.222878229	0.015732423	0.239521724	1.803204379	NA
TS_Blood_naive b cell	Adult	Lymphatic system	TS_Blood	naive b cell	B cell	CL:0000236	Blood	UBERON:0000178	7.42584E-15	0.0468113	0.050184502	0.016570517	0.249478336	1.780663947	NA
TS_Muscle_mesenchymal stem cell	Adult	Muscular system	TS_Muscle	mesenchymal stem cell	Mesenchymal stem cell	CL:0000134	Muscle organ	UBERON:0001630	1.3622E-21	0.181110737	0.013284133	0.016913065	0.251837404	1.771777669	NA
TS_Spleen_endothelial cell	Adult	Lymphatic system	TS_Spleen	endothelial cell	Endothelial cell	CL:0000115	Spleen	UBERON:0002106	8.71133E-19	0.09778086	0.025830258	0.017632539	0.259696641	1.753685136	NA
TS_Spleen_naive b cell	Adult	Lymphatic system	TS_Spleen	naive b cell	B cell	CL:0000236	Spleen	UBERON:0002106	1.12741E-08	0.021203007	0.122509225	0.018061333	0.263151683	1.743250194	NA
TS_Pancreas_pancreatic ductal cell	Adult	Digestive system	TS_Pancreas	pancreatic ductal cell	Pancreatic ductal cell	CL:0002079	Pancreas	UBERON:0001264	3.01623E-20	0.169747572	0.015498155	0.018258828	0.263199063	1.738527102	NA
Fetal_HCA_Pancreas_ENS glia	Fetal	Digestive system	Fetal_HCA_Pancreas	ENS glia	Glial cell	CL:0000125	Pancreas	UBERON:0001264	0.000241733	0.01206307	0.223616236	0.018654362	0.266070116	1.72921959	NA
TS_Salivary_Gland_fibroblast	Adult	Digestive system	TS_Salivary_Gland	fibroblast	Fibroblast	CL:0000057	Saliva-secreting gland	UBERON:0001044	8.56629E-19	0.113713993	0.024354244	0.019078849	0.268876359	1.719447821	NA
AdultFallopiantube_Stromal cell	Adult	Reproductive system	AdultFallopiantube	Stromal cell	Stromal cell	CL:0000499	Fallopian tube	UBERON:0003889	0.001681888	0.010416235	0.268634686	0.019247976	0.268876359	1.715614943	NA
TS_Blood_cd8-positive, alpha-beta cytokine secreting effector t cell	Adult	Lymphatic system	TS_Blood	cd8-positive, alpha-beta cytokine secreting effector t cell	T cell	CL:0000084	Blood	UBERON:0000178	1.81137E-19	0.135164026	0.02103321	0.019510821	0.269766967	1.70972445	NA
Fetal_HCA_Thymus_Vascular endothelial cells	Fetal	Lymphatic system	Fetal_HCA_Thymus	Vascular endothelial cells	Endothelial cell	CL:0000115	Thymus	UBERON:0002370	0.001044046	0.01251595	0.249077491	0.021107394	0.285958197	1.675565389	NA
TS_Small_Intestine_intestinal tuft cell	Adult	Digestive system	TS_Small_Intestine	intestinal tuft cell	Intestinal tuft cell	CL:0019032	Small intestine	UBERON:0002108	6.98976E-12	0.043970509	0.071217712	0.02118837	0.285958197	1.673902462	NA
TS_Small_Intestine_monocyte	Adult	Digestive system	TS_Small_Intestine	monocyte	Monocyte	CL:0000576	Small intestine	UBERON:0002108	3.89505E-16	0.07768966	0.040590406	0.021314965	0.285958197	1.671315371	NA
TS_Fat_mesenchymal stem cell	Adult	Integumentary system	TS_Fat	mesenchymal stem cell	Mesenchymal stem cell	CL:0000134	Perirenal fat	UBERON:0005406	4.03739E-18	0.120424848	0.026568266	0.021579743	0.286672081	1.665953724	NA
Fetal_HCA_Thymus_Thymic epithelial cells	Fetal	Lymphatic system	Fetal_HCA_Thymus	Thymic epithelial cells	Epithelial cell	CL:0000066	Thymus	UBERON:0002370	0.001044046	0.013792248	0.249077491	0.022926211	0.3016021	1.639667709	NA
FetalBrain_Fetal neuron	Fetal	Nervous system	FetalBrain	Fetal neuron	Neuron	CL:0000540	Brain	UBERON:0000955	0.000415028	0.015356741	0.22804428	0.023303879	0.303333779	1.632517788	NA
TS_Muscle_skeletal muscle satellite stem cell	Adult	Muscular system	TS_Muscle	skeletal muscle satellite stem cell	Skeletal muscle satellite cell	CL:0000594	Muscle organ	UBERON:0001630	4.21168E-18	0.126147602	0.02804428	0.02350557	0.303333779	1.62882922	NA
TS_Small_Intestine_enterocyte of epithelium of small intestine	Adult	Digestive system	TS_Small_Intestine	enterocyte of epithelium of small intestine	Enterocyte	CL:0000584	Small intestine	UBERON:0002108	8.72385E-17	0.10473874	0.034686347	0.024042115	0.307330815	1.619027323	NA
TS_Eye_corneal epithelial cell	Adult	Sensory system	TS_Eye	corneal epithelial cell	Epithelial cell	CL:0000066	Eye	UBERON:0000970	3.6911E-20	0.208448207	0.017712177	0.02437346	0.307410106	1.613082816	NA
Fetal_HCA_Cerebellum_10k_Inhibitory interneurons	Fetal	Nervous system	Fetal_HCA_Cerebellum_10k	Inhibitory interneurons	Inhibitory neuron	CL:0008029	Cerebellum	UBERON:0002037	4.67401E-05	0.018238704	0.203690037	0.02450206	0.307410106	1.610797399	NA
Madissoon_2020_spleen_T_CD8_gd	Adult	Lymphatic system	Madissoon_2020_spleen	T_CD8_gd	Thymocyte	CL:0000893	Spleen	UBERON:0002106	0.001204099	0.014574494	0.26199262	0.025079016	0.311762077	1.600689511	NA
Fetal_HCA_Lung_Stromal cells	Fetal	Respiratory system	Fetal_HCA_Lung	Stromal cells	Stromal cell	CL:0000499	Lung	UBERON:0002048	0.000263139	0.018279874	0.225092251	0.02671296	0.3276017	1.573207499	NA
FetalAdrenalGland_Fetal neuron	Fetal	Endocrine system	FetalAdrenalGland	Fetal neuron	Neuron	CL:0000540	Adrenal gland	UBERON:0002369	0.000393239	0.018197533	0.227306273	0.026836744	0.3276017	1.57127017	NA
TS_Lymph_Node_b cell	Adult	Lymphatic system	TS_Lymph_Node	b cell	B cell	CL:0000236	Lymph node	UBERON:0000029	3.93869E-16	0.099510037	0.042066421	0.027108747	0.327967425	1.566890562	NA
TS_Lung_type ii pneumocyte	Adult	Respiratory system	TS_Lung	type ii pneumocyte	Pneumocyte	CL:0000322	Lung	UBERON:0002048	8.63795E-19	0.177405358	0.025092251	0.028554208	0.342397809	1.544329875	NA
TS_Blood_nk cell	Adult	Lymphatic system	TS_Blood	nk cell	Natural killer cell	CL:0000623	Blood	UBERON:0000178	4.22308E-18	0.159619534	0.028782288	0.02932468	0.344994522	1.532766711	NA
van_Zyl_eye_2020_SchwannCell-nmy	Adult	Sensory system	van_Zyl_eye_2020	SchwannCell-nmy	Schwann cell	CL:0002573	Eye	UBERON:0000970	0.001584144	0.017250602	0.267896679	0.029470821	0.344994522	1.530607762	NA
TS_Salivary_Gland_endothelial cell of lymphatic vessel	Adult	Digestive system	TS_Salivary_Gland	endothelial cell of lymphatic vessel	Endothelial cell	CL:0000115	Saliva-secreting gland	UBERON:0001044	8.75536E-17	0.13117045	0.035424354	0.02960652	0.344994522	1.528612634	NA
Fetal_HCA_Liver_Erythroblasts	Fetal	Digestive system	Fetal_HCA_Liver	Erythroblasts	Erythroblast	CL:0000765	Liver	UBERON:0002107	0.000586033	0.019103292	0.24501845	0.029789195	0.344994522	1.525941234	NA
TS_Uterus_vascular associated smooth muscle cell	Adult	Reproductive system	TS_Uterus	vascular associated smooth muscle cell	Smooth muscle cell	CL:0000192	Uterus	UBERON:0000995	1.04562E-09	0.049734433	0.095571956	0.030177859	0.345497209	1.520311569	NA
Voigt_2019_retina_2_Choriocapillaris	Adult	Sensory system	Voigt_2019_retina_2	Choriocapillaris	Capillary endothelial cell	CL:0002144	Retina	UBERON:0000966	0.000619732	0.019350317	0.247232472	0.030342559	0.345497209	1.51794779	NA
TS_Skin_stromal cell	Adult	Integumentary system	TS_Skin	stromal cell	Stromal cell	CL:0000499	Skin of body	UBERON:0002097	3.7634E-16	0.141916052	0.037638376	0.033289503	0.375016614	1.477692685	NA
TS_Eye_limbal stem cell	Adult	Sensory system	TS_Eye	limbal stem cell	Stem cell	CL:0000034	Eye	UBERON:0000970	9.11517E-17	0.145786115	0.036900369	0.033488568	0.375016614	1.475103417	NA
Fetal_HCA_Spleen_Megakaryocytes	Fetal	Lymphatic system	Fetal_HCA_Spleen	Megakaryocytes	Megakaryocyte	CL:0000556	Spleen	UBERON:0002106	0.003140579	0.018979779	0.291143911	0.034250962	0.377939442	1.465327223	NA
Fetal_HCA_Liver_Megakaryocytes	Fetal	Digestive system	Fetal_HCA_Liver	Megakaryocytes	Megakaryocyte	CL:0000556	Liver	UBERON:0002107	0.001276264	0.020955982	0.264206642	0.034307418	0.377939442	1.464611967	NA
TS_Thymus_vascular associated smooth muscle cell	Adult	Lymphatic system	TS_Thymus	vascular associated smooth muscle cell	Smooth muscle cell	CL:0000192	Thymus	UBERON:0002370	3.86127E-16	0.149738521	0.038376384	0.03539324	0.386756777	1.451079678	NA
AdultPleura_Fibroblast	Adult	Respiratory system	AdultPleura	Fibroblast	Fibroblast	CL:0000057	Pleura	UBERON:0000977	0.00055052	0.024249653	0.243542435	0.036213405	0.388687333	1.441130635	NA
Fetal_HCA_Intestine_Lymphatic endothelial cells	Fetal	Digestive system	Fetal_HCA_Intestine	Lymphatic endothelial cells	Endothelial cell	CL:0000115	Intestine	UBERON:0000160	0.001215186	0.022520476	0.263099631	0.036312465	0.388687333	1.439944263	NA
TS_Bone_Marrow_myeloid progenitor	Adult	Lymphatic system	TS_Bone_Marrow	myeloid progenitor	Myeloid cell	CL:0000763	Bone marrow	UBERON:0002371	1.93897E-17	0.183416307	0.032472325	0.036470473	0.388687333	1.438058603	NA
Fetal_HCA_Intestine_ENS glia	Fetal	Digestive system	Fetal_HCA_Intestine	ENS glia	Enteric neural system glia	NA	Intestine	UBERON:0000160	0.000556081	0.02457902	0.244280443	0.036717327	0.388687333	1.435128938	NA
Fetal_HCA_Intestine_Chromaffin cells	Fetal	Digestive system	Fetal_HCA_Intestine	Chromaffin cells	Neuron	CL:0000540	Intestine	UBERON:0000160	0.001215186	0.023755603	0.263099631	0.037970294	0.398835263	1.420566035	NA
TS_Eye_retinal blood vessel endothelial cell	Adult	Sensory system	TS_Eye	retinal blood vessel endothelial cell	Endothelial cell	CL:0000115	Eye	UBERON:0000970	4.28544E-18	0.216847068	0.029889299	0.039140034	0.407959588	1.407378798	NA
AdultLiver_Endothelial cell (APC)	Adult	Digestive system	AdultLiver	Endothelial cell (APC)	Endothelial cell	CL:0000115	Liver	UBERON:0002107	0.001458206	0.024784875	0.267158672	0.039844406	0.412131068	1.399632645	NA
TS_Vasculature_vein or capillary endothelial cell	Adult	Cardiovascular system	TS_Vasculature	vein or capillary endothelial cell	Capillary endothelial cell	CL:0002144	Vasculature	UBERON:0002049	7.08456E-15	0.14273947	0.047232472	0.040447636	0.415201113	1.393106855	NA
FetalFemaleGonad_Fetal epithelial progenitor	Fetal	Reproductive system	FetalFemaleGonad	Fetal epithelial progenitor	Epithelial fate stem cell	CL:0000036	Immature gonad	UBERON:0005272	0.002529482	0.024661362	0.27896679	0.04113971	0.41908185	1.385788802	NA
TS_Eye_radial glial cell	Adult	Sensory system	TS_Eye	radial glial cell	Glial cell	CL:0000125	Eye	UBERON:0000970	4.28544E-18	0.23685612	0.029889299	0.042126752	0.425983196	1.375442029	NA
Fetal_HCA_Pancreas_Lymphatic endothelial cells	Fetal	Digestive system	Fetal_HCA_Pancreas	Lymphatic endothelial cells	Endothelial cell	CL:0000115	Pancreas	UBERON:0001264	0.001202534	0.027625666	0.261254613	0.042807948	0.429664955	1.368475594	NA
TS_Spleen_classical monocyte	Adult	Lymphatic system	TS_Spleen	classical monocyte	Monocyte	CL:0000576	Spleen	UBERON:0002106	8.87588E-17	0.206842543	0.036162362	0.044098091	0.436935156	1.355580208	NA
AdultBladder_Smooth muscle cell	Adult	Urinary system	AdultBladder	Smooth muscle cell	Smooth muscle cell	CL:0000192	Bladder organ	UBERON:0018707	0.003161985	0.025649463	0.292250923	0.044177208	0.436935156	1.354801738	NA
Fetal_HCA_Lung_Neuroendocrine cells	Fetal	Respiratory system	Fetal_HCA_Lung	Neuroendocrine cells	Epithelial cell	CL:0000066	Lung	UBERON:0002048	0.000594674	0.031166362	0.245756458	0.044974313	0.44159561	1.347035462	NA
TS_Bladder_myofibroblast cell	Adult	Urinary system	TS_Bladder	myofibroblast cell	Myofibroblast cell	CL:0000186	Bladder organ	UBERON:0018707	3.91623E-16	0.189427257	0.041328413	0.045797708	0.444311591	1.339156255	NA
AdultJeJunum_Smooth muscle cell	Adult	Digestive system	AdultJeJunum	Smooth muscle cell	Smooth muscle cell	CL:0000192	Jejunum	UBERON:0002115	0.003035406	0.027255128	0.288560886	0.045972398	0.444311591	1.337502843	NA
TS_Blood_naive thymus-derived cd4-positive, alpha-beta t cell	Adult	Lymphatic system	TS_Blood	naive thymus-derived cd4-positive, alpha-beta t cell	T cell	CL:0000084	Blood	UBERON:0000178	4.93106E-13	0.124047886	0.063837638	0.046234638	0.444311591	1.33503254	NA
TS_Spleen_memory b cell	Adult	Lymphatic system	TS_Spleen	memory b cell	B cell	CL:0000236	Spleen	UBERON:0002106	3.0478E-14	0.154843711	0.051660517	0.046623159	0.444890008	1.331399299	NA
TS_Blood_memory b cell	Adult	Lymphatic system	TS_Blood	memory b cell	B cell	CL:0000236	Blood	UBERON:0000178	3.08214E-14	0.15299102	0.052767528	0.046978442	0.445145377	1.328101391	NA
Fetal_HCA_Spleen_Stromal cells	Fetal	Lymphatic system	Fetal_HCA_Spleen	Stromal cells	Stromal cell	CL:0000499	Spleen	UBERON:0002106	0.003140579	0.028078546	0.291143911	0.047469073	0.446670787	1.323589253	NA
Lake_2017_VisualCortex_In6b	Adult	Nervous system	Lake_2017_VisualCortex	In6b	Inhibitory neuron	CL:0008029	Visual cortex	UBERON:0000411	2.30718E-06	0.052698737	0.158671587	0.048365296	0.451965352	1.31546615	NA
Fetal_HCA_Cerebrum_10k_Oligodendrocytes	Fetal	Nervous system	Fetal_HCA_Cerebrum_10k	Oligodendrocytes	Oligodendrocyte	CL:0000128	Cerebral hemisphere	UBERON:0001869	0.000268167	0.037918388	0.225830258	0.049326057	0.457786353	1.306923597	NA
TS_Small_Intestine_paneth cell of epithelium of small intestine	Adult	Digestive system	TS_Small_Intestine	paneth cell of epithelium of small intestine	Paneth cell	CL:0000510	Small intestine	UBERON:0002108	1.69862E-15	0.192679757	0.04501845	0.04985385	0.459537192	1.302301295	NA
TS_Eye_ocular surface cell	Adult	Sensory system	TS_Eye	ocular surface cell	Epithelial cell	CL:0000066	Eye	UBERON:0000970	3.1149E-14	0.16641273	0.053874539	0.051231768	0.466282577	1.290460655	NA
Madissoon_2020_spleen_T_CD4_naive	Adult	Lymphatic system	Madissoon_2020_spleen	T_CD4_naive	T cell	CL:0000084	Spleen	UBERON:0002106	0.005102248	0.029231331	0.30701107	0.051273877	0.466282577	1.290103839	NA
Fetal_HCA_Muscle_Smooth muscle cells	Fetal	Muscular system	Fetal_HCA_Muscle	Smooth muscle cells	Smooth muscle cell	CL:0000192	Muscle organ	UBERON:0001630	0.003808363	0.03149573	0.293726937	0.052574327	0.471891155	1.2792628	NA
TS_Pancreas_pancreatic beta cell	Adult	Digestive system	TS_Pancreas	pancreatic beta cell	Type B pancreatic cell	CL:0000169	Pancreas	UBERON:0001264	2.87304E-10	0.104491714	0.088560886	0.052587132	0.471891155	1.27912051	NA
TS_Bladder_pericyte cell	Adult	Urinary system	TS_Bladder	pericyte cell	Pericyte cell	CL:0000669	Bladder organ	UBERON:0018707	1.22407E-13	0.161142857	0.058302583	0.053247101	0.474669881	1.273704032	NA
TS_Eye_eye photoreceptor cell	Adult	Sensory system	TS_Eye	eye photoreceptor cell	Photoreceptor cell	CL:0000210	Eye	UBERON:0000970	4.97744E-13	0.146815388	0.065313653	0.054150642	0.476087593	1.266396392	NA
Braga_2020_lung_Fibroblast	Adult	Respiratory system	Braga_2020_lung	Fibroblast	Fibroblast	CL:0000057	Lung	UBERON:0002048	0.004619231	0.031701584	0.302583026	0.054166032	0.476087593	1.266272977	NA
TS_Fat_fibroblast	Adult	Integumentary system	TS_Fat	fibroblast	Fibroblast	CL:0000057	Perirenal fat	UBERON:0005406	7.16936E-15	0.201284473	0.04797048	0.054460204	0.476087593	1.263920733	NA
AdultSpleen_Endothelial cell (APC)	Adult	Lymphatic system	AdultSpleen	Endothelial cell (APC)	Endothelial cell	CL:0000115	Spleen	UBERON:0002106	0.001812808	0.036847945	0.269372694	0.055709862	0.478655246	1.254067921	NA
TS_Bladder_fibroblast	Adult	Urinary system	TS_Bladder	fibroblast	Fibroblast	CL:0000057	Bladder organ	UBERON:0018707	1.70724E-15	0.217217606	0.045756458	0.055771099	0.478655246	1.253590798	NA
TS_Bone_Marrow_erythroid progenitor	Adult	Lymphatic system	TS_Bone_Marrow	erythroid progenitor	Erythroid progenitor cell	CL:0000038	Bone marrow	UBERON:0002371	3.94891E-16	0.234715234	0.042804428	0.056267351	0.478655246	1.249743533	NA
Fetal_HCA_Heart_Lymphatic endothelial cells	Fetal	Cardiovascular system	Fetal_HCA_Heart	Lymphatic endothelial cells	Endothelial cell	CL:0000115	Heart	UBERON:0000948	0.002650648	0.036271553	0.279704797	0.056719908	0.478655246	1.246264485	NA
TS_Large_Intestine_cd8-positive, alpha-beta t cell	Adult	Digestive system	TS_Large_Intestine	cd8-positive, alpha-beta t cell	T cell	CL:0000084	Large intestine	UBERON:0000059	1.21245E-13	0.18263406	0.055719557	0.056861998	0.478655246	1.245177886	NA
TS_Large_Intestine_fibroblast	Adult	Digestive system	TS_Large_Intestine	fibroblast	Fibroblast	CL:0000057	Large intestine	UBERON:0000059	7.1911E-15	0.205854441	0.049446494	0.056873428	0.478655246	1.245090596	NA
TS_Blood_cd8-positive, alpha-beta t cell	Adult	Lymphatic system	TS_Blood	cd8-positive, alpha-beta t cell	T cell	CL:0000084	Blood	UBERON:0000178	3.08214E-14	0.197167384	0.052767528	0.057904299	0.481801949	1.237289191	NA
TS_Salivary_Gland_memory b cell	Adult	Digestive system	TS_Salivary_Gland	memory b cell	B cell	CL:0000236	Saliva-secreting gland	UBERON:0001044	8.84061E-13	0.168018395	0.06199262	0.057958463	0.481801949	1.236883138	NA
TS_Thymus_fibroblast	Adult	Lymphatic system	TS_Thymus	fibroblast	Fibroblast	CL:0000057	Thymus	UBERON:0002370	7.17638E-15	0.217135265	0.048708487	0.058689503	0.484904126	1.231439568	NA
TS_Eye_corneal keratocyte	Adult	Sensory system	TS_Eye	corneal keratocyte	Fibroblast	CL:0000057	Eye	UBERON:0000970	1.76722E-15	0.230845171	0.046494465	0.059401163	0.485858255	1.226205049	NA
TS_Salivary_Gland_macrophage	Adult	Digestive system	TS_Salivary_Gland	macrophage	Macrophage	CL:0000235	Saliva-secreting gland	UBERON:0001044	1.22376E-13	0.188768523	0.057195572	0.059689822	0.485858255	1.224099715	NA
TS_Skin_macrophage	Adult	Integumentary system	TS_Skin	macrophage	Macrophage	CL:0000235	Skin of body	UBERON:0002097	3.11044E-10	0.12145412	0.0900369	0.060316719	0.485858255	1.219562293	NA
Fetal_HCA_Muscle_Skeletal muscle cells	Fetal	Muscular system	Fetal_HCA_Muscle	Skeletal muscle cells	Cell of skeletal muscle	CL:0000188	Muscle organ	UBERON:0001630	0.007560156	0.034213008	0.321402214	0.060591036	0.485858255	1.217591623	NA
Fetal_HCA_Liver_Vascular endothelial cells	Fetal	Digestive system	Fetal_HCA_Liver	Vascular endothelial cells	Endothelial cell	CL:0000115	Liver	UBERON:0002107	0.002670838	0.039112344	0.281180812	0.060597819	0.485858255	1.217543005	NA
TS_Salivary_Gland_adventitial cell	Adult	Digestive system	TS_Salivary_Gland	adventitial cell	Adventitial cell	CL:0002503	Saliva-secreting gland	UBERON:0001044	3.17882E-10	0.122195196	0.092250923	0.061834586	0.489677813	1.208768546	NA
TS_Prostate_basal cell of prostate epithelium	Adult	Reproductive system	TS_Prostate	basal cell of prostate epithelium	Basal cell	CL:0000646	Prostate gland	UBERON:0002367	1.81818E-12	0.166989123	0.067896679	0.062127683	0.489677813	1.206714843	NA
TS_Large_Intestine_paneth cell of epithelium of large intestine	Adult	Digestive system	TS_Large_Intestine	paneth cell of epithelium of large intestine	Paneth cell	CL:0000510	Large intestine	UBERON:0000059	1.21245E-13	0.204289948	0.055719557	0.062328901	0.489677813	1.205310534	NA
TS_Salivary_Gland_ionocyte	Adult	Digestive system	TS_Salivary_Gland	ionocyte	Ionocyte	CL:0005006	Saliva-secreting gland	UBERON:0001044	2.56914E-11	0.144015767	0.079335793	0.06251975	0.489677813	1.203982766	NA
TS_Kidney_cd8-positive, alpha-beta t cell	Adult	Urinary system	TS_Kidney	cd8-positive, alpha-beta t cell	T cell	CL:0000084	Kidney	UBERON:0002113	8.37102E-05	0.056239433	0.205904059	0.063208827	0.492229659	1.199222268	NA
Fetal_HCA_Adrenal_Schwann cells	Fetal	Endocrine system	Fetal_HCA_Adrenal	Schwann cells	Schwann cell	CL:0002573	Adrenal gland	UBERON:0002369	0.001281538	0.044752756	0.264944649	0.064440875	0.498956486	1.190838574	NA
TS_Mammary_vascular associated smooth muscle cell	Adult	Endocrine system	TS_Mammary	vascular associated smooth muscle cell	Smooth muscle cell	CL:0000192	Mammary gland	UBERON:0001911	3.45748E-09	0.115113804	0.104428044	0.065167529	0.500977303	1.185968749	NA
Lake_2017_FrontalCortex_Ex3e	Adult	Nervous system	Lake_2017_FrontalCortex	Ex3e	Excitatory neuron	CL:0008030	Frontal cortex	UBERON:0001870	0.000100146	0.056980509	0.212546125	0.065564548	0.500977303	1.18333026	NA
Fetal_HCA_Liver_Stellate cells	Fetal	Digestive system	Fetal_HCA_Liver	Stellate cells	Hepatic stellate cell	CL:0000632	Liver	UBERON:0002107	0.002670838	0.043270604	0.281180812	0.06581104	0.500977303	1.181701243	NA
VentoTormo_2018_decidua_decidual macrophage (dM2)	Maternal-Fetal	Reproductive system	VentoTormo_2018_decidua	decidual macrophage (dM2)	Macrophage	CL:0000235	Decidua	UBERON:0002450	1.89852E-08	0.099015986	0.124723247	0.066615347	0.502426255	1.176425704	NA
TS_Lymph_Node_hematopoietic stem cell	Adult	Lymphatic system	TS_Lymph_Node	hematopoietic stem cell	Hematopoietic stem cell	CL:0000037	Lymph node	UBERON:0000029	3.19141E-10	0.133270165	0.093357934	0.067020283	0.502426255	1.173793744	NA
TS_Bone_Marrow_erythrocyte	Adult	Lymphatic system	TS_Bone_Marrow	erythrocyte	Erythrocyte	CL:0000232	Bone marrow	UBERON:0002371	6.29649E-06	0.070072852	0.177859779	0.067113765	0.502426255	1.173188394	NA
TS_Eye_melanocyte	Adult	Sensory system	TS_Eye	melanocyte	Melanocyte	CL:0000148	Eye	UBERON:0000970	4.97744E-13	0.193709029	0.065313653	0.0679398	0.505683936	1.167875734	NA
Fetal_HCA_Muscle_Schwann cells	Fetal	Muscular system	Fetal_HCA_Muscle	Schwann cells	Schwann cell	CL:0002573	Muscle organ	UBERON:0001630	0.007560156	0.03985342	0.321402214	0.068625492	0.505683936	1.163514526	NA
TS_Bladder_nk cell	Adult	Urinary system	TS_Bladder	nk cell	Natural killer cell	CL:0000623	Bladder organ	UBERON:0018707	4.84027E-13	0.209271625	0.061254613	0.06866852	0.505683936	1.163242315	NA
TS_Small_Intestine_cd4-positive, alpha-beta t cell	Adult	Digestive system	TS_Small_Intestine	cd4-positive, alpha-beta t cell	T cell	CL:0000084	Small intestine	UBERON:0002108	6.98976E-12	0.186174757	0.071217712	0.070578442	0.511918675	1.151327931	NA
TS_Trachea_connective tissue cell	Adult	Respiratory system	TS_Trachea	connective tissue cell	Connective tissue cell	CL:0002320	Trachea	UBERON:0003126	1.04563E-09	0.137346083	0.096678967	0.070662689	0.511918675	1.150808612	NA
TS_Small_Intestine_cd8-positive, alpha-beta t cell	Adult	Digestive system	TS_Small_Intestine	cd8-positive, alpha-beta t cell	T cell	CL:0000084	Small intestine	UBERON:0002108	1.85969E-12	0.195232352	0.068634686	0.071188262	0.511918675	1.147603809	NA
AdultTemporalLobe_Astrocyte	Adult	Nervous system	AdultTemporalLobe	Astrocyte	Astrocyte	CL:0000127	Temporal lobe	UBERON:0001871	0.006928454	0.042282502	0.319557196	0.071668654	0.511918675	1.14467075	NA
Fetal_HCA_Pancreas_Erythroblasts	Fetal	Digestive system	Fetal_HCA_Pancreas	Erythroblasts	Erythroblast	CL:0000765	Pancreas	UBERON:0001264	0.005099535	0.044464559	0.305535055	0.071986119	0.511918675	1.142751238	NA
TS_Skin_muscle cell	Adult	Integumentary system	TS_Skin	muscle cell	Cell of skeletal muscle	CL:0000188	Skin of body	UBERON:0002097	1.10421E-08	0.117048835	0.116605166	0.072256869	0.511918675	1.14112086	NA
TS_Blood_plasma cell	Adult	Lymphatic system	TS_Blood	plasma cell	Plasma cell	CL:0000786	Blood	UBERON:0000178	1.24826E-13	0.233191911	0.05904059	0.072768592	0.511918675	1.138056026	NA
TS_Bone_Marrow_monocyte	Adult	Lymphatic system	TS_Bone_Marrow	monocyte	Monocyte	CL:0000576	Bone marrow	UBERON:0002371	4.86907E-13	0.222199284	0.062730627	0.073499968	0.511918675	1.133712852	NA
Fetal_HCA_Liver_Mesothelial cells	Fetal	Digestive system	Fetal_HCA_Liver	Mesothelial cells	Mesothelial cell	CL:0000077	Liver	UBERON:0002107	0.002670838	0.049693262	0.281180812	0.073645601	0.511918675	1.132853188	NA
AdultArtery_Endothelial cell	Adult	Cardiovascular system	AdultArtery	Endothelial cell	Endothelial cell	CL:0000115	Artery	UBERON:0001637	0.003097288	0.048622819	0.289667897	0.074122093	0.511918675	1.130052328	NA
TS_Skin_langerhans cell	Adult	Integumentary system	TS_Skin	langerhans cell	Langerhans cell	CL:0000453	Skin of body	UBERON:0002097	3.42616E-08	0.112849405	0.125461255	0.074436308	0.511918675	1.128215178	NA
TS_Small_Intestine_intestinal crypt stem cell of small intestine	Adult	Digestive system	TS_Small_Intestine	intestinal crypt stem cell of small intestine	Intestinal crypt stem cell	CL:0002250	Small intestine	UBERON:0002108	4.82216E-13	0.234221183	0.060516605	0.074504601	0.511918675	1.127816908	NA
TS_Vasculature_pericyte cell	Adult	Cardiovascular system	TS_Vasculature	pericyte cell	Pericyte cell	CL:0000669	Vasculature	UBERON:0002049	2.5238E-11	0.184527921	0.077121771	0.074746456	0.511918675	1.126409394	NA
ChorionicVillus_Stromal cell	Adult	Reproductive system	ChorionicVillus	Stromal cell	Stromal cell	CL:0000499	Chorionic villus	UBERON:0007106	0.001878178	0.052986933	0.270110701	0.075091588	0.511918675	1.124408711	NA
FetalSpinalCord_Endothelial cell	Fetal	Nervous system	FetalSpinalCord	Endothelial cell	Endothelial cell	CL:0000115	Spinal cord	UBERON:0002240	0.006644314	0.04495861	0.318819188	0.075182152	0.511918675	1.123885246	NA
TS_Mammary_luminal epithelial cell of mammary gland	Adult	Endocrine system	TS_Mammary	luminal epithelial cell of mammary gland	Epithelial cell	CL:0000066	Mammary gland	UBERON:0001911	1.81763E-12	0.215941309	0.067158672	0.075897166	0.514203302	1.119774438	NA
Madissoon_2020_lung_Muscle_cells	Adult	Respiratory system	Madissoon_2020_lung	Muscle_cells	Muscle cell	CL:0000187	Lung	UBERON:0002048	0.003267299	0.050393167	0.29298893	0.077005278	0.519151182	1.113479507	NA
TS_Pancreas_pancreatic stellate cell	Adult	Digestive system	TS_Pancreas	pancreatic stellate cell	Pancreatic stellate cell	CL:0002410	Pancreas	UBERON:0001264	4.30691E-13	0.248630994	0.059778598	0.077418799	0.519319169	1.111153572	NA
TS_Lung_basal cell	Adult	Respiratory system	TS_Lung	basal cell	Basal cell	CL:0000646	Lung	UBERON:0002048	2.57443E-11	0.186257099	0.080442804	0.077924354	0.519792822	1.108326791	NA
TS_Trachea_basal cell	Adult	Respiratory system	TS_Trachea	basal cell	Basal cell	CL:0000646	Trachea	UBERON:0003126	2.49118E-11	0.199349441	0.07601476	0.078639448	0.519792822	1.104359544	NA
TS_Bone_Marrow_cd8-positive, alpha-beta t cell	Adult	Lymphatic system	TS_Bone_Marrow	cd8-positive, alpha-beta t cell	T cell	CL:0000084	Bone marrow	UBERON:0002371	4.01881E-05	0.076824878	0.197416974	0.078694052	0.519792822	1.10405809	NA
Fetal_HCA_Heart_Smooth muscle cells	Fetal	Cardiovascular system	Fetal_HCA_Heart	Smooth muscle cells	Smooth muscle cell	CL:0000192	Heart	UBERON:0000948	0.005329234	0.04961092	0.308487085	0.079270598	0.519792822	1.100887864	NA
TS_Spleen_innate lymphoid cell	Adult	Lymphatic system	TS_Spleen	innate lymphoid cell	Innate lymphoid cell	CL:0001065	Spleen	UBERON:0002106	3.49201E-08	0.118078108	0.129889299	0.079407464	0.519792822	1.100136672	NA
Fetal_HCA_Intestine_Mesothelial cells	Fetal	Digestive system	Fetal_HCA_Intestine	Mesothelial cells	Mesothelial cell	CL:0000077	Intestine	UBERON:0000160	0.005143845	0.050187313	0.307749077	0.079858322	0.520230897	1.097679819	NA
TS_Skin_endothelial cell	Adult	Integumentary system	TS_Skin	endothelial cell	Endothelial cell	CL:0000115	Skin of body	UBERON:0002097	1.81579E-12	0.238091247	0.066420664	0.08139318	0.523946359	1.089411984	NA
Madissoon_2020_oesophagus_Muscle	Adult	Digestive system	Madissoon_2020_oesophagus	Muscle	Muscle cell	CL:0000187	Esophagus	UBERON:0001043	0.006128468	0.050393167	0.317343173	0.082129282	0.523946359	1.085501973	NA
AdultCervix_Smooth muscle cell	Adult	Reproductive system	AdultCervix	Smooth muscle cell	Smooth muscle cell	CL:0000192	Uterine cervix	UBERON:0000002	0.002679793	0.056486459	0.283394834	0.082195623	0.523946359	1.085151311	NA
TS_Thymus_mature nk t cell	Adult	Lymphatic system	TS_Thymus	mature nk t cell	Natural killer cell	CL:0000623	Thymus	UBERON:0002370	9.62109E-05	0.076619023	0.209594096	0.082406097	0.523946359	1.084040656	NA
TS_Salivary_Gland_endothelial cell	Adult	Digestive system	TS_Salivary_Gland	endothelial cell	Endothelial cell	CL:0000115	Saliva-secreting gland	UBERON:0001044	2.56914E-11	0.204042922	0.079335793	0.082938593	0.523946359	1.081243336	NA
FetalPancreas_Fetal epithelial progenitor	Fetal	Digestive system	FetalPancreas	Fetal epithelial progenitor	Epithelial fate stem cell	CL:0000036	Pancreas	UBERON:0001264	0.007285053	0.050681363	0.320295203	0.08312446	0.523946359	1.080271161	NA
Fetal_HCA_Adrenal_SLC26A4_PAEP positive cells	Fetal	Endocrine system	Fetal_HCA_Adrenal	SLC26A4_PAEP positive cells	Cortical cell of adrenal gland	CL:0002097	Adrenal gland	UBERON:0002369	0.005373677	0.052739908	0.309594096	0.083515513	0.523946359	1.078233849	NA
TS_Bone_Marrow_cd24 neutrophil	Adult	Lymphatic system	TS_Bone_Marrow	cd24 neutrophil	Neutrophil	CL:0000775	Bone marrow	UBERON:0002371	7.04769E-12	0.225781151	0.072324723	0.083522076	0.523946359	1.078198717	NA
AdultArtery_Smooth muscle cell	Adult	Cardiovascular system	AdultArtery	Smooth muscle cell	Smooth muscle cell	CL:0000192	Artery	UBERON:0001637	0.006146824	0.052204686	0.318081181	0.084654489	0.527437654	1.072350007	NA
Fetal_HCA_Pancreas_Mesothelial cells	Fetal	Digestive system	Fetal_HCA_Pancreas	Mesothelial cells	Mesothelial cell	CL:0000077	Pancreas	UBERON:0001264	0.005099535	0.054510256	0.305535055	0.084857128	0.527437654	1.071311671	NA
Fetal_HCA_Cerebellum_10k_Oligodendrocytes	Fetal	Nervous system	Fetal_HCA_Cerebellum_10k	Oligodendrocytes	Oligodendrocyte	CL:0000128	Cerebellum	UBERON:0002037	0.002714491	0.058997883	0.284870849	0.085478815	0.528755457	1.068141506	NA
Voigt_2019_retina_2_Pericytes	Adult	Sensory system	Voigt_2019_retina_2	Pericytes	Pericyte cell	CL:0000669	Retina	UBERON:0000966	0.005598925	0.05422206	0.31402214	0.086376908	0.528755457	1.063602347	NA
TS_Salivary_Gland_nk cell	Adult	Digestive system	TS_Salivary_Gland	nk cell	Natural killer cell	CL:0000623	Saliva-secreting gland	UBERON:0001044	3.52303E-09	0.158384407	0.107749077	0.088535084	0.528755457	1.062807782	NA
TS_Thymus_memory b cell	Adult	Lymphatic system	TS_Thymus	memory b cell	B cell	CL:0000236	Thymus	UBERON:0002370	2.36004E-06	0.105644499	0.162730627	0.087046802	0.528755457	1.060247181	NA
TS_Spleen_cd8-positive, alpha-beta memory t cell	Adult	Lymphatic system	TS_Spleen	cd8-positive, alpha-beta memory t cell	T cell	CL:0000084	Spleen	UBERON:0002106	9.20992E-11	0.201819694	0.085608856	0.087395861	0.528755457	1.058509133	NA
TS_Large_Intestine_cd4-positive, alpha-beta t cell	Adult	Digestive system	TS_Large_Intestine	cd4-positive, alpha-beta t cell	T cell	CL:0000084	Large intestine	UBERON:0000059	9.08263E-11	0.204825169	0.084870849	0.08782626	0.528755457	1.056375612	NA
TS_Lymph_Node_macrophage	Adult	Lymphatic system	TS_Lymph_Node	macrophage	Macrophage	CL:0000235	Lymph node	UBERON:0000029	7.05098E-12	0.239038177	0.073062731	0.08815469	0.528755457	1.054754577	NA
Fetal_HCA_Placenta_Smooth muscle cells	Fetal	Reproductive system	Fetal_HCA_Placenta	Smooth muscle cells	Smooth muscle cell	CL:0000192	Placenta	UBERON:0001987	0.008331788	0.054180889	0.32398524	0.088514804	0.528755457	1.052984089	NA
Fetal_HCA_Cerebellum_10k_Astrocytes	Fetal	Nervous system	Fetal_HCA_Cerebellum_10k	Astrocytes	Astrocyte	CL:0000127	Cerebellum	UBERON:0002037	0.000599805	0.071555004	0.246494465	0.088854592	0.528755457	1.051320123	NA
TS_Tongue_keratinocyte	Adult	Digestive system	TS_Tongue	keratinocyte	Keratinocyte	CL:0000312	Tongue	UBERON:0001723	1.88548E-12	0.257029855	0.069372694	0.08963258	0.528755457	1.047534105	NA
TS_Blood_monocyte	Adult	Lymphatic system	TS_Blood	monocyte	Monocyte	CL:0000576	Blood	UBERON:0000178	4.93106E-13	0.27950916	0.063837638	0.089682312	0.528755457	1.047293206	NA
TS_Prostate_smooth muscle cell	Adult	Reproductive system	TS_Prostate	smooth muscle cell	Smooth muscle cell	CL:0000192	Prostate gland	UBERON:0002367	3.11355E-10	0.196755675	0.090774908	0.089751849	0.528755457	1.046956596	NA
TS_Lymph_Node_memory b cell	Adult	Lymphatic system	TS_Lymph_Node	memory b cell	B cell	CL:0000236	Lymph node	UBERON:0000029	6.31567E-06	0.1015274	0.181549815	0.092044399	0.532014289	1.036002635	NA
TS_Spleen_nk cell	Adult	Lymphatic system	TS_Spleen	nk cell	Natural killer cell	CL:0000623	Spleen	UBERON:0002106	2.58486E-11	0.225163588	0.081918819	0.092095723	0.532014289	1.035760538	NA
AdultEsophagus_Fibroblast	Adult	Digestive system	AdultEsophagus	Fibroblast	Fibroblast	CL:0000057	Esophagus	UBERON:0001043	0.004271887	0.062703263	0.294464945	0.092170692	0.532014289	1.035407154	NA
TS_Vasculature_fibroblast	Adult	Cardiovascular system	TS_Vasculature	fibroblast	Fibroblast	CL:0000057	Vasculature	UBERON:0002049	2.5238E-11	0.240396817	0.077121771	0.092473586	0.532014289	1.033982303	NA

Madissoon_2020_spleen_Platelet	Adult	Lymphatic system	Madissoon_2020_spleen	Platelet	Platelet	CL:0000233	Spleen	UBERON:0002106	0.009869477	0.05607475	0.331365314	0.092638631	0.532014289	1.033207873	NA
TS_Spleen_hematopoietic stem cell	Adult	Lymphatic system	TS_Spleen	hematopoietic stem cell	Hematopoietic stem cell	CL:0000037	Spleen	UBERON:0002106	2.58486E-11	0.226892765	0.08191819	0.092660791	0.532014289	1.033103995	NA
TS_Lymph_Node_regulatory t cell	Adult	Lymphatic system	TS_Lymph_Node	regulatory t cell	T cell	CL:0000084	Lymph node	UBERON:0000029	3.19141E-10	0.201655011	0.093357934	0.093612971	0.53334458	1.02866397	NA
AdultArtery_Fibroblast	Adult	Cardiovascular system	AdultArtery	Fibroblast	Fibroblast	CL:0000057	Artery	UBERON:0001637	0.003097288	0.065050003	0.289667897	0.093679712	0.53334458	1.028354452	NA
TS_Muscle_endothelial cell of vascular tree	Adult	Muscular system	TS_Muscle	endothelial cell of vascular tree	Endothelial cell	CL:0000115	Muscle organ	UBERON:0001630	7.10594E-12	0.256906343	0.073800738	0.09414395	0.533744988	1.026207584	NA
TS_Eye_muller cell	Adult	Sensory system	TS_Eye	muller cell	Mueller cell	CL:0000636	Eye	UBERON:0000970	2.62769E-11	0.230598145	0.083394834	0.095215928	0.53757326	1.021290395	NA
TS_Thymus_endothelial cell of artery	Adult	Lymphatic system	TS_Thymus	endothelial cell of artery	Endothelial cell	CL:0000115	Thymus	UBERON:0002370	2.54525E-11	0.252706923	0.078228782	0.097335263	0.542259254	1.011729793	NA
TS_Lung_club cell	Adult	Respiratory system	TS_Lung	club cell	Club cell	CL:0000158	Lung	UBERON:0002048	3.4835E-08	0.153238046	0.129151292	0.097421326	0.542259254	1.011345963	NA
TS_Thymus_t follicular helper cell	Adult	Lymphatic system	TS_Thymus	t follicular helper cell	T cell	CL:0000084	Thymus	UBERON:0002370	1.06435E-09	0.203754726	0.097416974	0.097649835	0.542259254	1.010328486	NA
Lake_2017_VisualCortex_Ex6a	Adult	Nervous system	Lake_2017_VisualCortex	Ex6a	Excitatory neuron	CL:0008030	Visual cortex	UBERON:0000411	0.005010362	0.065461712	0.304059041	0.097865558	0.542259254	1.009370123	NA
Fetal_HCA_Pancreas_Vascular endothelial cells	Fetal	Digestive system	Fetal_HCA_Pancreas	Vascular endothelial cells	Endothelial cell	CL:0000115	Pancreas	UBERON:0001264	0.005099535	0.065297029	0.305535055	0.098046676	0.542259254	1.008566238	NA
Voigt_2019_retina_1_Pericyte	Adult	Sensory system	Voigt_2019_retina_1	Pericyte	Pericyte cell	CL:0000669	Retina	UBERON:0000966	0.009158302	0.060932915	0.330258303	0.098723609	0.543782479	1.005578978	NA
TS_Liver_hepatocyte	Adult	Digestive system	TS_Liver	hepatocyte	Hepatocyte	CL:0000182	Liver	UBERON:0002107	3.44414E-09	0.199678808	0.103321033	0.10699176	0.55150299	0.996974082	NA
Fetal_HCA_Kidney_Mesangial cells	Fetal	Urinary system	Fetal_HCA_Kidney	Mesangial cells	Mesangial cell	CL:0000650	Kidney	UBERON:0002113	0.002711703	0.072831301	0.284132841	0.100942587	0.55150299	0.995925608	NA
TS_Salivary_Gland_naive b cell	Adult	Digestive system	TS_Salivary_Gland	naive b cell	B cell	CL:0000236	Saliva-secreting gland	UBERON:0001044	1.12398E-08	0.173452952	0.120664207	0.101855656	0.553184038	0.992014848	NA
TS_Bone_Marrow_cd4-positive, alpha-beta t cell	Adult	Lymphatic system	TS_Bone_Marrow	cd4-positive, alpha-beta t cell	T cell	CL:0000084	Bone marrow	UBERON:0002371	3.52809E-09	0.192762099	0.108856089	0.102063476	0.553184038	0.991129646	NA
TS_Lymph_Node_cd4-positive alpha-beta t cell	Adult	Lymphatic system	TS_Lymph_Node	cd4-positive alpha-beta t cell	T cell	CL:0000084	Lymph node	UBERON:0000029	3.53497E-09	0.192391561	0.110332103	0.103003488	0.553926989	0.987148087	NA
TS_Fat_myofibroblast cell	Adult	Integumentary system	TS_Fat	myofibroblast cell	Myofibroblast cell	CL:0000186	Perirenal fat	UBERON:0005406	1.11477E-08	0.185310168	0.117343173	0.10499249	0.553926989	0.978841766	NA
Voigt_2019_retina_1_Schwann cell 2	Adult	Sensory system	Voigt_2019_retina_1	Schwann cell 2	Schwann cell	CL:0002573	Retina	UBERON:0000966	0.009158302	0.065955763	0.330258303	0.105136232	0.553926989	0.97824759	NA
FetalSkin_Fetal stromal cell	Fetal	Integumentary system	FetalSkin	Fetal stromal cell	Stromal cell	CL:0000499	Skin of body	UBERON:0002097	0.008002161	0.067808453	0.322509225	0.105466735	0.553926989	0.976884499	NA
Fetal_HCA_Adrenal_Stromal cells	Fetal	Endocrine system	Fetal_HCA_Adrenal	Stromal cells	Stromal cell	CL:0000499	Adrenal gland	UBERON:0002369	0.002678108	0.077483612	0.282656827	0.105590643	0.553926989	0.976374565	NA
TS_Eye_limbal stromal cell	Adult	Sensory system	TS_Eye	limbal stromal cell	Stromal cell	CL:0000499	Eye	UBERON:0000970	1.09241E-09	0.219646689	0.100369004	0.106142132	0.553926989	0.974112192	NA
Fetal_HCA_Adrenal_Erythroblasts	Fetal	Endocrine system	Fetal_HCA_Adrenal	Erythroblasts	Erythroblast	CL:0000765	Adrenal gland	UBERON:0002369	0.005373677	0.071307978	0.309594096	0.106259639	0.553926989	0.973631664	NA
TS_Blood_cd4-positive, alpha-beta t cell	Adult	Lymphatic system	TS_Blood	cd4-positive, alpha-beta t cell	T cell	CL:0000084	Blood	UBERON:0000178	3.56051E-09	0.198814219	0.111439114	0.106561321	0.553926989	0.972400849	NA
TS_Tongue_tongue muscle cell	Adult	Digestive system	TS_Tongue	tongue muscle cell	Muscle cell	CL:0000187	Tongue	UBERON:0001723	8.69224E-07	0.143274691	0.15498155	0.106748904	0.553926989	0.971636577	NA
TS_Spleen_macrophage	Adult	Lymphatic system	TS_Spleen	macrophage	Macrophage	CL:0000235	Spleen	UBERON:0002106	2.58486E-11	0.271645521	0.08191819	0.106931446	0.553926989	0.970894724	NA
Fetal_HCA_Placenta_Syncytiotrophoblasts and villous cytotrophoblasts	Fetal	Reproductive system	Fetal_HCA_Placenta	Syncytiotrophoblasts and villous cytotrophoblasts	Trophoblast cell	CL:0000351	Placenta	UBERON:0001987	0.015698245	0.065626396	0.339114391	0.106938921	0.553926989	0.970864202	NA
TS_Pancreas_myeloid cell	Adult	Digestive system	TS_Pancreas	myeloid cell	Myeloid cell	CL:0000763	Pancreas	UBERON:0001264	2.30736E-11	0.296224541	0.075276753	0.107106178	0.553926989	0.970185478	NA
TS_Lung_type i pneumocyte	Adult	Respiratory system	TS_Lung	type i pneumocyte	Pneumocyte	CL:0000322	Lung	UBERON:0002048	1.07423E-09	0.225575297	0.099630996	0.107772841	0.554988555	0.96749067	NA
TS_Lymph_Node_cd8-positive alpha-beta t cell	Adult	Lymphatic system	TS_Lymph_Node	cd8-positive alpha-beta t cell	T cell	CL:0000084	Lymph node	UBERON:0000029	3.53497E-09	0.204660486	0.110332103	0.108176154	0.554988555	0.965868465	NA
Voigt_2019_retina_2_Fibroblasts	Adult	Sensory system	Voigt_2019_retina_2	Fibroblasts	Fibroblast	CL:0000057	Retina	UBERON:0000966	0.005598925	0.072213738	0.31402214	0.108540197	0.554988555	0.964409394	NA
TS_Eye_endothelial cell	Adult	Sensory system	TS_Eye	endothelial cell	Endothelial cell	CL:0000115	Eye	UBERON:0000970	3.58089E-09	0.206142638	0.112546125	0.110517679	0.562975395	0.956568245	NA
TS_Small_Intestine_fibroblast	Adult	Digestive system	TS_Small_Intestine	fibroblast	Fibroblast	CL:0000057	Small intestine	UBERON:0002108	1.06916E-09	0.239902766	0.098154982	0.11182142	0.567483237	0.951479906	NA
AdultTrachea_Stratified epithelial cell	Adult	Respiratory system	AdultTrachea	Stratified epithelial cell	Epithelial cell	CL:0000066	Trachea	UBERON:0003126	0.009870599	0.072543105	0.332103321	0.113855069	0.569649961	0.94364763	NA
CordBloodCD34P_CB CD34+	Neonatal	Reproductive system	CordBloodCD34P	CB CD34+	Hematopoietic stem cell	CL:0000037	Umbilical cord blood	UBERON:0012168	0.014438584	0.0718432	0.336531365	0.114174165	0.569649961	0.942431594	NA
TS_Thymus_capillary endothelial cell	Adult	Lymphatic system	TS_Thymus	capillary endothelial cell	Capillary endothelial cell	CL:0002144	Thymus	UBERON:0002370	1.1155E-08	0.204248777	0.118450185	0.114233029	0.569649961	0.942208306	NA
Segerstolpe_2016_PancreaticIslets_pancreatic A cell	Adult	Digestive system	Segerstolpe_2016_PancreaticIslets	pancreatic A cell	Pancreatic A cell	CL:0000171	Pancreas	UBERON:0001264	5.70564E-05	0.118325133	0.205166052	0.114541768	0.569649961	0.941036117	NA
TS_Tongue_fibroblast	Adult	Digestive system	TS_Tongue	fibroblast	Fibroblast	CL:0000057	Tongue	UBERON:0001723	1.05313E-07	0.174441054	0.139483395	0.114747436	0.569649961	0.940257009	NA
TS_Prostate_t cell	Adult	Reproductive system	TS_Prostate	t cell	T cell	CL:0000084	Prostate gland	UBERON:0002367	0.000497837	0.105850354	0.230258303	0.114900843	0.569649961	0.939676785	NA
TS_Prostate_fibroblast	Adult	Reproductive system	TS_Prostate	fibroblast	Fibroblast	CL:0000057	Prostate gland	UBERON:0002367	3.42865E-08	0.1937502	0.126199262	0.11519121	0.569649961	0.938580661	NA
TS_Mammary_fibroblast of breast	Adult	Endocrine system	TS_Mammary	fibroblast of breast	Fibroblast	CL:0000057	Mammary gland	UBERON:0001911	3.45748E-08	0.23640324	0.104428044	0.116065873	0.571888213	0.935295456	NA
TS_Mammary_endothelial cell of lymphatic vessel	Adult	Endocrine system	TS_Mammary	endothelial cell of lymphatic vessel	Endothelial cell	CL:0000115	Mammary gland	UBERON:0001911	8.5428E-07	0.163736623	0.15202952	0.116826309	0.571908078	0.932459344	NA
TS_Small_Intestine_intestinal enteroendocrine cell	Adult	Digestive system	TS_Small_Intestine	intestinal enteroendocrine cell	Endocrine cell	CL:0000163	Small intestine	UBERON:0002108	1.11986E-08	0.208407036	0.119557196	0.11691405	0.571908078	0.932133296	NA
VentoTormo_2018_decidua_decidual stromal cell (dS2)	Maternal-Fetal	Reproductive system	VentoTormo_2018_decidua	decidual stromal cell (dS2)	Stromal cell	CL:0000499	Decidua	UBERON:0002450	4.87383E-07	0.169088838	0.149077491	0.11798598	0.572041538	0.928169595	NA
AdultEsophagus_Smooth muscle cell	Adult	Digestive system	AdultEsophagus	Smooth muscle cell	Smooth muscle cell	CL:0000192	Esophagus	UBERON:0001043	0.015782561	0.073654719	0.343173432	0.11823986	0.572041538	0.927236095	NA
Madissoon_2020_oesophagus_Glands_duct	Adult	Digestive system	Madissoon_2020_oesophagus	Glands_duct	Salivary gland cell	CL:0009005	Esophagus	UBERON:0001043	0.011669071	0.075672093	0.334317343	0.118321316	0.572041538	0.926937009	NA
AdultSigmoidColon_Smooth muscle cell	Adult	Digestive system	AdultSigmoidColon	Smooth muscle cell	Smooth muscle cell	CL:0000192	Sigmoid colon	UBERON:0001159	0.023351502	0.071555004	0.357933579	0.119471925	0.572041538	0.922734138	NA
TS_Muscle_pericyte cell	Adult	Muscular system	TS_Muscle	pericyte cell	Pericyte cell	CL:0000669	Muscle organ	UBERON:0001630	9.25508E-11	0.296965617	0.086346863	0.119582255	0.572041538	0.922333261	NA
TS_Large_Intestine_enterocyte of epithelium of large intestine	Adult	Digestive system	TS_Large_Intestine	enterocyte of epithelium of large intestine	Enterocyte	CL:0000584	Large intestine	UBERON:0000059	3.45708E-08	0.20070808	0.12804428	0.119792741	0.572041538	0.921569408	NA
TS_Thymus_vein endothelial cell	Adult	Lymphatic system	TS_Thymus	vein endothelial cell	Endothelial cell	CL:0000115	Thymus	UBERON:0002370	3.15152E-10	0.282020585	0.095112915	0.120191605	0.572041538	0.920125865	NA
VentoTormo_2018_decidua_monocyte	Maternal-Fetal	Reproductive system	VentoTormo_2018_decidua	monocyte	Monocyte	CL:0000576	Decidua	UBERON:0002450	1.35272E-06	0.165177604	0.156457565	0.1203187	0.572041538	0.919666869	NA
TS_Heart_cardiac muscle cell	Adult	Cardiovascular system	TS_Heart	cardiac muscle cell	Muscle cell	CL:0000187	Heart	UBERON:0000948	3.41859E-09	0.25353033	0.102583026	0.120919989	0.572890158	0.917501901	NA
TS_Prostate_endothelial cell	Adult	Reproductive system	TS_Prostate	endothelial cell	Endothelial cell	CL:0000115	Prostate gland	UBERON:0002367	3.40955E-09	0.248672165	0.105535055	0.121779158	0.574950382	0.914427032	NA
TS_Bone_Marrow_memory b cell	Adult	Lymphatic system	TS_Bone_Marrow	memory b cell	B cell	CL:0000236	Bone marrow	UBERON:0002371	2.37166E-06	0.160978173	0.164575646	0.122680079	0.577220964	0.911204712	NA
AdultArtery_T cell	Adult	Cardiovascular system	AdultArtery	T cell	T cell	CL:0000084	Artery	UBERON:0001637	0.011702347	0.079871523	0.335055351	0.123658868	0.579784658	0.907774733	NA
Lake_2017_CerebellarHemisphere_Gran	Adult	Nervous system	Lake_2017_CerebellarHemisphere	Gran	Cerebellar granule cell	CL:0001031	Cerebellar hemisphere	UBERON:0002245	0.000539152	0.111079067	0.242066421	0.124113873	0.579934732	0.906162178	NA
Lake_2017_FrontalCortex_In6b	Adult	Nervous system	Lake_2017_FrontalCortex	In6b	Inhibitory neuron	CL:0008029	Frontal cortex	UBERON:0001870	0.004924	0.08991722	0.303321033	0.125509299	0.584416154	0.901324095	NA
TS_Bladder_bladder urothelial cell	Adult	Urinary system	TS_Bladder	bladder urothelial cell	Urothelial cell	CL:0000731	Bladder organ	UBERON:0018707	1.07144E-09	0.278768084	0.098892989	0.126568185	0.587328391	0.897675449	NA
FetalMaleGonad_Fetal fibroblast	Fetal	Reproductive system	FetalMaleGonad	Fetal fibroblast	Fibroblast	CL:0000057	Immature gonad	UBERON:2005272	0.012645379	0.082588802	0.335793358	0.127158652	0.587817095	0.895654085	NA
Fetal_HCA_Placenta_Stromal cells	Fetal	Reproductive system	Fetal_HCA_Placenta	Stromal cells	Stromal cell	CL:0000499	Placenta	UBERON:0001987	0.015698245	0.082094751	0.339114391	0.127541126	0.587817095	0.894349753	NA
FetalKidney_Proximal tubule progenitor	Fetal	Urinary system	FetalKidney	Proximal tubule progenitor	Epithelial cell of proximal tubule	CL:0002306	Kidney	UBERON:0002113	0.011214986	0.084976713	0.333579336	0.129352128	0.594142824	0.888224429	NA
MacParland_2018_liver_Stellate cell	Adult	Digestive system	MacParland_2018_liver	Stellate cell	Hepatic stellate cell	CL:0000632	Liver	UBERON:0002107	0.016251281	0.083782758	0.347601476	0.132108576	0.604753786	0.879068988	NA
Fetal_HCA_Stomach_Goblet cells	Fetal	Digestive system	Fetal_HCA_Stomach	Goblet cells	Goblet cell	CL:0000160	Stomach	UBERON:0000945	0.021711358	0.082918169	0.356826568	0.133747101	0.610193002	0.873715624	NA
TS_Muscle_smooth muscle cell	Adult	Muscular system	TS_Muscle	smooth muscle cell	Smooth muscle cell	CL:0000192	Muscle organ	UBERON:0001630	1.05268E-07	0.214747353	0.138745387	0.13447791	0.610721988	0.87134905	NA
VentoTormo_2018_decidua_T cell	Maternal-Fetal	Reproductive system	VentoTormo_2018_decidua	T cell	T cell	CL:0000084	Decidua	UBERON:0002450	0.00293503	0.104203518	0.286715867	0.134764483	0.610721988	0.870424551	NA
TS_Fat_endothelial cell	Adult	Integumentary system	TS_Fat	endothelial cell	Endothelial cell	CL:0000115	Perirenal fat	UBERON:0005406	3.49283E-09	0.285478939	0.106273063	0.136382577	0.615994461	0.865241106	NA
TS_Bladder_capillary endothelial cell	Adult	Urinary system	TS_Bladder	capillary endothelial cell	Capillary endothelial cell	CL:0002144	Bladder organ	UBERON:0018707	3.5166E-09	0.286796408	0.10701107	0.137609747	0.619472451	0.861350802	NA
TS_Lymph_Node_endothelial cell	Adult	Lymphatic system	TS_Lymph_Node	endothelial cell	Endothelial cell	CL:0000115	Lymph node	UBERON:0000029	1.1275E-08	0.252830425	0.123616236	0.139567921	0.626207062	0.855214939	NA
TS_Eye_fibroblast	Adult	Sensory system	TS_Eye	fibroblast	Fibroblast	CL:0000057	Eye	UBERON:0000970	1.06084E-07	0.223105044	0.14095941	0.140242449	0.626308986	0.853120512	NA
TS_Salivary_Gland_salivary gland cell	Adult	Digestive system	TS_Salivary_Gland	salivary gland cell	Salivary gland cell	CL:0009005	Saliva-secreting gland	UBERON:0001044	1.12398E-08	0.262135046	0.120664207	0.140870021	0.626308986	0.851181419	NA
TS_Lung_bronchial vessel endothelial cell	Adult	Respiratory system	TS_Lung	bronchial vessel endothelial cell	Endothelial cell	CL:0000115	Lung	UBERON:0002048	1.12439E-08	0.261311628	0.121771218	0.14152536	0.626308986	0.849165732	NA
TS_Lymph_Node_classical monocyte	Adult	Lymphatic system	TS_Lymph_Node	classical monocyte	Monocyte	CL:0000576	Lymph node	UBERON:0000029	1.1275E-08	0.259253083	0.123616236	0.142309424	0.626308986	0.84676634	NA
TS_Pancreas_pancreatic pp cell	Adult	Digestive system	TS_Pancreas	pancreatic pp cell	Pancreatic PP cell	CL:0002275	Pancreas	UBERON:0001264	0.07493609	0.078307029	0.410332103	0.14259831	0.626308986	0.845885621	NA
TS_Thymus_cd8-positive, alpha-beta cytotoxic t cell	Adult	Lymphatic system	TS_Thymus	cd8-positive, alpha-beta cytotoxic t cell	T cell	CL:0000084	Thymus	UBERON:0002370	1.1155E-08	0.272345426	0.118450185	0.143036314	0.626308986	0.844553689	NA
AdultCervix_Endothelial cell	Adult	Reproductive system	AdultCervix	Endothelial cell	Endothelial cell	CL:0000115	Uterine cervix	UBERON:0000002	0.005380917	0.104121176	0.310701107	0.143349358	0.626308986	0.843604249	NA
TS_Lymph_Node_mast cell	Adult	Lymphatic system	TS_Lymph_Node	mast cell	Mast cell	CL:0000097	Lymph node	UBERON:0000029	1.62269E-05	0.169047667	0.191881919	0.143646466	0.626308986	0.842705054	NA
TS_Bone_Marrow_hematopoietic stem cell	Adult	Lymphatic system	TS_Bone_Marrow	hematopoietic stem cell	Hematopoietic stem cell	CL:0000037	Bone marrow	UBERON:0002371	1.04797E-07	0.237803051	0.136531365	0.143750623	0.626308986	0.842390263	NA
TS_Lung_capillary aerocyte	Adult	Respiratory system	TS_Lung	capillary aerocyte	Alveolar capillary type 1 endothelial cell	CL:4028002	Lung	UBERON:0002048	4.02091E-05	0.163860135	0.199261993	0.144379129	0.627031155	0.840495561	NA
TS_Mammary_basal cell	Adult	Endocrine system	TS_Mammary	basal cell	Basal cell	CL:0000646	Mammary gland	UBERON:0001911	1.60344E-05	0.17567618	0.190405904	0.147102441	0.636817276	0.832380121	NA
TS_Eye_conjunctival epithelial cell	Adult	Sensory system	TS_Eye	conjunctival epithelial cell	Epithelial cell	CL:0000066	Eye	UBERON:0000970	3.58089E-09	0.300918022	0.112546125	0.148517969	0.640802309	0.828220999	NA
TS_Lung_alveolar fibroblast	Adult	Respiratory system	TS_Lung	alveolar fibroblast	Fibroblast	CL:0000057	Lung	UBERON:0002048	0.002237565	0.123183298	0.27601476	0.148968802	0.640802309	0.826904673	NA
TS_Bone_Marrow_nk cell	Adult	Lymphatic system	TS_Bone_Marrow	nk cell	Natural killer cell	CL:0000623	Bone marrow	UBERON:0002371	0.002235773	0.12561238	0.275276753	0.150917548	0.641609929	0.82129026	NA
TS_Lymph_Node_type i nk t cell	Adult	Lymphatic system	TS_Lymph_Node	type i nk t cell	Natural killer cell	CL:0000623	Lymph node	UBERON:0000029	8.67536E-07	0.22434017	0.154243542	0.151001163	0.641609929	0.821019709	NA
TS_Uterus_pericyte cell	Adult	Reproductive system	TS_Uterus	pericyte cell	Pericyte cell	CL:0000669	Uterus	UBERON:0000995	1.5979E-05	0.182922257	0.189298893	0.151081745	0.641609929	0.820788008	NA
Fetal_HCA_Adrenal_CSH1_CSH2 positive cells	Fetal	Endocrine system	Fetal_HCA_Adrenal	CSH1_CSH2 positive cells	Trophoblast cell	CL:0000351	Adrenal gland	UBERON:0002369	0.019076371	0.098768961	0.351660517	0.151438622	0.641609929	0.819753535	NA
Fetal_HCA_Stomach_Mesothelial cells	Fetal	Digestive system	Fetal_HCA_Stomach	Mesothelial cells	Mesothelial cell	CL:0000077	Stomach	UBERON:0000945	0.021711358	0.097410322	0.356826568	0.151524116	0.641609929	0.819518241	NA
TS_Trachea_goblet cell	Adult	Respiratory system	TS_Trachea	goblet cell	Goblet cell	CL:0000160	Trachea	UBERON:0003126	1.59764E-05	0.188315643	0.188191882	0.153804805	0.649238351	0.813030097	NA
TS_Trachea_endothelial cell	Adult	Respiratory system	TS_Trachea	endothelial cell	Endothelial cell	CL:0000115	Trachea	UBERON:0003126	8.50558E-07	0.236732608	0.151291513	0.15505916	0.65231711	0.809502572	NA
TS_Liver_endothelial cell of hepatic sinusoid	Adult	Digestive system	TS_Liver	endothelial cell of hepatic sinusoid	Endothelial cell	CL:0000115	Liver	UBERON:0002107	3.01041E-07	0.250524855	0.143911439	0.155849958	0.65231711	0.807293311	NA
VentoTormo_2018_decidua_decidual natural killer cell (dNK3)	Maternal-Fetal	Reproductive system	VentoTormo_2018_decidua	decidual natural killer cell (dNK3)	Natural killer cell	CL:0000623	Decidua	UBERON:0002450	0.001442136	0.136568077	0.266051661	0.155978409	0.65231711	0.806935615	NA
TS_Fat_macrophage	Adult	Integumentary system	TS_Fat	macrophage	Macrophage	CL:0000235	Perirenal fat	UBERON:0005406	3.45591E-08	0.286549382	0.126937269	0.156931361	0.653587313	0.804340629	NA
TS_Blood_hematopoietic stem cell	Adult	Lymphatic system	TS_Blood	hematopoietic stem cell	Hematopoietic stem cell	CL:0000037	Blood	UBERON:0000178	1.05616E-07	0.260241185	0.140221402	0.157302646	0.653587313	0.803263972	NA
TS_Thymus_cd8-positive, alpha-beta t cell	Adult	Lymphatic system	TS_Thymus	cd8-positive, alpha-beta t cell	T cell	CL:0000084	Thymus	UBERON:0002370	0.002233298	0.134505292	0.273800738	0.158414365	0.653587313	0.80020544	NA
TS_Spleen_cd8-positive, alpha-beta memory t cell	Adult	Lymphatic system	TS_Spleen	cd8-positive, alpha-beta memory t cell	T cell	CL:0000084	Spleen	UBERON:0002106	6.30743E-06	0.204495802	0.180442804	0.15865249	0.653587313	0.799553106	NA
AdultStomach_Smooth muscle cell	Adult	Digestive system	AdultStomach	Smooth muscle cell	Smooth muscle cell	CL:0000192	Stomach	UBERON:0000945	0.031507682	0.100333455	0.367896679	0.158693894	0.653587313	0.799439784	NA
TS_Bone_Marrow_granulocyte	Adult	Lymphatic system	TS_Bone_Marrow	granulocyte	Granulocyte	CL:0000094	Bone marrow	UBERON:0002371	3.52809E-09	0.340606759	0.108856089	0.159237153	0.653837399	0.797955597	NA
TS_Vasculature_artery endothelial cell	Adult	Cardiovascular system	TS_Vasculature	artery endothelial cell	Endothelial cell	CL:0000115	Vasculature	UBERON:0002049	3.02652E-07	0.259170742	0.144649446	0.160591605	0.655271211	0.794277162	NA
TS_Trachea_secretory cell	Adult	Respiratory system	TS_Trachea	secretory cell	Endocrine cell	CL:0000163	Trachea	UBERON:0003126	9.55254E-05	0.182263522	0.206642066	0.1611639	0.655271211	0.792732221	NA
TS_Thymus_cd4-positive helper t cell	Adult	Lymphatic system	TS_Thymus	cd4-positive helper t cell	T cell	CL:0000084	Thymus	UBERON:0002370	2.36004E-06	0.231874443	0.162730627	0.161392592	0.655271211	0.792116403	NA
TS_Tongue_pericyte cell	Adult	Digestive system	TS_Tongue	pericyte cell	Pericyte cell	CL:0000669	Tongue	UBERON:0001723	1.62496E-05	0.196099641	0.192619926	0.161520727	0.655271211	0.79177174	NA
AdultStomach_Stromal cell	Adult	Digestive system	AdultStomach	Stromal cell	Stromal cell	CL:0000499	Stomach	UBERON:0000945	0.031507682	0.103915322	0.367896679	0.163018203	0.659372135	0.787763858	NA
TS_Kidney_macrophage	Adult	Urinary system	TS_Kidney	macrophage	Macrophage	CL:0000235	Kidney	UBERON:0002113	1.97532E-06	0.245913716	0.157933579	0.164997764	0.664710731	0.782521942	NA
TS_Lymph_Node_intermediate monocyte	Adult	Lymphatic system	TS_Lymph_Node	intermediate monocyte	Monocyte	CL:0000576	Lymph node	UBERON:0000029	3.49318E-05	0.208077231	0.190627306	0.1653192	0.664710731	0.781676705	NA
TS_Spleen_erythrocyte	Adult	Lymphatic system	TS_Spleen	erythrocyte	Erythrocyte	CL:0000232	Spleen	UBERON:0002106	4.02525E-05	0.195932257	0.200738007	0.166596144	0.666965685	0.778335055	NA
TS_Thymus_endothelial cell of lymphatic vessel	Adult	Lymphatic system	TS_Thymus	endothelial cell of lymphatic vessel	Endothelial cell	CL:0000115	Thymus	UBERON:0002370	1.0407E-07	0.292683845	0.135055351	0.167234605	0.666965685	0.77667385	NA
TS_Kidney_endothelial cell	Adult	Urinary system	TS_Kidney	endothelial cell	Endothelial cell	CL:0000115	Kidney	UBERON:0002113	1.37579E-05	0.211906562	0.186715867	0.167356703	0.666965685	0.776356888	NA
TS_Lymph_Node_stromal cell	Adult	Lymphatic system	TS_Lymph_Node	stromal cell	Stromal cell	CL:0000499	Lymph node	UBERON:0000029	3.06597E-07	0.274321629	0.146863469	0.169680476	0.674243532	0.770368127	NA
TS_Spleen_naive thymus-derived cd4-positive, alpha-beta t cell	Adult	Lymphatic system	TS_Spleen	naive thymus-derived cd4-positive, alpha-beta t cell	T cell	CL:0000084	Spleen	UBERON:0002106	6.30743E-06	0.224216657	0.180442804	0.170227572	0.674436835	0.768970095	NA
Fetal_HCA_Intestine_Intestinal epithelial cells	Fetal	Digestive system	Fetal_HCA_Intestine	Intestinal epithelial cells	Epithelial cell	CL:0000066	Intestine	UBERON:0000160	0.018419974	0.11585488	0.350553506	0.170724601	0.674436835	0.767703893	NA
TS_Tongue_capillary endothelial cell	Adult	Digestive system	TS_Tongue	capillary endothelial cell	Capillary endothelial cell	CL:0002144	Tongue	UBERON:0001723	3.07256E-07	0.27576261	0.148339483	0.171662695	0.67617137	0.765324074	NA
TS_Pancreas_endothelial cell	Adult	Digestive system	TS_Pancreas	endothelial cell	Endothelial cell	CL:0000115	Pancreas	UBERON:0001264	1.02571E-08	0.357445652	0.115867159	0.173288864	0.67962188	0.761229346	NA
Lake_2017_FrontalCortex_Ex6a	Adult	Nervous system	Lake_2017_FrontalCortex	Ex6a	Excitatory neuron	CL:0008030	Frontal cortex	UBERON:0001870	0.017878915	0.119189722	0.349077491	0.173894157	0.67962188	0.759715009	NA
TS_Lung_bronchial smooth muscle cell	Adult	Respiratory system	TS_Lung	bronchial smooth muscle cell	Smooth muscle cell	CL:0000192	Lung	UBERON:0002048	0.000500269	0.176376085	0.236162362	0.174043389	0.67962188	0.759342468	NA
TS_Thymus_macrophage	Adult	Lymphatic system	TS_Thymus	macrophage	Macrophage	CL:0000235	Thymus	UBERON:0002370	1.0407E-07	0.311169574	0.135055351	0.17522321	0.680949361	0.756408369	NA
TS_Large_Intestine_mast cell	Adult	Digestive system	TS_Large_Intestine	mast cell	Mast cell	CL:0000097	Large intestine	UBERON:0000059	8.59748E-07	0.275433242	0.152767528	0.175388433	0.680949361	0.755999051	NA
Fetal_HCA_Cerebellum_10k_Granule neurons	Fetal	Nervous system	Fetal_HCA_Cerebellum_10k	Granule neurons	Neuron	CL:0000540	Cerebellum	UBERON:0002037	0.005436841	0.135658077	0.311439114	0.175932944	0.681111827	0.754652829	NA
VentoTormo_2018_decidua_decidual perivascular cell (dP1)	Maternal-Fetal	Reproductive system	VentoTormo_2018_decidua	decidual perivascular cell (dP1)	Pericyte cell	CL:0000669	Decidua	UBERON:0002450	0.000136932	0.198567194	0.215867159	0.177874154	0.684686063	0.749887154	NA
TS_Bladder_t cell	Adult	Urinary system	TS_Bladder	t cell	T cell	CL:0000084	Bladder organ	UBERON:0018707	4.015E-05	0.218699758	0.196309963	0.178090816	0.684686063	0.749358475	NA
TS_Large_Intestine_immature enterocyte	Adult	Digestive system	TS_Large_Intestine	immature enterocyte	Enterocyte	CL:0000584	Large intestine	UBERON:0000059	3.45708E-08	0.335995619	0.12804428	0.178372089	0.684686063	0.748673102	NA
TS_Small_Intestine_b cell	Adult	Digestive system	TS_Small_Intestine	b cell	B cell	CL:0000236	Small intestine	UBERON:0002108	3.04798E-07	0.296759763	0.146125461	0.179446175	0.686600157	0.746065794	NA
Fetal_HCA_Cerebrum_10k_Excitatory neurons	Fetal	Nervous system	Fetal_HCA_Cerebrum_10k	Excitatory neurons	Excitatory neuron	CL:0008030	Cerebral hemisphere	UBERON:0001869	0.005466366	0.139898678	0.312546125	0.180576389	0.686600157	0.743339036	NA
TS_Heart_fibroblast of cardiac tissue	Adult	Cardiovascular system	TS_Heart	fibroblast of cardiac tissue	Fibroblast	CL:0000057	Heart	UBERON:0000948	0.015739094	0.128782538	0.340221402	0.180857313	0.686600157	0.742663927	NA
AdultKidney_Epithelial cell	Adult	Urinary system	AdultKidney	Epithelial cell	Epithelial cell	CL:0000066	Kidney	UBERON:0002113	0.017771887	0.125818235	0.348339483	0.180897606	0.686600157	0.742567181	NA
TS_Muscle_t cell	Adult	Muscular system	TS_Muscle	t cell	T cell	CL:0000084	Muscle organ	UBERON:0001630	0.000224011	0.200296371	0.221402214	0.182516472	0.690809554	0.738697934	NA
TS_Thymus_naive regulatory t cell	Adult	Lymphatic system	TS_Thymus	naive regulatory t cell	T cell	CL:0000084	Thymus	UBERON:0002370	3.03865E-07	0.30639375	0.145387454	0.183138382	0.691232611	0.737220627	NA
Segerstolpe_2016_PancreaticIslets_pancreatic D cell	Adult	Digestive system	Segerstolpe_2016_PancreaticIslets	pancreatic D cell	Pancreatic D cell	CL:0000173	Pancreas	UBERON:0001264	0.033801776	0.119889627	0.373431734	0.183837172	0.691278103	0.73556667	NA
TS_Prostate_hillock-club cell of prostate epithelium	Adult	Reproductive system	TS_Prostate	hillock-club cell of prostate epithelium	Club cell	CL:0000158	Prostate gland	UBERON:0002367	2.34688E-06	0.281526534	0.159409594	0.184170771	0.691278103	0.734779293	NA
Fetal_HCA_Placenta_PAEP_MECOM positive cells	Fetal	Reproductive system	Fetal_HCA_Placenta	PAEP_MECOM positive cells	Epithelial cell	CL:0000066	Placenta	UBERON:0001987	0.02829674	0.124788962	0.366789668	0.186934482	0.698919199	0.728310581	NA
Voigt_2019_retina_2_Vein cell 1	Adult	Sensory system	Voigt_2019_retina_2	Vein cell 1	Endothelial cell	CL:0000115	Retina	UBERON:0000966	0.03477076	0.121989342	0.37601476	0.187238132	0.698919199	0.727605699	NA
TS_Spleen_plasma cell	Adult	Lymphatic system	TS_Spleen	plasma cell	Plasma cell	CL:0000786	Spleen	UBERON:0002106	1.05005E-07	0.335542739	0.137269373	0.18782315	0.699176837	0.72625088	NA
TS_Spleen_mature nk t cell	Adult	Lymphatic system	TS_Spleen	mature nk t cell	T cell	CL:0000084	Spleen	UBERON:0002106	9.66196E-06	0.220387765	0.210332103	0.188729797	0.699984067	0.724159527	NA
AdultRectum_Enterocyte	Adult	Digestive system	AdultRectum	Enterocyte	Enterocyte	CL:0000584	Rectum	UBERON:0001052	0.040399565	0.121783487	0.381549815	0.189073187	0.699984067	0.723370055	NA
TS_Thymus_naive b cell	Adult	Lymphatic system	TS_Thymus	naive b cell	B cell	CL:0000236	Thymus	UBERON:0002370	0.002233298	0.171147383	0.273800738	0.190280143	0.702130826	0.720606531	NA
TS_Tongue_endothelial cell of lymphatic vessel	Adult	Digestive system	TS_Tongue	endothelial cell of lymphatic vessel	Endothelial cell	CL:0000115	Tongue	UBERON:0001723	2.38285E-06	0.283008686	0.166051661	0.190689405	0.702130826	0.719673436	NA
TS_Small_Intestine_gut endothelial cell	Adult	Digestive system	TS_Small_Intestine	gut endothelial cell	Endothelial cell	CL:0000115	Small intestine	UBERON:0002108	2.3661E-06	0.291284034	0.163837638	0.192913556	0.708395306	0.714637254	NA
TS_Kidney_nk cell	Adult	Urinary system	TS_Kidney	nk cell	Natural killer cell	CL:0000623	Kidney	UBERON:0002113	0.014598138	0.142698299	0.337269373	0.194142402	0.709097423	0.7118796	NA
AdultUterus_Smooth muscle cell	Adult	Reproductive system	AdultUterus	Smooth muscle cell	Smooth muscle cell	CL:0000192	Uterus	UBERON:0000995	0.036009841	0.127753266	0.377490775	0.194439363	0.709097423	0.71121581	NA
AdultMuscle_Smooth muscle cell	Adult	Muscular system	AdultMuscle	Smooth muscle cell	Smooth muscle cell	CL:0000192	Muscle organ	UBERON:0001630	0.032552306	0.129605956	0.372693727	0.194674717	0.709097423	0.710690448	NA
TS_Large_Intestine_b cell	Adult	Digestive system	TS_Large_Intestine	b cell	B cell	CL:0000236	Large intestine	UBERON:0000059	2.35838E-06	0.302235491	0.161254613	0.195986514	0.711961734	0.707773811	NA
AdultFallopiantube_Smooth muscle cell	Adult	Reproductive system	AdultFallopiantube	Smooth muscle cell	Smooth muscle cell	CL:0000192	Fallopian tube	UBERON:0003889	0.042782426	0.128947222	0.382287823	0.197669487	0.714253676	0.704060366	NA
TS_Muscle_macrophage	Adult	Muscular system	TS_Muscle	macrophage	Macrophage	CL:0000235	Muscle organ	UBERON:0001630	3.07024E-07	0.333978246	0.147601476	0.197671681	0.714253676	0.704055544	NA
TS_Vasculature_macrophage	Adult	Cardiovascular system	TS_Vasculature	macrophage	Macrophage	CL:0000235	Vasculature	UBERON:0002049	2.35264E-06	0.310016789	0.160147601	0.198732211	0.715682428	0.701731737	NA
FetalEyes_Erythroid cell	Fetal	Sensory system	FetalEyes	Erythroid cell	Erythroid lineage cell	CL:0000764	Eye	UBERON:0000970	0.039803665	0.13071757	0.380811808	0.199123451	0.715682428	0.700877591	NA
Fetal_HCA_Liver_Hematopoietic stem cells	Fetal	Digestive system	Fetal_HCA_Liver	Hematopoietic stem cells	Hematopoietic stem cell	CL:0000037	Liver	UBERON:0002107	0.019085228	0.143110008	0.352767528	0.201235896	0.721361479	0.696294548	NA
Fetal_HCA_Cerebrum_10k_Astrocytes	Fetal	Nervous system	Fetal_HCA_Cerebrum_10k	Astrocytes	Astrocyte	CL:0000127	Cerebral hemisphere	UBERON:0001869	0.005466366	0.162625009	0.312546125	0.202259715	0.723118505	0.69409061	NA
TS_Trachea_serous cell of epithelium of trachea	Adult	Respiratory system	TS_Trachea	serous cell of epithelium of trachea	Serous cell of epithelium of trachea	CL:1000330	Trachea	UBERON:0003126	0.004443206	0.173288269	0.295571956	0.203424124	0.723761672	0.691597545	NA
Fetal_HCA_Lung_Bronchiolar and alveolar epithelial cells	Fetal	Respiratory system	Fetal_HCA_Lung	Bronchiolar and alveolar epithelial cells	Epithelial cell	CL:0000066	Lung	UBERON:0002048	0.019217863	0.144962698	0.353874539	0.203660175	0.723761672	0.691093887	NA
Lake_2017_CerebellarHemisphere_OPC	Adult	Nervous system	Lake_2017_CerebellarHemisphere	OPC	Oligodendrocyte precursor cell	CL:0002453	Cerebellar hemisphere	UBERON:0002245	0.03179885	0.138869406	0.370479705	0.204104547	0.723761672	0.690147321	NA
Enge_2017_Pancreas_pancreatic ductal cell	Adult	Digestive system	Enge_2017_Pancreas	pancreatic ductal cell	Pancreatic ductal cell	CL:0002079	Pancreas	UBERON:0001264	0.000676087	0.20811884	0.24797048	0.204576177	0.723761672	0.689144941	NA
TS_Bone_Marrow_nampt neutrophil	Adult	Lymphatic system	TS_Bone_Marrow	nampt neutrophil	Neutrophil	CL:0000775	Bone marrow	UBERON:0002371	4.01881E-05	0.263370172	0.197416974	0.2057201	0.725669727	0.686723272	NA
TS_Lung_lung ciliated cell	Adult	Respiratory system	TS_Lung	lung ciliated cell	Ciliated cell	CL:0000064	Lung	UBERON:0002048	1.61835E-05	0.272839477	0.191143911	0.206186602	0.725669727	0.68573956	NA
TS_Lung_pericyte cell	Adult	Respiratory system	TS_Lung	pericyte cell	Pericyte cell	CL:0000669	Lung	UBERON:0002048	0.000500269	0.222734506	0.236162362	0.207513497	0.727515159	0.68295365	NA
AdultKidney_Endothelial cell	Adult	Urinary system	AdultKidney	Endothelial cell	Endothelial cell	CL:0000115	Kidney	UBERON:0002113	0.031611385	0.142657128	0.369327694	0.207784772	0.727515159	0.682386283	NA
Voigt_2019_retina_2_White blood cell 2	Adult	Sensory system	Voigt_2019_retina_2	White blood cell 2	Leukocyte	CL:0000738	Retina	UBERON:0000966	0.03477076	0.141133805	0.37601476	0.208886419	0.729487364	0.680089796	NA
TS_Heart_cardiac endothelial cell	Adult	Cardiovascular system	TS_Heart	cardiac endothelial cell	Endothelial cell	CL:0000115	Heart	UBERON:0000948	6.19327E-06	0.308575808	0.173431734	0.210201238	0.731100644	0.677364731	NA
Fetal_HCA_Placenta_Megakaryocytes	Fetal	Reproductive system	Fetal_HCA_Placenta	Megakaryocytes	Megakaryocyte	CL:0000556	Placenta	UBERON:0001987	0.048749596	0.137798963	0.388929889	0.210427492	0.731100644	0.676897522	NA
TS_Blood_platelet	Adult	Lymphatic system	TS_Blood	platelet	Platelet	CL:0000233	Blood	UBERON:0000178	6.33516E-06	0.292230965	0.184501845	0.211371769	0.73250319	0.674953018	NA
TS_Lung_vascular associated smooth muscle cell	Adult	Respiratory system	TS_Lung	vascular associated smooth muscle cell	Smooth muscle cell	CL:0000192	Lung	UBERON:0002048	0.000223739	0.246901817	0.220664207	0.213019495	0.736330141	0.67158065	NA
Fetal_HCA_Lung_Mesothelial cells	Fetal	Respiratory system	Fetal_HCA_Lung	Mesothelial cells	Mesothelial cell	CL:0000077	Lung	UBERON:0002048	0.019217863	0.156408205	0.353874539	0.215534014	0.743043393	0.666484183	NA
Fetal_HCA_Stomach_ENS glia	Fetal	Digestive system	Fetal_HCA_Stomach	ENS glia	ENS glia	CL:0000125	Stomach	UBERON:0000945	0.038458692	0.146321337	0.380073801	0.216297501	0.743043393	0.664948499	NA
Fetal_HCA_Kidney_Erythroblasts	Fetal	Urinary system	Fetal_HCA_Kidney	Erythroblasts	Erythroblast	CL:0000765	Kidney	UBERON:0002113	0.033969836	0.148626907	0.374907749	0.216610809	0.743043393	0.664319877	NA
Fetal_HCA_Eye_Photoreceptor cells	Fetal	Sensory system	Fetal_HCA_Eye	Photoreceptor cells	Photoreceptor cell	CL:0000210	Eye	UBERON:0000970	0.032402004	0.151055989	0.371217712	0.217629748	0.743043393	0.662281742	NA
TS_Prostate_cd8b-positive nk t cell	Adult	Reproductive system	TS_Prostate	cd8b-positive nk t cell	Natural killer cell	CL:0000623	Prostate gland	UBERON:0002367	0.001073708	0.223063873	0.252767528	0.218518128	0.743043393	0.660512529	NA
TS_Spleen_naive thymus-derived cd8-positive, alpha-beta t cell	Adult	Lymphatic system	TS_Spleen	naive thymus-derived cd8-positive, alpha-beta t cell	T cell	CL:0000084	Spleen	UBERON:0002106	0.000500599	0.237803051	0.237638376	0.218885561	0.743043393	0.659782887	NA
Segerstolpe_2016_PancreaticIslets_beta cell	Adult	Digestive system	Segerstolpe_2016_PancreaticIslets	beta cell	Type B pancreatic cell	CL:0000169	Pancreas	UBERON:0001264	0.01075019	0.169871085	0.332841328	0.218968841	0.743043393	0.659617679	NA
TS_Lung_vein endothelial cell	Adult	Respiratory system	TS_Lung	vein endothelial cell	Endothelial cell	CL:0000115	Lung	UBERON:0002048	4.02091E-05	0.284861376	0.199261993	0.219605962	0.743043393	0.658355874	NA
TS_Tongue_vein endothelial cell	Adult	Digestive system	TS_Tongue	vein endothelial cell	Endothelial cell	CL:0000115	Tongue	UBERON:0001723	6.3256E-06	0.311498941	0.183394834	0.220652653	0.743043393	0.656290846	NA
TS_Salivary_Gland_cd4-positive helper t cell	Adult	Digestive system	TS_Salivary_Gland	cd4-positive helper t cell	T cell	CL:0000084	Saliva-secreting gland	UBERON:0001044	0.000501029	0.239573399	0.238745387	0.220852261	0.743043393	0.65589815	NA
Madissoon_2020_spleen_CD34_progenitor	Adult	Lymphatic system	Madissoon_2020_spleen	CD34_progenitor	Hematopoietic stem cell	CL:0000037	Spleen	UBERON:0002106	0.032447772	0.154061464	0.37195572	0.221158311	0.743043393	0.655296735	NA
TS_Bladder_macrophage	Adult	Urinary system	TS_Bladder	macrophage	Macrophage	CL:0000235	Bladder organ	UBERON:0018707	8.63497E-07	0.376054893	0.153505535	0.222364748	0.743043393	0.652934062	NA
TS_Blood_neutrophil	Adult	Lymphatic system	TS_Blood	neutrophil	Neutrophil	CL:0000775	Blood	UBERON:0000178	2.38719E-06	0.346205999	0.166789668	0.222413447	0.743043393	0.65283896	NA
TS_Large_Intestine_intestinal crypt stem cell	Adult	Digestive system	TS_Large_Intestine	intestinal crypt stem cell	Intestinal crypt stem cell	CL:0002250	Large intestine	UBERON:0000059	4.00235E-05	0.297006788	0.194833948	0.222765281	0.743043393	0.652152495	NA
AdultTrachea_Smooth muscle cell	Adult	Respiratory system	AdultTrachea	Smooth muscle cell	Smooth muscle cell	CL:0000192	Trachea	UBERON:0003126	0.055086159	0.147062413	0.395571956	0.223638618	0.743043393	0.650453199	NA
TS_Liver_intrahepatic cholangiocyte	Adult	Digestive system	TS_Liver	intrahepatic cholangiocyte	Intrahepatic cholangiocyte	CL:0002538	Liver	UBERON:0002107	0.000498015	0.251183589	0.231734317	0.223735575	0.743043393	0.650264955	NA
TS_Mammary_pericyte cell	Adult	Endocrine system	TS_Mammary	pericyte cell	Pericyte cell	CL:0000669	Mammary gland	UBERON:0001911	0.000222307	0.267734286	0.218450185	0.22452759	0.743850574	0.648730285	NA
TS_Lymph_Node_cd1c-positive myeloid dendritic cell	Adult	Lymphatic system	TS_Lymph_Node	cd1c-positive myeloid dendritic cell	Dendritic cell	CL:0000451	Lymph node	UBERON:0000029	2.37868E-06	0.355675304	0.165313653	0.225410756	0.74495506	0.647025364	NA
TS_Tongue_endothelial cell of artery	Adult	Digestive system	TS_Tongue	endothelial cell of artery	Endothelial cell	CL:0000115	Tongue	UBERON:0001723	6.3256E-06	0.324550112	0.183394834	0.227454577	0.746088945	0.643105319	NA
FetalKidney_Primordial germ cell	Fetal	Urinary system	FetalKidney	Primordial germ cell	Primordial germ cell	CL:0000670	Kidney	UBERON:0002113	0.060473214	0.147103584	0.404797048	0.227528726	0.746088945	0.642963766	NA
TS_Fat_neutrophil	Adult	Integumentary system	TS_Fat	neutrophil	Neutrophil	CL:0000775	Perirenal fat	UBERON:0005406	0.000498809	0.256247609	0.233210332	0.228127788	0.746088945	0.64182181	NA
TS_Prostate_myeloid cell	Adult	Reproductive system	TS_Prostate	myeloid cell	Myeloid cell	CL:0000763	Prostate gland	UBERON:0002367	6.23849E-06	0.340771442	0.175645756	0.228396686	0.746088945	0.641310203	NA
TS_Liver_macrophage	Adult	Digestive system	TS_Liver	macrophage	Macrophage	CL:0000235	Liver	UBERON:0002107	6.23167E-06	0.343488721	0.174907749	0.229026398	0.746088945	0.640114458	NA
Fetal_HCA_Muscle_Satellite cells	Fetal	Muscular system	Fetal_HCA_Muscle	Satellite cells	Skeletal muscle satellite cell	CL:0000594	Muscle organ	UBERON:0001630	0.045393134	0.156943426	0.38302583	0.229123538	0.746088945	0.639930294	NA
TS_Lung_respiratory goblet cell	Adult	Respiratory system	TS_Lung	respiratory goblet cell	Goblet cell	CL:0000160	Lung	UBERON:0002048	6.29853E-06	0.338054164	0.178597786	0.229860538	0.746088945	0.638535581	NA
TS_Large_Intestine_gut endothelial cell	Adult	Digestive system	TS_Large_Intestine	gut endothelial cell	Endothelial cell	CL:0000115	Large intestine	UBERON:0000059	6.26455E-06	0.341471347	0.177121771	0.230158804	0.746088945	0.637972408	NA
TS_Spleen_regulatory t cell	Adult	Lymphatic system	TS_Spleen	regulatory t cell	T cell	CL:0000084	Spleen	UBERON:0002106	0.001078725	0.236979633	0.257564576	0.231714802	0.74934023	0.635046223	NA
TS_Trachea_fibroblast	Adult	Respiratory system	TS_Trachea	fibroblast	Fibroblast	CL:0000057	Trachea	UBERON:0003126	6.20981E-06	0.35456369	0.174169742	0.233714715	0.752815625	0.631313942	NA
Fetal_HCA_Kidney_Stromal cells	Fetal	Urinary system	Fetal_HCA_Kidney	Stromal cells	Stromal cell	CL:0000499	Kidney	UBERON:0002113	0.019256725	0.174152857	0.35498155	0.233900648	0.752815625	0.630968575	NA
TS_Vasculature_lymphatic endothelial cells	Adult	Cardiovascular system	TS_Vasculature	lymphatic endothelial cells	Endothelial cell	CL:0000115	Vasculature	UBERON:0002049	0.00022277	0.283790933	0.219188192	0.234964355	0.75444716	0.628998016	NA
TS_Kidney_kidney epithelial cell	Adult	Urinary system	TS_Kidney	kidney epithelial cell	Epithelial cell	CL:0000066	Kidney	UBERON:0002113	3.45276E-05	0.325579384	0.194095941	0.23770613	0.758298266	0.623959618	NA
VentoTormo_2018_decidua_decidual stromal cell (dS1)	Maternal-Fetal	Reproductive system	VentoTormo_2018_decidua	decidual stromal cell (dS1)	Stromal cell	CL:0000499	Decidua	UBERON:0002450	0.000136932	0.292807357	0.215867159	0.237744391	0.758298266	0.623889721	NA
TS_Large_Intestine_transit amplifying cell of large intestine	Adult	Digestive system	TS_Large_Intestine	transit amplifying cell of large intestine	Transit amplifying cell	CL:0009010	Large intestine	UBERON:0000059	2.35838E-06	0.392193882	0.161254613	0.23784263	0.758298266	0.623710302	NA
Fetal_HCA_Intestine_Erythroblasts	Fetal	Digestive system	Fetal_HCA_Intestine	Erythroblasts	Erythroblast	CL:0000765	Intestine	UBERON:0000160	0.055367246	0.161389882	0.398892989	0.240963839	0.765008362	0.618048126	NA
Fetal_HCA_Adrenal_Vascular endothelial cells	Fetal	Endocrine system	Fetal_HCA_Adrenal	Vascular endothelial cells	Endothelial cell	CL:0000115	Adrenal gland	UBERON:0002369	0.019076371	0.183828016	0.351660517	0.241697755	0.765008362	0.616727384	NA
TS_Thymus_thymocyte	Adult	Lymphatic system	TS_Thymus	thymocyte	Thymocyte	CL:0000893	Thymus	UBERON:0002370	0.002233298	0.236814949	0.273800738	0.24223168	0.765008362	0.615769059	NA
TS_Heart_hepatocyte	Adult	Cardiovascular system	TS_Heart	hepatocyte	Hepatocyte	CL:0000182	Heart	UBERON:0000948	1.59391E-05	0.346741221	0.187453875	0.242663414	0.765008362	0.614995697	NA
TS_Salivary_Gland_monocyte	Adult	Digestive system	TS_Salivary_Gland	monocyte	Monocyte	CL:0000576	Saliva-secreting gland	UBERON:0001044	6.30469E-06	0.362839038	0.179335793	0.242860292	0.765008362	0.614643487	NA
AdultUterus_Endothelial cell	Adult	Reproductive system	AdultUterus	Endothelial cell	Endothelial cell	CL:0000115	Uterus	UBERON:0000995	0.036009841	0.172835389	0.377490775	0.243334763	0.765008362	0.613795843	NA
Lake_2017_VisualCortex_In8	Adult	Nervous system	Lake_2017_VisualCortex	In8	Inhibitory neuron	CL:0008029	Visual cortex	UBERON:0000411	0.017945902	0.187574567	0.349815498	0.244351102	0.766425331	0.611985698	NA
van_Zyl_eye_2020_SchwannCell-my	Adult	Sensory system	van_Zyl_eye_2020	SchwannCell-my	Schwann cell	CL:0002573	Eye	UBERON:0000970	0.064406311	0.162830863	0.405904059	0.245649277	0.768717716	0.609684509	NA
AdultBladder_Epithelial cell (intermediated)	Adult	Urinary system	AdultBladder	Epithelial cell (intermediated)	Epithelial cell	CL:0000066	Bladder organ	UBERON:0018707	0.067069215	0.164436528	0.408856089	0.248728872	0.77656134	0.6042738	NA
TS_Muscle_tendon cell	Adult	Muscular system	TS_Muscle	tendon cell	Cell of skeletal muscle	CL:0000188	Muscle organ	UBERON:0001630	4.02859E-05	0.336077961	0.201476015	0.250025062	0.776682519	0.602016457	NA
TS_Uterus_endothelial cell of lymphatic vessel	Adult	Reproductive system	TS_Uterus	endothelial cell of lymphatic vessel	Endothelial cell	CL:0000115	Uterus	UBERON:0000995	1.5979E-05	0.359627709	0.189298893	0.251008128	0.776682519	0.600312215	NA
TS_Trachea_smooth muscle cell	Adult	Respiratory system	TS_Trachea	smooth muscle cell	Smooth muscle cell	CL:0000192	Trachea	UBERON:0003126	0.000496132	0.297912547	0.228782288	0.251223015	0.776682519	0.599940576	NA
TS_Lymph_Node_non-classical monocyte	Adult	Lymphatic system	TS_Lymph_Node	non-classical monocyte	Monocyte	CL:0000576	Lymph node	UBERON:0000029	9.67782E-05	0.32257391	0.211439114	0.251350924	0.776682519	0.599719515	NA
Fetal_HCA_Eye_Astrocytes	Fetal	Sensory system	Fetal_HCA_Eye	Astrocytes	Astrocyte	CL:0000127	Eye	UBERON:0000970	0.055013262	0.173658807	0.393357934	0.251633672	0.776682519	0.599231244	NA
TS_Mammary_t cell	Adult	Endocrine system	TS_Mammary	t cell	T cell	CL:0000084	Mammary gland	UBERON:0001911	0.0044525	0.231668588	0.29704797	0.252991399	0.777450776	0.596894243	NA
VentoTormo_2018_decidua_decidual perivascular cell (dP2)	Maternal-Fetal	Reproductive system	VentoTormo_2018_decidua	decidual perivascular cell (dP2)	Pericyte cell	CL:0000669	Decidua	UBERON:0002450	0.00293503	0.240067449	0.286715867	0.253030105	0.777450776	0.596827804	NA
AdultTrachea_Chondrocyte	Adult	Respiratory system	AdultTrachea	Chondrocyte	Chondrocyte	CL:0000138	Trachea	UBERON:0003126	0.055086159	0.175099788	0.395571956	0.254188614	0.779243376	0.594843907	NA
Enge_2017_Pancreas_acinar cell	Adult	Digestive system	Enge_2017_Pancreas	acinar cell	Acinar cell	CL:0000622	Pancreas	UBERON:0001264	0.005716969	0.223187385	0.315129151	0.257032551	0.785053343	0.590011874	NA
TS_Thymus_nk cell	Adult	Lymphatic system	TS_Thymus	nk cell	Natural killer cell	CL:0000623	Thymus	UBERON:0002370	0.00049899	0.301206219	0.234317343	0.257682453	0.785053343	0.588915153	NA
TS_Large_Intestine_plasma cell	Adult	Digestive system	TS_Large_Intestine	plasma cell	Plasma cell	CL:0000786	Large intestine	UBERON:0000059	9.61198E-05	0.338177676	0.208856089	0.257821947	0.785053343	0.588680117	NA
Madissoon_2020_spleen_T_CD8_activated	Adult	Lymphatic system	Madissoon_2020_spleen	T_CD8_activated	T cell	CL:0000084	Spleen	UBERON:0002106	0.055043861	0.181851813	0.394095941	0.260561658	0.790013532	0.584089491	NA
AdultLung_Endothelial cell (endothelial to mesenchymal transition)	Adult	Respiratory system	AdultLung	Endothelial cell (endothelial to mesenchymal transition)	Endothelial cell	CL:0000115	Lung	UBERON:0002048	0.037278858	0.189180232	0.37896679	0.260630086	0.790013532	0.583975453	NA
TS_Trachea_ciliated cell	Adult	Respiratory system	TS_Trachea	ciliated cell	Ciliated cell	CL:0000064	Trachea	UBERON:0003126	0.001070276	0.287002262	0.250553506	0.261200046	0.790013532	0.583026751	NA
TS_Trachea_mucus secreting cell	Adult	Respiratory system	TS_Trachea	mucus secreting cell	Mucus secreting cell	CL:0000319	Trachea	UBERON:0003126	0.027862098	0.202396087	0.359409594	0.263389748	0.794862158	0.579401133	NA
AdultLung_AT2 cell	Adult	Respiratory system	AdultLung	AT2 cell	Alveolar capillary type 2 endothelial cell	CL:4028003	Lung	UBERON:0002048	0.037278858	0.195273523	0.37896679	0.26667875	0.802999348	0.574011589	NA
TS_Heart_smooth muscle cell	Adult	Cardiovascular system	TS_Heart	smooth muscle cell	Smooth muscle cell	CL:0000192	Heart	UBERON:0000948	0.047260057	0.195108839	0.383763838	0.268948008	0.804852973	0.570331668	NA
Fetal_HCA_Spleen_Erythroblasts	Fetal	Lymphatic system	Fetal_HCA_Spleen	Erythroblasts	Erythroblast	CL:0000765	Spleen	UBERON:0002106	0.067200861	0.182881086	0.409594096	0.269029118	0.804852973	0.570200711	NA
AdultStomach_Fetal endocrine cell	Adult	Digestive system	AdultStomach	Fetal endocrine cell	Endocrine cell	CL:0000163	Stomach	UBERON:0000945	0.053620033	0.192268048	0.390774908	0.269615794	0.804852973	0.569254671	NA
TS_Muscle_endothelial cell of artery	Adult	Muscular system	TS_Muscle	endothelial cell of artery	Endothelial cell	CL:0000115	Muscle organ	UBERON:0001630	6.3157E-06	0.412285275	0.182287823	0.269670295	0.804852973	0.569166889	NA
TS_Lymph_Node_neutrophil	Adult	Lymphatic system	TS_Lymph_Node	neutrophil	Neutrophil	CL:0000775	Lymph node	UBERON:0000029	0.000224299	0.340771442	0.222140221	0.271077499	0.806288494	0.566906529	NA
TS_Lymph_Node_nk cell	Adult	Lymphatic system	TS_Lymph_Node	nk cell	Natural killer cell	CL:0000623	Lymph node	UBERON:0000029	0.000501481	0.317057011	0.239852399	0.271974475	0.806288494	0.565471853	NA
TS_Liver_endothelial cell	Adult	Digestive system	TS_Liver	endothelial cell	Endothelial cell	CL:0000115	Liver	UBERON:0002107	9.5886E-05	0.365968026	0.208118081	0.272277573	0.806288494	0.56498813	NA
Fetal_HCA_Stomach_Erythroblasts	Fetal	Digestive system	Fetal_HCA_Stomach	Erythroblasts	Erythroblast	CL:0000765	Stomach	UBERON:0000945	0.06500503	0.187204029	0.407380074	0.272531461	0.806288494	0.564583355	NA
TS_Eye_macrophage	Adult	Sensory system	TS_Eye	macrophage	Macrophage	CL:0000235	Eye	UBERON:0000970	4.04985E-05	0.386471128	0.202214022	0.277363833	0.818756253	0.556950169	NA
VentoTormo_2018_decidua_decidual natural killer cell (dNK2)	Maternal-Fetal	Reproductive system	VentoTormo_2018_decidua	decidual natural killer cell (dNK2)	Natural killer cell	CL:0000623	Decidua	UBERON:0002450	0.019561236	0.220346594	0.355719557	0.277954152	0.818756253	0.556026833	NA
Fetal_HCA_Stomach_Vascular endothelial cells	Fetal	Digestive system	Fetal_HCA_Stomach	Vascular endothelial cells	Epithelial cell	CL:0000066	Stomach	UBERON:0000945	0.06500503	0.194697131	0.407380074	0.28032708	0.823954867	0.552334947	NA
TS_Lung_neutrophil	Adult	Respiratory system	TS_Lung	neutrophil	Neutrophil	CL:0000775	Lung	UBERON:0002048	0.047395162	0.206142638	0.387453875	0.281731771	0.825549226	0.550164175	NA
Fetal_HCA_Intestine_Smooth muscle cells	Fetal	Digestive system	Fetal_HCA_Intestine	Smooth muscle cells	Smooth muscle cell	CL:0000192	Intestine	UBERON:0000160	0.055367246	0.200584568	0.398892989	0.282088038	0.825549226	0.54961533	NA
TS_Eye_epithelial cell of lacrimal sac	Adult	Sensory system	TS_Eye	epithelial cell of lacrimal sac	Epithelial cell	CL:0000066	Eye	UBERON:0000970	0.001082985	0.308658149	0.260516605	0.283094236	0.826708384	0.548068973	NA
FetalKidney_Smooth muscle cell	Fetal	Urinary system	FetalKidney	Smooth muscle cell	Smooth muscle cell	CL:0000192	Kidney	UBERON:0002113	0.060473214	0.199925833	0.404797048	0.284400294	0.827984694	0.54606996	NA
TS_Kidney_b cell	Adult	Urinary system	TS_Kidney	b cell	B cell	CL:0000236	Kidney	UBERON:0002113	0.02602624	0.226028177	0.358671587	0.284753408	0.827984694	0.54553107	NA
AdultTransverseColon_Smooth muscle cell	Adult	Digestive system	AdultTransverseColon	Smooth muscle cell	Smooth muscle cell	CL:0000192	Transverse colon	UBERON:0001157	0.055091708	0.210506752	0.397416974	0.291217395	0.844966959	0.535782688	NA
Fetal_HCA_Stomach_Lymphatic endothelial cells	Fetal	Digestive system	Fetal_HCA_Stomach	Lymphatic endothelial cells	Epithelial cell	CL:0000066	Stomach	UBERON:0000945	0.06500503	0.206842543	0.407380074	0.292715168	0.846012742	0.533554773	NA
Enge_2017_Pancreas_pancreatic D cell	Adult	Digestive system	Enge_2017_Pancreas	pancreatic D cell	Pancreatic D cell	CL:0000173	Pancreas	UBERON:0001264	0.056360053	0.210383239	0.400738007	0.29282655	0.846012742	0.533389549	NA

FetalHeart_Fetal neuron	Fetal	Cardiovascular system	FetalHeart	Fetal neuron	Neuron	CL:0000540	Heart	UBERON:0000948	0.075885844	0.205401562	0.411808118	0.293512354	0.846189873	0.532373614	NA
VentoTormo_2018_decidua_glandular epithelial cell (Epi2)	Maternal-Fetal	Reproductive system	VentoTormo_2018_decidua	glandular epithelial cell (Epi2)	Epithelial cell	CL:0000066	Decidua	UBERON:0002450	0.005747416	0.275350901	0.316236162	0.299626036	0.861488598	0.523420452	NA
TS_Liver_nk cell	Adult	Digestive system	TS_Liver	nk cell	Natural killer cell	CL:0000623	Liver	UBERON:0002107	0.000498015	0.377042995	0.231734317	0.300352666	0.861488598	0.522368509	NA
Braga_2020_lung_Lymphatic	Adult	Respiratory system	Braga_2020_lung	Lymphatic	Endothelial cell	CL:0000115	Lung	UBERON:0002048	0.089490581	0.205977954	0.425092251	0.300805443	0.861488598	0.52171431	NA
FetalStomach_Fetal stromal cell	Fetal	Digestive system	FetalStomach	Fetal stromal cell	Stromal cell	CL:0000499	Stomach	UBERON:0000945	0.058730581	0.217464632	0.403690037	0.301362063	0.861488598	0.52091142	NA
AdultStomach_Gastric chief cell	Adult	Digestive system	AdultStomach	Gastric chief cell	Peptic cell	CL:0000155	Stomach	UBERON:0000945	0.053620033	0.227139791	0.390774908	0.303721956	0.864792451	0.517523812	NA
Fetal_HCA_Eye_Retinal pigment cells	Fetal	Sensory system	Fetal_HCA_Eye	Retinal pigment cells	Retinal pigment epithelial cell	CL:0002586	Eye	UBERON:0000970	0.089226693	0.209600992	0.423616236	0.303794248	0.864792451	0.517420453	NA
TS_Thymus_immature natural killer cell	Adult	Lymphatic system	TS_Thymus	immature natural killer cell	Natural killer cell	CL:0000623	Thymus	UBERON:0002370	0.027931251	0.247807576	0.361623616	0.30578257	0.865249582	0.514587273	NA
TS_Lung_lung microvascular endothelial cell	Adult	Respiratory system	TS_Lung	lung microvascular endothelial cell	Endothelial cell	CL:0000115	Lung	UBERON:0002048	0.000500269	0.379554419	0.236162362	0.305839004	0.865249582	0.514507129	NA
TS_Small_Intestine_immature enterocyte	Adult	Digestive system	TS_Small_Intestine	immature enterocyte	Enterocyte	CL:0000584	Small intestine	UBERON:0002108	4.01359E-05	0.45839667	0.195571956	0.305870517	0.865249582	0.514462383	NA
FetalStomach_Smooth muscle cell	Fetal	Digestive system	FetalStomach	Smooth muscle cell	Smooth muscle cell	CL:0000192	Stomach	UBERON:0000945	0.094515752	0.209724505	0.430258303	0.307282372	0.867432529	0.512462353	NA
Fetal_HCA_Muscle_Vascular endothelial cells	Fetal	Muscular system	Fetal_HCA_Muscle	Vascular endothelial cells	Endothelial cell	CL:0000115	Muscle organ	UBERON:0001630	0.075291385	0.220758303	0.410701107	0.308511085	0.869090477	0.510729227	NA
TS_Lung_nk cell	Adult	Respiratory system	TS_Lung	nk cell	Natural killer cell	CL:0000623	Lung	UBERON:0002048	0.027956441	0.251512956	0.364206642	0.310560107	0.873047605	0.507854332	NA
TS_Uterus_fibroblast	Adult	Reproductive system	TS_Uterus	fibroblast	Fibroblast	CL:0000057	Uterus	UBERON:0000995	9.55425E-05	0.444357397	0.207380074	0.311868783	0.87491139	0.506028064	NA
TS_Lymph_Node_cd141-positive myeloid dendritic cell	Adult	Lymphatic system	TS_Lymph_Node	cd141-positive myeloid dendritic cell	Dendritic cell	CL:0000451	Lymph node	UBERON:0000029	9.67782E-05	0.439787428	0.211439114	0.313861613	0.876862823	0.503261797	NA
TS_Tongue_immune cell	Adult	Digestive system	TS_Tongue	immune cell	Immune cell	NA	Tongue	UBERON:0001723	0.001081408	0.35966888	0.259778598	0.314919952	0.878827907	0.501799824	NA
AdultKidney_Endothelial cell (APC)	Adult	Urinary system	AdultKidney	Endothelial cell (APC)	Endothelial cell	CL:0000115	Kidney	UBERON:0002113	0.031611385	0.256700488	0.369372694	0.318190222	0.887135289	0.497313171	NA
TS_Lung_macrophage	Adult	Respiratory system	TS_Lung	macrophage	Macrophage	CL:0000235	Lung	UBERON:0002048	4.02091E-05	0.483840279	0.199261993	0.321929291	0.895117182	0.492239507	NA
AdultTransverseColon_Enterocyte	Adult	Digestive system	AdultTransverseColon	Enterocyte	Enterocyte	CL:0000584	Transverse colon	UBERON:0001157	0.055091708	0.243072924	0.397416974	0.322374306	0.895117182	0.491639348	NA
TS_Lung_cd8-positive alpha-beta t cell	Adult	Respiratory system	TS_Lung	cd8-positive alpha-beta t cell	T cell	CL:0000084	Lung	UBERON:0002048	0.008575765	0.295565807	0.328782288	0.322717623	0.895821339	0.489833657	NA
AdultTrachea_Endothelial cell	Adult	Respiratory system	AdultTrachea	Endothelial cell	Endothelial cell	CL:0000115	Trachea	UBERON:0003126	0.055086159	0.245913716	0.395571956	0.323950152	0.895821339	0.489521812	NA
TS_Thymus_monocyte	Adult	Lymphatic system	TS_Thymus	monocyte	Monocyte	CL:0000576	Thymus	UBERON:0002370	0.001075749	0.385977077	0.253874539	0.325609401	0.898575842	0.487303064	NA
Voigt_2019_retina_1_Fibroblast	Adult	Sensory system	Voigt_2019_retina_1	Fibroblast	Fibroblast	CL:0000057	Retina	UBERON:0000966	0.08507369	0.234591721	0.419926199	0.326819306	0.900081624	0.485692297	NA
Fetal_HCA_Liver_Lymphoid cells	Fetal	Digestive system	Fetal_HCA_Liver	Lymphoid cells	Lymphoid cell	CL:0001065	Liver	UBERON:0002107	0.056988483	0.24710767	0.402214022	0.328852462	0.903843986	0.482998903	NA
VentoTormo_2018_decidua_decidual macrophage (dM1)	Maternal-Fetal	Reproductive system	VentoTormo_2018_decidua	decidual macrophage (dM1)	Macrophage	CL:0000235	Decidua	UBERON:0002450	0.001442136	0.376754799	0.266051661	0.330802193	0.905986508	0.480431621	NA
TS_Fat_t cell	Adult	Integumentary system	TS_Fat	t cell	T cell	CL:0000084	Perirenal fat	UBERON:0005406	0.008561879	0.30820527	0.325461255	0.330212546	0.905986508	0.480212546	NA
AdultThyroid_Endothelial cell (APC)	Adult	Endocrine system	AdultThyroid	Endothelial cell (APC)	Endothelial cell	CL:0000115	Thyroid gland	UBERON:0002046	0.051926976	0.258388495	0.389667897	0.331833049	0.906525185	0.479077745	NA
Lake_2017_VisualCortex_OPC	Adult	Nervous system	Lake_2017_VisualCortex	OPC	Oligodendrocyte precursor cell	CL:0002453	Visual cortex	UBERON:0000411	0.054031339	0.260447039	0.39220923	0.33521028	0.913349698	0.474682671	NA
TS_Small_Intestine_transit amplifying cell of small intestine	Adult	Digestive system	TS_Small_Intestine	transit amplifying cell of small intestine	Transit amplifying cell	CL:0009010	Small intestine	UBERON:0002108	0.000223425	0.465683917	0.219926199	0.335792816	0.913349698	0.4739286	NA
TS_Liver_monocyte	Adult	Digestive system	TS_Liver	monocyte	Monocyte	CL:0000576	Liver	UBERON:0002107	0.000498015	0.443451638	0.231734317	0.335582642	0.913349698	0.472906284	NA
NeonatalAdrenalGland_Endothelial cell	Neonatal	Endocrine system	NeonatalAdrenalGland	Endothelial cell	Endothelial cell	CL:0000115	Adrenal gland	UBERON:0002369	0.097317631	0.239202861	0.432472325	0.338140451	0.913349698	0.470902872	NA
TS_Spleen_neutrophil	Adult	Lymphatic system	TS_Spleen	neutrophil	Neutrophil	CL:0000775	Spleen	UBERON:0002106	0.001078725	0.401663186	0.257564576	0.338153135	0.913349698	0.470886582	NA
TS_Small_Intestine_duodenum glandular cell	Adult	Digestive system	TS_Small_Intestine	duodenum glandular cell	Duodenum glandular cell	CL:1001589	Small intestine	UBERON:0002108	0.001077016	0.405533249	0.255350554	0.338377526	0.913349698	0.470598489	NA
Fetal_HCA_Stomach_PDE1C_ACSM3 positive cells	Fetal	Digestive system	Fetal_HCA_Stomach	PDE1C_ACSM3 positive cells	Epithelial cell	CL:0000066	Stomach	UBERON:0000945	0.104749443	0.243155266	0.433948339	0.342812013	0.92030855	0.464943968	NA
TS_Small_Intestine_neutrophil	Adult	Digestive system	TS_Small_Intestine	neutrophil	Neutrophil	CL:0000775	Small intestine	UBERON:0002108	0.001077016	0.414714357	0.255350554	0.343667517	0.92030855	0.463861517	NA
AdultOmentum_Stromal cell	Adult	Integumentary system	AdultOmentum	Stromal cell	Stromal cell	CL:0000499	Omentum	UBERON:0003688	0.084321945	0.253653843	0.418450185	0.344214986	0.92030855	0.463170226	NA
TS_Lung_capillary endothelial cell	Adult	Respiratory system	TS_Lung	capillary endothelial cell	Endothelial cell	CL:0000115	Lung	UBERON:0002048	0.008575765	0.322862106	0.328782288	0.344237066	0.92030855	0.463142368	NA
TS_Small_Intestine_goblet cell	Adult	Digestive system	TS_Small_Intestine	goblet cell	Goblet cell	CL:0000160	Small intestine	UBERON:0002108	0.001077016	0.415908313	0.255350554	0.344351612	0.92030855	0.462997879	NA
TS_Eye_monocyte	Adult	Sensory system	TS_Eye	monocyte	Monocyte	CL:0000576	Eye	UBERON:0000970	0.000502823	0.441804802	0.241328413	0.345287298	0.920992694	0.461819398	NA
TS_Mammary_vein endothelial cell	Adult	Endocrine system	TS_Mammary	vein endothelial cell	Endothelial cell	CL:0000115	Mammary gland	UBERON:0001911	0.000497478	0.472477114	0.229520295	0.349352634	0.924273456	0.456735978	NA
TS_Thymus_medullary thymic epithelial cell	Adult	Lymphatic system	TS_Thymus	medullary thymic epithelial cell	Epithelial cell	CL:0000066	Thymus	UBERON:0002370	0.00049899	0.463584202	0.234317343	0.349758425	0.924273456	0.456231816	NA
Fetal_HCA_Lung_Vascular endothelial cells	Fetal	Respiratory system	Fetal_HCA_Lung	Vascular endothelial cells	Endothelial cell	CL:0000115	Lung	UBERON:0002048	0.057256123	0.270039856	0.40295203	0.350174071	0.924273456	0.455716081	NA
Fetal_HCA_Heart_Epicardial fat cells	Fetal	Cardiovascular system	Fetal_HCA_Heart	Epicardial fat cells	Epicardial adipocyte	CL:1000309	Heart	UBERON:0000948	0.091690067	0.255259507	0.42804428	0.351169614	0.924273456	0.45448307	NA
TS_Uterus_macrophage	Adult	Reproductive system	TS_Uterus	macrophage	Macrophage	CL:0000235	Uterus	UBERON:0000995	0.001070478	0.434270529	0.251660517	0.351228002	0.924273456	0.454410866	NA
Lake_2017_VisualCortex_In4a	Adult	Nervous system	Lake_2017_VisualCortex	In4a	Inhibitory neuron	CL:0008029	Visual cortex	UBERON:0000411	0.054031339	0.278973939	0.39220923	0.351535713	0.924273456	0.454030547	NA
VentoTormo_2018_decidua_Hofbauer cell (HB)	Maternal-Fetal	Reproductive system	VentoTormo_2018_decidua	Hofbauer cell (HB)	Hofbauer cell	CL:3000001	Decidua	UBERON:0002450	0.00293503	0.38190116	0.286715867	0.3516891	0.924273456	0.453841092	NA
VentoTormo_2018_decidua_placental extravillous trophoblast cell (EVT)	Maternal-Fetal	Reproductive system	VentoTormo_2018_decidua	placental extravillous trophoblast cell (EVT)	Trophoblast cell	CL:0000351	Decidua	UBERON:0002450	0.005747416	0.346658879	0.316236162	0.351974246	0.924273456	0.453489113	NA
VentoTormo_2018_decidua_maternal endothelial cell (Endo m)	Maternal-Fetal	Reproductive system	VentoTormo_2018_decidua	maternal endothelial cell (Endo m)	Endothelial cell	CL:0000115	Decidua	UBERON:0002450	0.00293503	0.387294546	0.286715867	0.355098579	0.929661499	0.449651065	NA
TS_Salivary_Gland_acinar cell of salivary gland	Adult	Digestive system	TS_Salivary_Gland	acinar cell of salivary gland	Acinar cell	CL:0000622	Saliva-secreting gland	UBERON:0001044	0.000501029	0.465683917	0.238745387	0.355398271	0.929661499	0.44928469	NA
MacParland_2018_liver_Kupffer cell	Adult	Digestive system	MacParland_2018_liver	Kupffer cell	Kupffer cell	CL:0000091	Liver	UBERON:0002107	0.082041493	0.267075552	0.417712177	0.356234137	0.930052517	0.448264465	NA
TS_Prostate_macrophage	Adult	Reproductive system	TS_Prostate	macrophage	Macrophage	CL:0000235	Prostate gland	UBERON:0002367	0.001073708	0.443822175	0.252767528	0.357599027	0.931602225	0.446603672	NA
Madissoon_2020_spleen_NK_CD160pos	Adult	Lymphatic system	Madissoon_2020_spleen	NK_CD160pos	Natural killer cell	CL:0000623	Spleen	UBERON:0002106	0.089214713	0.26649916	0.422509225	0.358505071	0.931602225	0.445504697	NA
FetalHeart_Ventricle cardiomyocyte	Fetal	Cardiovascular system	FetalHeart	Ventricle cardiomyocyte	Fetal cardiomyocyte	CL:0002495	Heart	UBERON:0000948	0.119905442	0.254683115	0.442804428	0.358890304	0.931602225	0.445038275	NA
TS_Blood_macrophage	Adult	Lymphatic system	TS_Blood	macrophage	Macrophage	CL:0000235	Blood	UBERON:0000178	0.000502041	0.475359076	0.240590406	0.362353488	0.938793453	0.440867554	NA
Fetal_HCA_Intestine_Vascular endothelial cells	Fetal	Digestive system	Fetal_HCA_Intestine	Vascular endothelial cells	Endothelial cell	CL:0000115	Intestine	UBERON:0000160	0.089686036	0.269669318	0.425830258	0.363364119	0.939411391	0.43965796	NA
TS_Mammary_macrophage	Adult	Endocrine system	TS_Mammary	macrophage	Macrophage	CL:0000235	Mammary gland	UBERON:0001911	0.0044525	0.389270749	0.29704797	0.365090018	0.939411391	0.437600041	NA
AdultStomach_Fibroblast	Adult	Digestive system	AdultStomach	Fibroblast	Fibroblast	CL:0000057	Stomach	UBERON:0000945	0.135302209	0.259006058	0.447601476	0.365735556	0.939411391	0.436832816	NA
AdultThyroid_Fibroblast	Adult	Endocrine system	AdultThyroid	Fibroblast	Fibroblast	CL:0000057	Thyroid gland	UBERON:0002046	0.084746654	0.276915394	0.419188192	0.366054731	0.939411391	0.436453976	NA
TS_Lung_endothelial cell of lymphatic vessel	Adult	Respiratory system	TS_Lung	endothelial cell of lymphatic vessel	Endothelial cell	CL:0000115	Lung	UBERON:0002048	0.047395162	0.299600554	0.387453875	0.366058461	0.939411391	0.436449551	NA
Madissoon_2020_spleen_NK_FCGR3Apos	Adult	Lymphatic system	Madissoon_2020_spleen	NK_FCGR3Apos	Natural killer cell	CL:0000623	Spleen	UBERON:0002106	0.089214713	0.276339002	0.422509225	0.367508763	0.940569257	0.434732301	NA
CordBloodCD34P_Antigen presenting cell (RPS high)	Neonatal	Reproductive system	CordBloodCD34P	Antigen presenting cell (RPS high)	Professional antigen presenting cell	CL:0000145	Umbilical cord blood	UBERON:0012168	0.131328114	0.263205489	0.444280443	0.367897938	0.940569257	0.434272646	NA
TS_Lymph_Node_naive b cell	Adult	Lymphatic system	TS_Lymph_Node	naive b cell	B cell	CL:0000236	Lymph node	UBERON:0000029	0.0158376	0.342459449	0.346125461	0.371314187	0.947515487	0.430258457	NA
VentoTormo_2018_decidua_CD16-positive natural killer cell (NK CD16+)	Maternal-Fetal	Reproductive system	VentoTormo_2018_decidua	CD16-positive natural killer cell (NK CD16+)	Natural killer cell	CL:0000623	Decidua	UBERON:0002450	0.089970484	0.279385648	0.427306273	0.373122343	0.950339801	0.428148744	NA
TS_Trachea_cd8-positive, alpha-beta t cell	Adult	Respiratory system	TS_Trachea	cd8-positive, alpha-beta t cell	T cell	CL:0000084	Trachea	UBERON:0003126	0.004443206	0.407715306	0.295571956	0.375510206	0.954262828	0.425378255	NA
TS_Pancreas_plasma cell	Adult	Digestive system	TS_Pancreas	plasma cell	Plasma cell	CL:0000786	Pancreas	UBERON:0001264	0.00213589	0.445304327	0.271217712	0.376071107	0.954262828	0.424730031	NA
TS_Pancreas_nk cell	Adult	Digestive system	TS_Pancreas	nk cell	Natural killer cell	CL:0000623	Pancreas	UBERON:0001264	0.00213589	0.448474486	0.271217712	0.377885532	0.957074572	0.422639735	NA
TS_Thymus_innate lymphoid cell	Adult	Lymphatic system	TS_Thymus	innate lymphoid cell	Innate lymphoid cell	CL:0001065	Thymus	UBERON:0002370	0.004460717	0.407715306	0.299261993	0.378684387	0.957308477	0.421722601	NA
FetalFemaleGonad_Fetal mesenchymal progenitor	Fetal	Reproductive system	FetalFemaleGonad	Fetal mesenchymal progenitor	Mesenchymal stem cell	CL:0000134	Immature gonad	UBERON:2005272	0.138388018	0.26929878	0.456088561	0.380386472	0.959820615	0.419774936	NA
TS_Lymph_Node_cd4-positive, alpha-beta memory t cell	Adult	Lymphatic system	TS_Lymph_Node	cd4-positive, alpha-beta memory t cell	T cell	CL:0000084	Lymph node	UBERON:0000029	0.119757978	0.280497262	0.442066421	0.382843443	0.964224657	0.416978786	NA
TS_Spleen_intermediate monocyte	Adult	Lymphatic system	TS_Spleen	intermediate monocyte	Monocyte	CL:0000576	Spleen	UBERON:0002106	0.004469449	0.41483787	0.301107011	0.38474424	0.964610982	0.414827873	NA
TS_Trachea_macrophage	Adult	Respiratory system	TS_Trachea	macrophage	Macrophage	CL:0000235	Trachea	UBERON:0003126	0.001070276	0.498949995	0.250553506	0.384958619	0.964610982	0.414585953	NA
TS_Spleen_cd8-positive, alpha-beta t cell	Adult	Lymphatic system	TS_Spleen	cd8-positive, alpha-beta t cell	T cell	CL:0000084	Spleen	UBERON:0002106	0.001078725	0.486310532	0.257564576	0.385463028	0.964610982	0.414017272	NA
TS_Trachea_mast cell	Adult	Respiratory system	TS_Trachea	mast cell	Mast cell	CL:0000097	Trachea	UBERON:0003126	0.015743807	0.367903058	0.34095941	0.385844393	0.964610982	0.413587807	NA
TS_Mammary_endothelial cell of artery	Adult	Endocrine system	TS_Mammary	endothelial cell of artery	Endothelial cell	CL:0000115	Mammary gland	UBERON:0001911	0.002228374	0.467289582	0.272324723	0.389598096	0.972201511	0.409383174	NA
TS_Lymph_Node_t cell	Adult	Lymphatic system	TS_Lymph_Node	t cell	T cell	CL:0000084	Lymph node	UBERON:0000029	0.0158376	0.371484925	0.346125461	0.392324612	0.974044836	0.406354446	NA
AdultStomach_Macrophage	Adult	Digestive system	AdultStomach	Macrophage	Macrophage	CL:0000235	Stomach	UBERON:0000945	0.087176775	0.305776187	0.420664207	0.392424509	0.974044836	0.406243876	NA
TS_Salivary_Gland_plasma cell	Adult	Digestive system	TS_Salivary_Gland	plasma cell	Plasma cell	CL:0000786	Saliva-secreting gland	UBERON:0001044	0.004475113	0.42690094	0.301845018	0.392893585	0.974044836	0.405725062	NA
TS_Liver_fibroblast	Adult	Digestive system	TS_Liver	fibroblast	Fibroblast	CL:0000057	Liver	UBERON:0002107	0.004459264	0.432706036	0.298154982	0.393212196	0.974044836	0.405373021	NA
TS_Pancreas_t cell	Adult	Digestive system	TS_Pancreas	t cell	T cell	CL:0000084	Pancreas	UBERON:0001264	0.00824172	0.406191984	0.323247232	0.397875399	0.980334498	0.400252913	NA
TS_Skin_cd4-positive, alpha-beta memory t cell	Adult	Integumentary system	TS_Skin	cd4-positive, alpha-beta memory t cell	T cell	CL:0000084	Skin of body	UBERON:0002097	0.015778717	0.386059419	0.342066421	0.399411204	0.980334498	0.398579757	NA
TS_Skin_mast cell	Adult	Integumentary system	TS_Skin	mast cell	Mast cell	CL:0000097	Skin of body	UBERON:0002097	0.119556724	0.301576757	0.43800738	0.399481817	0.980334498	0.398502983	NA
FetalIntestine_Smooth muscle cell	Fetal	Digestive system	FetalIntestine	Smooth muscle cell	Smooth muscle cell	CL:0000192	Intestine	UBERON:0000160	0.135007467	0.301247389	0.445756458	0.40389712	0.980334498	0.393729244	NA
Madissoon_2020_lung_Blood_vessel	Adult	Respiratory system	Madissoon_2020_lung	Blood_vessel	Endothelial cell	CL:0000115	Lung	UBERON:0002048	0.102389684	0.311334257	0.433210332	0.405080927	0.980334498	0.392458204	NA
TS_Lymph_Node_plasma cell	Adult	Lymphatic system	TS_Lymph_Node	plasma cell	Plasma cell	CL:0000786	Lymph node	UBERON:0000029	0.00108066	0.526205123	0.25904059	0.407948764	0.980334498	0.389394379	NA
Fetal_HCA_Stomach_Parietal and chief cells	Fetal	Digestive system	Fetal_HCA_Stomach	Parietal and chief cells	Epithelial cell	CL:0000066	Stomach	UBERON:0000945	0.160797917	0.293095554	0.466420664	0.408740012	0.980334498	0.388552846	NA
TS_Thymus_dendritic cell	Adult	Lymphatic system	TS_Thymus	dendritic cell	Dendritic cell	CL:0000451	Thymus	UBERON:0002370	0.004460717	0.457243885	0.299261993	0.408998429	0.980334498	0.388278361	NA
AdultStomach_Fetal stromal cell	Adult	Digestive system	AdultStomach	Fetal stromal cell	Stromal cell	CL:0000499	Stomach	UBERON:0000945	0.135302209	0.307134826	0.447601476	0.410266476	0.980334498	0.386933969	NA
TS_Skin_cd1c-positive myeloid dendritic cell	Adult	Integumentary system	TS_Skin	cd1c-positive myeloid dendritic cell	Myeloid cell	CL:0000763	Skin of body	UBERON:0002097	0.015778717	0.401992553	0.342066421	0.41033424	0.980334498	0.386862242	NA
TS_Eye_adipocyte	Adult	Sensory system	TS_Eye	adipocyte	Epicardial adipocyte	CL:1000309	Eye	UBERON:0000970	0.002246333	0.495326957	0.277859779	0.410578791	0.980334498	0.386603489	NA
AdultTrachea_Basal cell	Adult	Respiratory system	AdultTrachea	Basal cell	Basal cell	CL:0000646	Trachea	UBERON:0003126	0.138182125	0.302441345	0.455350554	0.410748118	0.980334498	0.386424417	NA
Fetal_HCA_Kidney_Ureteric bud cells	Fetal	Urinary system	Fetal_HCA_Kidney	Ureteric bud cells	Epithelial cell	CL:0000066	Kidney	UBERON:0002113	0.092422582	0.321503466	0.429151292	0.411256987	0.980334498	0.38588671	NA
TS_Eye_retinal pigment epithelial cell	Adult	Sensory system	TS_Eye	retinal pigment epithelial cell	Retinal pigment epithelial cell	CL:0002586	Eye	UBERON:0000970	0.002246333	0.498414773	0.277859779	0.412277647	0.980334498	0.384810211	NA
TS_Spleen_cd1c-positive myeloid dendritic cell	Adult	Lymphatic system	TS_Spleen	cd1c-positive myeloid dendritic cell	Dendritic cell	CL:0000451	Spleen	UBERON:0002106	0.002238533	0.501132052	0.276752768	0.412673416	0.980334498	0.384393507	NA
FetalKidney_Fetal neuron	Fetal	Urinary system	FetalKidney	Fetal neuron	Neuron	CL:0000540	Kidney	UBERON:0002113	0.148465407	0.303429447	0.460516605	0.414733149	0.980334498	0.382231251	NA
Fetal_HCA_Eye_Retinal progenitors and Muller glia	Fetal	Sensory system	Fetal_HCA_Eye	Retinal progenitors and Muller glia	Mueller cell	CL:0000636	Eye	UBERON:0000970	0.138143059	0.313186947	0.453505535	0.419236293	0.980334498	0.377541128	NA
AdultThyroid_Thyroid follicular cell	Adult	Endocrine system	AdultThyroid	Thyroid follicular cell	Thyroid follicular cell	CL:0002258	Thyroid gland	UBERON:0002046	0.13201515	0.320350682	0.44501845	0.42026961	0.980334498	0.376472013	NA
TS_Skin_regulatory t cell	Adult	Integumentary system	TS_Skin	regulatory t cell	T cell	CL:0000084	Skin of body	UBERON:0002097	0.008555596	0.441887144	0.324723247	0.422076387	0.980334498	0.374608944	NA
FetalMuscle_Fetal stromal cell	Fetal	Muscular system	FetalMuscle	Fetal stromal cell	Stromal cell	CL:0000499	Muscle organ	UBERON:0001630	0.097035038	0.333525366	0.431734317	0.423052688	0.980334498	0.373605541	NA
Voigt_2019_retina_2_RPE/Rod/melanocyte	Adult	Sensory system	Voigt_2019_retina_2	RPE/Rod/melanocyte	Retinal pigment epithelial cell	CL:0002586	Retina	UBERON:0000966	0.144725369	0.313886852	0.459409594	0.42345619	0.980334498	0.373191514	NA
AdultLung_Endothelial cell	Adult	Respiratory system	AdultLung	Endothelial cell	Endothelial cell	CL:0000115	Lung	UBERON:0002048	0.151846914	0.313351631	0.462361624	0.424769767	0.980334498	0.371846401	NA
AdultTransverseColon_Endothelial cell (APC)	Adult	Digestive system	AdultTransverseColon	Endothelial cell (APC)	Endothelial cell	CL:0000115	Transverse colon	UBERON:0001157	0.089277659	0.341471347	0.424354244	0.424814288	0.980334498	0.371800885	NA
TS_Skin_naive thymus-derived cd8-positive, alpha-beta t cell	Adult	Integumentary system	TS_Skin	naive thymus-derived cd8-positive, alpha-beta t cell	T cell	CL:0000084	Skin of body	UBERON:0002097	0.027918442	0.403392363	0.360516605	0.425827141	0.980334498	0.370766661	NA
FetalKidney_Endothelial cell	Fetal	Urinary system	FetalKidney	Endothelial cell	Endothelial cell	CL:0000115	Kidney	UBERON:0002113	0.096960228	0.338754069	0.43099631	0.426929078	0.980334498	0.369644264	NA
AdultLung_Smooth muscle cell	Adult	Respiratory system	AdultLung	Smooth muscle cell	Smooth muscle cell	CL:0000192	Lung	UBERON:0002048	0.151846914	0.31611008	0.462361624	0.427228038	0.980334498	0.369340254	NA
Madissoon_2020_spleen_T_CD8_CTL	Adult	Lymphatic system	Madissoon_2020_spleen	T_CD8_CTL	T cell	CL:0000084	Spleen	UBERON:0002106	0.138051197	0.328502517	0.450553506	0.430775574	0.980334498	0.36574893	NA
TS_Thymus_dn1 thymic pro-t cell	Adult	Lymphatic system	TS_Thymus	dn1 thymic pro-t cell	T cell	CL:0000084	Thymus	UBERON:0002370	0.008564142	0.458067303	0.326568266	0.433790083	0.980334498	0.362720381	NA
Fetal_HCA_Pancreas_CCL19_CCL21 positive cells	Fetal	Digestive system	Fetal_HCA_Pancreas	CCL19_CCL21 positive cells	Mesenchymal stem cell	CL:0000134	Pancreas	UBERON:0001264	0.138138932	0.335378056	0.45202952	0.437596361	0.980334498	0.358926298	NA
FetalSkin_Macrophage	Fetal	Integumentary system	FetalSkin	Macrophage	Macrophage	CL:0000235	Skin of body	UBERON:0002097	0.122726434	0.345794291	0.443542435	0.440932044	0.980334498	0.355628338	NA
Madissoon_2020_spleen_B_naive	Adult	Lymphatic system	Madissoon_2020_spleen	B_naive	B cell	CL:0000236	Spleen	UBERON:0002106	0.138051197	0.341430176	0.450553506	0.441790279	0.980334498	0.354783844	NA
Fetal_HCA_Pancreas_Smooth muscle cells	Fetal	Digestive system	Fetal_HCA_Pancreas	Smooth muscle cells	Smooth muscle cell	CL:0000192	Pancreas	UBERON:0001264	0.138138932	0.341800714	0.45202952	0.443045697	0.980334498	0.353551477	NA
Fetal_HCA_Kidney_Vascular endothelial cells	Fetal	Urinary system	Fetal_HCA_Kidney	Vascular endothelial cells	Endothelial cell	CL:0000115	Kidney	UBERON:0002113	0.092422582	0.360739323	0.429151292	0.443620094	0.980334498	0.352988791	NA
TS_Small_Intestine_mast cell	Adult	Digestive system	TS_Small_Intestine	mast cell	Mast cell	CL:0000097	Small intestine	UBERON:0002108	0.008570572	0.472806481	0.327675277	0.443835051	0.980334498	0.352778403	NA
TS_Muscle_capillary endothelial cell	Adult	Muscular system	TS_Muscle	capillary endothelial cell	Capillary endothelial cell	CL:0002144	Muscle organ	UBERON:0001630	0.004468804	0.516735819	0.300369004	0.444365193	0.980334498	0.352259966	NA
Fetal_HCA_Muscle_Erythroblasts	Fetal	Muscular system	Fetal_HCA_Muscle	Erythroblasts	Erythroblast	CL:0000765	Muscle organ	UBERON:0001630	0.179660887	0.330272866	0.47601476	0.448084469	0.980334498	0.348640109	NA
Voigt_2019_retina_2_Macrophage 1	Adult	Sensory system	Voigt_2019_retina_2	Macrophage 1	Macrophage	CL:0000235	Retina	UBERON:0000966	0.144725369	0.342459449	0.459409594	0.448296079	0.980334498	0.34843506	NA
TS_Kidney_cd4-positive helper t cell	Adult	Urinary system	TS_Kidney	cd4-positive helper t cell	T cell	CL:0000084	Kidney	UBERON:0002113	0.170425994	0.338383531	0.467896679	0.450141168	0.980334498	0.346651266	NA
VentoTormo_2018_decidua_plasmacytoid dendritic cell (DC2)	Maternal-Fetal	Reproductive system	VentoTormo_2018_decidua	plasmacytoid dendritic cell (DC2)	Plasmacytoid dendritic cell	CL:0000784	Decidua	UBERON:0002450	0.033938036	0.423771953	0.374169742	0.450572483	0.980334498	0.346235334	NA
FetalAdrenalGland_Endothelial cell	Fetal	Endocrine system	FetalAdrenalGland	Endothelial cell	Endothelial cell	CL:0000115	Adrenal gland	UBERON:0002369	0.110251828	0.364856412	0.434686347	0.45063778	0.980334498	0.346172401	NA
AdultJeJunum_Stromal cell	Adult	Digestive system	AdultJeJunum	Stromal cell	Stromal cell	CL:0000499	Jejunum	UBERON:0002115	0.16111673	0.341430176	0.467158672	0.452299757	0.980334498	0.344573646	NA
TS_Bladder_endothelial cell of lymphatic vessel	Adult	Urinary system	TS_Bladder	endothelial cell of lymphatic vessel	Endothelial cell	CL:0000115	Bladder organ	UBERON:0018707	0.047367107	0.423689611	0.385239852	0.459085609	0.980334498	0.338106321	NA
Fetal_HCA_Eye_Vascular endothelial cells	Fetal	Sensory system	Fetal_HCA_Eye	Vascular endothelial cells	Endothelial cell	CL:0000115	Eye	UBERON:0000970	0.138143059	0.362797867	0.453505535	0.461452615	0.980334498	0.335872889	NA
TS_Muscle_fast muscle cell	Adult	Muscular system	TS_Muscle	fast muscle cell	Cell of skeletal muscle	CL:0000188	Muscle organ	UBERON:0001630	0.015805248	0.481617051	0.344649446	0.464077739	0.980334498	0.333409263	NA
Madissoon_2020_lung_Lymph_vessel	Adult	Respiratory system	Madissoon_2020_lung	Lymph_vessel	Endothelial cell	CL:0000115	Lung	UBERON:0002048	0.155624889	0.358351411	0.464944649	0.465198102	0.980334498	0.332362065	NA
AdultAscendingColon_T cell	Adult	Digestive system	AdultAscendingColon	T cell	T cell	CL:0000084	Ascending colon	UBERON:0001156	0.20020031	0.347194101	0.480442804	0.465544464	0.980334498	0.332038834	NA
Fetal_HCA_Kidney_Megakaryocytes	Fetal	Urinary system	Fetal_HCA_Kidney	Megakaryocytes	Megakaryocyte	CL:0000556	Kidney	UBERON:0002113	0.14231723	0.366667931	0.458302583	0.467756953	0.980334498	0.329979748	NA
Lake_2017_VisualCortex_In7	Adult	Nervous system	Lake_2017_VisualCortex	In7	Inhibitory neuron	CL:0008029	Visual cortex	UBERON:0000411	0.135749176	0.37498445	0.448708487	0.468138216	0.980334498	0.329625905	NA
Madissoon_2020_oesophagus_Epi_basal	Adult	Digestive system	Madissoon_2020_oesophagus	Epi_basal	Basal cell	CL:0000646	Esophagus	UBERON:0001043	0.151612575	0.365021095	0.461254613	0.468332372	0.980334498	0.329445822	NA
AdultPleura_Monocyte	Adult	Respiratory system	AdultPleura	Monocyte	Monocyte	CL:0000576	Pleura	UBERON:0000977	0.138159501	0.375272647	0.454612546	0.47230124	0.980334498	0.325780914	NA
TS_Lung_endothelial cell of artery	Adult	Respiratory system	TS_Lung	endothelial cell of artery	Endothelial cell	CL:0000115	Lung	UBERON:0002048	0.027956441	0.469224614	0.364206642	0.47281572	0.980334498	0.325308093	NA
TS_Skin_cd8-positive, alpha-beta memory t cell	Adult	Integumentary system	TS_Skin	cd8-positive, alpha-beta memory t cell	T cell	CL:0000084	Skin of body	UBERON:0002097	0.027918442	0.475441418	0.360516605	0.473715636	0.980334498	0.32448228	NA
TS_Prostate_cd8-positive, alpha-beta t cell	Adult	Reproductive system	TS_Prostate	cd8-positive, alpha-beta t cell	T cell	CL:0000084	Prostate gland	UBERON:0002367	0.119559736	0.393223154	0.439114391	0.475942834	0.980334498	0.322445208	NA
AdultAdrenalGland_Macrophage	Adult	Endocrine system	AdultAdrenalGland	Macrophage	Macrophage	CL:0000235	Adrenal gland	UBERON:0002369	0.135052381	0.391452806	0.446494465	0.479638672	0.980334498	0.319085808	NA
TS_Lymph_Node_mature nk t cell	Adult	Lymphatic system	TS_Lymph_Node	mature nk t cell	Natural killer cell	CL:0000623	Lymph node	UBERON:0000029	0.0158376	0.505496166	0.346125461	0.479958784	0.980334498	0.318796055	NA
VentoTormo_2018_decidua_decidual natural killer cell (dNK1)	Maternal-Fetal	Reproductive system	VentoTormo_2018_decidua	decidual natural killer cell (dNK1)	Natural killer cell	CL:0000623	Decidua	UBERON:0002450	0.056454781	0.436411416	0.401476015	0.480383289	0.980334498	0.318412108	NA
AdultIleum_Smooth muscle cell	Adult	Digestive system	AdultIleum	Smooth muscle cell	Smooth muscle cell	CL:0000192	Ileum	UBERON:0002116	0.195606291	0.366997298	0.477490775	0.480433987	0.980334498	0.318366277	NA
VentoTormo_2018_decidua_innate lymphoid cell (ILC3)	Maternal-Fetal	Reproductive system	VentoTormo_2018_decidua	innate lymphoid cell (ILC3)	Innate lymphoid cell	CL:0001065	Decidua	UBERON:0002450	0.137275874	0.390999926	0.449446494	0.481296641	0.980334498	0.317587169	NA
Voigt_2019_retina_1_Retinal pigment epithelium	Adult	Sensory system	Voigt_2019_retina_1	Retinal pigment epithelium	Retinal pigment epithelial cell	CL:0002586	Retina	UBERON:0000966	0.196763246	0.368644134	0.478228782	0.482274137	0.980334498	0.316706027	NA
Madissoon_2020_lung_Monocyte	Adult	Respiratory system	Madissoon_2020_lung	Monocyte	Monocyte	CL:0000576	Lung	UBERON:0002048	0.155624889	0.38008964	0.464944649	0.483010228	0.980334498	0.316043673	NA
Fetal_HCA_Placenta_Vascular endothelial cells	Fetal	Reproductive system	Fetal_HCA_Placenta	Vascular endothelial cells	Endothelial cell	CL:0000115	Placenta	UBERON:0001987	0.188397026	0.370743849	0.476752768	0.483066573	0.980334498	0.315993014	NA
AdultTransverseColon_T cell	Adult	Digestive system	AdultTransverseColon	T cell	T cell	CL:0000084	Transverse colon	UBERON:0001157	0.203920892	0.366379735	0.484870849	0.484613095	0.980334498	0.314604855	NA
AdultAdrenalGland_Smooth muscle cell	Adult	Endocrine system	AdultAdrenalGland	Smooth muscle cell	Smooth muscle cell	CL:0000192	Adrenal gland	UBERON:0002369	0.200049084	0.372061317	0.479335793	0.48581345	0.980334498	0.313530465	NA
Fetal_HCA_Lung_Megakaryocytes	Fetal	Respiratory system	Fetal_HCA_Lung	Megakaryocytes	Megakaryocyte	CL:0000556	Lung	UBERON:0002048	0.142263224	0.39120578	0.457195572	0.486701013	0.980334498	0.31273775	NA
Lake_2017_VisualCortex_In4b	Adult	Nervous system	Lake_2017_VisualCortex	In4b	Inhibitory neuron	CL:0008029	Visual cortex	UBERON:0000411	0.087637477	0.424842396	0.421402214	0.486996929	0.980334498	0.312473777	NA
TS_Pancreas_pancreatic acinar cell	Adult	Digestive system	TS_Pancreas	pancreatic acinar cell	Acinar cell	CL:0000622	Pancreas	UBERON:0001264	0.015242677	0.53254544	0.33800738	0.488671072	0.980334498	0.31098337	NA
FetalFemaleGonad_Smooth muscle cell	Fetal	Reproductive system	FetalFemaleGonad	Smooth muscle cell	Smooth muscle cell	CL:0000192	Immature gonad	UBERON:2005272	0.204322819	0.375931381	0.485608856	0.493028012	0.980334498	0.307128405	NA
Madissoon_2020_lung_Fibroblast	Adult	Respiratory system	Madissoon_2020_lung	Fibroblast	Fibroblast	CL:0000057	Lung	UBERON:0002048	0.155624889	0.393470179	0.464944649	0.493684494	0.980334498	0.306550513	NA
TS_Thymus_plasma cell	Adult	Lymphatic system	TS_Thymus	plasma cell	Plasma cell	CL:0000786	Thymus	UBERON:0002370	0.008564142	0.57239886	0.326568266	0.500411024	0.980334498	0.300673131	NA
TS_Bladder_vein endothelial cell	Adult	Urinary system	TS_Bladder	vein endothelial cell	Endothelial cell	CL:0000115	Bladder organ	UBERON:0018707	0.027937787	0.51587123	0.362361624	0.500418795	0.980334498	0.300666387	NA
Fetal_HCA_Lung_Lymphatic endothelial cells	Fetal	Respiratory system	Fetal_HCA_Lung	Lymphatic endothelial cells	Endothelial cell	CL:0000115	Lung	UBERON:0002048	0.142263224	0.409197458	0.457195572	0.500672515	0.980334498	0.300446249	NA
Braga_2020_lung_Alveolar cells type 2	Adult	Respiratory system	Braga_2020_lung	Alveolar cells type 2	Alveolar capillary type 2 endothelial cell	CL:4028003	Lung	UBERON:0002048	0.209098011	0.384741951	0.488191882	0.501919187	0.980334498	0.299366202	NA
FetalLiver_Erythroid cell	Fetal	Digestive system	FetalLiver	Erythroid cell	Erythroid lineage cell	CL:0000764	Liver	UBERON:0002107	0.208503375	0.3875004	0.486346863	0.502974485	0.980334498	0.298454045	NA
TS_Large_Intestine_intestinal crypt stem cell of large intestine	Adult	Digestive system	TS_Large_Intestine	intestinal crypt stem cell of large intestine	Intestinal crypt stem cell	CL:0002250	Large intestine	UBERON:0000059	0.177597945	0.402692458	0.470848708	0.504886227	0.980334498	0.296806476	NA
CordBlood_Antigen presenting cell (RPS high)	Neonatal	Reproductive system	CordBlood	Antigen presenting cell (RPS high)	Professional antigen presenting cell	CL:0000145	Umbilical cord blood	UBERON:0012168	0.237588476	0.38330097	0.496678967	0.50616553	0.980334498	0.295707434	NA
TS_Lung_adventitial cell	Adult	Respiratory system	TS_Lung	adventitial cell	Adventitial cell	CL:0002503	Lung	UBERON:0002048	0.177770745	0.401992553	0.475276753	0.507292571	0.980334498	0.294741497	NA
Braga_2020_lung_Endothelium	Adult	Respiratory system	Braga_2020_lung	Endothelium	Endothelial cell	CL:0000115	Lung	UBERON:0002048	0.209098011	0.395158186	0.488191882	0.510354452	0.980334498	0.292128092	NA
TS_Skin_nkt cell	Adult	Integumentary system	TS_Skin	nkt cell	Natural killer cell	CL:0000623	Skin of body	UBERON:0002097	0.047349864	0.505331483	0.384501845	0.512633232	0.980334498	0.290193243	NA
Segerstolpe_2016_PancreaticIslets_pancreatic ductal cell	Adult	Digestive system	Segerstolpe_2016_PancreaticIslets	pancreatic ductal cell	Pancreatic ductal cell	CL:0002079	Pancreas	UBERON:0001264	0.056283385	0.489110153	0.4	0.514829185	0.980334498	0.288336841	NA
TS_Skin_cd4-positive helper t cells	Adult	Integumentary system	TS_Skin	cd4-positive helper t cells	T cell	CL:0000084	Skin of body	UBERON:0002097	0.076910936	0.476553032	0.413284133	0.516958357	0.980334498	0.28654444	NA
Voigt_2019_retina_2_Macrophage 2	Adult	Sensory system	Voigt_2019_retina_2	Macrophage 2	Macrophage	CL:0000235	Retina	UBERON:0000966	0.212242417	0.405327395	0.4900369	0.519670483	0.980334498	0.284271951	NA
TS_Thymus_dn4 thymocyte	Adult	Lymphatic system	TS_Thymus	dn4 thymocyte	Thymocyte	CL:0000893	Thymus	UBERON:0002370	0.015789912	0.578656835	0.343911439	0.520286465	0.980334498	0.283757472	NA
TS_Skin_epithelial cell	Adult	Integumentary system	TS_Skin	epithelial cell	Epithelial cell	CL:0000066	Skin of body	UBERON:0002097	0.177752532	0.425542301	0.473431734	0.524240424	0.980334498	0.280469493	NA
TS_Eye_microglial cell	Adult	Sensory system	TS_Eye	microglial cell	Microglial cell	CL:0000129	Eye	UBERON:0000970	0.028013422	0.550537118	0.366051661	0.524336218	0.980334498	0.280390143	NA
Madissoon_2020_spleen_Monocyte	Adult	Lymphatic system	Madissoon_2020_spleen	Monocyte	Monocyte	CL:0000576	Spleen	UBERON:0002106	0.203832373	0.41669056	0.483763838	0.524423989	0.980334498	0.280317451	NA
Fetal_HCA_Stomach_MUC13_DMBT1 positive cells	Fetal	Digestive system	Fetal_HCA_Stomach	MUC13_DMBT1 positive cells	Epithelial cell	CL:0000066	Stomach	UBERON:0000945	0.235020081	0.40742711	0.495940959	0.525192119	0.980334498	0.279681799	NA
TS_Small_Intestine_plasma cell	Adult	Digestive system	TS_Small_Intestine	plasma cell	Plasma cell	CL:0000786	Small intestine	UBERON:0002108	0.027945841	0.561612087	0.363099631	0.528160227	0.980334498	0.277234307	NA
Madissoon_2020_spleen_T_CD8_MAIT	Adult	Lymphatic system	Madissoon_2020_spleen	T_CD8_MAIT	T cell	CL:0000084	Spleen	UBERON:0002106	0.203832373	0.422495655	0.483763838	0.528902198	0.980334498	0.276624628	NA
TS_Liver_erythrocyte	Adult	Digestive system	TS_Liver	erythrocyte	Erythrocyte	CL:0000232	Liver	UBERON:0002107	0.076999422	0.490756988	0.41697417	0.529290837	0.980334498	0.276305625	NA
FetalSpinalCord_Fetal neuron	Fetal	Nervous system	FetalSpinalCord	Fetal neuron	Neuron	CL:0000540	Spinal cord	UBERON:0002240	0.229955245	0.413355718	0.495202952	0.529389168	0.980334498	0.27622495	NA
AdultArtery_Endothelial cell (APC)	Adult	Cardiovascular system	AdultArtery	Endothelial cell (APC)	Endothelial cell	CL:0000115	Artery	UBERON:0001637	0.151903037	0.442916416	0.463468635	0.530312946	0.980334498	0.275467771	NA
FetalKidney_Fetal stromal cell	Fetal	Urinary system	FetalKidney	Fetal stromal cell	Stromal cell	CL:0000499	Kidney	UBERON:0002113	0.21698889	0.41899613	0.490774908	0.530874617	0.980334498	0.275008039	NA
TS_Small_Intestine_intestinal crypt stem cell	Adult	Digestive system	TS_Small_Intestine	intestinal crypt stem cell	Intestinal crypt stem cell	CL:0002250	Small intestine	UBERON:0002108	0.047381627	0.533368857	0.38597786	0.531247411	0.980334498	0.274703173	NA
TS_Bone_Marrow_neutrophil	Adult	Lymphatic system	TS_Bone_Marrow	neutrophil	Neutrophil	CL:0000775	Bone marrow	UBERON:0002371	0.252503116	0.411873566	0.501107011	0.532075191	0.980334498	0.27402699	NA
Fetal_HCA_Spleen_Mesothelial cells	Fetal	Lymphatic system	Fetal_HCA_Spleen	Mesothelial cells	Mesothelial cell	CL:0000077	Spleen	UBERON:0002106	0.240229257	0.414508503	0.498892989	0.532710177	0.980334498	0.273509006	NA
TS_Liver_neutrophil	Adult	Digestive system	TS_Liver	neutrophil	Neutrophil	CL:0000775	Liver	UBERON:0002107	0.342918316	0.387376888	0.536531365	0.534353646	0.980334498	0.272171223	NA
van_Zyl_eye_2020_SchwalbeLine	Adult	Sensory system	van_Zyl_eye_2020	SchwalbeLine	Schwalbe line	NA	Eye	UBERON:0000970	0.226151055	0.42084882	0.494095941	0.534510475	0.980334498	0.27204378	NA
Fetal_HCA_Liver_Hepatoblasts	Fetal	Digestive system	Fetal_HCA_Liver	Hepatoblasts	Hepatoblast	CL:0005026	Liver	UBERON:0002107	0.20867289	0.433323599	0.487084871	0.539395995	0.980334498	0.268092282	NA
Lake_2017_FrontalCortex_In8	Adult	Nervous system	Lake_2017_FrontalCortex	In8	Inhibitory neuron	CL:0008029	Frontal cortex	UBERON:0001870	0.200675282	0.438593473	0.482287823	0.540115603	0.980334498	0.267513276	NA
TS_Lung_cd4-positive alpha-beta t cell	Adult	Respiratory system	TS_Lung	cd4-positive alpha-beta t cell	T cell	CL:0000084	Lung	UBERON:0002048	0.119596075	0.480793633	0.440221402	0.5403134	0.980334498	0.267354262	NA
AdultKidney_Smooth muscle cell	Adult	Urinary system	AdultKidney	Smooth muscle cell	Smooth muscle cell	CL:0000192	Kidney	UBERON:0002113	0.200639712	0.442587049	0.481180812	0.542341579	0.980334498	0.265727099	NA
TS_Thymus_erythrocyte	Adult	Lymphatic system	TS_Thymus	erythrocyte	Erythrocyte	CL:0000232	Thymus	UBERON:0002370	0.177257598	0.454608948	0.468634686	0.542467036	0.980334498	0.265626647	NA
Lake_2017_FrontalCortex_In1a	Adult	Nervous system	Lake_2017_FrontalCortex	In1a	Excitatory neuron	CL:0008030	Frontal cortex	UBERON:0001870	0.200675282	0.446045403	0.482287823	0.545668112	0.980334498	0.263071425	NA
TS_Lung_non-classical monocyte	Adult	Respiratory system	TS_Lung	non-classical monocyte	Monocyte	CL:0000576	Lung	UBERON:0002048	0.047395162	0.55527177	0.387453875	0.545698738	0.980334498	0.263047051	NA
AdultSigmoidColon_Stromal cell	Adult	Digestive system	AdultSigmoidColon	Stromal cell	Stromal cell	CL:0000499	Sigmoid colon	UBERON:0001159	0.255875359	0.426365719	0.505535055	0.546313898	0.980334498	0.262557751	NA
Madissoon_2020_oesophagus_Fibroblast	Adult	Digestive system	Madissoon_2020_oesophagus	Fibroblast	Fibroblast	CL:0000057	Esophagus	UBERON:0001043	0.220876244	0.44126958	0.491881919	0.548625419	0.980334498	0.260724075	NA
van_Zyl_eye_2020_Fibroblast	Adult	Sensory system	van_Zyl_eye_2020	Fibroblast	Fibroblast	CL:0000057	Eye	UBERON:0000970	0.226151055	0.441681289	0.494095941	0.550425419	0.980334498	0.259301518	NA
TS_Large_Intestine_mature enterocyte	Adult	Digestive system	TS_Large_Intestine	mature enterocyte	Enterocyte	CL:0000584	Large intestine	UBERON:0000059	0.252448124	0.441763631	0.500369004	0.554687627	0.980334498	0.255951521	NA
TS_Spleen_cd141-positive myeloid dendritic cell	Adult	Lymphatic system	TS_Spleen	cd141-positive myeloid dendritic cell	Dendritic cell	CL:0000451	Spleen	UBERON:0002106	0.027959446	0.610646615	0.365313653	0.557746472	0.980334498	0.253563168	NA
TS_Bladder_b cell	Adult	Urinary system	TS_Bladder	b cell	B cell	CL:0000236	Bladder organ	UBERON:0018707	0.076918096	0.537938826	0.414760148	0.55780354	0.980334498	0.253518734	NA
Segerstolpe_2016_PancreaticIslets_acinar cell	Adult	Digestive system	Segerstolpe_2016_PancreaticIslets	acinar cell	Acinar cell	CL:0000622	Pancreas	UBERON:0001264	0.089773332	0.527152053	0.426568266	0.560422929	0.980334498	0.251484105	NA
AdultIleum_Enterocyte progenitor	Adult	Digestive system	AdultIleum	Enterocyte progenitor	Enterocyte	CL:0000584	Ileum	UBERON:0002116	0.277286239	0.44312227	0.508856089	0.561345971	0.980334498	0.25076939	NA
AdultLiver_Antigen presenting cell (RPS high)	Adult	Digestive system	AdultLiver	Antigen presenting cell (RPS high)	Professional antigen presenting cell	CL:0000145	Liver	UBERON:0002107	0.256003062	0.449050878	0.506273063	0.564104195	0.980334498	0.248640671	NA
TS_Lung_basophil	Adult	Respiratory system	TS_Lung	basophil	Basophil	CL:0000767	Lung	UBERON:0002048	0.119596075	0.51727104	0.440221402	0.564654074	0.980334498	0.248217534	NA
Madissoon_2020_oesophagus_Endothelia_vascular_arterial	Adult	Digestive system	Madissoon_2020_oesophagus	Endothelia_vascular_arterial	Endothelial cell	CL:0000115	Esophagus	UBERON:0001043	0.220876244	0.466548506	0.491881919	0.567270625	0.980334498	0.246209705	NA
Madissoon_2020_spleen_Unknown	Adult	Lymphatic system	Madissoon_2020_spleen	Unknown	Unknown	NA	Spleen	UBERON:0002106	0.287083928	0.451521132	0.516236162	0.572548345	0.980334498	0.242187837	NA
AdultAdrenalGland_Endothelial cell (APC)	Adult	Endocrine system	AdultAdrenalGland	Endothelial cell (APC)	Endothelial cell	CL:0000115	Adrenal gland	UBERON:0002369	0.200049084	0.486969266	0.479335793	0.573029081	0.980334498	0.241823337	NA
FetalPancreas_Fetal mesenchymal progenitor	Fetal	Digestive system	FetalPancreas	Fetal mesenchymal progenitor	Mesenchymal stem cell	CL:0000134	Pancreas	UBERON:0001264	0.238586131	0.469306955	0.497416974	0.573057373	0.980334498	0.241801896	NA
TS_Uterus_nk cell	Adult	Reproductive system	TS_Uterus	nk cell	Natural killer cell	CL:0000623	Uterus	UBERON:0000995	0.119400374	0.53810351	0.436162362	0.574885973	0.980334498	0.240418288	NA
TS_Lung_cd4-positive, alpha-beta t cell	Adult	Respiratory system	TS_Lung	cd4-positive, alpha-beta t cell	T cell	CL:0000084	Lung	UBERON:0002048	0.252649942	0.470212715	0.502214022	0.576978755	0.980334498	0.238840178	NA
TS_Trachea_b cell	Adult	Respiratory system	TS_Trachea	b cell	B cell	CL:0000236	Trachea	UBERON:0003126	0.076785217	0.575404334	0.412546125	0.578755719	0.980334498	0.237504704	NA
TS_Salivary_Gland_neutrophil	Adult	Digestive system	TS_Salivary_Gland	neutrophil	Neutrophil	CL:0000775	Saliva-secreting gland	UBERON:0001044	0.119749698	0.542138257	0.443228413	0.581452171	0.980334498	0.235480033	NA
AdultSpleen_Erythroid progenitor cell (RP high)	Adult	Lymphatic system	AdultSpleen	Erythroid progenitor cell (RP high)	Erythroid progenitor cell	CL:0000038	Spleen	UBERON:0002106	0.276046699	0.471489012	0.507749077	0.581648204	0.980334498	0.235339606	NA
AdultUreter_Epithelial cell (intermediated)	Adult	Urinary system	AdultUreter	Epithelial cell (intermediated)	Epithelial cell	CL:0000066	Ureter	UBERON:0000056	0.3108735	0.456502809	0.527675277	0.583769666	0.980334498	0.233758432	NA
TS_Skin_naive thymus-derived cd4-positive, alpha-beta t cell	Adult	Integumentary system	TS_Skin	naive thymus-derived cd4-positive, alpha-beta t cell	T cell	CL:0000084	Skin of body	UBERON:0002097	0.119556724	0.551031169	0.43800738	0.584438871	0.980334498	0.233260907	NA
TS_Lung_intermediate monocyte	Adult	Respiratory system	TS_Lung	intermediate monocyte	Monocyte	CL:0000576	Lung	UBERON:0002048	0.076856098	0.579974365	0.412546125	0.584510785	0.980334498	0.233207836	NA
TS_Bone_Marrow_naive b cell	Adult	Lymphatic system	TS_Bone_Marrow	naive b cell	B cell	CL:0000236	Bone marrow	UBERON:0002371	0.343118289	0.45003898	0.53800738	0.585530164	0.980334498	0.232450727	NA
AdultBladder_B cell (Plasmocyte)	Adult	Urinary system	AdultBladder	B cell (Plasmocyte)	B cell	CL:0000236	Bladder organ	UBERON:0018707	0.33183736	0.454650119	0.533579336	0.586192093	0.980334498	0.23195942	NA
AdultLung_T cell	Adult	Respiratory system	AdultLung	T cell	T cell	CL:0000084	Lung	UBERON:0002048	0.307515925	0.462225563	0.525830258	0.58684442	0.980334498	0.231477917	NA
Madissoon_2020_spleen_T_CD4_fh	Adult	Lymphatic system	Madissoon_2020_spleen	T_CD4_fh	T cell	CL:0000084	Spleen	UBERON:0002106	0.287083928	0.471777209	0.516236162	0.58754588	0.980334498	0.230958214	NA
AdultBladder_Endothelial cell (APC)	Adult	Urinary system	AdultBladder	Endothelial cell (APC)	Endothelial cell	CL:0000115	Bladder organ	UBERON:0018707	0.239355848	0.49129221	0.498154982	0.589225478	0.980334498	0.229718482	NA
TS_Vasculature_endothelial cell	Adult	Cardiovascular system	TS_Vasculature	endothelial cell	Endothelial cell	CL:0000115	Vasculature	UBERON:0002049	0.076928461	0.590143512	0.415498155	0.589877976	0.980334498	0.229237818	NA
AdultLung_Endothelial cell (APC)	Adult	Respiratory system	AdultLung	Endothelial cell (APC)	Endothelial cell	CL:0000115	Lung	UBERON:0002048	0.22118228	0.499732242	0.49298893	0.591504301	0.980334498	0.228040093	NA
Fetal_HCA_Kidney_Myeloid cells	Fetal	Urinary system	Fetal_HCA_Kidney	Myeloid cells	Myeloid cell	CL:0000763	Kidney	UBERON:0002113	0.209162095	0.505125628	0.489298893	0.592616702	0.980334498	0.227226112	NA
AdultThyroid_Stromal cell	Adult	Endocrine system	AdultThyroid	Stromal cell	Stromal cell	CL:0000499	Thyroid gland	UBERON:0002046	0.277980119	0.485816482	0.5099631	0.59344215	0.980334498	0.226621611	NA
TS_Skin_nk cell	Adult	Integumentary system	TS_Skin	nk cell	Natural killer cell	CL:0000623	Skin of body	UBERON:0002097	0.177752532	0.523529015	0.473431734	0.593591109	0.980334498	0.226512613	NA
FetalCalvaria_Fetal mesenchymal progenitor	Fetal	Skeletal system	FetalCalvaria	Fetal mesenchymal progenitor	Mesenchymal stem cell	CL:0000134	Vault of skull	UBERON:0004339	0.284934973	0.482605152	0.515129151	0.594634394	0.980334498	0.225749975	NA
Fetal_HCA_Thymus_Thymocytes	Fetal	Lymphatic system	Fetal_HCA_Thymus	Thymocytes	Thymocyte	CL:0000893	Thymus	UBERON:0002370	0.325447902	0.469595152	0.531365314	0.595916835	0.980334498	0.224814345	NA
TS_Liver_t cell	Adult	Digestive system	TS_Liver	t cell	T cell	CL:0000084	Liver	UBERON:0002107	0.252910982	0.496644425	0.503690037	0.596788208	0.980334498	0.224179766	NA
NeonatalAdrenalGland_Smooth muscle cell	Neonatal	Endocrine system	NeonatalAdrenalGland	Smooth muscle cell	Smooth muscle cell	CL:0000192	Adrenal gland	UBERON:0002369	0.312194458	0.476388348	0.528413284	0.598965813	0.980334498	0.222597965	NA
TS_Thymus_b cell	Adult	Lymphatic system	TS_Thymus	b cell	B cell	CL:0000236	Thymus	UBERON:0002370	0.07691635	0.608011678	0.41402214	0.598966316	0.980334498	0.2225976	NA
FetalKidney_Fetal epithelial progenitor	Fetal	Urinary system	FetalKidney	Fetal epithelial progenitor	Epithelial fate stem cell	CL:0000036	Kidney	UBERON:0002113	0.302647022	0.483058032	0.523616236	0.600627991	0.980334498	0.221394432	NA
FetalHeart_Endothelial cell	Fetal	Cardiovascular system	FetalHeart	Endothelial cell	Endothelial cell	CL:0000115	Heart	UBERON:0000948	0.259295474	0.503437622	0.50701107	0.603794772	0.980334498	0.219101651	NA
CordBlood_Erythroid cell	Neonatal	Reproductive system	CordBlood	Erythroid cell	Erythroid lineage cell	CL:0000764	Umbilical cord blood	UBERON:0012168	0.337373868	0.477458791	0.535055351	0.604092954	0.980334498	0.21889623	NA
AdultPeripheralBlood_T cell	Adult	Lymphatic system	AdultPeripheralBlood	T cell	T cell	CL:0000084	Blood	UBERON:0000178	0.337725572	0.477047082	0.535793358	0.604272751	0.980334498	0.218766989	NA
AdultAdrenalGland_Myeloid cell	Adult	Endocrine system	AdultAdrenalGland	Myeloid cell	Myeloid cell	CL:0000763	Adrenal gland	UBERON:0002369	0.282615378	0.502161324	0.511439114	0.605942647	0.980334498	0.21756848	NA
Braga_2020_lung_Alveolar cells type 1	Adult	Respiratory system	Braga_2020_lung	Alveolar cells type 1	Alveolar capillary type 1 endothelial cell	CL:4028002	Lung	UBERON:0002048	0.296530676	0.496191545	0.521771218	0.608752965	0.980334498	0.215558911	NA
TS_Trachea_cd4-positive, alpha-beta t cell	Adult	Respiratory system	TS_Trachea	cd4-positive, alpha-beta t cell	T cell	CL:0000084	Trachea	UBERON:0003126	0.119383747	0.598336519	0.435424354	0.610992951	0.980334498	0.213992233	NA
Madissoon_2020_lung_Ciliated	Adult	Respiratory system	Madissoon_2020_lung	Ciliated	Ciliated cell	CL:0000064	Lung	UBERON:0002048	0.31293782	0.494427197	0.529151292	0.612421219	0.980334498	0.212949771	NA
TS_Small_Intestine_mature enterocyte	Adult	Digestive system	TS_Small_Intestine	mature enterocyte	Enterocyte	CL:0000584	Small intestine	UBERON:0002108	0.446147143	0.459878822	0.570479705	0.613396237	0.980334498	0.212258893	NA
FetalKidney_Ureteric bud cell	Fetal	Urinary system	FetalKidney	Ureteric bud cell	Epithelial cell	CL:0000066	Kidney	UBERON:0002113	0.302647022	0.504466894	0.523616236	0.61579264	0.980334498	0.210566506	NA
AdultLung_Macrophage	Adult	Respiratory system	AdultLung	Macrophage	Macrophage	CL:0000235	Lung	UBERON:0002048	0.307515925	0.503643476	0.525830258	0.61670227	0.980334498	0.209924483	NA
FetalMuscle_Fetal neuron	Fetal	Muscular system	FetalMuscle	Fetal neuron	Neuron	CL:0000540	Muscle organ	UBERON:0001630	0.302664848	0.506648951	0.524723247	0.618054845	0.980334498	0.208972985	NA
van_Zyl_eye_2020_Melanocyte	Adult	Sensory system	van_Zyl_eye_2020	Melanocyte	Melanocyte	CL:0000148	Eye	UBERON:0000970	0.31334405	0.501996641	0.529889299	0.618256393	0.980334498	0.208831384	NA
FetalBrain_Erythroid cell	Fetal	Nervous system	FetalBrain	Erythroid cell	Erythroid lineage cell	CL:0000764	Brain	UBERON:0000955	0.332947615	0.498044235	0.534317343	0.618403399	0.980334498	0.208728136	NA
AdultUterus_Stromal cell	Adult	Reproductive system	AdultUterus	Stromal cell	Stromal cell	CL:0000499	Uterus	UBERON:0000995	0.316396099	0.501749615	0.530627306	0.618573309	0.980334498	0.208608824	NA
AdultStomach_Endothelial cell (APC)	Adult	Digestive system	AdultStomach	Endothelial cell (APC)	Endothelial cell	CL:0000115	Stomach	UBERON:0000945	0.282949858	0.521635154	0.512177122	0.619799161	0.980334498	0.207749016	NA

TS_Muscle_slow muscle cell	Adult	Muscular system	TS_Muscle	slow muscle cell	Cell of skeletal muscle	CL:0000188	Muscle organ	UBERON:0001630	0.119537665	0.611634716	0.436900369	0.619870219	0.980334498	0.207699228	NA
AdultTransverseColon_Stromal cell	Adult	Digestive system	AdultTransverseColon	Stromal cell	Stromal cell	CL:0000499	Transverse colon	UBERON:0001157	0.287177546	0.519164901	0.517343173	0.621665439	0.980334498	0.206443276	NA
Fetal_HCA_Pancreas_Stromal cells	Fetal	Digestive system	Fetal_HCA_Pancreas	Stromal cells	Stromal cell	CL:0000499	Pancreas	UBERON:0001264	0.28728027	0.51957661	0.518081181	0.622448855	0.980334498	0.205896328	NA
Fetal_HCA_Heart_Vascular endothelial cells	Fetal	Cardiovascular system	Fetal_HCA_Heart	Vascular endothelial cells	Endothelial cell	CL:0000115	Heart	UBERON:0000948	0.292266853	0.521593983	0.520295203	0.625327069	0.980334498	0.203892771	NA
CordBloodCD34P_Monocyte	Neonatal	Reproductive system	CordBloodCD34P	Monocyte	Monocyte	CL:0000576	Umbilical cord blood	UBERON:0012168	0.289173506	0.525834585	0.518819188	0.627188642	0.980334498	0.202601815	NA
Lake_2017_FrontalCortex_In1b	Adult	Nervous system	Lake_2017_FrontalCortex	In1b	Inhibitory neuron	CL:0008029	Frontal cortex	UBERON:0001870	0.283162333	0.531763193	0.514391144	0.628124512	0.980334498	0.201954259	NA
AdultSigmoidColon_T cell	Adult	Digestive system	AdultSigmoidColon	T cell	T cell	CL:0000084	Sigmoid colon	UBERON:0001159	0.354441679	0.508131103	0.540221402	0.629378965	0.980334498	0.201087776	NA
FetalPancreas_Smooth muscle cell	Fetal	Digestive system	FetalPancreas	Smooth muscle cell	Smooth muscle cell	CL:0000192	Pancreas	UBERON:0001264	0.327748992	0.521141104	0.532472325	0.633228083	0.980334498	0.198439833	NA
AdultIleum_Endothelial cell (APC)	Adult	Digestive system	AdultIleum	Endothelial cell (APC)	Endothelial cell	CL:0000115	Ileum	UBERON:0002116	0.277286239	0.551401707	0.508856089	0.63717334	0.980334498	0.195742404	NA
Lake_2017_VisualCortex_In3	Adult	Nervous system	Lake_2017_VisualCortex	In3	Inhibitory neuron	CL:0008029	Visual cortex	UBERON:0000411	0.283088312	0.556424555	0.513284133	0.643508248	0.980334498	0.191445882	NA
Voigt_2019_retina_2_Artery	Adult	Sensory system	Voigt_2019_retina_2	Artery	Endothelial cell	CL:0000115	Retina	UBERON:0000966	0.29700691	0.555148257	0.522509225	0.649070312	0.980334498	0.187708255	NA
TS_Uterus_ciliated epithelial cell	Adult	Reproductive system	TS_Uterus	ciliated epithelial cell	Ciliated cell	CL:0000064	Uterus	UBERON:0000995	0.177550506	0.619786552	0.470110701	0.650674112	0.980334498	0.186636472	NA
AdultEpityphlon_T cell	Adult	Digestive system	AdultEpityphlon	T cell	T cell	CL:0000084	Caecum	UBERON:0001153	0.400291262	0.530075186	0.553505535	0.65317193	0.980334498	0.184972487	NA
TS_Lung_classical monocyte	Adult	Respiratory system	TS_Lung	classical monocyte	Monocyte	CL:0000576	Lung	UBERON:0002048	0.252649942	0.588867215	0.502214022	0.656029326	0.980334498	0.183076746	NA
Lake_2017_VisualCortex_In1c	Adult	Nervous system	Lake_2017_VisualCortex	In1c	Inhibitory neuron	CL:0008029	Visual cortex	UBERON:0000411	0.283088312	0.576845315	0.513284133	0.656453297	0.980334498	0.182796166	NA
TS_Trachea_t cell	Adult	Respiratory system	TS_Trachea	t cell	T cell	CL:0000084	Trachea	UBERON:0003126	0.177528029	0.636913641	0.469372694	0.659925675	0.980334498	0.180504975	NA
AdultAdrenalGland_Stromal cell	Adult	Endocrine system	AdultAdrenalGland	Stromal cell	Stromal cell	CL:0000499	Adrenal gland	UBERON:0002369	0.380816702	0.550331263	0.544649446	0.660875826	0.980334498	0.179880134	NA
AdultThyroid_Smooth muscle cell	Adult	Endocrine system	AdultThyroid	Smooth muscle cell	Smooth muscle cell	CL:0000192	Thyroid gland	UBERON:0002046	0.375649287	0.554448352	0.543173432	0.662588147	0.980334498	0.178756337	NA
AdultArtery_Macrophage	Adult	Cardiovascular system	AdultArtery	Macrophage	Macrophage	CL:0000235	Artery	UBERON:0001637	0.307594654	0.571863638	0.526937269	0.662797695	0.980334498	0.178619011	NA
Fetal_HCA_Adrenal_Megakaryocytes	Fetal	Endocrine system	Fetal_HCA_Adrenal	Megakaryocytes	Megakaryocyte	CL:0000556	Adrenal gland	UBERON:0002369	0.292118933	0.580344841	0.519557196	0.663020839	0.980334498	0.178472821	NA
AdultEsophagus_Endothelial cell	Adult	Digestive system	AdultEsophagus	Endothelial cell	Endothelial cell	CL:0000115	Esophagus	UBERON:0001043	0.365258261	0.559471201	0.541328413	0.664618816	0.980334498	0.177427368	NA
FetalPancreas_Endothelial cell	Fetal	Digestive system	FetalPancreas	Endothelial cell	Endothelial cell	CL:0000115	Pancreas	UBERON:0001264	0.327748992	0.56964041	0.532472325	0.665168048	0.980334498	0.177068621	NA
TS_Skin_melanocyte	Adult	Integumentary system	TS_Skin	melanocyte	Melanocyte	CL:0000148	Skin of body	UBERON:0002097	0.343359717	0.567499524	0.539483395	0.668541439	0.980334498	0.174871668	NA
AdultAdipose_Fibroblast	Adult	Integumentary system	AdultAdipose	Fibroblast	Fibroblast	CL:0000057	Adipose tissue	UBERON:0001013	0.43050059	0.54415563	0.563099631	0.668845845	0.980334498	0.174673966	NA
FetalMuscle_Primordial germ cell	Fetal	Muscular system	FetalMuscle	Primordial germ cell	Primordial germ cell	CL:0000670	Muscle organ	UBERON:0001630	0.402882051	0.558194903	0.554612546	0.672576766	0.980334498	0.172258139	NA
TS_Bladder_plasma cell	Adult	Urinary system	TS_Bladder	plasma cell	Plasma cell	CL:0000786	Bladder organ	UBERON:0018707	0.251494787	0.620939337	0.499630996	0.673348347	0.980334498	0.171760202	NA
FetalSpinalCord_B cell	Fetal	Nervous system	FetalSpinalCord	B cell	B cell	CL:0000236	Spinal cord	UBERON:0002240	0.41964695	0.555930504	0.559778598	0.674467487	0.980334498	0.171038981	NA
Madissoon_2020_lung_T_CD8_CytT	Adult	Respiratory system	Madissoon_2020_lung	T_CD8_CytT	T cell	CL:0000084	Lung	UBERON:0002048	0.414045113	0.561570916	0.557933579	0.676936491	0.980334498	0.169452074	NA
AdultKidney_Intercalated cell	Adult	Urinary system	AdultKidney	Intercalated cell	Renal intercalated cell	CL:0005010	Kidney	UBERON:0002113	0.381482596	0.5714931	0.548339483	0.676997948	0.980334498	0.169412647	NA
TS_Bone_Marrow_plasma cell	Adult	Lymphatic system	TS_Bone_Marrow	plasma cell	Plasma cell	CL:0000786	Bone marrow	UBERON:0002371	0.177656458	0.664456966	0.47195572	0.67725551	0.980334498	0.169247453	NA
Fetal_HCA_Stomach_Ciliated epithelial cells	Fetal	Digestive system	Fetal_HCA_Stomach	Ciliated epithelial cells	Ciliated cell	CL:0000064	Stomach	UBERON:0000945	0.433250992	0.556465726	0.564206642	0.677681344	0.980334498	0.16897447	NA
AdultEsophagus_Neutrophil	Adult	Digestive system	AdultEsophagus	Neutrophil	Neutrophil	CL:0000775	Esophagus	UBERON:0001043	0.365258261	0.580386012	0.541328413	0.677933564	0.980334498	0.168812864	NA
Fetal_HCA_Stomach_Squamous epithelial cells	Fetal	Digestive system	Fetal_HCA_Stomach	Squamous epithelial cells	Epithelial cell	CL:0000066	Stomach	UBERON:0000945	0.433250992	0.56268253	0.564206642	0.68172528	0.980334498	0.166390601	NA
FetalKidney_Loop of Henle	Fetal	Urinary system	FetalKidney	Loop of Henle	Kidney loop of Henle epithelial cell	CL:1000909	Kidney	UBERON:0002113	0.402909897	0.571904809	0.556088561	0.68236789	0.980334498	0.165981418	NA
TS_Prostate_epithelial cell	Adult	Reproductive system	TS_Prostate	epithelial cell	Epithelial cell	CL:0000066	Prostate gland	UBERON:0002367	0.177755085	0.671826555	0.474538745	0.683258234	0.980334498	0.165415126	NA
FetalIntestine_Hepatocyte/Endodermal cell	Fetal	Digestive system	FetalIntestine	Hepatocyte/Endodermal cell	Hepatocyte	CL:0000182	Intestine	UBERON:0000160	0.380726898	0.586396962	0.543911439	0.683418572	0.980334498	0.165313223	NA
AdultTrachea_Fibroblast	Adult	Respiratory system	AdultTrachea	Fibroblast	Fibroblast	CL:0000057	Trachea	UBERON:0003126	0.386081456	0.58120943	0.549077491	0.683625354	0.980334498	0.165181839	NA
AdultBoneMarrow_Erythroid cell	Adult	Lymphatic system	AdultBoneMarrow	Erythroid cell	Erythroid lineage cell	CL:0000764	Bone marrow	UBERON:0002371	0.387237463	0.585079493	0.550184502	0.686780159	0.980334498	0.16318226	NA
FetalKidney_Intercalated cell	Fetal	Urinary system	FetalKidney	Intercalated cell	Renal intercalated cell	CL:0005010	Kidney	UBERON:0002113	0.402909897	0.578986202	0.556088561	0.686854889	0.980334498	0.163135006	NA
Fetal_HCA_Cerebrum_10k_Microglia	Fetal	Nervous system	Fetal_HCA_Cerebrum_10k	Microglia	Microglial cell	CL:0000129	Cerebral hemisphere	UBERON:0001869	0.293169827	0.619992406	0.52103321	0.688064653	0.980334498	0.162370752	NA
AdultBoneMarrow_Erythroid progenitor cell (RP high)	Adult	Lymphatic system	AdultBoneMarrow	Erythroid progenitor cell (RP high)	Erythroid progenitor cell	CL:0000038	Bone marrow	UBERON:0002371	0.387237463	0.591337468	0.550184502	0.690664529	0.980334498	0.160732848	NA
FetalSpinalCord_Fetal mesenchymal progenitor	Fetal	Nervous system	FetalSpinalCord	Fetal mesenchymal progenitor	Mesenchymal stem cell	CL:0000134	Spinal cord	UBERON:0002240	0.41964695	0.583762025	0.559778598	0.692270201	0.980334498	0.159724362	NA
Fetal_HCA_Spleen_Lymphoid cells	Fetal	Lymphatic system	Fetal_HCA_Spleen	Lymphoid cells	Innate lymphoid cell	CL:0001065	Spleen	UBERON:0002106	0.439807293	0.577380537	0.567158672	0.693039983	0.980334498	0.159241709	NA
AdultDuodenum_T cell	Adult	Digestive system	AdultDuodenum	T cell	T cell	CL:0000084	Duodenum	UBERON:0002114	0.454609754	0.570257973	0.575645756	0.69393233	0.980334498	0.158682878	NA
FetalAdrenalGland_Fetal endocrine cell	Fetal	Endocrine system	FetalAdrenalGland	Fetal endocrine cell	Endocrine cell	CL:0000163	Adrenal gland	UBERON:0002369	0.430289577	0.58491481	0.561623616	0.694194452	0.980334498	0.158518862	NA
TS_Fat_nk cell	Adult	Integumentary system	TS_Fat	nk cell	Natural killer cell	CL:0000623	Perirenal fat	UBERON:0005406	0.343228458	0.609987881	0.538745387	0.6943349	0.980334498	0.158431005	NA
TS_Salivary_Gland_b cell	Adult	Digestive system	TS_Salivary_Gland	b cell	B cell	CL:0000236	Saliva-secreting gland	UBERON:0001044	0.252915817	0.651323452	0.504797048	0.694510687	0.980334498	0.158321067	NA
Fetal_HCA_Spleen_Myeloid cells	Fetal	Lymphatic system	Fetal_HCA_Spleen	Myeloid cells	Myeloid cell	CL:0000763	Spleen	UBERON:0002106	0.439807293	0.580509525	0.567158672	0.69501633	0.980334498	0.158004991	NA
Lake_2017_FrontalCortex_OPC	Adult	Nervous system	Lake_2017_FrontalCortex	OPC	Oligodendrocyte precursor cell	CL:0002453	Frontal cortex	UBERON:0001870	0.381202271	0.602206583	0.547232472	0.695356128	0.980334498	0.157792714	NA
Madissoon_2020_oesophagus_Glands_mucous	Adult	Digestive system	Madissoon_2020_oesophagus	Glands_mucous	Mucus secreting cell	CL:0000319	Esophagus	UBERON:0001043	0.407757658	0.593066645	0.557195572	0.696361807	0.980334498	0.157165056	NA
TS_Bone_Marrow_macrophage	Adult	Lymphatic system	TS_Bone_Marrow	macrophage	Macrophage	CL:0000235	Bone marrow	UBERON:0002371	0.177656458	0.702951746	0.47195572	0.697807541	0.980334498	0.156264341	NA
AdultRectum_T cell	Adult	Digestive system	AdultRectum	T cell	T cell	CL:0000084	Rectum	UBERON:0001052	0.461092809	0.584297246	0.576383764	0.703305619	0.980334498	0.152855913	NA
Fetal_HCA_Lung_Squamous epithelial cells	Fetal	Respiratory system	Fetal_HCA_Lung	Squamous epithelial cells	Epithelial cell	CL:0000066	Lung	UBERON:0002048	0.392605066	0.614393166	0.552767528	0.706381443	0.980334498	0.150960718	NA
Fetal_HCA_Placenta_Trophoblast giant cells	Fetal	Reproductive system	Fetal_HCA_Placenta	Trophoblast giant cells	Trophoblast cell	CL:0000351	Placenta	UBERON:0001987	0.473943317	0.58495598	0.581180812	0.706757731	0.980334498	0.150729432	NA
van_Zyl_eye_2020_BeamCellb	Adult	Sensory system	van_Zyl_eye_2020	BeamCellb	Beam B cell	NA	Eye	UBERON:0000970	0.414558816	0.609082121	0.558671587	0.707093868	0.980334498	0.150522929	NA
Fetal_HCA_Cerebellum_10k_Unipolar brush cells	Fetal	Nervous system	Fetal_HCA_Cerebellum_10k	Unipolar brush cells	Neuron	CL:0000540	Cerebellum	UBERON:0002037	0.500832512	0.576721803	0.591143911	0.707792553	0.980334498	0.150094011	NA
AdultBladder_Fibroblast	Adult	Urinary system	AdultBladder	Fibroblast	Fibroblast	CL:0000057	Bladder organ	UBERON:0018707	0.438255082	0.602494779	0.566051661	0.707919068	0.980334498	0.15001639	NA
VentoTormo_2018_decidua_proliferating decidual natural killer cell (dNK p)	Maternal-Fetal	Reproductive system	VentoTormo_2018_decidua	proliferating decidual natural killer cell (dNK p)	Natural killer cell	CL:0000623	Decidua	UBERON:0002450	0.279965338	0.66791532	0.510701107	0.707985676	0.980334498	0.149975529	NA
FetalHeart_Erythroid cell	Fetal	Cardiovascular system	FetalHeart	Erythroid cell	Erythroid lineage cell	CL:0000764	Heart	UBERON:0000948	0.462609127	0.595001677	0.578597786	0.711371532	0.980334498	0.147903519	NA
FetalAdrenalGland_Smooth muscle cell	Fetal	Endocrine system	FetalAdrenalGland	Smooth muscle cell	Smooth muscle cell	CL:0000192	Adrenal gland	UBERON:0002369	0.430289577	0.619168989	0.561623616	0.715057756	0.980334498	0.145658879	NA
TS_Liver_liver dendritic cell	Adult	Digestive system	TS_Liver	liver dendritic cell	Dendritic cell	CL:0000451	Liver	UBERON:0002107	0.252910982	0.691176872	0.503690037	0.715478967	0.980334498	0.145403129	NA
Lake_2017_FrontalCortex_In6a	Adult	Nervous system	Lake_2017_FrontalCortex	In6a	Inhibitory neuron	CL:0008029	Frontal cortex	UBERON:0001870	0.381202271	0.637160667	0.547232472	0.716044225	0.980334498	0.145060153	NA
Lake_2017_VisualCortex_In6a	Adult	Nervous system	Lake_2017_VisualCortex	In6a	Inhibitory neuron	CL:0008029	Visual cortex	UBERON:0000411	0.381016074	0.640001458	0.545756458	0.716686315	0.980334498	0.144670888	NA
AdultHeart_Smooth muscle cell	Adult	Cardiovascular system	AdultHeart	Smooth muscle cell	Smooth muscle cell	CL:0000192	Heart	UBERON:0000948	0.4908878	0.598130665	0.585608856	0.717721736	0.980334498	0.144043901	NA
FetalAdrenalGland_Fetal mesenchymal progenitor	Fetal	Endocrine system	FetalAdrenalGland	Fetal mesenchymal progenitor	Mesenchymal stem cell	CL:0000134	Adrenal gland	UBERON:0002369	0.430289577	0.62526228	0.561623616	0.718655776	0.980334498	0.14347908	NA
Fetal_HCA_Muscle_Lymphatic endothelial cells	Fetal	Muscular system	Fetal_HCA_Muscle	Lymphatic endothelial cells	Endothelial cell	CL:0000115	Muscle organ	UBERON:0001630	0.461440826	0.608423387	0.577490775	0.718861705	0.980334498	0.143354651	NA
Fetal_HCA_Adrenal_Lymphoid cells	Fetal	Endocrine system	Fetal_HCA_Adrenal	Lymphoid cells	Innate lymphoid cell	CL:0001065	Adrenal gland	UBERON:0002369	0.391223377	0.641854148	0.551291513	0.721457156	0.980334498	0.141789454	NA
Braga_2020_lung_Secretory	Adult	Respiratory system	Braga_2020_lung	Secretory	Club cell	CL:0000158	Lung	UBERON:0002048	0.513682337	0.593190158	0.59704797	0.721783472	0.980334498	0.141593067	NA
Fetal_HCA_Muscle_Myeloid cells	Fetal	Muscular system	Fetal_HCA_Muscle	Myeloid cells	Myeloid cell	CL:0000763	Muscle organ	UBERON:0001630	0.461440826	0.616863419	0.577490775	0.723926043	0.980334498	0.1403058	NA
AdultTrachea_Stromal cell	Adult	Respiratory system	AdultTrachea	Stromal cell	Stromal cell	CL:0000499	Trachea	UBERON:0003126	0.495174507	0.60578845	0.588191882	0.724015743	0.980334498	0.140251991	NA
FetalMuscle_Erythroid cell	Fetal	Muscular system	FetalMuscle	Erythroid cell	Erythroid lineage cell	CL:0000764	Muscle organ	UBERON:0001630	0.512294791	0.599159937	0.594833948	0.724099137	0.980334498	0.14020197	NA
Placenta_Stromal cell	Adult	Reproductive system	Placenta	Stromal cell	Stromal cell	CL:0000499	Placenta	UBERON:0001987	0.467007628	0.614969558	0.579704797	0.724202428	0.980334498	0.140140023	NA
Lake_2017_VisualCortex_In1a	Adult	Nervous system	Lake_2017_VisualCortex	In1a	Inhibitory neuron	CL:0008029	Visual cortex	UBERON:0000411	0.381016074	0.653464339	0.545756458	0.724338118	0.980334498	0.14005866	NA
FetalMuscle_Endothelial cell	Fetal	Muscular system	FetalMuscle	Endothelial cell	Endothelial cell	CL:0000115	Muscle organ	UBERON:0001630	0.402882051	0.648647345	0.554612546	0.727536859	0.980334498	0.138144999	NA
TS_Prostate_nkt cell	Adult	Reproductive system	TS_Prostate	nkt cell	Natural killer cell	CL:0000623	Prostate gland	UBERON:0002367	0.446253347	0.629173514	0.57195572	0.727650767	0.980334498	0.138077008	NA
AdultGallbladder_Macrophage	Adult	Digestive system	AdultGallbladder	Macrophage	Macrophage	CL:0000235	Gall bladder	UBERON:0002110	0.450102086	0.627773704	0.574907749	0.728725007	0.980334498	0.137436327	NA
Placenta_Smooth muscle cell	Adult	Reproductive system	Placenta	Smooth muscle cell	Smooth muscle cell	CL:0000192	Placenta	UBERON:0001987	0.467007628	0.623080223	0.579704797	0.729021075	0.980334498	0.137259917	NA
AdultAscendingColon_Stromal cell	Adult	Digestive system	AdultAscendingColon	Stromal cell	Stromal cell	CL:0000499	Ascending colon	UBERON:0001156	0.514352318	0.605541425	0.598523985	0.730269961	0.980334498	0.136516563	NA
AdultPleura_Stromal cell	Adult	Respiratory system	AdultPleura	Stromal cell	Stromal cell	CL:0000499	Pleura	UBERON:0000977	0.495067031	0.617563324	0.587453875	0.73063404	0.980334498	0.136300098	NA
AdultHeart_Macrophage	Adult	Cardiovascular system	AdultHeart	Macrophage	Macrophage	CL:0000235	Heart	UBERON:0000948	0.4908878	0.62110402	0.585608856	0.731579898	0.980334498	0.135738236	NA
FetalBrain_Macrophage	Fetal	Nervous system	FetalBrain	Macrophage	Macrophage	CL:0000235	Brain	UBERON:0000955	0.435701856	0.644036205	0.565313653	0.731942238	0.980334498	0.13552319	NA
TS_Salivary_Gland_cd8-positive, alpha-beta t cell	Adult	Digestive system	TS_Salivary_Gland	cd8-positive, alpha-beta t cell	T cell	CL:0000084	Saliva-secreting gland	UBERON:0001044	0.44649126	0.634731584	0.574169742	0.732307017	0.980334498	0.135306805	NA
VentoTormo_2018_decidua_CD16-negative natural killer cell (NK CD16-)	Maternal-Fetal	Reproductive system	VentoTormo_2018_decidua	CD16-negative natural killer cell (NK CD16-)	Natural killer cell	CL:0000623	Decidua	UBERON:0002450	0.478684625	0.627773704	0.582287823	0.733417002	0.980334498	0.134649026	NA
AdultArtery_Stromal cell	Adult	Cardiovascular system	AdultArtery	Stromal cell	Stromal cell	CL:0000499	Artery	UBERON:0001637	0.517756144	0.606982406	0.604059041	0.734530605	0.980334498	0.133990104	NA
Fetal_HCA_Placenta_Myeloid cells	Fetal	Reproductive system	Fetal_HCA_Placenta	Myeloid cells	Myeloid cell	CL:0000763	Placenta	UBERON:0001987	0.473943317	0.634608072	0.581180812	0.736700269	0.980334498	0.132709172	NA
TS_Trachea_neutrophil	Adult	Respiratory system	TS_Trachea	neutrophil	Neutrophil	CL:0000775	Trachea	UBERON:0003126	0.343046298	0.689735891	0.537269373	0.738443567	0.980334498	0.131682689	NA
AdultThyroid_Monocyte	Adult	Endocrine system	AdultThyroid	Monocyte	Monocyte	CL:0000576	Thyroid gland	UBERON:0002046	0.484396742	0.636296078	0.58302583	0.738843486	0.980334498	0.131447551	NA
FetalKidney_Erythroid cell	Fetal	Urinary system	FetalKidney	Erythroid cell	Erythroid lineage cell	CL:0000764	Kidney	UBERON:0002113	0.512371393	0.628679464	0.595940959	0.742473252	0.980334498	0.129319187	NA
TS_Lymph_Node_naive thymus-derived cd4-positive, alpha-beta t cell	Adult	Lymphatic system	TS_Lymph_Node	naive thymus-derived cd4-positive, alpha-beta t cell	T cell	CL:0000084	Lymph node	UBERON:0000029	0.555415949	0.612993355	0.617343173	0.746156916	0.980334498	0.127169831	NA
AdultCervix_Macrophage	Adult	Reproductive system	AdultCervix	Macrophage	Macrophage	CL:0000235	Uterine cervix	UBERON:0000002	0.501459519	0.641154243	0.592619926	0.747643979	0.980334498	0.12630516	NA
CordBloodCD34P_Dendritic cell	Neonatal	Reproductive system	CordBloodCD34P	Dendritic cell	Dendritic cell	CL:0000451	Umbilical cord blood	UBERON:0012168	0.514035275	0.638313452	0.597785978	0.749202452	0.980334498	0.12540081	NA
Fetal_HCA_Cerebellum_10k_Vascular endothelial cells	Fetal	Nervous system	Fetal_HCA_Cerebellum_10k	Vascular endothelial cells	Endothelial cell	CL:0000115	Cerebellum	UBERON:0002037	0.391811412	0.694264689	0.55202952	0.750817134	0.980334498	0.124465825	NA
Enge_2017_Pancreas_mesenchymal cell	Adult	Digestive system	Enge_2017_Pancreas	mesenchymal cell	Mesenchymal stem cell	CL:0000134	Pancreas	UBERON:0001264	0.374289774	0.706780639	0.542435424	0.750940114	0.980334498	0.124394696	NA
Madissoon_2020_oesophagus_T_CD4	Adult	Digestive system	Madissoon_2020_oesophagus	T_CD4	T cell	CL:0000084	Esophagus	UBERON:0001043	0.517225046	0.63823111	0.600738007	0.750965835	0.980334498	0.124379821	NA
Madissoon_2020_spleen_T_CD4_reg	Adult	Lymphatic system	Madissoon_2020_spleen	T_CD4_reg	T cell	CL:0000084	Spleen	UBERON:0002106	0.494779128	0.65399956	0.586715867	0.751255453	0.980334498	0.124123363	NA
AdultProstate_Basal cell	Adult	Reproductive system	AdultProstate	Basal cell	Basal cell	CL:0000646	Prostate gland	UBERON:0002367	0.567775044	0.622256805	0.618450185	0.752329412	0.980334498	0.123591959	NA
NeonatalAdrenalGland_Adrenal gland inflammatory cell	Neonatal	Endocrine system	NeonatalAdrenalGland	Adrenal gland inflammatory cell	Inflammatory cell	CL:0009002	Adrenal gland	UBERON:0002369	0.530918625	0.633249432	0.608487085	0.752796321	0.980334498	0.123322512	NA
Lake_2017_FrontalCortex_In3	Adult	Nervous system	Lake_2017_FrontalCortex	In3	Inhibitory neuron	CL:0008029	Frontal cortex	UBERON:0001870	0.489912938	0.663139497	0.584501845	0.754966003	0.980334498	0.122072605	NA
Madissoon_2020_oesophagus_Epi_upper	Adult	Digestive system	Madissoon_2020_oesophagus	Epi_upper	Basal cell	CL:0000646	Esophagus	UBERON:0001043	0.517225046	0.648565003	0.600738007	0.756867117	0.980334498	0.120980363	NA
Fetal_HCA_Heart_Megakaryocytes	Fetal	Cardiovascular system	Fetal_HCA_Heart	Megakaryocytes	Hematopoietic stem cell	CL:0000037	Heart	UBERON:0000948	0.500796569	0.660051681	0.590405904	0.756943233	0.980334498	0.120936689	NA
CordBlood_CB CD34+	Neonatal	Reproductive system	CordBlood	CB CD34+	Hematopoietic stem cell	CL:0000037	Umbilical cord blood	UBERON:0012168	0.576218882	0.630943863	0.620664207	0.758734632	0.980334498	0.119910093	NA
Fetal_HCA_Intestine_Stromal cells	Fetal	Digestive system	Fetal_HCA_Intestine	Stromal cells	Stromal cell	CL:0000499	Intestine	UBERON:0000160	0.495965763	0.665198042	0.588929889	0.758875919	0.980334498	0.119829228	NA
TS_Lung_cd8-positive, alpha-beta t cell	Adult	Respiratory system	TS_Lung	cd8-positive, alpha-beta t cell	T cell	CL:0000084	Lung	UBERON:0002048	0.446138183	0.691135701	0.569372694	0.760520169	0.980334498	0.118889264	NA
FetalKidney_Fetal mesenchymal progenitor	Fetal	Urinary system	FetalKidney	Fetal mesenchymal progenitor	Mesenchymal stem cell	CL:0000134	Kidney	UBERON:0002113	0.512371393	0.66050456	0.595940959	0.76062081	0.980334498	0.118831797	NA
Fetal_HCA_Stomach_Stromal cells	Fetal	Digestive system	Fetal_HCA_Stomach	Stromal cells	Stromal cell	CL:0000499	Stomach	UBERON:0000945	0.547158545	0.650911743	0.610701107	0.764229289	0.980334498	0.116776322	NA
Fetal_HCA_Lung_Ciliated epithelial cells	Fetal	Respiratory system	Fetal_HCA_Lung	Ciliated epithelial cells	Ciliated cell	CL:0000064	Lung	UBERON:0002048	0.50178672	0.670262061	0.593357934	0.764407096	0.980334498	0.11667529	NA
NeonatalAdrenalGland_Fasciculata cell	Neonatal	Endocrine system	NeonatalAdrenalGland	Fasciculata cell	Type II cell of adrenal cortex	CL:0002136	Adrenal gland	UBERON:0002369	0.530918625	0.654370098	0.608487085	0.764840586	0.980334498	0.116429074	NA
AdultBladder_Mast cell	Adult	Urinary system	AdultBladder	Mast cell	Mast cell	CL:0000097	Bladder organ	UBERON:0018707	0.55193048	0.651982187	0.612915129	0.766158516	0.980334498	0.115681367	NA
Fetal_HCA_Liver_Myeloid cells	Fetal	Digestive system	Fetal_HCA_Liver	Myeloid cells	Myeloid cell	CL:0000763	Liver	UBERON:0002107	0.50142395	0.678043359	0.591881919	0.767725086	0.980334498	0.114794268	NA
TS_Vasculature_t cell	Adult	Cardiovascular system	TS_Vasculature	t cell	T cell	CL:0000084	Vasculature	UBERON:0002049	0.446242707	0.704722094	0.571217712	0.768844489	0.980334498	0.114161494	NA
FetalStomach_Endothelial cell	Fetal	Digestive system	FetalStomach	Endothelial cell	Endothelial cell	CL:0000115	Stomach	UBERON:0000945	0.507285138	0.678455068	0.594095941	0.769315193	0.980334498	0.11389569	NA
AdultSigmoidColon_Mast cell	Adult	Digestive system	AdultSigmoidColon	Mast cell	Mast cell	CL:0000097	Sigmoid colon	UBERON:0001159	0.584150102	0.648606174	0.623616236	0.770597494	0.980334498	0.113172408	NA
Madissoon_2020_oesophagus_Epi_stratified	Adult	Digestive system	Madissoon_2020_oesophagus	Epi_stratified	Epithelial cell	CL:0000066	Esophagus	UBERON:0001043	0.517225046	0.67437915	0.600738007	0.771179759	0.980334498	0.112844378	NA
AdultArtery_Mast cell	Adult	Cardiovascular system	AdultArtery	Mast cell	Mast cell	CL:0000097	Artery	UBERON:0001637	0.517756144	0.672238264	0.604059041	0.772033795	0.980334498	0.112363688	NA
AdultBladder_Stromal cell	Adult	Urinary system	AdultBladder	Stromal cell	Stromal cell	CL:0000499	Bladder organ	UBERON:0018707	0.55193048	0.662851301	0.612915129	0.772213982	0.980334498	0.112262339	NA
Fetal_HCA_Stomach_Myeloid cells	Fetal	Digestive system	Fetal_HCA_Stomach	Myeloid cells	Myeloid cell	CL:0000763	Stomach	UBERON:0000945	0.547158545	0.665897947	0.610701107	0.772567801	0.980334498	0.112063397	NA
AdultPeripheralBlood_Neutrophil	Adult	Lymphatic system	AdultPeripheralBlood	Neutrophil	Neutrophil	CL:0000775	Blood	UBERON:0000178	0.576432157	0.657951965	0.621402214	0.774530788	0.980334498	0.110961314	NA
TS_Eye_dendritic cell	Adult	Sensory system	TS_Eye	dendritic cell	Dendritic cell	CL:0000451	Eye	UBERON:0000970	0.446258676	0.71332681	0.573431734	0.774701951	0.980334498	0.11086535	NA
Madissoon_2020_oesophagus_Epi_suprabasal	Adult	Digestive system	Madissoon_2020_oesophagus	Epi_suprabasal	Basal cell	CL:0000646	Esophagus	UBERON:0001043	0.517225046	0.681542884	0.600738007	0.775045473	0.980334498	0.110672816	NA
Madissoon_2020_lung_NK	Adult	Respiratory system	Madissoon_2020_lung	NK	Natural killer cell	CL:0000623	Lung	UBERON:0002048	0.523559742	0.677672821	0.606273063	0.776316456	0.980334498	0.109961208	NA
TS_Skin_t cell	Adult	Integumentary system	TS_Skin	t cell	T cell	CL:0000084	Skin of body	UBERON:0002097	0.446255505	0.718761367	0.572693727	0.77700541	0.980334498	0.109575957	NA
AdultDuodenum_Goblet cell	Adult	Digestive system	AdultDuodenum	Goblet cell	Goblet cell	CL:0000160	Duodenum	UBERON:0002114	0.572596759	0.667380099	0.619188192	0.778425827	0.980334498	0.108782763	NA
AdultAdipose_Macrophage	Adult	Integumentary system	AdultAdipose	Macrophage	Macrophage	CL:0000235	Adipose tissue	UBERON:0001013	0.548852999	0.675531935	0.611808118	0.778480654	0.980334498	0.108752176	NA
Madissoon_2020_lung_DC_activated	Adult	Respiratory system	Madissoon_2020_lung	DC_activated	Dendritic cell	CL:0000451	Lung	UBERON:0002048	0.523559742	0.683724941	0.606273063	0.779563963	0.980334498	0.108148245	NA
FetalThymus_CB CD34+	Fetal	Lymphatic system	FetalThymus	CB CD34+	Hematopoietic stem cell	CL:0000037	Thymus	UBERON:0002370	0.627179669	0.639836775	0.649446494	0.780457202	0.980334498	0.107650907	NA
Fetal_HCA_Adrenal_Myeloid cells	Fetal	Endocrine system	Fetal_HCA_Adrenal	Myeloid cells	Myeloid cell	CL:0000763	Adrenal gland	UBERON:0002369	0.500325483	0.704804436	0.589667897	0.78051059	0.980334498	0.1076212	NA
Madissoon_2020_oesophagus_Endothelia_vascular_venous	Adult	Digestive system	Madissoon_2020_oesophagus	Endothelia_vascular_venous	Endothelial cell	CL:0000115	Esophagus	UBERON:0001043	0.517225046	0.691917948	0.600738007	0.780564022	0.980334498	0.10759147	NA
FetalAdrenalGland_Fasciculata cell	Fetal	Endocrine system	FetalAdrenalGland	Fasciculata cell	Type II cell of adrenal cortex	CL:0002136	Adrenal gland	UBERON:0002369	0.539917771	0.682325131	0.609594096	0.780809702	0.980334498	0.107454799	NA
Fetal_HCA_Muscle_Stromal cells	Fetal	Muscular system	Fetal_HCA_Muscle	Stromal cells	Stromal cell	CL:0000499	Muscle organ	UBERON:0001630	0.574821688	0.676396523	0.619926199	0.783756246	0.980334498	0.105818985	NA
AdultSpleen_B cell	Adult	Lymphatic system	AdultSpleen	B cell	B cell	CL:0000236	Spleen	UBERON:0002106	0.606760908	0.656181617	0.640221402	0.784438291	0.980334498	0.105441215	NA
AdultLiver_Sinusoidal endothelial cell	Adult	Digestive system	AdultLiver	Sinusoidal endothelial cell	Endothelial cell	CL:0000115	Liver	UBERON:0002107	0.584039471	0.677261112	0.622878229	0.785952083	0.980334498	0.104603931	NA
TS_Thymus_dn3 thymocyte	Adult	Lymphatic system	TS_Thymus	dn3 thymocyte	Thymocyte	CL:0000893	Thymus	UBERON:0002370	0.446120286	0.742599312	0.568265683	0.786075054	0.980334498	0.104535986	NA
TS_Lung_dendritic cell	Adult	Respiratory system	TS_Lung	dendritic cell	Dendritic cell	CL:0000451	Lung	UBERON:0002048	0.446138183	0.741323014	0.569372694	0.786157331	0.980334498	0.104490531	NA
AdultRectum_Enterocyte progenitor	Adult	Digestive system	AdultRectum	Enterocyte progenitor	Enterocyte	CL:0000584	Rectum	UBERON:0001052	0.578867512	0.682242789	0.622140221	0.788187707	0.980334498	0.103370343	NA
MacParland_2018_liver_T cell	Adult	Digestive system	MacParland_2018_liver	T cell	T cell	CL:0000084	Liver	UBERON:0002107	0.590530079	0.677590479	0.626568266	0.788278541	0.980334498	0.103320296	NA
FetalRib_Fetal chondrocyte	Fetal	Skeletal system	FetalRib	Fetal chondrocyte	Chondrocyte	CL:0000138	Rib	UBERON:0002228	0.606547954	0.669191618	0.635055351	0.788635607	0.980334498	0.103123618	NA
Madissoon_2020_lung_Macrophage_Dividing	Adult	Respiratory system	Madissoon_2020_lung	Macrophage_Dividing	Macrophage	CL:0000235	Lung	UBERON:0002048	0.523559742	0.70266355	0.606273063	0.789517692	0.980334498	0.102638133	NA
Madissoon_2020_lung_Alveolar_Type2	Adult	Respiratory system	Madissoon_2020_lung	Alveolar_Type2	Alveolar capillary type 2 endothelial cell	CL:4028003	Lung	UBERON:0002048	0.523559742	0.703486968	0.606273063	0.789943382	0.980334498	0.102404035	NA
AdultLung_M2 Macrophage	Adult	Respiratory system	AdultLung	M2 Macrophage	Macrophage	CL:0000235	Lung	UBERON:0002048	0.517672741	0.70776874	0.60295203	0.790152424	0.980334498	0.102289123	NA
Lake_2017_VisualCortex_End	Adult	Nervous system	Lake_2017_VisualCortex	End	Endothelial cell	CL:0000115	Visual cortex	UBERON:0000411	0.489613007	0.732718298	0.583763838	0.790989091	0.980334498	0.101829506	NA
FetalStomach_Fetal epithelial progenitor	Fetal	Digestive system	FetalStomach	Fetal epithelial progenitor	Epithelial fate stem cell	CL:0000036	Stomach	UBERON:0000945	0.618613896	0.660875098	0.64797048	0.791407581	0.980334498	0.101599794	NA
FetalLung_Smooth muscle cell	Fetal	Respiratory system	FetalLung	Smooth muscle cell	Smooth muscle cell	CL:0000192	Lung	UBERON:0002048	0.602191127	0.677755163	0.632103321	0.791563367	0.980334498	0.101514313	NA
MacParland_2018_liver_Erythroid cell	Adult	Digestive system	MacParland_2018_liver	Erythroid cell	Erythroid lineage cell	CL:0000764	Liver	UBERON:0002107	0.590530079	0.683930796	0.626568266	0.791663413	0.980334498	0.101459426	NA
AdultArtery_B cell (Plasmocyte)	Adult	Cardiovascular system	AdultArtery	B cell (Plasmocyte)	B cell	CL:0000236	Artery	UBERON:0001637	0.628341714	0.662439592	0.651660517	0.794326533	0.980334498	0.10000093	NA
Madissoon_2020_spleen_B_T_doublet	Adult	Lymphatic system	Madissoon_2020_spleen	B_T_doublet	B/T doublet	NA	Spleen	UBERON:0002106	0.606689791	0.680678296	0.636900369	0.795867095	0.980334498	0.099159451	NA
Braga_2020_lung_Mast cell	Adult	Respiratory system	Braga_2020_lung	Mast cell	Mast cell	CL:0000097	Lung	UBERON:0002048	0.628959907	0.667133073	0.652767528	0.797499648	0.980334498	0.0982695	NA
FetalRib_Fetal mesenchymal progenitor	Fetal	Skeletal system	FetalRib	Fetal mesenchymal progenitor	Mesenchymal stem cell	CL:0000134	Rib	UBERON:0002228	0.606547954	0.688212568	0.635055351	0.798802183	0.980334498	0.097560757	NA
Voigt_2019_retina_1_Macrophage	Adult	Sensory system	Voigt_2019_retina_1	Macrophage	Macrophage	CL:0000235	Retina	UBERON:0000966	0.597666357	0.697228993	0.628782288	0.799919283	0.980334498	0.096953834	NA
AdultEsophagus_Macrophage	Adult	Digestive system	AdultEsophagus	Macrophage	Macrophage	CL:0000235	Esophagus	UBERON:0001043	0.58643847	0.703651651	0.624723247	0.800892632	0.980334498	0.096425702	NA
AdultPleura_T cell	Adult	Respiratory system	AdultPleura	T cell	T cell	CL:0000084	Pleura	UBERON:0000977	0.606987832	0.687430321	0.641697417	0.802151368	0.980334498	0.095743672	NA
Braga_2020_lung_Ciliated	Adult	Respiratory system	Braga_2020_lung	Ciliated	Ciliated cell	CL:0000064	Lung	UBERON:0002048	0.628959907	0.679237315	0.652767528	0.803996712	0.980334498	0.094745727	NA
FetalLung_Fetal epithelial progenitor	Fetal	Respiratory system	FetalLung	Fetal epithelial progenitor	Epithelial fate stem cell	CL:0000036	Lung	UBERON:0002048	0.602191127	0.704145702	0.632103321	0.805383202	0.980334498	0.093997433	NA
MacParland_2018_liver_Endothelial cell	Adult	Digestive system	MacParland_2018_liver	Endothelial cell	Endothelial cell	CL:0000115	Liver	UBERON:0002107	0.590530079	0.710980069	0.626568266	0.80569445	0.980334498	0.093829628	NA
AdultStomach_Endothelial cell	Adult	Digestive system	AdultStomach	Endothelial cell	Endothelial cell	CL:0000115	Stomach	UBERON:0000945	0.602226594	0.703445797	0.633579336	0.805865155	0.980334498	0.093737622	NA
AdultIleum_Enterocyte	Adult	Digestive system	AdultIleum	Enterocyte	Enterocyte	CL:0000584	Ileum	UBERON:0002116	0.595992952	0.710980069	0.62804428	0.806541782	0.980334498	0.093373129	NA
AdultStomach_Mast cell	Adult	Digestive system	AdultStomach	Mast cell	Mast cell	CL:0000097	Stomach	UBERON:0000945	0.602226594	0.705092632	0.633579336	0.806707143	0.980334498	0.093284098	NA
Fetal_HCA_Eye_Smooth muscle cells	Fetal	Sensory system	Fetal_HCA_Eye	Smooth muscle cells	Smooth muscle cell	CL:0000192	Eye	UBERON:0000970	0.607464149	0.696035037	0.643911439	0.807875444	0.980334498	0.092655592	NA
AdultTransverseColon_Mast cell	Adult	Digestive system	AdultTransverseColon	Mast cell	Mast cell	CL:0000097	Transverse colon	UBERON:0001157	0.606753446	0.702087158	0.638745387	0.80809206	0.980334498	0.09253916	NA
AdultPleura_Mesothelial cell	Adult	Respiratory system	AdultPleura	Mesothelial cell	Mesothelial cell	CL:0000077	Pleura	UBERON:0000977	0.606987832	0.702581208	0.641697417	0.810002048	0.980334498	0.091513883	NA
Madissoon_2020_spleen_Neutrophil	Adult	Lymphatic system	Madissoon_2020_spleen	Neutrophil	Neutrophil	CL:0000775	Spleen	UBERON:0002106	0.606689791	0.709127379	0.636900369	0.810637805	0.980334498	0.091173146	NA
AdultEsophagus_Endothelial cell (APC)	Adult	Digestive system	AdultEsophagus	Endothelial cell (APC)	Endothelial cell	CL:0000115	Esophagus	UBERON:0001043	0.58643847	0.723660703	0.624723247	0.810990644	0.980334498	0.090984156	NA
FetalCalvaria_Endothelial cell	Fetal	Skeletal system	FetalCalvaria	Endothelial cell	Endothelial cell	CL:0000115	Vault of skull	UBERON:0004339	0.616695492	0.700069784	0.647232472	0.811799435	0.980334498	0.090551255	NA
AdultSpleen_Macrophage	Adult	Lymphatic system	AdultSpleen	Macrophage	Macrophage	CL:0000235	Spleen	UBERON:0002106	0.606760908	0.708015765	0.640221402	0.811941061	0.980334498	0.090475495	NA
NeonatalAdrenalGland_Erythroid cell	Neonatal	Endocrine system	NeonatalAdrenalGland	Erythroid cell	Erythroid lineage cell	CL:0000764	Adrenal gland	UBERON:0002369	0.644907751	0.68874779	0.658302583	0.812034096	0.980334498	0.090425735	NA
Fetal_HCA_Thymus_Stromal cells	Fetal	Lymphatic system	Fetal_HCA_Thymus	Stromal cells	Stromal cell	CL:0000499	Thymus	UBERON:0002370	0.671417817	0.678866777	0.670110701	0.813227087	0.980334498	0.089788164	NA
Fetal_HCA_Eye_Microglia	Fetal	Sensory system	Fetal_HCA_Eye	Microglia	Microglial cell	CL:0000129	Eye	UBERON:0000970	0.607464149	0.707892253	0.643911439	0.81393824	0.980334498	0.089408547	NA
AdultUterus_Macrophage	Adult	Reproductive system	AdultUterus	Macrophage	Macrophage	CL:0000235	Uterus	UBERON:0000995	0.649194213	0.68784203	0.663099631	0.814164322	0.980334498	0.089287933	NA
TS_Lymph_Node_cd8-positive, alpha-beta memory t cell	Adult	Lymphatic system	TS_Lymph_Node	cd8-positive, alpha-beta memory t cell	T cell	CL:0000084	Lymph node	UBERON:0000029	0.555415949	0.739676179	0.617343173	0.814577133	0.980334498	0.089067786	NA
AdultTrachea_Macrophage	Adult	Respiratory system	AdultTrachea	Macrophage	Macrophage	CL:0000235	Trachea	UBERON:0003126	0.60710641	0.710568361	0.642804428	0.814673101	0.980334498	0.089016623	NA
AdultLung_Basal cell	Adult	Respiratory system	AdultLung	Basal cell	Basal cell	CL:0000646	Lung	UBERON:0002048	0.628265342	0.703898677	0.650553506	0.815586284	0.980334498	0.088530087	NA
CordBloodCD34P_Erythroid progenitor cell (RP high)	Neonatal	Reproductive system	CordBloodCD34P	Erythroid progenitor cell (RP high)	Erythroid progenitor cell	CL:0000038	Umbilical cord blood	UBERON:0012168	0.634007804	0.698999341	0.656088561	0.816118236	0.980334498	0.088246918	NA
TS_Muscle_cd4-positive, alpha-beta t cell	Adult	Muscular system	TS_Muscle	cd4-positive, alpha-beta t cell	T cell	CL:0000084	Muscle organ	UBERON:0001630	0.662103781	0.687512663	0.667527675	0.816374017	0.980334498	0.088110826	NA
AdultLung_Stromal cell	Adult	Respiratory system	AdultLung	Stromal cell	Stromal cell	CL:0000499	Lung	UBERON:0002048	0.628265342	0.706615955	0.650553506	0.81696357	0.980334498	0.087797309	NA
AdultMuscle_Dendritic cell	Adult	Muscular system	AdultMuscle	Dendritic cell	Dendritic cell	CL:0000451	Muscle organ	UBERON:0001630	0.633730882	0.701551936	0.655350554	0.817018717	0.980334498	0.087767994	NA
FetalKidney_Erythroid progenitor cell (RP high)	Fetal	Urinary system	FetalKidney	Erythroid progenitor cell (RP high)	Erythroid progenitor cell	CL:0000038	Kidney	UBERON:0002113	0.623341189	0.709786114	0.648708487	0.817548022	0.980334498	0.087486728	NA
Fetal_HCA_Heart_Stromal cells	Fetal	Cardiovascular system	Fetal_HCA_Heart	Stromal cells	Stromal cell	CL:0000499	Heart	UBERON:0000948	0.612460825	0.713203297	0.645756458	0.817634374	0.980334498	0.087440859	NA
Madissoon_2020_spleen_DC_activated	Adult	Lymphatic system	Madissoon_2020_spleen	DC_activated	Dendritic cell	CL:0000451	Spleen	UBERON:0002106	0.606689791	0.723948899	0.636900369	0.818043199	0.980334498	0.087223762	NA
TS_Trachea_tracheal goblet cell	Adult	Respiratory system	TS_Trachea	tracheal goblet cell	Goblet cell	CL:0000160	Trachea	UBERON:0003126	0.554906013	0.752109787	0.61402214	0.818606796	0.980334498	0.086924654	NA
FetalAdrenalGland_Fetal epithelial progenitor	Fetal	Endocrine system	FetalAdrenalGland	Fetal epithelial progenitor	Epithelial fate stem cell	CL:0000036	Adrenal gland	UBERON:0002369	0.648947124	0.699164025	0.660885609	0.818804032	0.980334498	0.08682027	NA
AdultTransverseColon_Enterocyte progenitor	Adult	Digestive system	AdultTransverseColon	Enterocyte progenitor	Enterocyte	CL:0000584	Transverse colon	UBERON:0001157	0.606753446	0.723866577	0.638745387	0.819034764	0.980334498	0.086697664	NA
Lake_2017_VisualCortex_In1b	Adult	Nervous system	Lake_2017_VisualCortex	In1b	Inhibitory neuron	CL:0008029	Visual cortex	UBERON:0000411	0.601484341	0.73502868	0.629520295	0.819301551	0.980334498	0.086559223	NA
AdultJejunum_T cell	Adult	Digestive system	AdultJejunum	T cell	T cell	CL:0000084	Jejunum	UBERON:0002115	0.657621775	0.69893487	0.664206642	0.820402012	0.980334498	0.085973283	NA
CordBlood_Neutrophil (RPS high)	Neonatal	Reproductive system	CordBlood	Neutrophil (RPS high)	Neutrophil	CL:0000775	Umbilical cord blood	UBERON:0012168	0.695497459	0.685165023	0.681180812	0.822374101	0.980334498	0.084930576	NA
Lake_2017_FrontalCortex_In4b	Adult	Nervous system	Lake_2017_FrontalCortex	In4b	Inhibitory neuron	CL:0008029	Frontal cortex	UBERON:0001870	0.601873641	0.744781369	0.630627306	0.824618497	0.980334498	0.083746928	NA
AdultUterus_Fibroblast	Adult	Reproductive system	AdultUterus	Fibroblast	Fibroblast	CL:0000057	Uterus	UBERON:0000995	0.649194213	0.709127379	0.663099631	0.825082655	0.980334498	0.083251178	NA
TS_Skin_cd8-positive, alpha-beta cytotoxic t cell	Adult	Integumentary system	TS_Skin	cd8-positive, alpha-beta cytotoxic t cell	T cell	CL:0000084	Skin of body	UBERON:0002097	0.555238114	0.765572667	0.615498155	0.825771555	0.980334498	0.083140081	NA
Lake_2017_FrontalCortex_In7	Adult	Nervous system	Lake_2017_FrontalCortex	In7	Inhibitory neuron	CL:0008029	Frontal cortex	UBERON:0001870	0.601873641	0.747992698	0.630627306	0.826144554	0.980334498	0.082943956	NA
FetalEyes_Endothelial cell	Fetal	Sensory system	FetalEyes	Endothelial cell	Endothelial cell	CL:0000115	Eye	UBERON:0000970	0.639650331	0.720200007	0.656886568	0.827111601	0.980334498	0.082438888	NA
AdultSigmoidColon_B cell (Plasmocyte)	Adult	Digestive system	AdultSigmoidColon	B cell (Plasmocyte)	B cell	CL:0000236	Sigmoid colon	UBERON:0001159	0.69746589	0.693276587	0.684501845	0.828273346	0.980334498	0.081826314	NA
TS_Spleen_plasmacytoid dendritic cell	Adult	Lymphatic system	TS_Spleen	plasmacytoid dendritic cell	Plasmacytoid dendritic cell	CL:0000784	Spleen	UBERON:0002106	0.555029466	0.772036497	0.614760148	0.828532164	0.980334498	0.081799675	NA
FetalSkin_Erythroid cell	Fetal	Integumentary system	FetalSkin	Erythroid cell	Erythroid lineage cell	CL:0000764	Skin of body	UBERON:0002097	0.69003133	0.705874879	0.675276753	0.829842764	0.980334498	0.081004189	NA
TS_Blood_erythrocyte	Adult	Lymphatic system	TS_Blood	erythrocyte	Erythrocyte	CL:0000232	Blood	UBERON:0000178	0.661866391	0.716414627	0.666420664	0.830414607	0.980334498	0.08070502	NA
NeonatalAdrenalGland_Macrophage	Neonatal	Endocrine system	NeonatalAdrenalGland	Macrophage	Macrophage	CL:0000235	Adrenal gland	UBERON:0002369	0.644907751	0.725719247	0.658302583	0.830643214	0.980334498	0.080568479	NA
FetalAdrenalGland_Monocyte	Fetal	Endocrine system	FetalAdrenalGland	Monocyte	Monocyte	CL:0000576	Adrenal gland	UBERON:0002369	0.648947124	0.724731146	0.660885609	0.831543976	0.980334498	0.080114778	NA
NeonatalAdrenalGland_Endothelial cell (APC)	Neonatal	Endocrine system	NeonatalAdrenalGland	Endothelial cell (APC)	Endothelial cell	CL:0000115	Adrenal gland	UBERON:0002369	0.644907751	0.72860121	0.658302583	0.832040883	0.980334498	0.079855334	NA
FetalAdrenalGland_Erythroid progenitor cell (RP high)	Fetal	Endocrine system	FetalAdrenalGland	Erythroid progenitor cell (RP high)	Erythroid progenitor cell	CL:0000038	Adrenal gland	UBERON:0002369	0.648947124	0.728107159	0.660885609	0.833181208	0.980334498	0.079260534	NA
AdultPancreas_Fetal endocrine cell	Adult	Digestive system	AdultPancreas	Fetal endocrine cell	Endocrine cell	CL:0000163	Pancreas	UBERON:0001264	0.69766285	0.702416505	0.68597786	0.83365378	0.980334498	0.079014276	NA
AdultGallbladder_Fibroblast	Adult	Digestive system	AdultGallbladder	Fibroblast	Fibroblast	CL:0000057	Gall bladder	UBERON:0002110	0.674652645	0.71888488	0.670848708	0.833960845	0.980334498	0.078854339	NA
AdultProstate_Endothelial cell (APC)	Adult	Reproductive system	AdultProstate	Endothelial cell (APC)	Endothelial cell	CL:0000115	Prostate gland	UBERON:0002367	0.683024463	0.71748507	0.672324723	0.834083311	0.980334498	0.078808783	NA
AdultBladder_Macrophage	Adult	Urinary system	AdultBladder	Macrophage	Macrophage	CL:0000235	Bladder organ	UBERON:0018707	0.664080351	0.722837285	0.669372694	0.835113807	0.980334498	0.078254339	NA
AdultFallopiantube_Macrophage	Adult	Reproductive system	AdultFallopiantube	Macrophage	Macrophage	CL:0000235	Fallopian tube	UBERON:0003889	0.674938719	0.721437475	0.671586716	0.835592707	0.980334498	0.078005359	NA
AdultEsophagus_Stromal cell	Adult	Digestive system	AdultEsophagus	Stromal cell	Stromal cell	CL:0000499	Esophagus	UBERON:0001043	0.69502497	0.71377968	0.679335793	0.835873898	0.980334498	0.077859236	NA
TS_Tongue_epithelial cell	Adult	Digestive system	TS_Tongue	epithelial cell	Epithelial cell	CL:0000066	Tongue	UBERON:0001723	0.555395154	0.787352067	0.616236162	0.836090029	0.980334498	0.077746956	NA
AdultRectum_B cell	Adult	Digestive system	AdultRectum	B cell	B cell	CL:0000236	Rectum	UBERON:0001052	0.692774594	0.71933776	0.677121771	0.837449212	0.980334498	0.077041522	NA
Fetal_HCA_Cerebellum_10k_Microglia	Fetal	Nervous system	Fetal_HCA_Cerebellum_10k	Microglia	Microglial cell	CL:0000129	Cerebellum	UBERON:0002037	0.612259551	0.756268047	0.64501845	0.837972042	0.980334498	0.076770471	NA
AdultDuodenum_Endothelial cell (APC)	Adult	Digestive system	AdultDuodenum	Endothelial cell (APC)	Endothelial cell	CL:0000115	Duodenum	UBERON:0002114	0.68724723	0.725184026	0.673062731	0.838178333	0.980334498	0.07666357	NA
FetalSkin_Erythroid progenitor cell (RP high)	Fetal	Integumentary system	FetalSkin	Erythroid progenitor cell (RP high)	Erythroid progenitor cell	CL:0000038	Skin of body	UBERON:0002097	0.69003133	0.725595735	0.675276753	0.839625762	0.980334498	0.075960033	NA
Placenta_Erythroid cell	Adult	Reproductive system	Placenta	Erythroid cell	Erythroid lineage cell	CL:0000764	Placenta	UBERON:0001987	0.689385755	0.727983646	0.675276753	0.839909204	0.980334498	0.07576766	NA
Madissoon_2020_lung_Macrophage_MARCOpos	Adult	Respiratory system	Madissoon_2020_lung	Macrophage_MARCOpos	Macrophage	CL:0000235	Lung	UBERON:0002048	0.633726889	0.750257097	0.654243542	0.840147668	0.980334498	0.075644374	NA
AdultJejunum_Macrophage	Adult	Digestive system	AdultJejunum	Macrophage	Macrophage	CL:0000235	Jejunum	UBERON:0002115	0.659257203	0.73926447	0.665313653	0.840852506	0.980334498	0.075280177	NA
Madissoon_2020_lung_Alveolar_Type1	Adult	Respiratory system	Madissoon_2020_lung	Alveolar_Type1	Alveolar capillary type 2 endothelial cell	CL:4028003	Lung	UBERON:0002048	0.633726889	0.752150956	0.654243542	0.841027829	0.980334498	0.075189303	NA
AdultPancreas_Pancreas exocrine cell	Adult	Digestive system	AdultPancreas	Pancreas exocrine cell	Pancreas exocrine glandular cell	CL:1001599	Pancreas	UBERON:0001264	0.69766285	0.71872019	0.68597786	0.841690818	0.980334498	0.074847411	NA
FetalAdrenalGland_Fetal fibroblast	Fetal	Endocrine system	FetalAdrenalGland	Fetal fibroblast	Fibroblast	CL:0000057	Adrenal gland	UBERON:0002369	0.648947124	0.746016495	0.660885609	0.841694653	0.980334498	0.074845431	NA
AdultLiver_T cell	Adult	Digestive system	AdultLiver	T cell	T cell	CL:0000084	Liver	UBERON:0002107	0.697291957	0.722960798	0.68302583	0.842238112	0.980334498	0.074565112	NA
AdultSigmoidColon_B cell	Adult	Digestive system	AdultSigmoidColon	B cell	B cell	CL:0000236	Sigmoid colon	UBERON:0001159	0.69746589	0.721478646	0.684501845	0.842275201	0.980334498	0.074545986	NA
AdultRectum_Epithelial cell	Adult	Digestive system	AdultRectum	Epithelial cell	Epithelial cell	CL:0000066	Rectum	UBERON:0001052	0.692774594	0.73200674	0.677121771	0.842687939	0.980334498	0.074333222	NA
TS_Muscle_mature nk t cell	Adult	Muscular system	TS_Muscle	mature nk t cell	Natural killer cell	CL:0000623	Muscle organ	UBERON:0001630	0.662103781	0.74206409	0.667527675	0.843327584	0.980334498	0.074003694	NA
AdultJejunum_Enterocyte	Adult	Digestive system	AdultJejunum	Enterocyte	Enterocyte	CL:0000584	Jejunum	UBERON:0002115	0.659257203	0.744987225	0.665313653	0.843539542	0.980334498	0.073894554	NA
AdultPeripheralBlood_Monocyte	Adult	Lymphatic system	AdultPeripheralBlood	Monocyte	Monocyte	CL:0000576	Blood	UBERON:0000178	0.695617996	0.728642381	0.681918819	0.844397686	0.980334498	0.073452966	NA
Fetal_HCA_Cerebrum_10k_Vascular endothelial cells	Fetal	Nervous system	Fetal_HCA_Cerebrum_10k	Vascular endothelial cells	Endothelial cell	CL:0000115	Cerebral hemisphere	UBERON:0001869	0.612657642	0.772036497	0.646494465	0.845960985	0.980334498	0.072649666	NA
AdultLiver_Monocyte	Adult	Digestive system	AdultLiver	Monocyte	Monocyte	CL:0000576	Liver	UBERON:0002107	0.697291957	0.732553615	0.68302583	0.846818175	0.980334498	0.072209829	NA
AdultEsophagus_Mast cell	Adult	Digestive system	AdultEsophagus	Mast cell	Mast cell	CL:0000097	Esophagus	UBERON:0001043	0.69502497	0.736670704	0.679335793	0.846683073	0.980334498	0.072176548	NA
AdultEsophagus_T cell	Adult	Digestive system	AdultEsophagus	T cell	T cell	CL:0000084	Esophagus	UBERON:0001043	0.69502497	0.737617634	0.679335793	0.847327975	0.980334498	0.071948555	NA
TS_Uterus_t cell	Adult	Reproductive system	TS_Uterus	t cell	T cell	CL:0000084	Uterus	UBERON:0000995	0.662250276	0.749680704	0.668634686	0.847441712	0.980334498	0.071887396	NA
AdultEsophagus_Dendritic cell	Adult	Digestive system	AdultEsophagus	Dendritic cell	Dendritic cell	CL:0000451	Esophagus	UBERON:0001043	0.69502497	0.739140957	0.679335793	0.848041956	0.980334498	0.071582661	NA
AdultKidney_Ureteric bud cell	Adult	Urinary system	AdultKidney	Ureteric bud cell	Epithelial cell	CL:0000066	Kidney	UBERON:0002113	0.709096597	0.7207357	0.697785978	0.848589462	0.980334498	0.071302366	NA
Voigt_2019_retina_1_Melanocyte	Adult	Sensory system	Voigt_2019_retina_1	Melanocyte	Melanocyte	CL:0000148	Retina	UBERON:0000966	0.704941627	0.727530767	0.69298893	0.849447199	0.980334498	0.070863611	NA
FetalThymus_T cell	Fetal	Lymphatic system	FetalThymus	T cell	T cell	CL:0000084	Thymus	UBERON:0002370	0.744665942	0.701304911	0.72398524	0.851875156	0.980334498	0.069624047	NA
MacParland_2018_liver_Cholangiocytes	Adult	Digestive system	MacParland_2018_liver	Cholangiocytes	Cholangiocyte	CL:1000488	Liver	UBERON:0002107	0.698646818	0.740417255	0.687453875	0.852733227	0.980334498	0.069186814	NA
MacParland_2018_liver_NK-like cell	Adult	Digestive system	MacParland_2018_liver	NK-like cell	Natural killer cell	CL:0000623	Liver	UBERON:0002107	0.698646818	0.741364185	0.687453875	0.853172413	0.980334498	0.068963196	NA
FetalSkin_Endothelial cell	Fetal	Integumentary system	FetalSkin	Endothelial cell	Endothelial cell	CL:0000115	Skin of body	UBERON:0002097	0.69003133	0.754744724	0.675276753	0.853177727	0.980334498	0.068960491	NA
AdultOmentum_Fibroblast	Adult	Digestive system	AdultOmentum	Fibroblast	Fibroblast	CL:0000057	Omentum	UBERON:0003688	0.703444984	0.742228774	0.690405904	0.855042272	0.980334498	0.068012414	NA
AdultHeart_Stromal cell	Adult	Cardiovascular system	AdultHeart	Stromal cell	Stromal cell	CL:0000499	Heart	UBERON:0000948	0.717951619	0.723331336	0.708856089	0.855241884	0.980334498	0.067911039	NA
AdultIleum_Stromal cell	Adult	Digestive system	AdultIleum	Stromal cell	Stromal cell	CL:0000499	Ileum	UBERON:0002116	0.70343965	0.744410831	0.688929889	0.855314716	0.980334498	0.067874056	NA
AdultIleum_Macrophage	Adult	Digestive system	AdultIleum	Macrophage	Macrophage	CL:0000235	Ileum	UBERON:0002116	0.70343965	0.745563616	0.688929889	0.855844443	0.980334498	0.067605165	NA

FetalCalvaria_Erythroid cell	Fetal	Skeletal system	FetalCalvaria	Erythroid cell	Erythroid lineage cell	CL:0000764	Vault of skull	UBERON:0004339	0.725793851	0.721355133	0.712177122	0.855905437	0.980334498	0.067574215	NA
Braga_2020_lung_T cell	Adult	Respiratory system	Braga_2020_lung	T cell	T cell	CL:0000084	Lung	UBERON:0002048	0.736267951	0.715467696	0.718450185	0.856102021	0.980334498	0.067474477	NA
FetalRib_Fetal stromal cell	Fetal	Skeletal system	FetalRib	Fetal stromal cell	Stromal cell	CL:0000499	Rib	UBERON:0002228	0.717027072	0.729918678	0.706642066	0.857272467	0.980334498	0.066881124	NA
Madissoon_2020_lung_T_regulatory	Adult	Respiratory system	Madissoon_2020_lung	T_regulatory	T cell	CL:0000084	Lung	UBERON:0002048	0.736128708	0.71979064	0.716605166	0.857282032	0.980334498	0.066876279	NA
AdultSpleen_CB CD34+	Adult	Lymphatic system	AdultSpleen	CB CD34+	Hematopoietic stem cell	CL:0000037	Spleen	UBERON:0002106	0.717152338	0.730824438	0.707749077	0.8582298	0.980334498	0.06639641	NA
AdultHeart_Endothelial cell (APC)	Adult	Cardiovascular system	AdultHeart	Endothelial cell (APC)	Endothelial cell	CL:0000115	Heart	UBERON:0000948	0.717951619	0.729712824	0.708856089	0.858243681	0.980334498	0.066389385	NA
AdultKidney_T cell	Adult	Urinary system	AdultKidney	T cell	T cell	CL:0000084	Kidney	UBERON:0002113	0.709096597	0.743422729	0.697785978	0.859222823	0.980334498	0.065894196	NA
AdultOmentum_Macrophage	Adult	Integumentary system	AdultOmentum	Macrophage	Macrophage	CL:0000235	Omentum	UBERON:0003688	0.703444984	0.752109787	0.690405904	0.85955801	0.980334498	0.065724808	NA
Voigt_2019_retina_1_B cell	Adult	Sensory system	Voigt_2019_retina_1	B cell	B cell	CL:0000236	Retina	UBERON:0000966	0.704941627	0.750010072	0.69298893	0.859877355	0.980334498	0.065563488	NA
AdultAdrenalGland_Endothelial cell	Adult	Endocrine system	AdultAdrenalGland	Endothelial cell	Endothelial cell	CL:0000115	Adrenal gland	UBERON:0002369	0.70867498	0.751574565	0.695202952	0.861668539	0.980334498	0.064659764	NA
FetalRib_Kidney intercalated cell	Fetal	Skeletal system	FetalRib	Kidney intercalated cell	Renal intercalated cell	CL:0005010	Rib	UBERON:0002228	0.717027072	0.740087887	0.706642066	0.861980148	0.980334498	0.064502736	NA
Madissoon_2020_spleen_ILC	Adult	Lymphatic system	Madissoon_2020_spleen	ILC	Innate lymphoid cell	CL:0001065	Spleen	UBERON:0002106	0.712799883	0.74856909	0.7	0.862641066	0.980334498	0.064169871	NA
AdultKidney_Loop of Henle	Adult	Urinary system	AdultKidney	Loop of Henle	Kidney loop of Henle epithelial cell	CL:1000909	Kidney	UBERON:0002113	0.709096597	0.751492224	0.697785978	0.862888041	0.980334498	0.06404555	NA
Voigt_2019_retina_1_Endothelial cell	Adult	Sensory system	Voigt_2019_retina_1	Endothelial cell	Endothelial cell	CL:0000115	Retina	UBERON:0000966	0.704941627	0.756967952	0.69298893	0.863010449	0.980334498	0.063983946	NA
Fetal_HCA_Eye_Stromal cells	Fetal	Sensory system	Fetal_HCA_Eye	Stromal cells	Stromal cell	CL:0000499	Eye	UBERON:0000970	0.713585726	0.746263521	0.704797048	0.863907919	0.980334498	0.063532545	NA
AdultThyroid_Endothelial cell	Adult	Endocrine system	AdultThyroid	Endothelial cell	Endothelial cell	CL:0000115	Thyroid gland	UBERON:0002046	0.704184271	0.760714502	0.691512915	0.863959043	0.980334498	0.063506845	NA
FetalStomach_Goblet cell	Fetal	Digestive system	FetalStomach	Goblet cell	Goblet cell	CL:0000160	Stomach	UBERON:0000945	0.723240575	0.740293742	0.711439114	0.864363373	0.980334498	0.063303645	NA
FetalLung_Endothelial cell	Fetal	Respiratory system	FetalLung	Endothelial cell	Endothelial cell	CL:0000115	Lung	UBERON:0002048	0.708930283	0.757173806	0.695940959	0.864539201	0.980334498	0.063215309	NA
FetalSpinalCord_Erythroid progenitor cell (RP high)	Fetal	Nervous system	FetalSpinalCord	Erythroid progenitor cell (RP high)	Erythroid progenitor cell	CL:0000038	Spinal cord	UBERON:0002240	0.741141014	0.729630482	0.722878229	0.864850159	0.980334498	0.06305913	NA
Fetal_HCA_Pancreas_Ductal cells	Fetal	Digestive system	Fetal_HCA_Pancreas	Ductal cells	Pancreatic ductal cell	CL:0002079	Pancreas	UBERON:0001264	0.713108071	0.751409882	0.70295203	0.8653429	0.980334498	0.062811766	NA
Madissoon_2020_lung_Plasma_cells	Adult	Respiratory system	Madissoon_2020_lung	Plasma_cells	Plasma cell	CL:0000786	Lung	UBERON:0002048	0.736128708	0.737617634	0.716605166	0.86558244	0.980334498	0.062691562	NA
AdultKidney_Macrophage	Adult	Urinary system	AdultKidney	Macrophage	Macrophage	CL:0000235	Kidney	UBERON:0002113	0.709096597	0.758491275	0.697785978	0.866018069	0.980334498	0.062473047	NA
FetalKidney_Fetal fibroblast	Fetal	Urinary system	FetalKidney	Fetal fibroblast	Fibroblast	CL:0000057	Kidney	UBERON:0002113	0.727274767	0.745810641	0.713284133	0.867735628	0.980334498	0.061612571	NA
Fetal_HCA_Pancreas_Acinar cells	Fetal	Digestive system	Fetal_HCA_Pancreas	Acinar cells	Acinar cell	CL:0000622	Pancreas	UBERON:0001264	0.713108071	0.757214977	0.70295203	0.86793177	0.980334498	0.061514414	NA
Madissoon_2020_spleen_Macrophage	Adult	Lymphatic system	Madissoon_2020_spleen	Macrophage	Macrophage	CL:0000235	Spleen	UBERON:0002106	0.712799883	0.761908458	0.7	0.868592912	0.980334498	0.061183719	NA
AdultTransverseColon_Macrophage	Adult	Digestive system	AdultTransverseColon	Macrophage	Macrophage	CL:0000235	Transverse colon	UBERON:0001157	0.712844375	0.760714502	0.701476015	0.868773279	0.980334498	0.061093545	NA
Braga_2020_lung_Macrophages	Adult	Respiratory system	Braga_2020_lung	Macrophages	Macrophage	CL:0000235	Lung	UBERON:0002048	0.736267951	0.74531659	0.718450185	0.869931913	0.980334498	0.060514737	NA
Fetal_HCA_Intestine_Myeloid cells	Fetal	Digestive system	Fetal_HCA_Intestine	Myeloid cells	Myeloid cell	CL:0000763	Intestine	UBERON:0000160	0.713815525	0.760508648	0.705535055	0.870613286	0.980334498	0.06017471	NA
AdultTransverseColon_Epithelial cell	Adult	Digestive system	AdultTransverseColon	Epithelial cell	Epithelial cell	CL:0000066	Transverse colon	UBERON:0001157	0.712844375	0.767631212	0.701476015	0.871798604	0.980334498	0.059583831	NA
FetalSpinalCord_Monocyte	Fetal	Nervous system	FetalSpinalCord	Monocyte	Monocyte	CL:0000576	Spinal cord	UBERON:0002240	0.741141014	0.745151907	0.722878229	0.871909506	0.980334498	0.059528587	NA
Madissoon_2020_lung_T_CD4	Adult	Respiratory system	Madissoon_2020_lung	T_CD4	T cell	CL:0000084	Lung	UBERON:0002048	0.736128708	0.751698078	0.716605166	0.871919798	0.980334498	0.059523461	NA
AdultTrachea_Endothelial cell (APC)	Adult	Respiratory system	AdultTrachea	Endothelial cell (APC)	Endothelial cell	CL:0000115	Trachea	UBERON:0003126	0.713199292	0.765407984	0.704059041	0.872056897	0.980334498	0.059455179	NA
AdultEpityphlon_B cell	Adult	Digestive system	AdultEpityphlon	B cell	B cell	CL:0000236	Caecum	UBERON:0001153	0.748565561	0.743669755	0.724723247	0.87209544	0.980334498	0.059435984	NA
AdultLung_Fetal mesenchymal progenitor	Adult	Respiratory system	AdultLung	Fetal mesenchymal progenitor	Mesenchymal stem cell	CL:0000134	Lung	UBERON:0002048	0.73147505	0.755650483	0.715129151	0.872979265	0.980334498	0.058996072	NA
Lake_2017_VisualCortex_In2	Adult	Nervous system	Lake_2017_VisualCortex	In2	Inhibitory neuron	CL:0008029	Visual cortex	UBERON:0000411	0.707995452	0.778500326	0.694464945	0.873135225	0.980334498	0.058918491	NA
FetalKidney_Macrophage	Fetal	Urinary system	FetalKidney	Macrophage	Macrophage	CL:0000235	Kidney	UBERON:0002113	0.727274767	0.759150009	0.713284133	0.873656368	0.980334498	0.058659353	NA
FetalSkin_Fetal mesenchymal progenitor	Fetal	Integumentary system	FetalSkin	Fetal mesenchymal progenitor	Mesenchymal stem cell	CL:0000134	Skin of body	UBERON:0002097	0.786953826	0.735394406	0.737638376	0.874247763	0.980334498	0.05836547	NA
FetalAdrenalGland_Primordial germ cell	Fetal	Endocrine system	FetalAdrenalGland	Primordial germ cell	Primordial germ cell	CL:0000670	Adrenal gland	UBERON:0002369	0.749136568	0.745892983	0.727675277	0.874438965	0.980334498	0.058270499	NA
FetalPancreas_Fetal fibroblast	Fetal	Digestive system	FetalPancreas	Fetal fibroblast	Fibroblast	CL:0000057	Pancreas	UBERON:0001264	0.749049105	0.748939628	0.725830258	0.874948667	0.980334498	0.058017426	NA
FetalLiver_Erythroid progenitor cell (RP high)	Fetal	Digestive system	FetalLiver	Erythroid progenitor cell (RP high)	Erythroid progenitor cell	CL:0000038	Liver	UBERON:0002107	0.753623309	0.747869185	0.729151292	0.875986309	0.980334498	0.057502681	NA
Fetal_HCA_Lung_Myeloid cells	Fetal	Respiratory system	Fetal_HCA_Lung	Myeloid cells	Myeloid cell	CL:0000763	Lung	UBERON:0002048	0.718520769	0.769236876	0.710701107	0.876826059	0.980334498	0.057086552	NA
van_Zyl_eye_2020_Pericyte	Adult	Sensory system	van_Zyl_eye_2020	Pericyte	Pericyte cell	CL:0000669	Eye	UBERON:0000970	0.736717941	0.761784946	0.719926199	0.87788867	0.980334498	0.056570353	NA
FetalHeart_Smooth muscle cell	Fetal	Cardiovascular system	FetalHeart	Smooth muscle cell	Smooth muscle cell	CL:0000192	Heart	UBERON:0000948	0.783474377	0.747910356	0.734317343	0.878333624	0.980334498	0.056340492	NA
Madissoon_2020_oesophagus_Mast_cell	Adult	Digestive system	Madissoon_2020_oesophagus	Mast_cell	Mast cell	CL:0000097	Esophagus	UBERON:0001043	0.731086613	0.768783997	0.714391144	0.878338977	0.980334498	0.056337845	NA
Fetal_HCA_Kidney_Lymphoid cells	Fetal	Urinary system	Fetal_HCA_Kidney	Lymphoid cells	Innate lymphoid cell	CL:0001065	Kidney	UBERON:0002113	0.718332374	0.774012699	0.7099631	0.878523452	0.980334498	0.056246641	NA
CordBlood_Erythroid progenitor cell (RP high)	Neonatal	Reproductive system	CordBlood	Erythroid progenitor cell (RP high)	Erythroid progenitor cell	CL:0000038	Umbilical cord blood	UBERON:0012168	0.799888156	0.738935103	0.747601476	0.880257125	0.980334498	0.055390451	NA
van_Zyl_eye_2020_BeamCella	Adult	Sensory system	van_Zyl_eye_2020	BeamCella	Beam A cell	NA	Eye	UBERON:0000970	0.736717941	0.767631212	0.719926199	0.880381022	0.980334498	0.055329328	NA
FetalAdrenalGland_Fetal stromal cell	Fetal	Endocrine system	FetalAdrenalGland	Fetal stromal cell	Stromal cell	CL:0000499	Adrenal gland	UBERON:0002369	0.749136568	0.760879186	0.727675277	0.880993952	0.980334498	0.055027073	NA
FetalMaleGonad_Fetal mesenchymal progenitor	Fetal	Reproductive system	FetalMaleGonad	Fetal mesenchymal progenitor	Mesenchymal stem cell	CL:0000134	Immature gonad	UBERON:2005272	0.740494517	0.768619313	0.721402214	0.88147263	0.980334498	0.054791168	NA
FetalMaleGonad_Endothelial cell	Fetal	Reproductive system	FetalMaleGonad	Endothelial cell	Endothelial cell	CL:0000115	Immature gonad	UBERON:2005272	0.740494517	0.769236876	0.721402214	0.881735178	0.980334498	0.054661832	NA
FetalAdrenalGland_Neutrophil (RPS high)	Fetal	Endocrine system	FetalAdrenalGland	Neutrophil (RPS high)	Neutrophil	CL:0000775	Adrenal gland	UBERON:0002369	0.749136568	0.764625737	0.727675277	0.882598972	0.980334498	0.054236583	NA
FetalPancreas_Endothelial cell (APC)	Fetal	Digestive system	FetalPancreas	Endothelial cell (APC)	Endothelial cell	CL:0000115	Pancreas	UBERON:0001264	0.749049105	0.768331117	0.725830258	0.883347182	0.980334498	0.053868572	NA
FetalSkin_Smooth muscle cell	Fetal	Integumentary system	FetalSkin	Smooth muscle cell	Smooth muscle cell	CL:0000192	Skin of body	UBERON:0002097	0.786953826	0.760796844	0.737638376	0.885388781	0.980334498	0.052865985	NA
AdultRectum_Stromal cell	Adult	Digestive system	AdultRectum	Stromal cell	Stromal cell	CL:0000499	Rectum	UBERON:0001052	0.793178517	0.75688561	0.742435424	0.885819093	0.980334498	0.052654963	NA
AdultSigmoidColon_Enterocyte progenitor	Adult	Digestive system	AdultSigmoidColon	Enterocyte progenitor	Enterocyte	CL:0000584	Sigmoid colon	UBERON:0001159	0.796944032	0.75503292	0.744280443	0.885829206	0.980334498	0.052650005	NA
AdultSigmoidColon_Enterocyte	Adult	Digestive system	AdultSigmoidColon	Enterocyte	Enterocyte	CL:0000584	Sigmoid colon	UBERON:0001159	0.796944032	0.755691654	0.744280443	0.886111558	0.980334498	0.052511599	NA
Fetal_HCA_Placenta_Lymphoid cells	Fetal	Reproductive system	Fetal_HCA_Placenta	Lymphoid cells	Innate lymphoid cell	CL:0001065	Placenta	UBERON:0001987	0.791513207	0.760755673	0.74095941	0.886825226	0.980334498	0.052161962	NA
TS_Muscle_endothelial cell of lymphatic vessel	Adult	Muscular system	TS_Muscle	endothelial cell of lymphatic vessel	Endothelial cell	CL:0000115	Muscle organ	UBERON:0001630	0.759766594	0.774877288	0.729889299	0.887902982	0.980334498	0.051634486	NA
AdultPancreas_Stromal cell	Adult	Digestive system	AdultPancreas	Stromal cell	Stromal cell	CL:0000499	Pancreas	UBERON:0001264	0.797083958	0.760302794	0.745387454	0.888554671	0.980334498	0.051315846	NA
Fetal_HCA_Placenta_Extravillous trophoblasts	Fetal	Reproductive system	Fetal_HCA_Placenta	Extravillous trophoblasts	Trophoblast cell	CL:0000351	Placenta	UBERON:0001987	0.791513207	0.764913933	0.74095941	0.88858307	0.980334498	0.051301965	NA
FetalSkin_Neutrophil	Fetal	Integumentary system	FetalSkin	Neutrophil	Neutrophil	CL:0000775	Skin of body	UBERON:0002097	0.786953826	0.768413459	0.737638376	0.888606394	0.980334498	0.051290566	NA
FetalSkin_Fetal neuron	Fetal	Integumentary system	FetalSkin	Fetal neuron	Neuron	CL:0000540	Skin of body	UBERON:0002097	0.786953826	0.769689756	0.737638376	0.889140099	0.980334498	0.051029803	NA
Fetal_HCA_Placenta_IGFBP1_DKK1 positive cells	Fetal	Reproductive system	Fetal_HCA_Placenta	IGFBP1_DKK1 positive cells	Epithelial cell	CL:0000066	Placenta	UBERON:0001987	0.791513207	0.766313743	0.74095941	0.889171047	0.980334498	0.051014687	NA
Placenta_Epithelial cell	Adult	Reproductive system	Placenta	Epithelial cell	Epithelial cell	CL:0000066	Placenta	UBERON:0001987	0.786414023	0.77170713	0.735793358	0.889176481	0.980334498	0.051012033	NA
AdultEsophagus_B cell	Adult	Digestive system	AdultEsophagus	B cell	B cell	CL:0000236	Esophagus	UBERON:0001043	0.79097291	0.769607414	0.739483395	0.889907875	0.980334498	0.05065495	NA
ChorionicVillus_Epithelial cell	Adult	Reproductive system	ChorionicVillus	Epithelial cell	Epithelial cell	CL:0000066	Chorionic villus	UBERON:0007106	0.782394017	0.777841592	0.733210332	0.890588409	0.980334498	0.050322961	NA
ChorionicVillus_Macrophage	Adult	Reproductive system	ChorionicVillus	Macrophage	Macrophage	CL:0000235	Chorionic villus	UBERON:0007106	0.782394017	0.779817795	0.733210332	0.891400248	0.980334498	0.049927249	NA
AdultLiver_Macrophage	Adult	Digestive system	AdultLiver	Macrophage	Macrophage	CL:0000235	Liver	UBERON:0002107	0.796744466	0.76940156	0.743173432	0.891416086	0.980334498	0.049919533	NA
FetalIntestine_Fetal epithelial progenitor	Fetal	Digestive system	FetalIntestine	Fetal epithelial progenitor	Epithelial fate stem cell	CL:0000036	Intestine	UBERON:0000160	0.801862164	0.765655009	0.751291513	0.893323805	0.980334498	0.048991093	NA
AdultSpleen_T cell	Adult	Lymphatic system	AdultSpleen	T cell	T cell	CL:0000084	Spleen	UBERON:0002106	0.812437484	0.753015546	0.764206642	0.893450599	0.980334498	0.048929456	NA
AdultOmentum_Endothelial cell (APC)	Adult	Integumentary system	AdultOmentum	Endothelial cell (APC)	Endothelial cell	CL:0000115	Omentum	UBERON:0003688	0.797762591	0.77355982	0.746494465	0.894551555	0.980334498	0.048394625	NA
FetalThymus_Erythroid cell	Fetal	Lymphatic system	FetalThymus	Erythroid cell	Erythroid lineage cell	CL:0000764	Thymus	UBERON:0002370	0.842163618	0.730330387	0.791143911	0.894737231	0.980334498	0.048304491	NA
TS_Lung_b cell	Adult	Respiratory system	TS_Lung	b cell	B cell	CL:0000236	Lung	UBERON:0002048	0.759970193	0.791222131	0.731365314	0.895217073	0.980334498	0.048071644	NA
AdultOmentum_Neutrophil	Adult	Integumentary system	AdultOmentum	Neutrophil	Neutrophil	CL:0000775	Omentum	UBERON:0003688	0.797762591	0.776647636	0.746494465	0.895812701	0.980334498	0.047782784	NA
FetalLung_Fetal mesenchymal progenitor	Fetal	Respiratory system	FetalLung	Fetal mesenchymal progenitor	Mesenchymal stem cell	CL:0000134	Lung	UBERON:0002048	0.802165519	0.768413459	0.754612546	0.895862451	0.980334498	0.047758666	NA
TS_Uterus_epithelial cell of uterus	Adult	Reproductive system	TS_Uterus	epithelial cell of uterus	Epithelial cell	CL:0000066	Uterus	UBERON:0000995	0.759943257	0.795668587	0.730627306	0.896668629	0.980334498	0.047368024	NA
FetalIntestine_Fetal enterocyte	Fetal	Digestive system	FetalIntestine	Fetal enterocyte	Enterocyte	CL:0000584	Intestine	UBERON:0000160	0.801862164	0.773848016	0.751291513	0.896694799	0.980334498	0.047355349	NA
FetalCalvaria_Fetal stromal cell	Fetal	Skeletal system	FetalCalvaria	Fetal stromal cell	Stromal cell	CL:0000499	Vault of skull	UBERON:0004339	0.819232032	0.758573616	0.767527675	0.89715019	0.980334498	0.047134846	NA
FetalRib_Endothelial cell	Fetal	Skeletal system	FetalRib	Endothelial cell	Endothelial cell	CL:0000115	Rib	UBERON:0002228	0.812385859	0.763020072	0.763099631	0.897168629	0.980334498	0.047125921	NA
FetalIntestine_Enterocyte progenitor	Fetal	Digestive system	FetalIntestine	Enterocyte progenitor	Enterocyte	CL:0000584	Intestine	UBERON:0000160	0.801862164	0.776524124	0.751291513	0.897781724	0.980334498	0.04682924	NA
FetalIntestine_Fetal stromal cell	Fetal	Digestive system	FetalIntestine	Fetal stromal cell	Stromal cell	CL:0000499	Intestine	UBERON:0000160	0.801862164	0.778088617	0.751291513	0.898413946	0.980334498	0.046523515	NA
TS_Eye_plasma cell	Adult	Sensory system	TS_Eye	plasma cell	Plasma cell	CL:0000786	Eye	UBERON:0000970	0.759994156	0.798632891	0.732103321	0.898473216	0.980334498	0.046494865	NA
FetalLung_Macrophage	Fetal	Respiratory system	FetalLung	Macrophage	Macrophage	CL:0000235	Lung	UBERON:0002048	0.802165519	0.775494851	0.754612546	0.898750108	0.980334498	0.046361045	NA
FetalIntestine_Fetal mesenchymal progenitor	Fetal	Digestive system	FetalIntestine	Fetal mesenchymal progenitor	Mesenchymal stem cell	CL:0000134	Intestine	UBERON:0000160	0.801862164	0.779364915	0.751291513	0.898927955	0.980334498	0.046275113	NA
AdultStomach_T cell	Adult	Digestive system	AdultStomach	T cell	T cell	CL:0000084	Stomach	UBERON:0000945	0.802106333	0.777800421	0.753505535	0.899220302	0.980334498	0.046133897	NA
AdultTrachea_Endothelial cell (endothelial to mesenchymal transition)	Adult	Respiratory system	AdultTrachea	Endothelial cell (endothelial to mesenchymal transition)	Endothelial cell	CL:0000115	Trachea	UBERON:0003126	0.805546511	0.772777573	0.758671587	0.899331114	0.980334498	0.046080381	NA
VentoTormo_2018_decidua_villous cytotrophoblast (VCT)	Maternal-Fetal	Reproductive system	VentoTormo_2018_decidua	villous cytotrophoblast (VCT)	Trophoblast cell	CL:0000351	Decidua	UBERON:0002450	0.784067078	0.797809473	0.735055351	0.899411046	0.980334498	0.046041783	NA
AdultSpleen_Neutrophil	Adult	Lymphatic system	AdultSpleen	Neutrophil	Neutrophil	CL:0000775	Spleen	UBERON:0002106	0.812437484	0.767919408	0.764206642	0.899632378	0.980334498	0.045934923	NA
Fetal_HCA_Eye_Skeletal muscle cells	Fetal	Sensory system	Fetal_HCA_Eye	Skeletal muscle cells	Cell of skeletal muscle	CL:0000188	Eye	UBERON:0000970	0.805913297	0.773271623	0.759409594	0.899835204	0.980334498	0.04583702	NA
AdultTransverseColon_B cell	Adult	Digestive system	AdultTransverseColon	B cell	B cell	CL:0000236	Transverse colon	UBERON:0001157	0.805226576	0.774918459	0.757933579	0.899892065	0.980334498	0.045809578	NA
FetalFemaleGonad_Fetal fibroblast	Fetal	Reproductive system	FetalFemaleGonad	Fetal fibroblast	Fibroblast	CL:0000057	Immature gonad	UBERON:2005272	0.805980461	0.772818744	0.760147601	0.899955695	0.980334498	0.045778871	NA
Madissoon_2020_spleen_T_CD4_conv	Adult	Lymphatic system	Madissoon_2020_spleen	T_CD4_conv	T cell	CL:0000084	Spleen	UBERON:0002106	0.805200002	0.77730637	0.756457565	0.900244278	0.980334498	0.04563963	NA
Fetal_HCA_Heart_ELF3_AGBL2 positive cells	Fetal	Cardiovascular system	Fetal_HCA_Heart	ELF3_AGBL2 positive cells	Endothelial cell	CL:0000115	Heart	UBERON:0000948	0.809281714	0.77355982	0.761623616	0.900860263	0.980334498	0.045342569	NA
Lake_2017_CerebellarHemisphere_End	Adult	Nervous system	Lake_2017_CerebellarHemisphere	End	Endothelial cell	CL:0000115	Cerebellar hemisphere	UBERON:0002245	0.801607179	0.789987004	0.748708487	0.902077095	0.980334498	0.044756344	NA
Lake_2017_CerebellarHemisphere_Mic	Adult	Nervous system	Lake_2017_CerebellarHemisphere	Mic	Microglial cell	CL:0000129	Cerebellar hemisphere	UBERON:0002245	0.801607179	0.790686909	0.748708487	0.902352052	0.980334498	0.04462399	NA
AdultAscendingColon_B cell	Adult	Digestive system	AdultAscendingColon	B cell	B cell	CL:0000236	Ascending colon	UBERON:0001156	0.838216599	0.754085989	0.78597786	0.902719086	0.980334498	0.044447375	NA
Madissoon_2020_spleen_B_mature	Adult	Lymphatic system	Madissoon_2020_spleen	B_mature	B cell	CL:0000236	Spleen	UBERON:0002106	0.805200002	0.78422308	0.756457565	0.90299952	0.980334498	0.044312481	NA
Madissoon_2020_spleen_DC_2	Adult	Lymphatic system	Madissoon_2020_spleen	DC_2	Dendritic cell	CL:0000451	Spleen	UBERON:0002106	0.805200002	0.784552447	0.756457565	0.903129567	0.980334498	0.044249939	NA
AdultCervix_T cell	Adult	Reproductive system	AdultCervix	T cell	T cell	CL:0000084	Uterine cervix	UBERON:0000002	0.809607767	0.779653111	0.762361624	0.903596889	0.980334498	0.044025273	NA
Fetal_HCA_Intestine_Lymphoid cells	Fetal	Digestive system	Fetal_HCA_Intestine	Lymphoid cells	Innate lymphoid cell	CL:0001065	Intestine	UBERON:0000160	0.806020984	0.782658586	0.760885609	0.904186819	0.980334498	0.043741828	NA
AdultBoneMarrow_Neutrophil	Adult	Lymphatic system	AdultBoneMarrow	Neutrophil	Neutrophil	CL:0000775	Bone marrow	UBERON:0002371	0.818885702	0.776771149	0.766789668	0.904241979	0.980334498	0.043715335	NA
FetalMuscle_Smooth muscle cell	Fetal	Muscular system	FetalMuscle	Smooth muscle cell	Smooth muscle cell	CL:0000192	Muscle organ	UBERON:0001630	0.816534394	0.779653111	0.765682657	0.904938292	0.980334498	0.043381035	NA
FetalSpinalCord_Fetal chondrocyte	Fetal	Nervous system	FetalSpinalCord	Fetal chondrocyte	Chondrocyte	CL:0000138	Spinal cord	UBERON:0002240	0.827604048	0.771130737	0.777490775	0.906263817	0.980334498	0.042745359	NA
FetalPancreas_Erythroid cell	Fetal	Digestive system	FetalPancreas	Erythroid cell	Erythroid lineage cell	CL:0000764	Pancreas	UBERON:0001264	0.833644026	0.768289946	0.780442804	0.906294164	0.980334498	0.042730817	NA
Madissoon_2020_oesophagus_NK_T_CD8_Cytotoxic	Adult	Digestive system	Madissoon_2020_oesophagus	NK_T_CD8_Cytotoxic	T cell	CL:0000084	Esophagus	UBERON:0001043	0.81962787	0.780147162	0.768634686	0.906315547	0.980334498	0.04272057	NA
AdultEpityphlon_Enterocyte progenitor	Adult	Digestive system	AdultEpityphlon	Enterocyte progenitor	Enterocyte	CL:0000584	Caecum	UBERON:0001153	0.840534411	0.764131686	0.786715867	0.907084024	0.980334498	0.042352482	NA
AdultLung_Stratified epithelial cell	Adult	Respiratory system	AdultLung	Stratified epithelial cell	Epithelial cell	CL:0000066	Lung	UBERON:0002048	0.819943562	0.781258776	0.769741697	0.907192349	0.980334498	0.042300621	NA
AdultUreter_B cell (Plasmocyte)	Adult	Urinary system	AdultUreter	B cell (Plasmocyte)	B cell	CL:0000236	Uterus	UBERON:0000995	0.851671753	0.756803268	0.794833948	0.907276477	0.980334498	0.042260349	NA
AdultTemporalLobe_Smooth muscle cell	Adult	Nervous system	AdultTemporalLobe	Smooth muscle cell	Smooth muscle cell	CL:0000192	Temporal lobe	UBERON:0001871	0.830451732	0.772818744	0.778597786	0.907368973	0.980334498	0.042216075	NA
FetalMuscle_Macrophage	Fetal	Muscular system	FetalMuscle	Macrophage	Macrophage	CL:0000235	Muscle organ	UBERON:0001630	0.816534394	0.7870227	0.765682657	0.907822774	0.980334498	0.041998926	NA
FetalSpinalCord_Macrophage	Fetal	Nervous system	FetalSpinalCord	Macrophage	Microglial cell	CL:0000129	Spinal cord	UBERON:0002240	0.827604048	0.776894662	0.777490775	0.908539707	0.980334498	0.041656087	NA
Madissoon_2020_lung_Mast_cells	Adult	Respiratory system	Madissoon_2020_lung	Mast_cells	Mast cell	CL:0000097	Lung	UBERON:0002048	0.823560245	0.782535073	0.77195572	0.908566925	0.980334498	0.041643077	NA
Madissoon_2020_lung_B_cell_mature	Adult	Respiratory system	Madissoon_2020_lung	B_cell_mature	B cell	CL:0000236	Lung	UBERON:0002048	0.823560245	0.785911086	0.77195572	0.909874918	0.980334498	0.041018307	NA
Madissoon_2020_lung_Basal	Adult	Respiratory system	Madissoon_2020_lung	Basal	Basal cell	CL:0000646	Lung	UBERON:0002048	0.823560245	0.786734504	0.77195572	0.910192241	0.980334498	0.040866871	NA
Madissoon_2020_oesophagus_Endothelia_Lymphatic	Adult	Digestive system	Madissoon_2020_oesophagus	Endothelia_Lymphatic	Endothelial cell	CL:0000115	Esophagus	UBERON:0001043	0.81962787	0.791016276	0.768634686	0.910530149	0.980334498	0.04070567	NA
FetalLiver_Macrophage	Fetal	Digestive system	FetalLiver	Macrophage	Macrophage	CL:0000235	Liver	UBERON:0002107	0.844282648	0.76894868	0.791881919	0.910984251	0.980334498	0.040489131	NA
FetalAdrenalGland_Erythroid cell	Fetal	Endocrine system	FetalAdrenalGland	Erythroid cell	Erythroid lineage cell	CL:0000764	Adrenal gland	UBERON:0002369	0.833700868	0.777676908	0.78302583	0.91099643	0.980334498	0.040483325	NA
van_Zyl_eye_2020_Macrophage	Adult	Sensory system	van_Zyl_eye_2020	Macrophage	Macrophage	CL:0000235	Eye	UBERON:0000970	0.824066315	0.786858017	0.774907749	0.911393359	0.980334498	0.040294141	NA
van_Zyl_eye_2020_CribiformJCT	Adult	Sensory system	van_Zyl_eye_2020	CribiformJCT	Trabecular meshwork cell	CL:0002367	Eye	UBERON:0000970	0.824066315	0.786899187	0.774907749	0.911409141	0.980334498	0.04028662	NA
Fetal_HCA_Spleen_STC2_TLX1 positive cells	Fetal	Lymphatic system	Fetal_HCA_Spleen	STC2_TLX1 positive cells	Mesenchymal stem cell	CL:0000134	Spleen	UBERON:0002106	0.850963672	0.768372288	0.794095941	0.911600472	0.980334498	0.040195458	NA
Madissoon_2020_lung_Secretory_club	Adult	Respiratory system	Madissoon_2020_lung	Secretory_club	Club cell	CL:0000158	Lung	UBERON:0002048	0.823560245	0.790975105	0.77195572	0.911815932	0.980334498	0.040092824	NA
FetalPancreas_Fetal stromal cell	Fetal	Digestive system	FetalPancreas	Fetal stromal cell	Stromal cell	CL:0000499	Pancreas	UBERON:0001264	0.833644026	0.782411561	0.780442804	0.911830591	0.980334498	0.040085842	NA
Madissoon_2020_lung_DC_plasmacytoid	Adult	Respiratory system	Madissoon_2020_lung	DC_plasmacytoid	Plasmacytoid dendritic cell	CL:0000784	Lung	UBERON:0002048	0.823560245	0.791098618	0.77195572	0.911862961	0.980334498	0.040070425	NA
FetalPancreas_Stromal cell	Fetal	Digestive system	FetalPancreas	Stromal cell	Stromal cell	CL:0000499	Pancreas	UBERON:0001264	0.833644026	0.782823269	0.780442804	0.911989001	0.980334498	0.040010399	NA
AdultAdipose_Stromal cell	Adult	Integumentary system	AdultAdipose	Stromal cell	Stromal cell	CL:0000499	Adipose tissue	UBERON:0001013	0.857487953	0.763884661	0.8	0.912067297	0.980334498	0.039973116	NA
van_Zyl_eye_2020_CollectorChnlAqVein	Adult	Sensory system	van_Zyl_eye_2020	CollectorChnlAqVein	Smooth muscle cell	CL:0000192	Eye	UBERON:0000970	0.824066315	0.789081244	0.774907749	0.912243227	0.980334498	0.039889353	NA
AdultProstate_Gastric endocrine cell	Adult	Reproductive system	AdultProstate	Gastric endocrine cell	Endocrine cell	CL:0000163	Prostate gland	UBERON:0002367	0.867669195	0.7587383	0.805904059	0.912245738	0.980334498	0.039888157	NA
TS_Bladder_mast cell	Adult	Urinary system	TS_Bladder	mast cell	Mast cell	CL:0000097	Bladder organ	UBERON:0018707	0.841639844	0.777224029	0.787453875	0.912519858	0.980334498	0.039757676	NA
FetalMaleGonad_Immature sertoli cell (Pre-Sertoli cell)	Fetal	Reproductive system	FetalMaleGonad	Immature sertoli cell (Pre-Sertoli cell)	Sertoli cell	CL:0000216	Immature gonad	UBERON:2005272	0.827015421	0.789040074	0.776383764	0.912799366	0.980334498	0.03962467	NA
FetalPancreas_CB CD34+	Fetal	Digestive system	FetalPancreas	CB CD34+	Hematopoietic stem cell	CL:0000037	Pancreas	UBERON:0001264	0.833644026	0.785005327	0.780442804	0.912825755	0.980334498	0.039612115	NA
FetalBrain_Fetal endocrine cell	Fetal	Nervous system	FetalBrain	Fetal endocrine cell	Endocrine cell	CL:0000163	Brain	UBERON:0000955	0.836980815	0.781505801	0.784870849	0.913182679	0.980334498	0.039442335	NA
FetalAdrenalGland_Fetal enterocyte	Fetal	Endocrine system	FetalAdrenalGland	Fetal enterocyte	Enterocyte	CL:0000584	Adrenal gland	UBERON:0002369	0.833700868	0.785046497	0.78302583	0.913831575	0.980334498	0.03913384	NA
CordBlood_T cell	Neonatal	Reproductive system	CordBlood	T cell	T cell	CL:0000084	Umbilical cord blood	UBERON:0012168	0.881591757	0.748322065	0.821771218	0.913947267	0.980334498	0.039078862	NA
AdultBladder_T cell	Adult	Urinary system	AdultBladder	T cell	T cell	CL:0000084	Bladder organ	UBERON:0018707	0.849570492	0.775741877	0.79298893	0.914046997	0.980334498	0.039031474	NA
CordBlood_B cell	Neonatal	Reproductive system	CordBlood	B cell	B cell	CL:0000236	Umbilical cord blood	UBERON:0012168	0.881591757	0.750545293	0.821771218	0.914832863	0.980334498	0.038658243	NA
FetalBrain_Fetal mesenchymal progenitor	Fetal	Nervous system	FetalBrain	Fetal mesenchymal progenitor	Mesenchymal stem cell	CL:0000134	Brain	UBERON:0000955	0.836980815	0.786158111	0.784870849	0.914956553	0.980334498	0.038599528	NA
FetalAdrenalGland_Macrophage	Fetal	Endocrine system	FetalAdrenalGland	Macrophage	Macrophage	CL:0000235	Adrenal gland	UBERON:0002369	0.833700868	0.78982232	0.78302583	0.915639916	0.980334498	0.038275283	NA
TS_Muscle_cd8-positive, alpha-beta t cell	Adult	Muscular system	TS_Muscle	cd8-positive, alpha-beta t cell	T cell	CL:0000084	Muscle organ	UBERON:0001630	0.904004136	0.740787793	0.836162362	0.916104123	0.980334498	0.038055162	NA
AdultFallopiantube_T cell	Adult	Reproductive system	AdultFallopiantube	T cell	T cell	CL:0000084	Fallopian tube	UBERON:0003889	0.856270476	0.776277098	0.798154982	0.916185795	0.980334498	0.038016446	NA
AdultGallbladder_Endothelial cell	Adult	Digestive system	AdultGallbladder	Endothelial cell	Endothelial cell	CL:0000115	Gall bladder	UBERON:0002110	0.856082788	0.780435358	0.795940959	0.916945379	0.980334498	0.037656534	NA
AdultBladder_Monocyte	Adult	Urinary system	AdultBladder	Monocyte	Monocyte	CL:0000576	Bladder organ	UBERON:0018707	0.849570492	0.783523175	0.79298893	0.917014276	0.980334498	0.037623903	NA
AdultFallopiantube_Dendritic cell	Adult	Reproductive system	AdultFallopiantube	Dendritic cell	Dendritic cell	CL:0000451	Fallopian tube	UBERON:0003889	0.856270476	0.779900136	0.798154982	0.917563331	0.980334498	0.037363951	NA
AdultDuodenum_Enterocyte	Adult	Digestive system	AdultDuodenum	Enterocyte	Enterocyte	CL:0000584	Duodenum	UBERON:0002114	0.870033667	0.771007225	0.807380074	0.917570295	0.980334498	0.037360655	NA
AdultFallopiantube_Fibroblast	Adult	Reproductive system	AdultFallopiantube	Fibroblast	Fibroblast	CL:0000057	Fallopian tube	UBERON:0003889	0.856270476	0.779982478	0.798154982	0.917594482	0.980334498	0.037349207	NA
AdultGallbladder_Smooth muscle cell	Adult	Digestive system	AdultGallbladder	Smooth muscle cell	Smooth muscle cell	CL:0000192	Gall bladder	UBERON:0002110	0.856082788	0.78286444	0.795940959	0.917862933	0.980334498	0.037222168	NA
Fetal_HCA_Muscle_Lymphoid cells	Fetal	Muscular system	Fetal_HCA_Muscle	Lymphoid cells	Innate lymphoid cell	CL:0001065	Muscle organ	UBERON:0001630	0.862383136	0.776977003	0.802214022	0.917951821	0.980334498	0.037180112	NA
FetalThymus_Proliferating T cell	Fetal	Lymphatic system	FetalThymus	Proliferating T cell	T cell	CL:0000084	Thymus	UBERON:0002370	0.913596713	0.740746622	0.842804428	0.918425009	0.980334498	0.036956299	NA
AdultFallopiantube_Endothelial cell (APC)	Adult	Reproductive system	AdultFallopiantube	Endothelial cell (APC)	Endothelial cell	CL:0000115	Fallopian tube	UBERON:0003889	0.856270476	0.782329219	0.798154982	0.91847938	0.980334498	0.036930589	NA
AdultThyroid_B cell (Plasmocyte)	Adult	Endocrine system	AdultThyroid	B cell (Plasmocyte)	B cell	CL:0000236	Thyroid gland	UBERON:0002046	0.873645035	0.770842541	0.810332103	0.918582237	0.980334498	0.036881957	NA
FetalHeart_Fetal mesenchymal progenitor	Fetal	Cardiovascular system	FetalHeart	Fetal mesenchymal progenitor	Mesenchymal stem cell	CL:0000134	Heart	UBERON:0000948	0.862827411	0.777553396	0.803690037	0.918710863	0.980334498	0.036821148	NA
FetalHeart_Macrophage	Fetal	Cardiovascular system	FetalHeart	Macrophage	Macrophage	CL:0000235	Heart	UBERON:0000948	0.862827411	0.779241402	0.803690037	0.919347205	0.980334498	0.03652044	NA
FetalHeart_Stromal cell	Fetal	Cardiovascular system	FetalHeart	Stromal cell	Stromal cell	CL:0000499	Heart	UBERON:0000948	0.862827411	0.779941307	0.803690037	0.919610193	0.980334498	0.036396224	NA
TS_Eye_erythroid lineage cell	Adult	Sensory system	TS_Eye	erythroid lineage cell	Erythroid lineage cell	CL:0000764	Eye	UBERON:0000970	0.901716542	0.751121686	0.835424354	0.919924777	0.980334498	0.036247684	NA
TS_Vasculature_nk cell	Adult	Cardiovascular system	TS_Vasculature	nk cell	Natural killer cell	CL:0000623	Vasculature	UBERON:0002049	0.841837443	0.795298049	0.789667897	0.920165017	0.980334498	0.036134282	NA
FetalSkin_Epithelial cell	Fetal	Integumentary system	FetalSkin	Epithelial cell	Epithelial cell	CL:0000066	Skin of body	UBERON:0002097	0.865359222	0.781217605	0.805166052	0.920624097	0.980334498	0.035917662	NA
AdultEsophagus_Monocyte	Adult	Digestive system	AdultEsophagus	Monocyte	Monocyte	CL:0000576	Esophagus	UBERON:0001043	0.868248078	0.783893713	0.806642066	0.922150749	0.980334498	0.035198077	NA
AdultHeart_Monocyte	Adult	Cardiovascular system	AdultHeart	Monocyte	Monocyte	CL:0000576	Heart	UBERON:0000948	0.887347962	0.768289946	0.823616236	0.922358877	0.980334498	0.035100068	NA
AdultIleum_T cell	Adult	Digestive system	AdultIleum	T cell	T cell	CL:0000084	Ileum	UBERON:0002116	0.873191643	0.782823269	0.808487085	0.922416723	0.980334498	0.035072832	NA
AdultIleum_Epithelial cell	Adult	Digestive system	AdultIleum	Epithelial cell	Epithelial cell	CL:0000066	Ileum	UBERON:0002116	0.873191643	0.783729029	0.808487085	0.92275128	0.980334498	0.034915344	NA
AdultLiver_Neutrophil	Adult	Digestive system	AdultLiver	Neutrophil	Neutrophil	CL:0000775	Liver	UBERON:0002107	0.875690995	0.779900136	0.812546125	0.922783232	0.980334498	0.034900306	NA
TS_Liver_plasma cell	Adult	Digestive system	TS_Liver	plasma cell	Plasma cell	CL:0000786	Liver	UBERON:0002107	0.842118227	0.802255929	0.790405904	0.922966936	0.980334498	0.034813857	NA
TS_Uterus_epithelial cell	Adult	Reproductive system	TS_Uterus	epithelial cell	Epithelial cell	CL:0000066	Uterus	UBERON:0000995	0.841699384	0.804561498	0.788191882	0.92298562	0.980334498	0.034805065	NA
TS_Mammary_plasma cell	Adult	Endocrine system	TS_Mammary	plasma cell	Plasma cell	CL:0000786	Mammary gland	UBERON:0001911	0.841792791	0.804067448	0.788929889	0.923078504	0.980334498	0.034761362	NA
AdultOmentum_Monocyte	Adult	Integumentary system	AdultOmentum	Monocyte	Monocyte	CL:0000576	Omentum	UBERON:0003688	0.873235962	0.786281624	0.809594096	0.924083304	0.980334498	0.034288877	NA
FetalStomach_Fetal mesenchymal progenitor	Fetal	Digestive system	FetalStomach	Fetal mesenchymal progenitor	Mesenchymal stem cell	CL:0000134	Stomach	UBERON:0000945	0.884604715	0.782205706	0.822878229	0.927246731	0.980334498	0.032804689	NA
FetalFemaleGonad_Primordial germ cell	Fetal	Reproductive system	FetalFemaleGonad	Primordial germ cell	Primordial germ cell	CL:0000670	Immature gonad	UBERON:2005272	0.879186998	0.785911086	0.819557196	0.927440016	0.980334498	0.03271417	NA
AdultAdrenalGland_Fasciculata cell	Adult	Endocrine system	AdultAdrenalGland	Fasciculata cell	Type II cell of adrenal cortex	CL:0002136	Adrenal gland	UBERON:0002369	0.876249345	0.790069346	0.815498155	0.927528408	0.980334498	0.03267278	NA
Braga_2020_lung_NK cell	Adult	Respiratory system	Braga_2020_lung	NK cell	Natural killer cell	CL:0000623	Lung	UBERON:0002048	0.897156008	0.775494851	0.83099631	0.927586977	0.980334498	0.032645357	NA
VentoTormo_2018_decidua_conventional dendritic cell (DC1)	Maternal-Fetal	Reproductive system	VentoTormo_2018_decidua	conventional dendritic cell (DC1)	Dendritic cell	CL:0000451	Decidua	UBERON:0002450	0.86002139	0.804973207	0.801107011	0.927778542	0.980334498	0.032555676	NA
FetalIntestine_Endothelial cell	Fetal	Digestive system	FetalIntestine	Endothelial cell	Endothelial cell	CL:0000115	Intestine	UBERON:0000160	0.876192889	0.792004378	0.814760148	0.92796438	0.980334498	0.032468694	NA
VentoTormo_2018_decidua_decidual stromal cell (dS3)	Maternal-Fetal	Reproductive system	VentoTormo_2018_decidua	decidual stromal cell (dS3)	Stromal cell	CL:0000499	Decidua	UBERON:0002450	0.86002139	0.805508429	0.801107011	0.927966503	0.980334498	0.0324677	NA
AdultKidney_Fetal epithelial progenitor	Adult	Urinary system	AdultKidney	Fetal epithelial progenitor	Epithelial fate stem cell	CL:0000036	Kidney	UBERON:0002113	0.876433418	0.791551498	0.816236162	0.928314042	0.980334498	0.03230508	NA
Madissoon_2020_spleen_Plasma_IgM	Adult	Lymphatic system	Madissoon_2020_spleen	Plasma_IgM	Plasma cell	CL:0000786	Spleen	UBERON:0002106	0.878583687	0.790933934	0.817712177	0.928603462	0.980334498	0.032169702	NA
AdultBoneMarrow_Antigen presenting cell (RPS high)	Adult	Lymphatic system	AdultBoneMarrow	Antigen presenting cell (RPS high)	Professional antigen presenting cell	CL:0000145	Bone marrow	UBERON:0002371	0.891263169	0.783276149	0.825830258	0.928645654	0.980334498	0.032149969	NA
FetalFemaleGonad_Endothelial cell	Fetal	Reproductive system	FetalFemaleGonad	Endothelial cell	Endothelial cell	CL:0000115	Immature gonad	UBERON:2005272	0.879186998	0.789451782	0.819557196	0.92871	0.980334498	0.032119878	NA
AdultEpityphlon_Dendritic cell	Adult	Digestive system	AdultEpityphlon	Dendritic cell	Dendritic cell	CL:0000451	Caecum	UBERON:0001153	0.909099755	0.773312794	0.839114391	0.929533192	0.980334498	0.031735098	NA
AdultProstate_Dendritic cell	Adult	Reproductive system	AdultProstate	Dendritic cell	Dendritic cell	CL:0000451	Prostate gland	UBERON:0002367	0.926748182	0.768042921	0.847232472	0.930314751	0.980334498	0.031370093	NA
Madissoon_2020_spleen_NK_dividing	Adult	Lymphatic system	Madissoon_2020_spleen	NK_dividing	Natural killer cell	CL:0000623	Spleen	UBERON:0002106	0.878583687	0.795915612	0.817712177	0.930365867	0.980334498	0.031346231	NA
Madissoon_2020_spleen_DC_1	Adult	Lymphatic system	Madissoon_2020_spleen	DC_1	Dendritic cell	CL:0000451	Spleen	UBERON:0002106	0.878583687	0.796368492	0.817712177	0.930524819	0.980334498	0.031272039	NA
Lake_2017_FrontalCortex_In1c	Adult	Nervous system	Lake_2017_FrontalCortex	In1c	Inhibitory neuron	CL:0008029	Frontal cortex	UBERON:0001870	0.875880846	0.800650264	0.813653137	0.9306326	0.980334498	0.031221738	NA
Fetal_HCA_Heart_Endocardial cells	Fetal	Cardiovascular system	Fetal_HCA_Heart	Endocardial cells	Endocardial cell	CL:0002350	Heart	UBERON:0000948	0.88154834	0.793898238	0.820664207	0.930663567	0.980334498	0.031207287	NA
FetalLiver_T cell	Fetal	Digestive system	FetalLiver	T cell	T cell	CL:0000084	Liver	UBERON:0002107	0.911554107	0.774259725	0.841697417	0.930735756	0.980334498	0.031173602	NA
Lake_2017_FrontalCortex_Per	Adult	Nervous system	Lake_2017_FrontalCortex	Per	Pericyte cell	CL:0000669	Frontal cortex	UBERON:0001870	0.875880846	0.801720707	0.813653137	0.931005274	0.980334498	0.031047859	NA
Lake_2017_VisualCortex_Ex3c	Adult	Nervous system	Lake_2017_VisualCortex	Ex3c	Excitatory neuron	CL:0008030	Visual cortex	UBERON:0000411	0.875525336	0.803943935	0.811439114	0.931017656	0.980334498	0.031042083	NA
Lake_2017_VisualCortex_Per	Adult	Nervous system	Lake_2017_VisualCortex	Per	Pericyte cell	CL:0000669	Visual cortex	UBERON:0000411	0.875525336	0.804396815	0.811439114	0.931174532	0.980334498	0.030968911	NA
FetalEyes_Fetal neuron	Fetal	Sensory system	FetalEyes	Fetal neuron	Neuron	CL:0000540	Eye	UBERON:0000970	0.89467402	0.786487479	0.830258303	0.931289115	0.980334498	0.030915473	NA
FetalLiver_Dendritic cell	Fetal	Digestive system	FetalLiver	Dendritic cell	Dendritic cell	CL:0000451	Liver	UBERON:0002107	0.911554107	0.775947731	0.841697417	0.931342566	0.980334498	0.030890548	NA
Madissoon_2020_oesophagus_T_CD8	Adult	Digestive system	Madissoon_2020_oesophagus	T_CD8	T cell	CL:0000084	Esophagus	UBERON:0001043	0.888880639	0.792951308	0.824354244	0.931580708	0.980334498	0.030779514	NA
AdultLung_Epithelial cell	Adult	Respiratory system	AdultLung	Epithelial cell	Epithelial cell	CL:0000066	Lung	UBERON:0002048	0.889112466	0.79258077	0.825092251	0.931699467	0.980334498	0.030724153	NA
NeonatalAdrenalGland_Dendritic cell	Neonatal	Endocrine system	NeonatalAdrenalGland	Dendritic cell	Dendritic cell	CL:0000451	Adrenal gland	UBERON:0002369	0.904154743	0.78097058	0.837638376	0.931792397	0.980334498	0.03068088	NA
AdultUterus_Endothelial cell (APC)	Adult	Reproductive system	AdultUterus	Endothelial cell (APC)	Endothelial cell	CL:0000115	Uterus	UBERON:0000995	0.906040505	0.780435358	0.838376384	0.931846465	0.980334498	0.030655638	NA
Fetal_HCA_Stomach_Lymphoid cells	Fetal	Digestive system	Fetal_HCA_Stomach	Lymphoid cells	Innate lymphoid cell	CL:0001065	Stomach	UBERON:0000945	0.91070695	0.778788522	0.840590406	0.931992121	0.980334498	0.030587759	NA
AdultCerebellum_Smooth muscle cell	Adult	Nervous system	AdultCerebellum	Smooth muscle cell	Smooth muscle cell	CL:0000192	Cerebellum	UBERON:0002037	0.901448576	0.787393238	0.834317343	0.932959861	0.980334498	0.030137041	NA
AdultCerebellum_Endothelial cell (APC)	Adult	Nervous system	AdultCerebellum	Endothelial cell (APC)	Endothelial cell	CL:0000115	Cerebellum	UBERON:0002037	0.901448576	0.789081244	0.834317343	0.93355009	0.980334498	0.029862375	NA
FetalThymus_Dendritic cell	Fetal	Lymphatic system	FetalThymus	Dendritic cell	Dendritic cell	CL:0000451	Thymus	UBERON:0002370	0.959081889	0.747210451	0.881180812	0.933584925	0.980334498	0.02984617	NA
Madissoon_2020_lung_B_cell_naive	Adult	Respiratory system	Madissoon_2020_lung	B_cell_naive	B cell	CL:0000236	Lung	UBERON:0002048	0.891654994	0.79813884	0.827306273	0.934366966	0.980334498	0.029482524	NA
CordBlood_Monocyte	Neonatal	Reproductive system	CordBlood	Monocyte	Monocyte	CL:0000576	Umbilical cord blood	UBERON:0012168	0.938074532	0.763555294	0.864944649	0.934419997	0.980334498	0.029457876	NA
FetalPancreas_Macrophage	Fetal	Digestive system	FetalPancreas	Macrophage	Macrophage	CL:0000235	Pancreas	UBERON:0001264	0.898774329	0.795298049	0.831734317	0.934852125	0.980334498	0.02925708	NA
FetalAdrenalGland_T cell	Fetal	Endocrine system	FetalAdrenalGland	T cell	T cell	CL:0000084	Adrenal gland	UBERON:0002369	0.898806546	0.794392289	0.832841328	0.934904207	0.980334498	0.029232886	NA
van_Zyl_eye_2020_CornealEpi	Adult	Sensory system	van_Zyl_eye_2020	CornealEpi	Epithelial cell	CL:0000066	Eye	UBERON:0000970	0.892045011	0.798344694	0.829151292	0.935046966	0.980334498	0.029166575	NA
FetalAdrenalGland_Neutrophil	Fetal	Endocrine system	FetalAdrenalGland	Neutrophil	Neutrophil	CL:0000775	Adrenal gland	UBERON:0002369	0.898806546	0.795133365	0.832841328	0.935158878	0.980334498	0.029114599	NA
van_Zyl_eye_2020_ScEndo	Adult	Sensory system	van_Zyl_eye_2020	ScEndo	Endothelial cell	CL:0000115	Eye	UBERON:0000970	0.892045011	0.798879916	0.829151292	0.935229906	0.980334498	0.029081614	NA
AdultJeJunum_Mast cell	Adult	Digestive system	AdultJeJunum	Mast cell	Mast cell	CL:0000097	Jejunum	UBERON:0002115	0.910152097	0.789987004	0.839852399	0.935673943	0.980334498	0.028875465	NA
Madissoon_2020_lung_T_cells_Dividing	Adult	Respiratory system	Madissoon_2020_lung	T_cells_Dividing	T cell	CL:0000084	Lung	UBERON:0002048	0.891654994	0.802379441	0.827306273	0.935813784	0.980334498	0.028810562	NA
AdultRectum_Dendritic cell	Adult	Digestive system	AdultRectum	Dendritic cell	Dendritic cell	CL:0000451	Rectum	UBERON:0001052	0.930203474	0.779858966	0.851660517	0.935961829	0.980334498	0.028741862	NA
Fetal_HCA_Thymus_Antigen presenting cells	Fetal	Lymphatic system	Fetal_HCA_Thymus	Antigen presenting cells	Professional antigen presenting cell	CL:0000145	Thymus	UBERON:0002370	0.962008365	0.752109787	0.883394834	0.936057875	0.980334498	0.028697299	NA
Madissoon_2020_lung_DC_1	Adult	Respiratory system	Madissoon_2020_lung	DC_1	Dendritic cell	CL:0000451	Lung	UBERON:0002048	0.891654994	0.803326372	0.827306273	0.936134323	0.980334498	0.028661831	NA
AdultGallbladder_Goblet cell	Adult	Digestive system	AdultGallbladder	Goblet cell	Goblet cell	CL:0000160	Gall bladder	UBERON:0002110	0.916583466	0.789081244	0.843542435	0.93655296	0.980334498	0.028467659	NA
FetalSkin_Fetal skeletal muscle cell	Fetal	Integumentary system	FetalSkin	Fetal skeletal muscle cell	Cell of skeletal muscle	CL:0000188	Skin of body	UBERON:0002097	0.922717295	0.78792846	0.845756458	0.936866769	0.980334498	0.028322166	NA
Placenta_Fetal stromal cell	Adult	Reproductive system	Placenta	Fetal stromal cell	Stromal cell	CL:0000499	Placenta	UBERON:0001987	0.922439869	0.790645738	0.844649446	0.937442767	0.980334498	0.028055238	NA
Placenta_Macrophage	Adult	Reproductive system	Placenta	Macrophage	Macrophage	CL:0000235	Placenta	UBERON:0001987	0.922439869	0.792374916	0.844649446	0.938030851	0.980334498	0.027782878	NA
AdultThyroid_Macrophage	Adult	Endocrine system	AdultThyroid	Macrophage	Macrophage	CL:0000235	Thyroid gland	UBERON:0002046	0.928115375	0.790810422	0.84797048	0.938553551	0.980334498	0.027540943	NA
FetalLung_Fetal chondrocyte	Fetal	Respiratory system	FetalLung	Fetal chondrocyte	Chondrocyte	CL:0000138	Lung	UBERON:0002048	0.929934041	0.788916561	0.850922509	0.938842064	0.980334498	0.027407461	NA
MacParland_2018_liver_Hepatocyte	Adult	Digestive system	MacParland_2018_liver	Hepatocyte	Hepatocyte	CL:0000182	Liver	UBERON:0002107	0.926017214	0.79583327	0.846494465	0.93977908	0.980334498	0.026974227	NA
FetalIntestine_Erythroid cell	Fetal	Digestive system	FetalIntestine	Erythroid cell	Erythroid lineage cell	CL:0000764	Intestine	UBERON:0000160	0.92976615	0.794104093	0.849446494	0.94012631	0.980334498	0.026813793	NA
TS_Eye_b cell	Adult	Sensory system	TS_Eye	b cell	B cell	CL:0000236	Eye	UBERON:0000970	0.904119549	0.806784726	0.836900369	0.940381711	0.980334498	0.026695826	NA
AdultKidney_B cell	Adult	Urinary system	AdultKidney	B cell	B cell	CL:0000236	Kidney	UBERON:0002113	0.929904027	0.794680485	0.850184502	0.940549122	0.980334498	0.026618518	NA
AdultSpleen_B cell (Plasmocyte)	Adult	Lymphatic system	AdultSpleen	B cell (Plasmocyte)	B cell	CL:0000236	Spleen	UBERON:0002106	0.938712838	0.780888238	0.865682657	0.940696599	0.980334498	0.026550426	NA
FetalStomach_T cell	Fetal	Digestive system	FetalStomach	T cell	T cell	CL:0000084	Stomach	UBERON:0000945	0.935193067	0.79072808	0.857195572	0.941401815	0.980334498	0.026224969	NA
FetalStomach_Macrophage	Fetal	Digestive system	FetalStomach	Macrophage	Macrophage	CL:0000235	Stomach	UBERON:0000945	0.935193067	0.791345643	0.857195572	0.941607476	0.980334498	0.026130102	NA
FetalCalvaria_Fetal chondrocyte	Fetal	Skeletal system	FetalCalvaria	Fetal chondrocyte	Chondrocyte	CL:0000138	Vault of skull	UBERON:0004339	0.941686138	0.777718079	0.872324723	0.941640354	0.980334498	0.026114938	NA
Fetal_HCA_Heart_Myeloid cells	Fetal	Cardiovascular system	Fetal_HCA_Heart	Myeloid cells	Myeloid cell	CL:0000763	Heart	UBERON:0000948	0.933227445	0.795668587	0.854612546	0.942245996	0.980334498	0.025835699	NA
Madissoon_2020_spleen_Plasma_IgG	Adult	Lymphatic system	Madissoon_2020_spleen	Plasma_IgG	Plasma cell	CL:0000786	Spleen	UBERON:0002106	0.931299443	0.797562447	0.853136531	0.942416055	0.980334498	0.025757324	NA
Lake_2017_FrontalCortex_In4a	Adult	Nervous system	Lake_2017_FrontalCortex	In4a	Inhibitory neuron	CL:0008029	Frontal cortex	UBERON:0001870	0.929492272	0.802997005	0.848708487	0.942831305	0.980334498	0.025566006	NA
AdultBoneMarrow_B cell	Adult	Lymphatic system	AdultBoneMarrow	B cell	B cell	CL:0000236	Bone marrow	UBERON:0002371	0.941457307	0.784305421	0.869372694	0.942962872	0.980334498	0.025505407	NA
Madissoon_2020_spleen_T_cell_dividing	Adult	Lymphatic system	Madissoon_2020_spleen	T_cell_dividing	T cell	CL:0000084	Spleen	UBERON:0002106	0.931299443	0.799415137	0.853136531	0.943022799	0.980334498	0.025477807	NA
Madissoon_2020_spleen_B_Hypermutation	Adult	Lymphatic system	Madissoon_2020_spleen	B_Hypermutation	B cell	CL:0000236	Spleen	UBERON:0002106	0.931299443	0.799620992	0.853136531	0.943089989	0.980334498	0.025446865	NA
AdultBoneMarrow_Neutrophil (RPS high)	Adult	Lymphatic system	AdultBoneMarrow	Neutrophil (RPS high)	Neutrophil	CL:0000775	Bone marrow	UBERON:0002371	0.941457307	0.78517001	0.869372694	0.943250294	0.980334498	0.025373051	NA
AdultBoneMarrow_Dendritic cell	Adult	Lymphatic system	AdultBoneMarrow	Dendritic cell	Dendritic cell	CL:0000451	Bone marrow	UBERON:0002371	0.941457307	0.785499377	0.869372694	0.943359571	0.980334498	0.02532274	NA
FetalLiver_Neutrophil (RPS high)	Fetal	Digestive system	FetalLiver	Neutrophil (RPS high)	Neutrophil	CL:0000775	Liver	UBERON:0002107	0.955664994	0.777141687	0.879335793	0.943541262	0.980334498	0.025239103	NA
FetalMuscle_Fetal mesenchymal progenitor	Fetal	Muscular system	FetalMuscle	Fetal mesenchymal progenitor	Mesenchymal stem cell	CL:0000134	Muscle organ	UBERON:0001630	0.936491966	0.795709758	0.85904059	0.943609189	0.980334498	0.025207838	NA
FetalLiver_B cell	Fetal	Digestive system	FetalLiver	B cell	B cell	CL:0000236	Liver	UBERON:0002107	0.955664994	0.777553396	0.879335793	0.943678999	0.980334498	0.02517571	NA
FetalKidney_Intermediated cell	Fetal	Urinary system	FetalKidney	Intermediated cell	Kidney cell	CL:1000497	Kidney	UBERON:0002113	0.936584551	0.794392289	0.860885609	0.943736147	0.980334498	0.02514941	NA
FetalKidney_Neutrophil	Fetal	Urinary system	FetalKidney	Neutrophil	Neutrophil	CL:0000775	Kidney	UBERON:0002113	0.936584551	0.794927511	0.860885609	0.943911069	0.980334498	0.025068921	NA
FetalMuscle_Dendritic cell	Fetal	Muscular system	FetalMuscle	Dendritic cell	Dendritic cell	CL:0000451	Muscle organ	UBERON:0001630	0.936491966	0.796986055	0.85904059	0.944025443	0.980334498	0.0250163	NA

FetalSpinalCord_Dendritic cell	Fetal	Nervous system	FetalSpinalCord	Dendritic cell	Dendritic cell	CL:0000451	Spinal cord	UBERON:0002240	0.941555738	0.786281624	0.870848708	0.944059143	0.980334498	0.025000797	NA
FetalMuscle_Fetal skeletal muscle cell	Fetal	Muscular system	FetalMuscle	Fetal skeletal muscle cell	Cell of skeletal muscle	CL:0000188	Muscle organ	UBERON:0001630	0.936491966	0.79723308	0.85904059	0.944105806	0.980334498	0.024979332	NA
Voigt_2019_retina_2_White blood cell 1	Adult	Sensory system	Voigt_2019_retina_2	White blood cell 1	Leukocyte	CL:0000738	Retina	UBERON:0000966	0.93460991	0.803120517	0.855719557	0.945006502	0.980334498	0.024565203	NA
AdultUterus_Loop of Henle	Adult	Reproductive system	AdultUterus	Loop of Henle	Kidney loop of Henle epithelial cell	CL:1000909	Uterus	UBERON:0000995	0.950458102	0.782699757	0.878228782	0.945060317	0.980334498	0.024540473	NA
AdultLung_Monocyte	Adult	Respiratory system	AdultLung	Monocyte	Monocyte	CL:0000576	Lung	UBERON:0002048	0.938032826	0.796203808	0.863468635	0.945100628	0.980334498	0.024521948	NA
Voigt_2019_retina_2_Macrophage 3	Adult	Sensory system	Voigt_2019_retina_2	Macrophage 3	Macrophage	CL:0000235	Retina	UBERON:0000966	0.93460991	0.803532226	0.855719557	0.94513855	0.980334498	0.024504523	NA
AdultMuscle_Macrophage	Adult	Muscular system	AdultMuscle	Macrophage	Macrophage	CL:0000235	Muscle organ	UBERON:0001630	0.946353592	0.785828744	0.875276753	0.945220688	0.980334498	0.024466782	NA
FetalEyes_Fetal mesenchymal progenitor	Fetal	Sensory system	FetalEyes	Fetal mesenchymal progenitor	Mesenchymal stem cell	CL:0000134	Eye	UBERON:0000970	0.941589745	0.789616466	0.871586716	0.945370858	0.980334498	0.024397789	NA
AdultLung_B cell	Adult	Respiratory system	AdultLung	B cell	B cell	CL:0000236	Lung	UBERON:0002048	0.938032826	0.797727131	0.863468635	0.945592226	0.980334498	0.024296107	NA
AdultLung_Mast cell	Adult	Respiratory system	AdultLung	Mast cell	Mast cell	CL:0000097	Lung	UBERON:0002048	0.938032826	0.797768302	0.863468635	0.945605478	0.980334498	0.024290021	NA
CordBlood_Neutrophil	Neonatal	Reproductive system	CordBlood	Neutrophil	Neutrophil	CL:0000775	Umbilical cord blood	UBERON:0012168	0.972025892	0.764625737	0.902583026	0.946085211	0.980334498	0.024069746	NA
FetalMaleGonad_Primordial germ cell	Fetal	Reproductive system	FetalMaleGonad	Primordial germ cell	Primordial germ cell	CL:0000670	Immature gonad	UBERON:2005272	0.941218627	0.797027226	0.867896679	0.946676397	0.980334498	0.023798451	NA
AdultCerebellum_Monocyte	Adult	Nervous system	AdultCerebellum	Monocyte	Monocyte	CL:0000576	Cerebellum	UBERON:0002037	0.945821335	0.791757352	0.874169742	0.946821139	0.980334498	0.023732054	NA
Madissoon_2020_oesophagus_Dendritic_Cells	Adult	Digestive system	Madissoon_2020_oesophagus	Dendritic_Cells	Dendritic cell	CL:0000451	Esophagus	UBERON:0001043	0.937880527	0.80324403	0.86199262	0.946916804	0.980334498	0.023688176	NA
AdultCerebellum_Neutrophil	Adult	Nervous system	AdultCerebellum	Neutrophil	Neutrophil	CL:0000775	Cerebellum	UBERON:0002037	0.945821335	0.79212789	0.874169742	0.946940257	0.980334498	0.02367742	NA
AdultPeripheralBlood_B cell	Adult	Lymphatic system	AdultPeripheralBlood	B cell	B cell	CL:0000236	Blood	UBERON:0000178	0.972000573	0.768866338	0.901476015	0.947182622	0.980334498	0.023566278	NA
AdultPeripheralBlood_Dendritic cell	Adult	Lymphatic system	AdultPeripheralBlood	Dendritic cell	Dendritic cell	CL:0000451	Blood	UBERON:0000178	0.972000573	0.769772098	0.901476015	0.947481445	0.980334498	0.023429286	NA
van_Zyl_eye_2020_VascularEndo	Adult	Sensory system	van_Zyl_eye_2020	VascularEndo	Endothelial cell	CL:0000115	Eye	UBERON:0000970	0.939900859	0.801350169	0.867158672	0.947833975	0.980334498	0.023267728	NA
AdultRectum_B cell (Plasmocyte)	Adult	Digestive system	AdultRectum	B cell (Plasmocyte)	B cell	CL:0000236	Rectum	UBERON:0001052	0.966139552	0.779817795	0.891512915	0.947950337	0.980334498	0.023214415	NA
TS_Eye_t cell	Adult	Sensory system	TS_Eye	t cell	T cell	CL:0000084	Eye	UBERON:0000970	0.947307652	0.794186435	0.87601476	0.948132432	0.980334498	0.023130998	NA
TS_Lymph_Node_innate lymphoid cell	Adult	Lymphatic system	TS_Lymph_Node	innate lymphoid cell	Innate lymphoid cell	CL:0001065	Lymph node	UBERON:0000029	0.947418305	0.793198333	0.877490775	0.948243088	0.980334498	0.023080314	NA
AdultGallbladder_Stromal cell	Adult	Digestive system	AdultGallbladder	Stromal cell	Stromal cell	CL:0000499	Gall bladder	UBERON:0002110	0.956698471	0.79072808	0.880442804	0.948303449	0.980334498	0.02305267	NA
AdultSigmoidColon_Epithelial cell	Adult	Digestive system	AdultSigmoidColon	Epithelial cell	Epithelial cell	CL:0000066	Sigmoid colon	UBERON:0001159	0.967087546	0.780147162	0.89298893	0.94847419	0.980334498	0.022974483	NA
Madissoon_2020_lung_NK_Dividing	Adult	Respiratory system	Madissoon_2020_lung	NK_Dividing	Natural killer cell	CL:0000623	Lung	UBERON:0002048	0.939633495	0.804437986	0.866420664	0.94858939	0.980334498	0.022921737	NA
FetalAdrenalGland_Dendritic cell	Fetal	Endocrine system	FetalAdrenalGland	Dendritic cell	Dendritic cell	CL:0000451	Adrenal gland	UBERON:0002369	0.944150542	0.798427036	0.873062731	0.948623748	0.980334498	0.022906007	NA
FetalSkin_Fetal epithelial progenitor	Fetal	Integumentary system	FetalSkin	Fetal epithelial progenitor	Epithelial fate stem cell	CL:0000036	Skin of body	UBERON:0002097	0.960284843	0.791057447	0.881918819	0.948829767	0.980334498	0.022811699	NA
AdultOmentum_T cell	Adult	Integumentary system	AdultOmentum	T cell	T cell	CL:0000084	Omentum	UBERON:0003688	0.96325002	0.791716181	0.884132841	0.949666139	0.980334498	0.022429047	NA
AdultPancreas_Enterocyte progenitor	Adult	Digestive system	AdultPancreas	Enterocyte progenitor	Enterocyte	CL:0000584	Pancreas	UBERON:0001264	0.967109426	0.783605516	0.893726937	0.949789857	0.980334498	0.022372473	NA
FetalLung_Proliferating T cell	Fetal	Respiratory system	FetalLung	Proliferating T cell	T cell	CL:0000084	Lung	UBERON:0002048	0.964444137	0.78887539	0.887822878	0.949808471	0.980334498	0.022363962	NA
AdultEsophagus_Basal cell	Adult	Digestive system	AdultEsophagus	Basal cell	Basal cell	CL:0000646	Esophagus	UBERON:0001043	0.961382678	0.79398058	0.882656827	0.949961745	0.980334498	0.022293883	NA
FetalIntestine_T cell	Fetal	Digestive system	FetalIntestine	T cell	T cell	CL:0000084	Intestine	UBERON:0000160	0.964340585	0.795256878	0.886346863	0.951393769	0.980334498	0.021639697	NA
AdultAscendingColon_Enterocyte progenitor	Adult	Digestive system	AdultAscendingColon	Enterocyte progenitor	Enterocyte	CL:0000584	Ascending colon	UBERON:0001156	0.979735488	0.772324693	0.912915129	0.951461402	0.980334498	0.021608825	NA
AdultPleura_Neutrophil	Adult	Respiratory system	AdultPleura	Neutrophil	Neutrophil	CL:0000775	Pleura	UBERON:0000977	0.96528769	0.794556973	0.888929889	0.9518914	0.980334498	0.021411803	NA
AdultPleura_Dendritic cell	Adult	Respiratory system	AdultPleura	Dendritic cell	Dendritic cell	CL:0000451	Pleura	UBERON:0000977	0.96528769	0.794721656	0.888929889	0.951944026	0.980334498	0.021388587	NA
FetalFemaleGonad_Erythroid cell	Fetal	Reproductive system	FetalFemaleGonad	Erythroid cell	Erythroid lineage cell	CL:0000764	Immature gonad	UBERON:2005272	0.965462505	0.793321846	0.890774908	0.95202022	0.980334498	0.021353828	NA
AdultStomach_Goblet cell	Adult	Digestive system	AdultStomach	Goblet cell	Goblet cell	CL:0000160	Stomach	UBERON:0000945	0.964394358	0.79673903	0.887084871	0.952056301	0.980334498	0.021337368	NA
TS_Mammary_b cell	Adult	Endocrine system	TS_Mammary	b cell	B cell	CL:0000236	Mammary gland	UBERON:0001911	0.94738716	0.807031752	0.876752768	0.952330778	0.980334498	0.021212179	NA
AdultEpityphlon_B cell (Plasmocyte)	Adult	Digestive system	AdultEpityphlon	B cell (Plasmocyte)	B cell	CL:0000236	Caecum	UBERON:0001153	0.98014762	0.775288997	0.913653137	0.952599445	0.980334498	0.021089676	NA
CordBloodCD34P_Neutrophil	Neonatal	Reproductive system	CordBloodCD34P	Neutrophil	Neutrophil	CL:0000775	Umbilical cord blood	UBERON:0012168	0.979637735	0.7772652	0.912177122	0.952826139	0.980334498	0.020986337	NA
FetalSpinalCord_Erythroid cell	Fetal	Nervous system	FetalSpinalCord	Erythroid cell	Erythroid lineage cell	CL:0000764	Spinal cord	UBERON:0002240	0.971017833	0.788340169	0.899630996	0.952898917	0.980334498	0.020953167	NA
AdultBoneMarrow_T cell	Adult	Lymphatic system	AdultBoneMarrow	T cell	T cell	CL:0000084	Bone marrow	UBERON:0002371	0.972231398	0.785911086	0.903321033	0.953144146	0.980334498	0.020841415	NA
Fetal_HCA_Heart_CLC_IL5RA positive cells	Fetal	Cardiovascular system	Fetal_HCA_Heart	CLC_IL5RA positive cells	Endothelial cell	CL:0000115	Heart	UBERON:0000948	0.966318998	0.796368492	0.892250923	0.953359871	0.980334498	0.020743132	NA
Fetal_HCA_Pancreas_Lymphoid cells	Fetal	Digestive system	Fetal_HCA_Pancreas	Lymphoid cells	Innate lymphoid cell	CL:0001065	Pancreas	UBERON:0001264	0.965301299	0.798427036	0.8900369	0.953383446	0.980334498	0.020732393	NA
Lake_2017_CerebellarHemisphere_Per	Adult	Nervous system	Lake_2017_CerebellarHemisphere	Per	Pericyte cell	CL:0000669	Cerebellar hemisphere	UBERON:0002245	0.964137183	0.802914663	0.885608856	0.953533211	0.980334498	0.020664176	NA
FetalEyes_Fetal epithelial progenitor	Fetal	Sensory system	FetalEyes	Fetal epithelial progenitor	Epithelial fate stem cell	CL:0000036	Eye	UBERON:0000970	0.971030704	0.789863491	0.900369004	0.953567394	0.980334498	0.020648607	NA
FetalLiver_Monocyte	Fetal	Digestive system	FetalLiver	Monocyte	Monocyte	CL:0000576	Liver	UBERON:0002107	0.980825945	0.778500326	0.916236162	0.954287353	0.980334498	0.020320832	NA
FetalMuscle_Fetal epithelial progenitor	Fetal	Muscular system	FetalMuscle	Fetal epithelial progenitor	Epithelial fate stem cell	CL:0000036	Muscle organ	UBERON:0001630	0.968151821	0.797480106	0.894464945	0.954296761	0.980334498	0.02031655	NA
FetalLiver_Antigen presenting cell (RPS high)	Fetal	Digestive system	FetalLiver	Antigen presenting cell (RPS high)	Professional antigen presenting cell	CL:0000145	Liver	UBERON:0002107	0.980825945	0.778541497	0.916236162	0.954300097	0.980334498	0.020315032	NA
Lake_2017_VisualCortex_Mic	Adult	Nervous system	Lake_2017_VisualCortex	Mic	Microglial cell	CL:0000129	Visual cortex	UBERON:0000411	0.963970816	0.806208334	0.884870849	0.954321167	0.980334498	0.020305443	NA
Braga_2020_lung_B cell	Adult	Respiratory system	Braga_2020_lung	B cell	B cell	CL:0000236	Lung	UBERON:0002048	0.97418808	0.786158111	0.908487085	0.954599058	0.980334498	0.020178999	NA
AdultMuscle_T cell	Adult	Muscular system	AdultMuscle	T cell	T cell	CL:0000084	Muscle organ	UBERON:0001630	0.974846321	0.786940358	0.909225092	0.95503255	0.980334498	0.019981826	NA
NeonatalAdrenalGland_T cell	Neonatal	Endocrine system	NeonatalAdrenalGland	T cell	T cell	CL:0000084	Adrenal gland	UBERON:0002369	0.976404318	0.786158111	0.910332103	0.955085776	0.980334498	0.019957623	NA
NeonatalAdrenalGland_Monocyte	Neonatal	Endocrine system	NeonatalAdrenalGland	Monocyte	Monocyte	CL:0000576	Adrenal gland	UBERON:0002369	0.976404318	0.786487479	0.910332103	0.95518602	0.980334498	0.019912042	NA
AdultLung_Dendritic cell	Adult	Respiratory system	AdultLung	Dendritic cell	Dendritic cell	CL:0000451	Lung	UBERON:0002048	0.969018919	0.798756403	0.89704797	0.955372247	0.980334498	0.019827379	NA
AdultLung_Mesothelial cell	Adult	Respiratory system	AdultLung	Mesothelial cell	Mesothelial cell	CL:0000077	Lung	UBERON:0002048	0.969018919	0.79908577	0.89704797	0.955470676	0.980334498	0.019782637	NA
TS_Salivary_Gland_t cell	Adult	Digestive system	TS_Salivary_Gland	t cell	T cell	CL:0000084	Saliva-secreting gland	UBERON:0001044	0.974165824	0.790069346	0.907749077	0.955592586	0.980334498	0.019727228	NA
TS_Prostate_erythroid progenitor	Adult	Reproductive system	TS_Prostate	erythroid progenitor	Erythroid progenitor cell	CL:0000038	Prostate gland	UBERON:0002367	0.973332372	0.792663112	0.905535055	0.955791627	0.980334498	0.019636778	NA
Madissoon_2020_oesophagus_B_CD27pos	Adult	Digestive system	Madissoon_2020_oesophagus	B_CD27pos	B cell	CL:0000236	Esophagus	UBERON:0001043	0.968930508	0.803902764	0.895571956	0.956507412	0.980334498	0.01931166	NA
Madissoon_2020_oesophagus_Mono_macro	Adult	Digestive system	Madissoon_2020_oesophagus	Mono_macro	Macrophage	CL:0000235	Esophagus	UBERON:0001043	0.968930508	0.804437986	0.895571956	0.956664745	0.980334498	0.01924023	NA
AdultBladder_Sinusoidal endothelial cell	Adult	Urinary system	AdultBladder	Sinusoidal endothelial cell	Endothelial cell	CL:0000115	Bladder organ	UBERON:0018707	0.978895448	0.790933934	0.911439114	0.956814133	0.980334498	0.019172418	NA
van_Zyl_eye_2020_NKT	Adult	Sensory system	van_Zyl_eye_2020	NKT	Natural killer cell	CL:0000623	Eye	UBERON:0000970	0.97007141	0.802132416	0.898892989	0.956860945	0.980334498	0.019151171	NA
AdultFallopiantube_Stratified epithelial cell	Adult	Reproductive system	AdultFallopiantube	Stratified epithelial cell	Epithelial cell	CL:0000066	Fallopian tube	UBERON:0003889	0.980258831	0.790316371	0.914760148	0.957485954	0.980334498	0.018867588	NA
FetalAdrenalGland_CB CD34+	Fetal	Endocrine system	FetalAdrenalGland	CB CD34+	Hematopoietic stem cell	CL:0000037	Adrenal gland	UBERON:0002369	0.972433937	0.799373967	0.904428044	0.95749449	0.980334498	0.018863717	NA
AdultFallopiantube_Mast cell	Adult	Reproductive system	AdultFallopiantube	Mast cell	Mast cell	CL:0000097	Fallopian tube	UBERON:0003889	0.980258831	0.790357542	0.914760148	0.957498171	0.980334498	0.018862047	NA
FetalAdrenalGland_Hepatocyte/Endodermal cell	Fetal	Endocrine system	FetalAdrenalGland	Hepatocyte/Endodermal cell	Hepatocyte	CL:0000182	Adrenal gland	UBERON:0002369	0.972433937	0.799415137	0.904428044	0.957506568	0.980334498	0.018858238	NA
Madissoon_2020_lung_DC_2	Adult	Respiratory system	Madissoon_2020_lung	DC_2	Dendritic cell	CL:0000451	Lung	UBERON:0002048	0.969910229	0.805673112	0.898154982	0.957702808	0.980334498	0.018769239	NA
AdultPeripheralBlood_Antigen presenting cell (RPS high)	Adult	Lymphatic system	AdultPeripheralBlood	Antigen presenting cell (RPS high)	Professional antigen presenting cell	CL:0000145	Blood	UBERON:0000178	0.989393031	0.769689756	0.941328413	0.9579971	0.980334498	0.018635805	NA
Madissoon_2020_lung_DC_Monocyte_Dividing	Adult	Respiratory system	Madissoon_2020_lung	DC_Monocyte_Dividing	Dendritic cell	CL:0000451	Lung	UBERON:0002048	0.995026514	0.806455359	0.956826568	0.971677221	0.980334498	0.012477979	NA
AdultAdrenalGland_B cell (Plasmocyte)	Adult	Endocrine system	AdultAdrenalGland	B cell (Plasmocyte)	B cell	CL:0000236	Adrenal gland	UBERON:0002369	0.998035935	0.79538039	0.970479705	0.971745256	0.980334498	0.012447571	NA
FetalIntestine_CB CD34+	Fetal	Digestive system	FetalIntestine	CB CD34+	Hematopoietic stem cell	CL:0000037	Intestine	UBERON:0000160	0.998034049	0.796533175	0.969741697	0.971882525	0.980334498	0.012386227	NA
AdultDuodenum_Dendritic cell	Adult	Digestive system	AdultDuodenum	Dendritic cell	Dendritic cell	CL:0000451	Duodenum	UBERON:0002114	0.999956598	0.7805177	0.993357934	0.972626415	0.980334498	0.01205394	NA
TS_Vasculature_erythrocyte	Adult	Cardiovascular system	TS_Vasculature	erythrocyte	Erythrocyte	CL:0000232	Vasculature	UBERON:0002049	0.995476836	0.807608144	0.961623616	0.972951479	0.980334498	0.011908817	NA
FetalPancreas_Monocyte	Fetal	Digestive system	FetalPancreas	Monocyte	Monocyte	CL:0000576	Pancreas	UBERON:0001264	0.998608374	0.798180011	0.973431734	0.973042076	0.980334498	0.01186838	NA
FetalFemaleGonad_Dendritic cell	Fetal	Reproductive system	FetalFemaleGonad	Dendritic cell	Dendritic cell	CL:0000451	Immature gonad	UBERON:2005272	0.999517898	0.794556973	0.980811808	0.973628331	0.980334498	0.011606797	NA
FetalStomach_Dendritic cell	Fetal	Digestive system	FetalStomach	Dendritic cell	Dendritic cell	CL:0000451	Stomach	UBERON:0000945	0.999557868	0.7935277	0.982287823	0.973668643	0.980334498	0.011588816	NA
TS_Lung_plasma cell	Adult	Respiratory system	TS_Lung	plasma cell	Plasma cell	CL:0000786	Lung	UBERON:0002048	0.995832355	0.809213809	0.964575646	0.973935808	0.980334498	0.011469667	NA
AdultCerebellum_T cell	Adult	Nervous system	AdultCerebellum	T cell	T cell	CL:0000084	Cerebellum	UBERON:0002037	0.999670138	0.793815897	0.983763838	0.974029725	0.980334498	0.011427789	NA
AdultTransverseColon_B cell (Plasmocyte)	Adult	Digestive system	AdultTransverseColon	B cell (Plasmocyte)	B cell	CL:0000236	Transverse colon	UBERON:0001157	0.999511232	0.797356593	0.979704797	0.97409069	0.980334498	0.011400607	NA
AdultTransverseColon_Dendritic cell	Adult	Digestive system	AdultTransverseColon	Dendritic cell	Dendritic cell	CL:0000451	Transverse colon	UBERON:0001157	0.999511232	0.797356593	0.979704797	0.97409069	0.980334498	0.011400607	NA
AdultLiver_Dendritic cell	Adult	Digestive system	AdultLiver	Dendritic cell	Dendritic cell	CL:0000451	Liver	UBERON:0002107	0.999960284	0.786446308	0.994095941	0.974245774	0.980334498	0.011331469	NA
AdultCervix_Basal cell	Adult	Reproductive system	AdultCervix	Basal cell	Basal cell	CL:0000646	Uterine cervix	UBERON:0000002	0.999535459	0.797727131	0.981549815	0.974541664	0.980334498	0.011199589	NA
AdultJeJunum_B cell (Plasmocyte)	Adult	Digestive system	AdultJeJunum	B cell (Plasmocyte)	B cell	CL:0000236	Jejunum	UBERON:0002115	0.999839709	0.794351118	0.98597786	0.974591464	0.980334498	0.011177397	NA
FetalPancreas_Dendritic cell	Fetal	Digestive system	FetalPancreas	Dendritic cell	Dendritic cell	CL:0000451	Pancreas	UBERON:0001264	0.999652398	0.798180011	0.98302583	0.974936902	0.980334498	0.011023491	NA
FetalStomach_Fetal enterocyte	Fetal	Digestive system	FetalStomach	Fetal enterocyte	Enterocyte	CL:0000584	Stomach	UBERON:0000945	0.999915525	0.7935277	0.989667897	0.975105722	0.980334498	0.010948295	NA
AdultTrachea_B cell (Plasmocyte)	Adult	Respiratory system	AdultTrachea	B cell (Plasmocyte)	B cell	CL:0000236	Trachea	UBERON:0003126	0.999906472	0.795545074	0.988929889	0.975445046	0.980334498	0.010797193	NA
AdultIleum_Dendritic cell	Adult	Digestive system	AdultIleum	Dendritic cell	Dendritic cell	CL:0000451	Ileum	UBERON:0002116	0.999897143	0.796862542	0.987453875	0.975475427	0.980334498	0.010783667	NA
TS_Pancreas_mast cell	Adult	Digestive system	TS_Pancreas	mast cell	Mast cell	CL:0000097	Pancreas	UBERON:0001264	0.998652622	0.808719758	0.974169742	0.975706219	0.980334498	0.010680927	NA
AdultCerebellum_Macrophage	Adult	Nervous system	AdultCerebellum	Macrophage	Microglial cell	CL:0000129	Cerebellum	UBERON:0002037	0.999938445	0.793815897	0.992619926	0.975736532	0.980334498	0.010667434	NA
MacParland_2018_liver_B cell	Adult	Digestive system	MacParland_2018_liver	B cell	B cell	CL:0000236	Liver	UBERON:0002107	0.99989262	0.798962258	0.986715867	0.975829677	0.980334498	0.010625978	NA
FetalLung_T cell	Fetal	Respiratory system	FetalLung	T cell	T cell	CL:0000084	Lung	UBERON:0002048	0.999987481	0.791839694	0.996309963	0.975964798	0.980334498	0.010565847	NA
TS_Large_Intestine_intestinal tuft cell	Adult	Digestive system	TS_Large_Intestine	intestinal tuft cell	Intestinal tuft cell	CL:0019032	Large intestine	UBERON:0000059	0.99870237	0.807649315	0.977121771	0.976024967	0.980334498	0.010539073	NA
TS_Eye_lacrimal gland functional unit cell	Adult	Sensory system	TS_Eye	lacrimal gland functional unit cell	Epithelial cell	CL:0000066	Eye	UBERON:0000970	0.9987019	0.809419663	0.976383764	0.976292296	0.980334498	0.010420138	NA
TS_Prostate_stromal cell	Adult	Reproductive system	TS_Prostate	stromal cell	Stromal cell	CL:0000499	Prostate gland	UBERON:0002367	0.998708866	0.807608144	0.978597786	0.976296843	0.980334498	0.010418115	NA
FetalBrain_Fetal epithelial progenitor	Fetal	Nervous system	FetalBrain	Fetal epithelial progenitor	Epithelial fate stem cell	CL:0000036	Brain	UBERON:0000955	0.99993779	0.797562447	0.991881919	0.976476307	0.980334498	0.01033829	NA
TS_Vasculature_plasma cell	Adult	Cardiovascular system	TS_Vasculature	plasma cell	Plasma cell	CL:0000786	Vasculature	UBERON:0002049	0.998707869	0.809460834	0.977859779	0.976581967	0.980334498	0.010291299	NA
AdultLung_B cell (Plasmocyte)	Adult	Respiratory system	AdultLung	B cell (Plasmocyte)	B cell	CL:0000236	Lung	UBERON:0002048	0.999921566	0.799950359	0.990405904	0.976753769	0.980334498	0.010214904	NA
FetalIntestine_Dendritic cell	Fetal	Digestive system	FetalIntestine	Dendritic cell	Dendritic cell	CL:0000451	Intestine	UBERON:0000160	0.99998739	0.796533175	0.994833948	0.97678698	0.980334498	0.010200138	NA
AdultStomach_B cell (Plasmocyte)	Adult	Digestive system	AdultStomach	B cell (Plasmocyte)	B cell	CL:0000236	Stomach	UBERON:0000945	0.999987409	0.797480106	0.995571956	0.977141618	0.980334498	0.010042489	NA
Lake_2017_FrontalCortex_Mic	Adult	Nervous system	Lake_2017_FrontalCortex	Mic	Microglial cell	CL:0000129	Frontal cortex	UBERON:0001870	0.999900389	0.804314473	0.988191882	0.97734147	0.980334498	0.009953673	NA
TS_Prostate_monocyte	Adult	Reproductive system	TS_Prostate	monocyte	Monocyte	CL:0000576	Prostate gland	UBERON:0002367	0.999678193	0.808308049	0.984501845	0.9775622	0.980334498	0.0098556	NA
TS_Pancreas_b cell	Adult	Digestive system	TS_Pancreas	b cell	B cell	CL:0000236	Pancreas	UBERON:0001264	0.999992047	0.808966784	0.99704797	0.979955858	0.980334498	0.008793487	NA
TS_Fat_leucocyte	Adult	Integumentary system	TS_Fat	leucocyte	Leukocyte	CL:0000738	Perirenal fat	UBERON:0005406	0.999992438	0.808472733	0.997785978	0.97997822	0.980334498	0.008783576	NA
TS_Salivary_Gland_cd4-positive, alpha-beta t cell	Adult	Digestive system	TS_Salivary_Gland	cd4-positive, alpha-beta t cell	T cell	CL:0000084	Saliva-secreting gland	UBERON:0001044	0.999992464	0.808678587	0.998523985	0.980150156	0.980334498	0.008707387	NA
TS_Heart_macrophage	Adult	Cardiovascular system	TS_Heart	macrophage	Macrophage	CL:0000235	Heart	UBERON:0000948	0.999992472	0.808678587	0.999261993	0.980277552	0.980334498	0.008650942	NA
TS_Large_Intestine_goblet cell	Adult	Digestive system	TS_Large_Intestine	goblet cell	Goblet cell	CL:0000160	Large intestine	UBERON:0000059	1	0.80834922	1	0.980334498	0.980334498	0.008625714	NA

Supplementary Table 5

Worksheet 1: Enriched GO terms

ONTOLOGY	ID	Description	GeneRatio	BgRatio	pvalue	p.adjust	qvalue	geneID	Count
BP	GO:0035637	multicellular organismal signaling	14/281	203/18862	2.27753E-06	0.003878488	0.003440132	CAMK2D/ATP2B1/RYR2/ASPH/SPTBN4/AHCYL1/CACNA2D1/PAFAH1B1/CACNB4/PDE4D/ANK2/NFASC/ITPR1/BIN1	14
BP	GO:0061337	cardiac conduction	12/281	149/18862	2.44875E-06	0.003878488	0.003440132	CAMK2D/ATP2B1/RYR2/ASPH/SPTBN4/AHCYL1/CACNA2D1/CACNB4/PDE4D/ANK2/ITPR1/BIN1	12
BP	GO:0003012	muscle system process	21/281	453/18862	4.73923E-06	0.003878488	0.003440132	DTNA/CAMK2D/ATP2B1/RYR2/PARP2/NEDD4L/CALD1/ERRFI1/ASPH/SORBS1/INPP5F/CACNA2D1/BBS2/GATM/DOCK5/ACTN2/TPM1/PDE4D/ANK2/BIN1/DLG1	21
BP	GO:0032409	regulation of transporter activity	16/281	283/18862	5.75377E-06	0.003878488	0.003440132	CAMK2D/KMT2A/RYR2/NEDD4L/HECW1/DAPK1/DLG2/FHL1/AHCYL1/CACNA2D1/CACNB4/ACTN2/PKD2/PDE4D/ANK2/DLG1	16
BP	GO:0003015	heart process	16/281	289/18862	7.5E-06	0.003878488	0.003440132	CAMK2D/ATP2B1/RYR2/NEDD4L/ASPH/SPTBN4/AHCYL1/CACNA2D1/CACNB4/TPM1/PDE4D/ANK2/ITPR1/BIN1/FYN/DLG1	16
BP	GO:0032412	regulation of ion transmembrane transporter activity	15/281	257/18862	7.71816E-06	0.003878488	0.003440132	CAMK2D/RYR2/NEDD4L/HECW1/DAPK1/DLG2/FHL1/AHCYL1/CACNA2D1/CACNB4/ACTN2/PKD2/PDE4D/ANK2/DLG1	15
BP	GO:0086004	regulation of cardiac muscle cell contraction	6/281	33/18862	8.18493E-06	0.003878488	0.003440132	CAMK2D/RYR2/PDE4D/ANK2/BIN1/DLG1	6
BP	GO:0086003	cardiac muscle cell contraction	8/281	72/18862	1.15168E-05	0.00422135	0.003744244	CAMK2D/RYR2/NEDD4L/CACNA2D1/PDE4D/ANK2/BIN1/DLG1	8
BP	GO:0070252	actin-mediated cell contraction	10/281	120/18862	1.26067E-05	0.00422135	0.003744244	CAMK2D/RYR2/NEDD4L/CACNA2D1/ACTN2/TPM1/PDE4D/ANK2/BIN1/DLG1	10
BP	GO:0022898	regulation of transmembrane transporter activity	15/281	268/18862	1.27264E-05	0.00422135	0.003744244	CAMK2D/RYR2/NEDD4L/HECW1/DAPK1/DLG2/FHL1/AHCYL1/CACNA2D1/CACNB4/ACTN2/PKD2/PDE4D/ANK2/DLG1	15
BP	GO:1904062	regulation of cation transmembrane transport	17/281	340/18862	1.48071E-05	0.004465013	0.003960367	CAMK2D/RYR2/NEDD4L/HECW1/DAPK1/DLG2/FHL1/CACNA2D1/CACNB4/ACTN2/PKD2/PDE4D/ANK2/BIN1/FYN/DPP10/DLG1	17
BP	GO:1903115	regulation of actin filament-based movement	6/281	37/18862	1.63424E-05	0.004517319	0.004006761	CAMK2D/RYR2/PDE4D/ANK2/BIN1/DLG1	6
BP	GO:0008016	regulation of heart contraction	14/281	245/18862	1.9679E-05	0.004841118	0.004293964	CAMK2D/ATP2B1/RYR2/ASPH/SPTBN4/AHCYL1/CACNA2D1/CACNB4/TPM1/PDE4D/ANK2/ITPR1/BIN1/DLG1	14
BP	GO:0060047	heart contraction	15/281	279/18862	2.04328E-05	0.004841118	0.004293964	CAMK2D/ATP2B1/RYR2/NEDD4L/ASPH/SPTBN4/AHCYL1/CACNA2D1/CACNB4/TPM1/PDE4D/ANK2/ITPR1/BIN1/DLG1	15
BP	GO:0006936	muscle contraction	17/281	352/18862	2.30855E-05	0.005104979	0.004528003	DTNA/CAMK2D/ATP2B1/RYR2/NEDD4L/CALD1/ASPH/SORBS1/CACNA2D1/BBS2/DOCK5/ACTN2/TPM1/PDE4D/ANK2/BIN1/DLG1	17
BP	GO:1903522	regulation of blood circulation	15/281	290/18862	3.20112E-05	0.006636323	0.005886271	CAMK2D/ATP2B1/RYR2/ASPH/SPTBN4/AHCYL1/CACNA2D1/CACNB4/DOCK5/TPM1/PDE4D/ANK2/ITPR1/BIN1/DLG1	15
BP	GO:0098901	regulation of cardiac muscle cell action potential	5/281	27/18862	4.36974E-05	0.008526128	0.007562486	CAMK2D/RYR2/ANK2/BIN1/DLG1	5
BP	GO:0042176	regulation of protein catabolic process	17/281	383/18862	6.62933E-05	0.012216381	0.010835659	PSMD2/OGT/NEDD4L/RNF19A/HECW1/UBXN1/OPHN1/GFAP/ADAM9/PLK2/HGS/UBE3A/SNF8/FMN2/TIMP3/FYN/NRDC	17
BP	GO:0030048	actin filament-based movement	10/281	150/18862	8.59377E-05	0.015002905	0.013307243	CAMK2D/RYR2/NEDD4L/CACNA2D1/ACTN2/TPM1/PDE4D/ANK2/BIN1/DLG1	10
BP	GO:0002027	regulation of heart rate	8/281	97/18862	0.000100915	0.016736681	0.014845064	CAMK2D/RYR2/SPTBN4/CACNA2D1/TPM1/PDE4D/ANK2/BIN1	8
BP	GO:0086001	cardiac muscle cell action potential	7/281	76/18862	0.000137414	0.020004343	0.017743407	CAMK2D/RYR2/NEDD4L/CACNA2D1/ANK2/BIN1/DLG1	7
BP	GO:0086002	cardiac muscle cell action potential involved in contraction	6/281	54/18862	0.000146885	0.020004343	0.017743407	RYR2/NEDD4L/CACNA2D1/ANK2/BIN1/DLG1	6
BP	GO:0007626	locomotory behavior	10/281	161/18862	0.000154421	0.020004343	0.017743407	SLC4A10/SPG11/SPTBN4/INPP5F/ARRDC3/VPS13A/PAFAH1B1/GAD1/KALRN/LGI4	10
BP	GO:0043547	positive regulation of GTPase activity	17/281	411/18862	0.00015546	0.020004343	0.017743407	ELMOD1/STXBP5L/RAPGEF2/ERRFI1/AGFG1/ARHGAP21/NF1/OPHN1/NTRK3/KALRN/DOCK5/ARHGAP32/ARHGAP26/ARAP2/RABGAP1/ARHGEF12/ARFGAP2	17
BP	GO:0086005	ventricular cardiac muscle cell action potential	5/281	35/18862	0.000159413	0.020004343	0.017743407	RYR2/NEDD4L/ANK2/BIN1/DLG1	5
BP	GO:0050808	synapse organization	17/281	412/18862	0.000160011	0.020004343	0.017743407	CNKSR2/UNC13A/NRCAM/DRP2/AFG3L2/OPHN1/NFIA/CACNB4/KALRN/SETD5/UBE3A/PCLO/NFASC/GPHN/GABRG2/FYN/DLG1	17
BP	GO:0098900	regulation of action potential	6/281	55/18862	0.000162833	0.020004343	0.017743407	CAMK2D/RYR2/CACNA2D1/ANK2/BIN1/DLG1	6
BP	GO:0035418	protein localization to synapse	7/281	80/18862	0.000189831	0.022038893	0.019548008	LGI1/DLG2/ANKS1B/MAPK8IP3/PCLO/GPHN/DLG1	7
BP	GO:0060048	cardiac muscle contraction	9/281	135/18862	0.000193558	0.022038893	0.019548008	CAMK2D/RYR2/NEDD4L/CACNA2D1/TPM1/PDE4D/ANK2/BIN1/DLG1	9
BP	GO:0070588	calcium ion transmembrane transport	14/281	304/18862	0.000199327	0.022038893	0.019548008	CAMK2D/ATP2B1/RYR2/AFG3L2/CACNA2D1/CACNB4/PKD2/PDE4D/CACNA1E/ANK2/ITPR1/BIN1/TMEM165/FYN	14
BP	GO:1902414	protein localization to cell junction	8/281	109/18862	0.000227539	0.024205024	0.021469319	LGI1/DLG2/ANKS1B/MAPK8IP3/PCLO/GPHN/KIF3A/DLG1	8
BP	GO:1901019	regulation of calcium ion transmembrane transporter activity	7/281	83/18862	0.000238883	0.024205024	0.021469319	CAMK2D/RYR2/CACNA2D1/CACNB4/PKD2/PDE4D/ANK2	7
BP	GO:1903779	regulation of cardiac conduction	6/281	59/18862	0.00024081	0.024205024	0.021469319	ATP2B1/RYR2/ASPH/AHCYL1/ANK2/ITPR1	6
BP	GO:0031056	regulation of histone modification	9/281	141/18862	0.000268119	0.026157349	0.023200987	THAP7/CAMK2D/OGT/KMT2A/TRIP12/SETD5/SART3/KDM1A/UBR5	9
BP	GO:0060401	cytosolic calcium ion transport	10/281	173/18862	0.000276689	0.026222168	0.02325848	CAMK2D/ATP2B1/RYR2/AFG3L2/CACNA2D1/PKD2/PDE4D/ANK2/ITPR1/FYN	10
BP	GO:1901379	regulation of potassium ion transmembrane transport	7/281	86/18862	0.000297663	0.026479834	0.023487024	NEDD4L/FHL1/ACTN2/ANK2/BIN1/DPP10/DLG1	7
BP	GO:0006941	striated muscle contraction	10/281	176/18862	0.000317506	0.026479834	0.023487024	DTNA/CAMK2D/RYR2/NEDD4L/CACNA2D1/TPM1/PDE4D/ANK2/BIN1/DLG1	10
BP	GO:0051899	membrane depolarization	7/281	87/18862	0.000319651	0.026479834	0.023487024	CAMK2D/NEDD4L/FHL1/CACNA2D1/CACNB4/CACNA1E/ANK2	7
BP	GO:0051592	response to calcium ion	9/281	145/18862	0.000329968	0.026479834	0.023487024	ADGRV1/CAMK2D/RYR2/TXNIP/AHCYL1/ADAM9/ANXA7/PKD2/ANXA11	9
BP	GO:1903169	regulation of calcium ion transmembrane transport	9/281	145/18862	0.000329968	0.026479834	0.023487024	CAMK2D/RYR2/CACNA2D1/CACNB4/PKD2/PDE4D/ANK2/BIN1/FYN	9
BP	GO:2001257	regulation of cation channel activity	10/281	177/18862	0.000332181	0.026479834	0.023487024	CAMK2D/DAPK1/DLG2/CACNA2D1/CACNB4/ACTN2/PKD2/PDE4D/ANK2/DLG1	10
BP	GO:1901990	regulation of mitotic cell cycle phase transition	17/281	439/18862	0.000335852	0.026479834	0.023487024	PSMD2/PSME4/USP47/ANKRD17/PCNT/FHL1/CDC16/PAFAH1B1/APPL2/PLK2/PKD2/DCTN2/CKAP5/CCP110/CNOT9/DONSON/DLG1	17
BP	GO:0031503	protein-containing complex localization	13/281	283/18862	0.000343272	0.026479834	0.023487024	LGI1/AGFG1/NCBP1/DLG2/ANKS1B/OPHN1/CLUAP1/WDR33/GPHN/KIF3A/DYNC2LI1/LTV1/DLG1	13
BP	GO:1990778	protein localization to cell periphery	14/281	329/18862	0.000443621	0.033442973	0.029663173	MACF1/PREPL/RAPGEF2/LGI1/SPTBN4/SORBS1/CLASP2/BBS2/ACTN2/ANK2/NFASC/GPHN/DPP10/DLG1	14
BP	GO:0046710	GDP metabolic process	3/281	11/18862	0.000494002	0.036413462	0.032297932	DLG2/ENTPD4/DLG1	3
BP	GO:0006418	tRNA aminoacylation for protein translation	5/281	45/18862	0.000531398	0.038318425	0.033987591	IARS1/MARS1/SARS1/FARSB/NARS1	5
BP	GO:0000209	protein polyubiquitination	14/281	337/18862	0.000562633	0.039707537	0.035219703	FBXO9/PSMD2/PSME4/HECTD2/NEDD4L/RNF19A/HECW1/MARCHF6/CDC16/TRIP12/UBE3A/RNF34/NHLRC3/UBR5	14
BP	GO:0007528	neuromuscular junction development	5/281	47/18862	0.000651216	0.042368437	0.037579862	UNC13A/AFG3L2/CACNB4/KALRN/GPHN	5
BP	GO:0033182	regulation of histone ubiquitination	3/281	12/18862	0.000651429	0.042368437	0.037579862	TRIP12/KDM1A/UBR5	3
BP	GO:0098911	regulation of ventricular cardiac muscle cell action potential	3/281	12/18862	0.000651429	0.042368437	0.037579862	RYR2/BIN1/DLG1	3
BP	GO:2000650	negative regulation of sodium ion transmembrane transporter activity	3/281	12/18862	0.000651429	0.042368437	0.037579862	CAMK2D/NEDD4L/HECW1	3
BP	GO:0034329	cell junction assembly	16/281	425/18862	0.000677304	0.042389004	0.037598105	MACF1/CAPZA1/RAPGEF2/SORBS1/CLASP2/NRCAM/OPHN1/MICALL2/ACTN2/SLK/SETD5/PCLO/VMP1/GABRG2/ILK/DLG1	16
BP	GO:0043161	proteasome-mediated ubiquitin-dependent protein catabolic process	16/281	425/18862	0.000677304	0.042389004	0.037598105	FBXO9/PSMD2/PSME4/OGT/NEDD4L/RNF19A/HECW1/TRIM9/UBXN1/UBXN4/MARCHF6/CDC16/PLK2/NEMF/RNF34/NHLRC3	16
BP	GO:0006937	regulation of muscle contraction	9/281	161/18862	0.000706843	0.043313643	0.038418239	CAMK2D/ATP2B1/RYR2/DOCK5/TPM1/PDE4D/ANK2/BIN1/DLG1	9
BP	GO:0043039	tRNA aminoacylation	5/281	48/18862	0.000718194	0.043313643	0.038418239	IARS1/MARS1/SARS1/FARSB/NARS1	5
BP	GO:0043266	regulation of potassium ion transport	7/281	100/18862	0.000743387	0.044032427	0.039055784	NEDD4L/FHL1/ACTN2/ANK2/BIN1/DPP10/DLG1	7
BP	GO:0043038	amino acid activation	5/281	49/18862	0.00079019	0.045983486	0.04078633	IARS1/MARS1/SARS1/FARSB/NARS1	5
BP	GO:0007050	cell cycle arrest	11/281	235/18862	0.000831324	0.04702568	0.041710733	CDC123/UHRF2/STRADB/MAGI2/PRKAG2/TBRG1/APBB1/PLK2/PKD2/BIN1/CNOT9	11
BP	GO:1902306	negative regulation of sodium ion transmembrane transport	3/281	13/18862	0.000837555	0.04702568	0.041710733	CAMK2D/NEDD4L/HECW1	3
BP	GO:0010880	regulation of release of sequestered calcium ion into cytosol by sarcoplasmic reticulum	4/281	29/18862	0.000853803	0.04702568	0.041710733	CAMK2D/RYR2/PDE4D/ANK2	4
BP	GO:1901987	regulation of cell cycle phase transition	17/281	478/18862	0.000873199	0.04702568	0.041710733	PSMD2/PSME4/USP47/ANKRD17/PCNT/FHL1/CDC16/PAFAH1B1/APPL2/PLK2/PKD2/DCTN2/CKAP5/CCP110/CNOT9/DONSON/DLG1	17
BP	GO:0055117	regulation of cardiac muscle contraction	6/281	75/18862	0.000882547	0.04702568	0.041710733	CAMK2D/RYR2/PDE4D/ANK2/BIN1/DLG1	6
BP	GO:0072659	protein localization to plasma membrane	12/281	275/18862	0.00090342	0.04702568	0.041710733	MACF1/PREPL/RAPGEF2/SPTBN4/SORBS1/CLASP2/BBS2/ACTN2/ANK2/NFASC/DPP10/DLG1	12
BP	GO:0097553	calcium ion transmembrane import into cytosol	8/281	134/18862	0.000907339	0.04702568	0.041710733	CAMK2D/RYR2/CACNA2D1/PKD2/PDE4D/ANK2/ITPR1/FYN	8
BP	GO:0010389	regulation of G2/M transition of mitotic cell cycle	10/281	203/18862	0.000968031	0.049166655	0.04360973	PSMD2/PSME4/USP47/PCNT/FHL1/PAFAH1B1/DCTN2/CKAP5/CCP110/DONSON	10
BP	GO:0010498	proteasomal protein catabolic process	17/281	483/18862	0.000978293	0.049166655	0.04360973	FBXO9/PSMD2/PSME4/OGT/NEDD4L/RNF19A/HECW1/TRIM9/UBXN1/UBXN4/MARCHF6/OPHN1/CDC16/PLK2/NEMF/RNF34/NHLRC3	17
MF	GO:0030507	spectrin binding	6/283	26/18337	2.27567E-06	0.000975965	0.000877443	CAMSAP2/SPTBN4/DCTN2/ANK2/KIF3A/PTPRN	6
MF	GO:0030695	GTPase regulator activity	22/283	467/18337	3.79388E-06	0.000975965	0.000877443	ELMOD1/RAPGEF5/STXBP5L/TRIO/EIF2B4/RAPGEF2/ERRFI1/AGFG1/HERC1/ARHGAP21/NF1/SH3BP5/OPHN1/KALRN/DOCK5/ARHGAP32/ARHGAP26/ARAP2/RABGAP1/ARHGEF12/DOCK3/ARF(	22
MF	GO:0044325	ion channel binding	11/283	128/18337	4.79983E-06	0.000975965	0.000877443	CAMK2D/RYR2/NEDD4L/FHL1/ACTN2/PKD2/PDE4D/ANK2/FYN/DPP10/DLG1	11
MF	GO:0005543	phospholipid binding	20/283	451/18337	2.52445E-05	0.003296498	0.002963719	SGIP1/OGT/RAPGEF2/SPTBN4/ATG2B/NF1/UNC13A/OPHN1/APPL2/ANXA7/ACTN2/ARHGAP32/ANXA11/ARHGAP26/ARAP2/PCLO/ITPR1/BIN1/PCYT1B/RNF34	20
MF	GO:0005096	GTPase activator activity	15/283	276/18337	2.70205E-05	0.003296498	0.002963719	ELMOD1/STXBP5L/RAPGEF2/ERRFI1/AGFG1/ARHGAP21/NF1/OPHN1/DOCK5/ARHGAP32/ARHGAP26/ARAP2/RABGAP1/ARHGEF12/ARFGAP2	15
MF	GO:0004842	ubiquitin-protein transferase activity	18/283	429/18337	0.000129367	0.013152271	0.011824561	UHRF2/FBXO9/HECTD2/NEDD4L/RNF19A/HECW1/TRIM9/HERC1/RNF149/MARCHF6/HERC4/HECTD4/TRIP12/UBE3A/NFX1/RNF34/NHLRC3/UBR5	18
MF	GO:0003779	actin binding	18/283	439/18337	0.000172068	0.014994519	0.013480836	SYNE1/MACF1/FMNL2/NEBL/CAPZA1/CALD1/SPTBN4/SYNPO2/SORBS1/PALLD/OPHN1/MICALL2/ACTN2/TPM1/CLMN/FMN2/BIN1/ABLIM1	18
MF	GO:0031690	adrenergic receptor binding	4/283	20/18337	0.000221416	0.016655954	0.01497455	MAGI2/RAPGEF2/ARRDC3/PDE4D	4
MF	GO:0019787	ubiquitin-like protein transferase activity	18/283	452/18337	0.000245744	0.016655954	0.01497455	UHRF2/FBXO9/HECTD2/NEDD4L/RNF19A/HECW1/TRIM9/HERC1/RNF149/MARCHF6/HERC4/HECTD4/TRIP12/UBE3A/NFX1/RNF34/NHLRC3/UBR5	18
MF	GO:0004812	aminoacyl-tRNA ligase activity	5/283	41/18337	0.000401913	0.020451172	0.018386645	IARS1/MARS1/SARS1/FARSB/NARS1	5
MF	GO:0016875	ligase activity, forming carbon-oxygen bonds	5/283	41/18337	0.000401913	0.020451172	0.018386645	IARS1/MARS1/SARS1/FARSB/NARS1	5
MF	GO:0051015	actin filament binding	11/283	208/18337	0.000402318	0.020451172	0.018386645	SYNE1/MACF1/FMNL2/NEBL/CAPZA1/MICALL2/ACTN2/TPM1/CLMN/BIN1/ABLIM1	11
MF	GO:0061630	ubiquitin protein ligase activity	13/283	294/18337	0.0006832	0.032057839	0.028821629	UHRF2/NEDD4L/RNF19A/HECW1/TRIM9/RNF149/MARCHF6/TRIP12/UBE3A/NFX1/RNF34/NHLRC3/UBR5	13
MF	GO:0017124	SH3 domain binding	8/283	128/18337	0.000843828	0.033968052	0.030539008	SGIP1/RUFY2/ERRFI1/SH3BP5/ADAM9/ABI1/CIT/DOCK3	8
MF	GO:0031005	filamin binding	3/283	13/18337	0.000927532	0.033968052	0.030539008	NEBL/SYNPO2/MICALL2	3
MF	GO:0061659	ubiquitin-like protein ligase activity	13/283	305/18337	0.000957666	0.033968052	0.030539008	UHRF2/NEDD4L/RNF19A/HECW1/TRIM9/RNF149/MARCHF6/TRIP12/UBE3A/NFX1/RNF34/NHLRC3/UBR5	13
MF	GO:0046966	thyroid hormone receptor binding	4/283	29/18337	0.000973159	0.033968052	0.030539008	JMJD1C/TACC1/TRIP12/TAF11	4
MF	GO:0005085	guanyl-nucleotide exchange factor activity	10/283	197/18337	0.001002336	0.033968052	0.030539008	RAPGEF5/TRIO/EIF2B4/RAPGEF2/HERC1/SH3BP5/KALRN/DOCK5/ARHGEF12/DOCK3	10
MF	GO:0015085	calcium ion transmembrane transporter activity	8/283	134/18337	0.001136281	0.036480587	0.032797905	ATP2B1/RYR2/CACNA2D1/CACNB4/PKD2/CACNA1E/ITPR1/TMEM165	8
MF	GO:0051371	muscle alpha-actinin binding	3/283	15/18337	0.001442268	0.043989162	0.039548496	SYNPO2/PALLD/PKD2	3
CC	GO:0042641	actomyosin	9/286	74/19520	1.31842E-06	0.000647345	0.000552349	NEBL/SYNPO2/SORBS1/PALLD/CDC42BPA/MICALL2/TPM1/ILK/ABLIM1	9
CC	GO:0001725	stress fiber	8/286	64/19520	4.18705E-06	0.000685281	0.000584718	NEBL/SYNPO2/SORBS1/PALLD/MICALL2/TPM1/ILK/ABLIM1	8
CC	GO:0097517	contractile actin filament bundle	8/286	64/19520	4.18705E-06	0.000685281	0.000584718	NEBL/SYNPO2/SORBS1/PALLD/MICALL2/TPM1/ILK/ABLIM1	8
CC	GO:0032432	actin filament bundle	8/286	73/19520	1.13477E-05	0.001275261	0.001088121	NEBL/SYNPO2/SORBS1/PALLD/MICALL2/TPM1/ILK/ABLIM1	8
CC	GO:0098984	neuron to neuron synapse	17/286	342/19520	1.29864E-05	0.001275261	0.001088121	MAGI2/CNKSR2/RNF19A/DGKI/DLG2/ANKS1B/DCLK1/DRP2/ACTN2/ARHGAP32/ABI1/PCLO/ITPR1/GPHN/DLGAP1/FYN/DLG1	17
CC	GO:0014069	postsynaptic density	16/286	313/19520	1.65858E-05	0.001357271	0.001158096	MAGI2/CNKSR2/DGKI/DLG2/ANKS1B/DCLK1/DRP2/ACTN2/ARHGAP32/ABI1/PCLO/ITPR1/GPHN/DLGAP1/FYN/DLG1	16
CC	GO:0032279	asymmetric synapse	16/286	318/19520	2.01454E-05	0.001413053	0.001205692	MAGI2/CNKSR2/DGKI/DLG2/ANKS1B/DCLK1/DRP2/ACTN2/ARHGAP32/ABI1/PCLO/ITPR1/GPHN/DLGAP1/FYN/DLG1	16
CC	GO:0099572	postsynaptic specialization	16/286	333/19520	3.52304E-05	0.002162265	0.00184496	MAGI2/CNKSR2/DGKI/DLG2/ANKS1B/DCLK1/DRP2/ACTN2/ARHGAP32/ABI1/PCLO/ITPR1/GPHN/DLGAP1/FYN/DLG1	16
CC	GO:0033268	node of Ranvier	4/286	14/19520	4.02319E-05	0.002194874	0.001872784	SPTBN4/NFASC/BIN1/DLG1	4
CC	GO:0043194	axon initial segment	4/286	16/19520	7.14755E-05	0.003509445	0.002994445	SPTBN4/NRCAM/NFASC/BIN1	4
CC	GO:0031252	cell leading edge	17/286	411/19520	0.000128265	0.005725274	0.004885109	ADGRV1/MACF1/ATP2B1/APBB1/PALLD/CDC42BPA/CLASP2/PIEZO1/PAFAH1B1/APPL2/PKD2/SLK/TPM1/ABI1/GABRG2/ILK/ABLIM1	17
CC	GO:0016528	sarcoplasm	7/286	79/19520	0.000158646	0.006491267	0.005538695	CAMK2D/FABP3/RYR2/ASPH/IRAG1/CACNA2D1/ITPR1	7
CC	GO:0044304	main axon	6/286	62/19520	0.000289962	0.010951645	0.009344527	SPTBN4/DLG2/NRCAM/NFASC/BIN1/DLG1	6
CC	GO:0016607	nuclear speck	16/286	411/19520	0.000395965	0.013887073	0.011849191	THAP7/SNRPA1/DDX42/PRPF40B/ZNF638/PSME4/MBD1/NAMPT/APBB1/API5/TRIP12/LUC7L3/SART3/YLPM1/RNF34/PNISR	16
CC	GO:0030016	myofibril	11/286	224/19520	0.000488702	0.015996843	0.013649359	SYNE1/RYR2/NEBL/CALD1/SYNPO2/PALLD/ACTN2/TPM1/ANK2/BIN1/ILK	11
CC	GO:0005938	cell cortex	13/286	301/19520	0.000528052	0.016201044	0.013823594	CALD1/ASPH/SPTBN4/UNC13A/CLASP2/NCL/PAFAH1B1/GAD1/ACTN2/PKD2/ARHGAP32/PCLO/FMN2	13
CC	GO:0016529	sarcoplasmic reticulum	6/286	70/19520	0.000560932	0.016201044	0.013823594	CAMK2D/RYR2/ASPH/IRAG1/CACNA2D1/ITPR1	6
CC	GO:0043292	contractile fiber	11/286	231/19520	0.000631564	0.017227655	0.014699553	SYNE1/RYR2/NEBL/CALD1/SYNPO2/PALLD/ACTN2/TPM1/ANK2/BIN1/ILK	11
CC	GO:0030017	sarcomere	10/286	203/19520	0.00085371	0.022061658	0.018824183	SYNE1/RYR2/NEBL/SYNPO2/PALLD/ACTN2/TPM1/ANK2/BIN1/ILK	10
CC	GO:0098978	glutamatergic synapse	12/286	308/19520	0.002054525	0.048050241	0.040999027	ATP2B1/CNKSR2/RNF19A/LGI1/ADAM23/DRP2/OPHN1/ACTN2/PCLO/DLGAP1/FYN/DLG1	12
CC	GO:0045180	basal cortex	2/286	5/19520	0.002077727	0.048050241	0.040999027	CLASP2/PKD2	2
CC	GO:0030018	Z disc	7/286	122/19520	0.002152964	0.048050241	0.040999027	RYR2/NEBL/SYNPO2/PALLD/ACTN2/ANK2/BIN1	7

Worksheet 2: Actin-related GO terms

ONTOLOGY	ID	Description	GeneRatio	BgRatio	pvalue	p.adjust	qvalue	geneID	Count
BP	GO:0070252	actin-mediated cell contraction	10/281	120/18862	1.26E-05	0.00422135	0.003744244	CAMK2D/RYR2/NEDD4L/CACNA2D1/ACTN2/TPM1/PDE4D/ANK2/BIN1/DLG1	10
BP	GO:1903115	regulation of actin filament-based movement	6/281	37/18862	1.63E-05	0.004517319	0.004006761	CAMK2D/RYR2/PDE4D/ANK2/BIN1/DLG1	6
BP	GO:0030048	actin filament-based movement	10/281	150/18862	8.59E-05	0.015002905	0.013307243	CAMK2D/RYR2/NEDD4L/CACNA2D1/ACTN2/TPM1/PDE4D/ANK2/BIN1/DLG1	10
MF	GO:0003779	actin binding	18/283	439/18337	0.0001721	0.014994519	0.013480836	SYNE1/MACF1/FMNL2/NEBL/CAPZA1/CALD1/SPTBN4/SYNPO2/SORBS1/PALLD/OPHN1/MICALL2/ACTN2/TPM1/CLMN/FMN2/BIN1/AB	18
MF	GO:0051015	actin filament binding	11/283	208/18337	0.0004023	0.020451172	0.018386645	SYNE1/MACF1/FMNL2/NEBL/CAPZA1/MICALL2/ACTN2/TPM1/CLMN/BIN1/ABLIM1	11
CC	GO:0042641	actomyosin	9/286	74/19520	1.318E-06	0.000647345	0.000552349	NEBL/SYNPO2/SORBS1/PALLD/CDC42BPA/MICALL2/TPM1/ILK/ABLIM1	9
CC	GO:0001725	stress fiber	8/286	64/19520	4.187E-06	0.000685281	0.000584718	NEBL/SYNPO2/SORBS1/PALLD/MICALL2/TPM1/ILK/ABLIM1	8
CC	GO:0097517	contractile actin filament bundle	8/286	64/19520	4.187E-06	0.000685281	0.000584718	NEBL/SYNPO2/SORBS1/PALLD/MICALL2/TPM1/ILK/ABLIM1	8
CC	GO:0032432	actin filament bundle	8/286	73/19520	1.135E-05	0.001275261	0.001088121	NEBL/SYNPO2/SORBS1/PALLD/MICALL2/TPM1/ILK/ABLIM1	8
CC	GO:0098984	neuron to neuron synapse	17/286	342/19520	1.299E-05	0.001275261	0.001088121	MAGI2/CNKSR2/RNF19A/DGKI/DLG2/ANKS1B/DCLK1/DRP2/ACTN2/ARHGAP32/ABI1/PCLO/ITPR1/GPHN/DLGAP1/FYN/DLG1	17

Worksheet 3: Actin-related genes

Symbol	Description	Ex neuronal cells enriched?
ABLIM1	Actin Binding LIM Protein 1	Yes
ACTN2	Actinin Alpha 2	
ANK2	Ankyrin 2	Yes
BIN1	Bridging Integrator 1	
CACNA2D1	Calcium Voltage-Gated Channel Auxiliary Subunit Alpha 2 Delta 1	Yes
CALD1	Caldesmon 1	
CAMK2D	Calcium/Calmodulin Dependent Protein Kinase II Delta	
CAPZA1	Capping Actin Protein Of Muscle Z-Line Subunit Alpha 1	
CLMN	Calmin	
DLG1	Discs Large MAGUK Scaffold Protein 1	Yes
FMN2	Formin 2	Yes
FMNL2	Formin-Like Protein 2	
MACF1	Microtubule Actin Crosslinking Factor 1	
MICALL2	MICAL-Like Protein 2	
NEBL	Nebulette	Yes
NEDD4L	NEDD4-Like E3 Ubiquitin Protein Ligase	Yes
OPHN1	Oligophrenin 1	
PALLD	Palladin, Cytoskeletal Associated Protein	
PDE4D	Phosphodiesterase 4D	Yes
RYR2	Ryanodine Receptor 2	Yes
SORBS1	Sorbin And SH3 Domain Containing Protein 1	Yes
SPTBN4	Spectrin Beta, Non-Erythrocytic 4	Yes
SYNE1	Spectrin Repeat Containing Nuclear Envelope Protein 1	
SYNPO2	Synaptopodin 2	Yes
TPM1	Tropomyosin 1	

Supplementary Table 6

Gene	logFC	logCPM	LR	PValue	FDR
EMP1	1.083845706	3.408872128	48.64623808	3.07E-12	6.63E-08
NPAS4	1.889010902	2.353350903	40.91832221	1.59E-10	1.14E-07
SELE	1.661186796	1.663478237	43.76276042	3.71E-11	4.01E-07
LIF	2.047941583	-1.623197845	41.94196454	9.40E-11	5.51E-07
CCL2	1.240660259	1.060354846	41.78325229	1.02E-10	5.51E-07
SLC16A6	0.382547832	2.856459672	38.97007822	4.30E-10	1.14E-06
EDN1	0.737576938	2.056060642	38.7717385	4.76E-10	1.14E-06
SNORD3C	1.987648197	7.740314152	39.31755413	3.60E-10	1.14E-06
GADD45A	0.588817553	2.244153022	39.39523973	3.46E-10	1.14E-06
LOC100286906	1.135877519	-1.235749751	39.22130268	3.78E-10	1.14E-06
TNK2-AS1	1.342031491	5.725007807	34.41529884	4.45E-09	2.05E-06
FOSL1	1.320632932	-0.523152939	36.60697322	1.45E-09	3.09E-06
SFN	1.793264864	-0.084387825	36.44205377	1.57E-09	3.09E-06
OPN1SW	1.346491621	2.781813223	35.24873712	2.90E-09	5.23E-06
ZNF300P1	-0.482719591	0.850580404	34.88110361	3.50E-09	5.83E-06
SERPINA5	1.653297466	-0.29559825	31.75321157	1.75E-08	6.49E-06
IRAIN	0.665753813	5.459000036	31.18800037	2.34E-08	8.30E-06
TNFRSF12A	1.001443444	-1.021715817	33.68449953	6.48E-09	1.00E-05
KIAA0040	0.679463439	1.64707699	32.73419252	1.06E-08	1.52E-05
CDKN1A	0.823667688	3.233538853	32.56741074	1.15E-08	1.56E-05
LINC01515	0.829198508	-0.562658412	32.42813499	1.24E-08	1.57E-05
CBLN1	0.776548378	1.629148809	29.62206672	5.25E-08	1.65E-05
LINC00667	0.210303308	4.038309371	31.88625417	1.63E-08	1.96E-05
ZNF442	0.383036539	1.295095436	31.6774257	1.82E-08	2.07E-05
MT1A	1.106985037	-1.224399324	30.92594132	2.68E-08	2.79E-05
GRHL3	0.962884604	0.189118312	30.87242231	2.76E-08	2.79E-05
F2RL1	0.526363548	0.722944017	30.81772178	2.83E-08	2.79E-05
PTGS2	0.742927691	3.11547931	28.46284008	9.55E-08	2.87E-05
ICAM1	0.62845922	1.996958711	30.6095759	3.16E-08	2.97E-05
GNL1	1.824915372	-2.517056524	30.50500301	3.33E-08	3.00E-05
STEAP4	0.751042399	2.27067439	30.17608719	3.95E-08	3.41E-05
NUP210L	-0.902109355	4.109717875	29.88355394	4.59E-08	3.82E-05
CLCF1	1.144678413	3.376812708	29.79364157	4.81E-08	3.85E-05
MGAM	0.661998087	-0.582902406	29.65119645	5.17E-08	3.99E-05
DUSP5	0.633094524	2.325365616	26.9655323	2.07E-07	5.53E-05
CXCL1	1.462404894	-1.451627708	28.67969867	8.54E-08	6.37E-05
ENTHD1	2.110436596	0.172660536	26.38180635	2.80E-07	7.05E-05
RNF122	0.672943251	0.206703352	28.39531148	9.89E-08	7.13E-05
SERPINA3	1.361323856	5.27596767	28.31036257	1.03E-07	7.21E-05
LMCD1	0.540881139	1.591131054	26.26046183	2.98E-07	7.33E-05
EVA1C	0.241334468	3.290196105	28.09646184	1.15E-07	7.80E-05
ARRDC3	0.348251867	5.562807777	27.84032277	1.32E-07	8.63E-05
SRPX2	0.570797668	3.33162239	27.78067221	1.36E-07	8.64E-05
IL18R1	0.85950917	0.674062484	25.67388192	4.04E-07	9.50E-05
CDHR1	0.427980796	4.45939664	27.39382531	1.66E-07	0.000102249
UNC13C	-0.320272741	7.252084274	27.34449014	1.70E-07	0.000102249
SERTAD1	0.706706894	0.900909787	25.43949194	4.56E-07	0.000103933
SOCS3	1.082337177	1.540418636	26.76310921	2.30E-07	0.000132364
HSPA6	1.652464538	2.567224292	26.74096604	2.33E-07	0.000132364
MEOX1	0.674305566	-1.569895976	26.31524379	2.90E-07	0.000156747
PYGM	-0.337631169	3.433300749	26.34141669	2.86E-07	0.000156747
SLC30A2	0.991778085	-1.218318773	26.15180223	3.16E-07	0.00016643
NPPA	-0.516418852	-0.075539775	26.00536037	3.40E-07	0.000175269
SLC7A2	0.487090653	4.468880935	25.90121602	3.59E-07	0.000176576
CENPO	-0.141759643	6.742379708	25.90139445	3.59E-07	0.000176576
NPTX2	0.629944403	5.014041374	24.08232529	9.23E-07	0.000180371
EGR4	0.709235216	0.986090101	23.9103056	1.01E-06	0.000193196
APOL4	0.960744911	0.247388938	25.43002655	4.59E-07	0.000220401
MAFF	0.687687896	1.895097611	25.23772408	5.07E-07	0.000233144
LINC01268	-0.462625807	1.478602003	25.26306595	5.00E-07	0.000233144
ODC1	0.254432183	4.714433875	24.96213169	5.85E-07	0.000263359
IFITM3	0.474835691	3.690532186	24.73971162	6.56E-07	0.000289537
HMGB2	0.28686888	3.583106159	24.58443685	7.11E-07	0.000307554
LOC100287036	0.907104565	0.906640558	22.62669544	1.97E-06	0.000337723
LINC00960	0.792204805	2.830060919	22.23407189	2.41E-06	0.000392516
APBB1IP	-0.46896488	2.59555427	23.99986417	9.63E-07	0.000408436
TIPARP	0.368661787	4.876614731	22.07830993	2.62E-06	0.00041326
IL1RL1	1.832908012	2.280989333	23.74712157	1.10E-06	0.000456783
SLC25A25	0.294522605	4.840663745	21.79018579	3.04E-06	0.000469915
ANXA2	0.592180913	3.423863252	23.62425341	1.17E-06	0.000477711
ADAD2	0.912308112	-0.316886762	21.66037633	3.25E-06	0.000484467
LOC105371485	0.54477732	4.012848848	21.55702011	3.43E-06	0.000501967
PCSK1	0.48350444	5.576050868	21.38350706	3.76E-06	0.000538589
FBLIM1	0.527660004	-0.402693995	23.27781216	1.40E-06	0.000561378
CD163	0.882342929	2.734992561	21.15060396	4.25E-06	0.000600222
FPR2	1.109566061	-1.880475769	21.12236098	4.31E-06	0.000605178
PLCB2	-0.272727822	3.419730631	22.95141861	1.66E-06	0.000653141
S100A9	1.07340268	1.144008407	22.89337971	1.71E-06	0.000661142
FPR1	0.684761578	1.525944501	20.65929487	5.49E-06	0.000732618
XIRP1	0.728661815	-1.289337933	20.63748933	5.55E-06	0.000734605
GIPC3	-0.455311244	-0.927992845	22.65644965	1.94E-06	0.000734768
EPHA2	0.61338855	0.444287263	22.57789829	2.02E-06	0.000752235
FKBP5	0.502644885	4.363981195	22.5248562	2.07E-06	0.000759999
DNAJC15	-0.179923206	4.730012461	22.49304633	2.11E-06	0.000759999
CETP	-0.601361776	-1.655451058	22.41798311	2.19E-06	0.000777331
PDPR	0.274004738	5.318075945	22.32782222	2.30E-06	0.000785488
MFHAS1	0.220089707	3.163477461	22.34499394	2.28E-06	0.000785488
THBS1	0.511580521	5.582194242	22.30573493	2.33E-06	0.000785488
RFX5	-0.145754933	3.740622676	22.16068583	2.51E-06	0.000834093
ARC	0.643471316	1.715049828	20.25486294	6.78E-06	0.000862413
LINC00400	-0.280260046	0.262371555	21.97329796	2.76E-06	0.000905691
GADD45B	0.650178388	3.132942022	21.44846753	3.63E-06	0.001172911
COL6A5	0.965558937	-1.223492389	19.4208735	1.05E-05	0.001206387
OSMR	0.452210614	3.271295567	21.35883263	3.81E-06	0.001210973
CDH5	0.427451128	3.0829026	21.29398831	3.94E-06	0.001234481
FAM111B	0.849081211	-2.506902971	19.31347709	1.11E-05	0.001249591
KIF19	0.622348831	1.605813949	21.11674785	4.32E-06	0.001310092
RCSD1	-0.226505787	2.459345675	21.09848332	4.36E-06	0.001310092
LINC00499	-0.579779476	-0.193899706	21.14961096	4.25E-06	0.001310092

ERRFI1	0.282834901	3.846539336	19.11897357	1.23E-05	0.001348502
LOC729968	-0.815909702	-2.592915632	20.97442554	4.65E-06	0.001378577
IL6	1.140923036	-1.300701462	20.82261737	5.04E-06	0.001452482
LINC02192	-0.636224165	0.205308127	20.83824031	5.00E-06	0.001452482
SERPING1	0.319663036	2.957454151	20.71430567	5.33E-06	0.001483892
GRIK1-AS1	-0.32542747	2.447951645	20.71012356	5.34E-06	0.001483892
LHFPL1	-0.366468269	1.351802233	20.70653605	5.35E-06	0.001483892
GBP1	0.471516627	3.304929957	18.81749191	1.44E-05	0.001540247
TIMP1	0.500449357	3.493571277	20.52209061	5.89E-06	0.001613283
LINC00526	-0.218423652	0.634258066	20.47089065	6.05E-06	0.001636307
TUBB6	0.395956796	6.256760561	20.41406981	6.24E-06	0.001653712
PDLIM1	0.672880126	0.078432524	20.40338234	6.27E-06	0.001653712
S100A10	0.481938338	2.304339659	18.62621903	1.59E-05	0.001669694
FAM46B	0.504240836	-0.674373019	18.61655562	1.60E-05	0.001670071
LOC100996455	-0.3954549	-1.057044664	20.32789487	6.52E-06	0.001699527
CNTNAP3	0.355003726	4.121801826	20.19211396	7.00E-06	0.001802808
LOC285556	0.787674304	1.431583346	18.37511039	1.81E-05	0.001859691
SERPIND1	0.72456853	4.568264032	19.98848672	7.79E-06	0.001958708
LOXL2	0.394250534	2.297668755	20.00792501	7.71E-06	0.001958708
ENTPD1	-0.176443713	7.697696278	19.9593045	7.91E-06	0.001965971
PLCD4	0.247714582	3.29751653	19.88130616	8.24E-06	0.002024565
LPAR6	-0.278500575	5.010824133	19.74973934	8.83E-06	0.002144457
SLC11A1	0.734599215	1.234427509	19.68614017	9.13E-06	0.002168298
ZNF718	-0.251115925	3.523547171	19.69626306	9.08E-06	0.002168298
DRAIC	-0.185866711	1.20843702	19.65011984	9.30E-06	0.002185542
PBK	0.882692581	-1.774681292	17.96717702	2.25E-05	0.002189765
KDELR3	0.315776364	4.646198931	17.88312609	2.35E-05	0.002268201
RPLP0P2	0.786574231	0.351061135	17.83964555	2.40E-05	0.002300087
FOSL2	0.487164969	3.500197115	19.51276759	9.99E-06	0.0023232
GBP2	0.525758077	3.340107903	19.48983488	1.01E-05	0.002326246
S100A8	1.018845842	0.015382513	19.4073946	1.06E-05	0.002403286
MYBPH	0.914849293	3.842629312	19.34765277	1.09E-05	0.002418189
KCTD19	0.474714994	-1.249328703	19.35051979	1.09E-05	0.002418189
LOC100506178	-0.36545757	-0.329100112	19.33621659	1.10E-05	0.002418189
AASDH	-0.126829472	4.554835189	19.26547164	1.14E-05	0.002484119
MYC	0.430016321	1.129757546	19.1876236	1.18E-05	0.002513688
F2R	0.375599643	1.369722025	19.18589139	1.19E-05	0.002513688
FAT4	-0.219943255	5.310696545	19.20771647	1.17E-05	0.002513688
MT2A	0.406796884	4.418200293	19.13864311	1.22E-05	0.002551669
MILR1	-0.456791424	0.770165342	19.07885706	1.25E-05	0.00260755
CD44	0.639072574	4.688706285	17.47343972	2.91E-05	0.002693117
LOC654780	0.752264899	-1.387966061	17.45506136	2.94E-05	0.002696234
GPR3	0.534591525	0.048763721	17.38810024	3.05E-05	0.002752967
ZNF470	-0.138148204	4.286857508	18.93460063	1.35E-05	0.002785542
HELZ2	0.524305251	1.095947844	18.88243918	1.39E-05	0.002835749
IRF1	0.417537021	4.253003851	18.79049183	1.46E-05	0.00292069
TFCP2L1	0.504371497	1.157486225	18.7932005	1.46E-05	0.00292069
LCAL1	1.009069254	-0.894207359	18.64921614	1.57E-05	0.00303296
EIF2S3B	0.524188371	0.222844994	18.65548699	1.57E-05	0.00303296
TRAF3IP3	-0.305069762	3.704280128	18.66936029	1.55E-05	0.00303296
LDHC	-1.364438528	-1.348127836	18.68443003	1.54E-05	0.00303296
ITGA5	0.476102769	3.736799746	18.62854556	1.59E-05	0.003037844
PI4KAP1	-0.567194201	1.396018921	18.61240419	1.60E-05	0.003037844
CSF3	1.642895847	-0.905210163	18.56288142	1.64E-05	0.003089788
PI4KAP2	0.315316682	2.391085814	18.54693151	1.66E-05	0.003089788
LOC105370361	1.038356411	-0.973114856	18.52696142	1.68E-05	0.003095643
MT1X	0.518233217	3.290036185	18.45303137	1.74E-05	0.003190813
EMP3	0.376759417	1.035913306	17.03975875	3.66E-05	0.003218585
CSRNP1	0.407667389	3.420340167	17.02088965	3.70E-05	0.00322519
CXCL11	1.157403075	-0.482086745	16.96006302	3.82E-05	0.003302852
CRISPLD2	0.462727227	2.418986596	18.34672002	1.84E-05	0.003317665
CD33	-0.439804304	-0.985248972	18.3468116	1.84E-05	0.003317665
SLC6A6	0.159990779	4.245614699	18.32568816	1.86E-05	0.00332677
ABCC3	0.620124434	-0.599617089	18.26331988	1.92E-05	0.003397681
VIT	-0.348886102	0.088104582	18.25428031	1.93E-05	0.003397681
PNPLA5	0.39290008	0.49417533	18.22421982	1.96E-05	0.003412224
LOC101926963	0.524350985	0.615094617	18.21542266	1.97E-05	0.003412224
LINC01503	0.36172465	-0.120092845	16.78950856	4.18E-05	0.003528695
ATP6V0A4	-0.313541547	-0.333412382	18.11179659	2.08E-05	0.003574447
LINC01265	0.783177101	-2.789207653	16.68636683	4.41E-05	0.003682675
TET3	0.299090069	4.807278034	16.6404782	4.52E-05	0.003715468
C11orf91	0.611126285	-1.977012728	18.00205554	2.21E-05	0.003756718
UGDH	0.156087909	3.209950878	16.57099542	4.69E-05	0.003834673
DMKN	0.351089335	0.882566647	17.92804055	2.29E-05	0.00387516
LOC729867	0.302878551	7.905282034	17.8083113	2.44E-05	0.004049489
SERPINH1	0.637553487	2.31172375	17.8002156	2.45E-05	0.004049489
BPIFA1	1.164595313	-3.693029977	17.82415721	2.42E-05	0.004049489
C2CD4A	0.807834093	-0.17731655	17.78450411	2.47E-05	0.004052134
MPEG1	-0.310091448	3.05671146	17.75941321	2.51E-05	0.004075056
FCGR3B	0.972836436	0.104396051	17.72390359	2.55E-05	0.004090326
TGFBI	0.535750374	2.077720345	17.73417984	2.54E-05	0.004090326
ZNF525	-0.191276549	3.036754354	17.65999086	2.64E-05	0.004198992
SPOCD1	0.697613528	-0.787540606	17.63591732	2.67E-05	0.004221438
COL4A1	0.366653729	3.175953439	17.55682286	2.79E-05	0.004368827
LINC01181	0.409136062	2.683289042	16.23508652	5.59E-05	0.004432824
LAMC2	0.483012101	2.203232179	16.22245392	5.63E-05	0.004446194
F3	0.385132669	5.72642789	16.2049891	5.68E-05	0.004471056
BAG3	0.449821937	3.808995817	17.49393557	2.88E-05	0.004483256
CXorf21	-0.491032966	-1.03643286	17.47335632	2.91E-05	0.004499677
RANBP17	0.239722162	3.203126802	17.39424864	3.04E-05	0.004657619
LOC100128239	0.381874605	0.55955248	17.36803288	3.08E-05	0.004689051
CDH16	1.261554806	-3.17695988	16.05282762	6.16E-05	0.004810104
TPST1	0.20593651	5.012802769	15.99814502	6.34E-05	0.004849926
SERTAD2	0.259398472	3.925733034	16.01342033	6.29E-05	0.004849926
RELT	0.28209458	2.380406559	17.25561592	3.27E-05	0.004939019
CYSLTR1	-0.338143809	0.695340308	17.24276307	3.29E-05	0.004939019
C1orf87	0.688668693	0.678179762	15.92683066	6.58E-05	0.004979344
HSPA7	0.961283464	1.071867882	17.11837436	3.51E-05	0.005236886
SPAAR	0.532518936	-1.210236603	17.06924294	3.60E-05	0.005337322
SECTM1	0.790996834	-1.540968496	17.05109553	3.64E-05	0.005351915

NFIL3	0.363926368	3.180182237	16.96585578	3.81E-05	0.005450359
ARAP3	0.307922539	1.711251633	16.9655112	3.81E-05	0.005450359
SOD2	0.261493463	8.036715619	16.97423448	3.79E-05	0.005450359
ITGAL	-0.343957669	0.906915147	16.97819471	3.78E-05	0.005450359
SLC12A1	-0.276282249	1.630948717	15.63310513	7.69E-05	0.005655722
HPGDS	-0.439175914	0.376792104	16.87360196	4.00E-05	0.005683107
ANGPTL4	0.529624436	3.265824989	15.58550882	7.89E-05	0.005722115
MTHFD2	0.385415501	2.589324081	15.55219036	8.03E-05	0.005767421
MMP19	0.815537351	3.826569269	16.80835976	4.14E-05	0.005805483
CHI3L1	0.682485609	5.046811075	16.81718303	4.12E-05	0.005805483
AQP10	1.241497238	-2.592876734	15.49297658	8.28E-05	0.005911656
FNDC9	0.316391906	4.685774544	15.46810992	8.39E-05	0.005950666
CANX	0.565517411	7.338356526	15.4533708	8.46E-05	0.005977654
CXCL10	0.956499053	-0.924316605	15.4366468	8.53E-05	0.006011141
CLEC17A	-0.564545862	-0.690663978	15.41433806	8.63E-05	0.006062774
BMP2	0.309271025	1.5343311	15.37405204	8.82E-05	0.006173376
IFITM1	0.453169168	2.377572386	16.66779259	4.45E-05	0.006211595
FZD6	0.240101238	3.994975252	16.65230529	4.49E-05	0.006222373
GP1BA	0.418605499	3.216697495	16.62370196	4.56E-05	0.006276682
CPED1	-0.303500019	2.948525785	16.60495475	4.60E-05	0.00629891
TM4SF1	0.41255493	3.267621479	15.3012014	9.17E-05	0.006372118
AGMO	-0.375917888	-0.249235392	15.29593584	9.19E-05	0.006372118
RDH10	0.3085177	2.855313155	15.24766998	9.43E-05	0.006479037
PTGIR	0.990928595	-3.007646794	15.2445642	9.44E-05	0.006479037
PSME4	0.234135764	6.277523365	15.24044437	9.47E-05	0.006479037
HIST1H3B	0.671463378	-0.475576793	15.19038142	9.72E-05	0.006632102
TAL1	-0.310335244	1.869357395	16.48451924	4.90E-05	0.006669729
CCNT2-AS1	-0.160941015	1.535737851	16.42677585	5.06E-05	0.006833031
LOXHD1	0.444114477	1.315425179	15.1092359	0.000101466	0.006837078
FCER2	1.081956654	-1.279628918	14.96550286	0.000109495	0.00715358
RUNDC1	0.173325648	4.257603298	14.9764067	0.000108864	0.00715358
KCTD21	-0.152258858	3.290973314	16.31247124	5.37E-05	0.007212727
HS3ST2	0.581572492	2.315939346	16.28561535	5.45E-05	0.007270519
LDLR	0.324213344	4.108604697	16.24859045	5.56E-05	0.007368504
ACKR1	0.395027674	2.045229506	16.16074417	5.82E-05	0.007671179
NEMP2	-0.149299096	3.971716222	14.7935399	0.000119946	0.007675815
ITGB3	0.377215604	1.584675955	16.09330403	6.03E-05	0.007901081
A2M	-0.205448783	7.414852818	16.07595714	6.09E-05	0.007925755
LAMB4	0.411673014	-1.166966831	16.050702	6.17E-05	0.007936549
GUCY2D	0.484253835	-0.991687805	16.05130107	6.16E-05	0.007936549
TRIM26	1.399729861	-2.121067244	16.03213858	6.23E-05	0.007967317
MPZL2	0.660633773	0.481279489	16.01605105	6.28E-05	0.007988036
PIMREG	0.805307592	-1.247909791	14.6460339	0.000129707	0.008179501
DNAJB5	0.273614156	3.665063877	14.61449596	0.000131896	0.00824541
ADGRG5	-0.421834471	-1.132308737	15.90930838	6.65E-05	0.008353135
LOC101929106	-0.544049972	1.322351431	15.91820301	6.61E-05	0.008353135
STK32A-AS1	0.362055812	-0.457180969	15.87729216	6.76E-05	0.008446535
SPSB1	0.348626841	1.621699518	15.84888703	6.86E-05	0.008479973
IQGAP3	0.482360545	0.246166661	15.82680509	6.94E-05	0.008479973
CCDC141	-0.389410021	3.603487737	15.83265372	6.92E-05	0.008479973
MIR133A1HG	-0.29047118	3.994605188	15.82655967	6.94E-05	0.008479973
GRK5	0.195968465	2.974472358	15.80459685	7.02E-05	0.008530781
STX11	0.482256236	-0.589136244	15.69148721	7.46E-05	0.008964706
COPZ2	0.267473397	0.983459996	15.68960229	7.46E-05	0.008964706
FAM85A	-0.444075286	1.124628743	15.67226633	7.53E-05	0.008997269
CTGF	0.408925756	2.976270793	15.61671301	7.76E-05	0.009114424
BMP8B	0.203685792	4.15176045	15.63629601	7.68E-05	0.009114424
TPK1	-0.128977106	2.516739369	15.62383539	7.73E-05	0.009114424
ROR1-AS1	0.734187596	-0.481104836	14.28302217	0.000157277	0.009502522
TLR2	0.43740303	3.644081	14.21409281	0.000163144	0.009748085
VCL	0.154186208	5.376726289	15.43391168	8.54E-05	0.009949514
AOAH	-0.239857222	2.523570076	15.43055358	8.56E-05	0.009949514
STAT1	0.154724606	5.948735637	14.1532483	0.000168506	0.010023414
CCDC144B	0.225652808	5.650303637	14.14837433	0.000168943	0.010023414
PLOD2	0.220109028	4.678795653	14.14258393	0.000169464	0.010023414
SELPLG	-0.369670103	2.23582758	15.39323561	8.73E-05	0.010093683
LINC00680	-0.176868858	1.463627178	15.37727659	8.80E-05	0.010125149
SNHG9	0.464655245	-1.665274917	15.31902226	9.08E-05	0.010383898
PTAFR	-0.358902159	1.858699481	15.30960966	9.13E-05	0.010383898
ADAMTSL4	0.425809347	4.915674088	15.21545484	9.59E-05	0.010708208
MSL3	-0.104830252	4.002389653	15.21427522	9.60E-05	0.010708208
PLAC8	-0.637631779	-1.408998054	15.21402652	9.60E-05	0.010708208
NCK1-AS1	-0.190020032	2.704452928	15.21217119	9.61E-05	0.010708208
PLP2	0.414507399	-0.222598367	15.18420825	9.75E-05	0.010738747
GVINP1	-0.192941704	2.942860834	15.19463374	9.70E-05	0.010738747
LINC02338	-0.611833786	-1.735840817	15.17781543	9.78E-05	0.010738747
METAP1D	-0.151583302	4.616982383	15.1529148	9.91E-05	0.010826348
BMP6	0.250620542	3.389016012	13.92740272	0.000190008	0.011012891
PCED1B-AS1	-0.385792018	0.805094393	15.08939155	0.000102538	0.011140563
SLC4A5	0.24850037	2.590034	15.0571749	0.000104303	0.011228669
GTF2IP7	0.380433415	0.116444564	15.05564634	0.000104388	0.011228669
DNAJC9	-0.121816739	4.482094304	15.04119569	0.00010519	0.011258954
TPM4	0.167812763	5.769776381	13.8714137	0.000195753	0.011291055
LTBP2	0.323107141	3.985760606	15.01229389	0.000106813	0.011376381
MAML1	0.600751189	2.716624883	14.98118078	0.000108589	0.011414206
PDLIM4	0.406751755	1.054667045	14.97859684	0.000108738	0.011414206
FAM163A	-0.52966815	0.680941042	14.97834619	0.000108752	0.011414206
TLNRD1	0.26632661	2.061276944	13.79641968	0.000203724	0.011719554
ANXA2P2	0.60174192	-1.086158414	13.77803809	0.000205727	0.011743149
LYPD3	0.409474932	-2.151633087	13.77771104	0.000205763	0.011743149
NCMAP	-0.478936965	-1.313050225	13.78678892	0.000204771	0.011743149
AZGP1	0.503108483	1.018135998	13.73423511	0.000210581	0.011986482
UBAP1	0.082952608	4.193812994	13.72517925	0.000211599	0.012012801
GGT5	0.5064406	0.817024303	14.86075838	0.000115746	0.012089585
DUOX1	0.376710253	6.019247002	14.83277439	0.000117476	0.012211302
ATXN1L	0.095220095	4.939081831	14.79423451	0.000119902	0.012403784
MT1M	0.356193696	2.609151724	14.78287073	0.000120626	0.012419334
PRX	0.357611233	1.889755029	14.76031047	0.000122078	0.012450243
TFEC	-0.39318818	1.721310024	14.76902203	0.000121515	0.012450243
MAP3K6	0.310251526	1.597609816	14.74147762	0.000123304	0.012516185

ENPEP	0.406051999	1.969690856	14.72950491	0.000124089	0.01253706
PKHD1L1	0.505398753	0.646561275	13.61770811	0.000224062	0.012613193
RRAD	0.58365348	-0.565711069	13.61398631	0.000224507	0.012613193
XBP1	0.176686262	3.828195707	13.59053374	0.000227329	0.012738668
ANXA1	0.527178829	4.071568415	14.68969426	0.000126737	0.012745069
MCEMP1	0.902523988	-1.706353268	14.65935005	0.000128794	0.012874995
GAS6-AS1	-0.277217091	4.533635265	14.65312268	0.00012922	0.012874995
SIK2	0.153906737	6.041959369	14.63933711	0.000130169	0.012910016
ETV3	0.200699543	3.762499476	13.53749031	0.000233845	0.013002738
MIR4697HG	0.345501439	4.34221186	14.59846919	0.000133023	0.013090548
KIF2B	-0.488336385	-0.024071169	14.59595493	0.0001332	0.013090548
LOC401320	-0.132634807	4.272972254	13.51819144	0.000236262	0.013103454
ITGAM	-0.328513955	2.215931773	14.5622715	0.000135603	0.013266352
MYOZ2	0.260610696	-0.284636203	13.46492859	0.000243064	0.013276473
B3GLCT	0.493763146	4.737003034	13.45130221	0.000244836	0.013304615
SUSD3	-0.449678084	-1.038950658	14.53932783	0.000137264	0.013368397
FEM1C	0.17205175	5.859298256	13.40798395	0.000250556	0.013405869
NLRP1	-0.309071795	2.105682081	14.51931244	0.00013873	0.013450592
CLEC9A	-0.484869048	-0.489284334	14.51052015	0.000139379	0.013453185
KIF26B	0.431794409	0.433077009	13.37448088	0.000255071	0.013555751
SERPINB1	0.253663301	4.129934284	14.45733255	0.000143371	0.013655577
BCL2A1	0.763532695	-1.302142518	14.46009043	0.000143161	0.013655577
FAM86B3P	0.362736264	-0.752681327	14.45755776	0.000143353	0.013655577
EGR2	0.511249017	1.362901952	13.35595224	0.000257604	0.013656785
C1S	0.249844457	3.382112121	13.29691767	0.000265843	0.013823229
PTPN1	0.116227481	4.105199952	14.42345737	0.000145973	0.013842434
C1orf54	0.221294275	1.707212726	14.40827433	0.000147154	0.013893551
TM9SF4	0.204889581	4.698469696	14.38214311	0.000149211	0.014026444
SLC13A4	0.767453443	4.278216196	14.36239342	0.000150784	0.014112981
IL1B	0.638093643	0.376540636	14.34014415	0.000152576	0.014214029
DOCK2	-0.274977212	3.204748445	14.33273061	0.000153178	0.014214029
STK4	0.166867419	5.518609214	13.19465826	0.000280748	0.014458532
CALCA	1.156376134	-3.136984348	13.1808793	0.00028282	0.014496202
METTL7B	0.318241973	0.833425168	13.13675708	0.000289559	0.014741418
CRISPLD1	0.317181692	2.987431248	13.12737759	0.000291012	0.014741418
KCNMB4	0.155998165	5.749235874	13.1317477	0.000290334	0.014741418
GEM	0.311479208	3.084134351	13.10067163	0.00029519	0.014883352
MROH8	-0.110029186	4.141384154	13.10105431	0.00029513	0.014883352
CHSY1	0.173928966	4.018459122	13.07910086	0.000298609	0.014985864
KCNJ15	0.964324162	-2.352439247	14.21615339	0.000162966	0.015025153
PIM1	0.327803349	2.018036262	14.21218795	0.000163309	0.015025153
NAPSB	-0.489473658	1.693118129	14.20163832	0.000164227	0.015045602
HMGN2	0.177603674	4.480040065	14.18630653	0.000165571	0.015104692
SLC22A3	0.240924666	1.599969341	13.05569363	0.000302364	0.015139189
S100A6	0.281345763	4.73929056	14.1496236	0.000168831	0.015337363
ARID5A	0.375262308	0.797279185	14.1276158	0.000170818	0.015452912
STIL	-0.135985508	2.349101407	14.10496696	0.000172887	0.015574926
ZNF132	-0.138136695	3.658849423	14.09295542	0.000173994	0.015609663
LOC102724265	-0.492652198	-0.99092751	14.0790774	0.000175283	0.015660284
MMP9	0.78583089	-0.97484088	14.04381909	0.0001786	0.015825851
CRHR2	0.39293689	0.995908224	14.05003661	0.00017801	0.015825851
CCNYL1	0.181250145	4.044905688	12.92611011	0.00032403	0.015929042
SGMS2	0.336394812	5.494158277	12.91079717	0.000326692	0.015986485
ZBTB21	0.161064315	4.953301461	12.90665314	0.000327416	0.015986485
LOC100128253	0.380538395	0.164459307	14.01185608	0.000181662	0.016031447
SERPINB8	0.219897386	1.722966082	13.99862591	0.000182944	0.016035585
CHI3L2	0.968785673	1.799713398	13.99608395	0.000183192	0.016035585
LOC643406	0.456274816	-0.886362607	12.89571769	0.000329335	0.016043945
NRSN1	0.18145283	5.654902251	12.87723118	0.000332604	0.016130546
ITPKC	0.260436669	3.154727723	12.86083219	0.000335531	0.016236114
FER1L4	0.318179684	0.859382812	13.94431334	0.000188306	0.016416828
DUSP15	0.245925256	0.419310589	13.92026453	0.000190731	0.016561427
MLKL	0.437473347	1.032201432	12.81246975	0.000344317	0.016587018
SLC39A14	0.268464933	4.780899346	13.90516856	0.000192269	0.016628194
HOOK3	0.102771106	7.207108901	12.79672583	0.000347227	0.016690021
CLRN1	0.807369031	-2.47000011	12.78508075	0.000349395	0.016719927
INPP1	0.126273529	3.69255014	13.88622918	0.000194216	0.016729682
LOC101926905	-0.625728467	-3.023652309	13.85456088	0.000197517	0.016946468
EDN3	-0.315401163	1.548663083	13.84221261	0.000198819	0.016990771
RELB	0.320544657	-0.050461897	12.69165332	0.000367291	0.017460467
ADRA2B	0.434563581	-0.380469819	13.77183416	0.000206408	0.017569845
KIF20A	0.696413877	-0.085339207	12.67585379	0.000370408	0.017570013
LOC374443	-0.193547378	1.839945476	12.66335002	0.000372893	0.017649203
FAM87B	0.412044942	3.233897159	12.6534033	0.000374883	0.01770461
PIK3C2G	-0.44827266	-0.822290468	13.74986466	0.000208836	0.017706825
GPR4	0.621466221	-0.778681621	13.73568852	0.000210418	0.017745514
ZNF616	-0.110912692	2.682852727	13.73109041	0.000210934	0.017745514
LHFPL6	0.151144312	3.992053062	12.61378109	0.000382913	0.018044469
C1orf141	0.536376411	-0.866066729	12.58725979	0.000388385	0.018262545
LINC01521	-0.165021093	2.529119844	12.58259391	0.000389356	0.018268481
EGR3	0.2800531	4.484498167	12.57102883	0.000391773	0.018342092
COL8A1	0.541415763	0.337331875	12.54528722	0.000397207	0.018476523
VTRNA1-3	0.677465419	-1.642257386	12.54658156	0.000396932	0.018476523
TNS3	-0.237632262	5.73806141	13.63642477	0.000221839	0.018590662
POTEJ	0.63087416	-0.738364652	13.57291957	0.000229472	0.018751575
HSPG2	0.388601245	2.483089944	13.57665557	0.000229016	0.018751575
CFLAR-AS1	0.540330716	5.086388728	13.60386352	0.000225721	0.018751575
DOCK8	-0.292022833	4.041035835	13.58901933	0.000227512	0.018751575
CLEC7A	-0.351755312	1.296332607	13.57630119	0.000229059	0.018751575
P2RY13	-0.364800756	4.665537222	13.58556364	0.000227932	0.018751575
FREM3	-0.458785075	4.485494067	13.56999002	0.000229831	0.018751575
PDPN	0.330618225	2.48897117	13.55699108	0.000231428	0.018810904
FMOD	0.358854274	0.787447494	13.54156185	0.000233338	0.018895145
C7orf25	-0.130751022	2.976639128	13.52715739	0.000235136	0.018969679
CNN2	0.418509794	0.109654677	13.52007165	0.000236025	0.018970652
FAM105A	-0.242956164	3.108966861	13.50975135	0.000237327	0.019004618
MDK	0.338997471	1.73599823	13.49533023	0.000239158	0.019080563
GPR176	0.156419667	5.136202328	12.46208605	0.000415296	0.019276502
PLK3	0.349930105	2.071213468	13.4575755	0.000244019	0.01939681
LOC105376731	0.606259672	-2.751704005	13.44627515	0.000245493	0.019442515

GPR32	1.027001213	-3.818833975	13.43240216	0.000247315	0.019444373
S100A12	1.044713226	-2.142265712	13.43715983	0.000246689	0.019444373
LOC101927636	-0.254393245	1.148515881	13.41845298	0.000249161	0.019518522
LINC00115	0.29072094	-0.312139757	13.40277432	0.000251252	0.019565263
SPTSSA	0.181221824	3.380478478	13.40042269	0.000251568	0.019565263
HYAL2	0.268533975	2.480053058	12.40978443	0.00042709	0.019655235
LINC00943	-0.41043897	-0.050765569	13.37150294	0.000255477	0.019798059
PPP1R36	0.256174587	0.213021872	12.38508051	0.000432778	0.019874708
FAM57A	0.144302318	3.543144425	13.34621393	0.000258945	0.019995169
HPGD	-0.317972129	0.757439134	13.33712052	0.000260204	0.020020861
PLSCR1	0.29459103	3.588989658	13.32359878	0.000262087	0.020094242
CMTR2	-0.230998922	3.144652102	12.3561761	0.00043953	0.020099438
HIST1H1B	0.680033728	-2.402047233	12.31661146	0.000448945	0.020315229
S1PR3	0.262211148	3.070751883	12.31998165	0.000448135	0.020315229
FOSB	0.582187117	2.847853909	13.27737922	0.000268628	0.020323385
CDCP1	0.369530433	0.432908423	13.26290068	0.00027071	0.020323385
ZNF142	0.246533869	3.820774149	13.26277264	0.000270729	0.020323385
ELMO2	0.103405278	5.789186974	13.25636946	0.000271655	0.020323385
LINC01554	0.466625767	-0.667161515	13.26818648	0.000269948	0.020323385
BCO1	0.354923673	4.467398046	13.25707008	0.000271554	0.020323385
RCBTB2	-0.143719071	3.780110486	13.26025291	0.000271093	0.020323385
PNP	0.313156511	2.5763723	13.23142642	0.000275294	0.020510797
TSPEAR	0.549268918	-1.284091506	13.22623593	0.000276058	0.020510797
BLNK	-0.34123544	2.388595409	13.21513771	0.000277697	0.020561948
MIR3976HG	-0.506610132	-0.698725336	13.20108772	0.000279787	0.020645966
CARD11	-0.344396465	0.873338583	12.26620406	0.000461236	0.020698183
MAP3K1	-0.126937554	4.600946244	12.25692074	0.000463536	0.020758342
C2orf83	-0.558989243	-2.497921351	13.18416855	0.000282324	0.020762341
KIAA1109	-0.104745108	8.879655693	13.17736478	0.000283351	0.020767222
LATS2	0.26896568	1.907113716	13.16611741	0.000285057	0.020821662
SNORA71A	-0.406491079	1.876890068	12.24264819	0.000467095	0.020831469
C15orf62	0.358523588	4.308072795	12.22007857	0.000472779	0.020981669
PUS10	-0.13191927	4.065655655	12.21162032	0.000474928	0.020981669
RRH	-0.199973983	0.536984921	12.22346095	0.000471923	0.020981669
PTPN7	0.491591569	1.781217989	12.19954855	0.000478011	0.021057788
LOC102723665	-0.143160606	2.107721541	13.13277177	0.000290175	0.021124166
CNN3	0.187497925	5.987917972	12.17885119	0.000483344	0.021156996
CYR61	0.402297699	3.060337238	12.16828237	0.00048609	0.021156996
SYNDIG1	-0.174591267	3.605305567	12.16812451	0.000486132	0.021156996
CLEC4E	0.722767652	-0.975097833	12.14599436	0.000491934	0.021314044
SH3RF2	0.509334185	1.316372979	12.14310665	0.000492696	0.021314044
CXCL2	0.533153994	-0.230980051	12.1270425	0.000496959	0.021455523
GAST	0.904833253	-3.590801693	12.11652423	0.00049977	0.021491091
LIMS1	0.192674034	3.462205168	12.11898552	0.00049911	0.021491091
MIR7641-2	1.127503855	3.344954292	13.09105929	0.000296709	0.021499578
ST3GAL6	-0.163821005	3.847997015	13.08719745	0.000297321	0.021499578
GNLY	-0.57145292	-0.959154238	13.0598448	0.000301694	0.021743118
LPAR5	-0.285922193	0.789867898	12.07843326	0.000510084	0.021847775
SLC44A3	0.229762532	2.825845447	13.03203333	0.000306208	0.021922255
TUBA3D	0.407438447	2.849663985	13.03652036	0.000305475	0.021922255
LONRF3	0.21063071	1.88928291	13.02346244	0.000307613	0.021950134
CLEC4A	-0.171148858	0.987385777	13.01346792	0.000309259	0.021995007
FSTL3	0.282046553	2.125019073	12.05535045	0.000516439	0.02203271
APOL6	0.243030586	3.805357508	12.05679805	0.000516039	0.02203271
PGM5	-0.251649002	2.446981858	12.99801689	0.000311821	0.022104534
IL1R1	0.365138446	2.271440462	12.98210704	0.000314482	0.022220302
OASL	0.473158255	-1.212812145	12.0246887	0.000525005	0.022267541
TCTA	-0.119601493	3.144773831	12.03125535	0.000523158	0.022267541
ZEB1-AS1	-0.145833215	2.897544815	12.00806189	0.000529709	0.02229876
CYB5RL	-0.153979179	1.648987666	12.00741881	0.000529892	0.02229876
C9orf152	0.638733462	-3.134023085	12.96338225	0.000317643	0.022370538
LOC100288181	0.197324348	2.662744731	12.95708496	0.000318713	0.022371458
LOC101927948	-0.766919158	-1.906309781	12.9511485	0.000319725	0.022371458
FAT2	0.536258693	6.684982259	12.9252725	0.000324175	0.02260967
FAM95C	0.385536741	2.473520193	12.91810158	0.00032542	0.022623473
TBC1D8	0.107770473	5.042178325	11.94829232	0.000546975	0.022839898
LOC645485	-0.297081585	-1.011443182	11.95082794	0.000546231	0.022839898
CES4A	0.213488502	4.368939286	12.87954631	0.000332193	0.023020319
MC1R	0.264679082	0.985644533	12.86996416	0.000333898	0.023064565
CD59	0.1368338	7.169953624	11.9216389	0.000554856	0.023079894
KCNK10	0.217825104	3.472561485	12.83812349	0.000339628	0.023385662
LOC100129055	0.288876021	-0.194995014	12.81718985	0.000343449	0.02342865
HBEGF	0.248726472	2.49256981	12.8168958	0.000343503	0.02342865
NR2F2-AS1	-0.272574872	1.35789009	12.828437	0.000341391	0.02342865
SNTB2	0.20187975	5.403234993	12.80660669	0.000345397	0.023483769
CXCL8	0.803337633	0.63917015	12.77858334	0.00035061	0.023763468
CYP4Z1	-0.169143624	2.169678489	12.77032832	0.000352161	0.023793979
GPD1	-0.273622911	2.882933173	11.85467687	0.000575167	0.02387882
RPA2	-0.144537781	3.916938462	11.85018357	0.000576557	0.023890651
LINC00672	-0.180003385	3.958511557	12.75126361	0.000355769	0.023962853
MXD1	0.146435771	5.203984367	12.72412516	0.000360969	0.024237589
PXDC1	0.250093427	2.267839331	12.71831136	0.000362093	0.024237782
ADGRG2	0.334457302	3.206565658	11.81615713	0.000587189	0.024238367
TES	0.30722979	0.164734063	11.79833614	0.000592837	0.024378441
HILPDA	0.44272909	1.996560785	11.78368871	0.00059752	0.024444224
ALOX15P1	0.347216758	-1.107971174	12.68995747	0.000367625	0.024456653
LOC101927967	-0.437779499	1.002887504	12.69290761	0.000367045	0.024456653
ABCG2	-0.300963202	4.995044357	12.67867212	0.00036985	0.024529232
ZBTB5	0.096529053	4.040552048	12.66308753	0.000372946	0.024658904
ANKRD1	0.625885019	-2.083770735	12.64265116	0.000377045	0.024853926
TLR6	-0.297474847	2.112033859	12.62284572	0.000381061	0.025042295
SKI	0.228833114	4.718186177	12.61353085	0.000382964	0.025054432
LINC01736	-0.422132815	-0.604580463	12.61061121	0.000383563	0.025054432
ARSG	0.126413883	4.86440685	12.59615207	0.000386542	0.025104799
TNFRSF13C	-0.427135252	-1.333819353	12.59559761	0.000386656	0.025104799
TSSK3	0.226691254	3.340713443	11.72519704	0.000616595	0.025114896
KLRA1P	-0.167781256	2.20586797	11.72182747	0.000617713	0.025114896
DNAJC3-AS1	-0.152609694	1.971621144	12.58380162	0.000389104	0.025188106
BAALC-AS1	0.380612999	3.890277074	12.56667402	0.000392687	0.025268696
ADAM28	-0.287152755	4.076115908	12.57155375	0.000391663	0.025268696

TCEANC	-0.137593091	1.827059679	11.69391616	0.000627048	0.025446613
TYMP	0.508274007	2.477607891	12.54723731	0.000396793	0.025457124
CHST3	0.347441919	3.502026215	12.53849629	0.000398653	0.025500817
LOC101928101	0.486146547	-1.194599313	12.51878308	0.000402881	0.02555688
EXOC3L1	0.315631364	2.42189732	12.5178821	0.000403076	0.02555688
UROS	-0.163090555	4.913743571	12.51848723	0.000402945	0.02555688
BCL6B	0.409700455	0.452480146	12.46087902	0.000415564	0.026271686
ZNF793-AS1	-0.18809292	0.300156099	11.62647435	0.000650197	0.026287397
MAPK4	0.231390167	5.262536046	11.61239878	0.000655136	0.026419595
TNFRSF11B	0.509763146	0.789838339	12.44448952	0.000419227	0.026425959
CRIM1	0.102864562	6.705725585	11.59753384	0.000660393	0.026550759
LINC01351	-0.166630607	2.093501949	12.43000543	0.000422491	0.026554276
PLIN2	0.294321781	2.148582297	12.41341667	0.00042626	0.026651485
IRF8	-0.411728631	1.453301932	12.41235512	0.000426503	0.026651485
KLF6	0.194486583	4.41299282	12.39297544	0.000430952	0.026683902
LINC00513	0.766469713	6.341632169	12.3886213	0.000431958	0.026683902
CXCL14	-0.229976759	3.854549019	12.39063026	0.000431494	0.026683902
ACY3	-0.44675996	0.457607159	12.39382159	0.000430757	0.026683902
LRRC17	-0.207453097	1.864929507	11.5820802	0.000665904	0.026722644
GIMAP8	-0.253733874	1.65481579	12.36113842	0.000438363	0.027002431
TMEM119	-0.291333485	0.488981579	11.55923159	0.000674137	0.027002935
GPR27	0.149950021	4.826605112	12.35157807	0.000440614	0.027063956
EIF1	0.099569038	6.848015504	12.34374796	0.000442466	0.027100717
LOC105373656	-0.458455455	-2.865881888	11.54674395	0.00067868	0.027134659
ACTRT3	0.25632869	0.692858296	12.3336685	0.000444861	0.027170469
SGO2	-0.160000204	1.959076288	12.29751587	0.000453562	0.027623816
SERPINE1	0.644251922	0.684076788	12.28361927	0.000456951	0.027752092
OCA2	0.237843713	1.504603163	11.48901393	0.000700088	0.027785132
SMURF1	0.104765194	5.650623057	11.48555273	0.000701393	0.02778594
CABP7	0.389003211	3.757222376	11.46585264	0.000708867	0.027940784
FABP5	0.21840222	2.864506259	11.46504111	0.000709177	0.027940784
SPN	-0.281717434	0.26609129	11.47088927	0.000706948	0.027940784
C17orf107	0.236524771	1.638032268	12.25943581	0.000462911	0.028035318
ADRB1	0.202626569	3.352903976	11.4500608	0.000714917	0.02811572
MKI67	0.656269866	-0.657311242	11.44099196	0.000718414	0.028201998
PLA1A	0.530909404	0.506031377	11.43734664	0.000719825	0.028206189
SLC26A3	0.647566223	0.195754658	12.23648275	0.000468641	0.028303029
DNAJB1	0.377828142	5.638915166	11.40787402	0.000731335	0.028502289
CCDC14	-0.097033721	4.807391295	11.41051404	0.000730296	0.028502289
DUSP2	0.33480652	1.650695916	11.3946919	0.000736543	0.028653628
SYK	-0.303689247	2.298200462	12.20177271	0.000477441	0.028754199
CEBPD	0.345137744	1.993916957	11.3815066	0.000741789	0.028792375
FAM66A	-0.351564522	-0.070690372	11.37572308	0.000744103	0.028792375
LOC158434	0.360213031	-0.002843222	11.37070675	0.000746115	0.028818685
KCNN4	0.45562587	-0.833119316	12.17843477	0.000483452	0.029035318
EGLN3	-0.151854017	4.351716502	11.34364133	0.000757067	0.029137664
LPXN	-0.115243143	2.82710416	12.14746564	0.000491546	0.029270682
ESR1	-0.375144478	5.198823266	12.15054635	0.000490735	0.029270682
ITGB7	-0.241937476	1.525815114	12.14276696	0.000492786	0.029270682
CX3CR1	-0.429558456	4.475498189	12.15427455	0.000489755	0.029270682
PTP4A1	0.17002686	5.981335614	11.32479153	0.000764791	0.029278641
LOC650226	0.580827795	0.989603817	11.32486026	0.000764763	0.029278641
CA12	0.353187794	4.452553879	12.13212526	0.000495606	0.029357531
RBM12B-AS1	0.34912646	4.383350851	11.30306319	0.000773793	0.029418255
EOGT	0.170339062	3.908757563	11.30285918	0.000773878	0.029418255
MGEA5	0.108598358	7.990084567	11.30399965	0.000773403	0.029418255
NPR1	0.412832612	-0.392125669	11.31163958	0.000770227	0.029418255
LOC105374972	-0.461938213	4.21089774	12.12135147	0.000498478	0.029446954
BIRC3	0.220080399	2.772071828	11.29574753	0.000776848	0.029457417
DRAXIN	0.343329149	-0.166269738	11.28774973	0.000780202	0.029503089
CLK1	-0.164132021	6.950708031	12.11251881	0.000500844	0.029506147
CLDN14	0.725932066	-2.787336188	12.10179804	0.000503732	0.029587087
LOC101927070	-0.340309742	-1.272087182	12.09727692	0.000504955	0.029587087
DNMBP	0.153682541	3.143116496	12.07323406	0.000511509	0.029890093
MALAT1	0.159312957	12.1068973	12.05683324	0.000516029	0.029930236
RASAL3	-0.316385248	-0.048808606	12.05928326	0.000515351	0.029930236
GAS6-AS2	-0.201387068	0.292579924	12.05567738	0.000516349	0.029930236
COL6A3	0.465642527	1.379861594	12.04019447	0.000520655	0.02993907
ITM2A	-0.231130096	5.295045187	12.04576751	0.000519101	0.02993907
FREM1	-0.232451008	2.97966978	12.04187674	0.000520186	0.02993907
TEX44	0.869697432	-3.604865298	11.24816405	0.000797018	0.029981742
CYP1B1	0.21655641	3.848942193	11.24215927	0.000799601	0.030026675
SRGAP2-AS1	-0.248007301	3.118766062	12.01341919	0.000528189	0.030291691
LRRC3	0.392671334	-0.536420783	12.00018751	0.000531952	0.030366388
LINC01378	-0.492176501	-0.916340823	11.99896823	0.0005323	0.030366388
C1R	0.434388772	1.487111555	11.98990957	0.000534894	0.030398514
SH3RF3-AS1	0.384705058	5.558665751	11.98718931	0.000535675	0.030398514
SLC2A3	0.215580865	6.272287885	11.20994508	0.000813601	0.030499472
LOC158435	0.316467558	-0.132890892	11.96227887	0.000542884	0.030574603
CNTNAP3P2	0.267751408	1.182955953	11.95696542	0.000544435	0.030574603
LAMC1	0.132334585	4.728704149	11.95851818	0.000543981	0.030574603
GAL3ST4	-0.250836149	1.113806094	11.96369866	0.000542471	0.030574603
CD109	0.209138994	4.042826027	11.95092275	0.000546203	0.030594456
ARG1	0.402740696	-0.18208545	11.92730338	0.000553172	0.030904728
GCHFR	0.220955083	3.428904774	11.16612247	0.000833046	0.031066853
HIST2H3PS2	-0.262943834	1.47558636	11.90936057	0.000558526	0.031123412
MX1	0.213037466	4.39811777	11.88785557	0.000565011	0.031148907
NEDD9	0.250144275	4.01003587	11.89286132	0.000563495	0.031148907
C11orf96	0.458091382	2.881308601	11.88398537	0.000566187	0.031148907
COL6A2	0.375681131	2.413401025	11.90157812	0.000560864	0.031148907
PLAGL2	0.205287443	2.627707489	11.88891895	0.000564689	0.031148907
RHPN2	0.191361692	2.901531356	11.15612987	0.000837545	0.031180879
PLAUR	0.362117731	1.480576682	11.87534224	0.00056882	0.031214367
GJA4	0.345611746	1.918556307	11.8649848	0.000571992	0.031307994
SUV39H2	-0.101918703	3.627280797	11.85979924	0.000573587	0.031307994
TAGAP	-0.266989953	4.917391146	11.85563784	0.00057487	0.031307994
SPHK1	0.407950756	0.178565822	11.13384576	0.000847667	0.031426939
C14orf39	0.532975264	-1.762668955	11.13117421	0.000848888	0.031426939
CXCL3	0.621837144	-2.858977988	11.12882152	0.000849966	0.031426939
MMP8	1.339015279	-1.545100053	11.84378539	0.000578541	0.031428731

LINC01257	0.746757364	-1.262009188	11.83256615	0.000582037	0.031490409
CRLF3	-0.093196875	7.438393652	11.83080088	0.000582589	0.031490409
LGALS3	0.217021186	2.474407385	11.10943392	0.000858897	0.031541499
LINC00173	0.264470449	0.170966544	11.81430464	0.000587774	0.031691415
MMP3	0.966395284	-2.563985586	11.08732561	0.000869197	0.031830105
ITGB2-AS1	-0.323601102	-0.896464674	11.08625568	0.000869699	0.031830105
ARSJ	0.194013563	2.05209053	11.06515768	0.000879651	0.032139954
EIF4E1B	-0.292117497	0.161882575	11.06058695	0.000881822	0.032164949
TAGLN	0.387661841	5.782739571	11.77605464	0.000599975	0.032268811
ARHGAP22	-0.209325591	2.634170224	11.04473514	0.000889394	0.032386522
KCNJ14	-0.229197151	0.8868974	11.76341076	0.000604064	0.032408127
STAP2	0.225141974	0.754941521	11.03263788	0.000895217	0.03254376
GCNT3	0.703893214	-2.77715787	11.74057189	0.000611522	0.032727039
VIM	0.263215105	6.397228993	11.01828982	0.000902173	0.032741602
CTD-2270F17.1	-0.409056814	-1.59366647	11.00962318	0.000906401	0.032785029
FAM27C	0.409250809	-1.857705718	11.7202767	0.000618228	0.032922905
LINC01141	-0.291609484	0.532829803	11.72405748	0.000616973	0.032922905
GPX3	0.253222477	4.166840145	11.71183696	0.000621038	0.032957181
SUCNR1	-0.513994144	0.291669935	11.70919587	0.00062192	0.032957181
SCIN	-0.506803169	2.164549789	11.69482735	0.000626741	0.033131452
TLR10	-0.35631227	1.396551639	11.68342912	0.000630592	0.033253733
HSD17B7P2	0.20950926	1.456647769	10.97588266	0.000923053	0.033276054
GCH1	0.239640507	2.110414014	10.96055282	0.00093072	0.033441003
HSD3BP4	0.733384358	7.482167615	10.94053707	0.000940829	0.03357375
F11R	0.257418391	2.732091823	10.94393708	0.000939104	0.03357375
LACTB	0.163528083	3.289339823	10.9317917	0.00094528	0.03357375
ARHGAP24	-0.155042907	6.904779513	10.93487242	0.00094371	0.03357375
CD93	0.443731051	2.423730111	10.92225064	0.000950161	0.033654923
CLEC18B	0.369632553	-0.709818902	10.91201447	0.000955426	0.033712656
ACTG1P4	-0.183111413	2.201298378	10.90286501	0.000960156	0.033824402
LOC101927811	0.263652533	4.551361441	11.63704282	0.000646513	0.03401036
PTCRA	0.747232593	-3.651382775	10.87188842	0.000976349	0.034338918
CD4	-0.181256752	2.617227171	10.86034443	0.000982454	0.034496951
TINAGL1	0.345527708	1.634253539	10.85335295	0.00098617	0.034515962
OR1D5	0.677852361	-3.351870868	11.59340495	0.000661861	0.034733254
FRG1DP	0.665266561	-1.877629812	10.83603325	0.000995438	0.034784029
FABP4	0.644356589	-2.605464457	10.8129639	0.001007918	0.035050272
SLC22A8	0.848042312	3.682635022	11.55726041	0.000674852	0.035329245
LINGO3	0.398915534	1.998386125	11.54169201	0.000680527	0.035540264
SAT1	0.234822608	5.38009476	11.5175453	0.000689424	0.035918143
IGSF9B	0.388043117	3.571768491	10.75010813	0.001042732	0.036086878
PTPN9	0.113086626	4.245152951	10.73206983	0.001052946	0.036125865
OSGEPL1	-0.120343215	4.942425554	10.7274993	0.00105555	0.036125865
TRPC5OS	-0.256330477	1.738664095	10.73044481	0.001053871	0.036125865
TBX15	0.35063139	-0.645526778	11.49549138	0.000697652	0.036259463
STAT3	0.159031115	5.552234214	11.49019198	0.000699644	0.036275791
FRMPD3	0.303503199	2.977493119	11.48388977	0.000702021	0.036311926
IL7R	0.56482062	0.236393308	10.69039485	0.001076932	0.036625854
LPCAT2	-0.271174745	3.590571626	10.67121671	0.001088155	0.036949445
LINC01389	0.350971072	0.096702752	11.42930405	0.000722948	0.037039929
PLEKHG4	0.261873275	0.856925313	11.4379187	0.000719603	0.037039929
SLC6A20	0.503717288	1.055755895	11.43294165	0.000721534	0.037039929
RERGL	-0.357870854	0.783365237	11.43992017	0.000718829	0.037039929
GAN	0.103103865	5.204594994	10.66290366	0.001093057	0.037045425
INPP5D	-0.276693578	1.830822548	10.66061977	0.001094407	0.037045425
CACNA1S	-0.283935809	0.337175967	11.41501743	0.000728528	0.037177485
SLC25A5-AS1	-0.139875648	5.954012809	11.41363244	0.000729071	0.037177485
NAMPT	0.277286268	5.485565701	11.39508441	0.000736387	0.037462174
TTN	-0.358313926	10.16649049	11.37905799	0.000742768	0.037698079
GRM4	0.334704579	2.816957155	10.61807934	0.001119873	0.037730302
MKRN2OS	0.300834359	3.124527022	10.61177703	0.001123696	0.03780024
TEAD4	0.482517043	-1.263681758	11.36348837	0.00074902	0.03792638
SYCE2	0.267592817	2.816402356	10.59787113	0.001132179	0.038026462
ADAMTSL4-AS1	0.342016994	-0.949315615	11.3405035	0.000758348	0.038219659
ERVFRD-1	-0.259616465	3.372999887	11.34370597	0.000757041	0.038219659
OXTR	0.306169409	1.985990218	11.32056444	0.000766534	0.038363967
SMTNL1	0.369782214	2.33213252	11.32215247	0.000765879	0.038363967
CLK4	-0.121366557	4.765887673	11.3217948	0.000766026	0.038363967
LTB4R	0.316180766	-1.045505312	11.31595436	0.00076844	0.038370512
SOWAHC	0.186670296	1.868671985	10.56707989	0.001151194	0.038401447
BIK	0.47999927	0.266887324	10.56543215	0.001152221	0.038401447
MAPKAPK5	0.096666027	4.531961017	10.57045427	0.001149095	0.038401447
LINC01959	-0.47154966	-0.012711882	11.29817173	0.000775835	0.038650502
ZNF233	-0.13188083	4.461350502	10.54311778	0.001166215	0.038808055
ANKRD34B	0.429367686	3.707502947	10.53619661	0.001170591	0.038834037
LOC105371998	-0.205584725	5.859914436	10.53054228	0.001174178	0.038834037
BCL11A	0.310316128	3.471061773	11.27954991	0.000783656	0.038950382
CAPG	0.260146684	2.58220675	10.50947758	0.001187638	0.039104417
C5orf17	0.618777498	-1.925750992	10.50926281	0.001187776	0.039104417
COQ5	-0.120375934	3.441335788	11.25111954	0.00079575	0.039460807
LOC284412	0.238090535	1.845287189	11.24261452	0.000799405	0.039551324
MGME1	-0.121599543	2.082377052	10.46226585	0.001218377	0.040050917
GTF2E1	-0.102189362	2.708840298	11.20787762	0.000814508	0.04020658
LOC101928295	-0.378282903	-1.345024861	11.20108229	0.000817496	0.040262162
UAP1	0.217870896	4.307182095	10.44567908	0.001229367	0.04028971
APCDD1L	0.695302798	-0.31591549	10.43573409	0.001236004	0.040445945
YBX3	0.431428539	1.937366227	11.18833638	0.000823131	0.040447541
STARD13	0.132513081	5.163606669	10.43156823	0.001238795	0.040463598
IFITM2	0.42334737	1.255637299	10.41751295	0.001248259	0.040662408
MMD2	-0.293455175	3.647068019	11.16778257	0.0008323	0.040805369
TSPAN9	0.202405796	2.569015496	11.1631591	0.000834377	0.040814638
NMB	0.264648313	0.568089404	10.4049463	0.001256782	0.040878494
ELMSAN1	0.138951576	4.307481592	11.15257971	0.000839149	0.040886109
LINC01007	-0.498723791	1.588057467	11.1515379	0.00083962	0.040886109
LOC102546294	-0.166941577	0.949303792	10.39537018	0.001263317	0.04102934
TMEM211	0.464327963	-1.502122528	11.13398147	0.000847605	0.041182155
JARID2	0.148597374	4.263064775	11.12434803	0.000852018	0.041298173
REN	-0.45228596	-1.750338745	11.12044574	0.000853813	0.041298173
P2RY2	0.380322772	0.349092528	10.37897512	0.001274584	0.0413093
ZMYND15	-0.289041947	-1.216359851	10.37727793	0.001275756	0.0413093

SORCS3	0.193793236	4.518400572	10.36885929	0.001281587	0.041346241
EGR1	0.352652278	3.685986429	10.36735485	0.001282632	0.041346241
TH	0.609689717	-1.201905658	11.10509167	0.00086091	0.04148759
GRID2IP	0.273435186	1.639028127	11.09955302	0.000863485	0.04148759
MAF	-0.195700247	4.992373421	11.10148757	0.000862585	0.04148759
RNA5S9	1.442542592	0.467664879	11.09296219	0.00086656	0.04154298
CLEC2D	-0.159844762	2.131637905	10.34206256	0.001300326	0.041719275
GIMAP1	-0.251267975	-0.345391663	10.33980293	0.001301919	0.041719275
ACTA1	0.271730914	-0.931522681	10.31846182	0.00131706	0.042017715
SPP1	0.40533422	6.702864374	10.31961594	0.001316237	0.042017715
PLPP4	0.231115421	1.682696475	10.29531958	0.001333681	0.042422809
HCAR2	0.548942664	-2.721163006	10.2919514	0.001336117	0.042437906
IFI35	0.257913808	1.559690156	10.27936347	0.001345263	0.042622963
RAB40A	-0.131838183	1.484116865	10.27851181	0.001345885	0.042622963
ZNF491	-0.132220581	1.657062192	10.275478	0.001348099	0.042630688
NFKBIZ	0.216505227	2.422067391	10.27259905	0.001350205	0.042634929
MTBP	-0.15169181	2.441052095	11.0402458	0.00089155	0.042646485
PRSS23	0.140670977	5.804566555	10.26348198	0.001356893	0.042783679
ADRA1A	-0.184004003	4.284837583	10.25241735	0.001365056	0.042853647
HMCN1	-0.283867583	1.769649074	10.25594416	0.001362449	0.042853647
DGCR11	0.250350017	0.983473523	11.02466876	0.000899073	0.042911405
PPDPF	0.342398521	0.707335051	11.01896236	0.000901845	0.042948897
MS4A15	0.837814189	-3.261376839	11.00414933	0.000909081	0.043198352
MPZL3	0.196591433	0.918550237	10.22246006	0.001387407	0.043429244
KIAA0408	0.255897814	-0.693090423	10.98557228	0.000918239	0.043458696
AQP9	0.422115795	1.831432987	10.98083322	0.00092059	0.043458696
LINC01851	-0.822832963	-3.548755882	10.98204736	0.000919987	0.043458696
ISLR2	0.363726111	2.411786339	10.97611129	0.000922939	0.043474646
ZNF285	-0.116824559	2.549509765	10.21640036	0.001391973	0.043509202
PPP1R15B	0.101220363	5.427806967	10.21309373	0.001394471	0.043524384
TNFRSF1A	0.244223106	3.01636287	10.20045053	0.001404063	0.043760653
GBP5	0.465997957	0.267662206	10.19407778	0.001408924	0.04384896
PTRHD1	-0.141461406	1.648908381	10.95434212	0.000933845	0.043892756
RGS2	0.386722872	4.973614324	10.942185	0.000939993	0.043995274
FAM222A-AS1	-0.402600792	0.216924403	10.94198094	0.000940096	0.043995274
FAM46C	0.368473571	0.777037098	10.92890688	0.000946753	0.044145567
MCL1	0.209818585	6.357905522	10.92765942	0.000947391	0.044145567
CD74	-0.244841485	5.152171363	10.17290779	0.001425193	0.044228022
C20orf196	-0.123053381	0.870285908	10.16748931	0.001429388	0.044294643
LOC101929798	0.542512943	-2.003262163	10.91289343	0.000954972	0.044298672
ATF3	0.476232576	2.033711596	10.90930641	0.000956823	0.044298672
GUSBP5	-0.232842588	0.323549461	10.9117823	0.000955545	0.044298672
LINC01622	-0.356500617	0.12132811	10.9049077	0.000959098	0.044309106
TSC22D1	0.131608333	8.060318108	10.15416006	0.00143976	0.044371501
MAOB	0.138240703	5.594573121	10.15674851	0.00143774	0.044371501
TNFSF9	0.30250411	-0.023114798	10.15375817	0.001440074	0.044371501
ZNF726	-0.136519026	1.24514043	10.16010755	0.001435122	0.044371501
EXOC3L2	0.543152609	-2.818300772	10.89724213	0.000963075	0.044397983
SLC2A9	-0.19680282	-0.110710076	10.14055498	0.001450425	0.044563481
TRPM5	0.448473048	-2.578608942	10.12690629	0.001461205	0.044831
ZNF211	-0.087739783	3.426062578	10.8561839	0.000984664	0.045292686
PINLYP	-0.164001366	1.297766023	10.8524095	0.000986673	0.045292686
LOC101927468	0.348294079	0.53433475	10.84354339	0.000991408	0.045317632
INHBA	0.454765401	2.848331617	10.84597526	0.000990107	0.045317632
PGC	0.638955208	-3.293637056	10.83713101	0.000994848	0.045378901
SELP	0.672515267	3.154598836	10.83310381	0.000997014	0.045381964
SALRNA2	-0.580183708	-3.24840395	10.09643307	0.001485566	0.045513867
SLC5A5	0.902335481	-0.412740923	10.81602507	0.001006253	0.045706292
LINC00397	0.550070105	-1.327130566	10.79885487	0.001015629	0.045843246
TUFT1	0.143476452	2.038380355	10.79994416	0.001015032	0.045843246
CFAP99	0.460895463	-0.391550752	10.80557976	0.001011946	0.045843246
IL4R	0.421848912	1.756381352	10.07794478	0.001500546	0.045907797
KIF15	-0.2178907	0.379932908	10.78923988	0.001020918	0.045985973
SEC24D	0.219083886	3.082449873	10.06938438	0.001507534	0.04605644
SPNS3	-0.335917947	-0.235799455	10.77874363	0.001026723	0.046151327
ADCY7	-0.137130378	4.992621209	10.06141527	0.001514069	0.046190841
HKDC1	-0.320983396	0.261313039	10.05429696	0.00151993	0.046304349
LIPC	-0.261029809	-0.407712624	10.75665746	0.001039049	0.046529718
MIR4458HG	-0.180830529	3.075612737	10.75595062	0.001039446	0.046529718
SOX21-AS1	-0.164902413	3.261938776	10.03765676	0.001533722	0.046658794
AGO2	0.108375005	6.470421152	10.03106878	0.001539217	0.046694619
CR1	0.399899237	-0.146642296	10.02751135	0.001542193	0.046719365
ZNF806	-0.182105469	-0.242955857	10.02139038	0.001547326	0.046809322
MIDN	0.292169958	2.854008399	10.01536655	0.001552395	0.046897078
OR10A2	-0.347939577	-0.04420488	10.01269513	0.001554649	0.046899648
YAP1	0.21914621	4.347265878	10.00132288	0.001564278	0.047124425
PRIM2	-0.137435206	1.688137979	10.69730033	0.00107292	0.047928925
CLEC5A	0.595860968	0.662159087	10.68802462	0.001078313	0.048024169
BTBD18	0.200473274	4.790854846	10.6859989	0.001079494	0.048024169
WEE1	0.338614789	1.497747574	10.68207759	0.001081785	0.048027254
THBD	0.319023196	1.43432414	10.67731397	0.001084574	0.04805242
PPP2R5B	0.12209252	4.434102506	10.66927136	0.0010893	0.048163107
CHRND	0.640806353	-2.934212551	10.65832487	0.001095766	0.04835011
VIP	-0.323882113	2.978997422	9.938330792	0.001618723	0.04856168
NLRP3	-0.157405244	1.952549465	9.921371414	0.001633706	0.048943299
WASHC2C	-0.097032009	4.179822162	10.62785518	0.001113968	0.049053179
CDNF	0.252908714	-0.415058878	10.61965725	0.001118918	0.049108307
OSER1-AS1	-0.117477508	2.665764393	10.61826112	0.001119763	0.049108307
ACP5	-0.28096841	0.204380431	9.911776729	0.001642245	0.049131064
DNAJC9-AS1	-0.122003544	5.131873802	9.900312355	0.001652507	0.049369794
LOC101929497	-0.415147996	-0.172356166	9.890797339	0.001661074	0.049514875
TYRP1	-0.511464711	1.742755879	9.889836115	0.001661942	0.049514875
ABCB4	-0.254495173	1.129002201	9.883262643	0.001667889	0.049623723
NFKBIA	0.207692512	4.304178815	9.87976851	0.00167106	0.049649752
PRPH	0.755138613	-2.495816911	10.59123024	0.001136253	0.049682931
LOC105374546	-0.275057545	-0.464087214	10.58926593	0.001137461	0.049682931
FAM182B	0.275770072	1.308462163	10.58041263	0.001142921	0.049820776
H3F3B	0.176671713	6.442498675	9.868706877	0.001681136	0.049880626
CPS1	-0.205685757	2.876839818	9.8623835	0.001686924	0.049945506

Supplementary Table 7

Worksheet 1: Highlighted neuroinflammatory and neuroimmune pathways

	ONTOLOGY	ID	Description	setSize	enrichmentScore	NES	pvalue	p.adjust	qvalues	rank	leading_edge
GO:0002684	BP	GO:0002684	positive regulation of immune system process	780	0.442817245	1.497112807	0.000100271	0.007318455	0.006585065	2646	tags=18%, list=12%, signal=17%
GO:0050776	BP	GO:0050776	regulation of immune response	725	0.42675678	1.440707476	0.000100523	0.007318455	0.006585065	3280	tags=22%, list=15%, signal=19%
GO:0045087	BP	GO:0045087	innate immune response	704	0.45004679	1.519030663	0.000100533	0.007318455	0.006585065	3531	tags=25%, list=16%, signal=22%
GO:0002263	BP	GO:0002263	cell activation involved in immune response	638	0.516972153	1.741744639	0.000100735	0.007318455	0.006585065	2071	tags=17%, list=10%, signal=16%
GO:0002366	BP	GO:0002366	leukocyte activation involved in immune response	634	0.516543394	1.740005636	0.000100735	0.007318455	0.006585065	2071	tags=17%, list=10%, signal=16%
GO:0002283	BP	GO:0002283	neutrophil activation involved in immune response	435	0.560145733	1.868893851	0.00010248	0.007318455	0.006585065	1551	tags=15%, list=7%, signal=14%
GO:0031349	BP	GO:0031349	positive regulation of defense response	294	0.556919351	1.830606464	0.000105419	0.007318455	0.006585065	1711	tags=16%, list=8%, signal=15%
GO:0002285	BP	GO:0002285	lymphocyte activation involved in immune response	162	0.525121742	1.658929006	0.000337154	0.01290064	0.011607853	2146	tags=23%, list=10%, signal=21%
GO:0070486	BP	GO:0070486	leukocyte aggregation	13	0.816729826	1.755727128	0.002114165	0.049619071	0.04464669	1765	tags=54%, list=8%, signal=49%
GO:0045651	BP	GO:0045651	positive regulation of macrophage differentiation	12	0.831917474	1.761160588	0.002133946	0.049851696	0.044856003	2234	tags=33%, list=10%, signal=30%
GO:0006954	BP	GO:0006954	inflammatory response	595	0.573929218	1.930740003	0.000100888	0.007318455	0.006585065	2621	tags=26%, list=12%, signal=23%
GO:0010942	BP	GO:0010942	positive regulation of cell death	572	0.457220778	1.536861501	0.000101051	0.007318455	0.006585065	2349	tags=15%, list=11%, signal=13%
GO:0043068	BP	GO:0043068	positive regulation of programmed cell death	514	0.470455511	1.577662599	0.000101399	0.007318455	0.006585065	2507	tags=15%, list=12%, signal=14%
**GO:0097190**	**BP**	**GO:0097190**	**apoptotic signaling pathway**	**517**	**0.4644442**	**1.55774**	**0.00010145**	**0.00732**	**0.00659**	**3785**	**tags=21%, list=17%, signal=18**
**GO:0043065**	**BP**	**GO:0043065**	**positive regulation of apoptotic process**	**500**	**0.4566162**	**1.53006**	**0.00010157**	**0.00732**	**0.00659**	**2507**	**tags=15%, list=12%, signal=13**
**GO:0002250**	**BP**	**GO:0002250**	**adaptive immune response**	**318**	**0.4998401**	**1.64827**	**0.00010474**	**0.00732**	**0.00659**	**2374**	**tags=22%, list=11%, signal=20**
GO:2001233	BP	GO:2001233	regulation of apoptotic signaling pathway	310	0.481560626	1.586646721	0.000104899	0.007318455	0.006585065	4792	tags=30%, list=22%, signal=24%
GO:0050727	BP	GO:0050727	regulation of inflammatory response	274	0.599801853	1.963467665	0.000106202	0.007318455	0.006585065	2039	tags=22%, list=9%, signal=20%
GO:0034612	BP	GO:0034612	response to tumor necrosis factor	267	0.55653409	1.818301314	0.000106644	0.007318455	0.006585065	2498	tags=21%, list=12%, signal=19%
GO:0097696	BP	GO:0097696	receptor signaling pathway via STAT	148	0.679411038	2.130704906	0.000113947	0.007318455	0.006585065	2323	tags=31%, list=11%, signal=28%
GO:0007259	BP	GO:0007259	receptor signaling pathway via JAK-STAT	141	0.684336939	2.137580952	0.000114718	0.007318455	0.006585065	2323	tags=32%, list=11%, signal=29%
GO:0034340	BP	GO:0034340	response to type I interferon	92	0.64116489	1.912257403	0.000122234	0.007318455	0.006585065	2323	tags=36%, list=11%, signal=32%
GO:1904892	BP	GO:1904892	regulation of receptor signaling pathway via STAT	92	0.698970172	2.084660136	0.000122234	0.007318455	0.006585065	2312	tags=33%, list=11%, signal=29%
GO:0071357	BP	GO:0071357	cellular response to type I interferon	88	0.649967814	1.931480626	0.000122264	0.007318455	0.006585065	2323	tags=38%, list=11%, signal=34%
GO:0060337	BP	GO:0060337	type I interferon signaling pathway	87	0.650207738	1.928392739	0.000122549	0.007318455	0.006585065	2323	tags=38%, list=11%, signal=34%
GO:0046425	BP	GO:0046425	regulation of receptor signaling pathway via JAK-STAT	86	0.704354139	2.086962967	0.000122714	0.007318455	0.006585065	2312	tags=34%, list=11%, signal=30%
GO:0002675	BP	GO:0002675	positive regulation of acute inflammatory response	21	0.828500413	1.955095131	0.000153586	0.008009678	0.00720702	354	tags=33%, list=2%, signal=33%
**GO:0150076**	**BP**	**GO:0150076**	**neuroinflammatory response**	**63**	**0.6122135**	**1.80844**	**0.00019407**	**0.01136**	**0.01055**	**810**	**tags=21%, list=4%, signal=20%**
GO:0002825	BP	GO:0002825	regulation of T-helper 1 type immune response	23	0.81809591	1.96985108	0.000301341	0.012158809	0.010940362	483	tags=30%, list=2%, signal=30%
GO:0097193	BP	GO:0097193	intrinsic apoptotic signaling pathway	254	0.487141267	1.586318076	0.000321578	0.012504165	0.011251109	2251	tags=13%, list=10%, signal=12%
GO:0034341	BP	GO:0034341	response to interferon-gamma	164	0.533354876	1.68609671	0.000336852	0.01290064	0.011607853	2323	tags=26%, list=11%, signal=24%
GO:0002286	BP	GO:0002286	T cell activation involved in immune response	96	0.584815981	1.753824348	0.000364964	0.013428977	0.012083244	2322	tags=34%, list=11%, signal=31%
GO:0042088	BP	GO:0042088	T-helper 1 type immune response	36	0.717255781	1.865107363	0.00042343	0.014786127	0.013304393	871	tags=25%, list=4%, signal=24%
**GO:0150077**	**BP**	**GO:0150077**	**regulation of neuroinflammatory response**	**32**	**0.7163296**	**1.89481**	**0.00043023**	**0.0103**	**0.00956**	**806**	**tags=28%, list=4%, signal=27%**
GO:2001235	BP	GO:2001235	positive regulation of apoptotic signaling pathway	113	0.550103718	1.680162365	0.000710985	0.022188584	0.019965042	2470	tags=18%, list=11%, signal=16%
GO:0097191	BP	GO:0097191	extrinsic apoptotic signaling pathway	199	0.50570014	1.621153925	0.000768893	0.023586483	0.021222856	4792	tags=34%, list=22%, signal=27%
GO:0045471	BP	GO:0045471	response to ethanol	114	0.542980298	1.65993643	0.000827325	0.025005736	0.022499884	2279	tags=19%, list=11%, signal=17%
**GO:0002765**	**BP**	**GO:0002765**	**immune response-inhibiting signal transduction**	**3**	**-0.9699629**	**-1.598**	**0.00145682**	**0.03749**	**0.03374**	**243**	**tags=67%, list=1%, signal=66%**
GO:0038061	BP	GO:0038061	NIK/NF-kappaB signaling	161	0.498144564	1.573901372	0.001460018	0.037509426	0.033750566	2945	tags=17%, list=14%, signal=15%
GO:0002323	BP	GO:0002323	natural killer cell activation involved in immune response	27	0.725459949	1.794909966	0.001614087	0.040329543	0.036288076	2146	tags=56%, list=10%, signal=50%
**GO:0043330**	**BP**	**GO:0043330**	**response to exogenous dsRNA**	**45**	**0.6881636**	**1.92138**	**0.000038919**	**0.00378**	**0.00351**	**2343**	**tags=38%, list=11%, signal=34**
**GO:0043331**	**BP**	**GO:0043331**	**response to dsRNA**	**52**	**0.6531638**	**1.87832**	**0.000285560**	**0.01518**	**0.0141**	**2343**	**tags=33%, list=11%, signal=29**
GO:0005132	MF	GO:0005132	type I interferon receptor binding	16	0.860832986	1.928462027	0.000158504	0.008144261	0.007328116	2146	tags=88%, list=10%, signal=79%
GO:0030029	BP	GO:0030029	actin filament-based process	726	0.41383869	1.397176381	0.000100523	0.007318455	0.006585065	3640	tags=20%, list=17%, signal=17%
GO:0030036	BP	GO:0030036	actin cytoskeleton organization	638	0.418312334	1.409347217	0.000100735	0.007318455	0.006585065	3615	tags=21%, list=17%, signal=18%
GO:0007568	BP	GO:0007568	aging	259	0.504696658	1.646052119	0.000106952	0.007318455	0.006585065	3108	tags=21%, list=14%, signal=18%

Worksheet 2: Full pathway list

	ONTOLOG	ID	Description	setSi	enrichmentScore	NES	pvalue	p.adjust	qvalues	rar	leading_edge
GO:0044092	BP	GO:0044092	negative regulation of molecular function	974	0.443688293	1.503932775	0.00010009	0.007318455	0.006585065	3771	tags=22%, list=17%, signal=19%
GO:0031401	BP	GO:0031401	positive regulation of protein modification process	951	0.460657827	1.560989414	0.0001001	0.007318455	0.006585065	3057	tags=18%, list=14%, signal=16%
GO:0048646	BP	GO:0048646	anatomical structure formation involved in morphogenesis	965	0.506150803	1.715432217	0.0001001	0.007318455	0.006585065	3571	tags=28%, list=17%, signal=25%
GO:0071345	BP	GO:0071345	cellular response to cytokine stimulus	943	0.544788189	1.846179879	0.0001001	0.007318455	0.006585065	2603	tags=23%, list=12%, signal=21%
GO:0007167	BP	GO:0007167	enzyme linked receptor protein signaling pathway	960	0.407299934	1.380269781	0.00010011	0.007318455	0.006585065	4282	tags=26%, list=20%, signal=21%
GO:0072359	BP	GO:0072359	circulatory system development	960	0.480441395	1.628133677	0.00010011	0.007318455	0.006585065	3708	tags=26%, list=17%, signal=23%
GO:0060429	BP	GO:0060429	epithelium development	992	0.480383444	1.628544716	0.00010013	0.007318455	0.006585065	3571	tags=26%, list=17%, signal=22%
GO:0051270	BP	GO:0051270	regulation of cellular component movement	919	0.421619087	1.427852884	0.00010017	0.007318455	0.006585065	4209	tags=27%, list=19%, signal=22%
GO:1902533	BP	GO:1902533	positive regulation of intracellular signal transduction	877	0.4259481	1.442518766	0.00010017	0.007318455	0.006585065	3150	tags=18%, list=15%, signal=16%
GO:0035295	BP	GO:0035295	tube development	912	0.495441515	1.677751386	0.00010018	0.007318455	0.006585065	3481	tags=27%, list=16%, signal=24%
GO:0051248	BP	GO:0051248	negative regulation of protein metabolic process	912	0.412177732	1.395788887	0.00010018	0.007318455	0.006585065	3759	tags=19%, list=17%, signal=16%
GO:0007186	BP	GO:0007186	G protein-coupled receptor signaling pathway	910	0.421595991	1.427583961	0.00010019	0.007318455	0.006585065	2742	tags=24%, list=13%, signal=22%
GO:0010562	BP	GO:0010562	positive regulation of phosphorus metabolic process	882	0.473379806	1.603099729	0.00010019	0.007318455	0.006585065	2403	tags=17%, list=11%, signal=16%
GO:0045937	BP	GO:0045937	positive regulation of phosphate metabolic process	882	0.473379806	1.603099729	0.00010019	0.007318455	0.006585065	2403	tags=17%, list=11%, signal=16%
GO:0099503	CC	GO:0099503	secretory vesicle	874	0.487384584	1.650249005	0.00010019	0.007318455	0.006585065	3417	tags=22%, list=16%, signal=19%
GO:0009887	BP	GO:0009887	animal organ morphogenesis	876	0.460296686	1.558706035	0.0001002	0.007318455	0.006585065	3387	tags=25%, list=16%, signal=22%
GO:0022603	BP	GO:0022603	regulation of anatomical structure morphogenesis	871	0.456235884	1.544767054	0.0001002	0.007318455	0.006585065	3624	tags=21%, list=17%, signal=18%
GO:0040012	BP	GO:0040012	regulation of locomotion	887	0.426414058	1.443950045	0.0001002	0.007318455	0.006585065	3777	tags=24%, list=17%, signal=21%
GO:0051241	BP	GO:0051241	negative regulation of multicellular organismal process	887	0.446107101	1.51063586	0.0001002	0.007318455	0.006585065	3999	tags=28%, list=18%, signal=24%
GO:0051704	BP	GO:0051704	multi-organism process	890	0.435747216	1.475475054	0.0001002	0.007318455	0.006585065	3710	tags=21%, list=17%, signal=18%
GO:0004888	MF	GO:0004888	transmembrane signaling receptor activity	842	0.428690122	1.450601002	0.00010021	0.007318455	0.006585065	2643	tags=25%, list=12%, signal=23%
GO:0010628	BP	GO:0010628	positive regulation of gene expression	893	0.424598416	1.437730621	0.00010021	0.007318455	0.006585065	2947	tags=19%, list=14%, signal=17%
GO:0060548	BP	GO:0060548	negative regulation of cell death	842	0.453103711	1.533211666	0.00010021	0.007318455	0.006585065	2151	tags=14%, list=10%, signal=13%
GO:0002520	BP	GO:0002520	immune system development	841	0.43967173	1.487634435	0.000100231	0.007318455	0.006585065	2997	tags=20%, list=14%, signal=18%
GO:0009790	BP	GO:0009790	embryo development	863	0.464875304	1.573596955	0.000100231	0.007318455	0.006585065	4314	tags=28%, list=20%, signal=23%
GO:0019725	BP	GO:0019725	cellular homeostasis	849	0.410988596	1.390829955	0.000100231	0.007318455	0.006585065	2626	tags=15%, list=12%, signal=13%
GO:0032269	BP	GO:0032269	negative regulation of cellular protein metabolic process	858	0.414984396	1.404479198	0.000100231	0.007318455	0.006585065	3759	tags=19%, list=17%, signal=16%
GO:0040007	BP	GO:0040007	growth	827	0.436849734	1.47766892	0.000100231	0.007318455	0.006585065	3710	tags=21%, list=17%, signal=18%
GO:0098542	BP	GO:0098542	defense response to other organism	854	0.448989354	1.519543266	0.000100231	0.007318455	0.006585065	2631	tags=20%, list=12%, signal=19%
GO:0032101	BP	GO:0032101	regulation of response to external stimulus	855	0.459746432	1.555937265	0.000100241	0.007318455	0.006585065	3140	tags=20%, list=15%, signal=18%
GO:0042327	BP	GO:0042327	positive regulation of phosphorylation	824	0.483721351	1.636090687	0.000100241	0.007318455	0.006585065	2374	tags=17%, list=11%, signal=16%
GO:2000145	BP	GO:2000145	regulation of cell motility	851	0.428398568	1.449637295	0.000100241	0.007318455	0.006585065	3777	tags=24%, list=17%, signal=21%
GO:0000165	BP	GO:0000165	MAPK cascade	788	0.421669514	1.425644917	0.000100271	0.007318455	0.006585065	3171	tags=18%, list=15%, signal=16%
GO:0002684	BP	GO:0002684	positive regulation of immune system process	780	0.442817245	1.497112807	0.000100271	0.007318455	0.006585065	2646	tags=18%, list=12%, signal=17%
GO:0009725	BP	GO:0009725	response to hormone	787	0.459559153	1.553821938	0.000100271	0.007318455	0.006585065	3970	tags=25%, list=18%, signal=21%
GO:0014070	BP	GO:0014070	response to organic cyclic compound	818	0.461749811	1.561562158	0.000100271	0.007318455	0.006585065	2952	tags=20%, list=14%, signal=18%
GO:0006887	BP	GO:0006887	exocytosis	805	0.461698076	1.560960281	0.000100281	0.007318455	0.006585065	3710	tags=21%, list=17%, signal=18%
GO:0008284	BP	GO:0008284	positive regulation of cell population proliferation	775	0.451883627	1.52761689	0.000100281	0.007318455	0.006585065	3263	tags=23%, list=15%, signal=20%
GO:0044703	BP	GO:0044703	multi-organism reproductive process	801	0.443132957	1.498087285	0.000100281	0.007318455	0.006585065	3797	tags=22%, list=18%, signal=19%
GO:0098609	BP	GO:0098609	cell-cell adhesion	778	0.425214739	1.437479792	0.000100281	0.007318455	0.006585065	3251	tags=23%, list=15%, signal=20%
GO:0030334	BP	GO:0030334	regulation of cell migration	798	0.429851964	1.453255293	0.000100291	0.007318455	0.006585065	3777	tags=24%, list=17%, signal=21%
GO:0048534	BP	GO:0048534	hematopoietic or lymphoid organ development	794	0.447782142	1.514027285	0.000100291	0.007318455	0.006585065	2997	tags=21%, list=14%, signal=18%
GO:0003006	BP	GO:0003006	developmental process involved in reproduction	772	0.424519488	1.435036374	0.000100311	0.007318455	0.006585065	3554	tags=21%, list=16%, signal=18%
GO:0043069	BP	GO:0043069	negative regulation of programmed cell death	766	0.455443425	1.539431311	0.000100311	0.007318455	0.006585065	2151	tags=15%, list=10%, signal=14%
GO:0035239	BP	GO:0035239	tube morphogenesis	748	0.497535487	1.681527377	0.000100321	0.007318455	0.006585065	3559	tags=28%, list=16%, signal=25%
GO:0033993	BP	GO:0033993	response to lipid	750	0.512461793	1.731845983	0.000100351	0.007318455	0.006585065	2940	tags=22%, list=14%, signal=19%
GO:0043066	BP	GO:0043066	negative regulation of apoptotic process	749	0.460072024	1.554807105	0.000100351	0.007318455	0.006585065	2151	tags=15%, list=10%, signal=14%
GO:0051093	BP	GO:0051093	negative regulation of developmental process	752	0.420795391	1.422245587	0.000100351	0.007318455	0.006585065	3117	tags=20%, list=14%, signal=18%
GO:0030097	BP	GO:0030097	hemopoiesis	763	0.448898307	1.51713695	0.000100371	0.007318455	0.006585065	2997	tags=20%, list=14%, signal=18%
GO:0001934	BP	GO:0001934	positive regulation of protein phosphorylation	743	0.495937957	1.675820022	0.000100402	0.007318455	0.006585065	2374	tags=18%, list=11%, signal=17%
GO:0045597	BP	GO:0045597	positive regulation of cell differentiation	739	0.44972825	1.519142637	0.000100432	0.007318455	0.006585065	3154	tags=22%, list=15%, signal=19%
GO:0045859	BP	GO:0045859	regulation of protein kinase activity	721	0.409282756	1.381878917	0.000100462	0.007318455	0.006585065	2738	tags=15%, list=13%, signal=13%
GO:0050801	BP	GO:0050801	ion homeostasis	708	0.411484442	1.389381594	0.000100472	0.007318455	0.006585065	2626	tags=16%, list=12%, signal=14%
GO:0030141	CC	GO:0030141	secretory granule	729	0.521230562	1.760007954	0.000100492	0.007318455	0.006585065	3417	tags=24%, list=16%, signal=21%
GO:0045055	BP	GO:0045055	regulated exocytosis	705	0.474098949	1.600541697	0.000100492	0.007318455	0.006585065	3710	tags=22%, list=17%, signal=19%
GO:0030029	BP	GO:0030029	actin filament-based process	726	0.41383869	1.397176381	0.000100523	0.007318455	0.006585065	3640	tags=20%, list=17%, signal=17%
GO:0050776	BP	GO:0050776	regulation of immune response	725	0.42675678	1.440707476	0.000100523	0.007318455	0.006585065	3280	tags=22%, list=15%, signal=19%
GO:0045087	BP	GO:0045087	innate immune response	704	0.45004679	1.519030663	0.000100533	0.007318455	0.006585065	3531	tags=25%, list=16%, signal=22%
GO:0043086	BP	GO:0043086	negative regulation of catalytic activity	681	0.475832956	1.604865049	0.000100664	0.007318455	0.006585065	2459	tags=17%, list=11%, signal=15%
GO:0019221	BP	GO:0019221	cytokine-mediated signaling pathway	668	0.574384435	1.93714232	0.000100675	0.007318455	0.006585065	2365	tags=24%, list=11%, signal=22%
GO:0070848	BP	GO:0070848	response to growth factor	655	0.44023099	1.483937975	0.000100685	0.007318455	0.006585065	4235	tags=28%, list=20%, signal=23%
GO:0098771	BP	GO:0098771	inorganic ion homeostasis	666	0.418833126	1.412514477	0.000100695	0.007318455	0.006585065	2626	tags=16%, list=12%, signal=15%
GO:0009986	CC	GO:0009986	cell surface	677	0.442601772	1.492446118	0.000100705	0.007318455	0.006585065	3132	tags=25%, list=14%, signal=22%
GO:0048609	BP	GO:0048609	multicellular organismal reproductive process	653	0.42547118	1.434126548	0.000100705	0.007318455	0.006585065	3797	tags=21%, list=18%, signal=18%
GO:0002443	BP	GO:0002443	leukocyte mediated immunity	664	0.512681713	1.728814188	0.000100715	0.007318455	0.006585065	1551	tags=14%, list=7%, signal=14%
GO:0032504	BP	GO:0032504	multicellular organism reproduction	665	0.421692952	1.422023541	0.000100725	0.007318455	0.006585065	3797	tags=21%, list=18%, signal=18%
GO:0002263	BP	GO:0002263	cell activation involved in immune response	638	0.516972153	1.741744639	0.000100735	0.007318455	0.006585065	2071	tags=17%, list=10%, signal=16%
GO:0002366	BP	GO:0002366	leukocyte activation involved in immune response	634	0.516543394	1.740005636	0.000100735	0.007318455	0.006585065	2071	tags=17%, list=10%, signal=16%
GO:0030036	BP	GO:0030036	actin cytoskeleton organization	638	0.418312334	1.409347217	0.000100735	0.007318455	0.006585065	3615	tags=21%, list=17%, signal=18%
GO:0030162	BP	GO:0030162	regulation of proteolysis	635	0.456820186	1.538814241	0.000100746	0.007318455	0.006585065	2370	tags=14%, list=11%, signal=13%
GO:0001944	BP	GO:0001944	vasculature development	644	0.531672547	1.791446878	0.000100766	0.007318455	0.006585065	3481	tags=28%, list=16%, signal=24%
GO:0001816	BP	GO:0001816	cytokine production	640	0.462656129	1.558722203	0.000100776	0.007318455	0.006585065	2997	tags=24%, list=14%, signal=21%
GO:0043408	BP	GO:0043408	regulation of MAPK cascade	606	0.438664477	1.476339618	0.000100827	0.007318455	0.006585065	3171	tags=19%, list=15%, signal=17%
GO:0008285	BP	GO:0008285	negative regulation of cell population proliferation	608	0.480039868	1.615579875	0.000100847	0.007318455	0.006585065	3123	tags=22%, list=14%, signal=19%
GO:0030155	BP	GO:0030155	regulation of cell adhesion	629	0.436835536	1.470903455	0.000100847	0.007318455	0.006585065	3716	tags=26%, list=17%, signal=22%
GO:0071363	BP	GO:0071363	cellular response to growth factor stimulus	629	0.438860587	1.477722165	0.000100847	0.007318455	0.006585065	4235	tags=28%, list=20%, signal=23%
GO:0001568	BP	GO:0001568	blood vessel development	616	0.523260773	1.76115047	0.000100867	0.007318455	0.006585065	3341	tags=28%, list=15%, signal=24%
GO:0001817	BP	GO:0001817	regulation of cytokine production	600	0.443858356	1.493482768	0.000100888	0.007318455	0.006585065	2997	tags=24%, list=14%, signal=21%
GO:0006954	BP	GO:0006954	inflammatory response	595	0.573929218	1.930740003	0.000100888	0.007318455	0.006585065	2621	tags=26%, list=12%, signal=23%
GO:0002274	BP	GO:0002274	myeloid leukocyte activation	578	0.535275786	1.799738681	0.000100959	0.007318455	0.006585065	1553	tags=15%, list=7%, signal=15%
GO:0040008	BP	GO:0040008	regulation of growth	584	0.474353678	1.594962017	0.000100969	0.007318455	0.006585065	3873	tags=24%, list=18%, signal=20%
GO:0010942	BP	GO:0010942	positive regulation of cell death	572	0.457220778	1.536861501	0.000101051	0.007318455	0.006585065	2349	tags=15%, list=11%, signal=13%
GO:0009611	BP	GO:0009611	response to wounding	569	0.515288476	1.731655539	0.000101082	0.007318455	0.006585065	4092	tags=32%, list=19%, signal=27%
GO:0048729	BP	GO:0048729	tissue morphogenesis	558	0.488035011	1.639460124	0.000101133	0.007318455	0.006585065	3559	tags=26%, list=16%, signal=22%
GO:0031347	BP	GO:0031347	regulation of defense response	559	0.508649785	1.708455137	0.000101163	0.007318455	0.006585065	2323	tags=18%, list=11%, signal=16%
GO:0030855	BP	GO:0030855	epithelial cell differentiation	539	0.504727235	1.694427453	0.000101215	0.007318455	0.006585065	3442	tags=29%, list=16%, signal=25%
GO:0048514	BP	GO:0048514	blood vessel morphogenesis	545	0.520714131	1.748326048	0.000101215	0.007318455	0.006585065	3481	tags=30%, list=16%, signal=25%
GO:0043068	BP	GO:0043068	positive regulation of programmed cell death	514	0.470455511	1.577662599	0.000101399	0.007318455	0.006585065	2507	tags=15%, list=12%, signal=14%
GO:0009792	BP	GO:0009792	embryo development ending in birth or egg hatching	519	0.46354212	1.554846255	0.00010142	0.007318455	0.006585065	3197	tags=20%, list=15%, signal=17%
GO:0097190	BP	GO:0097190	apoptotic signaling pathway	517	0.464444202	1.557736231	0.000101451	0.007318455	0.006585065	3785	tags=21%, list=17%, signal=18%
GO:0043009	BP	GO:0043009	chordate embryonic development	503	0.467333278	1.566559443	0.000101471	0.007318455	0.006585065	2997	tags=19%, list=14%, signal=17%
GO:0002444	BP	GO:0002444	myeloid leukocyte mediated immunity	493	0.535307953	1.793297366	0.000101523	0.007318455	0.006585065	1551	tags=15%, list=7%, signal=14%
GO:0010035	BP	GO:0010035	response to inorganic substance	493	0.45980217	1.540350776	0.000101523	0.007318455	0.006585065	2935	tags=19%, list=14%, signal=17%
GO:0040017	BP	GO:0040017	positive regulation of locomotion	498	0.455297668	1.525678123	0.000101533	0.007318455	0.006585065	2997	tags=22%, list=14%, signal=20%
GO:0002275	BP	GO:0002275	myeloid cell activation involved in immune response	490	0.527676146	1.767467574	0.000101554	0.007318455	0.006585065	1551	tags=15%, list=7%, signal=14%
GO:0043065	BP	GO:0043065	positive regulation of apoptotic process	500	0.45661615	1.530061533	0.000101574	0.007318455	0.006585065	2507	tags=15%, list=12%, signal=13%
GO:0051272	BP	GO:0051272	positive regulation of cellular component movement	499	0.46036681	1.542376849	0.000101585	0.007318455	0.006585065	2997	tags=22%, list=14%, signal=20%
GO:2000147	BP	GO:2000147	positive regulation of cell motility	487	0.462821614	1.549584753	0.000101647	0.007318455	0.006585065	2997	tags=23%, list=14%, signal=20%
GO:0071396	BP	GO:0071396	cellular response to lipid	482	0.489765545	1.639147392	0.000101729	0.007318455	0.006585065	3590	tags=22%, list=17%, signal=19%
GO:0043299	BP	GO:0043299	leukocyte degranulation	481	0.533871899	1.786655507	0.00010174	0.007318455	0.006585065	1551	tags=15%, list=7%, signal=14%
GO:0030312	CC	GO:0030312	external encapsulating structure	479	0.554323749	1.854620896	0.000101833	0.007318455	0.006585065	3888	tags=39%, list=18%, signal=33%
GO:0048598	BP	GO:0048598	embryonic morphogenesis	479	0.478232228	1.600038758	0.000101833	0.007318455	0.006585065	4430	tags=33%, list=20%, signal=27%
GO:0031012	CC	GO:0031012	extracellular matrix	478	0.552537021	1.848507646	0.000101874	0.007318455	0.006585065	3888	tags=39%, list=18%, signal=32%
GO:0002009	BP	GO:0002009	morphogenesis of an epithelium	474	0.467711985	1.564688096	0.000101895	0.007318455	0.006585065	3558	tags=25%, list=16%, signal=21%
GO:0030335	BP	GO:0030335	positive regulation of cell migration	470	0.453467063	1.516424293	0.000101895	0.007318455	0.006585065	2997	tags=22%, list=14%, signal=19%
GO:0042060	BP	GO:0042060	wound healing	460	0.547155916	1.828896754	0.000101916	0.007318455	0.006585065	4092	tags=35%, list=19%, signal=29%
GO:0009617	BP	GO:0009617	response to bacterium	463	0.510431237	1.70654679	0.000101937	0.007318455	0.006585065	2619	tags=24%, list=12%, signal=22%
GO:0001525	BP	GO:0001525	angiogenesis	467	0.531034232	1.775145842	0.000101947	0.007318455	0.006585065	3481	tags=30%, list=16%, signal=26%
GO:0048871	BP	GO:0048871	multicellular organismal homeostasis	464	0.488909998	1.634364351	0.000101978	0.007318455	0.006585065	2947	tags=22%, list=14%, signal=19%
GO:0002521	BP	GO:0002521	leukocyte differentiation	453	0.481502748	1.608582002	0.000102062	0.007318455	0.006585065	2378	tags=21%, list=11%, signal=19%
GO:0002446	BP	GO:0002446	neutrophil mediated immunity	446	0.565812964	1.889030358	0.000102229	0.007318455	0.006585065	1551	tags=15%, list=7%, signal=14%
GO:0036230	BP	GO:0036230	granulocyte activation	446	0.563542259	1.881449353	0.000102229	0.007318455	0.006585065	1551	tags=15%, list=7%, signal=14%
GO:0042119	BP	GO:0042119	neutrophil activation	442	0.566826497	1.892359774	0.00010227	0.007318455	0.006585065	1551	tags=15%, list=7%, signal=15%
GO:0010817	BP	GO:0010817	regulation of hormone levels	429	0.469522799	1.567046806	0.000102407	0.007318455	0.006585065	3771	tags=28%, list=17%, signal=24%
GO:0043312	BP	GO:0043312	neutrophil degranulation	434	0.560895261	1.871659111	0.000102449	0.007318455	0.006585065	1551	tags=15%, list=7%, signal=14%
GO:0002283	BP	GO:0002283	neutrophil activation involved in immune response	435	0.560145733	1.868893851	0.00010248	0.007318455	0.006585065	1551	tags=15%, list=7%, signal=14%
GO:0050878	BP	GO:0050878	regulation of body fluid levels	435	0.562914381	1.878131285	0.00010248	0.007318455	0.006585065	4092	tags=35%, list=19%, signal=29%
GO:0032103	BP	GO:0032103	positive regulation of response to external stimulus	417	0.502488862	1.674826221	0.00010268	0.007318455	0.006585065	2069	tags=16%, list=10%, signal=15%
GO:0030545	MF	GO:0030545	receptor regulator activity	396	0.582615043	1.938828913	0.000102934	0.007318455	0.006585065	2349	tags=29%, list=11%, signal=27%
GO:0001558	BP	GO:0001558	regulation of cell growth	374	0.479791371	1.592163635	0.000103477	0.007318455	0.006585065	3736	tags=21%, list=17%, signal=18%
GO:0052547	BP	GO:0052547	regulation of peptidase activity	374	0.526744076	1.747973835	0.000103477	0.007318455	0.006585065	2424	tags=19%, list=11%, signal=17%
GO:0001228	MF	GO:0001228	DNA-binding transcription activator activity, RNA polymerase II-specific	367	0.499278687	1.655569349	0.000103498	0.007318455	0.006585065	3177	tags=23%, list=15%, signal=20%
GO:0001216	MF	GO:0001216	DNA-binding transcription activator activity	371	0.499026951	1.655611074	0.000103509	0.007318455	0.006585065	3177	tags=23%, list=15%, signal=20%
GO:0051346	BP	GO:0051346	negative regulation of hydrolase activity	375	0.524564008	1.74050445	0.00010352	0.007318455	0.006585065	2370	tags=18%, list=11%, signal=17%
GO:0062023	CC	GO:0062023	collagen-containing extracellular matrix	368	0.558143768	1.850882414	0.000103552	0.007318455	0.006585065	3999	tags=42%, list=18%, signal=35%
GO:0050900	BP	GO:0050900	leukocyte migration	364	0.506462084	1.678524066	0.000103563	0.007318455	0.006585065	3577	tags=30%, list=17%, signal=26%
GO:0030546	MF	GO:0030546	signaling receptor activator activity	363	0.598046432	1.982045111	0.000103605	0.007318455	0.006585065	2742	tags=34%, list=13%, signal=30%
GO:0043062	BP	GO:0043062	extracellular structure organization	356	0.53168611	1.760978209	0.000103767	0.007318455	0.006585065	3887	tags=39%, list=18%, signal=33%
GO:0030198	BP	GO:0030198	extracellular matrix organization	355	0.530897829	1.758377208	0.000103788	0.007318455	0.006585065	3887	tags=39%, list=18%, signal=32%
GO:0045229	BP	GO:0045229	external encapsulating structure organization	357	0.529047539	1.751998747	0.00010381	0.007318455	0.006585065	3887	tags=39%, list=18%, signal=32%
GO:0048018	MF	GO:0048018	receptor ligand activity	357	0.600415357	1.988341077	0.00010381	0.007318455	0.006585065	2742	tags=34%, list=13%, signal=30%
GO:0001819	BP	GO:0001819	positive regulation of cytokine production	354	0.476769663	1.578681991	0.000103821	0.007318455	0.006585065	2613	tags=22%, list=12%, signal=19%
GO:0050673	BP	GO:0050673	epithelial cell proliferation	354	0.491567444	1.627680477	0.000103821	0.007318455	0.006585065	3133	tags=25%, list=14%, signal=22%
GO:0001503	BP	GO:0001503	ossification	353	0.473363766	1.567094289	0.000103864	0.007318455	0.006585065	4163	tags=32%, list=19%, signal=26%
GO:0061458	BP	GO:0061458	reproductive system development	353	0.476381155	1.577083506	0.000103864	0.007318455	0.006585065	3133	tags=22%, list=14%, signal=19%
GO:0030099	BP	GO:0030099	myeloid cell differentiation	350	0.476208628	1.575629374	0.000103972	0.007318455	0.006585065	2580	tags=19%, list=12%, signal=17%
GO:0048608	BP	GO:0048608	reproductive structure development	350	0.477977472	1.58148194	0.000103972	0.007318455	0.006585065	3133	tags=22%, list=14%, signal=19%
GO:0052548	BP	GO:0052548	regulation of endopeptidase activity	347	0.523021124	1.730366547	0.000103993	0.007318455	0.006585065	2424	tags=18%, list=11%, signal=17%
GO:0048568	BP	GO:0048568	embryonic organ development	340	0.492622779	1.628312575	0.000104178	0.007318455	0.006585065	4119	tags=31%, list=19%, signal=26%
GO:0018212	BP	GO:0018212	peptidyl-tyrosine modification	327	0.483287854	1.595525935	0.000104428	0.007318455	0.006585065	3302	tags=23%, list=15%, signal=20%
GO:0018108	BP	GO:0018108	peptidyl-tyrosine phosphorylation	324	0.48208965	1.590573369	0.000104559	0.007318455	0.006585065	3302	tags=23%, list=15%, signal=20%
GO:0042493	BP	GO:0042493	response to drug	320	0.492574581	1.624345657	0.00010469	0.007318455	0.006585065	3175	tags=22%, list=15%, signal=19%
GO:0002250	BP	GO:0002250	adaptive immune response	318	0.499840074	1.64826817	0.000104745	0.007318455	0.006585065	2374	tags=22%, list=11%, signal=20%
GO:0051098	BP	GO:0051098	regulation of binding	318	0.468290212	1.544229627	0.000104745	0.007318455	0.006585065	3037	tags=17%, list=14%, signal=15%
GO:0009615	BP	GO:0009615	response to virus	314	0.547319319	1.803785825	0.000104833	0.007318455	0.006585065	2346	tags=22%, list=11%, signal=20%
GO:0004857	MF	GO:0004857	enzyme inhibitor activity	308	0.544028786	1.792575385	0.000104866	0.007318455	0.006585065	2401	tags=21%, list=11%, signal=19%
GO:0019058	BP	GO:0019058	viral life cycle	310	0.473344132	1.559575004	0.000104899	0.007318455	0.006585065	2980	tags=19%, list=14%, signal=16%
GO:2001233	BP	GO:2001233	regulation of apoptotic signaling pathway	310	0.481560626	1.586646721	0.000104899	0.007318455	0.006585065	4792	tags=30%, list=22%, signal=24%
GO:0050678	BP	GO:0050678	regulation of epithelial cell proliferation	306	0.49363971	1.626259372	0.000104954	0.007318455	0.006585065	3123	tags=25%, list=14%, signal=22%
GO:0048545	BP	GO:0048545	response to steroid hormone	299	0.520057635	1.710381585	0.000105285	0.007318455	0.006585065	2940	tags=22%, list=14%, signal=19%
GO:0071214	BP	GO:0071214	cellular response to abiotic stimulus	299	0.527525574	1.734942374	0.000105285	0.007318455	0.006585065	2947	tags=21%, list=14%, signal=18%
GO:0104004	BP	GO:0104004	cellular response to environmental stimulus	299	0.527525574	1.734942374	0.000105285	0.007318455	0.006585065	2947	tags=21%, list=14%, signal=18%
GO:0007599	BP	GO:0007599	hemostasis	297	0.55119248	1.81244854	0.00010533	0.007318455	0.006585065	4092	tags=36%, list=19%, signal=30%
GO:0001655	BP	GO:0001655	urogenital system development	292	0.482353297	1.585146616	0.000105374	0.007318455	0.006585065	4014	tags=33%, list=19%, signal=27%
GO:0050817	BP	GO:0050817	coagulation	295	0.558996783	1.837384963	0.000105385	0.007318455	0.006585065	4092	tags=37%, list=19%, signal=30%
GO:0007596	BP	GO:0007596	blood coagulation	293	0.559992241	1.840371918	0.000105407	0.007318455	0.006585065	4092	tags=37%, list=19%, signal=30%
GO:0018105	BP	GO:0018105	peptidyl-serine phosphorylation	294	0.479156842	1.574999346	0.000105419	0.007318455	0.006585065	2325	tags=12%, list=11%, signal=11%
GO:0031349	BP	GO:0031349	positive regulation of defense response	294	0.556919351	1.830606464	0.000105419	0.007318455	0.006585065	1711	tags=16%, list=8%, signal=15%
GO:0031983	CC	GO:0031983	vesicle lumen	284	0.582244167	1.909119287	0.000105787	0.007318455	0.006585065	3417	tags=24%, list=16%, signal=20%

GO:0060205	CC	GO:0060205 cytoplasmic vesicle lumen	282	0.585487978	1.919165254	0.000105921	0.007318455	0.006585065	3417	tags=24%, list=16%, signal=21%
GO:0071496	BP	GO:0071496 cellular response to external stimulus	278	0.495799356	1.623677284	0.00010609	0.007318455	0.006585065	4426	tags=29%, list=20%, signal=23%
GO:0005788	CC	GO:0005788 endoplasmic reticulum lumen	276	0.514973755	1.686049706	0.000106101	0.007318455	0.006585065	3908	tags=33%, list=18%, signal=27%
GO:0002237	BP	GO:0002237 response to molecule of bacterial origin	279	0.579654376	1.89803477	0.000106202	0.007318455	0.006585065	2618	tags=25%, list=12%, signal=23%
GO:0034774	CC	GO:0034774 secretory granule lumen	279	0.583862073	1.911812558	0.000106202	0.007318455	0.006585065	1551	tags=14%, list=7%, signal=13%
GO:0045861	BP	GO:0045861 negative regulation of proteolysis	274	0.55550083	1.818447062	0.000106202	0.007318455	0.006585065	2370	tags=20%, list=11%, signal=18%
GO:0050727	BP	GO:0050727 regulation of inflammatory response	274	0.599801853	1.963467665	0.000106202	0.007318455	0.006585065	2039	tags=22%, list=9%, signal=20%
GO:0030667	CC	GO:0030667 secretory granule membrane	270	0.517968538	1.693260204	0.000106576	0.007318455	0.006585065	3766	tags=27%, list=17%, signal=23%
GO:0032496	BP	GO:0032496 response to lipopolysaccharide	266	0.593208253	1.938497902	0.000106644	0.007318455	0.006585065	2618	tags=26%, list=12%, signal=23%
GO:0034612	BP	GO:0034612 response to tumor necrosis factor	267	0.55653409	1.818301314	0.000106644	0.007318455	0.006585065	2498	tags=21%, list=12%, signal=19%
GO:0008544	BP	GO:0008544 epidermis development	268	0.497257373	1.624651371	0.000106689	0.007318455	0.006585065	3364	tags=26%, list=16%, signal=22%
GO:0007568	BP	GO:0007568 aging	259	0.504696658	1.646052119	0.000106952	0.007318455	0.006585065	3108	tags=21%, list=14%, signal=18%
GO:1901342	BP	GO:1901342 regulation of vasculature development	251	0.546987206	1.780746254	0.000107193	0.007318455	0.006585065	3303	tags=30%, list=15%, signal=26%
GO:0071356	BP	GO:0071356 cellular response to tumor necrosis factor	246	0.536449126	1.744685945	0.000107308	0.007318455	0.006585065	2498	tags=21%, list=12%, signal=19%
GO:0045765	BP	GO:0045765 regulation of angiogenesis	247	0.549311756	1.786121942	0.000107377	0.007318455	0.006585065	3303	tags=30%, list=15%, signal=26%
GO:0072593	BP	GO:0072593 reactive oxygen species metabolic process	245	0.531478533	1.727739519	0.0001074	0.007318455	0.006585065	2439	tags=21%, list=11%, signal=19%
GO:0051607	BP	GO:0051607 defense response to virus	229	0.572378571	1.85427029	0.000108085	0.007318455	0.006585065	2323	tags=24%, list=11%, signal=22%
GO:0140546	BP	GO:0140546 defense response to symbiont	229	0.572378571	1.85427029	0.000108085	0.007318455	0.006585065	2323	tags=24%, list=11%, signal=22%
GO:0043588	BP	GO:0043588 skin development	231	0.522014432	1.691485288	0.000108131	0.007318455	0.006585065	3364	tags=29%, list=16%, signal=24%
GO:0050730	BP	GO:0050730 regulation of peptidyl-tyrosine phosphorylation	223	0.525410888	1.69762991	0.000108672	0.007318455	0.006585065	3270	tags=26%, list=15%, signal=22%
GO:0097305	BP	GO:0097305 response to alcohol	209	0.54740154	1.761607036	0.000109349	0.007318455	0.006585065	2279	tags=17%, list=11%, signal=15%
GO:0009636	BP	GO:0009636 response to toxic substance	211	0.545245584	1.754754233	0.000109373	0.007318455	0.006585065	3114	tags=28%, list=14%, signal=25%
GO:0002460	BP	GO:0002460 adaptive immune response based on somatic recombination of immune receptors built from immunoglobulin superfamily domains	210	0.553523098	1.781265471	0.000109397	0.007318455	0.006585065	2608	tags=23%, list=12%, signal=20%
GO:0061448	BP	GO:0061448 connective tissue development	205	0.551709484	1.772254048	0.000109733	0.007318455	0.006585065	2947	tags=31%, list=14%, signal=27%
GO:0000302	BP	GO:0000302 response to reactive oxygen species	197	0.550353022	1.763560587	0.00010983	0.007318455	0.006585065	2935	tags=21%, list=14%, signal=19%
GO:0005126	MF	GO:0005126 cytokine receptor binding	197	0.640388328	2.052071252	0.00010983	0.007318455	0.006585065	2475	tags=27%, list=11%, signal=24%
GO:0010466	BP	GO:0010466 negative regulation of peptidase activity	195	0.60133409	1.924702359	0.000110132	0.007318455	0.006585065	2370	tags=26%, list=11%, signal=23%
GO:0035966	BP	GO:0035966 response to topologically incorrect protein	194	0.568405912	1.81891383	0.000110144	0.007318455	0.006585065	4690	tags=28%, list=22%, signal=22%
GO:0005539	MF	GO:0005539 glycosaminoglycan binding	186	0.55036687	1.757747153	0.000110387	0.007318455	0.006585065	4063	tags=40%, list=19%, signal=33%
GO:0071216	BP	GO:0071216 cellular response to biotic stimulus	186	0.558173278	1.782679052	0.000110387	0.007318455	0.006585065	2370	tags=21%, list=11%, signal=19%
GO:0002064	BP	GO:0002064 epithelial cell development	189	0.552950534	1.766458037	0.000110461	0.007318455	0.006585065	3442	tags=29%, list=16%, signal=24%
GO:0010951	BP	GO:0010951 negative regulation of endopeptidase activity	184	0.601341851	1.918462718	0.000110632	0.007318455	0.006585065	2370	tags=25%, list=11%, signal=22%
GO:0044706	BP	GO:0044706 multi-multicellular organism process	181	0.577089311	1.838873651	0.000110889	0.007318455	0.006585065	1324	tags=18%, list=6%, signal=17%
GO:1901654	BP	GO:1901654 response to ketone	178	0.564603486	1.7969288	0.000111222	0.007318455	0.006585065	3590	tags=29%, list=17%, signal=25%
GO:0006986	BP	GO:0006986 response to unfolded protein	174	0.578520292	1.838640788	0.000111421	0.007318455	0.006585065	1276	tags=10%, list=6%, signal=10%
GO:0030595	BP	GO:0030595 leukocyte chemotaxis	174	0.544894552	1.731772181	0.000111421	0.007318455	0.006585065	540	tags=11%, list=2%, signal=11%
GO:0002573	BP	GO:0002573 myeloid leukocyte differentiation	176	0.544278539	1.730255159	0.000111445	0.007318455	0.006585065	2578	tags=23%, list=12%, signal=20%
GO:0070555	BP	GO:0070555 response to interleukin-1	169	0.603819786	1.914728976	0.000111732	0.007318455	0.006585065	2475	tags=18%, list=11%, signal=16%
GO:0101002	CC	GO:0101002 ficolin-1-rich granule	171	0.553390719	1.754816099	0.000111832	0.007318455	0.006585065	1548	tags=13%, list=7%, signal=12%
GO:0006959	BP	GO:0006959 humoral immune response	170	0.63267847	2.005718689	0.000111857	0.007318455	0.006585065	2225	tags=35%, list=10%, signal=31%
GO:0071478	BP	GO:0071478 cellular response to radiation	168	0.552814613	1.752111044	0.000111857	0.007318455	0.006585065	2879	tags=20%, list=13%, signal=17%
GO:2000377	BP	GO:2000377 regulation of reactive oxygen species metabolic process	168	0.549215822	1.740704905	0.000111857	0.007318455	0.006585065	2281	tags=18%, list=11%, signal=17%
GO:0050731	BP	GO:0050731 positive regulation of peptidyl-tyrosine phosphorylation	167	0.563623862	1.784991603	0.000112095	0.007318455	0.006585065	2160	tags=24%, list=10%, signal=22%
GO:0005125	MF	GO:0005125 cytokine activity	166	0.709896767	2.246166342	0.000112296	0.007318455	0.006585065	2719	tags=43%, list=13%, signal=38%
GO:0097529	BP	GO:0097529 myeloid leukocyte migration	166	0.565141979	1.78815139	0.000112296	0.007318455	0.006585065	2864	tags=26%, list=13%, signal=23%
GO:0071219	BP	GO:0071219 cellular response to molecule of bacterial origin	162	0.591385016	1.868263451	0.000112385	0.007318455	0.006585065	2370	tags=23%, list=11%, signal=20%
GO:0006022	BP	GO:0006022 aminoglycan metabolic process	163	0.577544488	1.824416298	0.000112486	0.007318455	0.006585065	3887	tags=31%, list=18%, signal=26%
GO:0007565	BP	GO:0007565 female pregnancy	159	0.58565362	1.848646378	0.000112778	0.007318455	0.006585065	1324	tags=18%, list=6%, signal=17%
GO:0035967	BP	GO:0035967 cellular response to topologically incorrect protein	157	0.555306679	1.750851508	0.000113007	0.007318455	0.006585065	3873	tags=22%, list=18%, signal=18%
GO:0007369	BP	GO:0007369 gastrulation	155	0.577402801	1.819269384	0.000113122	0.007318455	0.006585065	4268	tags=38%, list=20%, signal=31%
GO:0071222	BP	GO:0071222 cellular response to lipopolysaccharide	155	0.60664999	1.911420852	0.000113122	0.007318455	0.006585065	2370	tags=23%, list=11%, signal=21%
GO:0051216	BP	GO:0051216 cartilage development	154	0.541389471	1.703864194	0.000113327	0.007318455	0.006585065	2947	tags=31%, list=14%, signal=27%
GO:0071347	BP	GO:0071347 cellular response to interleukin-1	148	0.597794877	1.874747991	0.000113947	0.007318455	0.006585065	2475	tags=19%, list=11%, signal=17%
GO:0097696	BP	GO:0097696 receptor signaling pathway via STAT	148	0.679411038	2.130704906	0.000113947	0.007318455	0.006585065	2323	tags=31%, list=11%, signal=28%
GO:0070820	CC	GO:0070820 tertiary granule	145	0.572046036	1.78915824	0.000114495	0.007318455	0.006585065	1548	tags=19%, list=7%, signal=18%
GO:0007259	BP	GO:0007259 receptor signaling pathway via JAK-STAT	141	0.684336939	2.137580952	0.000114718	0.007318455	0.006585065	2323	tags=32%, list=11%, signal=29%
GO:0045766	BP	GO:0045766 positive regulation of angiogenesis	141	0.557840264	1.74245851	0.000114718	0.007318455	0.006585065	3678	tags=35%, list=17%, signal=29%
GO:1904018	BP	GO:1904018 positive regulation of vasculature development	141	0.557840264	1.74245851	0.000114718	0.007318455	0.006585065	3678	tags=35%, list=17%, signal=29%
GO:0061135	MF	GO:0061135 endopeptidase regulator activity	143	0.557851954	1.74283134	0.000114745	0.007318455	0.006585065	2370	tags=26%, list=11%, signal=23%
GO:0034620	BP	GO:0034620 cellular response to unfolded protein	139	0.569651512	1.777322866	0.000115048	0.007318455	0.006585065	3705	tags=22%, list=17%, signal=18%
GO:0002819	BP	GO:0002819 regulation of adaptive immune response	138	0.582473198	1.815765398	0.000115128	0.007318455	0.006585065	2350	tags=21%, list=11%, signal=19%
GO:0030414	MF	GO:0030414 peptidase inhibitor activity	137	0.569741742	1.774381401	0.000115128	0.007318455	0.006585065	2370	tags=28%, list=11%, signal=26%
GO:0042581	CC	GO:0042581 specific granule	137	0.567847965	1.768483497	0.000115128	0.007318455	0.006585065	1992	tags=19%, list=9%, signal=17%
GO:0031960	BP	GO:0031960 response to corticosteroid	134	0.576535042	1.792528377	0.000115367	0.007318455	0.006585065	2618	tags=24%, list=12%, signal=21%
GO:0008083	MF	GO:0008083 growth factor activity	132	0.629094563	1.954046838	0.00011554	0.007318455	0.006585065	2337	tags=29%, list=11%, signal=26%
GO:0033135	BP	GO:0033135 regulation of peptidyl-serine phosphorylation	132	0.671342918	2.085275542	0.00011554	0.007318455	0.006585065	2146	tags=20%, list=10%, signal=18%
GO:0004866	MF	GO:0004866 endopeptidase inhibitor activity	131	0.568856801	1.766287688	0.000115554	0.007318455	0.006585065	2370	tags=28%, list=11%, signal=25%
GO:0030856	BP	GO:0030856 regulation of epithelial cell differentiation	127	0.662111512	2.047696349	0.000116171	0.007318455	0.006585065	3118	tags=39%, list=14%, signal=34%
GO:0002822	BP	GO:0002822 regulation of adaptive immune response based on somatic recombination of immune receptors built from immunoglobulin superfamily domains	125	0.58755124	1.815150833	0.000116266	0.007318455	0.006585065	2350	tags=21%, list=11%, signal=19%
GO:0097530	BP	GO:0097530 granulocyte migration	110	0.630519343	1.920767292	0.000118638	0.007318455	0.006585065	2619	tags=29%, list=12%, signal=26%
GO:0050729	BP	GO:0050729 positive regulation of inflammatory response	101	0.689869735	2.081999489	0.00012038	0.007318455	0.006585065	2205	tags=31%, list=10%, signal=28%
GO:0033138	BP	GO:0033138 positive regulation of peptidyl-serine phosphorylation	98	0.708422303	2.128490437	0.000121256	0.007318455	0.006585065	2146	tags=24%, list=10%, signal=22%
GO:0034340	BP	GO:0034340 response to type I interferon	92	0.64116489	1.912257403	0.000122234	0.007318455	0.006585065	2323	tags=36%, list=11%, signal=32%
GO:0072562	CC	GO:0072562 blood microparticle	92	0.630869199	1.881550777	0.000122234	0.007318455	0.006585065	3449	tags=46%, list=16%, signal=39%
GO:1904892	BP	GO:1904892 regulation of receptor signaling pathway via STAT	92	0.698970172	2.084660136	0.000122234	0.007318455	0.006585065	2312	tags=33%, list=11%, signal=29%
GO:0071357	BP	GO:0071357 cellular response to type I interferon	88	0.649967814	1.931480626	0.000122264	0.007318455	0.006585065	2323	tags=38%, list=11%, signal=34%
GO:0071621	BP	GO:0071621 granulocyte chemotaxis	93	0.635966466	1.899174308	0.000122264	0.007318455	0.006585065	785	tags=17%, list=4%, signal=17%
GO:2000379	BP	GO:2000379 positive regulation of reactive oxygen species metabolic process	89	0.672528259	1.9997424	0.000122309	0.007318455	0.006585065	715	tags=20%, list=3%, signal=20%
GO:0060337	BP	GO:0060337 type I interferon signaling pathway	87	0.650207738	1.928392739	0.000122549	0.007318455	0.006585065	2323	tags=38%, list=11%, signal=34%
GO:1990266	BP	GO:1990266 neutrophil migration	87	0.647473779	1.920284335	0.000122549	0.007318455	0.006585065	2337	tags=29%, list=11%, signal=26%
GO:0046425	BP	GO:0046425 regulation of receptor signaling pathway via JAK-STAT	86	0.704354139	2.086962967	0.000122714	0.007318455	0.006585065	2312	tags=34%, list=11%, signal=30%
GO:0034644	BP	GO:0034644 cellular response to UV	83	0.644860056	1.900233945	0.000123732	0.007318455	0.006585065	1874	tags=14%, list=9%, signal=13%
GO:0002526	BP	GO:0002526 acute inflammatory response	82	0.675118698	1.985473653	0.000124394	0.007318455	0.006585065	2210	tags=30%, list=10%, signal=27%
GO:0031091	CC	GO:0031091 platelet alpha granule	78	0.677412296	1.983777514	0.000124626	0.007318455	0.006585065	3469	tags=38%, list=16%, signal=32%
GO:1900182	BP	GO:1900182 positive regulation of protein localization to nucleus	81	0.64093598	1.882778876	0.000124766	0.007318455	0.006585065	1937	tags=19%, list=9%, signal=17%
GO:0030593	BP	GO:0030593 neutrophil chemotaxis	73	0.663657646	1.926684493	0.000125755	0.007318455	0.006585065	540	tags=18%, list=2%, signal=17%
GO:0019730	BP	GO:0019730 antimicrobial humoral response	69	0.685624019	1.972795915	0.000127405	0.007318455	0.006585065	1005	tags=29%, list=5%, signal=28%
GO:0007260	BP	GO:0007260 tyrosine phosphorylation of STAT protein	66	0.705696228	2.018299596	0.000128551	0.007318455	0.006585065	2146	tags=30%, list=10%, signal=27%
GO:0048306	MF	GO:0048306 calcium-dependent protein binding	66	0.686778717	1.96419529	0.000128551	0.007318455	0.006585065	1256	tags=21%, list=6%, signal=20%
GO:0045685	BP	GO:0045685 regulation of glial cell differentiation	65	0.667368936	1.904051976	0.000128833	0.007318455	0.006585065	1117	tags=15%, list=5%, signal=15%
GO:0048708	BP	GO:0048708 astrocyte differentiation	65	0.72871235	2.079069187	0.000128833	0.007318455	0.006585065	1156	tags=17%, list=5%, signal=16%
GO:0043154	BP	GO:0043154 negative regulation of cysteine-type endopeptidase activity involved in apoptotic process	64	0.666545002	1.897763065	0.000129082	0.007318455	0.006585065	1743	tags=16%, list=8%, signal=14%
GO:0042509	BP	GO:0042509 regulation of tyrosine phosphorylation of STAT protein	63	0.721691396	2.051977212	0.000129182	0.007318455	0.006585065	2146	tags=32%, list=10%, signal=29%
GO:0014015	BP	GO:0014015 positive regulation of gliogenesis	59	0.690966287	1.941106225	0.000131062	0.007318455	0.006585065	1097	tags=14%, list=5%, signal=13%
GO:0046824	BP	GO:0046824 positive regulation of nucleocytoplasmic transport	59	0.703982556	1.977672352	0.000131062	0.007318455	0.006585065	1282	tags=14%, list=6%, signal=13%
GO:0061077	BP	GO:0061077 chaperone-mediated protein folding	59	0.682112569	1.9162338	0.000131062	0.007318455	0.006585065	1276	tags=14%, list=6%, signal=13%
GO:0072413	BP	GO:0072413 signal transduction involved in mitotic cell cycle checkpoint	59	0.682551446	1.917466721	0.000131062	0.007318455	0.006585065	913	tags=12%, list=4%, signal=11%
GO:1902402	BP	GO:1902402 signal transduction involved in mitotic DNA damage checkpoint	57	0.688862282	1.924412276	0.000131909	0.007318455	0.006585065	913	tags=12%, list=4%, signal=12%
GO:1902403	BP	GO:1902403 signal transduction involved in mitotic DNA integrity checkpoint	57	0.688862282	1.924412276	0.000131909	0.007318455	0.006585065	913	tags=12%, list=4%, signal=12%
GO:0072431	BP	GO:0072431 signal transduction involved in mitotic G1 DNA damage checkpoint	56	0.691603993	1.927305508	0.000132398	0.007318455	0.006585065	913	tags=12%, list=4%, signal=12%
GO:1902400	BP	GO:1902400 intracellular signal transduction involved in G1 DNA damage checkpoint	56	0.691603993	1.927305508	0.000132398	0.007318455	0.006585065	913	tags=12%, list=4%, signal=12%
GO:0006977	BP	GO:0006977 DNA damage response, signal transduction by p53 class mediator resulting in cell cycle arrest	55	0.693932222	1.927765101	0.000132767	0.007318455	0.006585065	913	tags=13%, list=4%, signal=12%
GO:0042531	BP	GO:0042531 positive regulation of tyrosine phosphorylation of STAT protein	53	0.732570378	2.026795483	0.00013328	0.007318701	0.006585286	2146	tags=36%, list=10%, signal=32%
GO:0035580	CC	GO:0035580 specific granule lumen	50	0.702963765	1.930788671	0.000133994	0.007329951	0.006595408	1344	tags=24%, list=6%, signal=23%
GO:0030858	BP	GO:0030858 positive regulation of epithelial cell differentiation	47	0.729782716	1.982614019	0.000135704	0.007395333	0.006654239	3118	tags=38%, list=14%, signal=33%
GO:0002548	BP	GO:0002548 monocyte chemotaxis	44	0.704619741	1.894020817	0.000136874	0.007430956	0.006686292	409	tags=20%, list=2%, signal=20%
GO:0032570	BP	GO:0032570 response to progesterone	42	0.752864366	2.006782428	0.000138293	0.007431179	0.006686492	1584	tags=33%, list=7%, signal=31%
GO:0007566	BP	GO:0007566 embryo implantation	41	0.795176959	2.113104456	0.000138427	0.007431179	0.006686492	1023	tags=27%, list=5%, signal=26%
GO:0045687	BP	GO:0045687 positive regulation of glial cell differentiation	41	0.714721096	1.899300923	0.000138427	0.007431179	0.006686492	1097	tags=15%, list=5%, signal=14%
GO:0046825	BP	GO:0046825 regulation of protein export from nucleus	30	0.802160296	2.023629097	0.000143699	0.007685482	0.006915312	840	tags=13%, list=4%, signal=13%
GO:0051385	BP	GO:0051385 response to mineralocorticoid	28	0.82821571	2.061008257	0.000146113	0.007785679	0.007005467	264	tags=25%, list=1%, signal=25%
GO:0042501	BP	GO:0042501 serine phosphorylation of STAT protein	25	0.848167214	2.067445469	0.000149187	0.007920119	0.007126436	2146	tags=64%, list=10%, signal=58%
GO:0048710	BP	GO:0048710 regulation of astrocyte differentiation	22	0.837792403	1.99685761	0.000152184	0.008009678	0.00720702	1117	tags=27%, list=5%, signal=26%
GO:0002675	BP	GO:0002675 positive regulation of acute inflammatory response	21	0.828500413	1.955095131	0.000153586	0.008009678	0.00720702	354	tags=33%, list=2%, signal=33%
GO:0033139	BP	GO:0033139 regulation of peptidyl-serine phosphorylation of STAT protein	21	0.868899222	2.050428235	0.000153586	0.008009678	0.00720702	2146	tags=76%, list=10%, signal=69%
GO:0033141	BP	GO:0033141 positive regulation of peptidyl-serine phosphorylation of STAT protein	19	0.866544908	2.005666334	0.000155328	0.008009678	0.00720702	2146	tags=79%, list=10%, signal=71%
GO:0046827	BP	GO:0046827 positive regulation of protein export from nucleus	19	0.867980762	2.0089897	0.000155328	0.008009678	0.00720702	840	tags=21%, list=4%, signal=20%
GO:0051412	BP	GO:0051412 response to corticosterone	19	0.852946246	1.974191477	0.000155328	0.008009678	0.00720702	264	tags=26%, list=1%, signal=26%
GO:0051767	BP	GO:0051767 nitric-oxide synthase biosynthetic process	19	0.870026768	2.013725293	0.000155328	0.008009678	0.00720702	449	tags=26%, list=2%, signal=26%
GO:0051769	BP	GO:0051769 regulation of nitric-oxide synthase biosynthetic process	19	0.870026768	2.013725293	0.000155328	0.008009678	0.00720702	449	tags=26%, list=2%, signal=26%
GO:0005132	MF	GO:0005132 type I interferon receptor binding	16	0.860832986	1.928462027	0.000158504	0.008144261	0.007328116	2146	tags=88%, list=10%, signal=79%
GO:0051770	BP	GO:0051770 positive regulation of nitric-oxide synthase biosynthetic process	15	0.876800963	1.938182613	0.000159084	0.008144971	0.007328755	449	tags=27%, list=2%, signal=26%
GO:0070141	BP	GO:0070141 response to UV-A	13	0.863426395	1.856110914	0.000162628	0.008267597	0.007439092	94	tags=31%, list=0%, signal=31%
GO:1903025	BP	GO:1903025 regulation of RNA polymerase II regulatory region sequence-specific DNA binding	13	0.865998197	1.861639528	0.000162628	0.008267597	0.007439092	3	tags=8%, list=0%, signal=8%
GO:0046348	BP	GO:0046348 amino sugar catabolic process	12	0.926331642	1.961034394	0.00016415	0.00831557	0.007482257	16	tags=17%, list=0%, signal=17%
GO:0045236	MF	GO:0045236 CXCR chemokine receptor binding	10	0.952922722	1.942892181	0.00016787	0.008474182	0.007624975	292	tags=60%, list=1%, signal=59%
GO:0050786	MF	GO:0050786 RAGE receptor binding	9	0.934195382	1.855909153	0.000171527	0.008628507	0.007763835	949	tags=56%, list=4%, signal=53%
GO:1901072	BP	GO:1901072 glucosamine-containing compound catabolic process	8	0.975026416	1.897919648	0.000172325	0.008638449	0.007772781	16	tags=25%, list=0%, signal=25%
GO:0008061	MF	GO:0008061 chitin binding	7	0.94496036	1.787360332	0.000174064	0.008665275	0.007796919	16	tags=29%, list=0%, signal=29%
GO:0050543	MF	GO:0050543 icosatetraenoic acid binding	7	0.937361205	1.77298679	0.000174064	0.008665275	0.007796919	43	tags=29%, list=0%, signal=29%
GO:0006030	BP	GO:0006030 chitin metabolic process	5	0.977884619	1.71571816	0.000182548	0.009025167	0.008120745	16	tags=40%, list=0%, signal=40%
GO:0006032	BP	GO:0006032 chitin catabolic process	5	0.977884619	1.71571816	0.000182548	0.009025167	0.008120745	16	tags=40%, list=0%, signal=40%
GO:0035662	MF	GO:0035662 Toll-like receptor 4 binding	4	0.98969555	1.647200617	0.000187899	0.009257901	0.008330157	43	tags=50%, list=0%, signal=50%
GO:0055080	BP	GO:0055080 cation homeostasis	656	0.413488663	1.393917159	0.000201349	0.009772509	0.008793195	2626	tags=16%, list=12%, signal=14%
GO:0097435	BP	GO:0097435 supramolecular fiber organization	651	0.409730549	1.381003623	0.00020141	0.009772509	0.008793195	4218	tags=24%, list=20%, signal=20%
GO:0006935	BP	GO:0006935 chemotaxis	545	0.428135726	1.437489012	0.000202429	0.009772509	0.008793195	3516	tags=22%, list=16%, signal=19%
GO:0061061	BP	GO:0061061 muscle structure development	527	0.436279142	1.463798768	0.000202655	0.009772509	0.008793195	3271	tags=20%, list=15%, signal=18%
GO:0050839	MF	GO:0050839 cell adhesion molecule binding	495	0.429813589	1.439804463	0.000203128	0.009772509	0.008793195	4093	tags=25%, list=19%, signal=21%
GO:0004930	MF	GO:0004930 G protein-coupled receptor activity	484	0.434907503	1.456128409	0.000203314	0.009772509	0.008793195	2643	tags=30%, list=12%, signal=27%
GO:1901652	BP	GO:1901652 response to peptide	464	0.441243303	1.475020611	0.000203957	0.009772509	0.008793195	3970	tags=24%, list=18%, signal=20%
GO:0043410	BP	GO:0043410 positive regulation of MAPK cascade	449	0.460087099	1.536487982	0.000204269	0.009772509	0.008793195	3149	tags=20%, list=15%, signal=18%
GO:0071900	BP	GO:0071900 regulation of protein serine/threonine kinase activity	446	0.440095361	1.469307968	0.000204457	0.009772509	0.008793195	2325	tags=14%, list=11%, signal=13%
GO:0051090	BP	GO:0051090 regulation of DNA-binding transcription factor activity	391	0.457801953	1.522085709	0.000206228	0.009824515	0.00883999	2608	tags=15%, list=12%, signal=14%
GO:1903131	BP	GO:1903131 mononuclear cell differentiation	353	0.456478621	1.511195176	0.000207727	0.009852328	0.008865016	2348	tags=21%, list=11%, signal=19%
GO:0070482	BP	GO:0070482 response to oxygen levels	345	0.457825265	1.514331955	0.000208182	0.009852328	0.008865016	3925	tags=22%, list=18%, signal=18%
GO:0018209	BP	GO:0018209 peptidyl-serine modification	315	0.457720255	1.508394658	0.00020982	0.009897292	0.008905473	2325	tags=12%, list=11%, signal=11%
GO:0007517	BP	GO:0007517 muscle organ development	273	0.482738969	1.579787301	0.000212585	0.00999497	0.008993363	3251	tags=24%, list=15%, signal=21%
GO:0072001	BP	GO:0072001 renal system development	265	0.494658764	1.615978971	0.000213311	0.009996415	0.008994663	4014	tags=33%, list=19%, signal=27%
GO:0001664	MF	GO:0001664 G protein-coupled receptor binding	229	0.509123225	1.649349081	0.000216169	0.010097501	0.009085619	3005	tags=24%, list=14%, signal=21%
GO:0045444	BP	GO:0045444 fat cell differentiation	192	0.533313944	1.705090542	0.000220824	0.010281522	0.009251199	3730	tags=29%, list=17%, signal=24%
GO:0061134	MF	GO:0061134 peptidase regulator activity	179	0.534065246	1.700559085	0.000222099	0.010307535	0.009274606	2370	tags=25%, list=11%, signal=22%
GO:0005201	MF	GO:0005201 extracellular matrix structural constituent	148	0.544236537	1.706783368	0.000227894	0.010542491	0.009486016	3958	tags=45%, list=18%, signal=37%
GO:1903034	BP	GO:1903034 regulation of response to wounding	136	0.562348058	1.750137859	0.000230362	0.010606081	0.009543234	4275	tags=40%, list=20%, signal=33%
GO:0008201	MF	GO:0008201 heparin binding	130	0.576125901	1.787255202	0.000231428	0.010606081	0.009543234	3834	tags=40%, list=18%, signal=33%
GO:0055076	BP	GO:0055076 transition metal ion homeostasis	126	0.579396431	1.790253525	0.000232504	0.010606081	0.009543234	1263	tags=15%, list=6%, signal=14%
GO:0042770	BP	GO:0042770 signal transduction in response to DNA damage	123	0.587121904	1.809870419	0.000233781	0.010606081	0.009543234	2211	tags=15%, list=10%, signal=14%
GO:0042542	BP	GO:0042542 response to hydrogen peroxide	120	0.575558727	1.76874867	0.000234632	0.010606081	0.009543234	2935	tags=22%, list=14%, signal=20%
GO:0051384	BP	GO:0051384 response to glucocorticoid	120	0.59792792	1.837491402	0.000234632	0.010606081	0.009543234	2618	tags=25%, list=12%, signal=22%
GO:0001890	BP	GO:0001890 placenta development	119	0.584717968	1.79422159	0.000235377	0.010606081	0.009543234	2464	tags=23%, list=11%, signal=20%
GO:0071482	BP	GO:0071482 cellular response to light stimulus	115	0.585127444	1.789699139	0.00023649	0.010606081	0.009543234	2752	tags=19%, list=13%, signal=17%

GO:0002020	MF	GO:0002020	protease binding	111	0.595817045	1.816532366	0.000237164	0.010606081	0.009543234	2320	tags=25%, list=11%, signal=23%
GO:0019079	BP	GO:0019079	viral genome replication	109	0.596766943	1.815884622	0.000237897	0.010606081	0.009543234	2069	tags=19%, list=10%, signal=18%
GO:0061041	BP	GO:0061041	regulation of wound healing	106	0.57874091	1.754104887	0.000238977	0.010606081	0.009543234	4063	tags=42%, list=19%, signal=34%
GO:0046916	BP	GO:0046916	cellular transition metal ion homeostasis	105	0.592470658	1.794820727	0.00023932	0.010606081	0.009543234	1965	tags=18%, list=9%, signal=17%
GO:1902807	BP	GO:1902807	negative regulation of cell cycle G1/S phase transition	105	0.574232285	1.739569705	0.00023932	0.010606081	0.009543234	913	tags=10%, list=4%, signal=9%
GO:0045446	BP	GO:0045446	endothelial cell differentiation	104	0.582620178	1.762575089	0.000239837	0.010606081	0.009543234	3442	tags=36%, list=16%, signal=30%
GO:2000134	BP	GO:2000134	negative regulation of G1/S transition of mitotic cell cycle	103	0.585304183	1.768701603	0.000240327	0.010606081	0.009543234	2346	tags=14%, list=11%, signal=12%
GO:0001704	BP	GO:0001704	formation of primary germ layer	99	0.637244972	1.916706991	0.000241838	0.010607692	0.009544683	4268	tags=47%, list=20%, signal=38%
GO:0030330	BP	GO:0030330	DNA damage response, signal transduction by p53 class mediator	99	0.619531432	1.863428163	0.000241838	0.010607692	0.009544683	2211	tags=18%, list=10%, signal=16%
GO:0072395	BP	GO:0072395	signal transduction involved in cell cycle checkpoint	74	0.631231048	1.836490882	0.000251004	0.010943119	0.009846497	913	tags=9%, list=4%, signal=9%
GO:0072401	BP	GO:0072401	signal transduction involved in DNA integrity checkpoint	71	0.643465409	1.858853666	0.000253678	0.010943119	0.009846497	913	tags=10%, list=4%, signal=9%
GO:0072422	BP	GO:0072422	signal transduction involved in DNA damage checkpoint	71	0.643465409	1.858853666	0.000253678	0.010943119	0.009846497	913	tags=10%, list=4%, signal=9%
GO:0030104	BP	GO:0030104	water homeostasis	70	0.662859999	1.911401337	0.000254388	0.010943119	0.009846497	1210	tags=20%, list=6%, signal=19%
GO:1904035	BP	GO:1904035	regulation of epithelial cell apoptotic process	70	0.648352553	1.86956814	0.000254388	0.010943119	0.009846497	2673	tags=30%, list=12%, signal=26%
GO:2000117	BP	GO:2000117	negative regulation of cysteine-type endopeptidase activity	70	0.667261038	1.924092028	0.000254388	0.010943119	0.009846497	1341	tags=16%, list=6%, signal=15%
GO:0048525	BP	GO:0048525	negative regulation of viral process	69	0.668462922	1.923417041	0.00025481	0.010943119	0.009846497	2575	tags=35%, list=12%, signal=31%
GO:0007492	BP	GO:0007492	endoderm development	68	0.658439447	1.890613642	0.00025582	0.010953812	0.009856119	2385	tags=29%, list=11%, signal=26%
GO:0044783	BP	GO:0044783	G1 DNA damage checkpoint	62	0.665258124	1.886820958	0.000258598	0.011028654	0.00992346	913	tags=11%, list=4%, signal=11%
GO:0031571	BP	GO:0031571	mitotic G1 DNA damage checkpoint	61	0.666672529	1.885781963	0.000259101	0.011028654	0.00992346	913	tags=11%, list=4%, signal=11%
GO:0031093	CC	GO:0031093	platelet alpha granule lumen	56	0.676941451	1.886445133	0.000264795	0.011237794	0.010111642	3338	tags=39%, list=15%, signal=33%
GO:0046888	BP	GO:0046888	negative regulation of hormone secretion	54	0.679527386	1.884501813	0.000265745	0.011244939	0.010118072	1293	tags=20%, list=6%, signal=19%
GO:1903428	BP	GO:1903428	positive regulation of reactive oxygen species biosynthetic process	46	0.697915425	1.889344196	0.000271776	0.011466399	0.010317339	2281	tags=30%, list=11%, signal=27%
GO:0023023	MF	GO:0023023	MHC protein complex binding	18	-0.795666997	-2.107462604	0.000278164	0.0117016	0.01052897	2104	tags=44%, list=10%, signal=40%
GO:0006040	BP	GO:0006040	amino sugar metabolic process	37	0.736512853	1.923670847	0.000281096	0.01176158	0.010582939	1686	tags=16%, list=8%, signal=15%
GO:0014002	BP	GO:0014002	astrocyte development	36	0.751854231	1.955075022	0.000282287	0.01176158	0.010582939	1828	tags=25%, list=8%, signal=23%
GO:0061844	BP	GO:0061844	antimicrobial humoral immune response mediated by antimicrobial peptide	36	0.759161893	1.974077411	0.000282287	0.01176158	0.010582939	789	tags=36%, list=4%, signal=35%
GO:0150076	BP	GO:0150076	neuroinflammatory response	35	0.731370482	1.893339891	0.000283688	0.01176158	0.010582939	798	tags=26%, list=4%, signal=25%
GO:0042379	MF	GO:0042379	chemokine receptor binding	34	0.730971319	1.883543623	0.000284495	0.01176158	0.010582939	409	tags=24%, list=2%, signal=23%
GO:0044183	MF	GO:0044183	protein folding chaperone	34	0.732871988	1.888441205	0.000284495	0.01176158	0.010582939	1276	tags=18%, list=6%, signal=17%
GO:0042044	BP	GO:0042044	fluid transport	29	0.772885157	1.939981972	0.000288809	0.011905703	0.010712619	1210	tags=45%, list=6%, signal=42%
GO:0030291	MF	GO:0030291	protein serine/threonine kinase inhibitor activity	28	0.773414131	1.924634962	0.000292227	0.01201219	0.010808435	457	tags=11%, list=2%, signal=11%
GO:0008009	MF	GO:0008009	chemokine activity	25	0.783912946	1.910822821	0.000298374	0.012158809	0.010940362	409	tags=32%, list=2%, signal=31%
GO:1900745	BP	GO:1900745	positive regulation of p38MAPK cascade	25	0.797420433	1.943747923	0.000298374	0.012158809	0.010940362	1383	tags=40%, list=6%, signal=37%
GO:1901071	BP	GO:1901071	glucosamine-containing compound metabolic process	24	0.791103043	1.917589080	0.000300391	0.012158809	0.010940362	489	tags=12%, list=2%, signal=12%
GO:0010243	BP	GO:0010243	response to organonitrogen compound	934	0.400341944	1.356243352	0.000300451	0.012158809	0.010940362	4009	tags=20%, list=19%, signal=17%
GO:0043549	BP	GO:0043549	regulation of kinase activity	827	0.401906811	1.359472507	0.000300692	0.012158809	0.010940362	2738	tags=15%, list=13%, signal=13%
GO:0002825	BP	GO:0002825	regulation of T-helper 1 type immune response	23	0.81809591	1.96985108	0.000301341	0.012158809	0.010940362	483	tags=30%, list=2%, signal=30%
GO:0042330	BP	GO:0042330	taxis	546	0.428058412	1.437117747	0.000303644	0.012158809	0.010940362	3516	tags=22%, list=16%, signal=19%
GO:0003013	BP	GO:0003013	circulatory system process	547	0.430548212	1.445539859	0.000303674	0.012158809	0.010940362	3485	tags=25%, list=16%, signal=22%
GO:0045936	BP	GO:0045936	negative regulation of phosphate metabolic process	462	0.430022399	1.437695089	0.000305686	0.012158809	0.010940362	4042	tags=20%, list=19%, signal=16%
GO:0010563	BP	GO:0010563	negative regulation of phosphorus metabolic process	463	0.429698041	1.436628009	0.00030581	0.012158809	0.010940362	4042	tags=20%, list=19%, signal=16%
GO:0008015	BP	GO:0008015	blood circulation	464	0.428313037	1.431796366	0.000305935	0.012158809	0.010940362	3485	tags=25%, list=16%, signal=22%
GO:0098552	CC	GO:0098552	side of membrane	455	0.434648429	1.45251775	0.000305935	0.012158809	0.010940362	2292	tags=19%, list=11%, signal=17%
GO:0006979	BP	GO:0006979	response to oxidative stress	389	0.446263586	1.483399573	0.000309342	0.012230446	0.01100482	3252	tags=19%, list=15%, signal=16%
GO:0043434	BP	GO:0043434	response to peptide hormone	387	0.445350512	1.480103615	0.000309438	0.012230446	0.01100482	3970	tags=24%, list=18%, signal=20%
GO:0045787	BP	GO:0045787	positive regulation of cell cycle	348	0.452673898	1.497373132	0.000312077	0.012300979	0.011068285	2347	tags=12%, list=11%, signal=11%
GO:0001701	BP	GO:0001701	in utero embryonic development	309	0.460138825	1.516109411	0.00031463	0.012367722	0.011128339	2997	tags=17%, list=14%, signal=15%
GO:0090130	BP	GO:0090130	tissue migration	292	0.465975758	1.531325482	0.000316122	0.012392508	0.011150642	3118	tags=23%, list=14%, signal=20%
GO:0048511	BP	GO:0048511	rhythmic process	280	0.472448971	1.547280369	0.000318505	0.012451993	0.011204166	3043	tags=20%, list=14%, signal=17%
GO:0001822	BP	GO:0001822	kidney development	257	0.489137962	1.593994997	0.000321509	0.012504165	0.011251109	4014	tags=33%, list=19%, signal=27%
GO:0097193	BP	GO:0097193	intrinsic apoptotic signaling pathway	254	0.487141267	1.586318076	0.000321578	0.012504165	0.011251109	2251	tags=13%, list=10%, signal=12%
GO:0003018	BP	GO:0003018	vascular process in circulatory system	227	0.51218812	1.657189499	0.000325239	0.012612416	0.011348512	3247	tags=28%, list=15%, signal=24%
GO:0045927	BP	GO:0045927	positive regulation of growth	222	0.508658492	1.642323718	0.000326513	0.012627796	0.011362351	3435	tags=23%, list=16%, signal=20%
GO:0048711	BP	GO:0048711	positive regulation of astrocyte differentiation	12	0.864020394	1.829122133	0.000328299	0.012662851	0.011393893	880	tags=25%, list=4%, signal=24%
GO:0009612	BP	GO:0009612	response to mechanical stimulus	188	0.527556064	1.685614924	0.000331199	0.012740532	0.01146379	4614	tags=37%, list=21%, signal=29%
GO:0034341	BP	GO:0034341	response to interferon-gamma	164	0.533354876	1.68609671	0.000336852	0.01290064	0.011607853	2323	tags=26%, list=11%, signal=24%
GO:0002285	BP	GO:0002285	lymphocyte activation involved in immune response	162	0.525121742	1.658929006	0.000337154	0.01290064	0.011607853	2146	tags=23%, list=10%, signal=21%
GO:0048861	BP	GO:0048861	leukemia inhibitory factor signaling pathway	8	0.934023219	1.818105633	0.000344649	0.013145584	0.01182825	324	tags=25%, list=1%, signal=25%
GO:0009411	BP	GO:0009411	response to UV	137	0.541732744	1.687151272	0.000345383	0.013145584	0.01182825	2890	tags=13%, list=13%, signal=11%
GO:0071901	BP	GO:0071901	negative regulation of protein serine/threonine kinase activity	124	0.554559218	1.711505791	0.000349528	0.013245828	0.011918449	3154	tags=19%, list=15%, signal=17%
GO:1903900	BP	GO:1903900	regulation of viral life cycle	123	0.538625827	1.660375715	0.000350672	0.013245828	0.011918449	2350	tags=22%, list=11%, signal=20%
GO:1900180	BP	GO:1900180	regulation of protein localization to nucleus	122	0.555604897	1.710858568	0.00035137	0.013245828	0.011918449	2281	tags=16%, list=11%, signal=15%
GO:0003158	BP	GO:0003158	endothelium development	121	0.562711098	1.731116538	0.0003517	0.013245828	0.011918449	3442	tags=35%, list=16%, signal=29%
GO:0019838	MF	GO:0019838	growth factor binding	118	0.567851959	1.741386628	0.000353482	0.013265638	0.011936274	5340	tags=53%, list=25%, signal=40%
GO:0098754	BP	GO:0098754	detoxification	117	0.557964009	1.710005217	0.000354108	0.013265638	0.011936274	2386	tags=28%, list=11%, signal=25%
GO:0002576	BP	GO:0002576	platelet degranulation	112	0.577530356	1.762882402	0.000355534	0.013265638	0.011936274	3523	tags=36%, list=16%, signal=30%
GO:0042476	BP	GO:0042476	odontogenesis	110	0.56892063	1.733117549	0.000355914	0.013265638	0.011936274	3324	tags=37%, list=15%, signal=32%
GO:0007292	BP	GO:0007292	female gamete generation	106	0.561348987	1.701391736	0.000358466	0.01327039	0.01194055	1521	tags=16%, list=7%, signal=15%
GO:0050544	MF	GO:0050544	arachidonic acid binding	6	0.944700716	1.719953355	0.000358809	0.01327039	0.01194055	43	tags=33%, list=0%, signal=33%
GO:0071389	BP	GO:0071389	cellular response to mineralocorticoid stimulus	6	0.946204632	1.722691434	0.000358809	0.01327039	0.01194055	173	tags=50%, list=1%, signal=50%
GO:0002286	BP	GO:0002286	T cell activation involved in immune response	96	0.584815981	1.753824348	0.000364964	0.013428977	0.012083244	2322	tags=34%, list=11%, signal=31%
GO:1990748	BP	GO:1990748	cellular detoxification	96	0.581414182	1.743622579	0.000364964	0.013428977	0.012083244	3114	tags=31%, list=14%, signal=27%
GO:0042116	BP	GO:0042116	macrophage activation	89	0.595676286	1.771225388	0.000366928	0.013439113	0.012092365	1740	tags=24%, list=8%, signal=22%
GO:1904019	BP	GO:1904019	epithelial cell apoptotic process	91	0.60083019	1.78963371	0.000367107	0.013439113	0.012092365	2673	tags=26%, list=12%, signal=23%
GO:0014013	BP	GO:0014013	regulation of gliogenesis	85	0.615987586	1.821820949	0.00036964	0.013497497	0.012144898	1117	tags=13%, list=5%, signal=12%
GO:0045069	BP	GO:0045069	regulation of viral genome replication	68	0.635293144	1.824152379	0.00038373	0.013943959	0.012546619	2069	tags=25%, list=10%, signal=23%
GO:0060395	BP	GO:0060395	SMAD protein signal transduction	67	0.632571239	1.813042397	0.000384615	0.013943959	0.012546619	2578	tags=33%, list=12%, signal=29%
GO:0005518	MF	GO:0005518	collagen binding	65	0.653793921	1.865321474	0.000386498	0.013943959	0.012546619	3887	tags=46%, list=18%, signal=38%
GO:0050891	BP	GO:0050891	multicellular organismal water homeostasis	65	0.66074981	1.88516713	0.000386498	0.013943959	0.012546619	1210	tags=20%, list=6%, signal=19%
GO:0019210	MF	GO:0019210	kinase inhibitor activity	63	0.652788011	1.856064975	0.000387547	0.013943959	0.012546619	2952	tags=19%, list=14%, signal=16%
GO:0044819	BP	GO:0044819	mitotic G1/S transition checkpoint	62	0.657797115	1.865659867	0.000387898	0.013943959	0.012546619	913	tags=11%, list=4%, signal=11%
GO:0004860	MF	GO:0004860	protein kinase inhibitor activity	61	0.65660557	1.857306077	0.000388651	0.013943959	0.012546619	2952	tags=20%, list=14%, signal=17%
GO:0050877	BP	GO:0050877	nervous system process	972	0.396300562	1.34327879	0.00040036	0.014247376	0.012819631	3091	tags=21%, list=14%, signal=19%
GO:0046914	MF	GO:0046914	transition metal ion binding	939	0.399145013	1.3523749	0.000400481	0.014247376	0.012819631	3463	tags=17%, list=16%, signal=15%
GO:0051050	BP	GO:0051050	positive regulation of transport	802	0.400065882	1.35264554	0.000401043	0.014247376	0.012819631	3790	tags=19%, list=18%, signal=16%
GO:0043030	BP	GO:0043030	regulation of macrophage activation	51	0.67338964	1.8538536	0.00040107	0.014247376	0.012819631	1947	tags=27%, list=9%, signal=25%
GO:0006873	BP	GO:0006873	cellular ion homeostasis	600	0.41529443	1.397371629	0.000403551	0.014300226	0.012867185	2626	tags=16%, list=12%, signal=14%
GO:0016049	BP	GO:0016049	cell growth	433	0.436877501	1.457813959	0.000409836	0.014487252	0.013035469	3736	tags=19%, list=17%, signal=16%
GO:0001501	BP	GO:0001501	skeletal system development	399	0.441543548	1.46978887	0.000411396	0.014506739	0.013053003	3197	tags=23%, list=15%, signal=20%
GO:0019216	BP	GO:0019216	regulation of lipid metabolic process	362	0.444359817	1.472490698	0.000414551	0.0145648	0.013105245	4026	tags=22%, list=19%, signal=18%
GO:0042326	BP	GO:0042326	negative regulation of phosphorylation	358	0.4468926	1.480163761	0.000415067	0.0145648	0.013105245	4042	tags=20%, list=19%, signal=17%
GO:1900744	BP	GO:1900744	regulation of p38MAPK cascade	38	0.701241223	1.842176374	0.000419287	0.014677093	0.013206285	1383	tags=26%, list=6%, signal=25%
GO:0042088	BP	GO:0042088	T-helper 1 type immune response	36	0.717255781	1.865107363	0.00042343	0.014786127	0.013304393	871	tags=25%, list=4%, signal=24%
GO:0009897	CC	GO:0009897	external side of plasma membrane	262	0.481872389	1.572794132	0.000427762	0.014900293	0.013407118	2292	tags=23%, list=11%, signal=21%
GO:0034504	BP	GO:0034504	protein localization to nucleus	254	0.483982503	1.576031934	0.000428771	0.014900293	0.013407118	2281	tags=13%, list=11%, signal=12%
GO:0150077	BP	GO:0150077	regulation of neuroinflammatory response	31	0.748358735	1.897957908	0.000430231	0.014906955	0.013413113	798	tags=29%, list=4%, signal=28%
GO:0060326	BP	GO:0060326	cell chemotaxis	239	0.490559165	1.592923292	0.000431034	0.014906955	0.013413113	2738	tags=20%, list=13%, signal=18%
GO:0043903	BP	GO:0043903	regulation of biological process involved in symbiotic interaction	168	0.532653488	1.688211632	0.000447427	0.015436778	0.013889842	2864	tags=21%, list=13%, signal=19%
GO:0043409	BP	GO:0043409	negative regulation of MAPK cascade	157	0.531046845	1.67436158	0.000452028	0.015558215	0.013999109	2401	tags=17%, list=11%, signal=15%
GO:0030307	BP	GO:0030307	positive regulation of cell growth	144	0.532312296	1.663533492	0.000458716	0.015750695	0.014172301	3873	tags=22%, list=18%, signal=18%
GO:0046906	MF	GO:0046906	tetrapyrrole binding	129	0.538041819	1.667963088	0.000463338	0.015871545	0.01428104	3205	tags=31%, list=15%, signal=27%
GO:0050832	BP	GO:0050832	defense response to fungus	19	0.796641993	1.843872154	0.000465983	0.01592423	0.014328445	789	tags=37%, list=4%, signal=36%
GO:0020037	MF	GO:0020037	heme binding	121	0.540586381	1.663052368	0.000468933	0.015987065	0.014384984	3205	tags=31%, list=15%, signal=27%
GO:1904996	BP	GO:1904996	positive regulation of leukocyte adhesion to vascular endothelial cell	17	0.82172842	1.860783571	0.000472813	0.016081239	0.014469721	1097	tags=35%, list=5%, signal=34%
GO:0002523	BP	GO:0002523	leukocyte migration involved in inflammatory response	15	0.835831967	1.84761999	0.000477251	0.016193893	0.014571085	1097	tags=33%, list=5%, signal=32%
GO:0055082	BP	GO:0055082	cellular chemical homeostasis	727	0.400955705	1.353737322	0.000502563	0.017009899	0.015305319	2626	tags=15%, list=12%, signal=14%
GO:0055065	BP	GO:0055065	metal ion homeostasis	578	0.417784916	1.404703321	0.000504796	0.017009899	0.015305319	2626	tags=16%, list=12%, signal=15%
GO:0005198	MF	GO:0005198	structural molecule activity	581	0.418690863	1.407814806	0.000504847	0.017009899	0.015305319	3887	tags=22%, list=18%, signal=19%
GO:0006875	BP	GO:0006875	cellular metal ion homeostasis	513	0.41822026	1.4024312	0.000507048	0.017044157	0.015336143	2626	tags=16%, list=12%, signal=14%
GO:0009314	BP	GO:0009314	response to radiation	404	0.440704393	1.467450813	0.000514245	0.017245773	0.015517556	4110	tags=21%, list=19%, signal=17%
GO:0006026	BP	GO:0006026	aminoglycan catabolic process	62	0.643750749	1.825821227	0.000517197	0.017304442	0.015570346	3887	tags=35%, list=18%, signal=29%
GO:0031589	BP	GO:0031589	cell-substrate adhesion	331	0.450279936	1.486769692	0.000521866	0.01742016	0.015674467	4063	tags=31%, list=19%, signal=26%
GO:0051348	BP	GO:0051348	negative regulation of transferase activity	261	0.477803913	1.559056461	0.000534645	0.017805411	0.016021111	3733	tags=19%, list=17%, signal=16%
GO:1902893	BP	GO:1902893	regulation of pri-miRNA transcription by RNA polymerase II	47	0.689341569	1.872746817	0.000542814	0.018035732	0.016228352	3118	tags=43%, list=14%, signal=36%
GO:0007188	BP	GO:0007188	adenylate cyclase-modulating G protein-coupled receptor signaling pathway	200	0.515988231	1.654785561	0.000549269	0.018208156	0.016383497	2717	tags=26%, list=13%, signal=23%
GO:0045071	BP	GO:0045071	negative regulation of viral genome replication	41	0.698312905	1.855697772	0.00055371	0.018276215	0.016444736	2069	tags=37%, list=10%, signal=33%
GO:2000351	BP	GO:2000351	regulation of endothelial cell apoptotic process	40	0.700032806	1.855550829	0.000553863	0.018276215	0.016444736	2595	tags=35%, list=12%, signal=31%
GO:0042445	BP	GO:0042445	hormone metabolic process	176	0.512250075	1.628437042	0.000557227	0.018345145	0.016506758	3197	tags=35%, list=15%, signal=30%
GO:0044409	BP	GO:0044409	entry into host	138	0.528865177	1.648651117	0.000575639	0.018908031	0.017013237	2350	tags=25%, list=11%, signal=23%
GO:0007189	BP	GO:0007189	adenylate cyclase-activating G protein-coupled receptor signaling pathway	119	0.545444846	1.67371104	0.000588443	0.019284577	0.017352048	2717	tags=29%, list=13%, signal=26%
GO:0051082	MF	GO:0051082	unfolded protein binding	104	0.565418637	1.710536026	0.000599592	0.019605305	0.017640636	3274	tags=17%, list=15%, signal=15%
GO:0070161	CC	GO:0070161	anchoring junction	784	0.398381196	1.346982369	0.000601624	0.019627143	0.017660286	5123	tags=29%, list=24%, signal=23%
GO:0090100	BP	GO:0090100	positive regulation of transmembrane receptor protein serine/threonine kinase signaling pathway	92	0.581998964	1.735796586	0.000611172	0.01986075	0.017870483	2337	tags=24%, list=11%, signal=21%
GO:0032963	BP	GO:0032963	collagen metabolic process	89	0.589603778	1.753168968	0.000611546	0.01986075	0.017870483	2359	tags=26%, list=11%, signal=23%
GO:0051591	BP	GO:0051591	response to cAMP	85	0.59482738	1.759238344	0.000616067	0.019962514	0.017962049	2323	tags=24%, list=11%, signal=21%
GO:0005902	CC	GO:0005902	microvillus	83	0.600203414	1.768642499	0.000618659	0.020001446	0.017997079	3105	tags=33%, list=14%, signal=28%
GO:0071158	BP	GO:0071158	positive regulation of cell cycle arrest	77	0.611313055	1.787214789	0.000624376	0.020129862	0.018112626	1002	tags=10%, list=5%, signal=10%
GO:0004867	MF	GO:0004867	serine-type endopeptidase inhibitor activity	75	0.614779046	1.792292666	0.000625861	0.020129862	0.018112626	1572	tags=31%, list=7%, signal=29%
GO:0010038	BP	GO:0010038	response to metal ion	328	0.447514406	1.477278709	0.000626828	0.020129862	0.018112626	2879	tags=19%, list=13%, signal=17%
GO:0017014	BP	GO:0017014	protein nitrosylation	15	0.818149941	1.808533588	0.000636335	0.020344328	0.018305601	43	tags=13%, list=0%, signal=13%
GO:0018119	BP	GO:0018119	peptidyl-cysteine S-nitrosylation	15	0.818149941	1.808533588	0.000636335	0.020344328	0.018305601	43	tags=13%, list=0%, signal=13%
GO:0020027	BP	GO:0020027	hemoglobin metabolic process	14	0.836639464	1.826144511	0.00064288	0.020508018	0.018452887	1804	tags=29%, list=8%, signal=26%
GO:0043393	BP	GO:0043393	regulation of protein binding	175	0.515137672	1.637503021	0.000668598	0.021281243	0.019148627	3531	tags=21%, list=16%, signal=18%
GO:0001706	BP	GO:0001706	endoderm formation	48	0.680825547	1.858314139	0.000674036	0.021359819	0.019219328	4063	tags=46%, list=19%, signal=37%
GO:0061614	BP	GO:0061614	pri-miRNA transcription by RNA polymerase II	48	0.684777693	1.869101526	0.000674036	0.021359819	0.019219328	3118	tags=42%, list=14%, signal=36%
GO:0051338	BP	GO:0051338	regulation of transferase activity	923	0.392813058	1.330669181	0.000700911	0.022123261	0.019906265	2401	tags=13%, list=11%, signal=12%
GO:0002673	BP	GO:0002673	regulation of acute inflammatory response	37	0.694431169	1.813759244	0.000702741	0.022123261	0.019906265	1159	tags=24%, list=5%, signal=23%
GO:1902895	BP	GO:1902895	positive regulation of pri-miRNA transcription by RNA polymerase II	37	0.696034987	1.817948197	0.000702741	0.022123261	0.019906265	4209	tags=59%, list=19%, signal=48%
GO:0032870	BP	GO:0032870	cellular response to hormone stimulus	558	0.416683967	1.399769962	0.000707929	0.022188584	0.019965042	4544	tags=26%, list=21%, signal=21%
GO:0070851	MF	GO:0070851	growth factor receptor binding	116	0.547390103	1.675226026	0.000710059	0.022188584	0.019965042	3118	tags=23%, list=14%, signal=20%
GO:0045598	BP	GO:0045598	regulation of fat cell differentiation	113	0.547875907	1.673358039	0.000710985	0.022188584	0.019965042	3678	tags=31%, list=17%, signal=26%
GO:2001235	BP	GO:2001235	positive regulation of apoptotic signaling pathway	113	0.550103718	1.680162365	0.000710985	0.022188584	0.019965042	2470	tags=18%, list=11%, signal=16%
GO:0030055	CC	GO:0030055	cell-substrate junction	411	0.434627162	1.447748185	0.000719498	0.022405679	0.020160381	5603	tags=34%, list=26%, signal=26%
GO:0001933	BP	GO:0001933	negative regulation of protein phosphorylation	317	0.449546889	1.48200954	0.000733599	0.022670065	0.020398274	4042	tags=19%, list=19%, signal=16%
GO:0001666	BP	GO:0001666	response to hypoxia	311	0.446250989	1.470696694	0.000733753	0.022670065	0.020398274	3590	tags=20%, list=17%, signal=17%
GO:0016324	CC	GO:0016324	apical plasma membrane	311	0.446183309	1.470473642	0.000733753	0.022670065	0.020398274	2622	tags=22%, list=12%, signal=19%
GO:0010948	BP	GO:0010948	negative regulation of cell cycle process	310	0.448460932	1.477589797	0.000734291	0.022670065	0.020398274	2603	tags=11%, list=12%, signal=10%
GO:0098869	BP	GO:0098869	cellular oxidant detoxification	85	0.584353534	1.728261305	0.00073928	0.02277522	0.02049289	3114	tags=31%, list=14%, signal=26%
GO:0023026	MF	GO:0023026	MHC class II protein complex binding	12	-0.836788512	-1.982171876	0.000767263	0.023586483	0.021222856	1671	tags=42%, list=8%, signal=38%
GO:0097191	BP	GO:0097191	extrinsic apoptotic signaling pathway	199	0.50570014	1.621153925	0.000768893	0.023586483	0.021222856	4792	tags=34%, list=22%, signal=27%
GO:0001836	BP	GO:0001836	release of cytochrome c from mitochondria	53	0.648992317	1.795560859	0.00079968	0.024478719	0.02202568	906	tags=13%, list=4%, signal=13%
GO:0019953	BP	GO:0019953	sexual reproduction	658	0.400632031	1.350670619	0.000805477	0.024564417	0.02210279	3797	tags=21%, list=18%, signal=18%
GO:0034614	BP	GO:0034614	cellular response to reactive oxygen species	138	0.52179642	1.626615416	0.000805895	0.024564417	0.02210279	2935	tags=20%, list=14%, signal=17%
GO:1902532	BP	GO:1902532	negative regulation of intracellular signal transduction	453	0.424213131	1.417191515	0.000816493	0.024834856	0.022346129	3955	tags=21%, list=18%, signal=18%
GO:0072503	BP	GO:0072503	cellular divalent inorganic cation homeostasis	428	0.428416579	1.429545317	0.000819588	0.024849489	0.022359295	2626	tags=17%, list=12%, signal=15%
GO:0009991	BP	GO:0009991	response to extracellular stimulus	425	0.430517553	1.435810796	0.000820429	0.024849489	0.022359295	3554	tags=23%, list=16%, signal=19%
GO:0045471	BP	GO:0045471	response to ethanol	114	0.542980298	1.65993643	0.000827325	0.025005736	0.022499884	2279	tags=19%, list=11%, signal=17%
GO:0043114	BP	GO:0043114	regulation of vascular permeability	40	0.685699796	1.817558853	0.000830795	0.025057955	0.022546871	1415	tags=22%, list=7%, signal=21%
GO:1903706	BP	GO:1903706	regulation of hemopoiesis	340	0.443378223	1.465539897	0.00083342	0.025084552	0.022570802	3133	tags=22%, list=14%, signal=19%
GO:0036293	BP	GO:0036293	response to decreased oxygen levels	322	0.445368258	1.469042776	0.000836645	0.025129045	0.022610836	3925	tags=22%, list=18%, signal=18%

GO:0071492	BP	GO:0071492	cellular response to UV-A	10	0.885781098	1.80599867	0.000839349	0.025157728	0.022636644	94	tags=40%, list=0%, signal=40%
GO:0090132	BP	GO:0090132	epithelium migration	287	0.461850843	1.514552616	0.000846113	0.025307755	0.022771637	3118	tags=23%, list=14%, signal=20%
GO:0044773	BP	GO:0044773	mitotic DNA damage checkpoint	97	0.572024301	1.716528669	0.000851064	0.025403019	0.022857355	1039	tags=9%, list=5%, signal=9%
GO:1903707	BP	GO:1903707	negative regulation of hemopoiesis	90	0.575849149	1.71483368	0.000854388	0.025440328	0.022890926	3246	tags=34%, list=15%, signal=29%
GO:0008630	BP	GO:0008630	intrinsic apoptotic signaling pathway in response to DNA damage	93	0.57301539	1.711184734	0.00085585	0.025440328	0.022890926	1827	tags=14%, list=8%, signal=13%
GO:1902106	BP	GO:1902106	negative regulation of leukocyte differentiation	87	0.581346029	1.72416198	0.000857843	0.025446988	0.022896918	2608	tags=31%, list=12%, signal=27%
GO:0033673	BP	GO:0033673	negative regulation of kinase activity	233	0.483369229	1.567338786	0.000863838	0.025544396	0.022984565	3733	tags=19%, list=17%, signal=16%
GO:0045926	BP	GO:0045926	negative regulation of growth	229	0.485203985	1.571860617	0.000864678	0.025544396	0.022984565	3843	tags=28%, list=18%, signal=23%
GO:0006469	BP	GO:0006469	negative regulation of protein kinase activity	212	0.495814814	1.595742748	0.000875082	0.025798781	0.023213458	2401	tags=13%, list=11%, signal=12%
GO:0032602	BP	GO:0032602	chemokine production	72	0.607730272	1.759311571	0.000884844	0.026033229	0.023424411	750	tags=17%, list=3%, signal=16%
GO:0010638	BP	GO:0010638	positive regulation of organelle organization	533	0.412365213	1.383897552	0.000911577	0.026765018	0.024082866	3662	tags=16%, list=17%, signal=13%
GO:0030168	BP	GO:0030168	platelet activation	142	0.526610206	1.645057461	0.000918274	0.026906727	0.024210375	4092	tags=35%, list=19%, signal=29%
GO:0030728	BP	GO:0030728	ovulation	16	0.79766184	1.786944268	0.000951022	0.027809672	0.025022835	1521	tags=44%, list=7%, signal=41%
GO:1901985	BP	GO:1901985	positive regulation of protein acetylation	41	0.683324116	1.815866542	0.000968992	0.028228243	0.02539946	913	tags=7%, list=4%, signal=7%
GO:0043616	BP	GO:0043616	keratinocyte proliferation	40	0.681533777	1.806516142	0.000969261	0.028228243	0.02539946	3051	tags=38%, list=14%, signal=32%
GO:0036041	MF	GO:0036041	long-chain fatty acid binding	13	0.834120661	1.79311227	0.000975768	0.028360364	0.025518341	170	tags=23%, list=1%, signal=23%
GO:2000116	BP	GO:2000116	regulation of cysteine-type endopeptidase activity	209	0.490707664	1.57915901	0.000984144	0.028546138	0.025685499	2251	tags=13%, list=10%, signal=12%
GO:0002449	BP	GO:0002449	lymphocyte mediated immunity	197	0.492063831	1.576777712	0.000988468	0.028613858	0.025746432	2935	tags=21%, list=14%, signal=19%
GO:1901215	BP	GO:1901215	negative regulation of neuron death	173	0.512436155	1.628134259	0.001002674	0.028966803	0.026064008	1311	tags=11%, list=6%, signal=10%
GO:0030003	BP	GO:0030003	cellular cation homeostasis	588	0.409239384	1.376227255	0.001009489	0.029105253	0.026188584	2626	tags=16%, list=12%, signal=14%
GO:0052126	BP	GO:0052126	movement in host environment	158	0.51145262	1.61370224	0.001016375	0.029245172	0.026314482	2350	tags=22%, list=11%, signal=20%
GO:0072507	BP	GO:0072507	divalent inorganic cation homeostasis	444	0.424499687	1.417262368	0.001022599	0.029344057	0.026403457	2626	tags=17%, list=12%, signal=15%
GO:0030203	BP	GO:0030203	glycosaminoglycan metabolic process	150	0.517371972	1.624487082	0.001023891	0.029344057	0.026403457	4377	tags=34%, list=20%, signal=27%
GO:0035066	BP	GO:0035066	positive regulation of histone acetylation	27	0.742618827	1.837363916	0.001027146	0.029356611	0.026414754	913	tags=11%, list=4%, signal=11%
GO:0033627	BP	GO:0033627	cell adhesion mediated by integrin	66	0.616882771	1.764292054	0.00102841	0.029356611	0.026414754	4379	tags=48%, list=20%, signal=39%
GO:2001236	BP	GO:2001236	regulation of extrinsic apoptotic signaling pathway	142	0.524319472	1.637901524	0.001033058	0.029427454	0.026478497	4792	tags=40%, list=22%, signal=31%
GO:0045177	CC	GO:0045177	apical part of cell	367	0.436575584	1.44765073	0.001034982	0.029427454	0.026478497	2622	tags=21%, list=12%, signal=19%
GO:0010631	BP	GO:0010631	epithelial cell migration	285	0.460279432	1.509456779	0.001056971	0.029993368	0.0269877	3118	tags=23%, list=14%, signal=20%
GO:0045178	CC	GO:0045178	basal part of cell	239	0.476769098	1.548144761	0.001077586	0.030473895	0.027420073	3380	tags=26%, list=16%, signal=22%
GO:1904724	CC	GO:1904724	tertiary granule lumen	47	0.671328942	1.823811585	0.001085629	0.030473895	0.027420073	1440	tags=23%, list=7%, signal=22%
GO:0072577	BP	GO:0072577	endothelial cell apoptotic process	46	0.668860014	1.810687569	0.001087104	0.030473895	0.027420073	1442	tags=24%, list=7%, signal=22%
GO:0006833	BP	GO:0006833	water transport	19	0.784373952	1.815477092	0.001087294	0.030473895	0.027420073	1210	tags=53%, list=6%, signal=50%
GO:0046822	BP	GO:0046822	regulation of nucleocytoplasmic transport	99	0.56358761	1.695160196	0.001088271	0.030473895	0.027420073	2281	tags=11%, list=11%, signal=10%
GO:0043330	BP	GO:0043330	response to exogenous dsRNA	45	0.673917581	1.817953903	0.001088583	0.030473895	0.027420073	2165	tags=36%, list=10%, signal=32%
GO:0030246	MF	GO:0030246	carbohydrate binding	219	0.485328151	1.565811441	0.001088732	0.030473895	0.027420073	2864	tags=24%, list=13%, signal=21%
GO:0045649	BP	GO:0045649	regulation of macrophage differentiation	18	0.790517218	1.814526145	0.001092555	0.030521532	0.027462936	2378	tags=33%, list=11%, signal=30%
GO:0043035	MF	GO:0043035	chromatin insulator sequence binding	5	0.95101626	1.668577084	0.00109529	0.030538645	0.027478334	245	tags=20%, list=1%, signal=20%
GO:0010001	BP	GO:0010001	glial cell differentiation	195	0.496798732	1.590113894	0.001101322	0.030591313	0.027525725	1828	tags=11%, list=8%, signal=10%
GO:0000785	CC	GO:0000785	chromatin	924	0.387661563	1.313184965	0.001101432	0.030591313	0.027525725	3173	tags=17%, list=15%, signal=15%
GO:0032092	BP	GO:0032092	positive regulation of protein binding	77	0.591882329	1.730407757	0.001123876	0.031154539	0.028032509	3531	tags=23%, list=16%, signal=20%
GO:0060298	BP	GO:0060298	positive regulation of sarcomere organization	4	0.973252851	1.619834198	0.001127396	0.031192004	0.02806622	390	tags=75%, list=2%, signal=74%
GO:0030323	BP	GO:0030323	respiratory tube development	155	0.50730123	1.598394734	0.001131222	0.031237787	0.028107414	4407	tags=35%, list=20%, signal=28%
GO:0071346	BP	GO:0071346	cellular response to interferon-gamma	148	0.509023898	1.596352806	0.001139471	0.031405313	0.028258153	2323	tags=24%, list=11%, signal=22%
GO:0044319	BP	GO:0044319	wound healing, spreading of cells	31	0.7291107	1.8491418	0.001147282	0.031499907	0.028343268	2359	tags=39%, list=11%, signal=35%
GO:0090505	BP	GO:0090505	epiboly involved in wound healing	31	0.7291107	1.8491418	0.001147282	0.031499907	0.028343268	2359	tags=39%, list=11%, signal=35%
GO:0046882	BP	GO:0046882	negative regulation of follicle-stimulating hormone secretion	3	0.984048858	1.553219398	0.001154512	0.031638033	0.028467551	35	tags=33%, list=0%, signal=33%
GO:0050680	BP	GO:0050680	negative regulation of epithelial cell proliferation	120	0.530398184	1.629965871	0.001173158	0.032087882	0.028872321	3123	tags=23%, list=14%, signal=20%
GO:0021782	BP	GO:0021782	glial cell development	102	0.558317707	1.686438129	0.001201634	0.032739373	0.029458525	1828	tags=15%, list=8%, signal=14%
GO:0097237	BP	GO:0097237	cellular response to toxic substance	103	0.555856639	1.679715534	0.001201634	0.032739373	0.029458525	3114	tags=29%, list=14%, signal=25%
GO:0044774	BP	GO:0044774	mitotic DNA integrity checkpoint	101	0.555293808	1.675854679	0.001203804	0.032739373	0.029458525	1039	tags=9%, list=5%, signal=9%
GO:0043281	BP	GO:0043281	regulation of cysteine-type endopeptidase activity involved in apoptotic process	189	0.493122658	1.575331659	0.001215067	0.032983333	0.029678037	2251	tags=13%, list=10%, signal=11%
GO:0045785	BP	GO:0045785	positive regulation of cell adhesion	359	0.437823589	1.449825237	0.001246106	0.0336397	0.030268629	3716	tags=26%, list=17%, signal=22%
GO:0002823	BP	GO:0002823	negative regulation of adaptive immune response based on somatic recombination of immune receptors built from immunoglobulin superfamily domains	40	0.678431226	1.798292326	0.001246192	0.0336397	0.030268629	2608	tags=30%, list=12%, signal=26%
GO:0005581	CC	GO:0005581	collagen trimer	78	0.588820665	1.724340112	0.001246261	0.0336397	0.030268629	3997	tags=50%, list=18%, signal=41%
GO:0030324	BP	GO:0030324	lung development	151	0.5104416	1.604077658	0.001249006	0.033650667	0.030278497	3133	tags=26%, list=14%, signal=22%
GO:0007178	BP	GO:0007178	transmembrane receptor protein serine/threonine kinase signaling pathway	321	0.442872359	1.460673384	0.00125523	0.033755132	0.030372494	4235	tags=31%, list=20%, signal=25%
GO:0046688	BP	GO:0046688	response to copper ion	38	0.679893926	1.786096547	0.001257862	0.03376279	0.030379384	2259	tags=37%, list=10%, signal=33%
GO:2000045	BP	GO:2000045	regulation of G1/S transition of mitotic cell cycle	144	0.512170451	1.600588047	0.001261468	0.033796534	0.030409746	2346	tags=11%, list=11%, signal=10%
GO:0045601	BP	GO:0045601	regulation of endothelial cell differentiation	35	0.705476021	1.826305442	0.001276596	0.034138258	0.030717227	3104	tags=51%, list=14%, signal=44%
GO:0006953	BP	GO:0006953	acute-phase response	32	0.718897334	1.834226809	0.001285714	0.034250684	0.030818386	937	tags=25%, list=4%, signal=24%
GO:0090504	BP	GO:0090504	epiboly	32	0.724802957	1.849294681	0.001285714	0.034250684	0.030818386	2359	tags=38%, list=11%, signal=33%
GO:0051783	BP	GO:0051783	regulation of nuclear division	122	0.530643743	1.633996385	0.001288358	0.034250684	0.030818386	2264	tags=14%, list=10%, signal=13%
GO:0051091	BP	GO:0051091	positive regulation of DNA-binding transcription factor activity	244	0.476228497	1.547826477	0.001290323	0.034250684	0.030818386	2580	tags=15%, list=12%, signal=13%
GO:0010837	BP	GO:0010837	regulation of keratinocyte proliferation	30	0.732698775	1.848396846	0.001293289	0.034266211	0.030832357	1799	tags=33%, list=8%, signal=31%
GO:0051101	BP	GO:0051101	regulation of DNA binding	105	0.548498552	1.661612363	0.001316262	0.03481077	0.031322345	2003	tags=16%, list=9%, signal=15%
GO:1900046	BP	GO:1900046	regulation of hemostasis	57	0.623857045	1.742813024	0.001319087	0.034821482	0.031331983	4063	tags=53%, list=19%, signal=43%
GO:0005925	CC	GO:0005925	focal adhesion	404	0.428731865	1.427584872	0.001337036	0.035230651	0.031700149	5388	tags=32%, list=25%, signal=24%
GO:0032379	BP	GO:0032379	positive regulation of intracellular lipid transport	3	0.980879555	1.548216981	0.001346931	0.035297441	0.031760246	115	tags=33%, list=1%, signal=33%
GO:0032382	BP	GO:0032382	positive regulation of intracellular sterol transport	3	0.980879555	1.548216981	0.001346931	0.035297441	0.031760246	115	tags=33%, list=1%, signal=33%
GO:0032385	BP	GO:0032385	positive regulation of intracellular cholesterol transport	3	0.980879555	1.548216981	0.001346931	0.035297441	0.031760246	115	tags=33%, list=1%, signal=33%
GO:0030098	BP	GO:0030098	lymphocyte differentiation	308	0.445875248	1.469159381	0.001363255	0.035660273	0.032086718	2322	tags=20%, list=11%, signal=19%
GO:0048771	BP	GO:0048771	tissue remodeling	149	0.506635542	1.58984409	0.001365809	0.035662246	0.032088494	3003	tags=25%, list=14%, signal=22%
GO:0042026	BP	GO:0042026	protein refolding	22	0.758975568	1.80899962	0.001369655	0.035697862	0.032120541	12	tags=9%, list=0%, signal=9%
GO:0035987	BP	GO:0035987	endodermal cell differentiation	40	0.67584501	1.791437141	0.001384658	0.036023643	0.032413675	4063	tags=45%, list=19%, signal=37%
GO:0051084	BP	GO:0051084	'de novo' posttranslational protein folding	37	0.686376201	1.792720768	0.001405481	0.036499387	0.032841744	1276	tags=14%, list=6%, signal=13%
GO:1901681	MF	GO:1901681	sulfur compound binding	219	0.478345964	1.54328485	0.001415351	0.036689471	0.03301278	4097	tags=31%, list=19%, signal=25%
GO:0008180	CC	GO:0008180	COP9 signalosome	33	0.719244967	1.844299074	0.001424501	0.036846567	0.033154133	12	tags=6%, list=0%, signal=6%
GO:0001946	BP	GO:0001946	lymphangiogenesis	16	0.788807797	1.767109194	0.001426534	0.036846567	0.033154133	1000	tags=25%, list=5%, signal=24%
GO:0035743	BP	GO:0035743	CD4-positive, alpha-beta T cell cytokine production	15	0.797026523	1.761839933	0.001431753	0.036915111	0.033215808	2146	tags=53%, list=10%, signal=48%
GO:0002765	BP	GO:0002765	immune response-inhibiting signal transduction	3	-0.96996285	-1.598038086	0.001456816	0.037494113	0.033736787	243	tags=67%, list=1%, signal=66%
GO:0038061	BP	GO:0038061	NIK/NF-kappaB signaling	161	0.498144564	1.573901372	0.001460018	0.037509426	0.033750566	2945	tags=17%, list=14%, signal=15%
GO:0050792	BP	GO:0050792	regulation of viral process	159	0.50299261	1.587722563	0.00146611	0.037598804	0.033830987	2575	tags=19%, list=12%, signal=17%
GO:0045620	BP	GO:0045620	negative regulation of lymphocyte differentiation	47	0.654177143	1.777214981	0.00149274	0.038213609	0.034384182	3663	tags=45%, list=17%, signal=37%
GO:0005509	MF	GO:0005509	calcium ion binding	626	0.39949298	1.345290989	0.001512402	0.038648176	0.034775201	3367	tags=19%, list=16%, signal=17%
GO:0006458	BP	GO:0006458	'de novo' protein folding	41	0.671193545	1.783630745	0.001522702	0.038784393	0.034897767	1276	tags=12%, list=6%, signal=11%
GO:0030225	BP	GO:0030225	macrophage differentiation	40	0.673209987	1.784452583	0.001523124	0.038784393	0.034897767	2378	tags=28%, list=11%, signal=25%
GO:0071260	BP	GO:0071260	cellular response to mechanical stimulus	70	0.600657878	1.732037342	0.001526329	0.038797346	0.034909422	2349	tags=26%, list=11%, signal=23%
GO:1903901	BP	GO:1903901	negative regulation of viral life cycle	19	0.778566818	1.802036158	0.001553277	0.039412702	0.035463113	2575	tags=53%, list=12%, signal=46%
GO:0060541	BP	GO:0060541	respiratory system development	173	0.50156886	1.593606221	0.001559715	0.039506368	0.035547392	3133	tags=25%, list=14%, signal=21%
GO:2000758	BP	GO:2000758	positive regulation of peptidyl-lysine acetylation	32	0.714569772	1.823185276	0.001571429	0.039733116	0.035751417	913	tags=9%, list=4%, signal=9%
GO:0035821	BP	GO:0035821	modulation of process of other organism	98	0.554412428	1.665760023	0.001576331	0.039787142	0.03580003	1581	tags=16%, list=7%, signal=15%
GO:0009408	BP	GO:0009408	response to heat	150	0.505000433	1.585641908	0.001592719	0.040130382	0.036108873	2325	tags=13%, list=11%, signal=11%
GO:0001818	BP	GO:0001818	negative regulation of cytokine production	263	0.466474388	1.523017207	0.001602393	0.040303543	0.036264682	3140	tags=25%, list=15%, signal=21%
GO:0001780	BP	GO:0001780	neutrophil homeostasis	14	0.810978534	1.770134044	0.0016072	0.040329543	0.036288076	265	tags=21%, list=1%, signal=21%
GO:0002323	BP	GO:0002323	natural killer cell activation involved in immune response	27	0.725459949	1.794909966	0.001614087	0.040329543	0.036288076	2146	tags=56%, list=10%, signal=50%
GO:0140467	BP	GO:0140467	integrated stress response signaling	27	0.723606616	1.790324507	0.001614087	0.040329543	0.036288076	886	tags=19%, list=4%, signal=18%
GO:0003097	BP	GO:0003097	renal water transport	6	0.929919295	1.69304181	0.001614639	0.040329543	0.036288076	1210	tags=83%, list=6%, signal=79%
GO:0046718	BP	GO:0046718	viral entry into host cell	126	0.52242313	1.614214033	0.001627528	0.040533592	0.036471677	2776	tags=29%, list=13%, signal=25%
GO:0051703	BP	GO:0051703	biological process involved in intraspecies interaction between organisms	47	0.65379491	1.776176561	0.001628443	0.040533592	0.036471677	869	tags=15%, list=4%, signal=14%
GO:0045637	BP	GO:0045637	regulation of myeloid cell differentiation	207	0.48353447	1.554884054	0.001641317	0.040783478	0.036696522	2580	tags=19%, list=12%, signal=17%
GO:0061436	BP	GO:0061436	establishment of skin barrier	22	0.757034874	1.804374022	0.001674022	0.04152441	0.037363204	626	tags=18%, list=3%, signal=18%
GO:1903409	BP	GO:1903409	reactive oxygen species biosynthetic process	101	0.547861692	1.653424849	0.001685326	0.04167217	0.037496157	3914	tags=31%, list=18%, signal=25%
GO:1902806	BP	GO:1902806	regulation of cell cycle G1/S phase transition	162	0.497955543	1.573107393	0.001685772	0.04167217	0.037496157	1946	tags=11%, list=9%, signal=10%
GO:0001935	BP	GO:0001935	endothelial cell proliferation	145	0.509269904	1.592816642	0.001717426	0.042381835	0.038134706	3133	tags=28%, list=14%, signal=24%
GO:1904862	BP	GO:1904862	inhibitory synapse assembly	16	0.780469197	1.74842883	0.001743541	0.04295261	0.038648282	1416	tags=19%, list=7%, signal=18%
GO:0007159	BP	GO:0007159	leukocyte cell-cell adhesion	296	0.447395694	1.47062433	0.001791737	0.044064477	0.039648728	2980	tags=26%, list=14%, signal=22%
GO:0030278	BP	GO:0030278	regulation of ossification	105	0.538096346	1.630100092	0.001794902	0.044067	0.039650998	4163	tags=38%, list=19%, signal=31%
GO:0008270	MF	GO:0008270	zinc ion binding	707	0.391630539	1.322364379	0.001808318	0.044320741	0.039879311	3463	tags=15%, list=16%, signal=13%
GO:0007600	BP	GO:0007600	sensory perception	573	0.404118604	1.358337852	0.001818917	0.044461699	0.040006144	3090	tags=25%, list=14%, signal=22%
GO:0061138	BP	GO:0061138	morphogenesis of a branching epithelium	150	0.502923027	1.579119097	0.00182025	0.044461699	0.040006144	3708	tags=33%, list=17%, signal=28%
GO:0006909	BP	GO:0006909	phagocytosis	243	0.468479831	1.52281044	0.001827171	0.044512672	0.040052009	3679	tags=24%, list=17%, signal=20%
GO:0051223	BP	GO:0051223	regulation of protein transport	499	0.409451772	1.371795099	0.001828525	0.044512672	0.040052009	4833	tags=25%, list=22%, signal=20%
GO:0001667	BP	GO:0001667	ameboidal-type cell migration	392	0.4237707	1.409143622	0.001856244	0.045111124	0.040590489	3118	tags=21%, list=14%, signal=18%
GO:0022407	BP	GO:0022407	regulation of cell-cell adhesion	356	0.430967948	1.427393252	0.001867801	0.045315439	0.04077433	2980	tags=23%, list=14%, signal=20%
GO:0043331	BP	GO:0043331	response to dsRNA	51	0.626149821	1.723801542	0.001871658	0.045332559	0.040789734	2165	tags=31%, list=10%, signal=28%
GO:0045216	BP	GO:0045216	cell-cell junction organization	187	0.488645996	1.561189383	0.001874931	0.045335518	0.040792397	4006	tags=33%, list=19%, signal=27%
GO:0006936	BP	GO:0006936	muscle contraction	314	0.439390608	1.448088023	0.00188699	0.045550552	0.040985881	4199	tags=31%, list=19%, signal=25%
GO:0038066	BP	GO:0038066	p38MAPK cascade	44	0.660188096	1.774588367	0.001916233	0.046178974	0.041551329	2325	tags=30%, list=11%, signal=26%
GO:0007276	BP	GO:0007276	gamete generation	538	0.405192361	1.360174023	0.001923466	0.046228673	0.041596048	3797	tags=20%, list=18%, signal=17%
GO:0042063	BP	GO:0042063	gliogenesis	261	0.462101076	1.507818687	0.001924722	0.046228673	0.041596048	1156	tags=8%, list=5%, signal=8%
GO:0022617	BP	GO:0022617	extracellular matrix disassembly	65	0.602468635	1.718886712	0.001932492	0.046337928	0.041694354	1688	tags=20%, list=8%, signal=18%
GO:0071674	BP	GO:0071674	mononuclear cell migration	147	0.50256852	1.574339556	0.001942635	0.046503647	0.041843466	2819	tags=24%, list=13%, signal=21%
GO:0034329	BP	GO:0034329	cell junction assembly	397	0.422558304	1.406545812	0.001954733	0.046715509	0.042034098	4494	tags=26%, list=21%, signal=21%
GO:0051051	BP	GO:0051051	negative regulation of transport	376	0.425456874	1.411798047	0.00196667	0.046922857	0.042220667	3746	tags=20%, list=17%, signal=17%
GO:0019887	MF	GO:0019887	protein kinase regulator activity	174	0.494208257	1.570682085	0.002005571	0.047771772	0.042984511	2401	tags=14%, list=11%, signal=13%
GO:0030659	CC	GO:0030659	cytoplasmic vesicle membrane	701	0.391831326	1.322415655	0.002011263	0.047828168	0.043035256	2299	tags=13%, list=11%, signal=12%
GO:0070371	BP	GO:0070371	ERK1 and ERK2 cascade	250	0.460607367	1.499210029	0.00203666	0.048236115	0.043402321	3171	tags=21%, list=15%, signal=18%
GO:1990868	BP	GO:1990868	response to chemokine	69	0.597043122	1.71791565	0.002038476	0.048236115	0.043402321	750	tags=17%, list=3%, signal=17%
GO:1990869	BP	GO:1990869	cellular response to chemokine	69	0.597043122	1.71791565	0.002038476	0.048236115	0.043402321	750	tags=17%, list=3%, signal=17%
GO:0002528	BP	GO:0002528	regulation of vascular permeability involved in acute inflammatory response	4	0.966630655	1.608812541	0.002066892	0.04874816	0.043863054	390	tags=75%, list=2%, signal=74%
GO:0070943	BP	GO:0070943	neutrophil-mediated killing of symbiont cell	4	0.966374575	1.608386334	0.002066892	0.04874816	0.043863054	387	tags=50%, list=2%, signal=49%
GO:0005179	MF	GO:0005179	hormone activity	86	0.564913209	1.673807082	0.002086146	0.049121728	0.044199187	1875	tags=36%, list=9%, signal=33%
GO:0012506	CC	GO:0012506	vesicle membrane	723	0.389032514	1.313439634	0.002110129	0.049605262	0.044634265	2727	tags=14%, list=13%, signal=13%
GO:0070486	BP	GO:0070486	leukocyte aggregation	13	0.816729826	1.755727128	0.002114165	0.049619071	0.04464669	1765	tags=54%, list=8%, signal=49%
GO:0046683	BP	GO:0046683	response to organophosphorus	119	0.528743415	1.622462285	0.002118395	0.04963737	0.044663155	2323	tags=18%, list=11%, signal=16%
GO:0045651	BP	GO:0045651	positive regulation of macrophage differentiation	12	0.831917474	1.761160588	0.002133946	0.049851696	0.044856003	2234	tags=33%, list=10%, signal=30%
GO:0006023	BP	GO:0006023	aminoglycan biosynthetic process	111	0.527388983	1.607908274	0.002134472	0.049851696	0.044856003	4006	tags=32%, list=19%, signal=27%

Supplementary Table 8

Worksheet1: Rat DEGs upregulated in alcohol exposure

Gene	logFC	logCPM	LR	PValue	FDR
ENSRNOG00000058589	2.986548599	1.638465599	214.250474	1.62E-48	2.34E-45
ENSRNOG00000009113	2.406105966	1.237920917	169.0495582	1.19E-38	1.18E-35
ENSRNOG00000022595	2.053684145	2.29732761	166.7386275	3.82E-38	3.18E-35
ENSRNOG00000047196	3.048989278	1.070855251	163.2811772	2.17E-37	1.50E-34
ENSRNOG00000017808	1.266936762	3.888126772	154.3470256	1.94E-35	1.03E-32
ENSRNOG00000056585	1.924193102	3.570167143	151.359667	8.75E-35	4.20E-32
ENSRNOG00000017427	2.209118515	1.973633839	149.1769709	2.62E-34	1.22E-31
ENSRNOG00000000488	1.600943476	3.096014623	139.4489633	3.51E-32	1.16E-29
ENSRNOG00000038184	3.143112878	1.904006471	137.9041035	7.65E-32	2.42E-29
ENSRNOG00000050401	1.866098004	3.270017365	134.6887093	3.86E-31	1.18E-28
ENSRNOG00000009040	1.575071958	3.328836032	134.4628272	4.33E-31	1.29E-28
ENSRNOG00000014322	1.030953323	4.474129242	131.6567013	1.78E-30	4.94E-28
ENSRNOG00000060518	3.278845107	6.257252348	122.3783416	1.91E-28	4.25E-26
ENSRNOG00000039876	3.637659806	1.260638462	120.6570135	4.54E-28	9.85E-26
ENSRNOG00000052025	0.99046511	6.611569698	119.9310524	6.55E-28	1.40E-25
ENSRNOG00000046500	1.452768883	2.856560374	118.063403	1.68E-27	3.37E-25
ENSRNOG00000019990	1.518575868	2.64408471	118.1023115	1.65E-27	3.37E-25
ENSRNOG00000060181	1.826837224	0.431563762	116.5647675	3.58E-27	6.82E-25
ENSRNOG00000054945	3.118677528	4.403365398	114.6823495	9.24E-27	1.70E-24
ENSRNOG00000050636	1.268582817	5.102220043	113.7902903	1.45E-26	2.64E-24
ENSRNOG00000017489	1.128719065	4.032621614	113.2445761	1.91E-26	3.43E-24
ENSRNOG00000010007	0.781642208	5.104391937	112.7029946	2.51E-26	4.46E-24
ENSRNOG00000000021	2.208553307	1.765086183	111.7120613	4.13E-26	7.19E-24
ENSRNOG00000055562	1.777956928	3.849899444	110.7083941	6.85E-26	1.13E-23
ENSRNOG00000050156	4.172788371	0.963209587	106.8216799	4.87E-25	7.49E-23
ENSRNOG00000046707	4.174169741	0.964438398	106.6063575	5.43E-25	8.19E-23
ENSRNOG00000061814	1.650899302	0.823846933	105.7909809	8.19E-25	1.21E-22
ENSRNOG00000039852	1.096319211	3.211296513	103.4943334	2.61E-24	3.73E-22
ENSRNOG00000052420	1.979083285	2.040164192	103.2274283	2.99E-24	4.22E-22
ENSRNOG00000054493	3.162527654	0.220755551	102.8197427	3.67E-24	5.05E-22
ENSRNOG00000043113	3.382754527	-0.635866632	102.6441903	4.01E-24	5.47E-22
ENSRNOG00000046560	3.343298088	0.993407907	100.9379847	9.49E-24	1.26E-21
ENSRNOG00000019692	1.717506999	3.741398231	100.0627769	1.48E-23	1.95E-21
ENSRNOG00000061240	3.238736153	-0.361048281	99.83782459	1.65E-23	2.15E-21
ENSRNOG00000005174	1.868961736	1.411815336	97.87453823	4.46E-23	5.51E-21
ENSRNOG00000055278	2.758264704	-0.196963038	97.80218867	4.62E-23	5.67E-21
ENSRNOG00000049575	1.744223401	0.665934523	97.45565087	5.51E-23	6.71E-21
ENSRNOG00000020038	1.291149959	5.339898399	96.98764754	6.98E-23	8.31E-21
ENSRNOG00000015664	1.268435955	3.6211464	96.56698772	8.63E-23	1.01E-20
ENSRNOG00000045811	1.006353281	4.794419272	95.63203515	1.38E-22	1.56E-20
ENSRNOG00000046207	1.360828897	2.801219044	95.12934076	1.78E-22	1.99E-20
ENSRNOG00000053168	1.227600718	2.638102957	94.90250993	2.00E-22	2.20E-20
ENSRNOG00000049203	1.836681695	0.502672917	94.9117198	1.99E-22	2.20E-20
ENSRNOG00000047628	0.925415734	4.56466675	94.75025015	2.16E-22	2.36E-20
ENSRNOG00000033496	0.914096038	5.78437778	94.45063956	2.51E-22	2.69E-20
ENSRNOG00000001004	1.393715027	6.177132521	93.50261347	4.06E-22	4.23E-20
ENSRNOG00000050071	0.903958308	4.917968618	93.48928355	4.08E-22	4.23E-20
ENSRNOG00000000495	1.014230587	3.902003286	93.46298416	4.14E-22	4.26E-20
ENSRNOG00000014503	1.788087801	5.865044043	93.43862269	4.19E-22	4.28E-20
ENSRNOG00000020676	1.805239662	1.862804938	91.60824771	1.06E-21	1.05E-19
ENSRNOG00000012830	0.853566507	6.018758949	91.02865411	1.42E-21	1.38E-19
ENSRNOG00000019322	0.942789929	5.264388586	90.85617631	1.55E-21	1.50E-19
ENSRNOG00000004793	1.29991659	5.243013332	90.54252958	1.81E-21	1.74E-19
ENSRNOG00000014347	1.02051763	4.123199393	90.24949786	2.10E-21	2.00E-19
ENSRNOG00000001410	0.883682202	5.153306285	89.87212917	2.54E-21	2.41E-19
ENSRNOG00000042061	1.482743632	2.381178521	89.34587458	3.31E-21	3.12E-19
ENSRNOG00000024149	3.151323031	-0.302468585	89.30259136	3.39E-21	3.17E-19
ENSRNOG00000043102	1.240807918	3.608655216	89.21760502	3.54E-21	3.29E-19
ENSRNOG00000003195	1.117812424	6.500048081	89.03341205	3.88E-21	3.57E-19
ENSRNOG00000059318	2.476486639	-0.325546252	88.62392347	4.77E-21	4.37E-19
ENSRNOG00000037672	3.929198457	-1.077273635	88.27898613	5.68E-21	5.14E-19
ENSRNOG00000010217	0.930319658	7.794276837	87.55004953	8.22E-21	7.35E-19
ENSRNOG00000055371	1.011596993	6.315311921	87.48013351	8.51E-21	7.53E-19
ENSRNOG00000042213	1.264285487	1.957527351	86.79767315	1.20E-20	1.05E-18
ENSRNOG00000001250	1.074830939	4.533898947	86.35345998	1.50E-20	1.31E-18
ENSRNOG00000055936	3.242967468	-0.417942199	85.80426019	1.99E-20	1.69E-18
ENSRNOG00000001337	0.976243194	4.240267938	85.33887857	2.51E-20	2.13E-18
ENSRNOG00000050500	0.876921135	3.65296066	85.3145803	2.54E-20	2.14E-18
ENSRNOG00000026840	1.060794338	3.068080707	85.16880687	2.74E-20	2.30E-18
ENSRNOG00000001211	0.974599356	5.274658161	84.74367446	3.40E-20	2.80E-18
ENSRNOG00000024482	1.093384219	5.571938889	84.46549531	3.91E-20	3.19E-18
ENSRNOG00000007604	0.844697538	5.340087179	84.21688147	4.43E-20	3.60E-18
ENSRNOG00000010868	1.017783025	2.955565574	83.66126482	5.87E-20	4.63E-18
ENSRNOG00000001864	2.007375376	1.211276069	83.6683887	5.85E-20	4.63E-18
ENSRNOG00000012764	0.953695217	6.558958285	82.86632445	8.78E-20	6.78E-18
ENSRNOG00000054489	2.712538392	2.446408601	82.52600033	1.04E-19	7.98E-18
ENSRNOG00000023781	1.056146508	6.544070065	82.51558748	1.05E-19	7.98E-18
ENSRNOG00000020947	1.035348885	5.296463495	82.36816645	1.13E-19	8.56E-18
ENSRNOG00000028956	1.047148421	5.003797217	82.09697407	1.30E-19	9.77E-18
ENSRNOG00000029510	0.805155618	6.461207735	81.93943602	1.40E-19	1.05E-17
ENSRNOG00000001888	1.043820645	4.352118306	81.50705941	1.75E-19	1.29E-17
ENSRNOG00000004346	1.131815289	4.056271844	81.07340118	2.17E-19	1.58E-17
ENSRNOG00000034031	1.357979945	4.135530414	81.01138221	2.24E-19	1.62E-17
ENSRNOG00000051720	1.287857901	2.987369914	80.9281055	2.34E-19	1.68E-17
ENSRNOG00000048004	1.340754546	2.701136265	80.90863642	2.36E-19	1.69E-17
ENSRNOG00000001156	0.718974208	4.865230842	79.7969678	4.15E-19	2.87E-17
ENSRNOG00000001214	1.123652135	5.509797765	79.48928389	4.85E-19	3.32E-17
ENSRNOG00000046313	1.565455849	4.857016803	79.38469548	5.11E-19	3.49E-17
ENSRNOG00000008052	1.165765886	6.655151815	79.37428882	5.14E-19	3.49E-17
ENSRNOG00000033791	0.863244975	6.55275502	79.27781401	5.40E-19	3.64E-17
ENSRNOG00000010237	0.69691385	6.396128089	79.2656695	5.43E-19	3.64E-17
ENSRNOG00000015753	0.884856257	6.718781196	79.11645174	5.86E-19	3.91E-17
ENSRNOG00000002262	0.675715488	5.219515206	79.03616519	6.10E-19	4.06E-17
ENSRNOG00000018553	1.045736474	7.328833761	78.88820943	6.57E-19	4.34E-17
ENSRNOG00000042062	1.5862659	0.923279094	78.01661265	1.02E-18	6.69E-17
ENSRNOG00000033202	1.457123398	4.041952894	78.00046383	1.03E-18	6.71E-17
ENSRNOG00000031475	1.442590432	3.804148162	77.96462354	1.05E-18	6.81E-17
ENSRNOG00000026243	0.739371492	6.756204836	77.8942826	1.09E-18	7.03E-17
ENSRNOG00000013927	1.398181587	4.344544521	77.61055455	1.25E-18	8.01E-17

ENSRNOG00000057881	1.245508137	4.363267153	77.36690884	1.42E-18	9.01E-17
ENSRNOG00000001198	1.958684809	1.235954831	77.36313849	1.42E-18	9.01E-17
ENSRNOG00000001377	0.928186535	3.972313414	77.3297601	1.45E-18	9.13E-17
ENSRNOG00000020134	0.817743929	5.555462566	77.29406998	1.47E-18	9.26E-17
ENSRNOG00000052687	0.67928381	7.232446744	76.81716104	1.88E-18	1.16E-16
ENSRNOG00000006359	1.70072593	2.292053898	76.80374134	1.89E-18	1.16E-16
ENSRNOG00000020383	0.8378325	3.510105465	76.54986375	2.15E-18	1.32E-16
ENSRNOG00000000033	0.982717684	6.696416699	76.40672274	2.31E-18	1.41E-16
ENSRNOG00000018556	0.920234031	4.634899018	76.31956703	2.41E-18	1.46E-16
ENSRNOG00000014205	2.705536976	0.517394502	75.98748259	2.85E-18	1.73E-16
ENSRNOG00000004392	1.169233496	6.648067592	75.64119808	3.40E-18	2.05E-16
ENSRNOG00000024879	1.141607044	3.48560522	75.60408831	3.47E-18	2.08E-16
ENSRNOG00000019698	1.38195209	5.065171569	75.56245269	3.54E-18	2.12E-16
ENSRNOG00000055226	0.78517052	6.005361251	75.53692246	3.59E-18	2.13E-16
ENSRNOG00000004956	0.781354588	4.927799625	75.35136067	3.94E-18	2.34E-16
ENSRNOG00000020238	1.061073431	2.10758271	75.31889695	4.01E-18	2.37E-16
ENSRNOG00000028648	1.130214429	6.015663728	75.08948459	4.50E-18	2.62E-16
ENSRNOG00000030630	0.987924548	6.618231269	75.07937937	4.52E-18	2.62E-16
ENSRNOG00000001352	0.670949706	7.304603122	74.91544546	4.91E-18	2.84E-16
ENSRNOG00000056656	1.530475343	0.918351783	74.87390769	5.02E-18	2.89E-16
ENSRNOG00000008377	0.942295415	5.934739107	74.77726772	5.27E-18	3.01E-16
ENSRNOG00000059408	1.266447408	4.815839902	74.61646223	5.72E-18	3.23E-16
ENSRNOG00000049484	1.047468075	7.355608012	74.37029483	6.48E-18	3.64E-16
ENSRNOG00000016684	0.937945725	6.135841973	74.06096963	7.57E-18	4.22E-16
ENSRNOG00000015430	0.618592566	6.999470933	73.83224512	8.50E-18	4.72E-16
ENSRNOG00000032574	0.888328532	5.620665433	73.80946184	8.60E-18	4.76E-16
ENSRNOG00000056637	0.893891321	4.906001463	73.7785131	8.74E-18	4.82E-16
ENSRNOG00000055583	1.078145649	6.805948144	73.5194868	9.96E-18	5.44E-16
ENSRNOG00000000463	1.413318582	5.48332567	73.31272437	1.11E-17	6.00E-16
ENSRNOG00000053452	1.582054976	4.946956065	73.23728106	1.15E-17	6.19E-16
ENSRNOG00000016257	0.870494082	5.167578916	73.07689946	1.25E-17	6.69E-16
ENSRNOG00000021871	0.841189843	5.965831779	73.05431915	1.26E-17	6.75E-16
ENSRNOG00000019869	1.702882005	2.934155106	72.99419485	1.30E-17	6.93E-16
ENSRNOG00000000867	0.837173947	4.657676	72.35359256	1.80E-17	9.49E-16
ENSRNOG00000004757	3.46193224	0.981466033	72.32951787	1.82E-17	9.58E-16
ENSRNOG00000019958	1.67311383	3.644587725	72.26771841	1.88E-17	9.85E-16
ENSRNOG00000014984	0.767176749	5.136218857	71.89494363	2.27E-17	1.18E-15
ENSRNOG00000057588	1.97407503	0.764640598	71.48861555	2.79E-17	1.44E-15
ENSRNOG00000009298	0.818007356	6.088411047	71.26913347	3.12E-17	1.60E-15
ENSRNOG00000009140	1.974517835	3.03839889	71.07691029	3.44E-17	1.75E-15
ENSRNOG00000017564	1.156880036	5.750383504	70.97637051	3.62E-17	1.84E-15
ENSRNOG00000049270	1.468262689	2.240021347	70.91859593	3.72E-17	1.89E-15
ENSRNOG00000029095	0.840326511	3.797770553	70.8646527	3.83E-17	1.94E-15
ENSRNOG00000020590	0.707423425	4.982200839	70.73620055	4.08E-17	2.06E-15
ENSRNOG00000000470	0.722863224	5.841530449	70.66731633	4.23E-17	2.13E-15
ENSRNOG00000042211	1.616819449	1.994877581	70.53257791	4.53E-17	2.26E-15
ENSRNOG00000012184	0.77685969	5.317818611	70.4829177	4.64E-17	2.31E-15
ENSRNOG00000010119	0.704608044	3.845104942	70.11988187	5.58E-17	2.77E-15
ENSRNOG00000020266	0.882213326	9.566895413	70.03672563	5.82E-17	2.88E-15
ENSRNOG00000016655	0.995863499	4.473126321	69.91201854	6.20E-17	3.06E-15
ENSRNOG00000058359	1.331919926	7.078349349	69.79023629	6.60E-17	3.23E-15
ENSRNOG00000019423	0.975226774	5.810559643	69.64445658	7.10E-17	3.47E-15
ENSRNOG00000005900	0.981641255	3.362215482	69.6106935	7.22E-17	3.51E-15
ENSRNOG00000008392	0.763985529	7.020485477	69.61127808	7.22E-17	3.51E-15
ENSRNOG00000001237	0.762101075	5.393430914	69.57646617	7.35E-17	3.55E-15
ENSRNOG00000018798	0.815877818	7.196044779	69.54910778	7.45E-17	3.59E-15
ENSRNOG00000004505	1.473397337	3.118822365	69.37175118	8.15E-17	3.89E-15
ENSRNOG00000032238	0.729053702	3.292877348	69.10769989	9.32E-17	4.41E-15
ENSRNOG00000018529	1.103779901	5.650549562	68.95214154	1.01E-16	4.74E-15
ENSRNOG00000053502	0.986598874	5.945224204	68.90036667	1.04E-16	4.85E-15
ENSRNOG00000053582	1.807285085	1.022151295	68.74335278	1.12E-16	5.24E-15
ENSRNOG00000009028	1.346509283	3.945281688	68.62471768	1.19E-16	5.53E-15
ENSRNOG00000017692	0.966112895	5.652383704	68.57843414	1.22E-16	5.65E-15
ENSRNOG00000023521	1.21734387	5.783369295	68.56330056	1.23E-16	5.67E-15
ENSRNOG00000045775	0.955826831	4.173895204	68.2743534	1.42E-16	6.53E-15
ENSRNOG00000055984	0.738887596	5.023037739	68.19548525	1.48E-16	6.76E-15
ENSRNOG00000012434	0.631671499	4.800872862	67.99516649	1.64E-16	7.41E-15
ENSRNOG00000021794	0.978341847	2.363394522	67.94321791	1.68E-16	7.59E-15
ENSRNOG00000060572	1.103420489	1.88897274	67.83283406	1.78E-16	8.01E-15
ENSRNOG00000021048	1.075900628	4.705336512	67.77250218	1.83E-16	8.21E-15
ENSRNOG00000001348	1.052822434	4.671580098	67.5916621	2.01E-16	8.95E-15
ENSRNOG00000032635	1.669550133	2.795970846	67.10728542	2.57E-16	1.14E-14
ENSRNOG00000033426	0.720595901	6.390087519	67.07058877	2.62E-16	1.16E-14
ENSRNOG00000042855	1.036369319	5.054350968	66.8635503	2.91E-16	1.27E-14
ENSRNOG00000001452	0.83779044	2.837088736	66.75031459	3.08E-16	1.34E-14
ENSRNOG00000003948	0.781128508	5.571900601	66.70253649	3.16E-16	1.37E-14
ENSRNOG00000000054	1.288328727	6.457326253	66.61714733	3.30E-16	1.43E-14
ENSRNOG00000010763	0.940291768	2.909323244	66.43121868	3.62E-16	1.56E-14
ENSRNOG00000014480	1.121567856	3.387123247	66.29529533	3.88E-16	1.66E-14
ENSRNOG00000006049	1.046738537	3.944085194	66.24931854	3.97E-16	1.69E-14
ENSRNOG00000021147	0.714199137	4.144486976	66.11324481	4.26E-16	1.81E-14
ENSRNOG00000007934	1.133551081	5.21920722	66.03717184	4.42E-16	1.87E-14
ENSRNOG00000020014	1.056694139	4.537089005	65.78009842	5.04E-16	2.11E-14
ENSRNOG00000049221	0.729492573	5.700261262	65.55558886	5.65E-16	2.35E-14
ENSRNOG00000020161	0.848046646	4.675171332	65.07747645	7.20E-16	2.97E-14
ENSRNOG00000016818	0.87102679	6.297071835	65.03598271	7.35E-16	3.02E-14
ENSRNOG00000018170	0.801425923	5.815713039	64.88277072	7.95E-16	3.26E-14
ENSRNOG00000045636	0.744137515	7.775596803	64.65022676	8.94E-16	3.65E-14
ENSRNOG00000013850	2.085957903	1.498614164	64.55160148	9.40E-16	3.82E-14
ENSRNOG00000018841	0.680587085	4.589893684	64.54391603	9.44E-16	3.82E-14
ENSRNOG00000019977	0.734112135	6.744546638	64.03931263	1.22E-15	4.90E-14
ENSRNOG00000041648	3.626215657	-1.041706644	63.99701716	1.25E-15	5.00E-14
ENSRNOG00000019459	1.06098907	6.924033377	63.9608004	1.27E-15	5.06E-14
ENSRNOG00000024066	0.623261299	3.706601761	63.86011587	1.34E-15	5.31E-14
ENSRNOG00000001094	1.074546114	1.882591291	63.84656971	1.34E-15	5.34E-14
ENSRNOG00000055994	2.18458252	-0.462650259	63.6445917	1.49E-15	5.90E-14
ENSRNOG00000047768	0.856676573	4.568823988	63.60425781	1.52E-15	6.01E-14
ENSRNOG00000020363	0.944311276	5.037505685	63.57947861	1.54E-15	6.05E-14
ENSRNOG00000046598	2.173847927	-0.470640478	63.50730723	1.60E-15	6.26E-14
ENSRNOG00000048242	0.566423598	4.652787591	63.16783781	1.90E-15	7.40E-14

ENSRNOG00000019935	0.873647703	4.183378837	63.04649784	2.02E-15	7.83E-14
ENSRNOG00000021962	1.607481028	2.234442044	63.04315407	2.02E-15	7.83E-14
ENSRNOG00000011007	0.604327822	6.615939297	62.97887158	2.09E-15	8.07E-14
ENSRNOG00000020142	0.99569335	5.086379702	62.84480755	2.24E-15	8.55E-14
ENSRNOG00000062030	0.99569335	5.086379702	62.84480755	2.24E-15	8.55E-14
ENSRNOG00000020239	1.058060934	4.581366301	62.6669129	2.45E-15	9.32E-14
ENSRNOG00000020249	0.592551921	5.322737103	62.45659053	2.72E-15	1.03E-13
ENSRNOG00000018781	1.012416392	4.306088661	62.46103964	2.72E-15	1.03E-13
ENSRNOG00000062223	1.785005774	0.630001195	62.41247175	2.79E-15	1.05E-13
ENSRNOG00000024019	0.821574771	3.271586037	62.1544866	3.18E-15	1.18E-13
ENSRNOG00000024243	0.925046904	6.700538647	62.12040042	3.23E-15	1.20E-13
ENSRNOG00000006327	0.896102252	4.637145086	62.1073876	3.25E-15	1.21E-13
ENSRNOG00000001010	0.783286609	6.005346986	61.8638227	3.68E-15	1.36E-13
ENSRNOG00000004310	0.96116404	4.606455629	61.69490925	4.01E-15	1.48E-13
ENSRNOG00000020839	0.946733354	2.438244099	61.67218655	4.06E-15	1.49E-13
ENSRNOG00000046176	3.159333968	-1.289900007	61.23293352	5.07E-15	1.86E-13
ENSRNOG00000008145	0.803306055	5.708659015	60.99888227	5.71E-15	2.09E-13
ENSRNOG00000053240	1.162289175	4.018927138	60.81373882	6.27E-15	2.28E-13
ENSRNOG00000019143	0.631982839	6.104173046	60.80051444	6.32E-15	2.29E-13
ENSRNOG00000001129	0.613595217	4.923108679	60.77778192	6.39E-15	2.31E-13
ENSRNOG00000047247	0.810995086	7.815418663	60.64990992	6.82E-15	2.46E-13
ENSRNOG00000006952	0.761889489	6.581173773	60.54129913	7.21E-15	2.59E-13
ENSRNOG00000020948	1.092748115	1.744042989	60.50146546	7.35E-15	2.63E-13
ENSRNOG00000004168	0.91355499	4.038467688	59.94351033	9.76E-15	3.44E-13
ENSRNOG00000020828	0.7416699	4.508252019	59.69286723	1.11E-14	3.88E-13
ENSRNOG00000017748	1.844912783	1.541713523	59.65217499	1.13E-14	3.95E-13
ENSRNOG00000021725	0.49360612	6.119550885	59.52693247	1.21E-14	4.20E-13
ENSRNOG00000002516	0.639337522	4.794863947	59.44762363	1.26E-14	4.36E-13
ENSRNOG00000058662	0.81030059	4.9432585	59.41558498	1.28E-14	4.42E-13
ENSRNOG00000037091	2.717502753	-0.945261753	59.39621261	1.29E-14	4.46E-13
ENSRNOG00000019382	1.031374986	4.660089523	59.34408373	1.32E-14	4.57E-13
ENSRNOG00000054421	2.526942912	-1.164041788	59.09832288	1.50E-14	5.15E-13
ENSRNOG00000001091	1.0103013	5.197283298	58.96187684	1.61E-14	5.50E-13
ENSRNOG00000028350	1.457075904	2.450588232	58.92789302	1.64E-14	5.58E-13
ENSRNOG00000007118	0.994009438	2.623706951	58.91061563	1.65E-14	5.62E-13
ENSRNOG00000021526	1.200659545	2.482172437	58.5343605	2.00E-14	6.74E-13
ENSRNOG00000028323	1.410598073	3.203534787	58.52795914	2.00E-14	6.75E-13
ENSRNOG00000036441	2.600202294	0.7744596	58.44210761	2.09E-14	7.01E-13
ENSRNOG00000002556	1.149461656	2.181674672	58.43083386	2.11E-14	7.03E-13
ENSRNOG00000020431	0.900432604	6.135021111	58.4202484	2.12E-14	7.06E-13
ENSRNOG00000026474	0.703136354	4.21423817	58.33287131	2.21E-14	7.35E-13
ENSRNOG00000060161	2.839533364	-0.666577068	58.11536262	2.47E-14	8.17E-13
ENSRNOG00000039120	1.853356448	0.152185722	58.08938938	2.50E-14	8.25E-13
ENSRNOG00000056946	1.042500658	6.245346786	57.98357037	2.64E-14	8.65E-13
ENSRNOG00000003203	0.615778108	5.072099163	57.98706437	2.64E-14	8.65E-13
ENSRNOG00000056148	1.194544785	3.459632341	57.72598281	3.01E-14	9.80E-13
ENSRNOG00000000175	1.034207634	3.907569266	57.61128527	3.19E-14	1.03E-12
ENSRNOG00000024177	2.706069365	-0.714418267	57.60362642	3.21E-14	1.04E-12
ENSRNOG00000050780	0.918349825	2.216260252	57.57045139	3.26E-14	1.05E-12
ENSRNOG00000000421	0.939359364	4.353325487	57.55784279	3.28E-14	1.06E-12
ENSRNOG00000022368	0.753143007	3.99681609	57.38222529	3.59E-14	1.15E-12
ENSRNOG00000010769	2.04033856	0.122288332	57.24251867	3.85E-14	1.23E-12
ENSRNOG00000007597	1.497152176	2.527826755	57.16026666	4.02E-14	1.28E-12
ENSRNOG00000018212	1.419853853	4.924445911	57.11362588	4.11E-14	1.31E-12
ENSRNOG00000055082	0.713864573	4.917628071	57.0546179	4.24E-14	1.34E-12
ENSRNOG00000021103	0.915110975	7.138926139	57.02726051	4.30E-14	1.36E-12
ENSRNOG00000024349	1.4649729	7.046657483	57.01645588	4.32E-14	1.36E-12
ENSRNOG00000020878	0.533890382	5.236350396	57.002197	4.35E-14	1.37E-12
ENSRNOG00000020905	0.846818732	6.146700306	56.90924589	4.56E-14	1.43E-12
ENSRNOG00000038150	1.780798813	0.659813477	56.89404335	4.60E-14	1.44E-12
ENSRNOG00000020138	0.827025682	6.329718012	56.83305751	4.74E-14	1.48E-12
ENSRNOG00000013545	0.999933396	4.779259711	56.67867238	5.13E-14	1.59E-12
ENSRNOG00000032908	0.877312007	3.39266751	56.63791557	5.24E-14	1.62E-12
ENSRNOG00000019476	0.502660754	6.920959756	56.59650538	5.35E-14	1.65E-12
ENSRNOG00000008051	0.701190202	4.661620447	56.4620319	5.73E-14	1.76E-12
ENSRNOG00000037768	1.149862675	1.096734374	56.37931934	5.98E-14	1.83E-12
ENSRNOG00000000417	0.735400043	6.295498463	56.28849992	6.26E-14	1.92E-12
ENSRNOG00000049437	0.811075275	5.930787865	56.20057066	6.54E-14	2.00E-12
ENSRNOG00000042216	1.495799365	1.025020386	56.14885845	6.72E-14	2.05E-12
ENSRNOG00000009625	0.622075694	8.044541328	56.06157295	7.02E-14	2.14E-12
ENSRNOG00000042274	0.796210177	4.466186528	56.0599754	7.03E-14	2.14E-12
ENSRNOG00000014375	1.263157726	7.102218771	56.0300047	7.14E-14	2.16E-12
ENSRNOG00000015450	0.868953071	4.795660897	55.80093976	8.02E-14	2.43E-12
ENSRNOG00000053306	0.71809514	3.732153189	55.72096118	8.35E-14	2.52E-12
ENSRNOG00000033101	0.712541074	6.887845958	55.69565286	8.46E-14	2.55E-12
ENSRNOG00000056955	0.72247573	5.142554357	55.66566973	8.59E-14	2.59E-12
ENSRNOG00000052129	0.84630134	5.426812004	55.34567693	1.01E-13	3.02E-12
ENSRNOG00000019014	0.672010368	5.307433944	55.17695837	1.10E-13	3.28E-12
ENSRNOG00000017208	0.871362595	4.613180381	55.07304227	1.16E-13	3.44E-12
ENSRNOG00000028261	0.917941239	5.89159932	55.0452381	1.18E-13	3.49E-12
ENSRNOG00000032429	1.090350162	1.16381064	54.99083807	1.21E-13	3.58E-12
ENSRNOG00000032705	0.71615468	4.308476705	54.95567759	1.23E-13	3.64E-12
ENSRNOG00000010872	1.135240187	8.529572758	54.58130007	1.49E-13	4.35E-12
ENSRNOG00000029280	0.801906982	4.368517205	54.57940868	1.49E-13	4.35E-12
ENSRNOG00000019638	1.114025513	4.151160919	54.55791368	1.51E-13	4.39E-12
ENSRNOG00000060010	0.723101097	3.180271904	54.54118349	1.52E-13	4.42E-12
ENSRNOG00000009446	0.963491925	3.827367677	54.53739765	1.53E-13	4.42E-12
ENSRNOG00000056298	1.946103224	-0.08825214	54.52771065	1.53E-13	4.44E-12
ENSRNOG00000047706	1.886340456	2.632624817	54.4176518	1.62E-13	4.68E-12
ENSRNOG00000000474	0.746526332	4.367638501	54.39389365	1.64E-13	4.72E-12
ENSRNOG00000056617	1.517695359	0.705194953	54.38912132	1.64E-13	4.73E-12
ENSRNOG00000017621	0.714215807	5.525657162	54.34453834	1.68E-13	4.83E-12
ENSRNOG00000016281	0.838360679	4.813441217	54.33506583	1.69E-13	4.84E-12
ENSRNOG00000017510	0.659426439	6.825071723	54.119964	1.89E-13	5.32E-12
ENSRNOG00000000498	0.912160543	4.616596964	54.12071349	1.89E-13	5.32E-12
ENSRNOG00000003099	0.622950482	7.069658459	54.06911479	1.94E-13	5.45E-12
ENSRNOG00000042720	0.705666965	4.034585779	54.05521824	1.95E-13	5.48E-12
ENSRNOG00000049683	0.692632948	4.655751482	53.96992768	2.04E-13	5.72E-12
ENSRNOG00000049828	1.199659428	1.974046404	53.89073982	2.12E-13	5.94E-12
ENSRNOG00000007334	1.094138679	5.874809473	53.7208372	2.31E-13	6.47E-12

ENSRNOG00000039976	1.232221235	2.441129799	53.57467446	2.49E-13	6.92E-12
ENSRNOG00000060350	0.624150057	6.963747221	53.40892789	2.71E-13	7.47E-12
ENSRNOG00000004351	0.75491812	3.22026026	53.31760597	2.84E-13	7.80E-12
ENSRNOG00000010436	1.083624895	3.86985707	53.28227657	2.89E-13	7.91E-12
ENSRNOG00000042728	1.043388369	3.380986272	52.78371933	3.72E-13	1.01E-11
ENSRNOG00000003821	0.482514814	5.795877295	52.69578799	3.89E-13	1.06E-11
ENSRNOG00000045843	0.990382904	5.988825608	52.61444244	4.06E-13	1.10E-11
ENSRNOG00000003463	0.863007902	5.656259925	52.57830535	4.13E-13	1.12E-11
ENSRNOG00000059659	0.509664207	5.615489164	52.54938822	4.20E-13	1.13E-11
ENSRNOG00000040384	1.131950247	4.499470313	52.42861084	4.46E-13	1.20E-11
ENSRNOG00000051499	0.945975059	3.234253498	52.41065948	4.50E-13	1.20E-11
ENSRNOG00000032554	1.681479359	-0.114546243	52.39569833	4.54E-13	1.21E-11
ENSRNOG00000007818	0.72959048	3.441981031	52.37265057	4.59E-13	1.22E-11
ENSRNOG00000012164	0.689742592	6.308778018	52.18439467	5.05E-13	1.34E-11
ENSRNOG00000028208	0.793778279	3.273420753	52.1627176	5.11E-13	1.35E-11
ENSRNOG00000016892	1.112751756	3.126131019	52.1623215	5.11E-13	1.35E-11
ENSRNOG00000054978	0.780172894	3.756081997	52.16278322	5.11E-13	1.35E-11
ENSRNOG00000015335	0.73577823	6.299117455	52.14762405	5.15E-13	1.36E-11
ENSRNOG00000020130	0.807702263	4.724907616	52.11705855	5.23E-13	1.38E-11
ENSRNOG00000027489	1.44540236	3.808003766	52.08880527	5.30E-13	1.39E-11
ENSRNOG00000039417	0.559521797	5.022211737	52.01805318	5.50E-13	1.44E-11
ENSRNOG00000016427	0.472140275	5.733211504	51.93418387	5.74E-13	1.50E-11
ENSRNOG00000011475	1.04738782	5.700521225	51.86675013	5.94E-13	1.55E-11
ENSRNOG00000014666	1.173390886	2.396594312	51.80155894	6.14E-13	1.60E-11
ENSRNOG00000028187	2.958823223	-0.654851035	51.74762197	6.31E-13	1.64E-11
ENSRNOG00000018932	1.017481635	4.359634073	51.71137529	6.43E-13	1.66E-11
ENSRNOG00000055809	1.337397887	0.874620417	51.65240412	6.62E-13	1.71E-11
ENSRNOG00000016422	1.022775076	2.724189164	51.54396925	7.00E-13	1.80E-11
ENSRNOG00000016274	1.475786635	0.81078892	51.5217459	7.08E-13	1.82E-11
ENSRNOG00000021032	0.823286374	4.215428565	51.44225551	7.37E-13	1.89E-11
ENSRNOG00000009675	0.547645003	6.799744585	51.29333218	7.95E-13	2.03E-11
ENSRNOG00000033688	0.777489452	7.174379918	51.26439706	8.07E-13	2.06E-11
ENSRNOG00000011544	0.932258252	4.324555384	51.20678344	8.31E-13	2.12E-11
ENSRNOG00000027569	0.683338029	5.248395628	50.86547823	9.89E-13	2.49E-11
ENSRNOG00000004430	1.042000069	4.397746561	50.82743407	1.01E-12	2.54E-11
ENSRNOG00000027582	0.56991126	5.489601849	50.72957177	1.06E-12	2.66E-11
ENSRNOG00000019924	0.760596405	4.053903044	50.69880566	1.08E-12	2.70E-11
ENSRNOG00000025053	0.710335364	9.048122893	50.69548266	1.08E-12	2.70E-11
ENSRNOG00000054126	1.345543014	1.142901119	50.53037955	1.17E-12	2.91E-11
ENSRNOG00000026293	0.757875468	4.272205569	50.52333888	1.18E-12	2.92E-11
ENSRNOG00000004711	0.476357493	5.480965659	50.43626602	1.23E-12	3.04E-11
ENSRNOG00000008224	0.834225871	3.528113238	50.3718099	1.27E-12	3.13E-11
ENSRNOG00000015865	0.878608991	5.428309329	50.3662626	1.28E-12	3.14E-11
ENSRNOG00000059683	0.698830197	5.794845147	50.35167027	1.29E-12	3.16E-11
ENSRNOG00000039858	1.112536841	3.45112005	50.22109549	1.37E-12	3.36E-11
ENSRNOG00000053828	0.701601722	6.277012989	50.16453542	1.41E-12	3.45E-11
ENSRNOG00000018557	0.963960764	3.742354852	50.09206585	1.47E-12	3.57E-11
ENSRNOG00000005446	0.582639666	4.965461567	49.89076814	1.63E-12	3.94E-11
ENSRNOG00000055241	2.510186524	-0.70872982	49.85745778	1.65E-12	3.99E-11
ENSRNOG00000022171	0.76050856	3.481774795	49.73778676	1.76E-12	4.23E-11
ENSRNOG00000017342	0.548520198	4.70573135	49.68117282	1.81E-12	4.35E-11
ENSRNOG00000015434	0.802858174	4.855123513	49.61241067	1.87E-12	4.49E-11
ENSRNOG00000049496	1.163469765	1.602746257	49.6050638	1.88E-12	4.50E-11
ENSRNOG00000004131	0.757692172	4.938716668	49.54529565	1.94E-12	4.63E-11
ENSRNOG00000007112	2.484128061	0.686410439	49.15318278	2.37E-12	5.63E-11
ENSRNOG00000053691	1.12264285	3.260474377	49.12700378	2.40E-12	5.70E-11
ENSRNOG00000017916	0.877794278	4.790897991	49.10335076	2.43E-12	5.76E-11
ENSRNOG00000012357	0.650615989	5.101021812	49.06890061	2.47E-12	5.84E-11
ENSRNOG00000018840	0.658245712	4.771078581	49.06676656	2.47E-12	5.84E-11
ENSRNOG00000004259	1.141643376	3.90034076	49.06483244	2.48E-12	5.84E-11
ENSRNOG00000037957	0.563587967	5.272112815	49.05736135	2.49E-12	5.86E-11
ENSRNOG00000015080	0.489638566	4.464071937	49.00621663	2.55E-12	6.00E-11
ENSRNOG00000018788	1.027435644	5.430442773	48.99989881	2.56E-12	6.01E-11
ENSRNOG00000010048	0.731330889	8.008237312	48.97391549	2.59E-12	6.08E-11
ENSRNOG00000020466	1.014269264	2.689403018	48.97236979	2.60E-12	6.08E-11
ENSRNOG00000057235	0.889144988	1.883079183	48.91126343	2.68E-12	6.26E-11
ENSRNOG00000042410	2.813113762	-0.965942956	48.87853859	2.72E-12	6.35E-11
ENSRNOG00000009872	0.901324315	4.881415061	48.76173805	2.89E-12	6.73E-11
ENSRNOG00000016748	0.58566361	4.610053383	48.67637465	3.02E-12	7.02E-11
ENSRNOG00000019418	0.90821619	6.416824473	48.64857098	3.06E-12	7.11E-11
ENSRNOG00000018649	0.662216639	4.337867582	48.43741506	3.41E-12	7.89E-11
ENSRNOG00000020372	0.660300308	4.557694273	48.42211324	3.44E-12	7.94E-11
ENSRNOG00000061544	0.792595579	7.375448024	48.36591053	3.54E-12	8.15E-11
ENSRNOG00000021891	0.998274349	4.583362027	48.32285317	3.62E-12	8.31E-11
ENSRNOG00000008761	0.535312516	7.868785707	48.18258481	3.88E-12	8.89E-11
ENSRNOG00000001769	1.127813828	7.035978559	47.94508826	4.38E-12	9.97E-11
ENSRNOG00000020345	0.569289625	4.904421412	47.92951643	4.42E-12	1.00E-10
ENSRNOG00000031041	1.469670599	0.110630972	47.89871076	4.49E-12	1.02E-10
ENSRNOG00000005428	0.638937258	7.054248933	47.78493628	4.76E-12	1.07E-10
ENSRNOG00000018372	0.775716277	5.565339445	47.73285015	4.88E-12	1.10E-10
ENSRNOG00000024786	0.722652748	3.871073019	47.72213997	4.91E-12	1.10E-10
ENSRNOG00000046320	1.571982092	-0.052285238	47.53487517	5.40E-12	1.21E-10
ENSRNOG00000041730	2.173419146	-0.198962783	47.32273228	6.02E-12	1.34E-10
ENSRNOG00000013248	0.499209576	5.303618524	47.29608616	6.10E-12	1.36E-10
ENSRNOG00000017506	0.657860005	6.007168754	47.22904009	6.32E-12	1.40E-10
ENSRNOG00000020742	0.730028328	5.570269286	47.12397463	6.66E-12	1.47E-10
ENSRNOG00000003494	0.512987748	6.062562589	47.09661863	6.76E-12	1.49E-10
ENSRNOG00000008960	1.149925585	1.655712368	47.05365709	6.91E-12	1.52E-10
ENSRNOG00000044405	1.920659011	-0.609821114	46.9981583	7.11E-12	1.56E-10
ENSRNOG00000024867	0.813672886	3.909171873	46.9931157	7.12E-12	1.56E-10
ENSRNOG00000056162	0.68626432	5.796030819	46.96465609	7.23E-12	1.59E-10
ENSRNOG00000019964	0.451258471	6.933212123	46.94921817	7.29E-12	1.60E-10
ENSRNOG00000003864	0.689682604	4.493882989	46.83619461	7.72E-12	1.68E-10
ENSRNOG00000012379	0.836341811	4.255231568	46.79043549	7.90E-12	1.72E-10
ENSRNOG00000009409	1.119950535	4.92069826	46.77640764	7.96E-12	1.73E-10
ENSRNOG00000002294	0.55852559	3.788685994	46.65268084	8.48E-12	1.84E-10
ENSRNOG00000016630	0.603460183	5.636053525	46.62570192	8.59E-12	1.86E-10
ENSRNOG00000033473	0.980206868	3.969405096	46.56383916	8.87E-12	1.92E-10
ENSRNOG00000014087	1.012727767	4.953320232	46.51470993	9.09E-12	1.96E-10
ENSRNOG00000016315	0.547423341	4.926254984	46.40676546	9.61E-12	2.06E-10

ENSRNOG00000049236	1.609838106	0.473907179	46.39090989	9.69E-12	2.08E-10
ENSRNOG00000057470	0.637822606	3.38027916	46.31049639	1.01E-11	2.16E-10
ENSRNOG00000013895	0.685562973	6.382294688	46.11716625	1.11E-11	2.38E-10
ENSRNOG00000014477	1.200425533	1.282599702	46.09037135	1.13E-11	2.41E-10
ENSRNOG00000019356	1.059194827	3.496654147	45.86999828	1.26E-11	2.68E-10
ENSRNOG00000001177	1.073820661	3.033246814	45.86137947	1.27E-11	2.69E-10
ENSRNOG00000021106	0.633220943	5.760332873	45.79790618	1.31E-11	2.77E-10
ENSRNOG00000000842	0.859121678	4.14914442	45.76727967	1.33E-11	2.81E-10
ENSRNOG00000026124	1.13119281	3.589069642	45.74722805	1.35E-11	2.84E-10
ENSRNOG00000046202	1.277240225	0.36645641	45.67892356	1.39E-11	2.93E-10
ENSRNOG00000051719	0.916389784	3.976470101	45.61388726	1.44E-11	3.02E-10
ENSRNOG00000048929	1.08923948	1.010633419	45.46671012	1.55E-11	3.25E-10
ENSRNOG00000022615	1.087762837	2.041446507	45.42265101	1.59E-11	3.31E-10
ENSRNOG00000010067	0.84760307	4.826047111	45.41501927	1.59E-11	3.32E-10
ENSRNOG00000050748	0.510803554	5.086833294	45.40346125	1.60E-11	3.33E-10
ENSRNOG00000014287	0.848317634	6.416141292	45.3574837	1.64E-11	3.41E-10
ENSRNOG00000003923	0.64817151	5.881716668	45.2933616	1.70E-11	3.51E-10
ENSRNOG00000020996	0.796076745	4.332378818	45.20256569	1.78E-11	3.67E-10
ENSRNOG00000002894	0.554524978	4.847450931	45.19178717	1.79E-11	3.68E-10
ENSRNOG00000020607	0.816366182	4.815896519	45.16233524	1.81E-11	3.73E-10
ENSRNOG00000009271	0.47967964	6.847350858	45.11451417	1.86E-11	3.81E-10
ENSRNOG00000004141	0.841433753	2.59945938	45.00740508	1.96E-11	4.02E-10
ENSRNOG00000052795	0.901500562	2.689250069	45.00295516	1.97E-11	4.02E-10
ENSRNOG00000011431	0.60200838	4.493468111	44.99434828	1.98E-11	4.03E-10
ENSRNOG00000045545	2.023134878	-0.988668234	44.96910491	2.00E-11	4.08E-10
ENSRNOG00000007520	1.014548422	1.393850621	44.96385628	2.01E-11	4.08E-10
ENSRNOG00000014204	0.850432061	3.881783302	44.80891443	2.17E-11	4.40E-10
ENSRNOG00000049093	1.025090085	3.672488604	44.78678521	2.20E-11	4.45E-10
ENSRNOG00000059770	1.672246717	1.94327212	44.67076344	2.33E-11	4.70E-10
ENSRNOG00000025742	0.954097333	2.372863535	44.66105779	2.34E-11	4.72E-10
ENSRNOG00000058555	1.798788747	1.045653646	44.60090872	2.42E-11	4.85E-10
ENSRNOG00000042419	0.776431028	4.465900567	44.54007302	2.49E-11	4.99E-10
ENSRNOG00000000281	0.732144407	5.149609828	44.50409049	2.54E-11	5.07E-10
ENSRNOG00000055530	0.974198895	5.455014919	44.47113605	2.58E-11	5.15E-10
ENSRNOG00000013661	1.434303901	3.030625903	44.37750974	2.71E-11	5.39E-10
ENSRNOG00000018603	1.073151483	3.556830132	44.2927137	2.83E-11	5.61E-10
ENSRNOG00000017673	0.757488065	7.730282602	44.20748052	2.95E-11	5.84E-10
ENSRNOG00000001336	1.336711641	1.441635791	44.19676377	2.97E-11	5.86E-10
ENSRNOG00000004212	0.752589661	3.056056555	44.07853756	3.15E-11	6.20E-10
ENSRNOG00000018991	0.746340601	6.63910175	44.07407107	3.16E-11	6.21E-10
ENSRNOG00000002895	0.832965662	1.836028631	43.99180013	3.30E-11	6.45E-10
ENSRNOG00000019009	0.825511541	3.560909427	43.85245238	3.54E-11	6.90E-10
ENSRNOG00000017500	0.759650103	7.188718298	43.77478748	3.68E-11	7.16E-10
ENSRNOG00000020289	0.624559641	4.173316068	43.7140812	3.80E-11	7.36E-10
ENSRNOG00000029450	0.837047015	7.330196605	43.71355841	3.80E-11	7.36E-10
ENSRNOG00000009377	0.68570662	5.644780613	43.71093072	3.81E-11	7.36E-10
ENSRNOG00000018111	0.576254203	7.59772784	43.63032643	3.97E-11	7.64E-10
ENSRNOG00000002914	0.56356554	4.867754432	43.61828442	3.99E-11	7.67E-10
ENSRNOG00000000852	0.851677316	7.99583036	43.52981662	4.18E-11	8.01E-10
ENSRNOG00000001409	0.759689777	7.057053764	43.51395317	4.21E-11	8.07E-10
ENSRNOG00000024801	0.494640428	4.433056035	43.47224011	4.30E-11	8.23E-10
ENSRNOG00000051531	0.919925986	2.821660883	43.46323664	4.32E-11	8.26E-10
ENSRNOG00000000918	0.798848651	2.811517953	43.45405432	4.34E-11	8.28E-10
ENSRNOG00000033863	1.142496019	4.013522713	43.43162932	4.39E-11	8.37E-10
ENSRNOG00000025350	1.45481981	0.592317217	43.42559535	4.40E-11	8.37E-10
ENSRNOG00000050333	1.806429291	-0.706458099	43.42533545	4.40E-11	8.37E-10
ENSRNOG00000024818	1.287382854	0.218010508	43.21942847	4.89E-11	9.29E-10
ENSRNOG00000047967	0.90177419	2.554156713	43.13860541	5.10E-11	9.64E-10
ENSRNOG00000016968	0.494954422	4.984864982	43.07978983	5.26E-11	9.92E-10
ENSRNOG00000000476	0.745495484	3.950418978	43.068056	5.29E-11	9.97E-10
ENSRNOG00000016433	0.696857467	2.484850518	43.01687253	5.43E-11	1.02E-09
ENSRNOG00000049181	0.478462729	5.504797479	43.01065363	5.44E-11	1.02E-09
ENSRNOG00000055525	0.613709505	5.527092999	42.99710924	5.48E-11	1.03E-09
ENSRNOG00000019442	1.248078482	3.581067598	42.96837766	5.56E-11	1.04E-09
ENSRNOG00000006695	0.606659195	5.209075224	42.8652666	5.86E-11	1.09E-09
ENSRNOG00000007710	0.667187536	5.958705779	42.84788195	5.92E-11	1.10E-09
ENSRNOG00000019766	0.550865843	4.172809477	42.82148957	6.00E-11	1.12E-09
ENSRNOG00000007404	0.718451667	2.144360813	42.73159928	6.28E-11	1.17E-09
ENSRNOG00000014035	0.435997442	6.831112677	42.72674157	6.29E-11	1.17E-09
ENSRNOG00000024456	0.862376047	2.924299537	42.71763841	6.32E-11	1.17E-09
ENSRNOG00000033378	0.545390186	6.233841173	42.71393742	6.34E-11	1.17E-09
ENSRNOG00000008557	0.568581603	5.386735561	42.63843742	6.59E-11	1.22E-09
ENSRNOG00000013178	0.6598918	6.581316522	42.63857428	6.58E-11	1.22E-09
ENSRNOG00000014353	0.594571035	5.934895755	42.60569905	6.70E-11	1.24E-09
ENSRNOG00000019921	1.366829771	4.429704393	42.58550535	6.77E-11	1.25E-09
ENSRNOG00000016816	0.531776348	5.497423388	42.58396893	6.77E-11	1.25E-09
ENSRNOG00000055340	1.002073513	4.900550261	42.54216989	6.92E-11	1.27E-09
ENSRNOG00000032546	0.74250567	5.044588533	42.54034027	6.92E-11	1.27E-09
ENSRNOG00000014252	0.57089793	4.133833923	42.51431201	7.02E-11	1.29E-09
ENSRNOG00000051484	1.14048055	1.606630178	42.5023787	7.06E-11	1.29E-09
ENSRNOG00000005147	0.958396082	3.29780722	42.42092594	7.36E-11	1.34E-09
ENSRNOG00000056118	0.684438226	6.447465475	42.32980236	7.71E-11	1.40E-09
ENSRNOG00000010466	0.834716638	5.009950745	42.32857273	7.72E-11	1.40E-09
ENSRNOG00000013023	0.433020754	5.153152811	42.22571191	8.13E-11	1.48E-09
ENSRNOG00000007968	0.659341603	3.940639108	42.21142201	8.19E-11	1.49E-09
ENSRNOG00000030721	1.314185131	3.503510903	42.12793622	8.55E-11	1.55E-09
ENSRNOG00000013102	0.620526989	4.356799225	42.1249611	8.56E-11	1.55E-09
ENSRNOG00000008246	1.308778894	1.970529353	42.11050291	8.63E-11	1.55E-09
ENSRNOG00000005397	0.665541098	5.875922037	42.09195807	8.71E-11	1.57E-09
ENSRNOG00000020005	0.933741515	5.137895824	42.06326901	8.84E-11	1.59E-09
ENSRNOG00000027724	1.301191964	2.088750607	42.03879594	8.95E-11	1.60E-09
ENSRNOG00000053045	1.09990998	3.300686137	42.03955489	8.94E-11	1.60E-09
ENSRNOG00000024065	1.217580481	1.549929513	41.98883547	9.18E-11	1.64E-09
ENSRNOG00000028674	0.670375699	5.308705617	41.89072276	9.65E-11	1.72E-09
ENSRNOG00000019684	0.7542935	4.168153576	41.86090916	9.80E-11	1.75E-09
ENSRNOG00000000798	0.544134091	6.274878677	41.85056271	9.85E-11	1.75E-09
ENSRNOG00000018326	0.633926602	2.795755594	41.78478402	1.02E-10	1.81E-09
ENSRNOG00000049970	1.254746432	2.500915605	41.74778868	1.04E-10	1.84E-09
ENSRNOG00000006784	0.669418319	5.352623568	41.70329913	1.06E-10	1.88E-09
ENSRNOG00000002828	0.573355059	4.404789788	41.67499806	1.08E-10	1.90E-09

ENSRNOG00000001276	0.455552889	4.935210031	41.65165958	1.09E-10	1.92E-09
ENSRNOG00000060463	1.431500219	2.682911228	41.56552593	1.14E-10	2.00E-09
ENSRNOG00000012185	0.912897657	4.126644231	41.48768106	1.19E-10	2.07E-09
ENSRNOG00000014328	1.195395548	3.503745033	41.40526067	1.24E-10	2.16E-09
ENSRNOG00000042556	0.885387856	3.05359226	41.39247368	1.25E-10	2.17E-09
ENSRNOG00000018433	0.723907235	2.798467437	41.37394712	1.26E-10	2.19E-09
ENSRNOG00000046581	1.514846926	-0.217793886	41.33556322	1.28E-10	2.22E-09
ENSRNOG00000015632	0.689467291	3.01511763	41.27942593	1.32E-10	2.28E-09
ENSRNOG00000022738	1.431897542	-0.579803445	41.27297189	1.32E-10	2.29E-09
ENSRNOG00000001288	0.580148931	3.572038293	41.23147545	1.35E-10	2.33E-09
ENSRNOG00000048495	0.501697927	3.982962763	41.08727097	1.46E-10	2.50E-09
ENSRNOG00000009355	0.569611481	4.848875469	40.98563057	1.53E-10	2.62E-09
ENSRNOG00000007338	0.845487202	3.694341568	40.98679351	1.53E-10	2.62E-09
ENSRNOG00000020731	0.591909215	6.359710268	40.96078947	1.55E-10	2.65E-09
ENSRNOG00000048561	1.789704319	0.174327114	40.77914699	1.70E-10	2.90E-09
ENSRNOG00000003224	0.727193283	4.871739193	40.77267714	1.71E-10	2.91E-09
ENSRNOG00000031090	1.215552817	2.304785011	40.75530512	1.73E-10	2.93E-09
ENSRNOG00000015346	0.993817378	3.000686981	40.73961009	1.74E-10	2.95E-09
ENSRNOG00000049531	0.464412229	6.313432174	40.7272973	1.75E-10	2.96E-09
ENSRNOG00000000917	1.73777874	-0.471353397	40.71319155	1.76E-10	2.98E-09
ENSRNOG00000006551	0.756269012	3.623294083	40.64259338	1.83E-10	3.09E-09
ENSRNOG00000019742	0.383150284	5.634166077	40.63528893	1.83E-10	3.09E-09
ENSRNOG00000013460	0.917049674	2.489618831	40.60628107	1.86E-10	3.14E-09
ENSRNOG00000017078	0.842676946	4.286696318	40.46405109	2.00E-10	3.36E-09
ENSRNOG00000053725	0.500255748	5.517608225	40.42868214	2.04E-10	3.41E-09
ENSRNOG00000010549	1.24996251	0.425966371	40.3520915	2.12E-10	3.53E-09
ENSRNOG00000020020	0.504327441	5.529715429	40.22436736	2.26E-10	3.75E-09
ENSRNOG00000020793	0.655987096	4.375863932	40.20297284	2.29E-10	3.79E-09
ENSRNOG00000053285	0.580589327	6.101234334	40.19802917	2.29E-10	3.79E-09
ENSRNOG00000043212	0.856575879	5.37314033	40.0585343	2.46E-10	4.06E-09
ENSRNOG00000019968	0.448729629	5.803178708	40.05186054	2.47E-10	4.07E-09
ENSRNOG00000011507	0.593369067	4.302976723	39.97993962	2.57E-10	4.21E-09
ENSRNOG00000001729	0.868915705	2.311506254	39.96130738	2.59E-10	4.25E-09
ENSRNOG00000013013	0.553408578	4.692368002	39.8812026	2.70E-10	4.42E-09
ENSRNOG00000003334	0.575102161	3.468894898	39.85541485	2.73E-10	4.47E-09
ENSRNOG00000030830	0.77471849	2.503462392	39.64608363	3.04E-10	4.97E-09
ENSRNOG00000047873	0.562438614	3.202298392	39.62016942	3.08E-10	5.03E-09
ENSRNOG00000055597	1.022695178	3.101054683	39.5649211	3.17E-10	5.16E-09
ENSRNOG00000018952	0.662660869	3.686456029	39.50803382	3.27E-10	5.31E-09
ENSRNOG00000032590	0.883483508	3.886138379	39.47398462	3.32E-10	5.38E-09
ENSRNOG00000013498	0.700997584	4.777130122	39.4591114	3.35E-10	5.42E-09
ENSRNOG00000000423	1.124959131	0.878644581	39.44220232	3.38E-10	5.46E-09
ENSRNOG00000015762	0.595765415	3.780243429	39.33691586	3.57E-10	5.76E-09
ENSRNOG00000029500	0.898374602	2.864417967	39.17406029	3.88E-10	6.23E-09
ENSRNOG00000019718	1.149687019	0.481039184	39.16171878	3.90E-10	6.26E-09
ENSRNOG00000061365	1.595986561	0.343800677	39.02796283	4.18E-10	6.68E-09
ENSRNOG00000009531	1.529823131	2.917832853	39.01926377	4.20E-10	6.69E-09
ENSRNOG00000006513	0.599925905	3.311076589	38.97264128	4.30E-10	6.85E-09
ENSRNOG00000021872	0.692262459	3.317297465	38.94913587	4.35E-10	6.92E-09
ENSRNOG00000007727	0.816687322	3.572975376	38.92139578	4.41E-10	7.01E-09
ENSRNOG00000014278	0.983619976	3.433225194	38.89770857	4.47E-10	7.09E-09
ENSRNOG00000032677	0.801837539	3.780065142	38.85516522	4.56E-10	7.23E-09
ENSRNOG00000018020	0.741722887	7.503238854	38.74108992	4.84E-10	7.65E-09
ENSRNOG00000060615	0.648696262	5.789710524	38.74285388	4.83E-10	7.65E-09
ENSRNOG00000006539	0.740826755	3.533338664	38.64069004	5.09E-10	8.02E-09
ENSRNOG00000056678	1.260139034	3.654918525	38.61953807	5.15E-10	8.09E-09
ENSRNOG00000008401	1.079197811	0.854874422	38.59932145	5.20E-10	8.17E-09
ENSRNOG00000026874	0.586977536	3.121135762	38.56566666	5.29E-10	8.29E-09
ENSRNOG00000016488	0.529143643	5.888421644	38.55981871	5.31E-10	8.31E-09
ENSRNOG00000020773	0.78013743	3.747837227	38.51499623	5.43E-10	8.49E-09
ENSRNOG00000019388	0.705866196	2.80970766	38.49877363	5.48E-10	8.55E-09
ENSRNOG00000007957	0.698928333	7.054695786	38.49899579	5.48E-10	8.55E-09
ENSRNOG00000032925	2.577926681	-1.146668779	38.44485207	5.63E-10	8.76E-09
ENSRNOG00000021255	0.695094355	5.004250628	38.4326887	5.67E-10	8.81E-09
ENSRNOG00000032878	0.761872538	4.453505252	38.42912202	5.68E-10	8.81E-09
ENSRNOG00000009084	0.66677328	2.775585813	38.36466023	5.87E-10	9.08E-09
ENSRNOG00000007514	1.683066817	0.366875284	38.31084596	6.03E-10	9.33E-09
ENSRNOG00000004032	0.634772445	6.057624369	38.29452997	6.08E-10	9.39E-09
ENSRNOG00000002753	0.69432232	6.583148006	38.24182118	6.25E-10	9.64E-09
ENSRNOG00000018582	1.557459833	-0.446908833	38.2339905	6.27E-10	9.67E-09
ENSRNOG00000018536	0.69972368	3.395016542	38.20802293	6.36E-10	9.79E-09
ENSRNOG00000032561	0.88739329	2.494686135	38.1914904	6.41E-10	9.85E-09
ENSRNOG00000013429	0.564759262	3.820495418	38.05826887	6.87E-10	1.05E-08
ENSRNOG00000050735	0.994459064	3.49884012	37.97952444	7.15E-10	1.09E-08
ENSRNOG00000024435	0.769442459	4.091178544	37.9059343	7.42E-10	1.13E-08
ENSRNOG00000013468	0.92219403	3.434472411	37.79888769	7.84E-10	1.19E-08
ENSRNOG00000016774	0.537540425	4.329334565	37.79218844	7.87E-10	1.19E-08
ENSRNOG00000011781	0.589017154	4.038721765	37.72363013	8.15E-10	1.23E-08
ENSRNOG00000059961	0.622998323	5.477497264	37.69796534	8.26E-10	1.25E-08
ENSRNOG00000024294	2.018355943	0.350619465	37.64187276	8.50E-10	1.28E-08
ENSRNOG00000000851	0.520216146	6.74490432	37.5745416	8.80E-10	1.32E-08
ENSRNOG00000001449	0.548079512	5.874931111	37.5433507	8.94E-10	1.34E-08
ENSRNOG00000020021	0.554522668	5.399522689	37.46702011	9.30E-10	1.39E-08
ENSRNOG00000060004	1.591092962	0.001257431	37.45329591	9.36E-10	1.40E-08
ENSRNOG00000020862	0.632953923	5.093408631	37.45460224	9.36E-10	1.40E-08
ENSRNOG00000058695	1.545976819	0.300760123	37.3813028	9.71E-10	1.45E-08
ENSRNOG00000055770	1.545976819	0.300760123	37.3813028	9.71E-10	1.45E-08
ENSRNOG00000050183	1.164856334	1.024619227	37.35491949	9.85E-10	1.47E-08
ENSRNOG00000019883	0.810120024	2.740877474	37.31569396	1.00E-09	1.50E-08
ENSRNOG00000040281	0.626270989	3.151289168	37.2950936	1.02E-09	1.51E-08
ENSRNOG00000015303	1.038286792	3.753639233	37.28675676	1.02E-09	1.52E-08
ENSRNOG00000024025	0.459675079	6.244536731	37.26493305	1.03E-09	1.53E-08
ENSRNOG00000014021	1.362891015	0.323219026	37.26114788	1.03E-09	1.53E-08
ENSRNOG00000048487	0.662165731	3.477872702	37.2228771	1.05E-09	1.56E-08
ENSRNOG00000021437	1.379213215	1.398628239	37.16093548	1.09E-09	1.61E-08
ENSRNOG00000019587	0.572343203	8.320921539	37.10204803	1.12E-09	1.66E-08
ENSRNOG00000012163	0.667165347	6.126240724	37.06708297	1.14E-09	1.68E-08
ENSRNOG00000046480	0.920451613	0.766050415	37.00506922	1.18E-09	1.74E-08
ENSRNOG00000020501	1.298138648	1.745850211	36.94279389	1.22E-09	1.79E-08
ENSRNOG00000017349	0.398538085	5.972403078	36.90863322	1.24E-09	1.82E-08

ENSRNOG00000019612	0.84250778	3.262266623	36.85853086	1.27E-09	1.87E-08
ENSRNOG00000018101	1.067125323	2.408528567	36.81223642	1.30E-09	1.91E-08
ENSRNOG00000043381	0.430026762	5.205512533	36.81316313	1.30E-09	1.91E-08
ENSRNOG00000019075	0.638390952	4.517507349	36.79851987	1.31E-09	1.91E-08
ENSRNOG00000047737	1.500873751	0.413015257	36.71548723	1.37E-09	1.99E-08
ENSRNOG00000059507	1.104222389	1.390831978	36.64956287	1.41E-09	2.06E-08
ENSRNOG00000047575	0.700901364	2.479381505	36.59659998	1.45E-09	2.11E-08
ENSRNOG00000026917	1.35214154	-0.224586693	36.59239959	1.46E-09	2.12E-08
ENSRNOG00000021058	0.622411608	3.244129666	36.58229149	1.46E-09	2.12E-08
ENSRNOG00000056038	0.645212132	4.329012777	36.57358956	1.47E-09	2.13E-08
ENSRNOG00000032948	0.509226413	3.976545659	36.56583084	1.48E-09	2.14E-08
ENSRNOG00000028556	0.55154049	5.611266104	36.49925712	1.53E-09	2.21E-08
ENSRNOG00000014658	0.931680443	4.373325703	36.48663315	1.54E-09	2.22E-08
ENSRNOG00000038375	1.295371597	1.082242349	36.44325876	1.57E-09	2.27E-08
ENSRNOG00000052493	1.594049472	0.347689394	36.29321957	1.70E-09	2.44E-08
ENSRNOG00000019907	0.763092942	1.894282886	36.29066829	1.70E-09	2.44E-08
ENSRNOG00000038999	1.146863793	3.693987411	36.28197924	1.71E-09	2.45E-08
ENSRNOG00000015382	0.715884235	2.199058036	36.22089931	1.76E-09	2.52E-08
ENSRNOG00000020416	0.957109989	4.461842173	36.18978273	1.79E-09	2.56E-08
ENSRNOG00000008334	0.994639745	4.318161541	36.17756303	1.80E-09	2.57E-08
ENSRNOG00000018949	0.946525281	3.240545164	36.17866737	1.80E-09	2.57E-08
ENSRNOG00000031208	0.516178006	4.974301891	36.15523816	1.82E-09	2.60E-08
ENSRNOG00000049097	1.482530026	0.400074021	36.12900731	1.85E-09	2.63E-08
ENSRNOG00000010219	0.630819053	5.940449125	36.09171436	1.88E-09	2.67E-08
ENSRNOG00000031643	0.852843228	4.761947594	36.05762991	1.92E-09	2.72E-08
ENSRNOG00000005153	0.526044367	3.151719772	36.03722025	1.94E-09	2.74E-08
ENSRNOG00000028017	0.606951722	4.410495331	35.97444984	2.00E-09	2.82E-08
ENSRNOG00000001339	0.592186367	3.826021659	35.9191703	2.06E-09	2.89E-08
ENSRNOG00000018462	0.774151813	2.817858278	35.88905187	2.09E-09	2.93E-08
ENSRNOG00000036696	0.5870051	5.920781587	35.88834837	2.09E-09	2.93E-08
ENSRNOG00000018863	0.813788083	4.116795219	35.85822154	2.12E-09	2.97E-08
ENSRNOG00000020030	0.933517354	4.600612925	35.85990334	2.12E-09	2.97E-08
ENSRNOG00000014170	0.71321877	6.861641711	35.82121622	2.16E-09	3.02E-08
ENSRNOG00000025832	0.658031958	7.31600168	35.81217627	2.17E-09	3.03E-08
ENSRNOG00000021447	2.1125378	0.746587948	35.8095357	2.18E-09	3.04E-08
ENSRNOG00000017108	1.0622453	5.051311331	35.78648653	2.20E-09	3.07E-08
ENSRNOG00000053889	0.474081309	6.196585625	35.78462096	2.20E-09	3.07E-08
ENSRNOG00000019773	0.657819283	4.00964607	35.75468073	2.24E-09	3.11E-08
ENSRNOG00000015187	0.620666928	4.303499905	35.74382996	2.25E-09	3.13E-08
ENSRNOG00000058402	1.7775707	-0.940530586	35.70803963	2.29E-09	3.18E-08
ENSRNOG00000048699	1.044484	2.467627317	35.68595174	2.32E-09	3.21E-08
ENSRNOG00000050646	0.559005709	4.281031236	35.5977483	2.43E-09	3.35E-08
ENSRNOG00000010634	0.57812745	3.404401058	35.59439766	2.43E-09	3.35E-08
ENSRNOG00000001189	0.618474318	3.321303665	35.57577064	2.45E-09	3.38E-08
ENSRNOG00000001429	0.743423286	3.061616904	35.53897642	2.50E-09	3.44E-08
ENSRNOG00000017155	0.769008928	3.711502069	35.52755028	2.51E-09	3.46E-08
ENSRNOG00000054121	0.610190086	4.195912558	35.49878519	2.55E-09	3.51E-08
ENSRNOG00000016920	0.874738568	3.766696134	35.48484108	2.57E-09	3.53E-08
ENSRNOG00000045580	0.969349546	0.982979856	35.43578467	2.64E-09	3.62E-08
ENSRNOG00000022567	1.107187047	1.087174372	35.40249846	2.68E-09	3.67E-08
ENSRNOG00000013987	0.556934625	7.027162094	35.40048215	2.68E-09	3.67E-08
ENSRNOG00000053095	0.642374862	3.73243277	35.38152146	2.71E-09	3.71E-08
ENSRNOG00000001891	0.881300084	1.784986592	35.3742137	2.72E-09	3.72E-08
ENSRNOG00000022607	0.610054542	3.650042101	35.34157443	2.77E-09	3.78E-08
ENSRNOG00000025808	0.52581151	3.247044755	35.32686744	2.79E-09	3.80E-08
ENSRNOG00000012323	0.641863996	5.091922573	35.27690555	2.86E-09	3.89E-08
ENSRNOG00000027238	0.680504867	2.859172384	35.26265395	2.88E-09	3.91E-08
ENSRNOG00000013874	1.53472059	7.729916234	35.26383293	2.88E-09	3.91E-08
ENSRNOG00000052141	0.782504885	4.373092616	35.23101929	2.93E-09	3.97E-08
ENSRNOG00000018748	1.340014648	3.013007535	35.22064806	2.94E-09	3.99E-08
ENSRNOG00000050298	0.557979868	5.008686144	35.19574083	2.98E-09	4.03E-08
ENSRNOG00000013101	0.582792759	4.727791184	35.16353265	3.03E-09	4.10E-08
ENSRNOG00000046566	0.610598901	5.190695131	35.15283999	3.05E-09	4.11E-08
ENSRNOG00000013653	0.890792293	4.242555382	35.13012119	3.08E-09	4.16E-08
ENSRNOG00000007439	0.923916018	0.880483876	35.07522226	3.17E-09	4.27E-08
ENSRNOG00000009422	0.525452082	2.914413292	35.02678343	3.25E-09	4.37E-08
ENSRNOG00000046756	0.456110766	5.3060374	35.02354138	3.26E-09	4.38E-08
ENSRNOG00000050848	0.875709854	4.067321234	34.98559335	3.32E-09	4.46E-08
ENSRNOG00000017513	0.551113488	5.836109915	34.97208271	3.34E-09	4.49E-08
ENSRNOG00000008897	0.501626759	5.524786808	34.94886183	3.38E-09	4.53E-08
ENSRNOG00000008382	1.775619527	-1.349952816	34.88702127	3.49E-09	4.68E-08
ENSRNOG00000005193	0.364529195	5.818572293	34.80723348	3.64E-09	4.86E-08
ENSRNOG00000013376	0.923242842	2.82902338	34.74723809	3.75E-09	5.00E-08
ENSRNOG00000017641	0.52924437	6.421967211	34.71983693	3.81E-09	5.05E-08
ENSRNOG00000019682	0.978524067	4.432073233	34.70879416	3.83E-09	5.08E-08
ENSRNOG00000000661	0.522299621	4.251934496	34.62725923	3.99E-09	5.28E-08
ENSRNOG00000049255	1.335778184	0.266815899	34.58458197	4.08E-09	5.39E-08
ENSRNOG00000008487	0.634298526	4.415189325	34.55966044	4.13E-09	5.45E-08
ENSRNOG00000018977	0.393977398	7.510977571	34.48541687	4.29E-09	5.65E-08
ENSRNOG00000050381	0.660019404	3.918863357	34.4846592	4.30E-09	5.65E-08
ENSRNOG00000020061	0.98961534	3.402674444	34.48314966	4.30E-09	5.65E-08
ENSRNOG00000003728	0.541501444	4.312854578	34.42576264	4.43E-09	5.81E-08
ENSRNOG00000016414	0.554648853	8.589894565	34.37603394	4.54E-09	5.94E-08
ENSRNOG00000014615	0.482129749	5.479748805	34.3041353	4.71E-09	6.14E-08
ENSRNOG00000017002	2.039550466	-0.099987465	34.27744668	4.78E-09	6.21E-08
ENSRNOG00000056009	0.73659484	5.465522507	34.27395115	4.79E-09	6.22E-08
ENSRNOG00000029028	0.644995935	4.969193427	34.25168188	4.84E-09	6.28E-08
ENSRNOG00000021067	0.741765183	3.670627657	34.24115898	4.87E-09	6.31E-08
ENSRNOG00000020261	0.573813526	4.700482326	34.21762505	4.93E-09	6.38E-08
ENSRNOG00000054172	0.459209294	7.713553905	34.19764831	4.98E-09	6.44E-08
ENSRNOG00000004014	0.535752368	4.11596549	34.08390575	5.28E-09	6.80E-08
ENSRNOG00000018457	0.578013047	7.033864127	33.98026287	5.57E-09	7.15E-08
ENSRNOG00000027606	1.644211506	1.637979472	33.93323921	5.70E-09	7.31E-08
ENSRNOG00000031801	0.963004671	2.89262265	33.89565202	5.81E-09	7.45E-08
ENSRNOG00000061121	0.445710541	7.20088006	33.84991874	5.95E-09	7.61E-08
ENSRNOG00000016665	0.494528936	4.249811195	33.79310355	6.13E-09	7.83E-08
ENSRNOG00000023764	0.594625962	4.455754176	33.78889137	6.14E-09	7.84E-08
ENSRNOG00000012991	0.813247281	3.722256833	33.78607079	6.15E-09	7.84E-08
ENSRNOG00000027193	0.793336176	4.728484604	33.77407886	6.19E-09	7.88E-08
ENSRNOG00000020853	0.452939425	6.291180383	33.71698375	6.37E-09	8.11E-08

ENSRNOG00000026400	1.483659151	0.182590242	33.71302586	6.39E-09	8.11E-08
ENSRNOG00000009712	0.911603066	2.382342947	33.70108116	6.43E-09	8.15E-08
ENSRNOG00000013220	0.967733155	2.14544851	33.66113838	6.56E-09	8.29E-08
ENSRNOG00000001082	0.750349103	4.119622453	33.64544543	6.61E-09	8.34E-08
ENSRNOG00000019674	0.500291744	4.125837074	33.62935506	6.67E-09	8.41E-08
ENSRNOG00000020280	0.98344313	3.35765449	33.54163228	6.98E-09	8.78E-08
ENSRNOG00000020191	0.454860137	4.933585436	33.51393046	7.08E-09	8.90E-08
ENSRNOG00000021338	1.080674981	6.764709952	33.46378725	7.26E-09	9.11E-08
ENSRNOG00000007905	0.763557028	3.887804397	33.45843755	7.28E-09	9.13E-08
ENSRNOG00000017468	0.914520996	1.385159877	33.45435287	7.30E-09	9.13E-08
ENSRNOG00000053712	0.813716058	2.75208741	33.45466709	7.29E-09	9.13E-08
ENSRNOG00000020847	0.5565215	3.09484653	33.43586939	7.37E-09	9.21E-08
ENSRNOG00000004205	1.040234251	1.978731362	33.4281077	7.39E-09	9.24E-08
ENSRNOG00000024549	0.590857189	4.515432174	33.42272392	7.42E-09	9.26E-08
ENSRNOG00000001148	0.564134469	7.720210457	33.40237255	7.49E-09	9.35E-08
ENSRNOG00000043300	0.975370956	4.823736875	33.31766963	7.83E-09	9.74E-08
ENSRNOG00000055814	1.694827485	-0.927635218	33.30168611	7.89E-09	9.82E-08
ENSRNOG00000014629	0.706810547	3.07297356	33.29123321	7.93E-09	9.86E-08
ENSRNOG00000008362	0.56630826	2.946410371	33.25862932	8.07E-09	1.00E-07
ENSRNOG00000057322	0.795702312	2.45947442	33.22496839	8.21E-09	1.02E-07
ENSRNOG00000028206	1.170402051	0.622632638	33.20107934	8.31E-09	1.03E-07
ENSRNOG00000019735	0.546968234	3.065003421	33.12757768	8.63E-09	1.07E-07
ENSRNOG00000021041	0.937012627	4.748062434	33.06709018	8.90E-09	1.10E-07
ENSRNOG00000024616	1.312153666	0.286767635	33.04368219	9.01E-09	1.11E-07
ENSRNOG00000016976	0.39927908	5.064274617	33.0410248	9.02E-09	1.11E-07
ENSRNOG00000020058	0.651381857	7.343024419	32.96977252	9.36E-09	1.15E-07
ENSRNOG00000010029	1.328277573	2.179498566	32.96666094	9.38E-09	1.15E-07
ENSRNOG00000030705	0.409058409	5.458041147	32.94437477	9.48E-09	1.16E-07
ENSRNOG00000018118	0.496154301	4.359005227	32.90882484	9.66E-09	1.18E-07
ENSRNOG00000045857	0.534622253	3.182381953	32.91017384	9.65E-09	1.18E-07
ENSRNOG00000016161	0.651659289	4.983088485	32.86871728	9.86E-09	1.20E-07
ENSRNOG00000021540	0.73651063	4.515028185	32.80015585	1.02E-08	1.24E-07
ENSRNOG00000001107	0.718603801	1.9871709	32.78524512	1.03E-08	1.25E-07
ENSRNOG00000018231	0.66551752	6.153350415	32.76343891	1.04E-08	1.27E-07
ENSRNOG00000038970	0.772395289	2.014584674	32.75394801	1.05E-08	1.27E-07
ENSRNOG00000019955	0.389998706	6.061417434	32.73712052	1.06E-08	1.28E-07
ENSRNOG00000020060	0.706015593	4.315504622	32.73091316	1.06E-08	1.28E-07
ENSRNOG00000012055	0.419374284	6.571553574	32.69020528	1.08E-08	1.31E-07
ENSRNOG00000021025	0.33664085	6.722527331	32.60121974	1.13E-08	1.37E-07
ENSRNOG00000023152	0.593181072	4.022197837	32.57141165	1.15E-08	1.39E-07
ENSRNOG00000009760	0.826695511	6.705779662	32.52474391	1.18E-08	1.42E-07
ENSRNOG00000049814	1.235638719	1.796114014	32.51379477	1.18E-08	1.42E-07
ENSRNOG00000001397	0.491622903	3.318670856	32.4730236	1.21E-08	1.45E-07
ENSRNOG00000009173	1.658654477	-1.161039448	32.46959625	1.21E-08	1.45E-07
ENSRNOG00000046051	1.577324501	0.772612976	32.43846046	1.23E-08	1.47E-07
ENSRNOG00000045586	0.840644948	2.095232934	32.42346938	1.24E-08	1.48E-07
ENSRNOG00000037562	1.027792227	0.026371598	32.40483165	1.25E-08	1.50E-07
ENSRNOG00000018168	0.822971753	4.481025674	32.40608063	1.25E-08	1.50E-07
ENSRNOG00000020277	0.529638792	7.734728964	32.34752962	1.29E-08	1.54E-07
ENSRNOG00000037984	1.464606717	0.810179023	32.34793796	1.29E-08	1.54E-07
ENSRNOG00000021611	0.74834061	6.470635059	32.34062379	1.29E-08	1.54E-07
ENSRNOG00000053203	0.476873802	5.31384817	32.32841492	1.30E-08	1.55E-07
ENSRNOG00000017953	0.532909256	3.289627641	32.28494644	1.33E-08	1.58E-07
ENSRNOG00000018830	0.663435037	4.428608484	32.24798588	1.36E-08	1.61E-07
ENSRNOG00000003243	0.572747847	4.323409051	32.20202576	1.39E-08	1.64E-07
ENSRNOG00000011346	0.812599391	2.502105048	32.17495826	1.41E-08	1.66E-07
ENSRNOG00000006394	0.545975147	3.193846525	32.1681482	1.41E-08	1.67E-07
ENSRNOG00000018985	0.465437557	6.999630078	32.05440509	1.50E-08	1.76E-07
ENSRNOG00000014008	0.434005875	4.998306779	32.02627237	1.52E-08	1.78E-07
ENSRNOG00000039807	0.598317462	4.697019722	32.01080402	1.53E-08	1.80E-07
ENSRNOG00000001432	0.697056985	2.294459469	31.95498211	1.58E-08	1.85E-07
ENSRNOG00000026295	0.805921224	1.476495058	31.77745176	1.73E-08	2.01E-07
ENSRNOG00000021198	0.508440736	3.872879736	31.77207464	1.73E-08	2.02E-07
ENSRNOG00000061961	1.027957018	2.924381647	31.74527846	1.76E-08	2.04E-07
ENSRNOG00000033973	1.386459572	-0.588684185	31.65842499	1.84E-08	2.13E-07
ENSRNOG00000048733	0.502091464	4.76301798	31.64172941	1.85E-08	2.15E-07
ENSRNOG00000012324	0.568366779	4.880120218	31.62965284	1.87E-08	2.16E-07
ENSRNOG00000010254	1.308709548	1.535580953	31.57052792	1.92E-08	2.22E-07
ENSRNOG00000030579	1.115346404	1.909881228	31.55429524	1.94E-08	2.24E-07
ENSRNOG00000053370	0.411954647	3.833183416	31.50641646	1.99E-08	2.29E-07
ENSRNOG00000046502	0.438354043	6.149371567	31.50178536	1.99E-08	2.29E-07
ENSRNOG00000060098	0.717010577	7.835691946	31.49649082	2.00E-08	2.29E-07
ENSRNOG00000018408	0.435753267	4.220818959	31.41413648	2.08E-08	2.39E-07
ENSRNOG00000019932	0.517463636	5.875018308	31.38868466	2.11E-08	2.42E-07
ENSRNOG00000012378	0.511140039	5.695105334	31.36747769	2.14E-08	2.44E-07
ENSRNOG00000016258	0.636804217	3.238173265	31.35929085	2.14E-08	2.45E-07
ENSRNOG00000010771	0.434499821	7.361240775	31.31861275	2.19E-08	2.50E-07
ENSRNOG00000018359	0.469732681	4.138950298	31.28306139	2.23E-08	2.54E-07
ENSRNOG00000012106	0.370919969	5.833394663	31.25408077	2.26E-08	2.58E-07
ENSRNOG00000013090	1.01942385	1.771343988	31.24955412	2.27E-08	2.58E-07
ENSRNOG00000013604	0.534789993	6.916135569	31.22003455	2.30E-08	2.62E-07
ENSRNOG00000039861	0.910148851	3.520703634	31.19468297	2.33E-08	2.65E-07
ENSRNOG00000000316	0.635285556	4.191525313	31.18855751	2.34E-08	2.66E-07
ENSRNOG00000000504	0.882511094	3.220776635	31.1634716	2.37E-08	2.69E-07
ENSRNOG00000007648	0.396120102	6.938159393	31.13770359	2.40E-08	2.72E-07
ENSRNOG00000022373	0.484224234	5.45569824	31.12377187	2.42E-08	2.74E-07
ENSRNOG00000017795	0.767549459	4.828549122	31.11439857	2.43E-08	2.75E-07
ENSRNOG00000020254	0.443761059	5.307135321	31.10802342	2.44E-08	2.76E-07
ENSRNOG00000049355	0.682061323	2.322650362	31.07767714	2.48E-08	2.80E-07
ENSRNOG00000007628	0.71332817	5.16472643	30.96592031	2.63E-08	2.95E-07
ENSRNOG00000039928	1.047381012	0.572564765	30.95377534	2.64E-08	2.97E-07
ENSRNOG00000031367	1.696582038	0.567551044	30.93395585	2.67E-08	3.00E-07
ENSRNOG00000060707	0.591900927	5.309665979	30.79990713	2.86E-08	3.20E-07
ENSRNOG00000052204	0.466008519	5.081862394	30.79736648	2.86E-08	3.21E-07
ENSRNOG00000038622	0.751759146	2.31097394	30.78884583	2.88E-08	3.21E-07
ENSRNOG00000046211	0.611682218	4.252450025	30.78980769	2.88E-08	3.21E-07
ENSRNOG00000020358	0.658258828	6.304002238	30.73448663	2.96E-08	3.30E-07
ENSRNOG00000061639	1.117371036	1.149205953	30.70086018	3.01E-08	3.35E-07
ENSRNOG00000033639	0.446334614	4.149448973	30.68258653	3.04E-08	3.38E-07
ENSRNOG00000020623	0.720803235	3.605446751	30.66919467	3.06E-08	3.40E-07

ENSRNOG00000055761	0.601158666	4.311316652	30.64393806	3.10E-08	3.44E-07
ENSRNOG00000019681	0.911377905	1.7229638	30.63275107	3.12E-08	3.46E-07
ENSRNOG00000060973	1.74600877	0.014524621	30.63127836	3.12E-08	3.46E-07
ENSRNOG00000016037	1.387179534	0.175244926	30.61857582	3.14E-08	3.48E-07
ENSRNOG00000020640	0.744640679	3.333510813	30.6058559	3.16E-08	3.50E-07
ENSRNOG00000000465	0.467267872	5.591098092	30.59493626	3.18E-08	3.52E-07
ENSRNOG00000020797	0.842550262	0.847339761	30.5769269	3.21E-08	3.55E-07
ENSRNOG00000016565	0.804339215	1.480249707	30.49050898	3.36E-08	3.71E-07
ENSRNOG00000043225	0.975670731	2.137476222	30.43011921	3.46E-08	3.82E-07
ENSRNOG00000006238	0.490443941	3.09364687	30.38301249	3.55E-08	3.91E-07
ENSRNOG00000001117	1.245528735	0.204409484	30.35359116	3.60E-08	3.97E-07
ENSRNOG00000042355	0.695332771	3.646232245	30.33733298	3.63E-08	4.00E-07
ENSRNOG00000020427	0.671443274	3.651822189	30.32142933	3.66E-08	4.03E-07
ENSRNOG00000004860	1.371860877	-0.170176042	30.31648819	3.67E-08	4.03E-07
ENSRNOG00000014592	0.73927977	2.002482955	30.28845282	3.72E-08	4.09E-07
ENSRNOG00000021185	0.84176439	1.233462514	30.26275506	3.77E-08	4.14E-07
ENSRNOG00000018502	0.585692222	3.54796652	30.23796516	3.82E-08	4.18E-07
ENSRNOG00000001104	0.51608258	4.348926716	30.2317517	3.83E-08	4.20E-07
ENSRNOG00000015911	0.847572737	3.266560728	30.16846008	3.96E-08	4.33E-07
ENSRNOG00000014969	0.734620712	4.115546926	30.1294887	4.04E-08	4.41E-07
ENSRNOG00000016398	0.59718767	8.855263747	30.06637818	4.18E-08	4.55E-07
ENSRNOG00000010652	0.712079307	2.92792287	30.06029377	4.19E-08	4.56E-07
ENSRNOG00000016064	0.573118143	6.767957075	30.04338295	4.22E-08	4.59E-07
ENSRNOG00000046447	1.385707138	0.155840798	30.03875906	4.23E-08	4.60E-07
ENSRNOG00000023403	0.832668838	2.963029387	30.02744431	4.26E-08	4.63E-07
ENSRNOG00000020673	0.440287006	5.12542743	29.96301339	4.40E-08	4.78E-07
ENSRNOG00000007939	0.630755079	2.803986613	29.90530394	4.54E-08	4.91E-07
ENSRNOG00000060110	0.690567636	1.525784167	29.90525033	4.54E-08	4.91E-07
ENSRNOG00000023258	0.37534299	4.252070183	29.90276447	4.54E-08	4.91E-07
ENSRNOG00000001450	0.507442079	3.283715528	29.87420121	4.61E-08	4.98E-07
ENSRNOG00000009224	0.625047105	3.907623387	29.85557617	4.65E-08	5.01E-07
ENSRNOG00000016465	0.438490782	2.986077822	29.85261219	4.66E-08	5.02E-07
ENSRNOG00000030800	0.902657055	1.517996311	29.81682415	4.75E-08	5.11E-07
ENSRNOG00000058068	0.989189231	1.130787827	29.80401276	4.78E-08	5.13E-07
ENSRNOG00000043077	0.993165187	1.921455406	29.78958923	4.82E-08	5.17E-07
ENSRNOG00000030712	0.952865012	3.353469589	29.78478573	4.83E-08	5.18E-07
ENSRNOG00000000693	0.5158062	5.973932166	29.77703335	4.85E-08	5.19E-07
ENSRNOG00000045840	1.480925695	-0.315598124	29.74264039	4.93E-08	5.28E-07
ENSRNOG00000028161	0.532852034	3.928633458	29.7074073	5.02E-08	5.37E-07
ENSRNOG00000049951	1.205193741	0.58476309	29.66686963	5.13E-08	5.47E-07
ENSRNOG00000018836	0.44928203	6.885342752	29.66691803	5.13E-08	5.47E-07
ENSRNOG00000024796	0.514625342	5.390312611	29.66442705	5.14E-08	5.47E-07
ENSRNOG00000004169	0.855367067	4.038773694	29.65582133	5.16E-08	5.49E-07
ENSRNOG00000001454	0.559982046	7.848709326	29.63829905	5.21E-08	5.53E-07
ENSRNOG00000054513	1.383613179	0.539731674	29.61531774	5.27E-08	5.59E-07
ENSRNOG00000018117	0.50139299	6.528336201	29.58386356	5.35E-08	5.68E-07
ENSRNOG00000025589	1.12206193	5.584051507	29.56289647	5.41E-08	5.73E-07
ENSRNOG00000019553	0.573344384	5.494777575	29.55171559	5.44E-08	5.75E-07
ENSRNOG00000049931	0.541578448	4.505048965	29.54962279	5.45E-08	5.76E-07
ENSRNOG00000048843	0.659850551	3.134093999	29.54369493	5.47E-08	5.77E-07
ENSRNOG00000054605	0.652881685	3.462156468	29.5351063	5.49E-08	5.79E-07
ENSRNOG00000013515	1.198042314	4.41201142	29.51913113	5.54E-08	5.84E-07
ENSRNOG00000023456	0.506954704	5.373569438	29.49583188	5.60E-08	5.90E-07
ENSRNOG00000037340	0.988195421	3.426161376	29.49153993	5.62E-08	5.91E-07
ENSRNOG00000034107	0.873337407	2.291911918	29.45726898	5.72E-08	6.01E-07
ENSRNOG00000018823	0.404427866	8.375631522	29.43388772	5.79E-08	6.07E-07
ENSRNOG00000018951	0.483608672	4.14433723	29.40918102	5.86E-08	6.14E-07
ENSRNOG00000050805	1.543364285	-0.391114776	29.40107612	5.88E-08	6.16E-07
ENSRNOG00000000567	0.668281809	5.269454033	29.40141465	5.88E-08	6.16E-07
ENSRNOG00000028624	0.937769096	2.652198703	29.38803701	5.92E-08	6.20E-07
ENSRNOG00000057149	1.076921411	1.470257478	29.2956852	6.21E-08	6.49E-07
ENSRNOG00000001272	0.372677349	5.736106574	29.28577576	6.25E-08	6.52E-07
ENSRNOG00000059344	0.349407365	5.727646354	29.2790213	6.27E-08	6.54E-07
ENSRNOG00000031834	0.653531168	4.397663233	29.26851832	6.30E-08	6.57E-07
ENSRNOG00000037967	0.682425044	3.294310138	29.21419473	6.48E-08	6.75E-07
ENSRNOG00000048282	0.72337817	5.166557786	29.18500268	6.58E-08	6.85E-07
ENSRNOG00000003302	0.400547192	5.32570461	29.1778151	6.60E-08	6.87E-07
ENSRNOG00000014405	0.739279092	3.949342112	29.17597199	6.61E-08	6.87E-07
ENSRNOG00000001215	0.540540988	4.048387387	29.15218766	6.69E-08	6.95E-07
ENSRNOG00000020006	0.678219683	3.754966213	29.12264917	6.79E-08	7.05E-07
ENSRNOG00000050424	0.558399673	4.229051171	29.12263439	6.79E-08	7.05E-07
ENSRNOG00000018336	1.158072214	0.714939823	29.0882938	6.92E-08	7.17E-07
ENSRNOG00000050869	1.47577354	-0.579577904	29.054005	7.04E-08	7.29E-07
ENSRNOG00000022448	0.544901328	4.52069651	29.04078063	7.09E-08	7.33E-07
ENSRNOG00000055508	1.802877581	-0.739279657	29.03493527	7.11E-08	7.35E-07
ENSRNOG00000046871	1.386817054	0.263463791	28.98453029	7.30E-08	7.53E-07
ENSRNOG00000009046	0.556192877	3.475442084	28.96868307	7.36E-08	7.58E-07
ENSRNOG00000014297	0.368778528	5.61399097	28.95519983	7.41E-08	7.63E-07
ENSRNOG00000030445	0.506615787	4.703747657	28.95438909	7.41E-08	7.63E-07
ENSRNOG00000057228	0.852344692	0.716960507	28.9337368	7.49E-08	7.71E-07
ENSRNOG00000027940	0.976083951	5.107091878	28.90878494	7.59E-08	7.80E-07
ENSRNOG00000042252	0.947468179	0.509926855	28.90923071	7.59E-08	7.80E-07
ENSRNOG00000014904	1.737719333	-1.321381224	28.88862895	7.67E-08	7.87E-07
ENSRNOG00000060407	0.719734749	5.694335883	28.87640237	7.71E-08	7.92E-07
ENSRNOG00000019492	0.957689305	4.202013709	28.86921268	7.74E-08	7.94E-07
ENSRNOG00000013970	1.207731267	-0.485888955	28.83645394	7.88E-08	8.06E-07
ENSRNOG00000001235	0.602225081	7.789084211	28.82985148	7.90E-08	8.08E-07
ENSRNOG00000019590	0.357382472	6.271239035	28.80410454	8.01E-08	8.19E-07
ENSRNOG00000020871	0.76963772	4.42096288	28.80051906	8.02E-08	8.20E-07
ENSRNOG00000032487	0.763385263	3.049500539	28.79681437	8.04E-08	8.20E-07
ENSRNOG00000000133	0.390200823	4.906530399	28.79232185	8.06E-08	8.21E-07
ENSRNOG00000020594	0.911262659	1.883551227	28.69633225	8.47E-08	8.61E-07
ENSRNOG00000055408	0.681637187	2.137050516	28.68615263	8.51E-08	8.64E-07
ENSRNOG00000045838	0.603959476	3.032425064	28.66557226	8.60E-08	8.72E-07
ENSRNOG00000059840	0.836269229	4.761051494	28.65859939	8.63E-08	8.73E-07
ENSRNOG00000020390	0.615021675	3.948855808	28.61873848	8.81E-08	8.90E-07
ENSRNOG00000060603	0.625711625	3.849333909	28.59940236	8.90E-08	8.98E-07
ENSRNOG00000020989	0.599226447	3.611478944	28.57792849	9.00E-08	9.07E-07
ENSRNOG00000009329	0.547682611	6.504340713	28.57767615	9.00E-08	9.07E-07
ENSRNOG00000030654	0.440247608	5.709814172	28.57542573	9.01E-08	9.07E-07
ENSRNOG00000007728	0.509845522	2.23148782	28.55662429	9.10E-08	9.15E-07

ENSRNOG00000025497	0.643209952	3.023989393	28.4831554	9.45E-08	9.49E-07
ENSRNOG00000017431	0.422554613	7.608275906	28.42258525	9.75E-08	9.77E-07
ENSRNOG00000048915	0.729142813	3.467096166	28.41953773	9.77E-08	9.78E-07
ENSRNOG00000011093	0.504853204	3.627696531	28.39666495	9.88E-08	9.89E-07
ENSRNOG00000003186	0.441488467	6.022889289	28.37614614	9.99E-08	9.97E-07
ENSRNOG00000014626	0.52970477	4.904076648	28.36865577	1.00E-07	1.00E-06
ENSRNOG00000038087	1.372349009	0.529866752	28.3537785	1.01E-07	1.01E-06
ENSRNOG00000060146	1.081726051	0.32836366	28.33870121	1.02E-07	1.01E-06
ENSRNOG00000011967	0.684959398	3.541919424	28.32901628	1.02E-07	1.02E-06
ENSRNOG00000019930	0.432180268	5.905929562	28.30375175	1.04E-07	1.03E-06
ENSRNOG00000010448	0.489199129	4.837806934	28.28816822	1.05E-07	1.04E-06
ENSRNOG00000001795	0.496898741	5.449727383	28.27385489	1.05E-07	1.04E-06
ENSRNOG00000014367	1.032982168	4.594104557	28.24597188	1.07E-07	1.06E-06
ENSRNOG00000036684	0.558040893	5.134356868	28.22341568	1.08E-07	1.07E-06
ENSRNOG00000029644	0.773765632	4.273421454	28.13189869	1.13E-07	1.12E-06
ENSRNOG00000061004	0.74015567	4.419036931	28.11564023	1.14E-07	1.13E-06
ENSRNOG00000020703	0.652403127	4.893947757	28.11374796	1.14E-07	1.13E-06
ENSRNOG00000015845	0.403433606	5.216043654	28.07259003	1.17E-07	1.15E-06
ENSRNOG00000047052	0.700530664	3.987001119	28.06121397	1.18E-07	1.16E-06
ENSRNOG00000005933	0.530966877	2.869408355	28.05985514	1.18E-07	1.16E-06
ENSRNOG00000023589	0.334666594	5.761289502	28.05354155	1.18E-07	1.16E-06
ENSRNOG00000005840	0.602939116	3.684218399	28.04990885	1.18E-07	1.16E-06
ENSRNOG00000014268	0.713170939	8.209530032	28.03618266	1.19E-07	1.17E-06
ENSRNOG00000021318	0.390884908	7.752853502	28.03320595	1.19E-07	1.17E-06
ENSRNOG00000007387	0.660721526	5.636135728	27.98511986	1.22E-07	1.20E-06
ENSRNOG00000025060	1.337478109	-0.648590299	27.97463266	1.23E-07	1.20E-06
ENSRNOG00000001519	1.425133249	0.911225301	27.9368157	1.25E-07	1.22E-06
ENSRNOG00000021231	1.087632049	6.363928561	27.91686205	1.27E-07	1.23E-06
ENSRNOG00000025669	0.848153462	2.228139061	27.90647768	1.27E-07	1.24E-06
ENSRNOG00000054603	0.461808134	6.365275763	27.82733986	1.33E-07	1.29E-06
ENSRNOG00000028629	0.476627895	6.79127695	27.77633259	1.36E-07	1.32E-06
ENSRNOG00000007261	0.827440166	1.012642791	27.77149503	1.37E-07	1.32E-06
ENSRNOG00000037165	0.449702768	5.316090841	27.74591654	1.38E-07	1.34E-06
ENSRNOG00000011572	0.414480883	8.653185976	27.74557147	1.38E-07	1.34E-06
ENSRNOG00000028628	0.727384073	3.051343239	27.67002405	1.44E-07	1.39E-06
ENSRNOG00000017212	0.759028201	4.305501231	27.66985944	1.44E-07	1.39E-06
ENSRNOG00000010607	0.888682055	5.312550269	27.56197794	1.52E-07	1.46E-06
ENSRNOG00000022694	1.060655819	3.765054158	27.54015792	1.54E-07	1.48E-06
ENSRNOG00000022289	0.449168543	5.24533097	27.5337704	1.54E-07	1.48E-06
ENSRNOG00000000527	0.514867722	8.349471626	27.52241983	1.55E-07	1.49E-06
ENSRNOG00000029194	0.326902007	6.382257667	27.48884102	1.58E-07	1.51E-06
ENSRNOG00000000503	0.843294211	3.626604253	27.48497131	1.58E-07	1.52E-06
ENSRNOG00000003109	0.678443547	2.162597161	27.47319807	1.59E-07	1.52E-06
ENSRNOG00000019372	0.53304346	6.475528461	27.44445892	1.62E-07	1.55E-06
ENSRNOG00000008530	0.524044976	4.643814485	27.44187593	1.62E-07	1.55E-06
ENSRNOG00000009960	0.351163805	6.49151867	27.42485635	1.63E-07	1.56E-06
ENSRNOG00000045555	1.675242469	1.198147586	27.40940406	1.65E-07	1.57E-06
ENSRNOG00000042029	0.874701523	2.934034994	27.39654463	1.66E-07	1.58E-06
ENSRNOG00000003267	0.666658707	3.620064231	27.39273157	1.66E-07	1.58E-06
ENSRNOG00000009253	0.742812486	3.077852916	27.38962265	1.66E-07	1.58E-06
ENSRNOG00000050073	0.989252478	0.366059821	27.38723895	1.67E-07	1.58E-06
ENSRNOG00000049142	0.508665581	5.792907346	27.38114595	1.67E-07	1.59E-06
ENSRNOG00000009473	0.493342917	4.136059403	27.36928266	1.68E-07	1.60E-06
ENSRNOG00000057031	0.959445033	2.192988486	27.31428176	1.73E-07	1.64E-06
ENSRNOG00000018784	0.670756764	7.212470847	27.31543002	1.73E-07	1.64E-06
ENSRNOG00000021548	0.449052632	3.068952322	27.29101853	1.75E-07	1.65E-06
ENSRNOG00000025142	1.008252079	2.638040627	27.28162959	1.76E-07	1.66E-06
ENSRNOG00000014161	0.390798838	4.047474571	27.28222595	1.76E-07	1.66E-06
ENSRNOG00000025244	0.369045428	7.506296228	27.26898946	1.77E-07	1.67E-06
ENSRNOG00000016617	0.77918517	1.968618639	27.22751074	1.81E-07	1.70E-06
ENSRNOG00000008857	0.514663509	3.68199039	27.21248445	1.82E-07	1.72E-06
ENSRNOG00000058077	0.53775549	3.246863467	27.17476957	1.86E-07	1.75E-06
ENSRNOG00000017708	0.346400751	6.208230821	27.15644875	1.88E-07	1.76E-06
ENSRNOG00000020658	0.496245715	5.294057304	27.15091244	1.88E-07	1.77E-06
ENSRNOG00000006970	0.750585812	1.532984755	27.12949675	1.90E-07	1.78E-06
ENSRNOG00000025679	0.547754115	4.907108281	27.09228932	1.94E-07	1.82E-06
ENSRNOG00000010720	1.342329314	-0.146630833	27.08671136	1.95E-07	1.82E-06
ENSRNOG00000032261	0.536287766	3.859260498	27.0414732	1.99E-07	1.86E-06
ENSRNOG00000007033	0.457003108	5.896384762	26.98023296	2.06E-07	1.92E-06
ENSRNOG00000031273	1.253316735	-0.404820266	26.97564597	2.06E-07	1.92E-06
ENSRNOG00000026328	0.514372405	2.781902974	26.96053689	2.08E-07	1.93E-06
ENSRNOG00000001083	0.315356283	4.81976973	26.9365157	2.10E-07	1.95E-06
ENSRNOG00000018416	0.663156262	6.050513787	26.88629434	2.16E-07	2.00E-06
ENSRNOG00000052405	0.58406063	4.183793367	26.88503341	2.16E-07	2.00E-06
ENSRNOG00000025731	0.70469359	1.543583092	26.88219965	2.16E-07	2.00E-06
ENSRNOG00000061099	1.243140305	3.286190382	26.87217302	2.17E-07	2.01E-06
ENSRNOG00000045524	0.534157461	4.491211172	26.83243447	2.22E-07	2.05E-06
ENSRNOG00000017069	0.407415501	5.152278229	26.82855417	2.22E-07	2.05E-06
ENSRNOG00000019812	0.699441146	4.478996904	26.81715436	2.24E-07	2.06E-06
ENSRNOG00000020982	0.57219372	6.94287762	26.81621829	2.24E-07	2.06E-06
ENSRNOG00000053469	0.678884331	2.362469304	26.80511914	2.25E-07	2.07E-06
ENSRNOG00000004050	0.459134635	5.357145968	26.80357831	2.25E-07	2.07E-06
ENSRNOG00000020230	0.549364612	3.380795207	26.80117808	2.25E-07	2.07E-06
ENSRNOG00000008885	0.451703722	3.710468663	26.77786687	2.28E-07	2.09E-06
ENSRNOG00000002627	0.343936899	5.805506497	26.7769074	2.28E-07	2.09E-06
ENSRNOG00000002919	0.834098278	7.884621116	26.7420836	2.33E-07	2.13E-06
ENSRNOG00000016203	0.791493726	1.150739331	26.7318106	2.34E-07	2.14E-06
ENSRNOG00000036699	0.56510484	3.157268136	26.72406089	2.35E-07	2.15E-06
ENSRNOG00000019987	1.018999752	1.371379095	26.71689761	2.36E-07	2.15E-06
ENSRNOG00000043288	0.790223698	0.275484796	26.7002125	2.38E-07	2.17E-06
ENSRNOG00000017949	0.557908387	3.068747641	26.69732351	2.38E-07	2.17E-06
ENSRNOG00000005298	0.599449601	4.136529296	26.69359549	2.38E-07	2.17E-06
ENSRNOG00000058478	0.368753671	8.158448196	26.68763673	2.39E-07	2.18E-06
ENSRNOG00000031420	0.490746087	3.493261082	26.63647077	2.46E-07	2.23E-06
ENSRNOG00000037638	0.418210235	4.294459108	26.56382231	2.55E-07	2.31E-06
ENSRNOG00000017704	0.651727018	3.565988575	26.55727154	2.56E-07	2.31E-06
ENSRNOG00000019918	0.426054442	4.519994796	26.54453403	2.58E-07	2.33E-06
ENSRNOG00000021007	0.485568172	3.782032288	26.50548596	2.63E-07	2.37E-06
ENSRNOG00000001861	0.858494807	2.814388586	26.49331821	2.64E-07	2.38E-06

ENSRNOG00000054130	1.409609491	-0.949604209	26.48290578	2.66E-07	2.39E-06
ENSRNOG00000015503	0.646103804	3.517491229	26.46677295	2.68E-07	2.41E-06
ENSRNOG00000017456	0.579200679	3.506502676	26.4546	2.70E-07	2.43E-06
ENSRNOG00000009300	0.760596127	1.329316042	26.3631088	2.83E-07	2.54E-06
ENSRNOG00000054033	0.532453009	5.909388016	26.32676641	2.88E-07	2.58E-06
ENSRNOG00000050946	0.689001614	2.424688208	26.31976356	2.89E-07	2.59E-06
ENSRNOG00000020880	0.725346883	3.539924441	26.31829336	2.90E-07	2.59E-06
ENSRNOG00000011026	0.576721323	4.326696792	26.27130711	2.97E-07	2.65E-06
ENSRNOG00000000464	0.531681539	5.513303226	26.23502144	3.02E-07	2.70E-06
ENSRNOG00000008564	0.451956473	4.99555413	26.21032991	3.06E-07	2.73E-06
ENSRNOG00000024278	0.479039236	4.184563288	26.20236431	3.07E-07	2.74E-06
ENSRNOG00000007869	0.646281769	6.663690638	26.12626449	3.20E-07	2.84E-06
ENSRNOG00000021118	0.392845672	6.428269155	26.11518727	3.22E-07	2.85E-06
ENSRNOG00000050543	0.327738772	6.006820591	26.10172219	3.24E-07	2.87E-06
ENSRNOG00000049959	1.062541858	3.284868898	26.06007793	3.31E-07	2.93E-06
ENSRNOG00000060410	0.610014214	5.83401107	26.03777037	3.35E-07	2.96E-06
ENSRNOG00000020848	0.534165325	4.313773137	25.96086034	3.48E-07	3.08E-06
ENSRNOG00000061147	0.576967551	2.1018354	25.96087939	3.48E-07	3.08E-06
ENSRNOG00000016960	0.440096704	4.573584902	25.93889239	3.52E-07	3.11E-06
ENSRNOG00000031950	0.674257589	1.985397675	25.93091315	3.54E-07	3.12E-06
ENSRNOG00000001344	0.600917499	5.735597339	25.93029685	3.54E-07	3.12E-06
ENSRNOG00000001313	0.498637884	5.004286966	25.92286109	3.55E-07	3.13E-06
ENSRNOG00000011352	0.541279874	4.707965557	25.92140247	3.56E-07	3.13E-06
ENSRNOG00000014595	0.315919488	4.242201985	25.91564096	3.57E-07	3.13E-06
ENSRNOG00000007582	0.512945326	3.423657064	25.90962428	3.58E-07	3.14E-06
ENSRNOG00000012874	0.636597988	5.811263412	25.90407196	3.59E-07	3.15E-06
ENSRNOG00000023768	0.411944719	4.371931475	25.90283903	3.59E-07	3.15E-06
ENSRNOG00000022248	1.3845437	7.411435806	25.89967676	3.60E-07	3.15E-06
ENSRNOG00000057658	0.887381497	0.537982256	25.88954148	3.62E-07	3.17E-06
ENSRNOG00000016683	0.472845388	3.034696663	25.8882151	3.62E-07	3.17E-06
ENSRNOG00000021129	0.404157876	5.240786767	25.87825437	3.64E-07	3.18E-06
ENSRNOG00000019177	0.419563414	6.945710839	25.86058378	3.67E-07	3.21E-06
ENSRNOG00000028426	0.362320916	7.287639547	25.8411468	3.71E-07	3.24E-06
ENSRNOG00000000151	1.054379361	2.173620473	25.81412728	3.76E-07	3.28E-06
ENSRNOG00000052357	1.496759907	1.002574973	25.81084921	3.77E-07	3.29E-06
ENSRNOG00000014420	1.428200057	2.156514249	25.73440246	3.92E-07	3.41E-06
ENSRNOG00000019915	0.618372887	4.824821041	25.72748078	3.93E-07	3.42E-06
ENSRNOG00000058157	1.048896409	-0.021969177	25.69153311	4.01E-07	3.48E-06
ENSRNOG00000007681	0.476825409	4.582988131	25.67425405	4.04E-07	3.51E-06
ENSRNOG00000024809	0.663711215	3.167512085	25.64981743	4.09E-07	3.55E-06
ENSRNOG00000049751	1.240679744	-0.066447056	25.62351461	4.15E-07	3.59E-06
ENSRNOG00000007284	0.503827257	6.722454188	25.54676174	4.32E-07	3.73E-06
ENSRNOG00000026116	0.62805491	2.808424787	25.52835645	4.36E-07	3.76E-06
ENSRNOG00000054400	0.40664615	4.991563363	25.50133872	4.42E-07	3.81E-06
ENSRNOG00000049107	0.950975889	1.384742182	25.48025458	4.47E-07	3.85E-06
ENSRNOG00000007993	0.789297393	1.656745506	25.43867224	4.57E-07	3.93E-06
ENSRNOG00000019207	0.805102628	7.492382447	25.43302794	4.58E-07	3.93E-06
ENSRNOG00000023386	1.117631839	-0.464682861	25.39758338	4.67E-07	4.00E-06
ENSRNOG00000000501	0.592910429	4.91400256	25.32335694	4.85E-07	4.15E-06
ENSRNOG00000024264	0.519721485	3.88203903	25.3203215	4.86E-07	4.16E-06
ENSRNOG00000029698	0.690145962	4.294044604	25.21754865	5.12E-07	4.37E-06
ENSRNOG00000018998	0.482254277	5.212110755	25.21038558	5.14E-07	4.38E-06
ENSRNOG00000028733	0.559846955	7.359838072	25.20547074	5.15E-07	4.39E-06
ENSRNOG00000049402	0.471591463	5.637608668	25.13871176	5.34E-07	4.53E-06
ENSRNOG00000021161	0.689588963	2.589580771	25.12346287	5.38E-07	4.57E-06
ENSRNOG00000010457	0.570245256	3.489245211	25.12184689	5.38E-07	4.57E-06
ENSRNOG00000052600	0.854316454	1.76890971	25.11565264	5.40E-07	4.58E-06
ENSRNOG00000019693	0.308535597	5.354725254	25.11454306	5.40E-07	4.58E-06
ENSRNOG00000037709	1.422205001	-0.69836997	25.09250086	5.46E-07	4.63E-06
ENSRNOG00000051507	0.933550667	3.27762465	25.063393	5.55E-07	4.69E-06
ENSRNOG00000008790	0.465494956	4.826117365	25.06197477	5.55E-07	4.69E-06
ENSRNOG00000011667	0.542963055	5.344649452	25.06037916	5.56E-07	4.69E-06
ENSRNOG00000001143	0.37745742	6.610773172	25.05888504	5.56E-07	4.69E-06
ENSRNOG00000020339	0.866143407	6.94431477	25.02980483	5.65E-07	4.76E-06
ENSRNOG00000023496	1.001152867	3.098763678	25.02264145	5.67E-07	4.77E-06
ENSRNOG00000011170	0.529016929	3.830637367	24.99911075	5.74E-07	4.83E-06
ENSRNOG00000015376	0.97295698	3.128169086	24.98644468	5.77E-07	4.85E-06
ENSRNOG00000021245	0.546646749	5.612891115	24.96308743	5.84E-07	4.90E-06
ENSRNOG00000031368	0.572524495	2.970015069	24.95990719	5.85E-07	4.91E-06
ENSRNOG00000011803	0.657079888	3.299840308	24.9387802	5.92E-07	4.95E-06
ENSRNOG00000006600	0.642860654	3.408687073	24.93812252	5.92E-07	4.95E-06
ENSRNOG00000012348	0.545891754	3.134457422	24.9323806	5.94E-07	4.97E-06
ENSRNOG00000017660	0.482934025	6.754703577	24.89434317	6.06E-07	5.06E-06
ENSRNOG00000054459	1.454470928	-0.367001119	24.87122658	6.13E-07	5.12E-06
ENSRNOG00000022983	0.665453772	2.563365517	24.86678472	6.14E-07	5.12E-06
ENSRNOG00000006178	0.359809164	8.340295538	24.83144309	6.26E-07	5.21E-06
ENSRNOG00000010883	0.817930815	1.789519551	24.82480833	6.28E-07	5.23E-06
ENSRNOG00000016109	0.376230342	6.285060994	24.82538647	6.28E-07	5.23E-06
ENSRNOG00000054426	0.749918511	2.107138487	24.80202834	6.35E-07	5.28E-06
ENSRNOG00000006961	0.334573175	4.508416764	24.80282779	6.35E-07	5.28E-06
ENSRNOG00000016204	0.706781051	3.40554855	24.79504563	6.38E-07	5.30E-06
ENSRNOG00000011132	0.633140467	4.569870679	24.7719319	6.45E-07	5.36E-06
ENSRNOG00000007158	0.762166847	2.11631095	24.66519643	6.82E-07	5.65E-06
ENSRNOG00000014526	0.501507919	3.719046145	24.6540801	6.86E-07	5.68E-06
ENSRNOG00000049591	1.426816237	-1.005301581	24.64220311	6.90E-07	5.71E-06
ENSRNOG00000009596	1.32833452	-0.055000032	24.59336422	7.08E-07	5.85E-06
ENSRNOG00000000967	0.622956118	4.393083406	24.59213407	7.08E-07	5.85E-06
ENSRNOG00000057080	0.693759925	2.504736393	24.58623685	7.11E-07	5.87E-06
ENSRNOG00000018835	0.419786086	4.836830163	24.5770278	7.14E-07	5.89E-06
ENSRNOG00000008056	0.831006109	0.821620899	24.55912443	7.21E-07	5.94E-06
ENSRNOG00000015393	0.531320899	6.863809773	24.542827	7.27E-07	5.98E-06
ENSRNOG00000032656	0.371609039	5.443239362	24.5112384	7.39E-07	6.08E-06
ENSRNOG00000033915	0.465208505	4.234110774	24.50085864	7.43E-07	6.11E-06
ENSRNOG00000047089	0.658211439	2.772860383	24.49715095	7.44E-07	6.11E-06
ENSRNOG00000020579	0.85179655	1.223064625	24.47497282	7.53E-07	6.18E-06
ENSRNOG00000020269	0.295603804	6.265804819	24.46761069	7.56E-07	6.19E-06
ENSRNOG00000057186	0.309695349	4.00977098	24.45081678	7.62E-07	6.24E-06
ENSRNOG00000023458	0.556798469	5.647306145	24.44746327	7.64E-07	6.25E-06
ENSRNOG00000000921	0.497715937	7.328336717	24.43056045	7.70E-07	6.30E-06
ENSRNOG00000046836	0.467127832	4.478780704	24.41797212	7.75E-07	6.33E-06

ENSRNOG00000047491	0.770512488	5.285696761	24.41746085	7.76E-07	6.33E-06
ENSRNOG00000019848	1.224675095	2.105967045	24.40268687	7.82E-07	6.38E-06
ENSRNOG00000008996	0.549508579	2.866478052	24.3394862	8.08E-07	6.57E-06
ENSRNOG00000010849	0.423326514	4.351053838	24.28191803	8.32E-07	6.76E-06
ENSRNOG00000010407	1.715113624	-1.143642865	24.27868455	8.34E-07	6.77E-06
ENSRNOG00000021174	0.728216511	2.843328255	24.27599266	8.35E-07	6.77E-06
ENSRNOG00000017654	0.501965252	3.419546574	24.2651115	8.39E-07	6.80E-06
ENSRNOG00000016362	1.040246175	1.422739948	24.26389198	8.40E-07	6.80E-06
ENSRNOG00000019328	0.619214715	6.328608494	24.24681305	8.47E-07	6.85E-06
ENSRNOG00000000559	1.480681396	-0.316203561	24.22503556	8.57E-07	6.92E-06
ENSRNOG00000003504	0.451751335	4.731566209	24.2225962	8.58E-07	6.93E-06
ENSRNOG00000000695	0.539752641	3.116477693	24.19624576	8.70E-07	7.02E-06
ENSRNOG00000058497	0.627147392	2.415329618	24.16721664	8.83E-07	7.11E-06
ENSRNOG00000022267	1.21784766	3.940922291	24.15241021	8.90E-07	7.16E-06
ENSRNOG00000042384	0.795235599	2.190458244	24.14598877	8.93E-07	7.18E-06
ENSRNOG00000005116	1.214536332	0.675137958	24.13738984	8.97E-07	7.21E-06
ENSRNOG00000009066	0.536415424	8.45754922	24.12380787	9.03E-07	7.25E-06
ENSRNOG00000057502	0.858313861	2.926113431	24.08007518	9.24E-07	7.41E-06
ENSRNOG00000046308	1.196204535	-0.537919874	24.0520947	9.38E-07	7.51E-06
ENSRNOG00000008471	0.555513309	5.719741221	24.03056998	9.48E-07	7.59E-06
ENSRNOG00000006460	0.628046646	2.501516181	24.01237936	9.57E-07	7.65E-06
ENSRNOG00000024526	1.090352373	-0.122393152	24.01129144	9.58E-07	7.66E-06
ENSRNOG00000028166	0.669298322	3.800980548	24.00403436	9.61E-07	7.67E-06
ENSRNOG00000009495	0.341702858	6.415089005	23.97449637	9.76E-07	7.79E-06
ENSRNOG00000020229	0.532956433	2.556051217	23.974021	9.76E-07	7.79E-06
ENSRNOG00000005806	1.696662354	-0.007509337	23.97110234	9.78E-07	7.79E-06
ENSRNOG00000001126	0.47073559	5.255702954	23.96323738	9.82E-07	7.82E-06
ENSRNOG00000046624	1.417961686	-1.011318594	23.9412101	9.93E-07	7.91E-06
ENSRNOG00000014007	0.9137973	3.554120834	23.91699153	1.01E-06	8.00E-06
ENSRNOG00000011009	0.45070877	3.996141968	23.91553785	1.01E-06	8.00E-06
ENSRNOG00000020205	0.39536485	7.15604934	23.90897926	1.01E-06	8.02E-06
ENSRNOG00000017003	0.633444725	6.207775007	23.87094353	1.03E-06	8.18E-06
ENSRNOG00000015904	0.70845816	3.568611209	23.85368274	1.04E-06	8.24E-06
ENSRNOG00000008065	0.610548565	5.790555599	23.85146429	1.04E-06	8.24E-06
ENSRNOG00000052539	0.428150459	5.525359856	23.7881565	1.08E-06	8.51E-06
ENSRNOG00000012562	0.940953703	1.87422011	23.7722162	1.08E-06	8.57E-06
ENSRNOG00000052945	0.598993698	2.867225837	23.73231576	1.11E-06	8.74E-06
ENSRNOG00000042838	1.626512695	2.997533534	23.68827398	1.13E-06	8.93E-06
ENSRNOG00000001654	0.411131905	4.513970708	23.67919157	1.14E-06	8.97E-06
ENSRNOG00000008686	0.938947882	1.007811728	23.67720427	1.14E-06	8.97E-06
ENSRNOG00000002950	1.121907964	0.080305433	23.6747169	1.14E-06	8.98E-06
ENSRNOG00000012927	0.48310818	3.579081037	23.61341822	1.18E-06	9.24E-06
ENSRNOG00000002917	0.713770372	2.27230297	23.57658654	1.20E-06	9.42E-06
ENSRNOG00000001191	0.347427349	4.279782098	23.56970132	1.20E-06	9.45E-06
ENSRNOG00000024036	0.620269866	3.457030855	23.55961977	1.21E-06	9.48E-06
ENSRNOG00000014235	0.430582568	4.502787376	23.54843642	1.22E-06	9.53E-06
ENSRNOG00000033835	0.423077744	8.736372692	23.54179815	1.22E-06	9.56E-06
ENSRNOG00000010880	1.010350646	1.540257213	23.51293905	1.24E-06	9.70E-06
ENSRNOG00000025518	0.822592452	5.34462707	23.48649835	1.26E-06	9.81E-06
ENSRNOG00000050233	0.712121009	2.460222852	23.4821009	1.26E-06	9.82E-06
ENSRNOG00000022173	0.528565118	5.266673738	23.47283811	1.27E-06	9.87E-06
ENSRNOG00000016033	0.489137198	3.713966368	23.46929904	1.27E-06	9.88E-06
ENSRNOG00000046975	0.517834319	7.68353065	23.45970827	1.28E-06	9.93E-06
ENSRNOG00000000977	0.562327762	5.246204585	23.45595132	1.28E-06	9.94E-06
ENSRNOG00000022540	0.734135943	2.620681019	23.42765055	1.30E-06	1.01E-05
ENSRNOG00000001374	0.766090259	4.961699683	23.37173358	1.34E-06	1.04E-05
ENSRNOG00000001707	0.786650397	4.963382901	23.35965916	1.34E-06	1.04E-05
ENSRNOG00000016256	0.402132543	6.211351505	23.35869121	1.34E-06	1.04E-05
ENSRNOG00000046307	0.659018444	1.939899387	23.35191378	1.35E-06	1.04E-05
ENSRNOG00000007400	0.493893736	6.767821298	23.34988063	1.35E-06	1.04E-05
ENSRNOG00000017295	0.529962129	6.877138922	23.32294617	1.37E-06	1.06E-05
ENSRNOG00000014678	0.909479634	0.338820766	23.32075838	1.37E-06	1.06E-05
ENSRNOG00000000480	0.545642337	4.918791408	23.29579142	1.39E-06	1.07E-05
ENSRNOG00000057292	1.220271805	-0.603515113	23.26900246	1.41E-06	1.09E-05
ENSRNOG00000004063	0.67019459	2.018184709	23.26638345	1.41E-06	1.09E-05
ENSRNOG00000010629	0.483381553	2.832665717	23.25145427	1.42E-06	1.09E-05
ENSRNOG00000010813	0.479743851	3.931151813	23.23097225	1.44E-06	1.10E-05
ENSRNOG00000012804	0.688563946	3.928541453	23.22511778	1.44E-06	1.11E-05
ENSRNOG00000000185	0.581575783	3.073747897	23.22153848	1.44E-06	1.11E-05
ENSRNOG00000026114	0.674127942	2.256006231	23.21367968	1.45E-06	1.11E-05
ENSRNOG00000013265	0.591655814	2.904468735	23.20706291	1.45E-06	1.11E-05
ENSRNOG00000019651	0.457131538	4.138933599	23.19341821	1.46E-06	1.12E-05
ENSRNOG00000003228	0.627683123	5.739014676	23.16685311	1.49E-06	1.14E-05
ENSRNOG00000013014	0.919302906	0.984435363	23.14337257	1.50E-06	1.15E-05
ENSRNOG00000016603	0.500539605	5.496091914	23.1162407	1.52E-06	1.16E-05
ENSRNOG00000000490	0.576911278	4.547004498	23.11021047	1.53E-06	1.17E-05
ENSRNOG00000021671	0.68808772	3.472498691	23.09651394	1.54E-06	1.17E-05
ENSRNOG00000033912	0.608920375	5.672154754	23.06755761	1.56E-06	1.19E-05
ENSRNOG00000019240	0.600546403	6.061758123	23.06347795	1.57E-06	1.19E-05
ENSRNOG00000015049	0.765067197	4.542744896	23.03961285	1.59E-06	1.21E-05
ENSRNOG00000019522	0.415839611	4.441815606	23.03529378	1.59E-06	1.21E-05
ENSRNOG00000021521	0.961056292	0.784565073	23.03166552	1.59E-06	1.21E-05
ENSRNOG00000016863	0.450004949	8.212855402	22.97011737	1.65E-06	1.25E-05
ENSRNOG00000014890	0.689152262	4.02241879	22.95704205	1.66E-06	1.25E-05
ENSRNOG00000031789	0.391317027	7.101345039	22.9515953	1.66E-06	1.26E-05
ENSRNOG00000006720	0.342972394	5.230128655	22.94713034	1.67E-06	1.26E-05
ENSRNOG00000006004	0.324188921	5.844923748	22.84218251	1.76E-06	1.32E-05
ENSRNOG00000050998	1.116203995	0.213954182	22.82725187	1.77E-06	1.33E-05
ENSRNOG00000010959	0.554521142	2.387302305	22.770543	1.83E-06	1.37E-05
ENSRNOG00000057284	0.530054306	5.768465462	22.76822551	1.83E-06	1.37E-05
ENSRNOG00000026447	0.834748136	3.103845494	22.76835729	1.83E-06	1.37E-05
ENSRNOG00000024839	0.488969732	2.603596966	22.73881209	1.86E-06	1.39E-05
ENSRNOG00000032293	0.363305917	4.647735048	22.62801058	1.97E-06	1.47E-05
ENSRNOG00000022852	0.630815211	1.671995202	22.61556834	1.98E-06	1.48E-05
ENSRNOG00000001163	0.43471153	3.056750158	22.57206757	2.02E-06	1.51E-05
ENSRNOG00000004155	0.696815343	4.804190677	22.56908631	2.03E-06	1.51E-05
ENSRNOG00000032639	1.256771856	0.650834522	22.54658769	2.05E-06	1.53E-05
ENSRNOG00000019555	0.626529511	4.546568339	22.54466977	2.05E-06	1.53E-05
ENSRNOG00000022054	0.587309671	5.389424763	22.51884638	2.08E-06	1.55E-05
ENSRNOG00000012420	0.709561928	4.958596882	22.50967608	2.09E-06	1.55E-05
ENSRNOG00000009421	0.333005544	6.006388162	22.49455428	2.11E-06	1.57E-05

ENSRNOG00000028904	0.44185128	4.746371867	22.48696662	2.12E-06	1.57E-05
ENSRNOG00000009348	0.602836051	2.969213021	22.46912928	2.14E-06	1.58E-05
ENSRNOG00000019799	0.572819102	6.727283849	22.46454448	2.14E-06	1.59E-05
ENSRNOG00000024266	0.503150369	2.575180364	22.45898828	2.15E-06	1.59E-05
ENSRNOG00000003129	0.359855975	3.689806958	22.44120638	2.17E-06	1.60E-05
ENSRNOG00000018271	0.544598339	4.419033813	22.43735836	2.17E-06	1.61E-05
ENSRNOG00000014647	0.657956708	4.07906184	22.42601631	2.18E-06	1.61E-05
ENSRNOG00000030127	0.525334473	6.72937731	22.40911578	2.20E-06	1.63E-05
ENSRNOG00000023023	0.563342044	2.755183404	22.40795048	2.20E-06	1.63E-05
ENSRNOG00000028390	0.659304576	2.094215591	22.3813438	2.24E-06	1.65E-05
ENSRNOG00000015576	0.359893757	5.004575723	22.37402446	2.24E-06	1.65E-05
ENSRNOG00000049336	0.434850676	4.2689296	22.35788809	2.26E-06	1.67E-05
ENSRNOG00000005874	0.780729582	2.598994297	22.34998142	2.27E-06	1.67E-05
ENSRNOG00000006569	0.299199992	5.729711286	22.31132348	2.32E-06	1.71E-05
ENSRNOG00000050714	0.800018179	4.237215361	22.2898144	2.34E-06	1.72E-05
ENSRNOG00000056559	0.591627355	2.167331252	22.28496747	2.35E-06	1.73E-05
ENSRNOG00000052785	0.460062119	3.054162996	22.26981783	2.37E-06	1.74E-05
ENSRNOG00000003999	0.395151613	5.909360635	22.24938176	2.39E-06	1.75E-05
ENSRNOG00000019022	0.614224347	1.815229241	22.2306346	2.42E-06	1.77E-05
ENSRNOG00000047625	0.492916655	4.234604135	22.22773659	2.42E-06	1.77E-05
ENSRNOG00000003835	0.333761181	5.199012093	22.22126658	2.43E-06	1.78E-05
ENSRNOG00000002879	0.631288603	3.803658651	22.21599163	2.44E-06	1.78E-05
ENSRNOG00000014302	1.046623276	6.218134592	22.20769959	2.45E-06	1.79E-05
ENSRNOG00000015448	0.540936415	2.212675487	22.20433389	2.45E-06	1.79E-05
ENSRNOG00000049782	0.993013197	0.758576358	22.19107698	2.47E-06	1.80E-05
ENSRNOG00000002997	0.440226003	5.462586995	22.17430758	2.49E-06	1.81E-05
ENSRNOG00000036693	0.616068735	4.095964138	22.16132505	2.51E-06	1.82E-05
ENSRNOG00000052581	1.617849877	-0.302229912	22.1453418	2.53E-06	1.84E-05
ENSRNOG00000054955	0.811592077	3.817331599	22.13220211	2.55E-06	1.85E-05
ENSRNOG00000018338	0.435641024	4.529193286	22.13277434	2.54E-06	1.85E-05
ENSRNOG00000009863	0.647995117	3.758720346	22.08849761	2.60E-06	1.89E-05
ENSRNOG00000015177	0.559958581	5.211910791	22.07053328	2.63E-06	1.90E-05
ENSRNOG00000048213	0.784611211	3.447437038	22.05073177	2.66E-06	1.92E-05
ENSRNOG00000033979	0.900216324	1.409242719	22.00156468	2.72E-06	1.97E-05
ENSRNOG00000020302	0.641871626	3.979617093	22.00085228	2.73E-06	1.97E-05
ENSRNOG00000001047	0.47323434	4.473916193	21.97252895	2.77E-06	1.99E-05
ENSRNOG00000050210	0.830372715	1.760037963	21.96565338	2.78E-06	2.00E-05
ENSRNOG00000018709	0.384453122	4.617553986	21.96031611	2.78E-06	2.00E-05
ENSRNOG00000052537	1.409125097	-0.775599094	21.94947099	2.80E-06	2.01E-05
ENSRNOG00000015283	0.473105718	2.576758557	21.94451496	2.81E-06	2.02E-05
ENSRNOG00000020694	1.280310457	5.447306941	21.93136936	2.83E-06	2.03E-05
ENSRNOG00000014065	0.679453705	2.948203139	21.91038656	2.86E-06	2.05E-05
ENSRNOG00000008026	1.210822837	1.775797979	21.8999093	2.87E-06	2.06E-05
ENSRNOG00000049937	0.777552854	5.224542169	21.88142444	2.90E-06	2.08E-05
ENSRNOG00000026286	0.548770843	2.533040575	21.87723683	2.91E-06	2.08E-05
ENSRNOG00000012215	0.987205368	0.689782946	21.83950686	2.96E-06	2.12E-05
ENSRNOG00000048193	0.570596143	2.41759389	21.82411051	2.99E-06	2.13E-05
ENSRNOG00000056095	1.018926509	3.871727139	21.8016613	3.02E-06	2.15E-05
ENSRNOG00000001222	0.470009028	4.419755547	21.79189328	3.04E-06	2.16E-05
ENSRNOG00000022893	0.593970156	5.355327557	21.76442567	3.08E-06	2.19E-05
ENSRNOG00000022307	0.443299001	6.044702351	21.75694209	3.09E-06	2.20E-05
ENSRNOG00000054314	0.897806192	3.217024857	21.74647844	3.11E-06	2.21E-05
ENSRNOG00000054008	0.357102161	7.419125008	21.74357039	3.12E-06	2.21E-05
ENSRNOG00000008287	0.531830592	2.426149083	21.73272554	3.13E-06	2.22E-05
ENSRNOG00000045998	0.420070121	5.619008903	21.72719642	3.14E-06	2.22E-05
ENSRNOG00000010821	0.590814948	1.94213441	21.72142917	3.15E-06	2.23E-05
ENSRNOG00000009595	0.519177707	2.945109002	21.70167309	3.19E-06	2.25E-05
ENSRNOG00000059463	0.27246457	5.390670388	21.66536249	3.25E-06	2.29E-05
ENSRNOG00000010653	0.306735091	4.73393138	21.65273306	3.27E-06	2.30E-05
ENSRNOG00000005530	0.529850153	4.787742692	21.64609148	3.28E-06	2.31E-05
ENSRNOG00000000833	0.457222009	4.514948281	21.63831963	3.29E-06	2.32E-05
ENSRNOG00000004604	0.530647969	5.30598812	21.603505	3.35E-06	2.35E-05
ENSRNOG00000052667	1.327970373	-0.833732848	21.59692125	3.36E-06	2.36E-05
ENSRNOG00000011751	0.751293109	9.282097433	21.58877944	3.38E-06	2.37E-05
ENSRNOG00000015184	0.426705021	4.742199449	21.57221085	3.41E-06	2.39E-05
ENSRNOG00000017290	0.415632067	5.608550724	21.56414497	3.42E-06	2.39E-05
ENSRNOG00000003149	0.393282733	4.316410403	21.55733558	3.43E-06	2.40E-05
ENSRNOG00000024813	0.556512492	2.555524392	21.54472381	3.46E-06	2.42E-05
ENSRNOG00000017753	0.374413032	4.212338559	21.54108822	3.46E-06	2.42E-05
ENSRNOG00000014908	0.575015208	4.267414627	21.53584055	3.47E-06	2.43E-05
ENSRNOG00000017197	0.576549738	2.980304813	21.53297462	3.48E-06	2.43E-05
ENSRNOG00000036384	0.623093906	4.191507126	21.50084497	3.54E-06	2.47E-05
ENSRNOG00000049324	1.33298757	-0.948295971	21.49360853	3.55E-06	2.47E-05
ENSRNOG00000005287	1.018813823	0.370294804	21.4929735	3.55E-06	2.47E-05
ENSRNOG00000057732	1.10075339	-0.559228786	21.48189013	3.57E-06	2.49E-05
ENSRNOG00000017285	0.361792827	4.817289643	21.473919	3.59E-06	2.50E-05
ENSRNOG00000006747	0.687388404	5.533825867	21.41819845	3.69E-06	2.56E-05
ENSRNOG00000042101	0.951854591	-0.119631343	21.40622057	3.72E-06	2.58E-05
ENSRNOG00000012950	0.323106075	6.493632223	21.40566521	3.72E-06	2.58E-05
ENSRNOG00000050453	1.027360947	-0.397558028	21.39150399	3.74E-06	2.59E-05
ENSRNOG00000019596	0.644395377	3.571798976	21.38319966	3.76E-06	2.60E-05
ENSRNOG00000011387	0.519430449	3.890827571	21.37598337	3.77E-06	2.61E-05
ENSRNOG00000023972	0.667205858	5.020767763	21.3426092	3.84E-06	2.65E-05
ENSRNOG00000018867	0.976140816	0.008813179	21.30251155	3.92E-06	2.70E-05
ENSRNOG00000002607	0.663442293	3.384354921	21.30397286	3.92E-06	2.70E-05
ENSRNOG00000022283	0.580723242	3.1920008	21.3024556	3.92E-06	2.70E-05
ENSRNOG00000049944	1.214603961	0.595356505	21.296712	3.93E-06	2.71E-05
ENSRNOG00000049965	1.104571197	0.485291499	21.2949161	3.94E-06	2.71E-05
ENSRNOG00000001205	0.287853021	7.85332252	21.29332703	3.94E-06	2.71E-05
ENSRNOG00000005633	0.600118282	2.063936265	21.27896629	3.97E-06	2.73E-05
ENSRNOG00000009195	0.53751207	5.058449327	21.26299109	4.00E-06	2.75E-05
ENSRNOG00000001241	0.347960098	3.880880374	21.24238641	4.05E-06	2.78E-05
ENSRNOG00000056297	0.382419948	3.543793614	21.23052429	4.07E-06	2.79E-05
ENSRNOG00000008577	0.386558348	4.635768413	21.19760451	4.14E-06	2.84E-05
ENSRNOG00000060905	0.602775146	1.638643725	21.17162671	4.20E-06	2.87E-05
ENSRNOG00000014373	0.531597864	3.910596269	21.16711642	4.21E-06	2.88E-05
ENSRNOG00000018434	0.620511135	1.736418595	21.15945581	4.23E-06	2.89E-05
ENSRNOG00000056106	0.268521304	6.359438597	21.15767447	4.23E-06	2.89E-05
ENSRNOG00000045948	1.392517703	1.88150407	21.12156402	4.31E-06	2.94E-05

ENSRNOG00000021247	0.429087267	3.055121134	21.11741349	4.32E-06	2.95E-05
ENSRNOG00000032446	0.421837724	3.353676706	21.10030633	4.36E-06	2.97E-05
ENSRNOG00000001258	0.798279524	4.235563116	21.08943617	4.38E-06	2.99E-05
ENSRNOG00000047653	0.874221046	0.616935521	21.08829394	4.39E-06	2.99E-05
ENSRNOG00000016998	0.659459993	4.606267858	21.06321862	4.44E-06	3.02E-05
ENSRNOG00000046955	0.470867225	7.141644987	21.06090523	4.45E-06	3.02E-05
ENSRNOG00000014191	0.662966912	3.601458918	21.04724909	4.48E-06	3.04E-05
ENSRNOG00000052909	1.867043977	0.27839249	21.04604546	4.48E-06	3.04E-05
ENSRNOG00000051858	1.867043977	0.27839249	21.04604546	4.48E-06	3.04E-05
ENSRNOG00000010776	0.430648844	5.06268329	20.98482973	4.63E-06	3.14E-05
ENSRNOG00000037060	1.781338745	0.449461052	20.95882063	4.69E-06	3.17E-05
ENSRNOG00000010794	0.535656839	3.296059567	20.95438302	4.70E-06	3.18E-05
ENSRNOG00000011542	1.108185723	-0.923441253	20.93732882	4.75E-06	3.20E-05
ENSRNOG00000019506	0.448376707	6.586151308	20.92687146	4.77E-06	3.22E-05
ENSRNOG00000009567	0.35669593	4.983941219	20.8754901	4.90E-06	3.30E-05
ENSRNOG00000020843	0.668897765	6.383753247	20.86960201	4.92E-06	3.31E-05
ENSRNOG00000005148	1.946576029	-0.525929377	20.86704219	4.92E-06	3.31E-05
ENSRNOG00000018090	0.327958102	6.980341069	20.85165472	4.96E-06	3.34E-05
ENSRNOG00000016405	0.84778814	1.791068457	20.85151552	4.96E-06	3.34E-05
ENSRNOG00000013813	0.89558677	1.598944333	20.81175966	5.07E-06	3.40E-05
ENSRNOG00000016272	0.537008676	5.422110466	20.81201131	5.07E-06	3.40E-05
ENSRNOG00000055925	0.350516119	8.233966958	20.78516984	5.14E-06	3.44E-05
ENSRNOG00000024631	0.871665958	2.502624056	20.76943387	5.18E-06	3.47E-05
ENSRNOG00000000231	0.581809286	5.559109562	20.74398106	5.25E-06	3.51E-05
ENSRNOG00000008323	0.422624284	6.177294563	20.74406516	5.25E-06	3.51E-05
ENSRNOG00000030537	0.667512803	2.403412625	20.74011075	5.26E-06	3.51E-05
ENSRNOG00000019264	0.651496327	3.457639935	20.73319018	5.28E-06	3.53E-05
ENSRNOG00000001003	0.359961252	4.116841715	20.70850928	5.35E-06	3.57E-05
ENSRNOG00000001700	0.968660988	4.848848284	20.67153923	5.45E-06	3.63E-05
ENSRNOG00000024602	0.631373304	3.893042921	20.6445099	5.53E-06	3.68E-05
ENSRNOG00000008992	0.48821543	4.895022951	20.62390729	5.59E-06	3.72E-05
ENSRNOG00000008470	1.12747264	-0.280292294	20.6190432	5.60E-06	3.73E-05
ENSRNOG00000037607	1.28720636	0.501199587	20.61244872	5.62E-06	3.74E-05
ENSRNOG00000019741	0.603030282	4.903523218	20.60048148	5.66E-06	3.76E-05
ENSRNOG00000001870	0.306257918	5.746933697	20.59601327	5.67E-06	3.76E-05
ENSRNOG00000033568	0.394866863	7.942212768	20.58028574	5.72E-06	3.79E-05
ENSRNOG00000009370	0.414248371	5.259755488	20.57943166	5.72E-06	3.79E-05
ENSRNOG00000005270	0.811265558	3.530290862	20.54309402	5.83E-06	3.86E-05
ENSRNOG00000002574	0.366285631	4.746409648	20.49179087	5.99E-06	3.96E-05
ENSRNOG00000000988	0.380423613	4.336139067	20.48894605	6.00E-06	3.96E-05
ENSRNOG00000001061	0.584901132	3.700018779	20.48936302	6.00E-06	3.96E-05
ENSRNOG00000061822	1.22154393	-0.693884903	20.48412091	6.01E-06	3.97E-05
ENSRNOG00000002116	1.284665643	0.499341845	20.46765967	6.06E-06	4.00E-05
ENSRNOG00000027498	0.708720226	3.35812935	20.44220815	6.15E-06	4.05E-05
ENSRNOG00000052586	0.87800143	0.139659517	20.42398747	6.20E-06	4.08E-05
ENSRNOG00000019791	0.430476333	5.382507204	20.423155	6.21E-06	4.08E-05
ENSRNOG00000001210	0.387478037	4.516027617	20.42070809	6.22E-06	4.09E-05
ENSRNOG00000013069	1.057344957	-0.561313939	20.40350532	6.27E-06	4.12E-05
ENSRNOG00000019349	0.57982854	3.689427152	20.39011752	6.32E-06	4.14E-05
ENSRNOG00000027880	0.556606657	4.478934338	20.3836008	6.34E-06	4.15E-05
ENSRNOG00000015097	0.324624685	5.259809541	20.37495073	6.37E-06	4.17E-05
ENSRNOG00000020032	0.40013155	4.555677619	20.35000908	6.45E-06	4.22E-05
ENSRNOG00000045737	1.081667981	0.84388025	20.32316936	6.54E-06	4.28E-05
ENSRNOG00000028512	0.590037959	3.410661962	20.30292165	6.61E-06	4.32E-05
ENSRNOG00000013767	0.647886168	2.161972128	20.29708112	6.63E-06	4.33E-05
ENSRNOG00000051120	0.651979958	2.116527628	20.28141556	6.68E-06	4.36E-05
ENSRNOG00000026044	0.489458198	4.509549502	20.26160367	6.75E-06	4.40E-05
ENSRNOG00000060005	0.33750871	3.63660286	20.22220931	6.89E-06	4.49E-05
ENSRNOG00000048851	1.263826794	-0.363691455	20.19468584	6.99E-06	4.55E-05
ENSRNOG00000026425	0.324161854	6.387702735	20.18327381	7.04E-06	4.57E-05
ENSRNOG00000042041	0.801648593	2.164046513	20.17548964	7.07E-06	4.59E-05
ENSRNOG00000037380	0.931761981	0.635744482	20.16015162	7.12E-06	4.62E-05
ENSRNOG00000061474	0.319988718	3.938213034	20.14989126	7.16E-06	4.65E-05
ENSRNOG00000061998	0.960164174	0.609520133	20.13480171	7.22E-06	4.68E-05
ENSRNOG00000057078	0.491375913	5.468700403	20.12972735	7.24E-06	4.69E-05
ENSRNOG00000005483	0.406225015	4.091907533	20.12795757	7.24E-06	4.69E-05
ENSRNOG00000001500	0.480713635	4.388160415	20.09199556	7.38E-06	4.78E-05
ENSRNOG00000046883	0.520444614	4.412657764	20.0913392	7.38E-06	4.78E-05
ENSRNOG00000019861	0.389510457	7.041358973	20.07570783	7.44E-06	4.81E-05
ENSRNOG00000010890	0.51327337	3.832676102	20.05524588	7.52E-06	4.86E-05
ENSRNOG00000005480	0.440200014	4.156005022	20.03780633	7.59E-06	4.90E-05
ENSRNOG00000017434	0.337891727	5.860192307	20.02771492	7.63E-06	4.92E-05
ENSRNOG00000008474	0.361765631	4.31867604	20.02768233	7.63E-06	4.92E-05
ENSRNOG00000008424	0.420051698	4.198598284	20.01459005	7.69E-06	4.95E-05
ENSRNOG00000018797	0.295159123	6.101294942	19.97982709	7.83E-06	5.04E-05
ENSRNOG00000015077	0.411351983	3.78995198	19.95489981	7.93E-06	5.10E-05
ENSRNOG00000054563	0.639069629	3.039365109	19.94848457	7.96E-06	5.11E-05
ENSRNOG00000055850	1.554676869	-0.750694016	19.93690022	8.00E-06	5.14E-05
ENSRNOG00000018584	0.449950108	7.841435998	19.93461989	8.01E-06	5.15E-05
ENSRNOG00000024728	0.881064194	2.419612671	19.91938949	8.08E-06	5.18E-05
ENSRNOG00000002969	0.670917158	4.885152377	19.91850681	8.08E-06	5.18E-05
ENSRNOG00000033556	0.387845894	5.927225861	19.87891831	8.25E-06	5.28E-05
ENSRNOG00000033564	1.392293794	-1.076788818	19.87137039	8.28E-06	5.30E-05
ENSRNOG00000019152	0.367708727	6.602291947	19.86764823	8.30E-06	5.31E-05
ENSRNOG00000061270	0.623766724	7.901753228	19.84464158	8.40E-06	5.37E-05
ENSRNOG00000059660	0.5810585	7.510455086	19.82317557	8.49E-06	5.43E-05
ENSRNOG00000038883	0.362873221	7.570779795	19.81478482	8.53E-06	5.45E-05
ENSRNOG00000058457	0.552546521	1.757376201	19.79750192	8.61E-06	5.49E-05
ENSRNOG00000006916	0.377312527	4.046804474	19.79338191	8.63E-06	5.50E-05
ENSRNOG00000016145	0.471966334	6.656368412	19.76496116	8.76E-06	5.58E-05
ENSRNOG00000015685	0.449295497	3.122226825	19.76143034	8.77E-06	5.59E-05
ENSRNOG00000016776	0.52687748	4.890893988	19.75946519	8.78E-06	5.59E-05
ENSRNOG00000032871	0.312876919	8.553243675	19.72574503	8.94E-06	5.68E-05
ENSRNOG00000004595	0.424280066	4.140517619	19.69888828	9.07E-06	5.76E-05
ENSRNOG00000006548	0.824546683	2.850143495	19.6457218	9.32E-06	5.91E-05
ENSRNOG00000017585	0.420510802	3.628201716	19.61496767	9.47E-06	5.99E-05
ENSRNOG00000036697	0.490486316	4.460484398	19.60405387	9.53E-06	6.03E-05
ENSRNOG00000015294	0.436331788	4.636616241	19.59992588	9.55E-06	6.04E-05
ENSRNOG00000024711	0.572804268	4.084136981	19.59505158	9.57E-06	6.05E-05
ENSRNOG00000019363	1.196952943	-0.663751157	19.58776054	9.61E-06	6.07E-05

ENSRNOG00000007946	0.449302013	6.114526322	19.55940518	9.75E-06	6.16E-05
ENSRNOG00000048897	0.757207085	0.816384084	19.55636052	9.77E-06	6.16E-05
ENSRNOG00000017548	0.543252371	2.145291292	19.54659062	9.82E-06	6.19E-05
ENSRNOG00000002983	0.860786838	4.034175786	19.51940157	9.96E-06	6.28E-05
ENSRNOG00000024207	0.631169778	2.373235804	19.51214496	1.00E-05	6.30E-05
ENSRNOG00000001086	0.703673455	3.418424899	19.50587516	1.00E-05	6.31E-05
ENSRNOG00000011451	0.418069019	5.608309892	19.50426458	1.00E-05	6.32E-05
ENSRNOG00000019087	0.412946496	4.017301913	19.47742353	1.02E-05	6.40E-05
ENSRNOG00000031387	0.381459208	4.046904096	19.46494559	1.02E-05	6.44E-05
ENSRNOG00000020713	0.615332228	4.048045674	19.45473563	1.03E-05	6.47E-05
ENSRNOG00000013548	1.172653885	2.030908484	19.430383	1.04E-05	6.54E-05
ENSRNOG00000036303	0.805720646	-0.055649801	19.42213183	1.05E-05	6.57E-05
ENSRNOG00000001277	0.5722535	3.259130598	19.41411538	1.05E-05	6.59E-05
ENSRNOG00000023296	0.733732246	0.908487284	19.41303611	1.05E-05	6.59E-05
ENSRNOG00000040214	1.008304436	0.768282752	19.40983759	1.05E-05	6.60E-05
ENSRNOG00000003772	0.45753162	3.204879041	19.39558225	1.06E-05	6.64E-05
ENSRNOG00000021137	0.475520038	3.284698518	19.37343982	1.07E-05	6.71E-05
ENSRNOG00000017857	0.601621072	5.223928001	19.36178441	1.08E-05	6.75E-05
ENSRNOG00000001417	0.534822629	4.488792615	19.35896927	1.08E-05	6.75E-05
ENSRNOG00000012295	0.483947136	4.446640692	19.32190475	1.10E-05	6.88E-05
ENSRNOG00000052574	0.43227601	2.874264933	19.31434814	1.11E-05	6.90E-05
ENSRNOG00000002669	0.269487152	7.002300069	19.29791921	1.12E-05	6.96E-05
ENSRNOG00000015614	0.596511595	6.161055022	19.2938654	1.12E-05	6.97E-05
ENSRNOG00000018452	0.446126008	6.373906264	19.29118122	1.12E-05	6.98E-05
ENSRNOG00000001232	0.53785266	2.557138708	19.28718882	1.12E-05	6.99E-05
ENSRNOG00000060899	1.103052806	0.122569479	19.25403912	1.14E-05	7.10E-05
ENSRNOG00000012561	0.634842737	5.267057376	19.25206604	1.15E-05	7.10E-05
ENSRNOG00000060123	0.501208527	7.578863938	19.24118849	1.15E-05	7.14E-05
ENSRNOG00000014903	0.347917798	5.766785992	19.22994629	1.16E-05	7.17E-05
ENSRNOG00000011268	0.575459684	7.355041496	19.21811652	1.17E-05	7.20E-05
ENSRNOG00000032134	0.38305059	6.924735563	19.21803128	1.17E-05	7.20E-05
ENSRNOG00000018229	0.663600586	4.965332862	19.21476215	1.17E-05	7.21E-05
ENSRNOG00000056435	0.604401672	1.755159191	19.19195396	1.18E-05	7.29E-05
ENSRNOG00000019721	0.399880995	5.822647798	19.17829974	1.19E-05	7.33E-05
ENSRNOG00000019992	0.336056514	6.750757492	19.17375705	1.19E-05	7.34E-05
ENSRNOG00000043137	1.456321832	1.230657552	19.12492577	1.22E-05	7.52E-05
ENSRNOG00000019891	0.44531831	6.430094968	19.11458919	1.23E-05	7.55E-05
ENSRNOG00000030920	1.944971895	1.473923109	19.1051123	1.24E-05	7.59E-05
ENSRNOG00000026157	0.677178534	1.206651776	19.09319664	1.24E-05	7.63E-05
ENSRNOG00000006420	0.957994694	0.788914483	19.08418535	1.25E-05	7.66E-05
ENSRNOG00000027436	0.476059623	2.387787333	19.07479227	1.26E-05	7.70E-05
ENSRNOG00000026212	0.54288472	5.541178771	19.07406034	1.26E-05	7.70E-05
ENSRNOG00000020546	0.52589072	3.522063167	19.05335476	1.27E-05	7.78E-05
ENSRNOG00000008553	0.494529107	3.545915529	19.0282913	1.29E-05	7.87E-05
ENSRNOG00000018775	0.480572278	3.389200975	19.02576871	1.29E-05	7.88E-05
ENSRNOG00000018987	0.44439852	3.680521282	19.0173034	1.30E-05	7.91E-05
ENSRNOG00000030442	1.039844261	0.528592569	19.00754235	1.30E-05	7.94E-05
ENSRNOG00000007219	1.147956784	4.656107816	19.00018498	1.31E-05	7.97E-05
ENSRNOG00000018997	0.835641819	1.342480303	18.9885594	1.32E-05	8.02E-05
ENSRNOG00000008680	0.624673414	1.630117373	18.96485913	1.33E-05	8.11E-05
ENSRNOG00000048736	0.570595381	5.588893584	18.94091718	1.35E-05	8.21E-05
ENSRNOG00000024617	0.555000454	1.415911969	18.92871625	1.36E-05	8.26E-05
ENSRNOG00000038406	0.591815209	4.112061408	18.90831999	1.37E-05	8.34E-05
ENSRNOG00000028713	0.493448436	3.046501491	18.88533165	1.39E-05	8.43E-05
ENSRNOG00000009687	0.448619601	2.867476467	18.8845784	1.39E-05	8.43E-05
ENSRNOG00000036695	0.354172992	4.999167765	18.8801345	1.39E-05	8.45E-05
ENSRNOG00000014050	0.301724708	4.591030933	18.86208842	1.41E-05	8.53E-05
ENSRNOG00000028685	0.783155924	3.082910436	18.84781688	1.42E-05	8.59E-05
ENSRNOG00000019639	0.379250953	3.466918933	18.81953134	1.44E-05	8.71E-05
ENSRNOG00000022060	0.443810469	5.469564762	18.81161021	1.44E-05	8.74E-05
ENSRNOG00000009183	0.394677666	5.327990177	18.79848762	1.45E-05	8.80E-05
ENSRNOG00000053728	1.017003121	-0.67867146	18.79663186	1.45E-05	8.80E-05
ENSRNOG00000013474	0.823990814	2.073147139	18.75882412	1.48E-05	8.97E-05
ENSRNOG00000027936	0.527342193	2.160295367	18.74504956	1.49E-05	9.03E-05
ENSRNOG00000021732	0.418895475	4.58579222	18.7194213	1.51E-05	9.13E-05
ENSRNOG00000003278	0.525059486	2.970459714	18.71239316	1.52E-05	9.16E-05
ENSRNOG00000037476	0.898735111	4.026586733	18.6973062	1.53E-05	9.23E-05
ENSRNOG00000049731	0.881588989	0.205212603	18.66797082	1.56E-05	9.37E-05
ENSRNOG00000019390	1.16519576	-0.841527364	18.65937158	1.56E-05	9.40E-05
ENSRNOG00000037500	0.411689873	2.993284563	18.6326105	1.58E-05	9.52E-05
ENSRNOG00000003682	0.527932107	2.986544794	18.6273332	1.59E-05	9.55E-05
ENSRNOG00000024455	0.279597309	5.00069908	18.62570057	1.59E-05	9.55E-05
ENSRNOG00000006283	0.610772761	4.359309154	18.61563895	1.60E-05	9.59E-05
ENSRNOG00000006689	0.39312372	4.430381484	18.59033425	1.62E-05	9.72E-05
ENSRNOG00000059633	0.728798608	3.411602782	18.58764372	1.62E-05	9.73E-05
ENSRNOG00000047281	1.218271191	-0.188439013	18.57956486	1.63E-05	9.76E-05
ENSRNOG00000010068	0.388966119	3.322599774	18.57586767	1.63E-05	9.78E-05
ENSRNOG00000015822	0.741763205	1.8293776	18.5707059	1.64E-05	9.80E-05
ENSRNOG00000014597	0.577728694	2.996020897	18.56662305	1.64E-05	9.82E-05
ENSRNOG00000007756	0.508153873	3.896565071	18.55405874	1.65E-05	9.87E-05
ENSRNOG00000001738	0.274853964	6.650066161	18.55343739	1.65E-05	9.87E-05
ENSRNOG00000008786	0.312528347	6.802840009	18.5535885	1.65E-05	9.87E-05
ENSRNOG00000029134	0.458595235	7.897551607	18.54605438	1.66E-05	9.91E-05
ENSRNOG00000047867	0.37600477	4.487713717	18.53448172	1.67E-05	9.96E-05
ENSRNOG00000007459	0.283986301	6.802158777	18.53005084	1.67E-05	9.98E-05
ENSRNOG00000039587	0.625662748	5.695876841	18.52905962	1.67E-05	9.98E-05
ENSRNOG00000012852	0.492824158	7.267777421	18.51706708	1.68E-05	0.000100349
ENSRNOG00000020689	0.389157626	4.561731538	18.50645208	1.69E-05	0.000100796
ENSRNOG00000020875	0.509403848	5.642439281	18.50517831	1.69E-05	0.000100825
ENSRNOG00000021101	0.561247095	3.612911643	18.47316444	1.72E-05	0.000102379
ENSRNOG00000031495	0.555618167	2.823099504	18.47158212	1.72E-05	0.000102426
ENSRNOG00000011690	0.547038329	3.637047301	18.45507033	1.74E-05	0.00010324
ENSRNOG00000019920	0.514093248	4.241927728	18.43922969	1.75E-05	0.000103985
ENSRNOG00000059604	1.299499756	-0.947063075	18.439339	1.75E-05	0.000103985
ENSRNOG00000013743	0.246376638	5.671040156	18.39643793	1.79E-05	0.000106157
ENSRNOG00000037299	0.893980686	-0.184728474	18.3965307	1.79E-05	0.000106157
ENSRNOG00000009149	0.317245574	5.092197873	18.39268888	1.80E-05	0.000106317
ENSRNOG00000011366	0.317718293	5.025141894	18.36131965	1.83E-05	0.00010792
ENSRNOG00000000467	0.466053236	4.088240379	18.31149073	1.88E-05	0.000110657
ENSRNOG00000028090	0.338047676	4.696154018	18.30880778	1.88E-05	0.000110771

ENSRNOG00000001391	0.77498067	2.268663166	18.28634289	1.90E-05	0.000112002
ENSRNOG00000003033	0.736445578	2.63248172	18.28487956	1.90E-05	0.000112046
ENSRNOG00000007422	0.547166797	4.560053575	18.28402813	1.90E-05	0.000112055
ENSRNOG00000020178	0.496155754	5.190767281	18.28162425	1.91E-05	0.000112154
ENSRNOG00000054031	0.544600561	3.512773094	18.27480632	1.91E-05	0.000112515
ENSRNOG00000014226	0.72821387	3.991688924	18.26994185	1.92E-05	0.000112677
ENSRNOG00000058841	1.005403623	-0.197864961	18.26615927	1.92E-05	0.000112819
ENSRNOG00000017193	1.015381328	5.015539632	18.26541624	1.92E-05	0.000112819
ENSRNOG00000020714	0.738923453	1.314745389	18.24510413	1.94E-05	0.00011386
ENSRNOG00000008113	0.838648146	3.239616963	18.24307152	1.94E-05	0.000113913
ENSRNOG00000057447	0.388213533	4.269210264	18.21029048	1.98E-05	0.000115618
ENSRNOG00000004538	0.288577708	5.592142175	18.18772354	2.00E-05	0.00011678
ENSRNOG00000048291	0.593024918	2.849378968	18.18151418	2.01E-05	0.000117032
ENSRNOG00000013679	0.49748996	5.569620367	18.17619513	2.01E-05	0.000117187
ENSRNOG00000055010	0.443076352	3.70537088	18.17365479	2.02E-05	0.0001173
ENSRNOG00000028753	0.551149107	3.360099305	18.14238042	2.05E-05	0.000119024
ENSRNOG00000060288	1.285378989	0.162797383	18.13793401	2.05E-05	0.000119118
ENSRNOG00000008427	0.379638361	4.649468153	18.13884794	2.05E-05	0.000119118
ENSRNOG00000053206	1.17476501	-0.884972178	18.1380726	2.05E-05	0.000119118
ENSRNOG00000026982	0.880516282	1.474764671	18.13061068	2.06E-05	0.000119499
ENSRNOG00000051272	0.437350895	3.491667663	18.12003414	2.07E-05	0.00012012
ENSRNOG00000061519	0.413204807	4.588636666	18.1137182	2.08E-05	0.000120431
ENSRNOG00000049802	0.919254239	5.285201388	18.10198889	2.09E-05	0.000121043
ENSRNOG00000028543	0.69943908	0.446509651	18.09207138	2.10E-05	0.000121586
ENSRNOG00000023643	0.972453927	3.609858237	18.08768074	2.11E-05	0.000121822
ENSRNOG00000001217	1.513828879	-0.461833032	18.08286957	2.11E-05	0.000122086
ENSRNOG00000028501	0.327346896	4.801327379	18.0677181	2.13E-05	0.000122927
ENSRNOG00000012762	0.75182754	2.670265563	18.05008783	2.15E-05	0.000124026
ENSRNOG00000057774	0.293236806	5.592170463	18.041752	2.16E-05	0.000124524
ENSRNOG00000018975	0.378377349	6.624284123	18.03027885	2.17E-05	0.000125141
ENSRNOG00000015999	0.326988216	5.123419054	18.0135016	2.19E-05	0.000126157
ENSRNOG00000052354	0.642897917	5.50036826	17.9848561	2.23E-05	0.000128023
ENSRNOG00000048986	0.748535808	2.279421095	17.95348635	2.26E-05	0.000130056
ENSRNOG00000011315	0.541397902	4.551500921	17.92961894	2.29E-05	0.000131601
ENSRNOG00000032394	0.407466622	4.683282226	17.92526652	2.30E-05	0.000131855
ENSRNOG00000010204	0.368769992	5.134752277	17.91572853	2.31E-05	0.000132421
ENSRNOG00000060996	1.171463177	-0.897349849	17.89071372	2.34E-05	0.000133931
ENSRNOG00000007405	0.444484543	4.617961266	17.88101462	2.35E-05	0.000134469
ENSRNOG00000008824	1.280388795	0.186449013	17.87865275	2.35E-05	0.000134588
ENSRNOG00000047683	1.246791587	-1.430597188	17.86527869	2.37E-05	0.000135459
ENSRNOG00000017558	0.434824067	6.295877129	17.86500111	2.37E-05	0.000135459
ENSRNOG00000026985	0.651396708	6.014991915	17.85461908	2.38E-05	0.000136003
ENSRNOG00000019822	0.877780091	1.801738406	17.84087919	2.40E-05	0.000136763
ENSRNOG00000041535	1.042400096	-0.888050911	17.84060121	2.40E-05	0.000136763
ENSRNOG00000017359	0.413856551	9.295854669	17.82698966	2.42E-05	0.000137695
ENSRNOG00000060571	0.494308711	4.997534732	17.79203692	2.46E-05	0.000140147
ENSRNOG00000007455	0.539797086	4.091805544	17.78298586	2.48E-05	0.000140765
ENSRNOG00000021261	0.271400737	4.99247108	17.78193156	2.48E-05	0.000140792
ENSRNOG00000011262	0.526733427	4.110445332	17.77464359	2.49E-05	0.000141255
ENSRNOG00000000422	0.645582922	2.189600913	17.76811242	2.50E-05	0.000141565
ENSRNOG00000013752	0.363254639	6.024921783	17.74823946	2.52E-05	0.000143
ENSRNOG00000015417	0.329369566	6.798550408	17.70761902	2.58E-05	0.000145877
ENSRNOG00000028640	0.631554906	1.602771758	17.70332187	2.58E-05	0.000146155
ENSRNOG00000054458	0.99127907	3.602187359	17.69638838	2.59E-05	0.000146584
ENSRNOG00000053803	1.284205797	-0.961883618	17.68949564	2.60E-05	0.000147011
ENSRNOG00000046505	0.773494751	0.37523556	17.64617979	2.66E-05	0.000150236
ENSRNOG00000042044	0.397573626	4.811301857	17.64440625	2.66E-05	0.000150323
ENSRNOG00000021285	1.040955165	3.725048525	17.62388572	2.69E-05	0.000151845
ENSRNOG00000022934	1.314388195	-1.107623963	17.60660045	2.72E-05	0.000153013
ENSRNOG00000004208	0.537247354	5.277728806	17.58881714	2.74E-05	0.000154286
ENSRNOG00000009906	0.612228442	1.729003881	17.56004875	2.78E-05	0.000156527
ENSRNOG00000017209	0.559780597	7.799654139	17.55175278	2.80E-05	0.000157044
ENSRNOG00000050511	0.928380708	-0.114650103	17.52276161	2.84E-05	0.000159175
ENSRNOG00000019012	0.395399995	3.764254257	17.49357079	2.88E-05	0.000161466
ENSRNOG00000033581	0.788210159	2.57843691	17.47385704	2.91E-05	0.000162976
ENSRNOG00000007989	0.34947603	6.810024292	17.46492391	2.93E-05	0.000163686
ENSRNOG00000006019	0.961892321	1.454593175	17.45893002	2.94E-05	0.000164145
ENSRNOG00000002232	0.558994346	2.232294462	17.45462316	2.94E-05	0.000164459
ENSRNOG00000004311	0.607956918	1.00262673	17.43854791	2.97E-05	0.000165797
ENSRNOG00000043378	1.057079879	0.55280072	17.43132622	2.98E-05	0.000166335
ENSRNOG00000010580	0.556439052	6.786837886	17.40644882	3.02E-05	0.000168324
ENSRNOG00000052296	0.901616417	6.990173624	17.39801308	3.03E-05	0.000169013
ENSRNOG00000048718	0.354598004	4.382873696	17.38390871	3.05E-05	0.000170212
ENSRNOG00000003837	0.417393199	2.956246719	17.37284027	3.07E-05	0.000171025
ENSRNOG00000050057	0.348280618	5.984757097	17.36485811	3.08E-05	0.000171624
ENSRNOG00000020635	0.604951706	2.817422094	17.34848741	3.11E-05	0.000172927
ENSRNOG00000005573	0.425797755	3.485965057	17.34766436	3.11E-05	0.000172941
ENSRNOG00000004333	0.644576849	7.231140988	17.33529084	3.13E-05	0.000173949
ENSRNOG00000010083	0.364358132	4.711629881	17.3334063	3.14E-05	0.000173999
ENSRNOG00000017164	0.368186017	3.567753851	17.31508184	3.17E-05	0.000175562
ENSRNOG00000019435	0.807906897	6.918021459	17.29800604	3.20E-05	0.000177085
ENSRNOG00000039596	1.073254611	-0.719759289	17.29257562	3.20E-05	0.000177529
ENSRNOG00000013663	0.402383544	2.496218852	17.28590003	3.22E-05	0.00017803
ENSRNOG00000055446	0.326632183	6.997088193	17.27737063	3.23E-05	0.000178706
ENSRNOG00000020185	0.663574164	7.735236779	17.24929804	3.28E-05	0.000181176
ENSRNOG00000036301	0.874052394	3.727108729	17.20870046	3.35E-05	0.000184766
ENSRNOG00000045846	0.875716679	1.94521436	17.20225619	3.36E-05	0.000185234
ENSRNOG00000045556	0.928528953	0.980737108	17.18670156	3.39E-05	0.000186528
ENSRNOG00000025711	0.376773722	4.629643129	17.17143098	3.42E-05	0.000187903
ENSRNOG00000055247	1.299488345	0.146173605	17.16030672	3.44E-05	0.00018881
ENSRNOG00000023861	0.369041004	7.73633757	17.16094991	3.43E-05	0.00018881
ENSRNOG00000024931	0.650896519	1.264938807	17.13776568	3.48E-05	0.000190931
ENSRNOG00000028659	0.326977421	5.619588433	17.13170815	3.49E-05	0.000191475
ENSRNOG00000012952	0.456139899	4.901580708	17.11202934	3.52E-05	0.000193268
ENSRNOG00000052386	0.481360963	3.189709401	17.11221939	3.52E-05	0.000193268
ENSRNOG00000039025	0.548905819	2.213008896	17.09701462	3.55E-05	0.000194613
ENSRNOG00000048898	0.369977835	6.162090874	17.09688323	3.55E-05	0.000194613
ENSRNOG00000016587	0.501920906	3.355665411	17.09812729	3.55E-05	0.000194613
ENSRNOG00000015409	0.275385766	7.06282239	17.08714003	3.57E-05	0.000195547
ENSRNOG00000007131	0.252612003	5.08293971	17.07561365	3.59E-05	0.000196601

ENSRNOG00000018801	0.333981148	4.674689656	17.07567045	3.59E-05	0.000196601
ENSRNOG00000006226	0.433714442	4.963831236	17.03882261	3.66E-05	0.000200309
ENSRNOG00000008951	0.457464776	4.451251562	17.01766787	3.70E-05	0.000202413
ENSRNOG00000049028	0.822838086	-0.25111123	17.01558596	3.71E-05	0.000202565
ENSRNOG00000003026	0.377625618	5.024714573	17.01031003	3.72E-05	0.000202919
ENSRNOG00000002208	1.169631534	0.983701946	17.00101096	3.74E-05	0.000203845
ENSRNOG00000046000	0.573316702	5.250579223	16.96235187	3.81E-05	0.000207823
ENSRNOG00000001880	0.45916085	5.004647063	16.94045757	3.86E-05	0.000210089
ENSRNOG00000009605	0.417514343	3.582453066	16.93132311	3.88E-05	0.00021103
ENSRNOG00000002475	0.97941502	0.301917464	16.92736863	3.88E-05	0.000211324
ENSRNOG00000062039	1.321579735	-0.777573951	16.92446412	3.89E-05	0.000211575
ENSRNOG00000011527	0.943993697	-0.309625184	16.92100287	3.90E-05	0.000211763
ENSRNOG00000003442	0.34875683	6.491680284	16.92193514	3.89E-05	0.000211763
ENSRNOG00000025634	0.550599429	5.023273427	16.92082322	3.90E-05	0.000211763
ENSRNOG00000037229	0.401512765	3.613880424	16.87351156	4.00E-05	0.000216866
ENSRNOG00000062036	1.211194338	0.024565814	16.87301883	4.00E-05	0.000216866
ENSRNOG00000020835	0.58701146	3.501192764	16.86625633	4.01E-05	0.000217417
ENSRNOG00000020710	0.403454137	4.930007867	16.864854	4.01E-05	0.000217488
ENSRNOG00000046021	1.366205155	1.095612408	16.86433551	4.01E-05	0.000217488
ENSRNOG00000000528	0.762501352	1.459577113	16.80553925	4.14E-05	0.000223719
ENSRNOG00000008749	1.123620906	1.903679096	16.77270553	4.21E-05	0.000227391
ENSRNOG00000000536	0.59780683	3.340692745	16.76334176	4.23E-05	0.000228283
ENSRNOG00000000442	0.476534003	3.617294161	16.74957537	4.27E-05	0.000229789
ENSRNOG00000014522	0.588138905	4.051349112	16.73634613	4.30E-05	0.000231239
ENSRNOG00000057378	0.291628818	4.952558739	16.72920162	4.31E-05	0.000231954
ENSRNOG00000018686	0.37178151	6.579408249	16.71847297	4.34E-05	0.000233111
ENSRNOG00000006908	0.482201042	2.993613249	16.71428659	4.35E-05	0.000233546
ENSRNOG00000045770	0.628497093	1.657834111	16.70311612	4.37E-05	0.000234846
ENSRNOG00000026040	0.521581201	2.71211592	16.69134183	4.40E-05	0.000236228
ENSRNOG00000022421	0.808266029	4.950396514	16.68471359	4.41E-05	0.000236974
ENSRNOG00000050240	0.89296178	0.261590977	16.66311005	4.46E-05	0.000239445
ENSRNOG00000037566	0.388357515	3.222175257	16.64939634	4.50E-05	0.000240955
ENSRNOG00000012347	0.527971239	1.59663658	16.62426113	4.56E-05	0.000243904
ENSRNOG00000059015	0.533487196	2.956843502	16.62431326	4.56E-05	0.000243904
ENSRNOG00000018226	0.616890208	4.640431634	16.61572864	4.58E-05	0.000244838
ENSRNOG00000026649	0.37140301	4.672329848	16.59702326	4.62E-05	0.000246951
ENSRNOG00000018233	0.468150568	6.287782667	16.5968711	4.62E-05	0.000246951
ENSRNOG00000021986	0.564659726	2.173738408	16.58954969	4.64E-05	0.000247822
ENSRNOG00000007057	0.433674148	5.16237706	16.5821601	4.66E-05	0.000248706
ENSRNOG00000025185	0.366041642	4.413815842	16.54277967	4.76E-05	0.000253583
ENSRNOG00000019438	0.399343991	4.041383229	16.52349346	4.81E-05	0.000256089
ENSRNOG00000024954	0.534527523	4.757224239	16.5205144	4.81E-05	0.000256405
ENSRNOG00000019451	0.831760759	1.763918922	16.5090159	4.84E-05	0.000257791
ENSRNOG00000027240	0.762254239	5.899043657	16.47905364	4.92E-05	0.000261633
ENSRNOG00000016062	0.414477179	4.399702717	16.46892796	4.95E-05	0.000262946
ENSRNOG00000008168	0.813604222	-0.055128457	16.436099	5.03E-05	0.000267001
ENSRNOG00000024364	0.577478623	1.409183632	16.41238196	5.10E-05	0.000270091
ENSRNOG00000015304	0.597731895	3.076552658	16.40644917	5.11E-05	0.000270847
ENSRNOG00000051771	1.206309739	-0.572348499	16.39491639	5.14E-05	0.000272409
ENSRNOG00000037483	0.223341587	6.723839	16.38009802	5.18E-05	0.000274294
ENSRNOG00000013067	0.593609733	1.71733506	16.36296043	5.23E-05	0.000276671
ENSRNOG00000057125	0.515813988	6.065631501	16.34404761	5.28E-05	0.000279259
ENSRNOG00000020107	0.298317327	4.694895585	16.33870751	5.30E-05	0.000279953
ENSRNOG00000008990	0.872770622	3.74836078	16.3379192	5.30E-05	0.000279976
ENSRNOG00000009917	0.749675325	2.09917643	16.33058828	5.32E-05	0.000280968
ENSRNOG00000004206	0.622507847	4.262200653	16.32843317	5.33E-05	0.000281194
ENSRNOG00000054549	0.459000918	4.22693184	16.32421866	5.34E-05	0.000281632
ENSRNOG00000046299	1.002856947	1.60746042	16.31437754	5.37E-05	0.000282533
ENSRNOG00000018689	0.581488097	2.276875263	16.2993004	5.41E-05	0.000284232
ENSRNOG00000011119	0.446040519	4.25351247	16.29923912	5.41E-05	0.000284232
ENSRNOG00000003108	0.394827469	4.426892146	16.27841218	5.47E-05	0.000287278
ENSRNOG00000010489	0.765917374	3.482168776	16.25413913	5.54E-05	0.000290597
ENSRNOG00000028641	0.51722609	4.660896219	16.24097138	5.58E-05	0.000292526
ENSRNOG00000061328	0.528291505	1.386899067	16.23760593	5.59E-05	0.000292922
ENSRNOG00000027722	1.325029904	1.154624808	16.23715797	5.59E-05	0.000292922
ENSRNOG00000005378	0.574597761	1.463208241	16.22901291	5.61E-05	0.000293892
ENSRNOG00000011460	0.347846707	6.007601716	16.22497596	5.62E-05	0.000294422
ENSRNOG00000002061	0.321148062	3.827900624	16.21775802	5.65E-05	0.000295448
ENSRNOG00000011162	0.647468333	0.447815277	16.19475617	5.72E-05	0.000298859
ENSRNOG00000030355	0.584275668	5.456912356	16.18324879	5.75E-05	0.000300581
ENSRNOG00000013036	1.042946783	2.986547969	16.17304329	5.78E-05	0.000302105
ENSRNOG00000007133	0.344456276	6.374571249	16.15941448	5.82E-05	0.000304086
ENSRNOG00000015051	0.547266012	5.347637321	16.12652751	5.92E-05	0.000308903
ENSRNOG00000028341	0.276293996	5.341714698	16.11292644	5.97E-05	0.000311027
ENSRNOG00000056786	0.579181442	2.681134531	16.10502099	5.99E-05	0.000312225
ENSRNOG00000045895	0.934739401	-0.408761263	16.10044924	6.01E-05	0.000312877
ENSRNOG00000017824	0.27388175	5.292358029	16.09107588	6.04E-05	0.000314326
ENSRNOG00000002949	0.333963212	5.288067579	16.08954773	6.04E-05	0.000314476
ENSRNOG00000018916	0.550489388	2.283965779	16.0874404	6.05E-05	0.000314723
ENSRNOG00000005996	1.127093954	2.111588511	16.08577088	6.05E-05	0.000314793
ENSRNOG00000019024	1.131476789	-0.430353523	16.08610685	6.05E-05	0.000314793
ENSRNOG00000028946	0.376518409	5.798593109	16.04920522	6.17E-05	0.00032051
ENSRNOG00000040346	0.821130117	1.388955394	16.04205847	6.20E-05	0.000321511
ENSRNOG00000032569	1.064113561	2.256750867	16.03087566	6.23E-05	0.000323204
ENSRNOG00000014452	0.960875147	4.098339971	16.00870731	6.31E-05	0.000326689
ENSRNOG00000017249	0.501986712	2.181364054	16.00071525	6.33E-05	0.000327964
ENSRNOG00000038044	0.349192496	3.991989906	15.99382987	6.35E-05	0.000329051
ENSRNOG00000008376	0.701784266	1.094239235	15.97659733	6.41E-05	0.000331735
ENSRNOG00000030404	0.258667936	6.003688233	15.95623082	6.48E-05	0.000334995
ENSRNOG00000028120	0.591803436	0.897864738	15.95334309	6.49E-05	0.000335178
ENSRNOG00000003028	0.507861361	2.785499195	15.95039493	6.50E-05	0.000335591
ENSRNOG00000028021	0.44388482	5.487125936	15.94764145	6.51E-05	0.00033597
ENSRNOG00000004709	0.412692437	5.077577359	15.93610457	6.55E-05	0.000337804
ENSRNOG00000011502	0.557459511	3.216512752	15.92152462	6.60E-05	0.000340194
ENSRNOG00000047023	0.406321156	4.338571474	15.90681376	6.65E-05	0.000342514
ENSRNOG00000016150	0.46932643	2.259453609	15.88985935	6.71E-05	0.000345484
ENSRNOG00000024824	1.001170912	-0.408958228	15.88734931	6.72E-05	0.00034583
ENSRNOG00000027376	0.622421005	0.881275854	15.88319859	6.74E-05	0.000346477
ENSRNOG00000001181	0.550400269	2.196888446	15.88181823	6.74E-05	0.000346617
ENSRNOG00000020726	0.614184841	3.243202675	15.87113546	6.78E-05	0.000348353

ENSRNOG00000017912	0.500950261	1.750254751	15.86847611	6.79E-05	0.000348617
ENSRNOG00000054039	0.802173278	0.249406417	15.83791209	6.90E-05	0.000353834
ENSRNOG00000029988	1.268498605	-0.058561711	15.82011737	6.97E-05	0.000357062
ENSRNOG00000008005	0.611176301	3.409445644	15.81882314	6.97E-05	0.00035719
ENSRNOG00000006711	0.41780926	2.862090016	15.78359585	7.10E-05	0.00036355
ENSRNOG00000036688	0.426935862	7.938682853	15.75399493	7.21E-05	0.000368925
ENSRNOG00000031778	0.586008744	5.525695162	15.72477092	7.33E-05	0.000374306
ENSRNOG00000016254	0.41145716	3.867325565	15.72533861	7.32E-05	0.000374306
ENSRNOG00000058271	0.457585013	5.435375954	15.72156144	7.34E-05	0.000374821
ENSRNOG00000006984	0.509411551	4.637105529	15.71640478	7.36E-05	0.000375723
ENSRNOG00000025890	0.2891399	4.753629101	15.70644651	7.40E-05	0.000377584
ENSRNOG00000059984	0.441202396	2.219715958	15.69341868	7.45E-05	0.000379827
ENSRNOG00000049604	0.565394356	3.602741034	15.66912891	7.54E-05	0.000384241
ENSRNOG00000015068	0.55134141	3.926994992	15.64162454	7.66E-05	0.000389494
ENSRNOG00000019873	0.51230742	4.022481815	15.62820427	7.71E-05	0.000392016
ENSRNOG00000005331	0.381404086	5.398686902	15.61531987	7.76E-05	0.000394443
ENSRNOG00000057813	0.238818349	5.136517424	15.60810297	7.79E-05	0.000395697
ENSRNOG00000048043	0.399390624	4.948913165	15.58791478	7.88E-05	0.00039956
ENSRNOG00000010227	0.285448051	4.104192891	15.57918613	7.91E-05	0.000401152
ENSRNOG00000012739	0.311958482	3.251446411	15.57159806	7.94E-05	0.000402636
ENSRNOG00000007407	0.718085465	2.853154072	15.56772153	7.96E-05	0.000403205
ENSRNOG00000012406	0.570595369	6.256822287	15.53877335	8.08E-05	0.000408903
ENSRNOG00000055042	1.443703521	0.661267927	15.51938887	8.17E-05	0.000412853
ENSRNOG00000047219	0.481023281	5.140307599	15.4955307	8.27E-05	0.000417697
ENSRNOG00000061408	0.629153857	1.223860428	15.49253074	8.28E-05	0.000418227
ENSRNOG00000053247	0.345800478	4.699587895	15.46055945	8.42E-05	0.000424955
ENSRNOG00000025296	0.513195487	6.366552182	15.43807072	8.53E-05	0.000429572
ENSRNOG00000028255	0.266940602	4.906469001	15.43773149	8.53E-05	0.000429572
ENSRNOG00000000431	0.400163717	5.370257361	15.43857643	8.52E-05	0.000429572
ENSRNOG00000046001	1.440109575	2.638376589	15.42204463	8.60E-05	0.000432911
ENSRNOG00000037894	1.322738659	-0.482149903	15.41789642	8.62E-05	0.00043369
ENSRNOG00000018533	0.417891438	5.179667155	15.40408521	8.68E-05	0.000436594
ENSRNOG00000039856	0.395709204	4.127631253	15.39369036	8.73E-05	0.000438584
ENSRNOG00000013733	0.274412379	4.434781469	15.37776605	8.80E-05	0.000441876
ENSRNOG00000058920	0.993371956	-1.048177892	15.37707391	8.81E-05	0.000441897
ENSRNOG00000048738	0.697888517	1.373621833	15.3734152	8.82E-05	0.000442614
ENSRNOG00000012832	0.385651241	3.924947996	15.34580176	8.95E-05	0.000448561
ENSRNOG00000036678	0.487357118	4.402216587	15.33456407	9.01E-05	0.000451095
ENSRNOG00000009892	0.649561378	1.389612747	15.33312064	9.01E-05	0.000451297
ENSRNOG00000020970	0.388882483	7.217984471	15.31075078	9.12E-05	0.000456095
ENSRNOG00000021004	0.57730128	2.841454614	15.29098641	9.22E-05	0.000460456
ENSRNOG00000001135	0.327364985	7.172903039	15.28514339	9.24E-05	0.000461737
ENSRNOG00000007281	0.698792714	2.986587437	15.27567446	9.29E-05	0.000463619
ENSRNOG00000054838	0.493919843	5.207225129	15.24181871	9.46E-05	0.000471261
ENSRNOG00000028569	0.623915453	2.579526907	15.16440869	9.85E-05	0.000490209
ENSRNOG00000017940	0.337145491	6.931931339	15.15464553	9.91E-05	0.000492596
ENSRNOG00000008133	0.775582622	2.552026472	15.14971101	9.93E-05	0.00049373
ENSRNOG00000015750	0.417788421	4.529045867	15.1430724	9.97E-05	0.000495314
ENSRNOG00000053773	0.846722095	0.196289541	15.12492978	0.000100626	0.000499626
ENSRNOG00000016071	0.537036479	5.658304327	15.10412522	0.000101741	0.000504673
ENSRNOG00000029242	0.486057191	3.402345682	15.08627465	0.000102707	0.000509004
ENSRNOG00000022919	1.155865906	1.56892894	15.0798641	0.000103057	0.000510257
ENSRNOG00000007769	0.365662795	5.665824505	15.05360592	0.0001045	0.00051692
ENSRNOG00000003304	0.551049098	3.243815344	15.04832355	0.000104793	0.000518206
ENSRNOG00000008146	0.78794234	4.016304819	15.0422421	0.000105131	0.000519717
ENSRNOG00000029490	0.409668297	3.064935908	15.03922768	0.0001053	0.000520385
ENSRNOG00000048113	0.58855359	1.816425726	15.03688504	0.00010543	0.000520715
ENSRNOG00000007184	1.252269503	-0.294897372	15.01991701	0.000106382	0.000525081
ENSRNOG00000005580	1.007165195	2.673283687	15.0108415	0.000106895	0.000527284
ENSRNOG00000014293	0.50967588	3.245761505	15.00765204	0.000107076	0.000528011
ENSRNOG00000028168	0.487176394	1.909418359	14.99645679	0.000107713	0.000530492
ENSRNOG00000003691	0.518472302	6.7471666	14.99701809	0.000107681	0.000530492
ENSRNOG00000011156	0.514132773	8.117947672	14.99483201	0.000107806	0.000530784
ENSRNOG00000002303	0.402860641	2.854657387	14.98332738	0.000108465	0.000533532
ENSRNOG00000001436	0.376974551	9.497444488	14.97893846	0.000108718	0.00053422
ENSRNOG00000019575	0.597460679	5.247432267	14.97874487	0.000108729	0.00053422
ENSRNOG00000059572	0.767385984	0.502150807	14.97098182	0.000109177	0.000536201
ENSRNOG00000026857	0.530964124	2.872703571	14.9245105	0.0001119	0.000548721
ENSRNOG00000001265	0.442592259	3.082164277	14.91230962	0.000112626	0.000551939
ENSRNOG00000000437	0.405102597	6.567027132	14.90912253	0.000112816	0.000552701
ENSRNOG00000049380	0.954020838	-0.098252892	14.8933028	0.000113766	0.000557011
ENSRNOG00000046845	0.560798814	1.364185973	14.87159298	0.000115083	0.000562936
ENSRNOG00000008064	0.343410478	4.928808611	14.86903561	0.000115239	0.000563526
ENSRNOG00000008332	0.484243068	3.762322589	14.86149228	0.000115701	0.000565435
ENSRNOG00000021904	0.328809375	5.236420006	14.84445521	0.000116751	0.000570213
ENSRNOG00000024677	0.830027818	6.228566876	14.84188317	0.00011691	0.00057064
ENSRNOG00000058202	0.491123142	6.575943796	14.84220631	0.00011689	0.00057064
ENSRNOG00000028584	0.629998805	1.120197703	14.82270027	0.000118105	0.000575763
ENSRNOG00000011467	0.333441795	4.953663902	14.8211438	0.000118203	0.000576061
ENSRNOG00000008831	0.4592604	5.89786667	14.80858429	0.000118993	0.000579197
ENSRNOG00000033787	0.991358237	-0.785437389	14.79323018	0.000119965	0.000583394
ENSRNOG00000055012	1.557148217	0.769059577	14.77961111	0.000120835	0.000587262
ENSRNOG00000018666	0.383042972	6.151364847	14.77637796	0.000121042	0.00058809
ENSRNOG00000025034	0.400945232	3.745046368	14.77355821	0.000121223	0.000588789
ENSRNOG00000049196	0.710115605	0.717072236	14.76844804	0.000121552	0.000590196
ENSRNOG00000017983	0.296779404	5.151156806	14.76192558	0.000121974	0.000591707
ENSRNOG00000062279	0.768064443	-0.256995073	14.75346722	0.000122522	0.000594182
ENSRNOG00000001178	0.237880798	5.792738857	14.73483344	0.000123739	0.000599719
ENSRNOG00000030714	0.523857739	7.383926233	14.73291216	0.000123865	0.000600147
ENSRNOG00000047367	0.580008119	0.600405152	14.72697888	0.000124255	0.00060167
ENSRNOG00000001385	0.585663592	5.390134103	14.71523252	0.000125032	0.000605061
ENSRNOG00000004027	0.291597137	4.592166482	14.70267065	0.000125868	0.000608734
ENSRNOG00000046245	0.454610004	2.772760993	14.68832626	0.000126829	0.000612448
ENSRNOG00000054757	0.456636391	5.322793178	14.68869012	0.000126805	0.000612448
ENSRNOG00000059453	0.419004786	3.246861596	14.68928547	0.000126765	0.000612448
ENSRNOG00000007391	1.093724348	-1.005772233	14.67913045	0.00012745	0.000615255
ENSRNOG00000017746	0.35145134	3.10322686	14.65647343	0.000128991	0.000621748
ENSRNOG00000001402	0.482445689	3.387675505	14.65457644	0.000129121	0.000622184
ENSRNOG00000016708	0.866078077	3.683553122	14.64161362	0.000130012	0.000626097
ENSRNOG00000037613	0.438571553	4.393436111	14.64033823	0.0001301	0.000626331

ENSRNOG00000032152	0.351463777	6.020915823	14.62495904	0.000131166	0.000631079
ENSRNOG00000008634	0.363614742	4.861934083	14.61745527	0.000131689	0.000633019
ENSRNOG00000017365	0.729395326	0.200140776	14.59550533	0.000133232	0.000640047
ENSRNOG00000048187	1.982907617	-0.425719911	14.58895034	0.000133696	0.000641522
ENSRNOG00000059215	0.449985521	3.895799255	14.58406305	0.000134043	0.00064297
ENSRNOG00000020716	0.256390515	5.404502354	14.58247604	0.000134156	0.000643317
ENSRNOG00000003239	0.28294444	4.798533604	14.57315431	0.000134822	0.00064592
ENSRNOG00000061515	0.343185852	3.323706686	14.496475	0.000140422	0.000671735
ENSRNOG00000013945	0.360836756	3.365893076	14.47885886	0.000141741	0.000677024
ENSRNOG00000062252	1.526262466	0.650662938	14.47138363	0.000142305	0.000679306
ENSRNOG00000011488	0.412919724	3.834667442	14.46608591	0.000142706	0.000680809
ENSRNOG00000057703	0.49173901	4.572460813	14.44592625	0.000144242	0.000687928
ENSRNOG00000001249	0.629469177	2.305216039	14.43671056	0.000144949	0.000690337
ENSRNOG00000043417	1.01914287	-0.83652841	14.42870887	0.000145566	0.000692785
ENSRNOG00000011927	0.529970171	7.608942117	14.38966009	0.000148616	0.000706451
ENSRNOG00000007332	0.667859176	2.756660189	14.3755166	0.000149737	0.000710923
ENSRNOG00000009691	0.35942887	6.10557219	14.37557658	0.000149732	0.000710923
ENSRNOG00000009369	0.48101415	2.052753478	14.36900459	0.000150255	0.000713173
ENSRNOG00000017523	0.522941896	2.692138765	14.36640624	0.000150463	0.00071373
ENSRNOG00000014338	0.267195487	6.076552945	14.35234616	0.000151591	0.000718649
ENSRNOG00000004146	0.535443989	5.147288955	14.33078992	0.000153336	0.000725838
ENSRNOG00000010199	0.426954472	6.854267642	14.32515842	0.000153796	0.000727795
ENSRNOG00000028630	0.430182051	3.334955398	14.32229286	0.00015403	0.000728686
ENSRNOG00000050630	0.527750703	1.975091732	14.31738669	0.000154432	0.000730155
ENSRNOG00000017100	1.445300163	0.455415092	14.31737675	0.000154433	0.000730155
ENSRNOG00000000572	0.539403974	2.517845893	14.30852836	0.00015516	0.000733376
ENSRNOG00000047896	0.270757649	5.140373349	14.28356183	0.000157232	0.000742724
ENSRNOG00000007567	0.734701213	-0.071620249	14.27760109	0.000157731	0.000744858
ENSRNOG00000047046	0.977382275	1.328895383	14.27484046	0.000157962	0.000745729
ENSRNOG00000023309	0.596954248	3.259124747	14.24829558	0.000160206	0.00075587
ENSRNOG00000005031	0.269415521	3.500762152	14.24676089	0.000160337	0.000756261
ENSRNOG00000010188	2.633096514	1.964709159	14.17341709	0.000166709	0.000784217
ENSRNOG00000060528	0.805536114	5.651987278	14.16992294	0.000167019	0.000785441
ENSRNOG00000004560	0.320886123	6.586579418	14.14324564	0.000169404	0.000795948
ENSRNOG00000003741	0.808592361	6.182133117	14.14030786	0.000169669	0.000796956
ENSRNOG00000057877	1.043104527	-0.297093286	14.13498079	0.00017015	0.000798448
ENSRNOG00000061282	1.043104527	-0.297093286	14.13498079	0.00017015	0.000798448
ENSRNOG00000054557	0.348224571	5.145145967	14.1278569	0.000170796	0.000800823
ENSRNOG00000019831	0.483662406	5.257025744	14.11203743	0.000172238	0.00080663
ENSRNOG00000010588	0.486160474	5.123171527	14.1107367	0.000172357	0.000806711
ENSRNOG00000011592	0.335509086	4.224465095	14.11106553	0.000172327	0.000806711
ENSRNOG00000048190	0.408305785	4.438441667	14.10305696	0.000173062	0.000809533
ENSRNOG00000006997	0.298594877	10.03735335	14.10144779	0.00017321	0.000809986
ENSRNOG00000007242	0.29507519	4.576858692	14.09952291	0.000173388	0.000810576
ENSRNOG00000019804	0.479320799	6.240734941	14.07597526	0.000175572	0.000819095
ENSRNOG00000061429	0.485770457	1.690017957	14.05812363	0.000177247	0.00082642
ENSRNOG00000059663	1.076035506	-1.104635153	14.05719669	0.000177334	0.000826584
ENSRNOG00000019635	0.255596429	5.862622631	14.05067165	0.00017795	0.000828725
ENSRNOG00000027906	0.248203058	7.344017847	14.05077084	0.000177941	0.000828725
ENSRNOG00000002139	0.395353382	6.603400212	14.04070215	0.000178896	0.00083264
ENSRNOG00000001169	0.489736466	2.583622886	14.03605381	0.000179339	0.000834455
ENSRNOG00000016502	0.495365257	4.894150983	14.01394839	0.00018146	0.000843827
ENSRNOG00000048747	0.291663993	5.102917839	14.01243109	0.000181606	0.000844065
ENSRNOG00000002963	0.75436471	2.953502315	14.00567327	0.00018226	0.000846554
ENSRNOG00000022014	0.219665197	5.663373541	13.9980277	0.000183002	0.000849256
ENSRNOG00000045994	0.967722948	-0.117981258	13.99309762	0.000183483	0.000850738
ENSRNOG00000012653	0.306700679	5.607819896	13.97025816	0.000185725	0.000860631
ENSRNOG00000008619	0.292529912	4.489127497	13.96201907	0.000186541	0.000863906
ENSRNOG00000057848	0.489941317	4.202330046	13.95132536	0.000187605	0.000868072
ENSRNOG00000022771	0.541291702	5.714433937	13.92918063	0.000189828	0.000877846
ENSRNOG00000015257	0.426029584	5.776837886	13.91359724	0.000191409	0.000884637
ENSRNOG00000016847	0.253748552	6.028572144	13.90230491	0.000192562	0.000889189
ENSRNOG00000052224	0.853311659	4.456925811	13.88973519	0.000193854	0.000894634
ENSRNOG00000004773	0.323724106	5.542599279	13.88166128	0.000194689	0.000897701
ENSRNOG00000052707	0.306354174	6.448675257	13.87202135	0.00019569	0.000901792
ENSRNOG00000026048	0.23640548	6.617125465	13.87038426	0.000195861	0.000902315
ENSRNOG00000018097	0.246514784	6.335959776	13.86613118	0.000196304	0.000904097
ENSRNOG00000017883	0.229829763	6.594232856	13.86035615	0.000196909	0.000906615
ENSRNOG00000056727	1.010455427	-0.408936135	13.85978931	0.000196968	0.000906625
ENSRNOG00000009615	0.209493987	6.659907165	13.84873054	0.000198131	0.000910917
ENSRNOG00000024352	0.305610186	3.301002955	13.84517803	0.000198505	0.000912111
ENSRNOG00000006237	0.300329597	7.463673735	13.84337031	0.000198697	0.000912531
ENSRNOG00000003434	0.28555179	4.880285734	13.83195846	0.000199907	0.000917752
ENSRNOG00000004361	0.545932571	1.763068955	13.82264734	0.0002009	0.000921242
ENSRNOG00000007657	0.541288299	3.964057783	13.82130624	0.000201043	0.000921633
ENSRNOG00000016525	0.667375819	1.116371851	13.81494995	0.000201725	0.000924489
ENSRNOG00000012559	0.259764408	6.615699743	13.80460963	0.000202838	0.000929322
ENSRNOG00000013383	0.453313883	3.942482037	13.80051213	0.000203281	0.000931016
ENSRNOG00000004766	0.453296945	3.3932757	13.79195752	0.000204209	0.00093452
ENSRNOG00000056659	0.305082129	4.159397329	13.78304363	0.00020518	0.000938693
ENSRNOG00000020310	0.437209131	6.566742078	13.76496686	0.000207164	0.000947495
ENSRNOG00000048848	0.306335946	5.150450486	13.7517239	0.000208629	0.000953648
ENSRNOG00000013694	0.851101526	2.081067011	13.74816193	0.000209025	0.000955182
ENSRNOG00000030597	0.345882936	6.505326028	13.74353573	0.000209541	0.000956985
ENSRNOG00000016015	0.250743901	4.880563845	13.74148063	0.00020977	0.00095748
ENSRNOG00000014963	0.293014286	7.525584298	13.73874542	0.000210076	0.000958323
ENSRNOG00000012835	0.831409931	-0.278497854	13.68356445	0.00021634	0.000984062
ENSRNOG00000054879	0.817009058	2.214968639	13.67784179	0.000217	0.000986782
ENSRNOG00000007307	0.706054209	1.843150159	13.67538898	0.000217283	0.000987788
ENSRNOG00000016280	0.240496512	6.866838321	13.66628944	0.000218339	0.000992017
ENSRNOG00000014132	0.367490285	4.024545337	13.66028559	0.000219038	0.000994623
ENSRNOG00000012710	0.245137447	5.431605414	13.6562281	0.000219512	0.000996489
ENSRNOG00000012175	0.697131739	2.959215712	13.65227401	0.000219975	0.000998303
ENSRNOG00000016218	0.242720641	7.210245234	13.64584124	0.00022073	0.001001155
ENSRNOG00000046271	1.152974384	0.1355116	13.64412964	0.000220931	0.001001782
ENSRNOG00000024578	0.63045672	4.89368789	13.64191472	0.000221192	0.001002677
ENSRNOG00000057589	0.38268446	6.46935778	13.62321385	0.000223406	0.001011845
ENSRNOG00000012939	0.650358417	4.44324066	13.62082001	0.000223691	0.001012267
ENSRNOG00000010600	0.860656154	-0.245646041	13.61576593	0.000224294	0.001014216
ENSRNOG00000005109	0.690665789	2.323150165	13.59899041	0.000226307	0.001022645

ENSRNOG00000036683	0.405785531	3.043392042	13.58466397	0.000228041	0.001029892
ENSRNOG00000030408	0.259918536	5.046761636	13.57909954	0.000228718	0.001032655
ENSRNOG00000015914	0.347189925	6.254977179	13.56191288	0.000230822	0.001041559
ENSRNOG00000019824	0.397637506	3.667003647	13.5589588	0.000231185	0.001042903
ENSRNOG00000029127	0.622668054	0.363083001	13.55268176	0.00023196	0.0010458
ENSRNOG00000045623	0.357221636	4.22237693	13.54316673	0.000233139	0.001050219
ENSRNOG00000046229	0.712606057	2.231815795	13.54328922	0.000233123	0.001050219
ENSRNOG00000031269	0.360728536	6.197886151	13.53445145	0.000234224	0.001054807
ENSRNOG00000049849	0.572350268	1.266156953	13.52747675	0.000235096	0.001058433
ENSRNOG00000001376	0.328408189	5.493270437	13.49919993	0.000238665	0.001073892
ENSRNOG00000031816	0.534777946	5.535711345	13.48196187	0.000240868	0.001082572
ENSRNOG00000019807	0.555955921	2.527425337	13.47598164	0.000241637	0.00108572
ENSRNOG00000014742	0.45367986	4.51554451	13.4730438	0.000242015	0.001087104
ENSRNOG00000045911	0.985737862	-1.13171338	13.47252686	0.000242082	0.001087104
ENSRNOG00000016367	0.317959242	3.232489848	13.46087193	0.000243591	0.001092692
ENSRNOG00000014044	0.244889672	5.115400685	13.46077862	0.000243603	0.001092692
ENSRNOG00000054355	0.639209933	0.29025879	13.45701383	0.000244092	0.001094577
ENSRNOG00000020833	0.267974621	6.66650823	13.44734988	0.000245353	0.001099918
ENSRNOG00000008526	0.504265196	1.258432866	13.44274602	0.000245955	0.001101997
ENSRNOG00000058245	0.437556808	4.13534277	13.43808306	0.000246567	0.001104114
ENSRNOG00000017613	0.223398681	5.177601844	13.41205336	0.000250013	0.001118277
ENSRNOG00000012807	0.415164924	5.188927644	13.40837622	0.000250503	0.001120155
ENSRNOG00000001383	0.440626531	2.202510426	13.40576641	0.000250852	0.001121397
ENSRNOG00000062192	0.93087589	-0.621312249	13.39634339	0.000252115	0.001126091
ENSRNOG00000010977	0.944219145	0.758506162	13.39393796	0.000252439	0.001127218
ENSRNOG00000027264	0.499919175	5.776385405	13.38642265	0.000253452	0.001131106
ENSRNOG00000014619	0.288570019	4.801540489	13.37924877	0.000254424	0.001134801
ENSRNOG00000027954	0.602452602	1.810729189	13.37590218	0.000254878	0.001136508
ENSRNOG00000008815	0.441800995	2.860005811	13.36197123	0.000256778	0.001144659
ENSRNOG00000016001	0.32252661	6.1615509	13.35960539	0.000257102	0.001145781
ENSRNOG00000045926	0.282371944	4.796722079	13.33422023	0.000260606	0.001158787
ENSRNOG00000005758	0.6797687	3.799877121	13.30631837	0.000264513	0.001173523
ENSRNOG00000004598	0.43206453	2.495891055	13.30448449	0.000264772	0.001174342
ENSRNOG00000028063	0.4015563	3.575267437	13.29894665	0.000265555	0.001177156
ENSRNOG00000040242	0.394967109	2.339429475	13.29087064	0.000266702	0.001181246
ENSRNOG00000018227	0.262163939	5.433649779	13.28903549	0.000266963	0.001181411
ENSRNOG00000019653	0.346038831	4.365651875	13.28661645	0.000267307	0.001182606
ENSRNOG00000006697	0.295877404	5.965757937	13.27870701	0.000268438	0.001187274
ENSRNOG00000005535	0.682352663	1.548610259	13.27364667	0.000269163	0.001189818
ENSRNOG00000003234	0.260194695	7.359876874	13.26760101	0.000270033	0.001193328
ENSRNOG00000060429	0.378558785	2.751024507	13.25708316	0.000271552	0.001199038
ENSRNOG00000017116	0.20978897	5.909631398	13.25731383	0.000271518	0.001199038
ENSRNOG00000015413	0.301122882	4.209787183	13.25085152	0.000272456	0.001202025
ENSRNOG00000002251	0.262316016	5.388779257	13.25094178	0.000272443	0.001202025
ENSRNOG00000007268	0.210937476	4.910119669	13.24670068	0.00027306	0.001204019
ENSRNOG00000020881	0.336660201	3.844360932	13.23458092	0.000274831	0.001211155
ENSRNOG00000010104	0.322463182	3.862054637	13.22740035	0.000275886	0.001215285
ENSRNOG00000001876	1.01684487	-0.354773144	13.22715615	0.000275922	0.001215285
ENSRNOG00000011203	0.380489477	5.34722901	13.2194272	0.000277062	0.001219714
ENSRNOG00000024961	0.42060838	3.939353922	13.21368484	0.000277912	0.001222352
ENSRNOG00000001482	0.363896248	3.516724844	13.20998045	0.000278462	0.00122443
ENSRNOG00000000839	0.538480712	5.531416878	13.20876295	0.000278643	0.001224885
ENSRNOG00000016097	0.305938963	3.890323759	13.2046899	0.000279249	0.001226973
ENSRNOG00000013689	0.30800348	4.723827021	13.20453049	0.000279273	0.001226973
ENSRNOG00000020474	0.422749439	5.534656655	13.20078249	0.000279832	0.001228879
ENSRNOG00000011427	0.74420939	4.175824217	13.20058155	0.000279862	0.001228879
ENSRNOG00000001351	0.254787228	5.901560676	13.19894392	0.000280107	0.001229613
ENSRNOG00000013171	0.804807791	1.225108797	13.19746547	0.000280328	0.001230242
ENSRNOG00000001080	0.404897125	5.1373764	13.19591038	0.000280561	0.001230922
ENSRNOG00000009042	0.216728376	6.45469216	13.19469844	0.000280742	0.001231377
ENSRNOG00000060665	0.258041703	5.113648802	13.18010411	0.000282937	0.00124066
ENSRNOG00000011407	0.455818205	4.20586498	13.14739618	0.000287919	0.001261109
ENSRNOG00000006028	0.248708673	5.592593693	13.14574642	0.000288173	0.001261871
ENSRNOG00000056658	0.294113964	4.211979435	13.13436476	0.000289929	0.001269209
ENSRNOG00000020651	0.272802324	5.320648549	13.11636162	0.000292728	0.001280947
ENSRNOG00000000307	0.460957942	1.673223843	13.11376175	0.000293134	0.00128218
ENSRNOG00000018364	0.276762369	6.01354378	13.10812597	0.000294018	0.001285688
ENSRNOG00000013267	0.457677819	2.258535491	13.08487352	0.00029769	0.00130031
ENSRNOG00000012394	0.328159454	3.843415053	13.08504534	0.000297663	0.00130031
ENSRNOG00000060598	0.662959881	0.70607321	13.06482742	0.000300893	0.001312129
ENSRNOG00000057556	0.593491971	3.316367605	13.06264295	0.000301244	0.001313298
ENSRNOG00000023546	0.597980827	1.992248329	13.05965946	0.000301724	0.001314668
ENSRNOG00000060120	0.238369246	4.93174092	13.03958851	0.000304975	0.001326642
ENSRNOG00000046268	0.585232997	1.031972353	13.03620052	0.000305527	0.001328674
ENSRNOG00000001309	0.785916611	4.153772677	13.03569275	0.00030561	0.001328674
ENSRNOG00000025612	0.527075174	7.047240618	13.02115841	0.000307991	0.001337924
ENSRNOG00000019271	0.409530959	2.532590831	13.0165422	0.000308751	0.001340858
ENSRNOG00000021287	0.626871302	2.859356313	13.001197	0.000311292	0.00135078
ENSRNOG00000013642	0.303615393	5.258927665	12.9917972	0.000312859	0.001356834
ENSRNOG00000005918	0.670269671	-0.163188285	12.97387746	0.000315867	0.001369132
ENSRNOG00000014795	0.839275922	3.995933958	12.96507525	0.000317356	0.001374832
ENSRNOG00000021232	0.260331674	5.717978274	12.93423064	0.000322628	0.001395762
ENSRNOG00000001874	0.498002274	4.505603503	12.9246445	0.000324284	0.001401779
ENSRNOG00000002851	0.24608375	5.285899771	12.88767292	0.000330753	0.001427016
ENSRNOG00000025498	1.446084409	0.086203223	12.87860506	0.00033236	0.001433557
ENSRNOG00000026994	0.308546385	4.090973841	12.87209902	0.000333517	0.001437374
ENSRNOG00000019713	0.366059179	4.311088206	12.85768401	0.000336096	0.001447307
ENSRNOG00000014744	0.429347198	7.282596023	12.8564834	0.000336312	0.001447842
ENSRNOG00000009373	0.509258415	2.184580848	12.85351412	0.000336846	0.001449094
ENSRNOG00000017441	0.247998141	6.368569882	12.84739346	0.00033795	0.001452846
ENSRNOG00000006393	0.71816202	3.453154286	12.84663473	0.000338087	0.001452846
ENSRNOG00000000699	0.698315969	4.595298792	12.8468196	0.000338053	0.001452846
ENSRNOG00000018369	0.573368839	0.909203279	12.83049124	0.000341016	0.001463717
ENSRNOG00000010302	1.62683318	-1.022499756	12.82708015	0.000341638	0.00146599
ENSRNOG00000032618	0.855867062	-0.523649109	12.81519507	0.000343816	0.001473497
ENSRNOG00000019960	0.300743005	5.119095625	12.79847709	0.000346902	0.001485354
ENSRNOG00000022274	0.378168734	4.750408414	12.79430099	0.000347677	0.001488271
ENSRNOG00000037720	0.402447185	3.700542795	12.7916636	0.000348167	0.001489968
ENSRNOG00000051249	1.42687288	-0.986921826	12.77113173	0.00035201	0.001504785
ENSRNOG00000015206	0.465287227	3.604744657	12.76095806	0.00035393	0.001512175

ENSRNOG00000003235	0.398427007	4.860764995	12.75175784	0.000355675	0.001519221
ENSRNOG00000001254	0.561959796	2.904362341	12.73517318	0.000358843	0.001531513
ENSRNOG00000018450	0.804863522	1.298248744	12.73106611	0.000359631	0.001534053
ENSRNOG00000026981	0.29388274	3.760502304	12.7315203	0.000359544	0.001534053
ENSRNOG00000018012	0.227835093	6.864541058	12.72229498	0.000361322	0.001540849
ENSRNOG00000021125	0.380936737	6.300253118	12.72139943	0.000361495	0.001541172
ENSRNOG00000014852	0.385524561	3.866749911	12.69799502	0.000366048	0.001558484
ENSRNOG00000003073	0.435494731	2.769113026	12.68642717	0.000368319	0.001566891
ENSRNOG00000017354	0.501329425	3.803287833	12.68592295	0.000368419	0.001566893
ENSRNOG00000023993	0.421772643	9.481986341	12.66002245	0.000373558	0.001587044
ENSRNOG00000047734	0.64385664	5.900506964	12.65902969	0.000373756	0.001587461
ENSRNOG00000033893	0.448546299	5.870694472	12.65697508	0.000374167	0.00158878
ENSRNOG00000014316	0.302727993	5.797695527	12.65568581	0.000374425	0.001589024
ENSRNOG00000015456	0.404711995	2.339570944	12.65172714	0.000375219	0.001591966
ENSRNOG00000016584	0.453191994	1.321460465	12.64291643	0.000376991	0.001598629
ENSRNOG00000013922	0.487857799	1.290611734	12.63206149	0.000379187	0.001607078
ENSRNOG00000020830	0.9599485	-0.96768415	12.62925296	0.000379757	0.001608584
ENSRNOG00000019228	0.485925145	6.08215737	12.62880951	0.000379847	0.001608584
ENSRNOG00000011171	0.943251423	4.445912995	12.62067194	0.000381504	0.001614307
ENSRNOG00000020583	0.395579211	3.911189371	12.58738526	0.000388359	0.001640682
ENSRNOG00000020813	0.441746793	4.477741622	12.58050982	0.00038979	0.001644974
ENSRNOG00000019843	0.348604462	3.907950805	12.58098245	0.000389692	0.001644974
ENSRNOG00000036516	0.510653048	1.102101884	12.57904318	0.000390096	0.001645826
ENSRNOG00000020353	0.492902604	3.486828023	12.56624126	0.000392778	0.001654054
ENSRNOG00000001103	0.483364344	3.087475229	12.56675179	0.00039267	0.001654054
ENSRNOG00000027552	0.405290424	3.026665511	12.54399143	0.000397482	0.001672087
ENSRNOG00000019262	0.276227237	6.510474365	12.5420407	0.000397898	0.001673389
ENSRNOG00000016023	0.410509066	5.278205014	12.53673659	0.000399029	0.0016777
ENSRNOG00000000487	0.682827904	3.745503223	12.52722955	0.000401064	0.001685363
ENSRNOG00000049056	0.389493515	4.421555148	12.51861536	0.000402917	0.001691804
ENSRNOG00000060047	1.073810851	-0.83243686	12.50980057	0.000404823	0.001698904
ENSRNOG00000014135	0.33771911	6.806007797	12.48842699	0.000409481	0.001717087
ENSRNOG00000010526	0.313472084	5.305324138	12.44965172	0.00041807	0.001752175
ENSRNOG00000061944	0.447846358	1.775667816	12.43551527	0.000421246	0.001764086
ENSRNOG00000006209	0.421093393	4.949077362	12.42630778	0.000423328	0.001771868
ENSRNOG00000032828	0.377165189	7.572879106	12.41682147	0.000425484	0.00177948
ENSRNOG00000015267	1.064759135	-0.837954029	12.40749925	0.000427613	0.001787609
ENSRNOG00000010589	0.471243406	1.934594602	12.37135995	0.00043597	0.001820931
ENSRNOG00000021812	0.926458458	-0.489583642	12.32397677	0.000447177	0.001864788
ENSRNOG00000016907	0.345369906	6.193658932	12.3174259	0.000448749	0.001869867
ENSRNOG00000008510	0.708998602	2.406436353	12.3114173	0.000450196	0.001875402
ENSRNOG00000003285	0.504555401	1.066939263	12.30614105	0.00045147	0.001880216
ENSRNOG00000045615	0.657462954	0.790375495	12.29906509	0.000453185	0.001886366
ENSRNOG00000006570	0.518533674	5.295117257	12.29661478	0.000453781	0.001888347
ENSRNOG00000025327	0.434031369	1.299117152	12.28702124	0.000456119	0.001896584
ENSRNOG00000018602	0.349611131	6.264486151	12.2792704	0.000458017	0.001903977
ENSRNOG00000048174	0.378586734	5.109760216	12.27545299	0.000458955	0.001907375
ENSRNOG00000001750	0.879200058	2.148564598	12.27072822	0.000460119	0.001911207
ENSRNOG00000016000	1.042786953	1.532564799	12.26675717	0.000461099	0.001914775
ENSRNOG00000015229	0.222676812	5.682780817	12.26196716	0.000462284	0.00191869
ENSRNOG00000061526	0.829858463	0.149866387	12.2479288	0.000465775	0.001932166
ENSRNOG00000049906	0.662811603	-0.080124862	12.2453893	0.000466409	0.001934291
ENSRNOG00000015886	0.256553708	3.812739549	12.23885594	0.000468045	0.001939684
ENSRNOG00000012053	0.330584911	6.14761731	12.23689839	0.000468536	0.00194108
ENSRNOG00000001457	0.333973513	3.321009151	12.23255467	0.000469629	0.001945095
ENSRNOG00000049025	0.985581359	2.006827619	12.22706453	0.000471012	0.001949807
ENSRNOG00000016539	0.294624394	6.506991312	12.21302142	0.000474571	0.001963512
ENSRNOG00000018242	0.302859472	6.489708716	12.1995835	0.000478002	0.001976673
ENSRNOG00000002542	0.294949783	3.230345478	12.19989193	0.000477923	0.001976673
ENSRNOG00000023109	0.354211015	2.798262783	12.18370898	0.000482087	0.001992525
ENSRNOG00000018145	0.28393493	6.077489018	12.17947678	0.000483182	0.00199653
ENSRNOG00000057713	0.449435372	4.377517461	12.17782718	0.000483609	0.001997775
ENSRNOG00000007663	0.984019205	2.009101848	12.17403832	0.000484593	0.002001315
ENSRNOG00000009180	0.47616524	1.395551237	12.1730138	0.000484859	0.002001892
ENSRNOG00000037919	0.433656118	3.090303237	12.17035458	0.000485551	0.002004226
ENSRNOG00000039559	0.286020463	6.910806373	12.16537265	0.000486849	0.002009063
ENSRNOG00000020407	0.280814959	7.430194968	12.15081909	0.000490663	0.002022165
ENSRNOG00000009590	0.238280926	6.474534686	12.13683717	0.000494355	0.002035793
ENSRNOG00000023352	0.512902608	0.842054695	12.12528894	0.000497426	0.002046842
ENSRNOG00000031364	0.487704702	7.547205129	12.11476709	0.000500241	0.002057354
ENSRNOG00000018390	0.692096369	7.616007153	12.1025467	0.00050353	0.002069269
ENSRNOG00000017095	0.758541772	-0.585879668	12.10045488	0.000504095	0.002070517
ENSRNOG00000000799	0.220175801	6.357683589	12.09200306	0.000506385	0.002078845
ENSRNOG00000007271	0.550107702	4.183763316	12.09025884	0.000506859	0.002080252
ENSRNOG00000019474	0.894188405	3.161551442	12.06656367	0.000513342	0.002104677
ENSRNOG00000020097	0.560514298	2.980354002	12.06569922	0.00051358	0.002105108
ENSRNOG00000039415	0.535054411	5.453976596	12.06482372	0.000513822	0.002105552
ENSRNOG00000049593	1.092832248	-0.175577855	12.06061278	0.000514984	0.002109767
ENSRNOG00000027459	0.405107651	3.375473572	12.05131765	0.000517558	0.002118669
ENSRNOG00000017408	0.277781782	5.325382835	12.05146025	0.000517518	0.002118669
ENSRNOG00000020812	0.2755886	4.531708523	12.04013636	0.000520672	0.002130314
ENSRNOG00000006947	0.233722671	5.052806644	12.03540074	0.000521996	0.002135181
ENSRNOG00000004733	0.408267891	1.665612841	12.03172882	0.000523025	0.002137468
ENSRNOG00000008703	0.192421795	6.300699038	12.03154102	0.000523078	0.002137468
ENSRNOG00000049045	0.866769273	-0.625505593	12.02342749	0.00052536	0.002145062
ENSRNOG00000027849	0.292188572	4.150987613	12.00730998	0.000529923	0.002161463
ENSRNOG00000019532	0.409588409	5.403329071	12.00447401	0.00053073	0.002164197
ENSRNOG00000021086	0.529510875	2.862657861	11.99082271	0.000534632	0.002178986
ENSRNOG00000011884	0.289547539	5.969825381	11.98882787	0.000535204	0.002180197
ENSRNOG00000030775	0.367624944	3.509621527	11.98770563	0.000535527	0.00218082
ENSRNOG00000050312	0.635345095	0.109131243	11.97890999	0.00053806	0.002189578
ENSRNOG00000023269	0.714156695	-0.3991243	11.97734306	0.000538513	0.002190295
ENSRNOG00000002388	0.309150128	5.534091988	11.96734521	0.00054141	0.002201512
ENSRNOG00000013783	0.511479455	6.132977246	11.95852983	0.000543978	0.00221025
ENSRNOG00000013456	0.370355803	4.298749582	11.94819896	0.000547002	0.002221969
ENSRNOG00000013169	0.477451959	3.364903795	11.94435343	0.000548132	0.002225989
ENSRNOG00000012172	0.538921202	1.274320027	11.9410298	0.000549111	0.002229391
ENSRNOG00000016090	0.274315368	5.476627255	11.91837744	0.000555829	0.002253198
ENSRNOG00000051470	0.567651297	2.104466579	11.90419381	0.000560077	0.002269258
ENSRNOG00000021102	0.497819305	6.022203196	11.89305814	0.000563435	0.002281697

ENSRNOG00000045941	0.251238794	4.989758826	11.89329573	0.000563364	0.002281697
ENSRNOG00000047276	0.623377505	2.559568591	11.87817291	0.000567956	0.002299417
ENSRNOG00000017071	0.398836928	5.196674847	11.87212247	0.000569804	0.002306309
ENSRNOG00000014644	0.801384571	4.242475762	11.86634935	0.000571573	0.002312288
ENSRNOG00000016611	0.347378665	5.566230969	11.85973763	0.000573606	0.002319326
ENSRNOG00000000894	0.311689045	7.187863768	11.84705656	0.000577526	0.002334578
ENSRNOG00000005062	0.946315118	-1.163744478	11.84598929	0.000577857	0.00233532
ENSRNOG00000018552	0.268308603	4.15269465	11.84540562	0.000578038	0.002335456
ENSRNOG00000027098	0.354185875	7.445605207	11.84329945	0.000578692	0.002337502
ENSRNOG00000016358	0.281013439	6.753299163	11.83961702	0.000579838	0.002340338
ENSRNOG00000021139	0.424050449	4.668712775	11.8388224	0.000580085	0.002340741
ENSRNOG00000020067	0.323609819	4.140416549	11.82282789	0.000585089	0.00235853
ENSRNOG00000010421	0.283281646	4.262697314	11.82131328	0.000585565	0.002359248
ENSRNOG00000022753	0.578912723	7.127945872	11.82083937	0.000585714	0.002359248
ENSRNOG00000014199	0.51393089	2.88436124	11.8198962	0.000586011	0.002359843
ENSRNOG00000017646	0.759559476	0.703786504	11.79407575	0.000594195	0.002390974
ENSRNOG00000020869	0.270284675	4.479746712	11.7832935	0.000597647	0.002403782
ENSRNOG00000001593	0.287247328	4.562864767	11.77847046	0.000599197	0.002408653
ENSRNOG00000026435	0.342765256	2.832298149	11.77852659	0.000599179	0.002408653
ENSRNOG00000027368	0.791745654	-0.767928085	11.77269157	0.00060106	0.002415529
ENSRNOG00000062202	0.656662811	0.351277378	11.77047426	0.000601776	0.002417795
ENSRNOG00000049552	0.94664415	-0.282042779	11.75844274	0.000605679	0.002431006
ENSRNOG00000020225	0.470807484	6.277974199	11.75511224	0.000606764	0.002434742
ENSRNOG00000054917	0.962723023	2.27899176	11.75181483	0.000607839	0.002437824
ENSRNOG00000019036	0.643529611	3.371277603	11.75220978	0.00060771	0.002437824
ENSRNOG00000061824	0.764546681	0.814857988	11.74856335	0.000608902	0.002441468
ENSRNOG00000000123	0.243566217	4.491291037	11.74034269	0.000611598	0.002451655
ENSRNOG00000011726	0.286687869	7.911043279	11.73935179	0.000611923	0.00245234
ENSRNOG00000011901	0.649646318	1.194423126	11.7350997	0.000613323	0.002457327
ENSRNOG00000013668	0.569088203	1.833188055	11.72805507	0.000615649	0.002466022
ENSRNOG00000015953	0.245935437	5.944639846	11.72400311	0.000616991	0.002470148
ENSRNOG00000014992	0.342800853	2.560619573	11.72438618	0.000616864	0.002470148
ENSRNOG00000043076	0.766252673	-0.426318212	11.72218573	0.000617594	0.002471936
ENSRNOG00000008037	0.412248745	3.456119555	11.72031907	0.000618214	0.002473792
ENSRNOG00000060914	0.500049202	3.609884586	11.71513642	0.000619938	0.002480064
ENSRNOG00000037960	1.242767422	2.417016242	11.70782072	0.00062238	0.002489204
ENSRNOG00000013737	0.382805594	4.010465209	11.6879341	0.000629067	0.002515315
ENSRNOG00000023393	0.303963835	4.963136482	11.6832707	0.000630646	0.002520991
ENSRNOG00000019743	0.304164849	6.237725286	11.67688908	0.000632813	0.002529014
ENSRNOG00000053560	0.564421798	2.800494008	11.67148013	0.000634655	0.002535378
ENSRNOG00000047781	0.43139307	7.422438104	11.66603286	0.000636516	0.002541249
ENSRNOG00000000777	0.571716123	3.927996049	11.66239354	0.000637762	0.002545583
ENSRNOG00000008994	0.260826167	6.170162443	11.65765625	0.000639388	0.002550788
ENSRNOG00000006911	0.428221194	6.521175117	11.65292999	0.000641015	0.002556632
ENSRNOG00000038766	0.280436973	3.955646016	11.64584885	0.00064346	0.002564445
ENSRNOG00000015191	0.226702488	5.321199572	11.6447297	0.000643847	0.002565343
ENSRNOG00000026589	0.352933492	6.374435037	11.64023305	0.000645405	0.002570258
ENSRNOG00000010258	0.289902177	5.527740965	11.63208798	0.000648237	0.002579591
ENSRNOG00000006341	0.612312283	0.593420275	11.63045283	0.000648808	0.002581211
ENSRNOG00000019340	0.364237175	4.504759864	11.62181103	0.000651829	0.002591278
ENSRNOG00000049714	0.704889811	0.643638251	11.62226467	0.00065167	0.002591278
ENSRNOG00000018391	0.290341352	3.469220064	11.61459392	0.000654363	0.0026007
ENSRNOG00000021712	0.297416669	4.521479755	11.60842183	0.000656538	0.002607381
ENSRNOG00000046982	0.811695024	-0.580361049	11.59108475	0.000662687	0.002630482
ENSRNOG00000055157	0.598727128	0.808652445	11.57122374	0.000669803	0.002656064
ENSRNOG00000029179	0.529383693	0.65680505	11.56619202	0.000671618	0.002661928
ENSRNOG00000017330	0.61823907	1.755233979	11.56102312	0.000673488	0.00266867
ENSRNOG00000016316	0.431075576	2.409939686	11.55841321	0.000674434	0.002670435
ENSRNOG00000002074	0.345143683	2.515599819	11.55884894	0.000674276	0.002670435
ENSRNOG00000013623	0.326102191	3.590239757	11.55793369	0.000674608	0.002670435
ENSRNOG00000018295	0.211294859	6.406474006	11.5581697	0.000674522	0.002670435
ENSRNOG00000016512	0.514318075	2.023361635	11.54561188	0.000679093	0.002684165
ENSRNOG00000000986	0.523227132	4.139325161	11.54157029	0.000680571	0.002689335
ENSRNOG00000007486	0.289255113	3.730922822	11.5297171	0.000684924	0.002705186
ENSRNOG00000001368	0.494148433	6.916860488	11.51937649	0.000688745	0.002718242
ENSRNOG00000002323	0.457386255	4.04457328	11.51731467	0.000689509	0.00272058
ENSRNOG00000049511	0.252058669	5.843851932	11.51116707	0.000691793	0.002727554
ENSRNOG00000042667	0.531646308	2.871794694	11.50918767	0.00069253	0.002729317
ENSRNOG00000024743	0.289239938	5.831975707	11.50568249	0.000693838	0.002732891
ENSRNOG00000053339	0.363527924	2.817972806	11.50093813	0.000695611	0.002739194
ENSRNOG00000018669	0.739927637	1.334987105	11.48703114	0.000700835	0.002756026
ENSRNOG00000051286	1.053364144	0.147433983	11.45933091	0.000711359	0.002792181
ENSRNOG00000016983	0.55854697	2.824601354	11.45658364	0.000712412	0.002795619
ENSRNOG00000054310	0.729872809	0.076335149	11.45277629	0.000713873	0.002800659
ENSRNOG00000022804	0.739639899	3.905288947	11.43333567	0.000721381	0.002828712
ENSRNOG00000048411	0.421712546	2.033599738	11.4213272	0.000726058	0.00284494
ENSRNOG00000001890	0.292776699	3.593646674	11.41914871	0.00072691	0.002847573
ENSRNOG00000046867	1.143773655	-0.796498789	11.41668962	0.000727873	0.00284923
ENSRNOG00000051180	1.049247446	0.144798199	11.41587459	0.000728192	0.002849776
ENSRNOG00000018421	0.29589491	3.259788138	11.40523876	0.000732373	0.002865429
ENSRNOG00000001055	0.326256928	4.433773936	11.39780629	0.000735309	0.002875495
ENSRNOG00000001877	0.353011879	5.16412916	11.39273197	0.00073732	0.002881056
ENSRNOG00000023210	0.185138725	6.808461271	11.39063351	0.000738154	0.002883059
ENSRNOG00000000471	0.608599609	1.021592824	11.38831182	0.000739077	0.002884766
ENSRNOG00000028743	0.504810699	4.00367894	11.38115431	0.00074193	0.002894953
ENSRNOG00000001236	0.312430343	4.131944092	11.37760259	0.00074335	0.002899206
ENSRNOG00000004423	0.875051394	4.545897712	11.37751233	0.000743386	0.002899206
ENSRNOG00000003603	0.374419939	5.894113457	11.37639747	0.000743832	0.002900232
ENSRNOG00000003144	0.714870069	1.142349216	11.36657482	0.000747776	0.002914175
ENSRNOG00000046891	0.247842053	7.696075554	11.33967757	0.000758685	0.002954505
ENSRNOG00000050206	0.493210309	3.242561915	11.33216514	0.00076176	0.002965752
ENSRNOG00000054344	0.559275314	1.098923079	11.29503496	0.000777146	0.003021939
ENSRNOG00000023816	0.259212008	5.346270464	11.27544765	0.000785389	0.003050994
ENSRNOG00000025443	0.375703003	6.24707876	11.26370058	0.000790375	0.003068856
ENSRNOG00000021638	0.223422788	5.34738591	11.2488298	0.000796732	0.003092782
ENSRNOG00000019141	0.60488887	0.236358305	11.24440441	0.000798634	0.003099405
ENSRNOG00000033921	0.551363896	2.028801326	11.2436932	0.00079894	0.003099833
ENSRNOG00000000479	1.083268537	-0.054656631	11.23442841	0.000802938	0.00311382
ENSRNOG00000028717	0.520124198	5.037419715	11.23244155	0.000803798	0.003116391
ENSRNOG00000035516	1.141723274	-0.888841888	11.23099959	0.000804423	0.00311805

ENSRNOG00000011684	0.330155348	5.560034592	11.21555284	0.000811146	0.003141803
ENSRNOG00000023126	0.918410782	1.613231283	11.21112159	0.000813086	0.003148544
ENSRNOG00000013141	0.496457555	1.373188813	11.20767658	0.000814596	0.003152853
ENSRNOG00000001289	0.396614592	3.684143151	11.19418921	0.000820539	0.003171976
ENSRNOG00000021054	0.28710808	5.29162856	11.18780791	0.000823366	0.003182126
ENSRNOG00000029738	0.491586103	5.906533626	11.18007874	0.000826803	0.003192293
ENSRNOG00000012438	0.229156573	4.446974948	11.16999458	0.000831309	0.003208908
ENSRNOG00000021828	0.248906943	5.850148033	11.16908486	0.000831716	0.0032097
ENSRNOG00000022725	0.377468935	3.040987207	11.16477842	0.000833649	0.003214808
ENSRNOG00000024144	0.246642222	5.976147505	11.14337383	0.000843324	0.003249741
ENSRNOG00000053232	0.656380759	0.438684112	11.12796971	0.000850356	0.003273654
ENSRNOG00000003464	0.326219521	5.885788337	11.12658562	0.000850991	0.003274506
ENSRNOG00000023989	0.354738438	2.686402726	11.09776346	0.000864319	0.00332256
ENSRNOG00000049298	1.095406827	-0.887909174	11.09349851	0.000866309	0.003328594
ENSRNOG00000021150	0.37521063	3.372857106	11.09097913	0.000867487	0.003331502
ENSRNOG00000033100	0.441932355	2.223552785	11.07850108	0.000873344	0.003353182
ENSRNOG00000015195	0.425692306	2.534817674	11.06158179	0.000881349	0.00338146
ENSRNOG00000020617	0.345049149	3.079860202	11.04360569	0.000889936	0.003411099
ENSRNOG00000027099	0.34175931	3.76392236	11.03821962	0.000892525	0.003420195
ENSRNOG00000060008	0.223236121	4.720625646	11.03351678	0.000894792	0.003428053
ENSRNOG00000051977	0.372118866	2.73843495	11.02684299	0.00089802	0.003439584
ENSRNOG00000005505	0.773709477	0.295145733	11.02355715	0.000899613	0.003444853
ENSRNOG00000008099	0.875997226	-0.719850392	11.01507674	0.000903738	0.003458131
ENSRNOG00000003661	0.263640468	5.39056029	11.01418596	0.000904172	0.003458131
ENSRNOG00000025418	0.248601558	4.984336802	11.00991679	0.000906257	0.003465268
ENSRNOG00000010021	0.264050796	3.706911303	11.00355827	0.000909371	0.003474659
ENSRNOG00000026059	0.666805453	3.664347291	11.00021738	0.000911012	0.003480088
ENSRNOG00000008529	0.966599444	-0.488163927	10.98959813	0.000916247	0.003497712
ENSRNOG00000013503	0.608905084	3.100071387	10.98951408	0.000916288	0.003497712
ENSRNOG00000022419	0.662410144	1.155777523	10.98850762	0.000916786	0.003497925
ENSRNOG00000008239	0.359897663	4.760987886	10.97618093	0.000922904	0.003519573
ENSRNOG00000046022	0.882594181	-0.609960249	10.97310946	0.000924435	0.003524562
ENSRNOG00000019093	0.543457643	1.712053889	10.97253208	0.000924723	0.003524812
ENSRNOG00000037505	0.286697293	5.89603455	10.96313149	0.000929426	0.003541885
ENSRNOG00000048295	0.726559264	-0.747514983	10.95407927	0.000933978	0.003557518
ENSRNOG00000000248	0.288110741	6.794387893	10.93515548	0.000943566	0.00359231
ENSRNOG00000004461	0.713457586	2.140124436	10.91786132	0.000952415	0.003621647
ENSRNOG00000021184	0.411396593	5.797786153	10.91492374	0.000953926	0.003625834
ENSRNOG00000012843	0.980366985	-1.112199875	10.91483143	0.000953974	0.003625834
ENSRNOG00000011391	0.209843479	5.960329377	10.89805695	0.000962652	0.003657938
ENSRNOG00000021244	0.400034478	2.945274688	10.89603476	0.000963703	0.003661055
ENSRNOG00000031129	0.251073304	6.020416851	10.8812686	0.000971417	0.00368682
ENSRNOG00000001120	0.345847356	6.30352521	10.88030332	0.000971923	0.003686975
ENSRNOG00000009922	0.752163989	1.673622125	10.87797368	0.000973147	0.003689847
ENSRNOG00000061348	0.352195992	4.990584343	10.87552974	0.000974432	0.003691992
ENSRNOG00000046924	0.723535774	-0.48127046	10.8636309	0.000980712	0.003712689
ENSRNOG00000010170	0.37689937	7.450167474	10.86123198	0.000981983	0.003716235
ENSRNOG00000007025	0.417592363	1.022564589	10.85337006	0.000986161	0.003731153
ENSRNOG00000056768	0.280679517	3.984961697	10.84729254	0.000989403	0.003741631
ENSRNOG00000000288	0.583801244	1.38479673	10.83400921	0.000996526	0.00376587
ENSRNOG00000020930	0.340605991	5.84659584	10.83148764	0.000997884	0.003770103
ENSRNOG00000055915	0.213231293	4.775338736	10.81163124	0.001008644	0.0038075
ENSRNOG00000002635	0.311465923	4.008688005	10.80291365	0.001013405	0.003824178
ENSRNOG00000010370	0.292805037	4.930182881	10.78613214	0.001022633	0.003857165
ENSRNOG00000030927	0.944183709	0.509672423	10.77388343	0.001029423	0.003881082
ENSRNOG00000021714	0.723933563	1.618096556	10.77380812	0.001029465	0.003881082
ENSRNOG00000032160	0.9040684	-1.008090296	10.77246999	0.001030209	0.003882041
ENSRNOG00000026974	0.456675938	4.002350743	10.76835643	0.001032502	0.003889752
ENSRNOG00000017503	0.425689231	2.761415188	10.76513244	0.001034302	0.003894681
ENSRNOG00000010897	0.219528346	5.064702878	10.76089907	0.00103667	0.003902671
ENSRNOG00000047598	0.294156361	4.126874705	10.75947853	0.001037466	0.003903866
ENSRNOG00000007752	0.341219692	2.549429273	10.74697598	0.001044499	0.003928406
ENSRNOG00000050834	0.267686686	5.670408023	10.73354809	0.001052105	0.003955136
ENSRNOG00000017512	0.597380331	2.848477947	10.70221543	0.001070073	0.004013241
ENSRNOG00000010484	0.432571457	4.347711698	10.69540913	0.001074017	0.004026992
ENSRNOG00000014137	0.46035001	3.717771024	10.69267852	0.001075604	0.004031986
ENSRNOG00000027032	0.309591536	3.428754736	10.68566209	0.001079691	0.004042522
ENSRNOG00000012014	0.328454246	5.376710853	10.68283821	0.00108134	0.00404774
ENSRNOG00000023828	1.005995742	1.20837944	10.68151248	0.001082115	0.004049685
ENSRNOG00000020857	0.256414774	4.886175667	10.67715134	0.00108467	0.004057326
ENSRNOG00000061989	0.662650434	0.360351413	10.67651623	0.001085042	0.00405776
ENSRNOG00000033235	0.566380254	0.446190688	10.67462479	0.001086152	0.0040599
ENSRNOG00000007792	0.230780078	3.958162136	10.66413482	0.001092329	0.004081157
ENSRNOG00000012565	0.335275875	3.843263147	10.66313299	0.001092921	0.004082405
ENSRNOG00000018468	0.265497235	5.782320368	10.65073416	0.001100272	0.004107925
ENSRNOG00000007806	0.408331298	5.955341773	10.64853658	0.00110158	0.00411184
ENSRNOG00000033609	1.11841707	-0.087815371	10.64579785	0.001103213	0.004116962
ENSRNOG00000028100	0.456348901	0.910278921	10.63763297	0.001108094	0.004132257
ENSRNOG00000055564	0.845987631	2.662726968	10.63407962	0.001110225	0.004139229
ENSRNOG00000001868	0.528292848	2.753351303	10.62571071	0.001115261	0.004156047
ENSRNOG00000020979	0.396339912	6.273551363	10.61959007	0.001118959	0.004167864
ENSRNOG00000003018	0.445093515	2.624501645	10.61422024	0.001122213	0.004177037
ENSRNOG00000051706	0.365665941	4.195580627	10.60035581	0.001130659	0.00420551
ENSRNOG00000046086	0.905317534	-1.120988537	10.59319589	0.001135046	0.004220836
ENSRNOG00000013201	0.395914762	4.733182447	10.59004463	0.001136982	0.004226051
ENSRNOG00000004640	0.572900575	2.623645518	10.58621119	0.001139342	0.004233829
ENSRNOG00000019097	0.288099542	7.213401442	10.57706238	0.001144995	0.00425184
ENSRNOG00000002553	0.246580735	5.964610484	10.57348812	0.001147211	0.004258071
ENSRNOG00000032492	0.214326751	6.832503882	10.57004202	0.001149351	0.004265017
ENSRNOG00000046497	0.252920283	3.724594848	10.56593842	0.001151905	0.004272492
ENSRNOG00000010853	0.480117188	4.048279517	10.56179301	0.001154492	0.004281082
ENSRNOG00000036762	0.973866032	-1.163498914	10.55316502	0.001159893	0.004298092
ENSRNOG00000012808	0.500148139	5.102884919	10.55120441	0.001161124	0.004301647
ENSRNOG00000029814	0.876458229	-0.614446279	10.54594815	0.001164431	0.004311879
ENSRNOG00000032929	0.318430791	2.920284099	10.53880817	0.001168938	0.004327557
ENSRNOG00000016229	0.255664562	4.89250036	10.47276932	0.00121147	0.004472467
ENSRNOG00000039214	0.306445132	5.516244514	10.47302792	0.0012113	0.004472467
ENSRNOG00000016143	0.344065043	6.482439764	10.46861271	0.001214199	0.004481497
ENSRNOG00000061153	0.829578598	5.470131621	10.46202787	0.001218534	0.004495404
ENSRNOG00000012404	0.76509188	2.956783283	10.46054409	0.001219514	0.004496921

ENSRNOG00000021578	0.733383146	0.381749171	10.46080882	0.001219339	0.004496921
ENSRNOG00000010531	1.012914646	-0.374393151	10.45918811	0.001220409	0.004499175
ENSRNOG00000000885	0.576023411	3.811150745	10.45779739	0.001221328	0.004501515
ENSRNOG00000014768	0.503148577	1.342883444	10.45283423	0.001224614	0.004511526
ENSRNOG00000024104	0.214061473	4.841907231	10.44476614	0.001229975	0.004528787
ENSRNOG00000061818	1.1924542	-0.348616081	10.43715091	0.001235056	0.004542598
ENSRNOG00000003649	0.448204769	4.291488345	10.42374034	0.001244057	0.004573577
ENSRNOG00000015380	0.389610981	4.962182961	10.42195005	0.001245263	0.00457695
ENSRNOG00000055976	0.679545601	-0.14750381	10.41834905	0.001247694	0.004583755
ENSRNOG00000032381	0.39731459	2.284692624	10.41568234	0.001249497	0.004589314
ENSRNOG00000006876	1.253332659	0.746833088	10.41295008	0.001251347	0.004595043
ENSRNOG00000020577	0.426785075	7.199804175	10.41241263	0.001251711	0.004595315
ENSRNOG00000008257	0.885458589	1.174082496	10.40982915	0.001253463	0.004599877
ENSRNOG00000019311	0.44150306	1.286997273	10.40550462	0.001256402	0.004608263
ENSRNOG00000048456	0.978469984	1.264832634	10.40555956	0.001256365	0.004608263
ENSRNOG00000028103	0.267861111	6.414026522	10.39954662	0.001260463	0.004621015
ENSRNOG00000048523	0.976661653	1.263660705	10.39176915	0.001265783	0.004637299
ENSRNOG00000020110	0.948914475	-0.189235042	10.39006001	0.001266955	0.00464052
ENSRNOG00000050979	0.931011676	0.577138764	10.37563705	0.001276891	0.00467583
ENSRNOG00000018803	0.504211968	4.529004508	10.35315785	0.001292534	0.004725463
ENSRNOG00000028749	0.406433056	3.434584803	10.35161842	0.001293612	0.004727223
ENSRNOG00000019813	0.281986779	4.40827841	10.34293967	0.001299709	0.00474512
ENSRNOG00000000702	0.208493664	4.742380619	10.33319614	0.001306588	0.004769135
ENSRNOG00000049426	0.370040265	3.282173667	10.330967	0.001308167	0.004773798
ENSRNOG00000026065	0.729041093	5.230518724	10.32973406	0.001309041	0.004775541
ENSRNOG00000030264	0.74403712	1.882955142	10.32691062	0.001311045	0.004780995
ENSRNOG00000024452	0.253407609	6.089483495	10.31242319	0.001321377	0.004815346
ENSRNOG00000019689	0.528914168	4.938280145	10.29692338	0.001332522	0.004851495
ENSRNOG00000009448	1.180430868	0.514832849	10.29582395	0.001333316	0.00485327
ENSRNOG00000024140	0.269039761	4.352022529	10.29496501	0.001333937	0.004854414
ENSRNOG00000004518	0.415211504	6.44385886	10.28857475	0.001338564	0.004865663
ENSRNOG00000045553	0.602511011	0.371164414	10.28859205	0.001338552	0.004865663
ENSRNOG00000002763	0.220220858	6.573275436	10.28048214	0.001344448	0.004884599
ENSRNOG00000017993	0.336061032	4.82427535	10.27487563	0.00134854	0.004896303
ENSRNOG00000012363	0.301849922	3.17197178	10.27120893	0.001351222	0.004903794
ENSRNOG00000056737	0.589912737	1.590610115	10.26807414	0.00135352	0.004911008
ENSRNOG00000005393	0.442213523	2.257292954	10.25860471	0.001360485	0.004935149
ENSRNOG00000021140	0.932073362	1.963735002	10.25335267	0.001364364	0.004944688
ENSRNOG00000056135	0.246857802	6.591048994	10.25351607	0.001364243	0.004944688
ENSRNOG00000021316	0.509578217	3.324423126	10.2481888	0.001368189	0.004957414
ENSRNOG00000028701	0.463783442	1.409805269	10.24655727	0.001369399	0.004960665
ENSRNOG00000029826	0.297052527	3.762878408	10.24360413	0.001371593	0.004966341
ENSRNOG00000013179	0.465493486	2.956668497	10.24264892	0.001372304	0.00496738
ENSRNOG00000020601	0.41816533	2.305106138	10.24104021	0.001373501	0.004969839
ENSRNOG00000049385	0.531131436	1.243571894	10.22171974	0.001387964	0.005017584
ENSRNOG00000014805	0.408141739	3.065164912	10.21687037	0.001391618	0.005029646
ENSRNOG00000055382	0.520472781	3.404786509	10.21442388	0.001393465	0.005035173
ENSRNOG00000038909	0.384795786	4.976263965	10.20587734	0.001399938	0.005056253
ENSRNOG00000003970	0.203242435	7.436222032	10.19855393	0.001405508	0.005075215
ENSRNOG00000019028	0.239835643	4.20435859	10.17554364	0.001423157	0.005134261
ENSRNOG00000061876	0.28972694	5.131204913	10.16494097	0.001431365	0.005162695
ENSRNOG00000010617	0.856503282	2.248567346	10.1585581	0.001436329	0.005179421
ENSRNOG00000056073	0.342270148	2.814667358	10.15009731	0.001442936	0.005199693
ENSRNOG00000024239	0.376432029	4.277631507	10.14134491	0.001449803	0.005217315
ENSRNOG00000016288	0.210050003	6.042516603	10.1261347	0.001461816	0.005258156
ENSRNOG00000047874	0.214245181	5.101826795	10.11746074	0.001468712	0.005278163
ENSRNOG00000018051	0.202218502	5.479911884	10.11436044	0.001471185	0.00528585
ENSRNOG00000006787	0.526272222	6.614207712	10.11195182	0.001473109	0.005291562
ENSRNOG00000020585	0.803838905	0.233650318	10.0946936	0.001486969	0.005337716
ENSRNOG00000058618	0.536423779	1.654358285	10.0894695	0.00149119	0.005350444
ENSRNOG00000019268	0.229788789	5.507990377	10.08960997	0.001491077	0.005350444
ENSRNOG00000017446	0.364827065	4.916311681	10.08812808	0.001492276	0.005352122
ENSRNOG00000026432	0.384611977	5.536071385	10.0822739	0.001497025	0.005363073
ENSRNOG00000048258	0.236520271	6.375717104	10.08226004	0.001497036	0.005363073
ENSRNOG00000009617	1.031187178	-0.813795496	10.07850612	0.001500089	0.005371418
ENSRNOG00000041468	0.74104353	0.076710013	10.07473864	0.001503159	0.005381195
ENSRNOG00000014863	0.241230908	4.349775745	10.07314548	0.00150446	0.005384633
ENSRNOG00000005358	0.239101902	5.876541203	10.07117423	0.00150607	0.005389179
ENSRNOG00000029087	0.356530789	5.968287639	10.06834566	0.001508384	0.005396239
ENSRNOG00000056259	0.462190677	1.409489859	10.06689705	0.001509571	0.005399264
ENSRNOG00000011151	0.334248593	6.251547365	10.06399497	0.00151195	0.005405333
ENSRNOG00000018399	0.283943875	5.705677808	10.05878369	0.001516233	0.005415753
ENSRNOG00000054296	0.836265897	-1.067668559	10.05478622	0.001519526	0.00542526
ENSRNOG00000019850	0.408483371	5.955453185	10.03833674	0.001533156	0.005469176
ENSRNOG00000013777	0.262278766	3.710494227	10.0280405	0.00154175	0.005496975
ENSRNOG00000020867	0.438981285	4.931132653	10.0254659	0.001543906	0.005503424
ENSRNOG00000046594	1.011641229	-0.82517232	10.02256833	0.001546337	0.005510848
ENSRNOG00000005390	0.209516436	4.592734962	10.02033122	0.001548216	0.005515063
ENSRNOG00000019404	0.791101807	2.181011614	10.01551055	0.001552274	0.005528273
ENSRNOG00000008970	0.374104365	6.223027187	10.01142816	0.001555718	0.005539295
ENSRNOG00000006472	0.390936281	2.997694324	9.998309263	0.00156684	0.005571378
ENSRNOG00000009588	0.374717053	7.064551406	9.994714809	0.001569901	0.00558101
ENSRNOG00000020322	0.264050673	4.264887408	9.993066573	0.001571307	0.0055835
ENSRNOG00000000701	0.291968792	5.771027759	9.992151288	0.001572088	0.00558377
ENSRNOG00000059487	0.543419687	1.980503544	9.984895133	0.001578295	0.005603303
ENSRNOG00000033663	0.315569406	4.166647589	9.983407696	0.001579571	0.005606574
ENSRNOG00000019772	0.249295077	6.01731524	9.97847769	0.001583806	0.005620345
ENSRNOG00000059442	0.328785192	2.859339225	9.970443024	0.001590732	0.005638606
ENSRNOG00000018486	0.64177314	0.911088074	9.95187955	0.001606853	0.005693126
ENSRNOG00000022623	0.470188555	3.685097039	9.950847857	0.001607753	0.005693842
ENSRNOG00000015903	0.362599028	6.202902561	9.933603123	0.001622885	0.005742295
ENSRNOG00000057074	0.771266616	-0.545926219	9.929896285	0.001626157	0.0057513
ENSRNOG00000047656	0.718551474	8.35241676	9.928677795	0.001627234	0.005753823
ENSRNOG00000061499	0.256869425	7.50508606	9.913534942	0.001640677	0.005794887
ENSRNOG00000003105	0.672546872	1.791947648	9.903739812	0.001649432	0.005824512
ENSRNOG00000049351	0.283935436	7.090100648	9.902396442	0.001650637	0.005827466
ENSRNOG00000017030	0.255890456	5.958000141	9.899915393	0.001652864	0.005832727
ENSRNOG00000005568	0.269449108	6.256515889	9.900084433	0.001652712	0.005832727
ENSRNOG00000011526	0.334402313	3.944996334	9.890208018	0.001661606	0.00586227
ENSRNOG00000020519	0.501911148	5.540891021	9.879987627	0.001670861	0.005892399

ENSRNOG00000018047	0.287811998	4.933625696	9.876340034	0.001674176	0.005902673
ENSRNOG00000048922	0.561498284	1.218233463	9.874921362	0.001675468	0.005905911
ENSRNOG00000003147	0.287722676	8.403806925	9.858001729	0.001690947	0.005956496
ENSRNOG00000019922	0.369806494	4.202496417	9.852018333	0.001696455	0.005974572
ENSRNOG00000042888	0.391094372	2.301476104	9.849616154	0.001698672	0.005981048
ENSRNOG00000017837	0.263849248	3.557921983	9.84742419	0.001700697	0.005986848
ENSRNOG00000006060	0.594013608	2.973734251	9.841348489	0.001706324	0.006003984
ENSRNOG00000000044	0.198980738	5.370669409	9.837532865	0.001709867	0.006015114
ENSRNOG00000020956	0.309273651	3.045804535	9.823779908	0.001722699	0.006056222
ENSRNOG00000024503	0.197424301	5.496209917	9.813899693	0.001731979	0.006086141
ENSRNOG00000021800	0.619315861	-0.04790538	9.810463016	0.001735218	0.006096171
ENSRNOG00000050706	0.290327181	4.691201576	9.797213231	0.001747765	0.00613889
ENSRNOG00000032215	0.320943666	2.497453592	9.796148343	0.001748778	0.006141084
ENSRNOG00000017045	0.291811738	3.97317274	9.793033248	0.001751743	0.006148768
ENSRNOG00000006450	0.82194823	-0.879481818	9.790111824	0.001754528	0.00615718
ENSRNOG00000016779	0.303779338	6.394129926	9.777938063	0.001766183	0.006195334
ENSRNOG00000000663	0.209367136	5.089484174	9.776298471	0.001767758	0.006199487
ENSRNOG00000019129	0.927586073	0.661292854	9.746671129	0.001796477	0.006289061
ENSRNOG00000041787	0.70217299	-0.309279292	9.739536121	0.001803464	0.006310729
ENSRNOG00000031052	1.100603333	-0.779405064	9.723959654	0.001818812	0.006358816
ENSRNOG00000018730	0.541143821	2.888520116	9.716600909	0.001826109	0.006381509
ENSRNOG00000016394	0.824026073	-0.594482531	9.712818419	0.001829872	0.006393245
ENSRNOG00000006264	0.283865299	4.210027275	9.704272418	0.001838401	0.006421628
ENSRNOG00000025848	1.007164612	-1.10111304	9.694059324	0.001848647	0.006455994
ENSRNOG00000016021	0.33407841	3.799328039	9.683650898	0.001859148	0.006491236
ENSRNOG00000048036	0.357910996	5.958543613	9.681874517	0.001860947	0.006496083
ENSRNOG00000011066	0.277232341	6.814220861	9.676958746	0.001865932	0.00651205
ENSRNOG00000016870	0.235451336	4.97121195	9.673123519	0.001869831	0.006522783
ENSRNOG00000031517	0.568828643	0.06070373	9.673380364	0.00186957	0.006522783
ENSRNOG00000046834	0.64348341	3.43996775	9.668625648	0.001874415	0.006537331
ENSRNOG00000030704	0.434288004	1.080014149	9.642407341	0.001901359	0.006622551
ENSRNOG00000000502	0.834661911	-1.025099066	9.641037582	0.001902777	0.006626034
ENSRNOG00000006204	2.425035849	2.104839231	9.619883944	0.001924819	0.00669837
ENSRNOG00000038228	0.319306781	5.226951219	9.615995929	0.001928898	0.006711091
ENSRNOG00000011518	0.438802511	5.71118156	9.614906533	0.001930043	0.006713598
ENSRNOG00000036833	0.594702539	5.336730293	9.609874442	0.001935339	0.006730541
ENSRNOG00000027091	0.238494731	4.281962283	9.609375803	0.001935864	0.006730891
ENSRNOG00000017869	0.335051823	2.821691899	9.607865607	0.001937457	0.006734949
ENSRNOG00000019394	0.319548165	7.468340447	9.588291215	0.001958221	0.006799665
ENSRNOG00000011295	0.275352171	5.998121567	9.587473635	0.001959093	0.006801202
ENSRNOG00000061731	0.563805329	5.626313657	9.557660284	0.001991167	0.006901958
ENSRNOG00000010473	0.549199407	3.072996232	9.557116872	0.001991757	0.00690249
ENSRNOG00000008614	0.210404535	4.693691044	9.555085097	0.001993963	0.006907872
ENSRNOG00000000435	0.343926937	4.162559927	9.55488279	0.001994182	0.006907872
ENSRNOG00000025676	0.576040554	0.636490674	9.550387191	0.001999072	0.00692027
ENSRNOG00000047717	0.507002287	0.185412326	9.54487468	0.002005085	0.00693805
ENSRNOG00000053875	0.446191007	5.704980147	9.54252163	0.002007657	0.006945433
ENSRNOG00000008106	0.228429292	4.386294432	9.53903927	0.00201147	0.006957103
ENSRNOG00000019430	0.350025796	4.570451822	9.535658306	0.002015179	0.006968408
ENSRNOG00000047307	0.493978873	5.215553433	9.528615571	0.002022927	0.00699062
ENSRNOG00000011285	0.470221078	3.75453187	9.510624837	0.002042856	0.00705487
ENSRNOG00000007508	0.242440376	4.943699732	9.507951072	0.002045835	0.007063438
ENSRNOG00000049471	0.471485108	2.52660733	9.507597353	0.002046229	0.007063438
ENSRNOG00000043180	0.770950166	-0.499392133	9.503458962	0.002050849	0.007076301
ENSRNOG00000026336	0.396629537	2.117338707	9.486658557	0.002069714	0.007135174
ENSRNOG00000010417	0.533320685	3.028895144	9.479408124	0.00207791	0.007158752
ENSRNOG00000045686	0.959496515	-0.740534037	9.445288053	0.002116922	0.007282066
ENSRNOG00000012747	0.412535708	6.008839002	9.437639887	0.002125769	0.007306148
ENSRNOG00000000708	0.394511747	6.989100979	9.436495978	0.002127095	0.00730912
ENSRNOG00000012726	0.239830211	3.44714306	9.431371874	0.002133047	0.007326391
ENSRNOG00000011624	0.399621856	6.937702642	9.419139486	0.002147323	0.007370628
ENSRNOG00000042702	0.291771064	5.073150395	9.417352819	0.002149416	0.007376214
ENSRNOG00000014654	0.396507139	5.473111112	9.416725246	0.002150152	0.00737714
ENSRNOG00000021256	2.071893761	0.355752294	9.414914331	0.002152276	0.00738283
ENSRNOG00000017051	0.273947338	3.662715769	9.412798972	0.002154761	0.007389751
ENSRNOG00000022514	0.246377988	3.70671707	9.410162429	0.002157861	0.007398781
ENSRNOG00000020368	0.379495478	2.003677116	9.407797313	0.002160646	0.007406727
ENSRNOG00000050578	0.395279233	6.257044701	9.398888347	0.00217117	0.007436364
ENSRNOG00000001009	0.355447229	3.842283003	9.393150109	0.002177977	0.007458062
ENSRNOG00000000622	0.381183332	6.019196729	9.385101651	0.002187559	0.007489257
ENSRNOG00000047814	0.616903073	1.054777995	9.378878065	0.002194999	0.007511477
ENSRNOG00000012794	0.377191636	3.852825547	9.377350096	0.002196829	0.007514978
ENSRNOG00000046062	1.018162728	-0.459772879	9.373207237	0.002201799	0.007528243
ENSRNOG00000021899	0.270272097	4.552986783	9.371507828	0.002203842	0.007532853
ENSRNOG00000032942	0.390736575	4.000865797	9.370502347	0.002205051	0.007532853
ENSRNOG00000046204	0.42732239	2.66505311	9.370551035	0.002204992	0.007532853
ENSRNOG00000002943	0.225910006	3.720699069	9.368330087	0.002207666	0.007540159
ENSRNOG00000055981	0.37772958	2.024757679	9.362127193	0.002215149	0.007562456
ENSRNOG00000049349	0.268148829	6.525314952	9.356193511	0.002222332	0.007585343
ENSRNOG00000050603	1.18662263	-1.027367429	9.352115231	0.002227283	0.007600602
ENSRNOG00000006167	0.549302773	0.645347833	9.350536956	0.002229201	0.007605511
ENSRNOG00000020525	0.46942478	4.586238589	9.348892672	0.002231202	0.007610698
ENSRNOG00000019299	0.236193689	3.418275548	9.345430604	0.002235421	0.007619314
ENSRNOG00000003455	0.294890568	3.004644147	9.344428959	0.002236643	0.007621048
ENSRNOG00000014369	0.388801813	5.657328987	9.333421389	0.002250118	0.007665313
ENSRNOG00000015421	0.587513627	3.497656916	9.330062566	0.002254247	0.00767633
ENSRNOG00000036680	0.760201884	1.18462906	9.330001193	0.002254322	0.00767633
ENSRNOG00000000041	0.409361044	2.856319328	9.328600498	0.002256046	0.007680549
ENSRNOG00000010846	0.233654364	3.972974976	9.327956235	0.002256839	0.007681598
ENSRNOG00000019332	0.273056794	4.906423217	9.319851808	0.002266843	0.00771399
ENSRNOG00000028580	0.425149498	5.047469116	9.296485477	0.002295939	0.007799592
ENSRNOG00000024757	0.323288383	5.536719719	9.292546689	0.002300881	0.007813027
ENSRNOG00000039278	0.587894922	0.96274453	9.290036579	0.002304036	0.00781871
ENSRNOG00000001203	0.189385403	5.160044871	9.28731361	0.002307463	0.007828663
ENSRNOG00000017635	0.194366649	5.283094313	9.28574464	0.00230944	0.007833692
ENSRNOG00000008102	0.320166143	3.821773228	9.260866202	0.002341023	0.007928931
ENSRNOG00000000639	0.537912263	1.844713018	9.259730041	0.002342475	0.007932155
ENSRNOG00000021006	0.28748579	4.62956681	9.256630348	0.002346444	0.007938801
ENSRNOG00000049862	0.275729668	5.729030039	9.24953144	0.002355557	0.00796623
ENSRNOG00000024363	0.524219751	1.971130213	9.248787727	0.002356514	0.007967764

ENSRNOG00000028879	0.408452506	4.577484238	9.248253507	0.002357201	0.007968387
ENSRNOG00000007426	0.415134023	1.674138758	9.247035517	0.00235877	0.007969355
ENSRNOG00000042960	0.409339752	4.045680323	9.238116441	0.002370286	0.00800237
ENSRNOG00000011130	1.158839687	-0.416660375	9.233476256	0.002376301	0.008019255
ENSRNOG00000060550	0.260291912	7.132007064	9.231069222	0.002379427	0.008028093
ENSRNOG00000010952	0.240988068	5.654825209	9.220596559	0.002393076	0.008070705
ENSRNOG00000000700	0.376943013	4.498782795	9.206605691	0.002411434	0.008130887
ENSRNOG00000010161	0.322534618	5.875559252	9.204166676	0.002414649	0.008139994
ENSRNOG00000012960	0.305161946	3.226533293	9.198547266	0.002422073	0.008163282
ENSRNOG00000000561	0.574250869	2.984215716	9.182031437	0.002444026	0.008232016
ENSRNOG00000060486	0.266422359	2.904608547	9.174872299	0.002453605	0.008260765
ENSRNOG00000026770	0.313131788	4.207462236	9.173177643	0.002455878	0.008264903
ENSRNOG00000025075	0.72474347	2.443342434	9.167738652	0.002463187	0.008287638
ENSRNOG00000060538	0.228151253	5.503739893	9.167372708	0.00246368	0.008287638
ENSRNOG00000049219	0.217285677	7.994177634	9.165852634	0.002465727	0.008291001
ENSRNOG00000013657	0.37449531	7.739928734	9.149264681	0.00248818	0.008361173
ENSRNOG00000046984	0.217168912	6.414714981	9.135783042	0.002506582	0.008414079
ENSRNOG00000011952	0.214720376	5.474565416	9.133044071	0.002510337	0.008424899
ENSRNOG00000061091	0.353198636	2.483199559	9.12893431	0.002515982	0.00843848
ENSRNOG00000046094	0.897210533	-0.180146258	9.127584744	0.002517839	0.008442919
ENSRNOG00000011321	0.949515024	3.096192985	9.123014641	0.002524137	0.008460453
ENSRNOG00000024577	0.260773234	4.919224346	9.1176966	0.002531485	0.008483288
ENSRNOG00000006508	0.288627055	2.91982546	9.113581333	0.002537186	0.008500595
ENSRNOG00000010214	0.406733435	2.434996398	9.092372104	0.002566777	0.008587018
ENSRNOG00000023995	0.494493229	0.446833357	9.083983837	0.002578577	0.008624671
ENSRNOG00000028658	0.446905825	4.632878546	9.07423397	0.002592361	0.008668944
ENSRNOG00000010574	0.389431476	4.041725554	9.065917998	0.002604178	0.008702944
ENSRNOG00000020475	0.191446033	5.132494634	9.05942444	0.002613442	0.008731331
ENSRNOG00000007062	0.574800004	2.009555935	9.059191955	0.002613775	0.008731331
ENSRNOG00000045670	0.384764227	3.014168314	9.046563637	0.00263189	0.008789992
ENSRNOG00000036649	0.375277859	3.313341926	9.0430079	0.002637014	0.008803391
ENSRNOG00000015625	0.402563559	4.035481659	9.042161626	0.002638235	0.00880561
ENSRNOG00000012961	0.376383365	1.577285044	9.041128193	0.002639727	0.008806876
ENSRNOG00000015589	0.216933896	4.049325835	9.034667177	0.002649073	0.008836195
ENSRNOG00000046286	1.166973734	-1.039765475	9.024807498	0.0026634	0.008876504
ENSRNOG00000030151	0.272351402	4.047955779	9.02421444	0.002664264	0.008877516
ENSRNOG00000001034	0.259160142	6.167449378	9.023593239	0.00266517	0.008878665
ENSRNOG00000014915	0.241971772	4.463879397	9.017738843	0.002673719	0.008903401
ENSRNOG00000018183	0.283243777	8.041312965	9.015578269	0.002676882	0.008910184
ENSRNOG00000018440	0.204581074	5.741308175	9.009287125	0.002686112	0.008939027
ENSRNOG00000016128	0.253778424	4.020081998	8.995461256	0.00270651	0.008993677
ENSRNOG00000020489	0.665888615	4.6794014	8.992596745	0.002710755	0.009005896
ENSRNOG00000017172	0.324763507	7.097653378	8.985286664	0.002721621	0.009038202
ENSRNOG00000016619	0.412865713	4.840766555	8.975855775	0.002735704	0.009077356
ENSRNOG00000001123	0.218400267	4.429370956	8.971393664	0.002742393	0.009097644
ENSRNOG00000052307	0.343781124	4.550879908	8.959953418	0.002759619	0.009141383
ENSRNOG00000002864	0.374791311	6.688490341	8.951915154	0.002771788	0.009177854
ENSRNOG00000019215	0.896471272	-0.180533019	8.948453876	0.002777045	0.009191416
ENSRNOG00000022784	0.290793543	2.905673386	8.947535561	0.002778442	0.009194116
ENSRNOG00000015150	0.307295495	5.567460441	8.922947087	0.002816097	0.009308994
ENSRNOG00000019737	0.369278111	5.724404473	8.918509787	0.002822948	0.009329692
ENSRNOG00000023760	0.241860248	4.068524295	8.907997255	0.002839245	0.00937377
ENSRNOG00000005450	0.644517797	0.871682567	8.906858633	0.002841016	0.009377661
ENSRNOG00000059260	0.66500235	-0.105643536	8.899530341	0.00285244	0.009413409
ENSRNOG00000020379	0.218407654	4.390853669	8.898741161	0.002853673	0.009415516
ENSRNOG00000017001	0.249550782	3.902541868	8.898248817	0.002854443	0.009416042
ENSRNOG00000026928	0.809707495	-0.603669953	8.891799155	0.002864543	0.009442658
ENSRNOG00000012953	0.249345988	5.144397681	8.884013108	0.002876785	0.009479026
ENSRNOG00000006740	0.494187198	1.550144899	8.883805264	0.002877113	0.009479026
ENSRNOG00000043143	0.217785167	4.319908784	8.862739356	0.002910505	0.009581067
ENSRNOG00000020551	0.356152868	4.835136579	8.851710168	0.002928144	0.00963713
ENSRNOG00000021855	1.268395615	1.498551066	8.849589315	0.002931548	0.00964633
ENSRNOG00000014879	0.353280563	3.284318225	8.833035836	0.002958259	0.009724116
ENSRNOG00000057517	0.457677775	1.726846156	8.824234381	0.002972561	0.009769101
ENSRNOG00000046949	0.425944754	5.211880304	8.818712238	0.00298157	0.009794643
ENSRNOG00000053729	0.375417605	4.893257726	8.783207859	0.003040161	0.009970569
ENSRNOG00000051061	0.214100634	4.501611549	8.779577454	0.003046218	0.009982162
ENSRNOG00000046904	0.927682553	-0.694452543	8.773846541	0.003055803	0.010007361
ENSRNOG00000039017	0.359421406	2.509074841	8.774002104	0.003055543	0.010007361
ENSRNOG00000027230	0.307455483	5.183842759	8.767258101	0.003066861	0.010041498
ENSRNOG00000019534	0.204923557	7.571685867	8.765100182	0.003070492	0.010051307
ENSRNOG00000001287	0.647014939	0.600618629	8.764486441	0.003071526	0.010052612
ENSRNOG00000048580	0.420358285	2.351851068	8.761574357	0.003076434	0.010066595
ENSRNOG00000019222	0.275032792	5.409608469	8.759301835	0.003080269	0.010077063
ENSRNOG00000004033	0.286907333	4.933301674	8.757820835	0.003082772	0.010079599
ENSRNOG00000013949	0.234913036	5.989682209	8.752055022	0.003092534	0.010108831
ENSRNOG00000031612	0.381801983	4.814298516	8.741896662	0.003109809	0.010154957
ENSRNOG00000014424	0.798130811	2.016473463	8.737485246	0.003117341	0.010173121
ENSRNOG00000025843	0.606393153	0.464757857	8.73340627	0.003124323	0.010193803
ENSRNOG00000055204	0.983043543	0.675047699	8.729931953	0.003130282	0.010211142
ENSRNOG00000037204	0.356440316	3.515435465	8.729378335	0.003131232	0.010212139
ENSRNOG00000016416	0.342170241	4.952327051	8.727538928	0.003134393	0.010217194
ENSRNOG00000058308	0.45961959	4.221628375	8.724084244	0.003140338	0.010229193
ENSRNOG00000046275	0.948222844	0.060427652	8.717017239	0.003152534	0.010264697
ENSRNOG00000025108	0.353259845	2.688690435	8.716304087	0.003153767	0.010266602
ENSRNOG00000050106	0.255012614	3.027566416	8.711194134	0.00316262	0.010293303
ENSRNOG00000018214	0.280820764	3.623437848	8.701574924	0.003179352	0.010345636
ENSRNOG00000002821	0.22338834	6.593395719	8.690457959	0.003198802	0.010400377
ENSRNOG00000059450	0.672200862	-0.632813069	8.669567502	0.00323568	0.010507332
ENSRNOG00000003403	0.310354908	3.768077386	8.655039781	0.00326158	0.010584925
ENSRNOG00000011338	0.270291708	3.501507243	8.653582233	0.00326419	0.010591225
ENSRNOG00000001225	0.333183488	4.10014513	8.63562921	0.003296514	0.010691724
ENSRNOG00000051631	0.819841877	-1.112324518	8.633765767	0.003299887	0.010700474
ENSRNOG00000017786	0.40743282	2.755823585	8.616821188	0.003330726	0.010791629
ENSRNOG00000009039	0.227627097	5.91958438	8.613434035	0.003336926	0.010807298
ENSRNOG00000000457	0.598707161	1.389226781	8.612569836	0.003338509	0.010810216
ENSRNOG00000016562	0.228604001	4.50509858	8.611028234	0.003341336	0.010814946
ENSRNOG00000007882	0.308419368	6.017237717	8.604963642	0.003352481	0.010846583
ENSRNOG00000060825	0.421810428	5.358150855	8.60167598	0.003358538	0.010863961
ENSRNOG00000018289	0.2435586	6.497435806	8.600851265	0.003360059	0.010866662

ENSRNOG00000003226	0.214173718	8.160589436	8.596203314	0.003368646	0.010887759
ENSRNOG00000003469	0.605249878	5.593199136	8.588719478	0.003382518	0.010929956
ENSRNOG00000011361	0.221012368	4.131194442	8.58151448	0.003395928	0.010962513
ENSRNOG00000033152	0.46656211	0.691437956	8.57537181	0.003407404	0.010995074
ENSRNOG00000005616	0.222464767	5.077105397	8.572734879	0.003412342	0.011006524
ENSRNOG00000059880	0.258194601	5.500133265	8.571753302	0.003414182	0.011010216
ENSRNOG00000001431	0.76216728	1.285393514	8.560388406	0.003435561	0.011070142
ENSRNOG00000010259	1.048382767	-0.782126616	8.548153804	0.003458728	0.011140257
ENSRNOG00000000697	0.258348093	6.332030263	8.547188686	0.003460563	0.011141632
ENSRNOG00000004726	0.291948319	4.608901337	8.547189924	0.00346056	0.011141632
ENSRNOG00000001443	0.53022652	5.04118409	8.545009643	0.003464708	0.01115271
ENSRNOG00000010539	0.276345467	3.11066208	8.533138787	0.003487378	0.011223402
ENSRNOG00000029264	0.526505543	3.138720386	8.528888644	0.003495531	0.01124507
ENSRNOG00000037931	0.984696077	-0.789730407	8.526764984	0.003499612	0.011255912
ENSRNOG00000022767	0.578834349	4.484315423	8.524219896	0.003504509	0.011269375
ENSRNOG00000008642	0.280741743	4.62739366	8.5181147	0.003516286	0.011304948
ENSRNOG00000028649	0.337177069	3.112796744	8.516218647	0.003519951	0.011314435
ENSRNOG00000061007	0.68207653	-0.413828494	8.512371232	0.003527401	0.01133378
ENSRNOG00000005132	0.423588589	1.705880776	8.502460044	0.003546666	0.011393369
ENSRNOG00000023509	0.346560173	6.483293391	8.50107813	0.003549361	0.011399713
ENSRNOG00000054560	0.601172706	3.765028944	8.490896879	0.003569278	0.011454969
ENSRNOG00000028415	0.690010648	0.27281697	8.48584253	0.003579208	0.011483926
ENSRNOG00000029941	0.312781446	8.043373502	8.480063038	0.003590597	0.0115158
ENSRNOG00000030034	0.474179463	1.801643853	8.475250179	0.003600109	0.01153696
ENSRNOG00000009131	0.633305366	0.443067525	8.473672203	0.003603233	0.0115423
ENSRNOG00000028198	0.255241235	4.757430197	8.467480483	0.003615519	0.011574631
ENSRNOG00000048622	0.297799019	5.652924594	8.458069194	0.003634275	0.011632323
ENSRNOG00000000641	0.324343003	2.438044158	8.453758102	0.003642899	0.011650508
ENSRNOG00000008142	0.212994118	4.612573376	8.447662448	0.003655129	0.011677829
ENSRNOG00000009351	0.226207194	5.245204391	8.433766511	0.003683166	0.011755545
ENSRNOG00000021659	0.940956432	-0.988635638	8.433142088	0.003684431	0.011757212
ENSRNOG00000039315	0.440970529	1.565477671	8.42497578	0.003701016	0.011798245
ENSRNOG00000016225	0.321789768	2.992854388	8.410839136	0.003729905	0.01188077
ENSRNOG00000009206	1.151910647	1.19121478	8.404947454	0.003742012	0.011912147
ENSRNOG00000003953	0.241630329	6.048487274	8.387289387	0.00377854	0.012021177
ENSRNOG00000009611	0.253832252	4.02407379	8.386713988	0.003779736	0.012022568
ENSRNOG00000043503	0.191155767	5.403113851	8.385544239	0.003782169	0.012027891
ENSRNOG00000020412	0.176616262	7.075137145	8.38328187	0.00378688	0.012040454
ENSRNOG00000012864	0.237530275	4.161132796	8.376474749	0.00380109	0.012083208
ENSRNOG00000055053	0.607871021	1.858652368	8.369153264	0.003816434	0.012127115
ENSRNOG00000019000	0.17603609	5.897644895	8.364309782	0.003826619	0.012152164
ENSRNOG00000007280	0.179526395	6.501131344	8.358007047	0.003839915	0.012184612
ENSRNOG00000011552	0.278477864	4.271350323	8.35493472	0.003846413	0.012202785
ENSRNOG00000007613	0.261308608	3.720094255	8.351827391	0.003852996	0.012221223
ENSRNOG00000048907	0.61464077	0.360252257	8.338889125	0.003880532	0.012303632
ENSRNOG00000001494	0.277375429	6.799105405	8.334674134	0.003889545	0.012324807
ENSRNOG00000001101	0.172949132	5.526892291	8.324437216	0.003911525	0.012389497
ENSRNOG00000017737	0.417740928	7.693124068	8.320738919	0.003919497	0.012409782
ENSRNOG00000019623	0.356959101	1.818575363	8.311544704	0.003939387	0.012470264
ENSRNOG00000020506	0.529192366	2.447509049	8.311067152	0.003940423	0.01247105
ENSRNOG00000061231	0.3955404	5.081967205	8.309923974	0.003942904	0.012473915
ENSRNOG00000022043	0.206091812	5.126257476	8.308724652	0.003945509	0.012479661
ENSRNOG00000006800	0.404038299	5.429523247	8.303083771	0.003957782	0.012515981
ENSRNOG00000015420	0.251921555	9.075172094	8.301113195	0.003962079	0.012527067
ENSRNOG00000018379	0.420255758	1.704665387	8.29682427	0.003971447	0.012552716
ENSRNOG00000012428	0.441825819	1.458857287	8.289442693	0.003987622	0.012597768
ENSRNOG00000007249	0.399441317	5.383045154	8.28821689	0.003990315	0.012601247
ENSRNOG00000014997	0.220185846	5.730826971	8.285714739	0.003995817	0.012616106
ENSRNOG00000014703	0.69471789	1.503151849	8.276920259	0.004015218	0.01266978
ENSRNOG00000001192	0.364557289	4.9034839	8.257536811	0.004058316	0.012800671
ENSRNOG00000022116	0.295574293	5.783673519	8.227309182	0.004126467	0.012997508
ENSRNOG00000052880	1.137027626	1.831686563	8.226207422	0.004128973	0.013000229
ENSRNOG00000019584	0.981901819	3.644163596	8.220471919	0.004142043	0.013033606
ENSRNOG00000007982	0.283359075	5.581096035	8.218057948	0.004147556	0.013044735
ENSRNOG00000048365	0.519764287	1.502293803	8.215231043	0.004154023	0.013058327
ENSRNOG00000042374	0.632728467	-0.016801732	8.183732022	0.004226771	0.013260689
ENSRNOG00000053991	0.256323395	3.068739849	8.183875046	0.004226438	0.013260689
ENSRNOG00000048050	0.327499154	2.289604941	8.178958867	0.004237908	0.01329036
ENSRNOG00000020361	0.208143399	5.624729233	8.177207568	0.004242001	0.013300564
ENSRNOG00000050669	0.649742623	0.908766005	8.174661392	0.00424796	0.013313974
ENSRNOG00000008517	0.582109462	4.139105745	8.171987148	0.004254227	0.013330979
ENSRNOG00000007500	0.290853097	3.686023154	8.171613561	0.004255104	0.013331087
ENSRNOG00000001854	0.248519389	4.268940499	8.168102261	0.004263349	0.013354277
ENSRNOG00000026111	0.487051215	5.550589795	8.165042891	0.004270546	0.013374176
ENSRNOG00000018358	0.415655368	2.528698434	8.162427736	0.004276709	0.013388178
ENSRNOG00000021886	0.28703528	4.038037975	8.155819795	0.004292319	0.013426429
ENSRNOG00000047880	0.646030279	0.429656234	8.153669279	0.004297412	0.013439705
ENSRNOG00000032944	0.859082902	0.349902176	8.147243029	0.004312668	0.013484751
ENSRNOG00000000186	0.381732959	5.463903397	8.142373755	0.004324264	0.01351834
ENSRNOG00000023603	0.38860521	3.58780164	8.135750277	0.004340088	0.013562455
ENSRNOG00000004888	0.282794519	4.249016151	8.118324064	0.004382005	0.01368264
ENSRNOG00000058126	0.707019182	-0.927855909	8.116798606	0.004385694	0.013686079
ENSRNOG00000031135	0.246791738	7.786921552	8.116796242	0.0043857	0.013686079
ENSRNOG00000001316	0.195544169	7.460370794	8.114210952	0.004391959	0.013702911
ENSRNOG00000000999	0.259004796	5.553368747	8.10180464	0.004422121	0.013791582
ENSRNOG00000002413	0.398650535	3.159311412	8.093645178	0.004442073	0.01385108
ENSRNOG00000004283	0.282700274	7.333223365	8.091651099	0.004446963	0.013860869
ENSRNOG00000013287	0.263293356	5.99618549	8.089066112	0.00445331	0.013877921
ENSRNOG00000011523	0.213162916	5.969255271	8.085600016	0.004461836	0.013901752
ENSRNOG00000002907	0.3070058	6.455153529	8.072936107	0.004493125	0.013993735
ENSRNOG00000016781	0.202133513	5.908517562	8.067495787	0.004506636	0.014024778
ENSRNOG00000037087	0.388664685	2.420746155	8.061716895	0.004521032	0.014066815
ENSRNOG00000005984	0.578757125	1.297657689	8.060314676	0.004524532	0.014073706
ENSRNOG00000054063	0.338906019	4.720653242	8.060117649	0.004525025	0.014073706
ENSRNOG00000021128	0.340491842	4.426766687	8.052986122	0.004542871	0.014120892
ENSRNOG00000000299	0.340925191	4.042167403	8.053334266	0.004541998	0.014120892
ENSRNOG00000018293	0.390299788	5.944718046	8.049125484	0.004552563	0.014148239
ENSRNOG00000001607	0.391556434	2.33217366	8.042592698	0.00456901	0.01419378
ENSRNOG00000001216	0.225855719	4.209570912	8.039624402	0.004576503	0.014211481
ENSRNOG00000008000	0.358468754	7.057104248	8.037698282	0.004581371	0.014222809

ENSRNOG00000028211	0.191908186	4.814456859	8.037444256	0.004582014	0.014222809
ENSRNOG00000023668	0.274500853	5.598496907	8.027612621	0.004606952	0.014292023
ENSRNOG00000020618	0.263321409	7.889204696	8.014580951	0.004640221	0.014383958
ENSRNOG00000012866	0.373992888	1.117750653	8.013401881	0.004643243	0.014387693
ENSRNOG00000001797	0.224723546	3.859688563	8.008769565	0.004655136	0.0144189
ENSRNOG00000059479	0.974797162	5.285376061	7.990769753	0.004701642	0.014543036
ENSRNOG00000012946	0.356183458	3.395206773	7.983599729	0.004720299	0.014595043
ENSRNOG00000000283	0.291976099	3.404502272	7.982474975	0.004723233	0.014601262
ENSRNOG00000002488	0.436037724	3.968205019	7.972252011	0.004749981	0.01466391
ENSRNOG00000059784	0.713029499	-0.064718964	7.967080385	0.004763571	0.014700133
ENSRNOG00000041170	0.724554921	-0.89572493	7.961403693	0.004778534	0.014740563
ENSRNOG00000020083	0.385223373	2.498222837	7.958086668	0.004787299	0.01476185
ENSRNOG00000001752	0.373144855	1.593307369	7.955179271	0.004794996	0.014782704
ENSRNOG00000042929	0.433733265	4.240605706	7.953606168	0.004799165	0.014792678
ENSRNOG00000060438	0.499437607	1.43716822	7.952642516	0.004801721	0.014797676
ENSRNOG00000043364	0.51702566	2.626457822	7.948114237	0.004813751	0.014823207
ENSRNOG00000013256	0.218023714	5.51795284	7.945285671	0.004821281	0.014840622
ENSRNOG00000000129	0.489067328	5.409229488	7.94410723	0.004824421	0.014844839
ENSRNOG00000004159	0.313615858	5.963220247	7.929382195	0.00486384	0.014941252
ENSRNOG00000002353	0.189243736	6.157682715	7.924526314	0.004876911	0.014965356
ENSRNOG00000003052	0.279240201	3.076932185	7.924521037	0.004876925	0.014965356
ENSRNOG00000014243	0.453223749	2.909281237	7.920126245	0.004888786	0.014995939
ENSRNOG00000052207	0.764956889	2.023695853	7.906981263	0.004924438	0.015093602
ENSRNOG00000053756	0.764956889	2.023695853	7.906981263	0.004924438	0.015093602
ENSRNOG00000001293	0.19622378	5.35536267	7.88676695	0.00497978	0.01525437
ENSRNOG00000051948	0.246234927	5.835677338	7.871354383	0.005022402	0.015373036
ENSRNOG00000037080	0.635105712	-0.203738966	7.865293961	0.005039263	0.015418685
ENSRNOG00000002341	0.383302656	1.594851937	7.862530303	0.005046971	0.015436304
ENSRNOG00000017871	0.210221595	6.485851612	7.860023889	0.005053972	0.015451747
ENSRNOG00000024071	0.604646323	0.771416007	7.859308801	0.005055972	0.015454876
ENSRNOG00000052887	0.256271992	7.724177846	7.84633721	0.005092378	0.015563155
ENSRNOG00000019080	0.315327386	3.186249401	7.837992763	0.005115938	0.015632143
ENSRNOG00000011381	0.236983836	7.896546039	7.827571494	0.005145519	0.015713432
ENSRNOG00000012568	0.196921335	7.164009672	7.826402079	0.005148849	0.01572057
ENSRNOG00000050258	0.323749236	4.214425085	7.824448702	0.005154417	0.015734535
ENSRNOG00000021814	0.944979813	0.05104425	7.823815959	0.005156221	0.015737011
ENSRNOG00000012122	0.337889603	4.305752138	7.815908232	0.005178831	0.015798609
ENSRNOG00000002276	0.329989923	2.546254306	7.815710693	0.005179398	0.015798609
ENSRNOG00000012543	0.513039539	1.051942417	7.804433995	0.005211819	0.015885263
ENSRNOG00000027742	0.361215398	2.284995837	7.799752979	0.005225338	0.015923402
ENSRNOG00000018526	0.348823651	8.806420428	7.799162119	0.005227047	0.015925545
ENSRNOG00000005227	0.438556627	2.043855807	7.789483987	0.00525512	0.016001841
ENSRNOG00000011329	0.261407577	8.874050635	7.778086957	0.005288377	0.016095347
ENSRNOG00000011311	0.171806914	5.977948231	7.776600303	0.005292731	0.016097791
ENSRNOG00000018087	0.437975061	5.441014524	7.775980304	0.005294548	0.016100224
ENSRNOG00000043150	0.223048969	5.54849261	7.769354587	0.005314003	0.016140788
ENSRNOG00000005218	0.221190133	5.982662206	7.76669822	0.005321824	0.016161442
ENSRNOG00000008054	0.609832947	2.052617569	7.764803933	0.005327408	0.016175298
ENSRNOG00000018412	0.245949456	4.557264628	7.762223921	0.005335023	0.016192209
ENSRNOG00000014357	0.415748309	1.061953436	7.754159366	0.005358898	0.016261554
ENSRNOG00000008738	0.624629907	5.223672642	7.75006311	0.005371066	0.016285995
ENSRNOG00000004556	0.165117777	6.117541369	7.747698913	0.005378102	0.016304207
ENSRNOG00000000847	0.242382137	5.525463278	7.744501256	0.005387634	0.016329976
ENSRNOG00000021096	0.279451227	3.184286974	7.724251325	0.005448395	0.016479443
ENSRNOG00000011881	0.292661095	3.835740051	7.722318512	0.005454231	0.016493944
ENSRNOG00000033343	0.626969643	-0.212901223	7.721946278	0.005455355	0.016494195
ENSRNOG00000051824	0.593057116	2.612458905	7.72008681	0.005460977	0.016508039
ENSRNOG00000004742	0.202854078	6.20814934	7.713253763	0.005481686	0.016567477
ENSRNOG00000021026	0.305683382	4.663908614	7.712775222	0.005483139	0.016568707
ENSRNOG00000027574	0.76941935	-0.996286918	7.708808374	0.005495201	0.016601988
ENSRNOG00000004932	0.253278376	4.252543281	7.707283074	0.005499847	0.016609684
ENSRNOG00000025481	0.213581844	7.076506205	7.7059121	0.005504025	0.016612798
ENSRNOG00000019374	0.36363525	4.770915414	7.706245819	0.005503008	0.016612798
ENSRNOG00000009000	0.213239745	5.331207623	7.705555032	0.005505114	0.016612918
ENSRNOG00000010257	0.349467838	4.224664237	7.70289111	0.005513245	0.016627946
ENSRNOG00000007234	0.219322716	6.930471646	7.696034384	0.005534228	0.016688054
ENSRNOG00000025936	0.164989116	7.233860122	7.6945076	0.005538912	0.016691619
ENSRNOG00000046195	0.747820771	0.181937139	7.689005479	0.005555823	0.016734051
ENSRNOG00000027282	0.216184727	4.660493026	7.686541195	0.005563415	0.01675373
ENSRNOG00000012534	0.379516522	3.933590899	7.683421159	0.005573042	0.016776338
ENSRNOG00000007290	0.263140953	10.6363735	7.658913853	0.00564925	0.0169767
ENSRNOG00000015121	0.22205503	5.227752232	7.644968781	0.005693088	0.017098702
ENSRNOG00000020182	0.227735421	4.405881298	7.643216142	0.005698622	0.017112077
ENSRNOG00000001435	0.541411797	0.164533749	7.637907361	0.005715419	0.017156007
ENSRNOG00000016778	0.251642121	6.216358559	7.636448284	0.005720044	0.017164808
ENSRNOG00000018878	0.472220041	4.912692139	7.634043202	0.005727676	0.017183028
ENSRNOG00000039265	0.376894431	2.613961467	7.633120698	0.005730606	0.017188562
ENSRNOG00000021130	0.366242255	3.902426865	7.625461874	0.005754993	0.017258439
ENSRNOG00000018576	0.418402432	2.333614449	7.620767546	0.005769993	0.017296869
ENSRNOG00000018671	0.243954208	5.347803729	7.616693708	0.005783042	0.017326146
ENSRNOG00000054625	0.346064049	2.369516443	7.607834219	0.005811524	0.017404895
ENSRNOG00000046424	0.223072379	3.760563224	7.604391232	0.005822632	0.017428271
ENSRNOG00000029958	0.468446199	0.098452057	7.604752695	0.005821465	0.017428271
ENSRNOG00000005332	0.306705664	7.032613176	7.601863635	0.0058308	0.017449421
ENSRNOG00000010077	0.256350209	5.862959606	7.596986551	0.005846594	0.01749338
ENSRNOG00000001686	0.313979213	2.710996576	7.588876223	0.005872955	0.017555667
ENSRNOG00000000938	0.212880729	7.003499152	7.57758611	0.005909853	0.017645976
ENSRNOG00000060154	0.193868518	6.473476983	7.574225758	0.005920881	0.01767557
ENSRNOG00000026941	0.235709497	6.692865286	7.563924374	0.005954818	0.017763488
ENSRNOG00000029165	0.757693289	4.880951558	7.558082327	0.005974153	0.017814451
ENSRNOG00000048231	0.954533931	-0.966988869	7.554457834	0.005986181	0.017840238
ENSRNOG00000019361	0.363646939	4.354915854	7.551890726	0.005994714	0.017862308
ENSRNOG00000031171	0.241109973	4.138858417	7.546280039	0.006013409	0.017914641
ENSRNOG00000001283	0.172464591	4.771365406	7.536440017	0.006046339	0.017999198
ENSRNOG00000016122	0.238510614	6.09846036	7.533603726	0.006055865	0.018024167
ENSRNOG00000039658	0.215443599	3.826263661	7.526884439	0.006078494	0.018081516
ENSRNOG00000017918	0.606717682	2.676780429	7.523750224	0.006089078	0.018106003
ENSRNOG00000019253	0.451832376	4.986080742	7.523006449	0.006091593	0.018110079
ENSRNOG00000001747	0.490065157	0.427967382	7.522619771	0.006092901	0.018110567
ENSRNOG00000028650	0.806685188	5.859185083	7.515030347	0.006118625	0.018183618

ENSRNOG00000012156	0.244532031	3.822329474	7.513051801	0.00612535	0.018196772
ENSRNOG00000007591	0.602808516	0.390020609	7.507234386	0.006145166	0.018248791
ENSRNOG00000011716	0.409414244	1.83999734	7.502721845	0.006160582	0.018280857
ENSRNOG00000061946	0.455866782	4.510909706	7.499094008	0.006173004	0.018314286
ENSRNOG00000010953	0.185807773	4.492991815	7.497845409	0.006177285	0.018323555
ENSRNOG00000015583	0.295710995	4.685403707	7.494768536	0.006187848	0.01835145
ENSRNOG00000009726	0.513368216	4.80925121	7.492821757	0.006194541	0.018367859
ENSRNOG00000059017	0.245004509	5.863354377	7.490136701	0.006203785	0.01838838
ENSRNOG00000016419	0.243963967	4.335214485	7.481952455	0.006232046	0.018452401
ENSRNOG00000015502	0.847040765	-0.940375215	7.472957015	0.00626326	0.01852998
ENSRNOG00000017960	0.299682857	4.573495726	7.471621327	0.006267908	0.018540268
ENSRNOG00000060988	0.792103329	4.81964221	7.471187487	0.006269419	0.018541273
ENSRNOG00000032771	0.721601523	-0.611693123	7.456842664	0.006319575	0.018675654
ENSRNOG00000008030	0.240583276	5.124023634	7.440181723	0.006378345	0.018838783
ENSRNOG00000006975	0.269122194	6.467485152	7.422797102	0.006440262	0.019003934
ENSRNOG00000019236	0.241810827	6.755351874	7.409251889	0.00648893	0.019126158
ENSRNOG00000049121	0.211139988	3.482049416	7.399865117	0.006522877	0.019222637
ENSRNOG00000004132	0.189887	6.941880321	7.395796003	0.006537649	0.019248258
ENSRNOG00000008053	0.282935661	4.478016321	7.388403273	0.006564574	0.019320346
ENSRNOG00000021109	0.895517657	1.501630728	7.378013699	0.006602605	0.019428666
ENSRNOG00000001325	0.659946312	2.644656381	7.368838966	0.006636377	0.01951716
ENSRNOG00000050968	0.527286289	1.142222637	7.363681213	0.006655439	0.019562322
ENSRNOG00000061779	0.303713513	4.604174381	7.357758073	0.0066774	0.019619586
ENSRNOG00000050181	0.881383721	-0.986857035	7.350808186	0.006703262	0.019691919
ENSRNOG00000050573	0.560414912	0.035384251	7.326737486	0.006793626	0.019935187
ENSRNOG00000017198	0.572301022	1.761392273	7.318766722	0.006823822	0.020012407
ENSRNOG00000028036	0.550408976	0.445748335	7.316837718	0.006831151	0.020023142
ENSRNOG00000019419	0.264888405	3.147504943	7.316020234	0.006834259	0.020028439
ENSRNOG00000005130	0.205659487	7.368003173	7.312964036	0.006845892	0.020058817
ENSRNOG00000014625	0.368478955	5.918939659	7.30929077	0.0068599	0.02009427
ENSRNOG00000024077	0.400409784	0.494913585	7.306307824	0.006871297	0.020122085
ENSRNOG00000003749	0.2901954	3.628026627	7.30438615	0.006878649	0.020138139
ENSRNOG00000019351	0.897714888	-0.999660966	7.292318821	0.006925004	0.020268116
ENSRNOG00000022039	0.235908283	4.14627054	7.287723327	0.00694274	0.020305015
ENSRNOG00000020541	0.27917062	4.086055522	7.285806088	0.006950154	0.02031919
ENSRNOG00000007837	0.575687616	3.803100066	7.285355714	0.006951896	0.020320533
ENSRNOG00000005849	0.162656942	5.136175122	7.2795718	0.006974316	0.020378542
ENSRNOG00000021773	0.20385915	4.529192827	7.279718897	0.006973745	0.020378542
ENSRNOG00000046973	0.605204332	-0.568163872	7.274276652	0.006994906	0.020431163
ENSRNOG00000022321	0.702232333	-0.6437295	7.270603283	0.007009226	0.020469214
ENSRNOG00000008163	0.21778012	7.029099609	7.269563625	0.007013284	0.020477289
ENSRNOG00000060617	0.443774683	3.030176648	7.265685964	0.007028442	0.020517764
ENSRNOG00000018039	0.545911749	7.32595299	7.265243858	0.007030172	0.020519032
ENSRNOG00000027286	0.377339879	2.11855246	7.241177624	0.007125023	0.020749976
ENSRNOG00000021945	0.527460589	3.400228315	7.240431572	0.007127984	0.020753704
ENSRNOG00000018745	0.22727875	5.570754114	7.236208902	0.007144769	0.020792182
ENSRNOG00000024567	0.377487332	3.01714711	7.227424048	0.007179816	0.020871159
ENSRNOG00000059456	0.292629477	4.081295281	7.226042333	0.007185344	0.020883395
ENSRNOG00000014340	0.173701703	4.745179804	7.212807338	0.007238519	0.021010945
ENSRNOG00000025643	0.390127524	1.417812742	7.212921868	0.007238057	0.021010945
ENSRNOG00000036681	0.485560615	1.260317487	7.205565726	0.007267784	0.021092025
ENSRNOG00000014163	0.395647843	3.663127256	7.196879836	0.007303045	0.021185076
ENSRNOG00000001227	0.287871013	7.215922816	7.196679382	0.007303861	0.021185076
ENSRNOG00000050378	0.369101471	1.376177139	7.188805716	0.007335979	0.021266549
ENSRNOG00000007377	0.50327927	4.338533058	7.184108925	0.007355207	0.021310586
ENSRNOG00000011124	0.267696864	3.550951401	7.17463337	0.007394156	0.02140385
ENSRNOG00000047339	0.280474386	8.540470968	7.174658984	0.00739405	0.02140385
ENSRNOG00000015594	0.185919843	4.359649715	7.168877395	0.007417918	0.021460865
ENSRNOG00000002883	0.234946247	3.586135851	7.167143164	0.007425093	0.021477698
ENSRNOG00000036700	0.494916694	-0.018269532	7.161389286	0.007448948	0.021530968
ENSRNOG00000013851	0.303977038	3.735285432	7.15877349	0.007459819	0.02155452
ENSRNOG00000003785	0.790300308	-0.650519045	7.156429971	0.007469572	0.021574827
ENSRNOG00000018854	0.592644793	-0.347004303	7.153741572	0.007480776	0.021603249
ENSRNOG00000017086	0.415972267	1.603043979	7.152528711	0.007485837	0.021613921
ENSRNOG00000012237	0.263668895	5.530093767	7.134591078	0.007561088	0.021815283
ENSRNOG00000000168	0.231889135	7.570084308	7.1289124	0.007585072	0.021880494
ENSRNOG00000008475	0.233102036	3.700625763	7.123408136	0.007608394	0.021939775
ENSRNOG00000002254	0.195620057	6.605922647	7.122419721	0.007612589	0.021942913
ENSRNOG00000009521	0.557120735	-0.565284795	7.122598805	0.007611829	0.021942913
ENSRNOG00000001817	0.178150899	5.53932579	7.116261038	0.007638785	0.022011379
ENSRNOG00000019283	0.566463823	0.42660042	7.111634306	0.007658526	0.022056466
ENSRNOG00000021787	0.359857209	1.581592168	7.109729076	0.00766667	0.022075659
ENSRNOG00000012192	0.176940821	6.205157664	7.107379986	0.007676723	0.022084526
ENSRNOG00000020695	0.732465987	-0.132216242	7.106833225	0.007679065	0.022086783
ENSRNOG00000018838	0.266831958	3.728899389	7.106545617	0.007680297	0.022086783
ENSRNOG00000049397	0.744266087	-0.826669435	7.1010632	0.007703824	0.022150418
ENSRNOG00000028622	0.484068615	1.301569222	7.100170644	0.007707661	0.022153405
ENSRNOG00000018927	0.634374011	1.605842549	7.094005643	0.007734219	0.022217639
ENSRNOG00000006345	0.244547337	3.496959268	7.090185987	0.00775072	0.022256965
ENSRNOG00000017146	0.337015748	2.352017274	7.088685505	0.007757212	0.022271569
ENSRNOG00000006505	0.31611266	3.788668305	7.082658567	0.007783345	0.022338496
ENSRNOG00000010185	0.290792939	5.068434999	7.077714902	0.007804848	0.022388036
ENSRNOG00000014254	0.178896816	5.461531917	7.076198999	0.007811453	0.022402926
ENSRNOG00000007147	0.480951935	6.583798004	7.074969644	0.007816815	0.022414242
ENSRNOG00000033916	0.497970139	4.830809141	7.071054408	0.007833914	0.022447013
ENSRNOG00000045913	0.654121289	3.173852802	7.070096824	0.007838102	0.02245495
ENSRNOG00000015641	0.362178066	1.772992681	7.06602146	0.007855951	0.022502013
ENSRNOG00000001407	0.414567746	0.661257778	7.060033306	0.007882253	0.022573268
ENSRNOG00000009953	0.267821718	4.564925991	7.055511857	0.007902173	0.02262213
ENSRNOG00000009323	0.388219239	4.164531949	7.055730288	0.007901209	0.02262213
ENSRNOG00000000047	0.313280514	4.658570212	7.050162347	0.007925807	0.022685688
ENSRNOG00000008378	0.41954034	3.501528745	7.048923973	0.007931288	0.022697274
ENSRNOG00000026748	0.277219347	4.596228878	7.037565848	0.007981744	0.022821042
ENSRNOG00000037302	0.538986982	-0.343244255	7.031809384	0.008007441	0.02289038
ENSRNOG00000048172	0.685268072	3.879267822	7.03066809	0.008012546	0.022900839
ENSRNOG00000057451	0.423133923	0.763637758	7.025083386	0.008037573	0.022955795
ENSRNOG00000012329	0.315678151	7.165854924	7.017738115	0.008070612	0.023045999
ENSRNOG00000061176	0.304978268	7.527637692	7.006044222	0.008123498	0.023176119
ENSRNOG00000055213	0.326901964	4.356097848	6.991715823	0.008188782	0.023347673
ENSRNOG00000017037	0.333209262	2.23021621	6.97884121	0.0082479	0.023501393

ENSRNOG00000020443	0.800464316	-1.178343325	6.970591187	0.008286012	0.023588765
ENSRNOG00000002072	0.379796942	0.688419528	6.966079583	0.00830693	0.023639814
ENSRNOG00000025695	0.187711021	7.025082383	6.963313003	0.008319784	0.023672139
ENSRNOG00000002941	0.258815895	6.486620223	6.962058645	0.008325618	0.023684485
ENSRNOG00000014082	0.528396218	0.265089418	6.961471692	0.00832835	0.023688
ENSRNOG00000006831	0.443194962	3.139004215	6.958811108	0.008340744	0.023706217
ENSRNOG00000008217	0.759404276	-1.018836514	6.954664223	0.008360099	0.023752701
ENSRNOG00000030633	0.353779619	4.037680557	6.952330072	0.008371013	0.023779445
ENSRNOG00000002768	1.10978042	-0.788226562	6.946933652	0.008396303	0.023847006
ENSRNOG00000017021	0.32312945	4.325167778	6.946459527	0.008398529	0.02384905
ENSRNOG00000013718	0.201002804	8.088519546	6.944375534	0.008408318	0.023864401
ENSRNOG00000015590	0.878240028	0.775731045	6.94434628	0.008408456	0.023864401
ENSRNOG00000020088	0.269622717	4.809461348	6.941756639	0.008420638	0.02389041
ENSRNOG00000015986	0.420335939	5.390897819	6.937555921	0.008440436	0.023938003
ENSRNOG00000028274	0.47712171	6.716045067	6.930052754	0.008475919	0.024017126
ENSRNOG00000001229	0.667624887	3.72969219	6.925417634	0.008497915	0.024070837
ENSRNOG00000046990	0.792001733	-0.769077006	6.913296453	0.008555711	0.024217221
ENSRNOG00000016631	0.293763422	3.087142761	6.907981059	0.008581183	0.024271964
ENSRNOG00000020882	0.298371265	2.977663465	6.904946836	0.008595758	0.024304506
ENSRNOG00000019433	0.188249758	8.375118918	6.900112444	0.008619033	0.02435292
ENSRNOG00000001297	0.448528549	5.499147462	6.883824642	0.008697924	0.024562678
ENSRNOG00000048545	0.73472995	-0.503237667	6.875726243	0.008737425	0.024656635
ENSRNOG00000016255	0.73304647	6.72237075	6.874641603	0.008742729	0.024667207
ENSRNOG00000005185	0.867115242	2.798104281	6.87026076	0.008764186	0.024714537
ENSRNOG00000017579	0.285899276	3.212197915	6.857149919	0.008828726	0.024869961
ENSRNOG00000054497	0.249691493	3.501609772	6.853014584	0.008849184	0.024918722
ENSRNOG00000018441	0.236251952	5.916605587	6.852112974	0.00885365	0.024922564
ENSRNOG00000012934	0.20305029	6.154623123	6.846854402	0.008879748	0.024982575
ENSRNOG00000016031	0.149649981	6.564802842	6.843709841	0.008895392	0.025022141
ENSRNOG00000017884	0.609362034	-0.498054639	6.840009769	0.008913835	0.025065116
ENSRNOG00000021108	0.433252369	0.777553255	6.835324301	0.008937247	0.025126485
ENSRNOG00000019649	0.202136297	5.992385293	6.829705869	0.008965403	0.025192225
ENSRNOG00000004279	0.196579425	4.251264666	6.825816427	0.008984947	0.025242665
ENSRNOG00000061450	0.373485762	2.962375409	6.81843464	0.009022161	0.025338224
ENSRNOG00000016392	0.238081265	2.585504353	6.817095343	0.00902893	0.025352736
ENSRNOG00000019802	0.325640521	3.721206578	6.808943103	0.009070243	0.025446179
ENSRNOG00000004094	0.353680872	2.18923324	6.806771396	0.009081282	0.025468121
ENSRNOG00000010409	0.215677759	6.287724587	6.805555459	0.009087468	0.025476446
ENSRNOG00000014362	0.324727392	2.40306405	6.801268403	0.009109314	0.02552865
ENSRNOG00000027833	0.248229889	3.968917001	6.789682639	0.009168621	0.025658526
ENSRNOG00000011747	0.274675587	3.571128453	6.788881969	0.009172734	0.025660966
ENSRNOG00000033740	0.436063581	1.653521734	6.783412638	0.009200881	0.025726074
ENSRNOG00000039339	0.59224483	-0.589601503	6.782760292	0.009204244	0.025726391
ENSRNOG00000056017	0.291813952	3.408871292	6.783037065	0.009202817	0.025726391
ENSRNOG00000019723	0.419882474	0.814920046	6.774159843	0.009248702	0.025832412
ENSRNOG00000008898	0.236891353	3.004335922	6.77200338	0.009259883	0.025854522
ENSRNOG00000006217	0.350764607	4.766589953	6.77039571	0.009268228	0.025873259
ENSRNOG00000004670	0.272997529	3.523681976	6.76800831	0.009280635	0.025903327
ENSRNOG00000016552	0.266791137	7.734315305	6.763650698	0.009303324	0.025948359
ENSRNOG00000051305	0.917407212	-1.359291405	6.762481175	0.009309423	0.025956226
ENSRNOG00000020486	0.441731404	2.13674801	6.761122063	0.009316516	0.025971429
ENSRNOG00000011955	0.740302275	1.744899429	6.760034644	0.009322195	0.025982686
ENSRNOG00000061899	0.247885202	2.808979362	6.757713437	0.009334329	0.02600735
ENSRNOG00000013076	0.177304127	5.734685375	6.755436896	0.009346246	0.026030038
ENSRNOG00000018241	0.407310084	5.579394942	6.755215829	0.009347404	0.026030038
ENSRNOG00000014865	0.605550357	0.674091385	6.752089687	0.009363795	0.026071098
ENSRNOG00000031864	0.305790644	1.829106959	6.748991038	0.009380071	0.026103586
ENSRNOG00000031681	0.441370129	1.197027092	6.748810793	0.009381019	0.026103586
ENSRNOG00000020580	0.304873114	5.055797328	6.74764181	0.009387167	0.026112675
ENSRNOG00000034102	0.790804238	1.983862902	6.742091422	0.009416417	0.026185379
ENSRNOG00000004482	0.290514808	4.787028631	6.731890243	0.009470419	0.026307827
ENSRNOG00000035610	0.653247936	-0.723934361	6.731501309	0.009472484	0.026308948
ENSRNOG00000011593	0.372208015	2.733087236	6.730980739	0.009475249	0.026312012
ENSRNOG00000011394	0.27536287	6.937476189	6.713348381	0.009569388	0.026559454
ENSRNOG00000004532	0.238153078	5.045676528	6.712169148	0.009575718	0.026572364
ENSRNOG00000049785	0.234309696	5.268466906	6.701478653	0.009633299	0.026713425
ENSRNOG00000017448	0.525362067	0.258122598	6.697810057	0.00965314	0.026754389
ENSRNOG00000051915	0.309049336	2.939482904	6.696067811	0.009662578	0.026771174
ENSRNOG00000020426	0.504531545	4.119223257	6.694103598	0.009673229	0.026795996
ENSRNOG00000013552	0.281036021	4.467659279	6.693649113	0.009675695	0.026798139
ENSRNOG00000010718	0.458104217	3.140105713	6.690795597	0.009691195	0.02683168
ENSRNOG00000056819	0.244335287	3.654223284	6.689119005	0.009700313	0.02685223
ENSRNOG00000018461	0.254840713	4.544975073	6.688297678	0.009704783	0.026859909
ENSRNOG00000037668	1.171506896	0.357784683	6.68585241	0.009718104	0.026892077
ENSRNOG00000001425	0.818535702	1.368248401	6.67570195	0.009773601	0.027031477
ENSRNOG00000011659	0.443599168	1.612995027	6.675281681	0.009775906	0.027033129
ENSRNOG00000009263	0.337370261	5.228546682	6.672375473	0.009791859	0.027072516
ENSRNOG00000001480	0.318786973	1.97351042	6.661842159	0.009849904	0.027223491
ENSRNOG00000018639	0.260437199	3.560782991	6.646143245	0.009937069	0.027450027
ENSRNOG00000057945	0.311999145	1.37963031	6.64267598	0.009956427	0.027489114
ENSRNOG00000025509	0.238629104	3.358773832	6.638909086	0.009977501	0.027537695
ENSRNOG00000017047	0.372002914	1.939414271	6.633459842	0.010008069	0.027593202
ENSRNOG00000017663	0.283215089	3.712604828	6.631598161	0.010018534	0.027612439
ENSRNOG00000042978	0.32831685	7.686653826	6.631700815	0.010017956	0.027612439
ENSRNOG00000009336	0.650095473	1.75678919	6.627094831	0.010043895	0.027663077
ENSRNOG00000006093	0.258603602	4.555332531	6.625959205	0.0100503	0.027673137
ENSRNOG00000042658	0.704494974	-0.858474138	6.60202264	0.010186299	0.028011437
ENSRNOG00000046980	0.562415446	1.841161424	6.601558307	0.010188956	0.028013878
ENSRNOG00000019533	0.645996211	2.190508496	6.593036785	0.010237839	0.028133624
ENSRNOG00000025269	0.199810955	4.352248901	6.579252405	0.010317422	0.028337564
ENSRNOG00000025724	0.277717858	2.941087538	6.564049211	0.010405932	0.028541057
ENSRNOG00000019675	0.4110428	0.662725874	6.560028069	0.010429472	0.028600669
ENSRNOG00000032447	0.446472613	1.461523558	6.557263721	0.010445687	0.028640174
ENSRNOG00000060435	0.81924711	-1.018205213	6.556247636	0.010451653	0.028651571
ENSRNOG00000012287	0.311482892	3.783666385	6.554958249	0.010459229	0.028667377
ENSRNOG00000013712	0.178430769	4.548648892	6.544325595	0.010521921	0.028824237
ENSRNOG00000023639	0.39260145	4.557330413	6.542638615	0.010531903	0.028841602
ENSRNOG00000017575	0.271871232	4.799782984	6.53392913	0.010583592	0.028954569
ENSRNOG00000037687	1.413687532	1.332420352	6.53027586	0.010605351	0.029002612
ENSRNOG00000011073	0.154008742	7.149220765	6.524978247	0.010636985	0.029070661

ENSRNOG00000019244	0.352497115	2.728707995	6.520846074	0.010661727	0.029126602
ENSRNOG00000008674	0.229211664	3.431207785	6.517985443	0.01067889	0.029161971
ENSRNOG00000000441	0.478320508	1.484403083	6.508927145	0.010733425	0.029292149
ENSRNOG00000051310	0.711596717	-0.216871177	6.495766032	0.010813169	0.029499608
ENSRNOG00000046143	0.382662465	2.543022738	6.486918243	0.010867121	0.029621275
ENSRNOG00000007720	0.212171408	4.824018647	6.482864415	0.010891932	0.02967358
ENSRNOG00000019189	0.415210413	3.808878288	6.477619951	0.010924117	0.029751025
ENSRNOG00000047427	0.217928925	3.731593494	6.473814467	0.010947531	0.029804541
ENSRNOG00000020349	0.509613256	1.779454563	6.470645914	0.010967067	0.029852593
ENSRNOG00000005099	0.305170428	2.248093101	6.469464175	0.010974362	0.029867315
ENSRNOG00000052704	0.163244699	5.422752368	6.46558603	0.010998337	0.029927421
ENSRNOG00000017659	0.836176358	2.065571791	6.464997415	0.01100198	0.029927621
ENSRNOG00000009713	0.211082656	5.612447713	6.464963463	0.01100219	0.029927621
ENSRNOG00000062281	0.326869393	2.34198105	6.464515302	0.011004965	0.029930028
ENSRNOG00000014010	0.596434541	4.482331922	6.456724423	0.011053322	0.030051219
ENSRNOG00000010780	0.180781829	5.293813284	6.450501682	0.011092102	0.030146299
ENSRNOG00000050767	0.90503016	3.316505828	6.448756181	0.011103005	0.030170753
ENSRNOG00000008225	0.513337111	0.528660832	6.441806231	0.011146525	0.030273428
ENSRNOG00000020173	0.316136866	3.809229664	6.440264413	0.011156204	0.030294518
ENSRNOG00000020299	0.324893393	6.998751809	6.433029736	0.011201734	0.030398515
ENSRNOG00000047816	0.262507358	4.142623291	6.429849414	0.011221809	0.030441344
ENSRNOG00000027747	0.362647732	1.42669628	6.426851886	0.011240764	0.030482319
ENSRNOG00000010649	0.228310653	7.198827364	6.425443041	0.011249685	0.030501285
ENSRNOG00000058289	0.428866061	1.460939963	6.422389696	0.011269042	0.030548538
ENSRNOG00000052477	0.193170232	4.255545215	6.421662742	0.011273656	0.030555814
ENSRNOG00000024277	0.516153391	1.464894777	6.419592282	0.011286807	0.030586224
ENSRNOG00000053654	0.559939167	-0.578740196	6.418989354	0.01129064	0.030591374
ENSRNOG00000016275	1.721014921	2.621907389	6.417470523	0.011300301	0.030612311
ENSRNOG00000055634	0.679815259	-0.968857138	6.404416175	0.011383685	0.030822379
ENSRNOG00000000113	0.315228357	3.898068945	6.402077766	0.011398688	0.030854913
ENSRNOG00000008047	0.4344784	2.355454796	6.399136473	0.011417589	0.030890604
ENSRNOG00000006509	0.201141781	7.152102197	6.368600692	0.011615715	0.031370158
ENSRNOG00000015318	0.436838716	5.250411821	6.366392368	0.01163018	0.031403866
ENSRNOG00000010834	0.505611771	6.1124995	6.362953706	0.01165274	0.031459418
ENSRNOG00000037480	0.187211612	3.994275447	6.360355308	0.011669817	0.031494782
ENSRNOG00000013360	0.195388092	6.577133512	6.359694937	0.011674161	0.031501137
ENSRNOG00000050318	0.289794532	5.187881068	6.359026648	0.011678559	0.031507635
ENSRNOG00000012942	0.49013827	4.289643938	6.355526776	0.011701619	0.031560739
ENSRNOG00000009522	0.348108088	2.664384838	6.35543429	0.011702229	0.031560739
ENSRNOG00000005434	0.164879991	9.661518399	6.348845872	0.011745769	0.031667377
ENSRNOG00000058842	0.5515343	8.89990059	6.344875375	0.011772088	0.031732691
ENSRNOG00000058885	0.532548282	0.852595476	6.344586849	0.011774003	0.031732691
ENSRNOG00000049880	0.367931418	3.81767018	6.343413708	0.011781792	0.031748281
ENSRNOG00000019186	0.397107031	1.817061623	6.332691638	0.011853229	0.031919052
ENSRNOG00000017956	0.17160853	6.853079024	6.328660829	0.011880199	0.031975365
ENSRNOG00000048509	0.172875992	4.996743134	6.320221891	0.011936869	0.032122431
ENSRNOG00000011970	0.808216717	-0.957057904	6.318595862	0.01194782	0.032146438
ENSRNOG00000008625	0.287370552	3.021567155	6.31708258	0.011958021	0.032168418
ENSRNOG00000001314	0.225536323	5.060372677	6.304708701	0.01204177	0.032344255
ENSRNOG00000017893	0.893252401	6.983167987	6.301358051	0.012064551	0.032394513
ENSRNOG00000004234	0.216273326	4.164873251	6.290502533	0.012138663	0.032571359
ENSRNOG00000011954	0.771171211	0.782280881	6.285685519	0.012171699	0.03265447
ENSRNOG00000016697	0.447760736	2.140734902	6.282728602	0.012192024	0.032697917
ENSRNOG00000004276	0.40917329	4.773273782	6.281737778	0.012198842	0.032710663
ENSRNOG00000005575	0.344296431	1.767012287	6.278015742	0.01222449	0.032768339
ENSRNOG00000023549	0.251239745	3.560526805	6.276890384	0.012232256	0.032783605
ENSRNOG00000039668	0.747118494	0.355505776	6.270759815	0.01227465	0.032891657
ENSRNOG00000061366	0.371508934	2.620035205	6.26918796	0.012285543	0.032915278
ENSRNOG00000015593	0.478583587	0.920580399	6.262744792	0.012330302	0.033007271
ENSRNOG00000002214	0.18393208	5.636478379	6.26206957	0.012335003	0.033014271
ENSRNOG00000053040	0.409023241	2.955342604	6.259589993	0.012352279	0.033047299
ENSRNOG00000048606	0.654468521	0.952118176	6.258585917	0.012359282	0.033056903
ENSRNOG00000016050	0.312640089	6.025619964	6.251079354	0.012411765	0.033186068
ENSRNOG00000010650	0.391485038	5.61323334	6.247072295	0.012439875	0.033255611
ENSRNOG00000031439	0.449030172	0.958942249	6.243329984	0.012466187	0.033311498
ENSRNOG00000057194	0.181769524	4.932404046	6.239285051	0.012494691	0.033373976
ENSRNOG00000015744	0.317635955	1.695293801	6.236395745	0.012515092	0.033417194
ENSRNOG00000004307	0.264780615	2.602882246	6.230069345	0.012559882	0.033531136
ENSRNOG00000056407	0.864533938	-0.178940366	6.219713206	0.012633558	0.033705097
ENSRNOG00000047763	1.051694029	2.606841315	6.214455875	0.012671129	0.033799639
ENSRNOG00000011457	0.524993861	-0.093922411	6.213593663	0.012677302	0.033810409
ENSRNOG00000061484	0.976960398	1.587211597	6.211274693	0.012693919	0.033843203
ENSRNOG00000019556	0.24127647	6.16302357	6.210125609	0.012702162	0.033853901
ENSRNOG00000015807	0.285249329	3.339859797	6.201703605	0.012762741	0.034009634
ENSRNOG00000019316	0.271480937	4.502686891	6.194544655	0.012814469	0.034118766
ENSRNOG00000011138	0.261408887	6.95215874	6.193049748	0.012825298	0.034141857
ENSRNOG00000000658	0.453352034	3.0438342	6.187086359	0.012868589	0.034251343
ENSRNOG00000009540	0.400657276	1.286316852	6.18387964	0.01289193	0.034301936
ENSRNOG00000003084	0.204895077	5.563353966	6.181739748	0.01290753	0.034331906
ENSRNOG00000051006	0.301508103	3.598462046	6.178461732	0.012931465	0.034389792
ENSRNOG00000019582	0.269216091	4.161958102	6.173028664	0.012971236	0.034478186
ENSRNOG00000054224	0.340833989	2.754831388	6.165913375	0.013023511	0.034605517
ENSRNOG00000008443	0.185450652	6.801146794	6.161724403	0.013054388	0.03467974
ENSRNOG00000027988	0.189754427	5.581440316	6.157028498	0.013089092	0.034756444
ENSRNOG00000014745	0.255560585	3.610524835	6.154379084	0.013108713	0.034796875
ENSRNOG00000001878	0.215517089	5.967171459	6.147268074	0.013161526	0.034907806
ENSRNOG00000020068	0.195290618	4.679155502	6.142880643	0.01319422	0.034976943
ENSRNOG00000018371	0.380445635	1.926363959	6.134213791	0.013259049	0.035116376
ENSRNOG00000043141	0.224555847	4.967855382	6.132336542	0.013273134	0.035144951
ENSRNOG00000023008	0.591023655	2.419331532	6.130661792	0.013285713	0.035172376
ENSRNOG00000051941	0.341485095	2.783040672	6.129639788	0.013293395	0.035186831
ENSRNOG00000032720	0.48649966	0.069118202	6.125943385	0.013321218	0.035254583
ENSRNOG00000017836	0.309345087	3.209010255	6.111899234	0.013427476	0.035470581
ENSRNOG00000007705	0.21816984	6.481617313	6.11028836	0.01343972	0.035497002
ENSRNOG00000001228	0.282234937	4.652818592	6.105237736	0.013478182	0.03559265
ENSRNOG00000009722	0.206374998	8.948962201	6.104665296	0.013482548	0.035598244
ENSRNOG00000004736	0.245743216	3.788109075	6.100043499	0.013517855	0.035685516
ENSRNOG00000007529	0.2614622	2.084839142	6.098946177	0.013526252	0.035695779
ENSRNOG00000010841	0.516331951	0.743678916	6.0980592	0.013533043	0.03570775
ENSRNOG00000057934	0.381946003	6.995770447	6.09597399	0.013549022	0.035738001

ENSRNOG00000013564	0.557713163	-0.056703041	6.096066074	0.013548316	0.035738001
ENSRNOG00000051204	0.185444669	5.864549469	6.090385691	0.013591941	0.035841188
ENSRNOG00000004781	0.190267203	6.64231142	6.08462759	0.013636311	0.035943848
ENSRNOG00000000963	0.964413429	2.13185127	6.081770931	0.013658378	0.035975014
ENSRNOG00000021685	0.327130632	4.975479538	6.081049894	0.013663954	0.035981216
ENSRNOG00000016190	0.167108693	5.304047683	6.079725235	0.013674204	0.035996233
ENSRNOG00000000456	0.437414316	0.25858587	6.079927653	0.013672637	0.035996233
ENSRNOG00000016391	0.390139166	1.458216939	6.074034182	0.013718329	0.03608839
ENSRNOG00000000991	0.211580596	3.482764365	6.071714573	0.013736356	0.03611781
ENSRNOG00000015196	0.405300659	0.701794359	6.062522184	0.013808034	0.036282624
ENSRNOG00000013838	0.999983104	1.592731581	6.060954214	0.013820299	0.036308381
ENSRNOG00000014366	0.250170741	4.708970207	6.055973163	0.013859336	0.036388263
ENSRNOG00000005041	0.313662605	5.933870561	6.05532134	0.013864452	0.036394187
ENSRNOG00000016242	0.214189005	3.37645603	6.053852113	0.013875993	0.036417974
ENSRNOG00000028436	0.952327959	1.641774856	6.053582189	0.013878114	0.036417974
ENSRNOG00000020342	0.44241101	0.941091095	6.045659462	0.013940525	0.036557464
ENSRNOG00000023919	0.169998184	5.915121267	6.029740522	0.014066801	0.036845943
ENSRNOG00000025909	0.260648748	4.822932886	6.027248547	0.014086675	0.036891896
ENSRNOG00000019509	0.326467382	2.021266151	6.019849283	0.014145855	0.037031753
ENSRNOG00000051193	0.410294946	0.788811826	6.019694784	0.014147093	0.037031753
ENSRNOG00000010308	1.047203375	3.566120418	6.012835035	0.014202192	0.037163692
ENSRNOG00000017382	0.271170867	3.880066287	6.010393618	0.014221855	0.037208541
ENSRNOG00000042446	0.773529057	2.368353453	6.001832445	0.014291028	0.037365279
ENSRNOG00000015290	0.563553152	0.917213993	5.997483979	0.014326295	0.037451305
ENSRNOG00000020608	0.264797317	3.398650095	5.993131387	0.014361686	0.037519045
ENSRNOG00000003946	0.165341856	4.470682532	5.988281647	0.014401225	0.037603725
ENSRNOG00000013991	0.466945643	2.198404692	5.982669444	0.014447119	0.037717344
ENSRNOG00000030463	0.644754265	-0.842162313	5.976790456	0.014495358	0.037819658
ENSRNOG00000050430	0.398645535	0.864865151	5.976728885	0.014495864	0.037819658
ENSRNOG00000022586	0.154535042	5.976791892	5.964316056	0.014598262	0.038049199
ENSRNOG00000023561	1.134432843	0.560804159	5.961684703	0.014620065	0.038093484
ENSRNOG00000000341	0.316557251	1.590443651	5.960428235	0.014630487	0.038101831
ENSRNOG00000016534	0.229852907	5.542057348	5.960675903	0.014628432	0.038101831
ENSRNOG00000026005	0.164412751	4.080572986	5.958818176	0.014643854	0.038124101
ENSRNOG00000054420	0.634623517	-1.109765559	5.952951359	0.014692667	0.038226041
ENSRNOG00000061373	0.313877408	1.948366584	5.939936104	0.014801555	0.038458783
ENSRNOG00000021441	0.600492671	5.360662239	5.939136023	0.014808276	0.038463622
ENSRNOG00000057616	0.597515148	2.171975203	5.931456785	0.014872942	0.038599928
ENSRNOG00000001259	0.4625589	4.493145049	5.927085592	0.014909881	0.038683115
ENSRNOG00000002194	0.240059533	4.68044358	5.925723874	0.014921407	0.038706678
ENSRNOG00000054286	0.657591107	-0.102742734	5.923949784	0.014936438	0.038739322
ENSRNOG00000045952	0.345745333	2.226525607	5.922334392	0.014950138	0.038768504
ENSRNOG00000022417	0.436631596	0.306971939	5.91666655	0.014998309	0.038880684
ENSRNOG00000016566	0.305132072	5.10034514	5.913606357	0.015024383	0.038941903
ENSRNOG00000019979	0.219299313	3.35419117	5.902500993	0.015119401	0.039149729
ENSRNOG00000010205	0.196874122	4.815717384	5.898687154	0.015152175	0.039215354
ENSRNOG00000006622	0.216085698	4.044719713	5.89455777	0.015187743	0.039268896
ENSRNOG00000002343	0.296466402	8.849271951	5.894639358	0.015187039	0.039268896
ENSRNOG00000060193	0.850725738	-0.209046613	5.88721641	0.015251189	0.039400771
ENSRNOG00000016921	0.215603783	5.660191219	5.880459182	0.015309829	0.039539361
ENSRNOG00000055433	0.262628457	5.041293781	5.87615583	0.015347294	0.039611772
ENSRNOG00000030107	0.735506508	0.418508701	5.875802488	0.015350375	0.039611772
ENSRNOG00000011145	0.834310659	2.041457575	5.870286936	0.015398542	0.039716649
ENSRNOG00000009218	1.090709639	0.241187266	5.862037111	0.015470878	0.03987724
ENSRNOG00000012944	0.298094208	5.17785515	5.862242017	0.015469077	0.03987724
ENSRNOG00000028384	0.32308575	2.988927788	5.860125226	0.015487692	0.039914081
ENSRNOG00000022182	0.165689172	3.817923957	5.850998465	0.015568216	0.040102022
ENSRNOG00000043215	0.579059208	1.839661393	5.848553314	0.015589862	0.040138191
ENSRNOG00000019914	0.388523407	4.210300783	5.844992424	0.015621441	0.040193354
ENSRNOG00000042731	0.232390225	6.337106278	5.843733302	0.015632623	0.04021559
ENSRNOG00000061051	0.661331689	1.195868159	5.840875583	0.015658033	0.040274414
ENSRNOG00000020304	0.165197545	5.648363193	5.824635575	0.015803242	0.040608331
ENSRNOG00000013795	0.206144598	2.707051687	5.818711567	0.015856556	0.040738717
ENSRNOG00000015397	0.395208591	5.325078482	5.803246101	0.015996615	0.041025334
ENSRNOG00000014231	0.825655763	3.037525057	5.802529646	0.016003135	0.041035406
ENSRNOG00000006628	0.26151461	3.098734675	5.802142665	0.016006657	0.041037793
ENSRNOG00000015736	0.235790162	3.424069722	5.801441675	0.01601304	0.04104751
ENSRNOG00000018791	0.259958443	3.697535176	5.799195167	0.016033512	0.041087492
ENSRNOG00000020029	0.30367464	1.66678447	5.798068571	0.016043789	0.041100181
ENSRNOG00000013469	0.215084069	5.850220731	5.797764296	0.016046566	0.041100181
ENSRNOG00000018938	0.593413004	-0.595562677	5.797943583	0.016044929	0.041100181
ENSRNOG00000040076	0.531987113	1.159631828	5.790556371	0.016112489	0.041234094
ENSRNOG00000057315	0.87168289	4.574234578	5.785364357	0.016160148	0.041317581
ENSRNOG00000043201	0.29121007	4.580438493	5.782241614	0.016188882	0.041371008
ENSRNOG00000007901	0.176787609	6.433526541	5.780160777	0.016208059	0.041406648
ENSRNOG00000050190	0.315003721	4.338169588	5.779171165	0.016217187	0.041416603
ENSRNOG00000012148	0.224915602	6.869476805	5.777979351	0.016228187	0.041438013
ENSRNOG00000005058	0.315974298	5.40581881	5.764985479	0.01634862	0.041691746
ENSRNOG00000019453	0.228977495	6.593329027	5.759287774	0.016401719	0.041793502
ENSRNOG00000011489	0.320607824	6.260236417	5.75450585	0.016446421	0.041893923
ENSRNOG00000012322	0.205674696	2.784647807	5.753505945	0.016455784	0.041911031
ENSRNOG00000029528	0.20395865	6.246415539	5.747305588	0.016513966	0.042032172
ENSRNOG00000017230	0.282720528	3.077320076	5.746388405	0.016522591	0.042040608
ENSRNOG00000004837	0.198150704	3.482106935	5.740050394	0.016582317	0.042165473
ENSRNOG00000010911	0.529214188	2.372680253	5.737863731	0.016602974	0.042203906
ENSRNOG00000033087	0.639897565	2.085836115	5.736919214	0.016611905	0.042213595
ENSRNOG00000016248	0.394880006	1.387460167	5.732919236	0.016649783	0.042296272
ENSRNOG00000018500	0.294205184	3.77975333	5.729980696	0.016677666	0.042346723
ENSRNOG00000000801	0.223567521	2.57491643	5.728069725	0.016695825	0.042386033
ENSRNOG00000059768	0.357813978	1.034558742	5.725523894	0.016720047	0.042440723
ENSRNOG00000006403	0.465505989	5.573483079	5.717044049	0.016800993	0.042612032
ENSRNOG00000018616	0.446267528	0.769992999	5.715453645	0.016816219	0.042643819
ENSRNOG00000028444	0.349602729	1.958443006	5.714775685	0.016822714	0.042653459
ENSRNOG00000001448	0.167724605	6.322367626	5.711446735	0.016854644	0.042713897
ENSRNOG00000001141	0.506550115	2.220979298	5.711148944	0.016857503	0.042714306
ENSRNOG00000015297	0.560564618	-0.456953561	5.708296778	0.016884914	0.042770073
ENSRNOG00000034242	0.185031851	8.150866025	5.698229264	0.016982037	0.04298806
ENSRNOG00000029370	0.210141767	7.282267383	5.69797	0.016984546	0.04298806
ENSRNOG00000022802	0.216360522	5.665881605	5.693015468	0.017032562	0.043102699
ENSRNOG00000008519	0.298330093	5.118310517	5.690664058	0.017055399	0.043153594

ENSRNOG00000060972	0.209011752	5.555345288	5.682770002	0.017132299	0.043340496
ENSRNOG00000011936	0.237658591	3.819623644	5.682519835	0.017134742	0.043340496
ENSRNOG00000032395	0.1634011	4.254816214	5.681277442	0.017146879	0.043364268
ENSRNOG00000006738	0.271051271	3.572250918	5.6722803	0.017235038	0.043552441
ENSRNOG00000003947	0.475420792	3.308978854	5.664939728	0.017307312	0.043693236
ENSRNOG00000009220	0.259780632	3.548325949	5.656441182	0.017391377	0.043898464
ENSRNOG00000010488	0.251371509	6.932104431	5.654288196	0.017412741	0.04392438
ENSRNOG00000023536	0.576953082	-0.0291947	5.648828	0.017467042	0.044047324
ENSRNOG00000018986	0.214441227	5.398152473	5.643508335	0.017520115	0.04416006
ENSRNOG00000042411	0.365609717	3.41042603	5.637309428	0.017582169	0.044302365
ENSRNOG00000047040	0.472734582	1.035106435	5.63539052	0.017601424	0.044341718
ENSRNOG00000032018	0.972665211	-0.38716415	5.624603056	0.017710076	0.044589179
ENSRNOG00000057161	0.811833012	1.503496148	5.613200619	0.017825675	0.044844572
ENSRNOG00000025757	0.259778005	4.248380083	5.61107884	0.017847272	0.044891771
ENSRNOG00000014837	0.567351167	1.559073905	5.609114027	0.017867295	0.044927861
ENSRNOG00000014083	0.362147924	7.670349522	5.601980045	0.017940191	0.045082523
ENSRNOG00000014187	0.249276055	3.463334174	5.599120274	0.017969498	0.045149006
ENSRNOG00000059765	0.157137555	6.562014083	5.581352055	0.018152703	0.04555871
ENSRNOG00000049076	0.478601576	2.714143496	5.576787065	0.018200083	0.045655911
ENSRNOG00000020865	0.528692658	1.880451495	5.576366653	0.018204452	0.045659639
ENSRNOG00000004320	0.306780877	1.477067782	5.568087071	0.018290733	0.045854252
ENSRNOG00000008956	0.74365732	-0.006889243	5.566107411	0.018311425	0.04589886
ENSRNOG00000001136	0.230454936	7.750736815	5.559863048	0.018376852	0.046033709
ENSRNOG00000005008	0.701747138	0.330733581	5.558945092	0.01838649	0.046041509
ENSRNOG00000007000	0.69975594	-0.932707086	5.558735741	0.018388689	0.046041509
ENSRNOG00000049077	0.313638165	3.219206007	5.554643637	0.018431725	0.04612738
ENSRNOG00000008178	0.194545879	4.235242566	5.554163113	0.018436785	0.046132753
ENSRNOG00000031916	0.373110448	0.956181779	5.551629921	0.018463486	0.046192264
ENSRNOG00000031607	0.661857398	-0.681237581	5.546189991	0.01852096	0.046328732
ENSRNOG00000025910	0.180938842	4.65451042	5.545702666	0.018526117	0.046334314
ENSRNOG00000012521	0.673737707	0.004541609	5.534913298	0.018640689	0.04656937
ENSRNOG00000009515	0.190342315	4.188757978	5.533869029	0.018651817	0.04658982
ENSRNOG00000049983	0.517444765	4.858579296	5.530446084	0.01868834	0.046666327
ENSRNOG00000021765	0.383587091	1.200501393	5.513650498	0.018868625	0.047064557
ENSRNOG00000024089	0.227832816	3.668576968	5.511891817	0.018887607	0.047104483
ENSRNOG00000002575	0.261770266	3.579784985	5.511307912	0.018893913	0.04711279
ENSRNOG00000018859	0.171447572	4.837641418	5.508237305	0.018927113	0.047173289
ENSRNOG00000009033	0.440916159	6.534007987	5.505909765	0.018952319	0.047221245
ENSRNOG00000053274	0.509841184	0.03462257	5.505927866	0.018952122	0.047221245
ENSRNOG00000007064	0.185419939	4.182745547	5.503925508	0.018973834	0.04725998
ENSRNOG00000048644	0.361608086	1.658957864	5.496703911	0.019052353	0.047418257
ENSRNOG00000023348	0.406081881	1.276413677	5.492018414	0.019103476	0.047523086
ENSRNOG00000055093	0.238002276	4.584008289	5.483794379	0.019193552	0.047709687
ENSRNOG00000010492	0.242477937	3.952566979	5.48275779	0.019204937	0.047730493
ENSRNOG00000029227	0.820376849	-1.024448425	5.481948362	0.019213831	0.047737613
ENSRNOG00000009265	0.655293364	3.997437028	5.478466071	0.019252146	0.047817801
ENSRNOG00000001483	0.251614424	3.578157378	5.477963638	0.019257681	0.047824046
ENSRNOG00000022426	0.522635418	-0.281743187	5.475516743	0.019284658	0.047883532
ENSRNOG00000016103	0.294844248	2.710093068	5.468671532	0.019360337	0.048041305
ENSRNOG00000054157	0.229963406	6.136590017	5.468242035	0.019365095	0.048045584
ENSRNOG00000007433	0.342855842	5.632763053	5.467852268	0.019369415	0.048048772
ENSRNOG00000034089	0.745364082	2.357259181	5.462385189	0.019430106	0.048169141
ENSRNOG00000022472	0.26089254	4.518508625	5.454266388	0.019520599	0.048385905
ENSRNOG00000025702	0.182662319	4.159961222	5.452031725	0.019545583	0.04844025
ENSRNOG00000051077	0.263066015	3.586503983	5.450412305	0.019563709	0.048477585
ENSRNOG00000051190	0.225835521	4.186055636	5.449966437	0.019568703	0.048482372
ENSRNOG00000014429	0.169731983	5.874578284	5.447464756	0.019596745	0.048529069
ENSRNOG00000048431	0.495643601	4.253838289	5.446759202	0.019604662	0.048541082
ENSRNOG00000020102	0.322084815	7.361533448	5.443263705	0.019643931	0.048630707
ENSRNOG00000009465	0.448810407	1.201670254	5.442616778	0.019651208	0.048641116
ENSRNOG00000013581	0.173475806	5.049886599	5.433586362	0.019753073	0.04886105
ENSRNOG00000059175	0.725614848	-0.881076363	5.432403337	0.019766458	0.048875308
ENSRNOG00000015357	0.564904495	1.939784262	5.432317538	0.019767429	0.048875308
ENSRNOG00000020194	0.325291006	3.790168399	5.430622447	0.019786625	0.048915133
ENSRNOG00000003812	0.190779154	6.909113837	5.424001648	0.019861788	0.049085618
ENSRNOG00000024145	0.298252038	3.346173095	5.421452547	0.019890805	0.049149659
ENSRNOG00000042732	0.329970673	1.663951865	5.419577476	0.019912178	0.049171776
ENSRNOG00000025864	0.255569777	2.334814264	5.408074714	0.020043811	0.049489116
ENSRNOG00000026691	0.216809194	3.207547817	5.402936012	0.020102906	0.049612445
ENSRNOG00000007436	0.333497396	1.27509132	5.394097426	0.020204973	0.049809363
ENSRNOG00000013907	0.228005698	5.060123795	5.394145919	0.020204411	0.049809363

Worksheet 2: Rat DEGs downregulated in alcohol exposure

Gene	logFC	logCPM	LR	PValue	FDR
ENSRNOG00000034246	-2.478001466	6.666381876	598.5006253	3.55E-132	5.62E-128
ENSRNOG00000020274	-2.783662488	4.976439668	362.3090319	8.85E-81	7.00E-77
ENSRNOG00000000993	-2.012135297	4.17298916	353.9718251	5.78E-79	3.05E-75
ENSRNOG00000034180	-1.943097854	7.345104692	292.1108799	1.72E-65	6.83E-62
ENSRNOG00000046349	-3.802992286	3.750324371	279.9638937	7.65E-63	2.42E-59
ENSRNOG00000009870	-1.519403774	4.926642882	268.1650873	2.85E-60	7.52E-57
ENSRNOG00000043400	-2.091614335	2.296742041	260.3208471	1.46E-58	3.30E-55
ENSRNOG00000046172	-1.201522321	4.538568524	252.7963638	6.38E-57	1.26E-53
ENSRNOG00000038902	-1.549700164	4.495659161	219.3796153	1.24E-49	2.17E-46
ENSRNOG00000023385	-1.714341392	8.657991707	216.774992	4.57E-49	7.24E-46
ENSRNOG00000013583	-1.614603363	7.759699702	210.8840578	8.81E-48	1.16E-44
ENSRNOG00000050051	-1.316430725	4.90342343	186.963409	1.46E-42	1.78E-39
ENSRNOG00000010807	-1.359196595	8.176414645	173.8234224	1.08E-39	1.22E-36
ENSRNOG00000016580	-1.012436843	8.162307673	170.3967714	6.06E-39	6.40E-36
ENSRNOG00000020296	-1.763253386	6.475443789	167.9241258	2.10E-38	1.96E-35
ENSRNOG00000011494	-1.198780568	6.256724717	166.9058041	3.51E-38	3.09E-35
ENSRNOG00000012314	-1.301795721	6.145606598	165.690836	6.46E-38	5.12E-35
ENSRNOG00000050410	-0.884630439	5.921115094	164.7936978	1.01E-37	7.65E-35
ENSRNOG00000016723	-0.823601874	5.945302491	164.1681769	1.39E-37	1.00E-34
ENSRNOG00000018075	-0.812573371	6.279577012	161.3523289	5.73E-37	3.78E-34
ENSRNOG00000043969	-1.758959384	3.077704642	160.5146685	8.73E-37	5.53E-34
ENSRNOG00000046174	-1.159518549	4.200416605	157.5152117	3.95E-36	2.41E-33
ENSRNOG00000004723	-0.968257825	5.010550597	155.7588782	9.56E-36	5.61E-33
ENSRNOG00000031685	-1.585665211	4.978815952	155.5141775	1.08E-35	6.11E-33
ENSRNOG00000008489	-1.113684282	4.413693569	154.8726213	1.49E-35	8.15E-33
ENSRNOG00000045885	-2.242116935	5.553796618	153.9533143	2.37E-35	1.21E-32
ENSRNOG00000060293	-1.597973704	6.830887851	151.9977752	6.34E-35	3.14E-32
ENSRNOG00000012778	-1.460575371	4.790455422	149.0126301	2.85E-34	1.26E-31
ENSRNOG00000032882	-2.059815074	12.56970054	149.0051221	2.86E-34	1.26E-31
ENSRNOG00000050169	-1.0780248	5.342475925	148.8525703	3.09E-34	1.32E-31
ENSRNOG00000016961	-1.504128201	7.901767933	147.7825145	5.29E-34	2.11E-31
ENSRNOG00000029377	-1.605775642	1.093233257	147.765233	5.34E-34	2.11E-31
ENSRNOG00000011825	-1.057269765	6.597121643	147.8600154	5.09E-34	2.11E-31
ENSRNOG00000027049	-1.221257617	7.346677574	147.692047	5.54E-34	2.14E-31
ENSRNOG00000031802	-1.586124518	5.835016997	147.5802172	5.86E-34	2.21E-31
ENSRNOG00000020941	-1.1423374	6.372543052	147.0107001	7.81E-34	2.87E-31
ENSRNOG00000004196	-1.834280578	8.841132178	143.2250388	5.25E-33	1.89E-30
ENSRNOG00000037402	-1.106529184	6.242292384	142.7221104	6.76E-33	2.38E-30
ENSRNOG00000012550	-0.988574883	7.511435042	142.4334569	7.82E-33	2.69E-30
ENSRNOG00000002178	-1.262037539	4.79941607	142.3463063	8.17E-33	2.75E-30
ENSRNOG00000025388	-1.225380611	5.046817552	138.5260626	5.59E-32	1.81E-29
ENSRNOG00000047225	-1.043167006	6.031731968	136.057367	1.94E-31	6.02E-29
ENSRNOG00000038905	-1.556036039	2.703391174	133.1185975	8.52E-31	2.50E-28
ENSRNOG00000008490	-0.852741747	5.873207597	132.9827686	9.12E-31	2.63E-28
ENSRNOG00000038045	-2.53801011	3.813714636	131.7125534	1.73E-30	4.89E-28
ENSRNOG00000009297	-0.862315836	5.050126878	131.2493506	2.18E-30	5.96E-28
ENSRNOG00000053456	-0.846687728	5.501058004	130.0036712	4.09E-30	1.10E-27
ENSRNOG00000019277	-0.768963373	8.043195052	129.7460994	4.66E-30	1.23E-27
ENSRNOG00000024845	-1.423021947	5.284653873	127.7786525	1.25E-29	3.26E-27
ENSRNOG00000055485	-1.136063147	6.728089991	127.1835192	1.69E-29	4.33E-27
ENSRNOG00000033615	-2.161783449	11.23749031	126.823709	2.03E-29	5.10E-27
ENSRNOG00000013508	-1.281092007	8.084507506	126.3688509	2.55E-29	6.32E-27
ENSRNOG00000023344	-1.269534368	8.465148728	125.568346	3.82E-29	9.31E-27
ENSRNOG00000024967	-1.336087576	6.731843928	124.7470027	5.78E-29	1.39E-26
ENSRNOG00000028273	-1.419274263	4.470280863	124.0629463	8.16E-29	1.93E-26
ENSRNOG00000015636	-1.312691882	6.010008058	123.9377262	8.69E-29	2.02E-26
ENSRNOG00000055023	-1.404057038	3.710599628	123.4484361	1.11E-28	2.55E-26
ENSRNOG00000021215	-0.722446734	5.475301586	123.0099864	1.39E-28	3.14E-26
ENSRNOG00000012100	-1.099546413	3.996695226	121.0819213	3.67E-28	8.06E-26
ENSRNOG00000030339	-1.773563757	3.814394511	118.8493981	1.13E-27	2.39E-25
ENSRNOG00000026528	-0.855904908	5.183299997	118.7934168	1.16E-27	2.42E-25
ENSRNOG00000000781	-1.353819501	3.495481605	118.0617213	1.68E-27	3.37E-25
ENSRNOG00000030478	-1.594111613	13.23593324	117.784002	1.93E-27	3.83E-25
ENSRNOG00000019428	-0.844947501	6.487038826	116.6992508	3.34E-27	6.53E-25
ENSRNOG00000008459	-1.292420622	4.561429225	116.6333164	3.45E-27	6.67E-25
ENSRNOG00000012579	-1.215600683	8.53361865	116.5255688	3.65E-27	6.87E-25
ENSRNOG00000011104	-0.920971479	5.750831444	115.3665339	6.54E-27	1.22E-24
ENSRNOG00000032130	-2.303901439	3.408645144	111.7435773	4.07E-26	7.15E-24
ENSRNOG00000005184	-1.497308642	6.319682094	111.4116557	4.81E-26	8.27E-24
ENSRNOG00000013510	-0.791300082	4.356112604	111.2686783	5.17E-26	8.80E-24
ENSRNOG00000003626	-0.764968603	7.39422931	111.1255437	5.55E-26	9.36E-24
ENSRNOG00000005811	-1.638969427	3.909602596	110.8409657	6.41E-26	1.07E-23
ENSRNOG00000010019	-1.384542327	4.931862101	110.172151	8.98E-26	1.47E-23
ENSRNOG00000013714	-0.936824976	5.707287625	109.2280283	1.45E-25	2.34E-23
ENSRNOG00000029418	-1.641578789	1.510400288	108.6916929	1.90E-25	3.03E-23
ENSRNOG00000005698	-0.76776601	6.636517878	108.6336892	1.95E-25	3.09E-23
ENSRNOG00000030371	-1.3031843	14.39040989	108.5361997	2.05E-25	3.21E-23
ENSRNOG00000015703	-0.852545499	5.794800677	106.8615677	4.77E-25	7.41E-23
ENSRNOG00000030963	-1.066477936	6.239212317	106.6517189	5.31E-25	8.08E-23
ENSRNOG00000010657	-1.087007956	6.14877446	106.3442378	6.20E-25	9.26E-23
ENSRNOG00000001551	-0.782244942	7.143930079	105.7776251	8.25E-25	1.21E-22
ENSRNOG00000009057	-0.964469229	7.728441203	105.0361258	1.20E-24	1.74E-22
ENSRNOG00000031127	-0.918893651	4.637018532	104.3184433	1.72E-24	2.48E-22
ENSRNOG00000008555	-0.737463236	6.270925067	103.203496	3.02E-24	4.24E-22
ENSRNOG00000018042	-0.687341657	5.856865796	103.1398829	3.12E-24	4.34E-22
ENSRNOG00000019895	-0.801645976	6.832889858	102.0944877	5.29E-24	7.16E-22
ENSRNOG00000045860	-0.928271733	5.929008675	101.3186202	7.83E-24	1.05E-21
ENSRNOG00000032807	-1.428860467	2.362797596	100.0483306	1.49E-23	1.95E-21
ENSRNOG00000009512	-0.89503641	5.980444316	99.75289666	1.73E-23	2.22E-21
ENSRNOG00000011383	-0.706648289	7.312690267	99.29215228	2.18E-23	2.78E-21
ENSRNOG00000035529	-3.147321369	0.751807684	99.25632196	2.22E-23	2.81E-21
ENSRNOG00000020354	-1.065733353	4.448306479	98.82340105	2.76E-23	3.47E-21
ENSRNOG00000029389	-1.855998203	3.958780351	98.50703675	3.24E-23	4.04E-21
ENSRNOG00000054689	-0.96424889	7.314611035	97.41840666	5.61E-23	6.78E-21
ENSRNOG00000018680	-0.702051529	7.457842427	97.2920918	5.98E-23	7.18E-21
ENSRNOG00000005975	-1.052938673	7.58387092	96.66103848	8.23E-23	9.72E-21
ENSRNOG00000030291	-0.999747277	4.822694578	96.37227284	9.52E-23	1.11E-20
ENSRNOG00000004412	-1.253764318	3.785192871	96.2876481	9.93E-23	1.15E-20
ENSRNOG00000020442	-0.835768103	6.59812293	95.88808888	1.22E-22	1.39E-20

ENSRNOG00000012488	-0.713502385	5.15864155	95.72533328	1.32E-22	1.50E-20
ENSRNOG00000027503	-1.160108452	7.807976611	95.48506293	1.49E-22	1.67E-20
ENSRNOG00000036971	-0.854515529	4.512002794	94.68107408	2.24E-22	2.43E-20
ENSRNOG00000009846	-0.802520348	4.829212501	94.52948739	2.41E-22	2.60E-20
ENSRNOG00000026260	-0.673559092	7.635485721	94.16077229	2.91E-22	3.09E-20
ENSRNOG00000013975	-1.045209363	5.23016022	94.01096566	3.14E-22	3.31E-20
ENSRNOG00000010189	-1.008014884	8.196536373	93.61176452	3.84E-22	4.03E-20
ENSRNOG00000035628	-2.087366065	2.538547842	93.26746004	4.57E-22	4.64E-20
ENSRNOG00000031022	-0.759848878	5.52958444	92.83745702	5.68E-22	5.73E-20
ENSRNOG00000002838	-0.731172448	4.924754816	92.55618834	6.54E-22	6.56E-20
ENSRNOG00000001430	-0.828265172	4.427212999	91.50911067	1.11E-21	1.10E-19
ENSRNOG00000031706	-1.662254161	4.168660106	91.50403116	1.11E-21	1.10E-19
ENSRNOG00000004814	-1.125982725	5.247348605	90.61526472	1.75E-21	1.68E-19
ENSRNOG00000005828	-0.887392607	9.474378938	89.19328983	3.58E-21	3.32E-19
ENSRNOG00000008733	-0.986333972	4.157206345	88.5680586	4.91E-21	4.47E-19
ENSRNOG00000062128	-1.417729698	5.212273964	87.6814248	7.69E-21	6.92E-19
ENSRNOG00000005775	-0.881532577	7.624766355	87.48589222	8.49E-21	7.53E-19
ENSRNOG00000022860	-0.674118117	6.550205598	87.26550022	9.49E-21	8.35E-19
ENSRNOG00000042503	-0.604224817	7.37414992	86.29622493	1.55E-20	1.34E-18
ENSRNOG00000023360	-0.628716732	7.833415785	86.24789436	1.59E-20	1.37E-18
ENSRNOG00000004288	-0.726708383	3.950928804	85.98715331	1.81E-20	1.55E-18
ENSRNOG00000011384	-0.693021769	5.383652649	84.84872806	3.22E-20	2.68E-18
ENSRNOG00000031889	-1.324349035	4.301021965	84.80571311	3.29E-20	2.73E-18
ENSRNOG00000058882	-0.806793333	4.587765449	84.61601284	3.62E-20	2.97E-18
ENSRNOG00000012264	-0.905625734	5.365806476	84.14380608	4.60E-20	3.72E-18
ENSRNOG00000062130	-0.8835055	4.970954839	83.9558143	5.06E-20	4.07E-18
ENSRNOG00000047970	-0.753661014	4.016198668	83.90879397	5.18E-20	4.14E-18
ENSRNOG00000053334	-0.686205854	7.938268489	83.87261983	5.28E-20	4.20E-18
ENSRNOG00000007845	-0.557714473	5.143886399	83.41534173	6.65E-20	5.21E-18
ENSRNOG00000011949	-0.519893324	6.299195369	83.26883684	7.16E-20	5.59E-18
ENSRNOG00000033299	-1.100265631	13.83694792	82.99178059	8.24E-20	6.40E-18
ENSRNOG00000039850	-0.795422214	4.385690114	82.59026777	1.01E-19	7.76E-18
ENSRNOG00000018186	-0.788829549	5.200421641	82.03388527	1.34E-19	1.00E-17
ENSRNOG00000009823	-0.821482158	5.385340894	81.51215277	1.74E-19	1.29E-17
ENSRNOG00000062084	-1.298561782	1.223025917	81.48505147	1.77E-19	1.30E-17
ENSRNOG00000027646	-0.71144532	4.752926438	81.12552055	2.12E-19	1.55E-17
ENSRNOG00000001852	-0.548536608	5.949095083	81.12658391	2.12E-19	1.55E-17
ENSRNOG00000039249	-2.373400323	3.859324826	81.00312494	2.25E-19	1.62E-17
ENSRNOG00000047431	-1.840832134	0.858349693	80.6297695	2.72E-19	1.93E-17
ENSRNOG00000029670	-1.452378758	0.423990902	80.34140467	3.15E-19	2.23E-17
ENSRNOG00000014581	-0.866598934	5.483111678	80.25294878	3.29E-19	2.32E-17
ENSRNOG00000031780	-1.628610431	3.979572722	80.1037283	3.55E-19	2.49E-17
ENSRNOG00000015205	-0.65905112	6.019305407	79.93331714	3.87E-19	2.70E-17
ENSRNOG00000045570	-1.271463921	1.736095558	79.79416365	4.16E-19	2.87E-17
ENSRNOG00000009090	-0.976723308	6.287938777	79.72729903	4.30E-19	2.96E-17
ENSRNOG00000032825	-1.122673675	5.350012862	79.33620408	5.24E-19	3.55E-17
ENSRNOG00000017447	-0.625289368	6.664488696	78.9941589	6.23E-19	4.13E-17
ENSRNOG00000042886	-0.789544438	5.528716079	78.21079947	9.26E-19	6.08E-17
ENSRNOG00000031979	-1.236993273	15.10708442	77.81143752	1.13E-18	7.30E-17
ENSRNOG00000016387	-0.988371089	6.803837748	77.74633641	1.17E-18	7.51E-17
ENSRNOG00000003849	-0.785070965	4.267555597	77.27602635	1.49E-18	9.30E-17
ENSRNOG00000027040	-0.882356849	3.642705865	77.24969922	1.51E-18	9.39E-17
ENSRNOG00000012026	-0.707958155	7.273684984	76.81574575	1.88E-18	1.16E-16
ENSRNOG00000002671	-0.761563597	6.991454203	76.51634591	2.18E-18	1.34E-16
ENSRNOG00000013493	-0.572769633	6.432896501	75.27166826	4.10E-18	2.41E-16
ENSRNOG00000009364	-0.65334205	6.757686283	75.21943897	4.21E-18	2.47E-16
ENSRNOG00000005826	-2.085912738	3.307539132	75.14176199	4.38E-18	2.56E-16
ENSRNOG00000009663	-0.520409388	5.723347609	74.82391886	5.15E-18	2.95E-16
ENSRNOG00000010408	-0.738100221	4.795677718	74.70188347	5.47E-18	3.12E-16
ENSRNOG00000010241	-0.673836425	3.484133121	74.66782627	5.57E-18	3.16E-16
ENSRNOG00000009289	-0.939511481	4.992100909	74.59751797	5.77E-18	3.25E-16
ENSRNOG00000042696	-0.570954141	5.521698776	74.17269321	7.16E-18	4.00E-16
ENSRNOG00000051624	-0.569294034	5.715481124	73.7657517	8.80E-18	4.84E-16
ENSRNOG00000015215	-0.67418742	6.153036354	73.57326839	9.70E-18	5.31E-16
ENSRNOG00000005718	-1.293521752	2.754584181	73.46665881	1.02E-17	5.57E-16
ENSRNOG00000043819	-1.306414311	1.151082841	73.29110782	1.12E-17	6.05E-16
ENSRNOG00000062091	-0.578869331	6.374390465	72.86132271	1.39E-17	7.39E-16
ENSRNOG00000052730	-0.565132252	5.861895294	72.51391902	1.66E-17	8.78E-16
ENSRNOG00000017417	-0.693169161	7.193258666	72.25667341	1.89E-17	9.87E-16
ENSRNOG00000004107	-1.000203029	7.796693096	71.98820755	2.16E-17	1.13E-15
ENSRNOG00000019568	-1.119253119	5.464261146	71.72068414	2.48E-17	1.28E-15
ENSRNOG00000016530	-0.664868634	5.758510372	71.37372153	2.96E-17	1.52E-15
ENSRNOG00000005512	-0.822654158	8.004818297	70.53229362	4.53E-17	2.26E-15
ENSRNOG00000008236	-0.711903243	4.612070805	69.82126248	6.49E-17	3.19E-15
ENSRNOG00000049908	-0.717241056	4.498877603	69.60042729	7.26E-17	3.52E-15
ENSRNOG00000047067	-1.400693454	2.101968667	69.54351598	7.47E-17	3.59E-15
ENSRNOG00000011463	-0.684008265	6.155617492	69.45346209	7.82E-17	3.74E-15
ENSRNOG00000042233	-0.753777079	8.282032558	69.28440044	8.52E-17	4.05E-15
ENSRNOG00000043114	-0.842189971	6.096155249	69.13775267	9.18E-17	4.35E-15
ENSRNOG00000040303	-0.7045467	3.834050551	69.03713376	9.66E-17	4.55E-15
ENSRNOG00000011283	-0.83644707	3.356811454	68.67761369	1.16E-16	5.40E-15
ENSRNOG00000023020	-0.728909168	4.484801578	68.51033266	1.26E-16	5.81E-15
ENSRNOG00000025715	-1.040286877	7.824478311	68.22915512	1.46E-16	6.66E-15
ENSRNOG00000037567	-0.704840721	4.794693515	68.05578948	1.59E-16	7.23E-15
ENSRNOG00000050960	-0.672318248	3.386159601	68.01043763	1.63E-16	7.38E-15
ENSRNOG00000000340	-0.573121502	6.148738462	67.79990298	1.81E-16	8.12E-15
ENSRNOG00000049920	-0.667234676	6.632865689	67.69914129	1.90E-16	8.49E-15
ENSRNOG00000000989	-0.712170966	5.35204462	67.29126037	2.34E-16	1.04E-14
ENSRNOG00000003385	-0.681396319	5.385256989	66.9520226	2.78E-16	1.22E-14
ENSRNOG00000054183	-1.792257077	2.355703559	66.89715352	2.86E-16	1.25E-14
ENSRNOG00000046918	-0.569634537	4.926552243	66.75463453	3.07E-16	1.34E-14
ENSRNOG00000043866	-1.030577793	13.31620829	66.40205525	3.68E-16	1.58E-14
ENSRNOG00000045542	-1.860373051	6.215721978	66.35320786	3.77E-16	1.62E-14
ENSRNOG00000053479	-1.038264351	5.690637709	66.27580876	3.92E-16	1.67E-14
ENSRNOG00000019326	-0.676075501	6.470302125	66.03217893	4.44E-16	1.87E-14
ENSRNOG00000011745	-0.593302651	6.612490451	65.99276273	4.53E-16	1.91E-14
ENSRNOG00000052968	-0.618983825	7.509275797	65.77781401	5.05E-16	2.11E-14
ENSRNOG00000011732	-0.593159378	6.440676015	65.71468307	5.21E-16	2.18E-14
ENSRNOG00000001201	-0.63696856	5.656250937	65.28814388	6.47E-16	2.69E-14
ENSRNOG00000004967	-0.675740739	4.375798937	65.13168006	7.01E-16	2.90E-14

ENSRNOG00000032204	-1.005526746	5.423441504	65.10315237	7.11E-16	2.94E-14
ENSRNOG00000001820	-0.823660764	4.357752608	64.86559279	8.02E-16	3.28E-14
ENSRNOG00000018007	-0.579887255	5.617223861	64.6409143	8.99E-16	3.66E-14
ENSRNOG00000052273	-0.594518475	4.136087905	64.40855908	1.01E-15	4.08E-14
ENSRNOG00000061213	-0.67774346	5.171465214	64.15744102	1.15E-15	4.63E-14
ENSRNOG00000019908	-0.708166054	6.138112653	63.9614428	1.27E-15	5.06E-14
ENSRNOG00000055652	-1.039822026	2.38498508	63.58748116	1.53E-15	6.04E-14
ENSRNOG00000018066	-0.537637612	5.488612957	63.20689341	1.86E-15	7.28E-14
ENSRNOG00000042126	-0.788773137	3.429828167	63.05282225	2.01E-15	7.83E-14
ENSRNOG00000020674	-0.854851477	7.022915002	62.92996676	2.14E-15	8.25E-14
ENSRNOG00000001073	-0.754184715	3.035341911	62.90064638	2.17E-15	8.36E-14
ENSRNOG00000007998	-0.661079035	7.219590346	62.8011606	2.29E-15	8.72E-14
ENSRNOG00000004909	-0.593345142	6.029164406	62.49299624	2.67E-15	1.02E-13
ENSRNOG00000045961	-0.670591962	4.610973786	62.48793706	2.68E-15	1.02E-13
ENSRNOG00000059750	-1.384680158	2.017962291	62.41075531	2.79E-15	1.05E-13
ENSRNOG00000059100	-0.749384853	2.720124876	62.30159535	2.95E-15	1.10E-13
ENSRNOG00000018676	-0.521941854	6.607530054	62.16083672	3.17E-15	1.18E-13
ENSRNOG00000026488	-1.547233975	2.136661407	61.19919126	5.16E-15	1.89E-13
ENSRNOG00000030700	-1.323735326	14.93731425	60.96927289	5.80E-15	2.12E-13
ENSRNOG00000019439	-0.510787283	5.285634915	60.83021192	6.22E-15	2.26E-13
ENSRNOG00000017571	-0.827573903	5.742200859	60.61102227	6.95E-15	2.50E-13
ENSRNOG00000008781	-0.586688894	5.025754912	60.48200627	7.43E-15	2.65E-13
ENSRNOG00000011175	-0.668097288	8.813111987	60.41732734	7.67E-15	2.74E-13
ENSRNOG00000029707	-1.226319399	11.19191428	60.21114785	8.52E-15	3.03E-13
ENSRNOG00000055079	-1.214095615	3.593155073	60.16451926	8.73E-15	3.10E-13
ENSRNOG00000012879	-0.882180445	4.797225314	60.10786752	8.98E-15	3.18E-13
ENSRNOG00000060539	-1.063072938	1.508405406	59.99391675	9.52E-15	3.36E-13
ENSRNOG00000031033	-1.307539594	14.53867972	59.92382446	9.86E-15	3.47E-13
ENSRNOG00000039871	-0.579617014	5.14029919	59.77272811	1.06E-14	3.74E-13
ENSRNOG00000002721	-0.93411138	6.623870019	59.65637976	1.13E-14	3.95E-13
ENSRNOG00000056098	-0.499487998	4.249947624	59.14041605	1.47E-14	5.05E-13
ENSRNOG00000005012	-0.611846679	4.102625368	59.01458949	1.57E-14	5.36E-13
ENSRNOG00000004553	-0.60408911	5.455075459	58.85461825	1.70E-14	5.77E-13
ENSRNOG00000011817	-0.895438867	5.893759904	58.82236182	1.73E-14	5.85E-13
ENSRNOG00000032740	-1.055638227	3.546303464	58.56257365	1.97E-14	6.66E-13
ENSRNOG00000037376	-0.608295244	3.783579683	58.50116416	2.03E-14	6.83E-13
ENSRNOG00000000990	-0.874796767	6.795744775	58.46427079	2.07E-14	6.94E-13
ENSRNOG00000050932	-1.140232025	4.507716428	58.38327766	2.16E-14	7.18E-13
ENSRNOG00000028585	-0.725339967	6.072992479	58.27845365	2.28E-14	7.54E-13
ENSRNOG00000062108	-0.542780429	4.728156938	58.11189163	2.48E-14	8.17E-13
ENSRNOG00000029145	-1.411785883	7.20231851	58.0155053	2.60E-14	8.54E-13
ENSRNOG00000002662	-0.608354819	4.550758495	57.89769401	2.76E-14	9.01E-13
ENSRNOG00000054806	-1.004409952	1.075973788	57.8178978	2.88E-14	9.37E-13
ENSRNOG00000032348	-1.421409725	2.90E-05	57.64902126	3.13E-14	1.02E-12
ENSRNOG00000012641	-0.657210575	6.527886072	57.40192147	3.55E-14	1.14E-12
ENSRNOG00000056765	-0.501342498	7.941206735	57.26675617	3.81E-14	1.22E-12
ENSRNOG00000013845	-0.510942444	4.016172013	57.09493941	4.15E-14	1.32E-12
ENSRNOG00000009884	-1.054369207	3.142076425	57.04510755	4.26E-14	1.35E-12
ENSRNOG00000005123	-0.771196208	6.067157224	56.92820385	4.52E-14	1.42E-12
ENSRNOG00000045647	-0.797864863	5.247825065	56.84375573	4.72E-14	1.47E-12
ENSRNOG00000059714	-0.759510912	7.593098944	56.65307993	5.20E-14	1.61E-12
ENSRNOG00000033803	-1.971906773	-0.652465284	56.43012196	5.82E-14	1.79E-12
ENSRNOG00000003822	-0.51325143	5.958832968	55.64543153	8.68E-14	2.61E-12
ENSRNOG00000005102	-0.57030838	4.181177311	55.62742386	8.76E-14	2.63E-12
ENSRNOG00000012085	-0.542037512	4.787775788	55.3495857	1.01E-13	3.02E-12
ENSRNOG00000029954	-1.499205376	6.971744569	55.27740437	1.05E-13	3.12E-12
ENSRNOG00000029171	-1.44081227	5.28651601	55.08447291	1.15E-13	3.43E-12
ENSRNOG00000014154	-0.428817102	6.828128257	54.82140951	1.32E-13	3.88E-12
ENSRNOG00000011714	-0.826698684	5.124636978	54.78557061	1.34E-13	3.95E-12
ENSRNOG00000029070	-1.216663255	5.784686107	54.76440446	1.36E-13	3.98E-12
ENSRNOG00000010156	-0.557899728	5.699918001	54.58021319	1.49E-13	4.35E-12
ENSRNOG00000047053	-0.468377411	5.598620732	54.41066207	1.63E-13	4.69E-12
ENSRNOG00000020595	-0.618125427	7.892178964	54.26731934	1.75E-13	5.00E-12
ENSRNOG00000019426	-0.523204782	4.944444139	54.26236675	1.75E-13	5.00E-12
ENSRNOG00000001238	-0.523158166	6.288662478	54.26516039	1.75E-13	5.00E-12
ENSRNOG00000042740	-1.410033587	5.530566872	54.22514579	1.79E-13	5.08E-12
ENSRNOG00000029339	-0.941021251	3.680385611	54.21187863	1.80E-13	5.11E-12
ENSRNOG00000008090	-0.480267729	5.489136392	54.15183874	1.86E-13	5.26E-12
ENSRNOG00000029574	-0.864626218	3.113003911	53.655007	2.39E-13	6.67E-12
ENSRNOG00000055096	-0.703544991	4.174928267	53.59148768	2.47E-13	6.88E-12
ENSRNOG00000008546	-0.498863926	5.02670512	53.58097932	2.48E-13	6.91E-12
ENSRNOG00000055020	-1.051082595	1.765930313	53.54391138	2.53E-13	7.01E-12
ENSRNOG00000042903	-0.851141721	6.688970568	53.4786505	2.61E-13	7.24E-12
ENSRNOG00000037998	-0.779138341	6.419656826	53.46348028	2.63E-13	7.28E-12
ENSRNOG00000019501	-0.529709787	5.049892031	53.37511106	2.76E-13	7.59E-12
ENSRNOG00000054626	-0.566497129	8.046449729	53.29100252	2.88E-13	7.89E-12
ENSRNOG00000019178	-0.873364935	4.872534181	53.09057388	3.19E-13	8.71E-12
ENSRNOG00000061441	-2.892687537	-0.161809409	53.06502204	3.23E-13	8.81E-12
ENSRNOG00000002563	-0.648257872	5.287813094	52.75002348	3.79E-13	1.03E-11
ENSRNOG00000009888	-0.637733666	6.844990633	52.74092018	3.81E-13	1.03E-11
ENSRNOG00000059181	-1.082012596	0.462696131	52.58254336	4.13E-13	1.11E-11
ENSRNOG00000047853	-0.570810479	5.119966657	52.48457802	4.34E-13	1.17E-11
ENSRNOG00000030120	-0.491883219	4.466792194	52.45615154	4.40E-13	1.18E-11
ENSRNOG00000030871	-1.037269056	9.344753691	52.35140674	4.64E-13	1.23E-11
ENSRNOG00000012803	-0.447151466	5.547922352	52.08928901	5.30E-13	1.39E-11
ENSRNOG00000047019	-0.814367175	2.962519052	51.91461018	5.80E-13	1.51E-11
ENSRNOG00000051238	-0.528092137	6.36686432	51.85550677	5.97E-13	1.56E-11
ENSRNOG00000006992	-0.609273569	8.281176743	51.79389166	6.16E-13	1.60E-11
ENSRNOG00000011893	-0.584117562	8.039125951	51.72049604	6.40E-13	1.66E-11
ENSRNOG00000009958	-0.570988034	7.225208123	51.56811919	6.92E-13	1.78E-11
ENSRNOG00000038012	-0.535138556	3.865543236	51.33841798	7.77E-13	1.99E-11
ENSRNOG00000062158	-1.136891397	6.093374564	51.12559407	8.66E-13	2.20E-11
ENSRNOG00000001274	-0.921208	6.530293465	51.0832659	8.85E-13	2.25E-11
ENSRNOG00000055952	-1.251854504	1.300812474	51.07177174	8.90E-13	2.26E-11
ENSRNOG00000037627	-0.671141524	6.029579749	50.95330741	9.46E-13	2.39E-11
ENSRNOG00000034182	-1.432240283	0.290633809	50.92943985	9.57E-13	2.42E-11
ENSRNOG00000007788	-0.480820378	6.058677535	50.77179236	1.04E-12	2.61E-11
ENSRNOG00000019838	-0.989485312	1.644051164	50.69342843	1.08E-12	2.70E-11
ENSRNOG00000060649	-1.014165644	2.217865683	50.61261798	1.13E-12	2.81E-11
ENSRNOG00000013255	-0.561096472	6.443804992	50.57769093	1.15E-12	2.85E-11

ENSRNOG00000008226	-0.589444671	3.672262355	50.54179502	1.17E-12	2.90E-11
ENSRNOG00000010921	-0.665168245	4.17260964	50.48982027	1.20E-12	2.96E-11
ENSRNOG00000009781	-0.433627814	7.357996624	50.47965507	1.20E-12	2.97E-11
ENSRNOG00000050087	-0.793352008	5.087417267	50.31055192	1.31E-12	3.22E-11
ENSRNOG00000030644	-1.407943465	14.42857155	50.24300299	1.36E-12	3.32E-11
ENSRNOG00000018610	-0.518148352	5.001442372	50.14858564	1.43E-12	3.47E-11
ENSRNOG00000013393	-0.498291862	5.360200059	49.95183877	1.58E-12	3.83E-11
ENSRNOG00000004751	-0.50572132	5.567477352	49.88063915	1.63E-12	3.96E-11
ENSRNOG00000002473	-0.598450611	3.734845053	49.86649669	1.65E-12	3.98E-11
ENSRNOG00000015720	-0.446404068	5.432240605	49.8526773	1.66E-12	3.99E-11
ENSRNOG00000000436	-0.888259167	3.160632625	49.65714021	1.83E-12	4.39E-11
ENSRNOG00000018230	-0.575313228	3.535403942	49.44321629	2.04E-12	4.87E-11
ENSRNOG00000046460	-0.587049442	3.622330568	49.30964493	2.19E-12	5.20E-11
ENSRNOG00000061152	-0.497614843	6.531244278	48.90878019	2.68E-12	6.26E-11
ENSRNOG00000005087	-0.507284352	5.850540481	48.6326755	3.09E-12	7.16E-11
ENSRNOG00000034234	-0.957901177	15.82238696	48.41343957	3.45E-12	7.97E-11
ENSRNOG00000019578	-0.583547145	8.371765968	48.33218524	3.60E-12	8.28E-11
ENSRNOG00000051377	-0.700697859	5.464531015	48.2606996	3.73E-12	8.56E-11
ENSRNOG00000031313	-1.389241323	0.303048015	48.22115947	3.81E-12	8.72E-11
ENSRNOG00000012985	-0.538636692	6.288316917	48.03989348	4.18E-12	9.54E-11
ENSRNOG00000042916	-0.507251875	4.66924133	47.97359184	4.32E-12	9.86E-11
ENSRNOG00000010746	-0.75995566	7.318091858	47.96382199	4.34E-12	9.89E-11
ENSRNOG00000014846	-0.913559702	5.36096324	47.9161554	4.45E-12	1.01E-10
ENSRNOG00000027213	-0.625578564	4.90674074	47.87852199	4.53E-12	1.03E-10
ENSRNOG00000009080	-0.537867153	8.010001474	47.7784562	4.77E-12	1.08E-10
ENSRNOG00000021847	-0.601788827	4.143639762	47.57127286	5.30E-12	1.19E-10
ENSRNOG00000001829	-0.576194544	6.658100506	47.54269801	5.38E-12	1.21E-10
ENSRNOG00000023591	-0.462526809	4.891786914	47.46728362	5.59E-12	1.25E-10
ENSRNOG00000008673	-0.499414162	6.681490223	47.40145579	5.78E-12	1.29E-10
ENSRNOG00000000397	-0.587731846	6.451601449	47.39620575	5.80E-12	1.29E-10
ENSRNOG00000029785	-0.533572706	5.155788111	47.22951922	6.31E-12	1.40E-10
ENSRNOG00000019106	-0.875591753	6.779867173	47.12846892	6.65E-12	1.47E-10
ENSRNOG00000012081	-0.631390298	5.281273315	47.07227211	6.84E-12	1.51E-10
ENSRNOG00000010912	-0.679995327	5.497357574	46.8794769	7.55E-12	1.65E-10
ENSRNOG00000046905	-0.598032332	5.156763477	46.86279409	7.61E-12	1.66E-10
ENSRNOG00000029042	-1.057159938	11.85681397	46.71821602	8.20E-12	1.78E-10
ENSRNOG00000039994	-0.621192604	5.814771093	46.53875329	8.98E-12	1.94E-10
ENSRNOG00000056429	-1.757438553	-0.410262772	46.52833974	9.03E-12	1.95E-10
ENSRNOG00000004401	-0.510116585	5.10427884	46.41701934	9.56E-12	2.06E-10
ENSRNOG00000014674	-0.496601473	4.981651293	46.23912655	1.05E-11	2.24E-10
ENSRNOG00000018471	-1.078638185	1.125343011	46.10332201	1.12E-11	2.39E-10
ENSRNOG00000018415	-0.617486888	3.957947504	46.01797598	1.17E-11	2.49E-10
ENSRNOG00000061688	-1.379402904	-0.267806843	45.87129823	1.26E-11	2.68E-10
ENSRNOG00000052934	-0.511896768	4.78262307	45.82318803	1.29E-11	2.74E-10
ENSRNOG00000021173	-0.416557268	5.464323336	45.73044649	1.36E-11	2.86E-10
ENSRNOG00000027126	-0.570750164	4.494667858	45.55468457	1.48E-11	3.11E-10
ENSRNOG00000018207	-1.089309242	4.053410574	45.5275971	1.51E-11	3.15E-10
ENSRNOG00000049370	-0.588363013	4.209668755	45.43403011	1.58E-11	3.30E-10
ENSRNOG00000036839	-0.461169524	8.222080248	45.42789284	1.58E-11	3.30E-10
ENSRNOG00000016532	-0.467772746	6.091340423	45.35056999	1.65E-11	3.41E-10
ENSRNOG00000006976	-0.721548581	4.004715772	45.2967637	1.69E-11	3.50E-10
ENSRNOG00000038183	-0.760111004	2.460930576	45.18101134	1.80E-11	3.70E-10
ENSRNOG00000023035	-0.579289946	3.276331659	45.11591051	1.86E-11	3.81E-10
ENSRNOG00000023529	-0.474050584	8.400562387	45.07610251	1.90E-11	3.88E-10
ENSRNOG00000035599	-1.783869242	-0.677819015	44.85461277	2.12E-11	4.31E-10
ENSRNOG00000013300	-0.541645779	6.453925815	44.83155978	2.15E-11	4.36E-10
ENSRNOG00000003011	-0.476678762	4.661630297	44.75110351	2.24E-11	4.52E-10
ENSRNOG00000037221	-1.065291731	1.839237856	44.69977728	2.30E-11	4.64E-10
ENSRNOG00000043105	-0.540496997	4.53673964	44.65275823	2.35E-11	4.73E-10
ENSRNOG00000021132	-0.831506198	4.965551174	44.60456319	2.41E-11	4.85E-10
ENSRNOG00000014058	-0.413818815	6.013688875	44.58083196	2.44E-11	4.89E-10
ENSRNOG00000016016	-0.735551993	6.833874021	44.38821328	2.69E-11	5.37E-10
ENSRNOG00000004135	-0.624252533	3.402061446	44.37718651	2.71E-11	5.39E-10
ENSRNOG00000047394	-0.515038567	6.37884967	44.31185475	2.80E-11	5.56E-10
ENSRNOG00000006499	-0.423572733	5.973575236	44.27207092	2.86E-11	5.66E-10
ENSRNOG00000001060	-0.553966357	4.778935317	44.23430267	2.91E-11	5.77E-10
ENSRNOG00000019473	-1.314810475	0.699542138	44.16202771	3.02E-11	5.96E-10
ENSRNOG00000009893	-0.587523755	3.697501642	44.09479488	3.13E-11	6.16E-10
ENSRNOG00000020356	-0.630661455	4.375683977	44.06374437	3.18E-11	6.24E-10
ENSRNOG00000047761	-0.467968503	4.484589933	44.04012934	3.22E-11	6.30E-10
ENSRNOG00000042561	-0.790615718	2.558234044	44.03358515	3.23E-11	6.32E-10
ENSRNOG00000016829	-0.697463166	2.909215858	43.95793734	3.36E-11	6.55E-10
ENSRNOG00000019973	-0.765867498	6.717671356	43.85121353	3.54E-11	6.90E-10
ENSRNOG00000051341	-0.625958914	3.898698859	43.82950354	3.58E-11	6.97E-10
ENSRNOG00000055314	-0.535090967	4.739395146	43.77205431	3.69E-11	7.16E-10
ENSRNOG00000011135	-0.569301173	4.800656188	43.70212717	3.82E-11	7.38E-10
ENSRNOG00000015939	-0.506586012	5.571951622	43.63349356	3.96E-11	7.64E-10
ENSRNOG00000010727	-0.684236807	6.826899794	43.62733225	3.97E-11	7.64E-10
ENSRNOG00000052869	-0.864296603	2.439015612	43.45880364	4.33E-11	8.27E-10
ENSRNOG00000003670	-0.630259253	5.700551319	43.18533823	4.98E-11	9.43E-10
ENSRNOG00000032997	-1.083753734	7.755841795	43.1875802	4.97E-11	9.43E-10
ENSRNOG00000019701	-0.640158598	3.459915218	43.14660663	5.08E-11	9.61E-10
ENSRNOG00000016213	-0.688320966	6.412334071	43.05380488	5.33E-11	1.00E-09
ENSRNOG00000030237	-0.770735211	7.373744025	42.96154475	5.58E-11	1.04E-09
ENSRNOG00000018653	-0.505259478	6.470511276	42.92840355	5.68E-11	1.06E-09
ENSRNOG00000014072	-0.504300399	4.794755199	42.74668157	6.23E-11	1.16E-09
ENSRNOG00000015848	-1.162111386	2.943309501	42.59191901	6.74E-11	1.24E-09
ENSRNOG00000030106	-0.653529531	5.0049573	42.49183459	7.10E-11	1.30E-09
ENSRNOG00000014475	-0.475051993	4.383331149	42.38783051	7.49E-11	1.37E-09
ENSRNOG00000016231	-0.491299437	3.665756721	42.38232063	7.51E-11	1.37E-09
ENSRNOG00000058677	-0.613887543	4.533230172	42.19181724	8.27E-11	1.50E-09
ENSRNOG00000017418	-0.479864556	7.917703242	42.14533712	8.47E-11	1.53E-09
ENSRNOG00000056735	-0.482143267	4.998793181	42.11107476	8.62E-11	1.55E-09
ENSRNOG00000053230	-0.427117899	4.93245757	42.01463314	9.06E-11	1.62E-09
ENSRNOG00000021493	-1.115510939	1.017611456	41.89404029	9.64E-11	1.72E-09
ENSRNOG00000013238	-0.87426042	4.275329734	41.80694975	1.01E-10	1.79E-09
ENSRNOG00000013965	-0.502820795	4.215197528	41.75306977	1.04E-10	1.84E-09
ENSRNOG00000033024	-1.203921826	-0.166022897	41.75433338	1.03E-10	1.84E-09
ENSRNOG00000008921	-0.468824462	9.907067269	41.7246201	1.05E-10	1.86E-09
ENSRNOG00000011905	-0.468209672	8.198698004	41.70453589	1.06E-10	1.88E-09

ENSRNOG00000032274	-2.116031498	9.977036103	41.62969016	1.10E-10	1.94E-09
ENSRNOG00000029667	-0.422678924	5.913744685	41.59977314	1.12E-10	1.97E-09
ENSRNOG00000031766	-0.999124183	14.66292607	41.5786795	1.13E-10	1.99E-09
ENSRNOG00000039848	-0.488216795	6.483005274	41.53826406	1.16E-10	2.02E-09
ENSRNOG00000051258	-0.497174321	6.361375675	41.53690456	1.16E-10	2.02E-09
ENSRNOG00000017340	-0.510757549	5.326105241	41.42092763	1.23E-10	2.14E-09
ENSRNOG00000016677	-0.493900072	5.165336386	41.40855574	1.24E-10	2.15E-09
ENSRNOG00000009035	-0.599967324	3.672346375	41.33835198	1.28E-10	2.22E-09
ENSRNOG00000004777	-0.553878438	5.44227767	41.28725151	1.31E-10	2.28E-09
ENSRNOG00000012155	-0.510879601	4.183863082	41.17603091	1.39E-10	2.40E-09
ENSRNOG00000005203	-0.953180244	5.331402964	41.17377673	1.39E-10	2.40E-09
ENSRNOG00000015213	-0.68773501	3.281589756	41.16504275	1.40E-10	2.41E-09
ENSRNOG00000013254	-0.971461525	6.090890191	41.04104605	1.49E-10	2.56E-09
ENSRNOG00000025981	-0.944066216	2.877300004	41.02619535	1.50E-10	2.57E-09
ENSRNOG00000057649	-0.565652035	3.230871182	41.02558613	1.50E-10	2.57E-09
ENSRNOG00000056628	-0.957403798	0.870931598	41.01153563	1.51E-10	2.59E-09
ENSRNOG00000009849	-0.452527648	4.019694384	40.95980798	1.55E-10	2.65E-09
ENSRNOG00000018510	-0.512663734	3.952354744	40.95591102	1.56E-10	2.65E-09
ENSRNOG00000030520	-0.600413277	5.726044866	40.70972711	1.77E-10	2.98E-09
ENSRNOG00000027039	-0.522806728	3.88553616	40.5726942	1.89E-10	3.19E-09
ENSRNOG00000031653	-0.766054674	3.234944299	40.5585302	1.91E-10	3.21E-09
ENSRNOG00000004616	-0.489800637	7.72673315	40.55383636	1.91E-10	3.21E-09
ENSRNOG00000012393	-0.720865287	5.423830088	40.45659887	2.01E-10	3.37E-09
ENSRNOG00000025592	-0.363267972	5.872375395	40.39364123	2.08E-10	3.47E-09
ENSRNOG00000010386	-0.69250845	3.879223605	40.39389076	2.08E-10	3.47E-09
ENSRNOG00000002280	-0.654911351	5.143715372	40.35450093	2.12E-10	3.53E-09
ENSRNOG00000003717	-0.485859511	4.353693788	40.34203314	2.13E-10	3.55E-09
ENSRNOG00000054446	-0.434547453	6.504084858	40.31813032	2.16E-10	3.59E-09
ENSRNOG00000022502	-0.678146477	4.7499569	40.29564547	2.18E-10	3.63E-09
ENSRNOG00000014284	-0.488289965	4.733528134	40.23411911	2.25E-10	3.74E-09
ENSRNOG00000037298	-1.124404525	3.809051958	40.21064999	2.28E-10	3.78E-09
ENSRNOG00000002255	-0.497965997	5.72396882	40.19449789	2.30E-10	3.80E-09
ENSRNOG00000000901	-0.5060423	5.446704221	40.15891206	2.34E-10	3.86E-09
ENSRNOG00000047708	-0.413977838	5.149457281	40.11593509	2.39E-10	3.94E-09
ENSRNOG00000020578	-0.7754191	2.495161033	39.91848354	2.65E-10	4.34E-09
ENSRNOG00000000587	-0.584695022	4.684262126	39.75777301	2.87E-10	4.70E-09
ENSRNOG00000012015	-0.412576181	6.556114223	39.5891682	3.13E-10	5.11E-09
ENSRNOG00000032605	-0.707762836	2.435158511	39.55446295	3.19E-10	5.19E-09
ENSRNOG00000012183	-0.548732599	5.554584814	39.48876285	3.30E-10	5.35E-09
ENSRNOG00000002196	-0.855855397	4.447933883	39.47656903	3.32E-10	5.38E-09
ENSRNOG00000012684	-0.405127458	4.984288228	39.29697632	3.64E-10	5.87E-09
ENSRNOG00000003951	-0.589879528	7.688562442	39.18463973	3.86E-10	6.21E-09
ENSRNOG00000014621	-0.470307185	5.489181343	39.17339314	3.88E-10	6.23E-09
ENSRNOG00000056226	-0.991871311	1.264983659	39.13496905	3.95E-10	6.34E-09
ENSRNOG00000019223	-0.367983127	7.797381319	39.11010626	4.01E-10	6.42E-09
ENSRNOG00000008279	-0.521061283	5.278293796	39.06047467	4.11E-10	6.58E-09
ENSRNOG00000008859	-0.491826517	4.278013536	39.04744783	4.14E-10	6.62E-09
ENSRNOG00000047978	-1.299583601	0.790086495	39.01909056	4.20E-10	6.69E-09
ENSRNOG00000057204	-0.862682904	1.852752795	38.85571839	4.56E-10	7.23E-09
ENSRNOG00000034084	-0.661867127	2.967721755	38.79977297	4.70E-10	7.44E-09
ENSRNOG00000000814	-0.775591525	5.647785537	38.68901249	4.97E-10	7.85E-09
ENSRNOG00000013892	-0.518942092	4.42998701	38.68386346	4.98E-10	7.85E-09
ENSRNOG00000002452	-0.449249593	4.802911209	38.68421571	4.98E-10	7.85E-09
ENSRNOG00000003139	-0.493648932	7.009329156	38.62087071	5.15E-10	8.09E-09
ENSRNOG00000019983	-0.359427564	5.370059121	38.58127602	5.25E-10	8.23E-09
ENSRNOG00000050552	-0.812075264	2.748875931	38.4933478	5.49E-10	8.56E-09
ENSRNOG00000029152	-0.531891231	4.761190072	38.49303373	5.49E-10	8.56E-09
ENSRNOG00000059041	-0.553390428	3.704610927	38.41765224	5.71E-10	8.86E-09
ENSRNOG00000000524	-0.40538115	5.914172155	38.40928928	5.74E-10	8.89E-09
ENSRNOG00000024779	-0.448792354	6.481705332	38.29354297	6.09E-10	9.39E-09
ENSRNOG00000059466	-1.012207968	0.529401279	38.19585002	6.40E-10	9.84E-09
ENSRNOG00000014107	-0.656827778	7.700200285	38.14249216	6.58E-10	1.01E-08
ENSRNOG00000006050	-0.475252472	4.994605003	38.10710086	6.70E-10	1.03E-08
ENSRNOG00000004526	-0.513092766	7.380939285	38.07621726	6.80E-10	1.04E-08
ENSRNOG00000021379	-0.553010366	4.505072695	38.03363009	6.95E-10	1.06E-08
ENSRNOG00000028883	-1.249689084	1.01558243	38.01449717	7.02E-10	1.07E-08
ENSRNOG00000024530	-0.509109069	3.740117159	37.9946354	7.09E-10	1.08E-08
ENSRNOG00000007341	-0.62942697	3.404290369	37.98317554	7.14E-10	1.09E-08
ENSRNOG00000052831	-1.0833578	3.237100591	37.82101827	7.75E-10	1.18E-08
ENSRNOG00000012679	-0.491023407	3.44349677	37.75822579	8.01E-10	1.21E-08
ENSRNOG00000016486	-0.476749992	5.733208709	37.67076773	8.38E-10	1.27E-08
ENSRNOG00000002835	-0.5538379	7.581667316	37.66622581	8.39E-10	1.27E-08
ENSRNOG00000019159	-0.445109293	5.499204052	37.6202149	8.59E-10	1.29E-08
ENSRNOG00000042037	-0.416114355	6.139222755	37.53188891	8.99E-10	1.35E-08
ENSRNOG00000037540	-0.639415386	3.639729017	37.50230684	9.13E-10	1.37E-08
ENSRNOG00000026569	-0.539430954	3.907299028	37.19183642	1.07E-09	1.59E-08
ENSRNOG00000022868	-0.748903152	2.976910685	37.18832707	1.07E-09	1.59E-08
ENSRNOG00000013536	-0.406791513	7.655156677	37.15285443	1.09E-09	1.61E-08
ENSRNOG00000009993	-0.449914022	4.108801575	37.00865156	1.18E-09	1.73E-08
ENSRNOG00000013609	-0.816400313	2.139615356	36.81799805	1.30E-09	1.90E-08
ENSRNOG00000005825	-0.695247002	5.620495615	36.80427249	1.31E-09	1.91E-08
ENSRNOG00000047931	-0.539962866	9.575911041	36.80204617	1.31E-09	1.91E-08
ENSRNOG00000004681	-0.373236712	6.088273905	36.6846263	1.39E-09	2.02E-08
ENSRNOG00000032350	-0.607421242	6.4780766	36.66207569	1.40E-09	2.05E-08
ENSRNOG00000028449	-1.812693444	-0.895449685	36.50945218	1.52E-09	2.20E-08
ENSRNOG00000005902	-0.663716889	3.604896191	36.39300574	1.61E-09	2.33E-08
ENSRNOG00000030449	-0.693892005	4.514054645	36.32615268	1.67E-09	2.40E-08
ENSRNOG00000001108	-0.51604716	4.953447599	36.28049583	1.71E-09	2.45E-08
ENSRNOG00000018044	-0.446240599	6.224413524	36.27253445	1.72E-09	2.46E-08
ENSRNOG00000006762	-0.727030827	5.192867277	36.19323728	1.79E-09	2.56E-08
ENSRNOG00000003809	-0.65271714	6.325161583	36.17540469	1.80E-09	2.57E-08
ENSRNOG00000012124	-0.393808712	6.224005241	36.10444265	1.87E-09	2.66E-08
ENSRNOG00000011376	-0.494251246	4.677106213	36.10104311	1.87E-09	2.66E-08
ENSRNOG00000029813	-0.878170241	5.164485855	36.06645394	1.91E-09	2.71E-08
ENSRNOG00000052910	-1.508302423	-0.773071624	36.03720028	1.94E-09	2.74E-08
ENSRNOG00000026636	-0.674267327	4.952398701	35.99709388	1.98E-09	2.79E-08
ENSRNOG00000003773	-0.467386288	6.087781976	35.99461814	1.98E-09	2.79E-08
ENSRNOG00000009437	-1.320398973	1.541933129	35.97221344	2.00E-09	2.82E-08
ENSRNOG00000004035	-0.616283929	4.371114972	35.96652275	2.01E-09	2.83E-08
ENSRNOG00000003211	-0.416895105	5.251547408	35.93876161	2.04E-09	2.87E-08

ENSRNOG00000013719	-0.568858976	3.888523034	35.90943343	2.07E-09	2.90E-08
ENSRNOG00000012089	-0.40741413	4.696986778	35.84782332	2.13E-09	2.98E-08
ENSRNOG00000047118	-0.522654624	4.083402674	35.75473517	2.24E-09	3.11E-08
ENSRNOG00000010728	-0.404961307	4.978047443	35.6965937	2.31E-09	3.20E-08
ENSRNOG00000003506	-0.413098541	6.588234906	35.69359882	2.31E-09	3.20E-08
ENSRNOG00000032780	-0.422142963	4.256628433	35.64614281	2.37E-09	3.27E-08
ENSRNOG00000004784	-0.388576089	5.066901171	35.56486092	2.47E-09	3.40E-08
ENSRNOG00000048682	-0.505324898	11.5972008	35.42701509	2.65E-09	3.63E-08
ENSRNOG00000022871	-0.452593687	4.190864794	35.30892832	2.81E-09	3.83E-08
ENSRNOG00000017251	-0.4352734	6.580505923	35.30957612	2.81E-09	3.83E-08
ENSRNOG00000004301	-0.460002936	4.637745653	35.22981079	2.93E-09	3.97E-08
ENSRNOG00000059386	-0.52065929	3.484608762	35.21552203	2.95E-09	3.99E-08
ENSRNOG00000016935	-0.429004455	5.058804867	35.05496585	3.21E-09	4.32E-08
ENSRNOG00000060069	-0.564764751	4.501093002	34.96597923	3.36E-09	4.50E-08
ENSRNOG00000009290	-0.475631706	4.39903793	34.88266456	3.50E-09	4.68E-08
ENSRNOG00000055118	-1.472559051	-0.762618432	34.86393531	3.54E-09	4.72E-08
ENSRNOG00000042201	-0.81757346	2.152850391	34.83238248	3.59E-09	4.80E-08
ENSRNOG00000002871	-0.785173692	5.55110912	34.75446319	3.74E-09	4.99E-08
ENSRNOG00000006789	-0.603773335	4.884036595	34.74272056	3.76E-09	5.01E-08
ENSRNOG00000002989	-0.473281512	6.566896513	34.73999963	3.77E-09	5.01E-08
ENSRNOG00000029078	-0.825038218	1.914438007	34.73808227	3.77E-09	5.01E-08
ENSRNOG00000056325	-0.381638671	5.693350288	34.70300796	3.84E-09	5.09E-08
ENSRNOG00000042160	-0.744571652	3.929122589	34.62689667	3.99E-09	5.28E-08
ENSRNOG00000007629	-0.490934511	5.69102575	34.57680154	4.10E-09	5.41E-08
ENSRNOG00000035613	-1.624284172	-0.476903849	34.5778776	4.10E-09	5.41E-08
ENSRNOG00000047106	-0.415000343	4.259120629	34.53742405	4.18E-09	5.51E-08
ENSRNOG00000049912	-0.7290044	6.536418216	34.46094201	4.35E-09	5.71E-08
ENSRNOG00000048989	-0.388820922	4.930052803	34.4176268	4.45E-09	5.83E-08
ENSRNOG00000012046	-0.496534874	6.018198054	34.40461834	4.48E-09	5.86E-08
ENSRNOG00000014415	-0.373283085	8.061608161	34.36700141	4.56E-09	5.97E-08
ENSRNOG00000009982	-0.464405478	6.556656725	34.34021663	4.63E-09	6.05E-08
ENSRNOG00000008410	-0.537429983	4.503627964	34.33331095	4.64E-09	6.06E-08
ENSRNOG00000043328	-0.39512596	5.901415142	34.32094869	4.67E-09	6.10E-08
ENSRNOG00000008329	-0.34921902	5.7871377	34.31025626	4.70E-09	6.12E-08
ENSRNOG00000004727	-0.454595653	3.179159606	34.29463374	4.74E-09	6.16E-08
ENSRNOG00000051606	-0.572447199	4.211967857	34.27060388	4.80E-09	6.22E-08
ENSRNOG00000009742	-0.482722469	3.637960408	34.21522022	4.93E-09	6.38E-08
ENSRNOG00000003634	-0.552419755	3.485649079	34.14210054	5.12E-09	6.62E-08
ENSRNOG00000033545	-1.944491274	7.648908148	34.12368058	5.17E-09	6.67E-08
ENSRNOG00000062247	-0.550951364	3.009507985	34.10445201	5.22E-09	6.73E-08
ENSRNOG00000009245	-0.406219967	4.689958519	34.09398287	5.25E-09	6.77E-08
ENSRNOG00000017279	-0.367174369	6.171974679	34.01350005	5.47E-09	7.04E-08
ENSRNOG00000001628	-1.490047447	8.465225081	33.9926224	5.53E-09	7.11E-08
ENSRNOG00000011260	-0.624589927	4.79480795	33.97685514	5.58E-09	7.16E-08
ENSRNOG00000016733	-0.617832766	3.807733659	33.8713556	5.89E-09	7.53E-08
ENSRNOG00000009734	-0.687787414	2.285539527	33.87136657	5.89E-09	7.53E-08
ENSRNOG00000008309	-0.744335182	3.079513866	33.74648889	6.28E-09	7.99E-08
ENSRNOG00000006818	-0.387636865	6.72728826	33.71580448	6.38E-09	8.11E-08
ENSRNOG00000011413	-0.396559558	7.911506585	33.68908248	6.47E-09	8.20E-08
ENSRNOG00000018937	-0.677114651	7.683896052	33.67564802	6.51E-09	8.25E-08
ENSRNOG00000000891	-0.521673859	4.170160719	33.66639449	6.54E-09	8.28E-08
ENSRNOG00000011661	-0.466034997	7.006905829	33.66146919	6.56E-09	8.29E-08
ENSRNOG00000056374	-0.612435513	3.882229416	33.65550186	6.58E-09	8.31E-08
ENSRNOG00000015799	-1.030973748	1.111129915	33.54448901	6.97E-09	8.77E-08
ENSRNOG00000019934	-0.437250942	7.259449094	33.49597829	7.14E-09	8.97E-08
ENSRNOG00000036835	-0.422252246	6.725526241	33.48765889	7.17E-09	9.01E-08
ENSRNOG00000020201	-0.480644614	3.846383308	33.39995555	7.50E-09	9.35E-08
ENSRNOG00000039740	-0.716703902	2.027991475	33.35350611	7.68E-09	9.57E-08
ENSRNOG00000046353	-0.657255725	2.845313918	33.07910731	8.85E-09	1.10E-07
ENSRNOG00000004842	-0.65515819	3.280243276	33.05984612	8.94E-09	1.10E-07
ENSRNOG00000019572	-0.434878838	4.961439277	33.04916977	8.99E-09	1.11E-07
ENSRNOG00000012486	-0.639457626	2.924361082	33.04027238	9.03E-09	1.11E-07
ENSRNOG00000021092	-0.484159675	3.324706851	33.0340665	9.06E-09	1.12E-07
ENSRNOG00000001161	-0.520628677	5.236259788	32.99637965	9.23E-09	1.14E-07
ENSRNOG00000003706	-0.336357455	5.779688434	32.97946362	9.31E-09	1.14E-07
ENSRNOG00000010420	-0.5589278	5.824694496	32.96213206	9.40E-09	1.15E-07
ENSRNOG00000009934	-0.443622287	6.634853804	32.95118402	9.45E-09	1.16E-07
ENSRNOG00000015844	-0.581455823	5.217018027	32.94323711	9.49E-09	1.16E-07
ENSRNOG00000018296	-0.526958116	4.199631315	32.91369219	9.63E-09	1.18E-07
ENSRNOG00000007542	-0.392961215	8.40346933	32.83349538	1.00E-08	1.22E-07
ENSRNOG00000025141	-0.602267845	3.691809512	32.70776729	1.07E-08	1.30E-07
ENSRNOG00000020646	-0.374606132	6.118142091	32.70511992	1.07E-08	1.30E-07
ENSRNOG00000012227	-0.683075033	4.499390525	32.70143696	1.07E-08	1.30E-07
ENSRNOG00000008891	-0.380436411	5.169893518	32.62808964	1.12E-08	1.35E-07
ENSRNOG00000017194	-0.53090106	6.876861807	32.5541555	1.16E-08	1.40E-07
ENSRNOG00000023299	-0.624491199	3.552340039	32.54774191	1.16E-08	1.40E-07
ENSRNOG00000015211	-0.730879763	1.70459917	32.50263751	1.19E-08	1.43E-07
ENSRNOG00000004322	-0.455564806	6.154570739	32.46588552	1.21E-08	1.46E-07
ENSRNOG00000010186	-0.34910131	4.869971782	32.42218636	1.24E-08	1.48E-07
ENSRNOG00000025701	-0.458559911	5.114922533	32.38855369	1.26E-08	1.51E-07
ENSRNOG00000005377	-0.476645652	4.287177236	32.31973474	1.31E-08	1.56E-07
ENSRNOG00000016327	-0.399883171	3.979878813	32.28353479	1.33E-08	1.58E-07
ENSRNOG00000056397	-0.719884677	1.835289153	32.2742817	1.34E-08	1.59E-07
ENSRNOG00000049939	-0.675321513	4.402549578	32.2616901	1.35E-08	1.60E-07
ENSRNOG00000014331	-0.383818688	6.490707861	32.20781329	1.39E-08	1.64E-07
ENSRNOG00000036813	-0.503154156	6.678685361	32.19814549	1.39E-08	1.65E-07
ENSRNOG00000006913	-0.462377678	3.308805055	32.19695277	1.39E-08	1.65E-07
ENSRNOG00000001598	-0.401254128	5.061642534	32.16325511	1.42E-08	1.67E-07
ENSRNOG00000008628	-0.390719853	5.176685033	32.15580245	1.42E-08	1.68E-07
ENSRNOG00000046639	-0.731035525	2.278827369	32.10148517	1.46E-08	1.72E-07
ENSRNOG00000008605	-0.767522817	5.700998063	32.06125778	1.49E-08	1.76E-07
ENSRNOG00000057835	-0.76139302	2.946204331	32.04587227	1.51E-08	1.77E-07
ENSRNOG00000012358	-0.353169266	4.950782198	32.04375531	1.51E-08	1.77E-07
ENSRNOG00000012087	-0.513472768	5.584470096	32.0143957	1.53E-08	1.79E-07
ENSRNOG00000027857	-0.548392299	3.468299746	31.93124379	1.60E-08	1.87E-07
ENSRNOG00000031579	-0.734262531	1.774951244	31.92102514	1.61E-08	1.88E-07
ENSRNOG00000003980	-0.419451174	5.743598686	31.88114971	1.64E-08	1.91E-07
ENSRNOG00000000064	-0.538350655	6.432187468	31.8622923	1.65E-08	1.93E-07
ENSRNOG00000056631	-0.534374341	5.876069825	31.80332395	1.71E-08	1.99E-07
ENSRNOG00000015142	-0.584578539	4.580785928	31.78751963	1.72E-08	2.00E-07

ENSRNOG00000002407	-0.377393567	4.623249227	31.77015577	1.74E-08	2.02E-07
ENSRNOG00000046383	-0.982877176	1.865841005	31.76775305	1.74E-08	2.02E-07
ENSRNOG00000008101	-0.572273985	2.747313279	31.73361302	1.77E-08	2.05E-07
ENSRNOG00000008465	-0.559981681	6.121353118	31.62259236	1.87E-08	2.17E-07
ENSRNOG00000006871	-0.369647469	5.37315977	31.59754326	1.90E-08	2.19E-07
ENSRNOG00000010301	-0.471037874	3.540364106	31.56366397	1.93E-08	2.23E-07
ENSRNOG00000007040	-0.461481965	5.580527818	31.55263417	1.94E-08	2.24E-07
ENSRNOG00000006221	-0.349933494	6.389950198	31.5439382	1.95E-08	2.25E-07
ENSRNOG00000019059	-0.637501218	3.486149641	31.50608164	1.99E-08	2.29E-07
ENSRNOG00000060308	-0.801411258	1.502991477	31.49349027	2.00E-08	2.30E-07
ENSRNOG00000057814	-0.429869318	5.011908503	31.45915578	2.04E-08	2.34E-07
ENSRNOG00000011052	-0.381556097	6.497379621	31.43680194	2.06E-08	2.36E-07
ENSRNOG00000037352	-0.415674801	3.998312028	31.29586358	2.22E-08	2.53E-07
ENSRNOG00000061301	-0.832361771	2.764984978	31.28001752	2.23E-08	2.54E-07
ENSRNOG00000024705	-0.621870923	4.098239565	31.15427049	2.38E-08	2.70E-07
ENSRNOG00000026299	-0.434765363	4.854834682	31.08608406	2.47E-08	2.79E-07
ENSRNOG00000003928	-0.388008335	4.60433954	31.08655045	2.47E-08	2.79E-07
ENSRNOG00000036660	-0.350615648	5.904085882	31.03411276	2.54E-08	2.86E-07
ENSRNOG00000011214	-0.47530532	4.831011807	31.0308002	2.54E-08	2.86E-07
ENSRNOG00000032192	-0.471475785	4.057742781	31.01360917	2.56E-08	2.88E-07
ENSRNOG00000016105	-0.414293756	4.675946437	30.97190287	2.62E-08	2.94E-07
ENSRNOG00000018920	-0.312803165	6.215446673	30.8545765	2.78E-08	3.12E-07
ENSRNOG00000046629	-0.796362156	1.134013633	30.82883897	2.82E-08	3.16E-07
ENSRNOG00000011639	-0.4003724	4.632548712	30.78715612	2.88E-08	3.22E-07
ENSRNOG00000051136	-0.80418326	1.186755915	30.78491606	2.88E-08	3.22E-07
ENSRNOG00000036659	-0.472972819	4.308205583	30.65943298	3.08E-08	3.42E-07
ENSRNOG00000029726	-0.600325172	7.555661678	30.45523828	3.42E-08	3.78E-07
ENSRNOG00000012994	-0.51386176	3.800450471	30.41564028	3.49E-08	3.85E-07
ENSRNOG00000010783	-0.440446355	4.685076815	30.32333447	3.66E-08	4.02E-07
ENSRNOG00000001996	-0.410344534	6.492493963	30.30599439	3.69E-08	4.05E-07
ENSRNOG00000008782	-0.438000597	7.551375263	30.25787097	3.78E-08	4.14E-07
ENSRNOG00000020463	-0.466594668	4.671339398	30.21249788	3.87E-08	4.23E-07
ENSRNOG00000010512	-0.48873131	4.963724564	30.08887969	4.13E-08	4.50E-07
ENSRNOG00000012654	-0.369908169	4.387913253	30.06953573	4.17E-08	4.55E-07
ENSRNOG00000036742	-0.379988846	8.201525355	30.04649466	4.22E-08	4.59E-07
ENSRNOG00000006884	-1.115778296	0.78210092	30.00958659	4.30E-08	4.67E-07
ENSRNOG00000007117	-0.322375441	5.753646826	29.95455946	4.42E-08	4.79E-07
ENSRNOG00000010320	-0.461427976	7.391101737	29.89592544	4.56E-08	4.93E-07
ENSRNOG00000019464	-0.322357546	5.845216123	29.87132797	4.62E-08	4.98E-07
ENSRNOG00000047456	-0.497869572	5.050311345	29.86423804	4.63E-08	5.00E-07
ENSRNOG00000018719	-0.444905485	5.384675525	29.86026026	4.64E-08	5.00E-07
ENSRNOG00000048342	-0.5262	5.509270323	29.81533287	4.75E-08	5.11E-07
ENSRNOG00000005856	-0.404449623	3.538783157	29.80278046	4.78E-08	5.13E-07
ENSRNOG00000012576	-0.399245182	5.18985703	29.77794419	4.84E-08	5.19E-07
ENSRNOG00000019634	-0.441495402	4.390483912	29.69914536	5.05E-08	5.39E-07
ENSRNOG00000003901	-0.419183779	7.041502959	29.66809351	5.13E-08	5.47E-07
ENSRNOG00000004829	-0.701089474	4.825597517	29.65479888	5.16E-08	5.49E-07
ENSRNOG00000012584	-0.585583211	2.767657531	29.64260176	5.20E-08	5.52E-07
ENSRNOG00000059593	-0.476801428	6.231437252	29.64133071	5.20E-08	5.52E-07
ENSRNOG00000060853	-0.384957605	4.382846855	29.61736085	5.26E-08	5.59E-07
ENSRNOG00000009063	-0.490696995	4.559150695	29.57122049	5.39E-08	5.71E-07
ENSRNOG00000055598	-0.801047166	2.765418061	29.55922763	5.42E-08	5.74E-07
ENSRNOG00000028822	-0.334654492	7.029776375	29.490244	5.62E-08	5.91E-07
ENSRNOG00000028884	-0.495898747	7.468758178	29.43601176	5.78E-08	6.07E-07
ENSRNOG00000015109	-0.426717988	4.212323355	29.42370255	5.82E-08	6.10E-07
ENSRNOG00000015660	-0.373046045	4.755476576	29.36463947	6.00E-08	6.27E-07
ENSRNOG00000030247	-0.397992683	6.211189517	29.34389055	6.06E-08	6.33E-07
ENSRNOG00000004776	-0.471951772	3.409790856	29.10520231	6.86E-08	7.11E-07
ENSRNOG00000058283	-0.84806934	1.574675099	29.00832041	7.21E-08	7.45E-07
ENSRNOG00000042869	-0.70108638	3.802145321	29.00538565	7.22E-08	7.46E-07
ENSRNOG00000011276	-0.506854978	4.173273482	28.99632631	7.25E-08	7.49E-07
ENSRNOG00000013110	-0.489076125	4.749778288	28.86128027	7.78E-08	7.96E-07
ENSRNOG00000007267	-0.424524362	4.14230542	28.861292	7.78E-08	7.96E-07
ENSRNOG00000014003	-0.6417312	3.367282404	28.79696858	8.04E-08	8.20E-07
ENSRNOG00000001603	-0.671324376	2.493093417	28.79129152	8.06E-08	8.21E-07
ENSRNOG00000004001	-0.4661103	3.705864265	28.75216471	8.23E-08	8.38E-07
ENSRNOG00000017741	-0.463596258	3.429192203	28.72602819	8.34E-08	8.48E-07
ENSRNOG00000005260	-0.585930188	4.81911032	28.69327001	8.48E-08	8.62E-07
ENSRNOG00000053984	-1.056269518	0.6953356	28.66968834	8.58E-08	8.71E-07
ENSRNOG00000003081	-0.447250392	5.260128025	28.66661784	8.60E-08	8.72E-07
ENSRNOG00000016973	-0.35221447	3.965332167	28.66133688	8.62E-08	8.73E-07
ENSRNOG00000017781	-0.387719291	7.318345895	28.65884376	8.63E-08	8.73E-07
ENSRNOG00000015088	-0.492213872	5.662885001	28.63235011	8.75E-08	8.84E-07
ENSRNOG00000046414	-0.936661593	0.339213628	28.63278294	8.75E-08	8.84E-07
ENSRNOG00000003879	-0.471809815	6.322486513	28.60271327	8.89E-08	8.97E-07
ENSRNOG00000019023	-0.325959804	5.386936787	28.48923303	9.42E-08	9.47E-07
ENSRNOG00000033593	-0.397694268	5.900919411	28.48985325	9.42E-08	9.47E-07
ENSRNOG00000004509	-0.436655361	5.318137436	28.4421752	9.65E-08	9.69E-07
ENSRNOG00000050729	-0.551586429	2.016471668	28.42951735	9.72E-08	9.74E-07
ENSRNOG00000003049	-0.487684174	5.181757385	28.40004077	9.87E-08	9.88E-07
ENSRNOG00000026646	-0.435171673	5.412255394	28.39477417	9.89E-08	9.89E-07
ENSRNOG00000021062	-1.101976961	3.64808721	28.39012136	9.92E-08	9.91E-07
ENSRNOG00000006636	-0.474550926	5.959538281	28.35927915	1.01E-07	1.00E-06
ENSRNOG00000053438	-0.821296141	0.650102811	28.34008757	1.02E-07	1.01E-06
ENSRNOG00000019616	-0.562451418	2.497927789	28.33573148	1.02E-07	1.01E-06
ENSRNOG00000054770	-0.332000837	5.331406226	28.33623449	1.02E-07	1.01E-06
ENSRNOG00000058510	-1.753726438	-0.946038975	28.33117416	1.02E-07	1.02E-06
ENSRNOG00000037124	-0.69878986	1.085420229	28.32343608	1.03E-07	1.02E-06
ENSRNOG00000005249	-0.377110864	5.505635384	28.29208137	1.04E-07	1.03E-06
ENSRNOG00000023363	-0.354426996	5.410180953	28.25239306	1.06E-07	1.05E-06
ENSRNOG00000015428	-0.447003744	5.011454981	28.2398887	1.07E-07	1.06E-06
ENSRNOG00000053811	-0.360283642	4.311406835	28.14457126	1.13E-07	1.11E-06
ENSRNOG00000051792	-0.745008546	1.338554997	28.11008724	1.15E-07	1.13E-06
ENSRNOG00000031333	-0.808388131	7.272097364	28.04622549	1.18E-07	1.16E-06
ENSRNOG00000007673	-0.401814326	6.598512157	27.9548199	1.24E-07	1.21E-06
ENSRNOG00000001040	-0.315758299	4.554235861	27.95480508	1.24E-07	1.21E-06
ENSRNOG00000007546	-0.3580257	5.53836596	27.95122061	1.24E-07	1.21E-06
ENSRNOG00000008607	-0.393228797	6.165353188	27.92916532	1.26E-07	1.23E-06
ENSRNOG00000000926	-0.470923084	4.396665546	27.87321872	1.30E-07	1.26E-06
ENSRNOG00000012121	-0.347253036	6.029813622	27.87053525	1.30E-07	1.26E-06
ENSRNOG00000037146	-0.750976013	3.961674496	27.77669165	1.36E-07	1.32E-06

ENSRNOG00000016701	-1.103602119	3.328619884	27.74077374	1.39E-07	1.34E-06
ENSRNOG00000022144	-0.73085287	2.33869677	27.69957347	1.42E-07	1.37E-06
ENSRNOG00000007371	-0.472125574	3.426063919	27.69745158	1.42E-07	1.37E-06
ENSRNOG00000031576	-0.60514335	2.348750151	27.68193924	1.43E-07	1.38E-06
ENSRNOG00000018547	-0.385154856	4.375416324	27.67558393	1.43E-07	1.39E-06
ENSRNOG00000047198	-0.459815555	3.647443857	27.67440368	1.44E-07	1.39E-06
ENSRNOG00000012035	-0.308212725	5.845853396	27.65096519	1.45E-07	1.40E-06
ENSRNOG00000019069	-0.452057576	6.837671932	27.6444739	1.46E-07	1.40E-06
ENSRNOG00000018849	-0.375515019	6.758646459	27.60779468	1.49E-07	1.43E-06
ENSRNOG00000060360	-1.022846163	0.540451829	27.51293757	1.56E-07	1.50E-06
ENSRNOG00000009266	-0.534210244	6.518042408	27.50446681	1.57E-07	1.50E-06
ENSRNOG00000026716	-0.564942627	8.378411677	27.4603614	1.60E-07	1.53E-06
ENSRNOG00000015867	-0.963926646	0.484767237	27.44079884	1.62E-07	1.55E-06
ENSRNOG00000027175	-0.438680444	3.620973192	27.40697001	1.65E-07	1.57E-06
ENSRNOG00000057745	-1.204679	0.105728178	27.36196203	1.69E-07	1.60E-06
ENSRNOG00000057416	-0.446889945	3.854558122	27.33837562	1.71E-07	1.62E-06
ENSRNOG00000003151	-0.328959747	5.32331901	27.3322088	1.71E-07	1.62E-06
ENSRNOG00000020487	-0.357070891	5.121708896	27.32262095	1.72E-07	1.63E-06
ENSRNOG00000046390	-0.512814519	5.34853028	27.30680024	1.74E-07	1.64E-06
ENSRNOG00000000586	-0.346614606	4.700840861	27.27756646	1.76E-07	1.66E-06
ENSRNOG00000014720	-0.428312588	4.407766769	27.22059999	1.82E-07	1.71E-06
ENSRNOG00000023322	-0.484154083	5.242692307	27.20814564	1.83E-07	1.72E-06
ENSRNOG00000028517	-0.776324996	2.954140583	27.17339928	1.86E-07	1.75E-06
ENSRNOG00000000840	-0.5342636	8.470334384	27.14705215	1.89E-07	1.77E-06
ENSRNOG00000004364	-0.371958041	5.280789289	27.12968331	1.90E-07	1.78E-06
ENSRNOG00000059319	-0.480048343	2.997309079	27.10511781	1.93E-07	1.80E-06
ENSRNOG00000024043	-0.485609506	2.771561587	27.08375295	1.95E-07	1.82E-06
ENSRNOG00000033932	-0.824946117	7.770189669	27.06331764	1.97E-07	1.84E-06
ENSRNOG00000012099	-0.452066633	4.48256001	26.99741703	2.04E-07	1.90E-06
ENSRNOG00000019047	-0.353705338	6.556983799	26.97148575	2.06E-07	1.92E-06
ENSRNOG00000016905	-0.310591892	6.518145899	26.97191225	2.06E-07	1.92E-06
ENSRNOG00000035631	-1.045851632	-0.453480039	26.96271721	2.07E-07	1.93E-06
ENSRNOG00000050490	-0.694097379	4.824472033	26.94667898	2.09E-07	1.94E-06
ENSRNOG00000007862	-0.324040557	6.188895197	26.92081473	2.12E-07	1.97E-06
ENSRNOG00000013738	-0.386365203	3.669723419	26.91673113	2.12E-07	1.97E-06
ENSRNOG00000028461	-0.393618737	5.218456791	26.91538995	2.13E-07	1.97E-06
ENSRNOG00000052394	-0.914594869	0.973171101	26.90973739	2.13E-07	1.98E-06
ENSRNOG00000039877	-0.456205138	3.472654128	26.9064125	2.14E-07	1.98E-06
ENSRNOG00000002050	-0.739088288	5.630931928	26.89735897	2.15E-07	1.99E-06
ENSRNOG00000020460	-0.657003844	5.846493828	26.85992727	2.19E-07	2.02E-06
ENSRNOG00000059106	-1.506627107	0.892480479	26.79932487	2.26E-07	2.07E-06
ENSRNOG00000016266	-0.403877956	4.19707929	26.7832223	2.28E-07	2.09E-06
ENSRNOG00000004883	-0.341841259	5.953133246	26.77494783	2.29E-07	2.09E-06
ENSRNOG00000016507	-0.481593475	3.622491326	26.73295437	2.34E-07	2.14E-06
ENSRNOG00000024159	-1.041054737	4.375549727	26.71584603	2.36E-07	2.15E-06
ENSRNOG00000054850	-1.349016219	0.945792921	26.68655764	2.39E-07	2.18E-06
ENSRNOG00000007356	-0.52920599	6.027766795	26.6740662	2.41E-07	2.19E-06
ENSRNOG00000016468	-0.699504637	3.249232818	26.65306889	2.43E-07	2.21E-06
ENSRNOG00000016338	-0.377658933	5.042866182	26.64897196	2.44E-07	2.22E-06
ENSRNOG00000012557	-1.257608549	0.273439792	26.647929	2.44E-07	2.22E-06
ENSRNOG00000049223	-0.302869794	5.887578469	26.639097	2.45E-07	2.23E-06
ENSRNOG00000009397	-0.325462069	5.651056589	26.6085799	2.49E-07	2.26E-06
ENSRNOG00000059558	-1.01975116	0.541348013	26.60684415	2.49E-07	2.26E-06
ENSRNOG00000019570	-0.539902401	6.854541716	26.58612795	2.52E-07	2.28E-06
ENSRNOG00000054194	-0.947316487	0.936106322	26.5737948	2.54E-07	2.30E-06
ENSRNOG00000018718	-0.445435095	4.409577199	26.55899048	2.56E-07	2.31E-06
ENSRNOG00000047145	-0.396977954	3.767205276	26.53955652	2.58E-07	2.33E-06
ENSRNOG00000006326	-0.450251718	4.578429738	26.529295	2.60E-07	2.34E-06
ENSRNOG00000006973	-0.385007995	5.132688615	26.51226402	2.62E-07	2.36E-06
ENSRNOG00000010184	-0.410023219	7.150173332	26.45147741	2.70E-07	2.43E-06
ENSRNOG00000062308	-0.680932465	3.316805748	26.41138089	2.76E-07	2.48E-06
ENSRNOG00000046662	-0.485286212	4.60224533	26.39512366	2.78E-07	2.50E-06
ENSRNOG00000043426	-0.510802578	3.30547889	26.35615065	2.84E-07	2.55E-06
ENSRNOG00000025025	-0.353349058	5.219145684	26.33613613	2.87E-07	2.57E-06
ENSRNOG00000032609	-0.806964107	8.06095391	26.31524171	2.90E-07	2.59E-06
ENSRNOG00000047132	-0.403284632	4.264534791	26.29333026	2.93E-07	2.62E-06
ENSRNOG00000024563	-0.403761616	4.443097202	26.27967858	2.95E-07	2.64E-06
ENSRNOG00000031378	-1.095102216	-0.050165009	26.26227883	2.98E-07	2.66E-06
ENSRNOG00000011853	-0.480272352	5.323123382	26.20609917	3.07E-07	2.73E-06
ENSRNOG00000003068	-0.839474865	4.656063576	26.19714033	3.08E-07	2.74E-06
ENSRNOG00000018048	-0.296461603	5.882680438	26.19312075	3.09E-07	2.75E-06
ENSRNOG00000007739	-0.347409475	5.291444287	26.13692218	3.18E-07	2.83E-06
ENSRNOG00000020693	-0.477202833	3.30709281	26.09345502	3.25E-07	2.88E-06
ENSRNOG00000047526	-0.514868046	4.454465346	26.08757387	3.26E-07	2.89E-06
ENSRNOG00000021153	-0.603549111	6.332396306	26.07941476	3.28E-07	2.90E-06
ENSRNOG00000013505	-0.47844167	7.758196629	26.02152588	3.38E-07	2.98E-06
ENSRNOG00000014816	-0.412718012	6.545394282	25.98168041	3.45E-07	3.05E-06
ENSRNOG00000045696	-0.564427044	2.586056014	25.93689798	3.53E-07	3.11E-06
ENSRNOG00000009746	-0.389381906	5.36497478	25.92962344	3.54E-07	3.12E-06
ENSRNOG00000022894	-0.37852096	4.312245648	25.81944177	3.75E-07	3.28E-06
ENSRNOG00000053659	-0.294094421	6.462890555	25.80986938	3.77E-07	3.29E-06
ENSRNOG00000046983	-0.786438954	1.915703616	25.80744871	3.77E-07	3.29E-06
ENSRNOG00000004474	-0.337935255	6.728429074	25.7633179	3.86E-07	3.36E-06
ENSRNOG00000001991	-0.432510546	7.247548723	25.76073204	3.86E-07	3.37E-06
ENSRNOG00000014188	-0.740526638	1.878254291	25.7418598	3.90E-07	3.40E-06
ENSRNOG00000052002	-0.541927386	3.197206495	25.71332953	3.96E-07	3.44E-06
ENSRNOG00000001035	-0.425324006	4.117000806	25.65351218	4.09E-07	3.54E-06
ENSRNOG00000015501	-0.382437091	6.04458997	25.63965831	4.11E-07	3.57E-06
ENSRNOG00000021289	-0.328825209	5.774918415	25.59435059	4.21E-07	3.65E-06
ENSRNOG00000058856	-1.462742527	0.428246161	25.59498325	4.21E-07	3.65E-06
ENSRNOG00000050024	-1.214699404	-0.950770909	25.59292428	4.22E-07	3.65E-06
ENSRNOG00000018994	-0.329296002	6.083130461	25.53308171	4.35E-07	3.76E-06
ENSRNOG00000009478	-0.468955047	4.662737471	25.52278735	4.37E-07	3.77E-06
ENSRNOG00000035499	-1.16400303	0.442226246	25.51343438	4.39E-07	3.79E-06
ENSRNOG00000000246	-0.313428681	7.039537457	25.48238369	4.46E-07	3.85E-06
ENSRNOG00000000866	-0.411026947	5.518002741	25.46136292	4.51E-07	3.88E-06
ENSRNOG00000012860	-0.31540574	5.650732739	25.4376262	4.57E-07	3.93E-06
ENSRNOG00000012651	-0.39818028	4.518392861	25.42679165	4.59E-07	3.95E-06
ENSRNOG00000018593	-0.416624013	5.594828838	25.38621349	4.69E-07	4.03E-06
ENSRNOG00000008075	-0.421920727	4.530187916	25.37570505	4.72E-07	4.04E-06

ENSRNOG00000005437	-0.466274396	5.303154465	25.30313842	4.90E-07	4.19E-06
ENSRNOG00000005360	-0.338730102	4.435330764	25.29463452	4.92E-07	4.21E-06
ENSRNOG00000011540	-0.351645727	5.754455651	25.28121116	4.96E-07	4.24E-06
ENSRNOG00000042276	-0.402921965	6.113270784	25.27663329	4.97E-07	4.24E-06
ENSRNOG00000020216	-0.340372718	5.221637734	25.25161547	5.03E-07	4.30E-06
ENSRNOG00000009382	-0.544447496	2.759853231	25.23666686	5.07E-07	4.33E-06
ENSRNOG00000031993	-0.421906886	5.414354466	25.19022146	5.19E-07	4.42E-06
ENSRNOG00000007336	-0.560692289	3.933139428	25.17730378	5.23E-07	4.45E-06
ENSRNOG00000052119	-1.348476556	-0.253499241	25.16610939	5.26E-07	4.48E-06
ENSRNOG00000055658	-1.008005795	-0.341264783	25.15824349	5.28E-07	4.49E-06
ENSRNOG00000011617	-0.405042016	3.807970393	25.09581654	5.46E-07	4.62E-06
ENSRNOG00000028746	-0.648325462	6.435133903	25.08295268	5.49E-07	4.65E-06
ENSRNOG00000050044	-0.369595576	6.686295544	25.08122491	5.50E-07	4.65E-06
ENSRNOG00000007972	-0.40178378	4.767314351	25.07763534	5.51E-07	4.66E-06
ENSRNOG00000060129	-0.511107789	2.574129098	25.02619596	5.66E-07	4.77E-06
ENSRNOG00000038911	-1.027531103	2.419206034	25.01316889	5.69E-07	4.80E-06
ENSRNOG00000009474	-0.319504543	6.53600819	25.00558879	5.72E-07	4.81E-06
ENSRNOG00000008744	-0.383558905	6.975507139	24.99658918	5.74E-07	4.83E-06
ENSRNOG00000012724	-0.363692844	4.9566364	24.99690956	5.74E-07	4.83E-06
ENSRNOG00000059339	-1.232579535	-0.95912669	24.9956942	5.75E-07	4.83E-06
ENSRNOG00000058658	-0.312733557	5.818484014	24.97869938	5.80E-07	4.86E-06
ENSRNOG00000021113	-0.307940648	5.646569514	24.94415915	5.90E-07	4.94E-06
ENSRNOG00000005366	-0.681642053	3.072587164	24.90467625	6.02E-07	5.04E-06
ENSRNOG00000055572	-0.517363759	7.219134305	24.88016441	6.10E-07	5.09E-06
ENSRNOG00000021758	-0.311300396	6.175604958	24.84669942	6.21E-07	5.18E-06
ENSRNOG00000009462	-0.389481545	4.268610701	24.78888058	6.40E-07	5.31E-06
ENSRNOG00000051058	-0.718634493	0.666310649	24.76787967	6.47E-07	5.37E-06
ENSRNOG00000010825	-0.459805588	4.263797262	24.72977332	6.60E-07	5.47E-06
ENSRNOG00000010659	-0.482197079	3.330607723	24.69463201	6.72E-07	5.57E-06
ENSRNOG00000014027	-0.419892823	4.536524742	24.64760908	6.88E-07	5.70E-06
ENSRNOG00000006744	-0.504465343	3.627160797	24.63952333	6.91E-07	5.72E-06
ENSRNOG00000004268	-0.414788845	5.326330919	24.56305904	7.19E-07	5.93E-06
ENSRNOG00000004717	-0.888368508	0.697608893	24.54263638	7.27E-07	5.98E-06
ENSRNOG00000050828	-0.361546616	3.743760457	24.52436733	7.34E-07	6.04E-06
ENSRNOG00000005710	-0.430076158	4.58735179	24.49954999	7.43E-07	6.11E-06
ENSRNOG00000045569	-0.979484902	0.466369786	24.47232522	7.54E-07	6.18E-06
ENSRNOG00000010999	-0.392672644	5.987409	24.46759154	7.56E-07	6.19E-06
ENSRNOG00000032112	-1.105275872	7.498693624	24.45375968	7.61E-07	6.23E-06
ENSRNOG00000055068	-0.49055836	4.013858416	24.44586232	7.64E-07	6.25E-06
ENSRNOG00000015772	-0.541535739	3.625887051	24.42754797	7.72E-07	6.30E-06
ENSRNOG00000051917	-1.240220028	3.029433878	24.39434818	7.85E-07	6.40E-06
ENSRNOG00000033624	-0.410537724	4.168904047	24.39052455	7.87E-07	6.41E-06
ENSRNOG00000042665	-0.852960366	0.233544112	24.36650376	7.96E-07	6.49E-06
ENSRNOG00000004735	-0.338536057	4.81769209	24.36532541	7.97E-07	6.49E-06
ENSRNOG00000024142	-0.57977329	6.697558941	24.33860822	8.08E-07	6.57E-06
ENSRNOG00000035526	-1.365459235	-0.595342787	24.33237925	8.11E-07	6.59E-06
ENSRNOG00000057352	-0.429376453	3.33560001	24.30686381	8.21E-07	6.67E-06
ENSRNOG00000004996	-0.479549339	4.678506099	24.27747721	8.34E-07	6.77E-06
ENSRNOG00000006895	-0.331716702	5.756780951	24.27548243	8.35E-07	6.77E-06
ENSRNOG00000060982	-0.85963901	0.998567542	24.2695657	8.38E-07	6.78E-06
ENSRNOG00000002291	-0.446450475	3.785011503	24.24635668	8.48E-07	6.85E-06
ENSRNOG00000041744	-0.629037577	6.393312065	24.20155142	8.68E-07	7.00E-06
ENSRNOG00000004459	-0.824631906	1.987991808	24.18726954	8.74E-07	7.04E-06
ENSRNOG00000027022	-0.836266101	0.246276363	24.18704761	8.74E-07	7.04E-06
ENSRNOG00000003218	-0.395453443	4.004800297	24.17084151	8.82E-07	7.10E-06
ENSRNOG00000001754	-0.431921568	3.045651728	24.14684712	8.93E-07	7.18E-06
ENSRNOG00000051860	-0.489905329	6.581967968	24.12763274	9.02E-07	7.24E-06
ENSRNOG00000013454	-0.353957419	5.204600397	24.10195975	9.14E-07	7.33E-06
ENSRNOG00000007992	-0.436549366	4.952126632	24.09042734	9.19E-07	7.37E-06
ENSRNOG00000014097	-0.282726891	6.635890608	24.0274923	9.50E-07	7.60E-06
ENSRNOG00000025625	-0.489697148	6.591986159	24.00873791	9.59E-07	7.66E-06
ENSRNOG00000043053	-0.366599829	3.789558045	24.00626045	9.60E-07	7.67E-06
ENSRNOG00000002474	-0.357408518	5.068636118	23.93637675	9.96E-07	7.92E-06
ENSRNOG00000018304	-0.29795358	6.404436979	23.91078369	1.01E-06	8.02E-06
ENSRNOG00000029115	-0.828667506	1.56824138	23.86855648	1.03E-06	8.18E-06
ENSRNOG00000027818	-0.397950729	3.842399497	23.85319802	1.04E-06	8.24E-06
ENSRNOG00000051756	-0.408032849	4.735627534	23.82562561	1.05E-06	8.35E-06
ENSRNOG00000010133	-0.287043916	6.716294507	23.77734002	1.08E-06	8.55E-06
ENSRNOG00000016484	-0.657662891	4.047453995	23.77221599	1.08E-06	8.57E-06
ENSRNOG00000051468	-1.603166262	-0.861166642	23.73499079	1.11E-06	8.73E-06
ENSRNOG00000019785	-0.315731115	5.315169361	23.69213838	1.13E-06	8.92E-06
ENSRNOG00000008551	-0.489523732	7.785999849	23.68517357	1.13E-06	8.94E-06
ENSRNOG00000003846	-0.385531218	7.566581197	23.65711314	1.15E-06	9.06E-06
ENSRNOG00000008482	-0.471613731	3.596911042	23.64653306	1.16E-06	9.10E-06
ENSRNOG00000018320	-0.520871734	7.941488266	23.62022784	1.17E-06	9.22E-06
ENSRNOG00000013211	-0.323820522	5.782374419	23.61843503	1.17E-06	9.23E-06
ENSRNOG00000003782	-0.402388219	5.406726216	23.56618703	1.21E-06	9.46E-06
ENSRNOG00000037707	-0.383827617	4.005015383	23.5635429	1.21E-06	9.47E-06
ENSRNOG00000025181	-0.51632783	4.568530573	23.51221852	1.24E-06	9.70E-06
ENSRNOG00000010087	-0.404790097	3.609112768	23.51060781	1.24E-06	9.70E-06
ENSRNOG00000042499	-0.776783061	7.634693191	23.50810939	1.24E-06	9.71E-06
ENSRNOG00000019201	-0.357687183	5.069765718	23.50113769	1.25E-06	9.74E-06
ENSRNOG00000000236	-0.297689654	6.845755183	23.50017148	1.25E-06	9.74E-06
ENSRNOG00000018972	-0.315043276	6.800734472	23.42519595	1.30E-06	1.01E-05
ENSRNOG00000045535	-0.367490692	6.260538529	23.42187745	1.30E-06	1.01E-05
ENSRNOG00000058914	-0.420113569	5.354168079	23.37611972	1.33E-06	1.03E-05
ENSRNOG00000016038	-0.727800498	1.157018182	23.37156118	1.34E-06	1.04E-05
ENSRNOG00000050786	-1.056976242	-0.871096932	23.35220008	1.35E-06	1.04E-05
ENSRNOG00000015322	-0.603076542	1.952639595	23.33811681	1.36E-06	1.05E-05
ENSRNOG00000032042	-0.41217557	4.959673453	23.33132719	1.36E-06	1.05E-05
ENSRNOG00000025997	-0.37156997	4.778361481	23.31964107	1.37E-06	1.06E-05
ENSRNOG00000048028	-2.034288381	-0.683801768	23.30001437	1.39E-06	1.07E-05
ENSRNOG00000005426	-0.393379121	3.874913585	23.25224011	1.42E-06	1.09E-05
ENSRNOG00000011258	-0.527035079	3.779504983	23.2370648	1.43E-06	1.10E-05
ENSRNOG00000023233	-0.481398704	3.365868781	23.23600119	1.43E-06	1.10E-05
ENSRNOG00000029805	-0.473414659	3.142479177	23.22221937	1.44E-06	1.11E-05
ENSRNOG00000047848	-0.897573481	-0.155910585	23.18168775	1.47E-06	1.13E-05
ENSRNOG00000031656	-0.451652696	4.835301899	23.15459742	1.49E-06	1.14E-05
ENSRNOG00000015145	-0.383307502	5.568777804	23.12907862	1.51E-06	1.16E-05

ENSRNOG00000003919	-0.349765391	5.155285236	23.12279061	1.52E-06	1.16E-05
ENSRNOG00000051299	-0.78861769	0.861049821	23.1088118	1.53E-06	1.17E-05
ENSRNOG00000045884	-0.575904242	5.148623555	23.10433153	1.53E-06	1.17E-05
ENSRNOG00000020692	-0.468936295	3.914394403	23.09097537	1.55E-06	1.18E-05
ENSRNOG00000004574	-0.36227669	4.300473132	23.066541	1.56E-06	1.19E-05
ENSRNOG00000019235	-0.402269358	4.669542793	23.06019633	1.57E-06	1.19E-05
ENSRNOG00000003863	-0.333189651	4.082078668	23.04159009	1.59E-06	1.20E-05
ENSRNOG00000050645	-0.56783846	1.448994835	22.99854801	1.62E-06	1.23E-05
ENSRNOG00000011420	-0.388915294	6.075372336	22.99550441	1.62E-06	1.23E-05
ENSRNOG00000014961	-0.478249641	4.190244279	22.97623475	1.64E-06	1.24E-05
ENSRNOG00000013947	-0.407369105	4.515334355	22.96419772	1.65E-06	1.25E-05
ENSRNOG00000002759	-0.351053929	4.701457203	22.92994415	1.68E-06	1.27E-05
ENSRNOG00000054832	-0.779180131	1.210312326	22.92077157	1.69E-06	1.27E-05
ENSRNOG00000017770	-0.303185083	6.056174713	22.91499351	1.69E-06	1.28E-05
ENSRNOG00000025285	-0.376470933	5.526345677	22.9131197	1.69E-06	1.28E-05
ENSRNOG00000010085	-0.472115688	5.078839136	22.90552988	1.70E-06	1.28E-05
ENSRNOG00000020204	-0.354914929	5.313093712	22.88787413	1.72E-06	1.29E-05
ENSRNOG00000015670	-0.529616606	4.530854549	22.8679702	1.74E-06	1.31E-05
ENSRNOG00000022745	-0.324079237	5.336214607	22.84376923	1.76E-06	1.32E-05
ENSRNOG00000022227	-0.578461886	1.915782597	22.79581064	1.80E-06	1.35E-05
ENSRNOG00000040273	-0.865321921	0.521970183	22.79538939	1.80E-06	1.35E-05
ENSRNOG00000058316	-1.5073055	-0.366011034	22.78459424	1.81E-06	1.36E-05
ENSRNOG00000029146	-0.375330447	4.62404497	22.72240112	1.87E-06	1.40E-05
ENSRNOG00000025504	-0.334592111	7.450785251	22.68574477	1.91E-06	1.43E-05
ENSRNOG00000014808	-0.357957898	4.641760989	22.66385692	1.93E-06	1.44E-05
ENSRNOG00000007671	-0.305915896	6.020066581	22.65096067	1.94E-06	1.45E-05
ENSRNOG00000018767	-0.34903033	5.361581121	22.63411575	1.96E-06	1.47E-05
ENSRNOG00000024533	-0.36823819	5.109784234	22.62876123	1.97E-06	1.47E-05
ENSRNOG00000025629	-0.324674476	4.923700873	22.60492197	1.99E-06	1.49E-05
ENSRNOG00000029971	-0.688985625	13.32827717	22.56758932	2.03E-06	1.51E-05
ENSRNOG00000023946	-0.356833207	3.964032233	22.5567903	2.04E-06	1.52E-05
ENSRNOG00000015020	-0.351079316	5.807277867	22.52415413	2.08E-06	1.54E-05
ENSRNOG00000007203	-0.362176295	6.743942235	22.49224509	2.11E-06	1.57E-05
ENSRNOG00000045741	-0.891304282	-0.191061206	22.46731874	2.14E-06	1.59E-05
ENSRNOG00000007785	-0.31957043	4.009305719	22.45493554	2.15E-06	1.59E-05
ENSRNOG00000022910	-0.792612539	2.898047255	22.42971296	2.18E-06	1.61E-05
ENSRNOG00000015018	-0.410730527	8.041092209	22.41617091	2.20E-06	1.62E-05
ENSRNOG00000008566	-0.275295435	5.458986668	22.40535826	2.21E-06	1.63E-05
ENSRNOG00000021233	-0.39887121	5.484588982	22.34969575	2.27E-06	1.67E-05
ENSRNOG00000026690	-0.423244177	3.062148852	22.319656	2.31E-06	1.70E-05
ENSRNOG00000011844	-0.403513978	3.038750174	22.29126378	2.34E-06	1.72E-05
ENSRNOG00000004629	-0.367928457	7.121344368	22.28806879	2.35E-06	1.72E-05
ENSRNOG00000015552	-0.52251621	5.479845541	22.26327838	2.38E-06	1.74E-05
ENSRNOG00000014075	-0.543301682	3.303953666	22.25250197	2.39E-06	1.75E-05
ENSRNOG00000015122	-0.310749258	5.419183367	22.24085809	2.41E-06	1.76E-05
ENSRNOG00000033611	-0.314332439	5.181965788	22.24171256	2.40E-06	1.76E-05
ENSRNOG00000033177	-1.194937134	-1.103487841	22.20610224	2.45E-06	1.79E-05
ENSRNOG00000018566	-0.352946939	6.921432458	22.20161405	2.45E-06	1.79E-05
ENSRNOG00000011586	-0.34257216	4.957801996	22.18561722	2.48E-06	1.80E-05
ENSRNOG00000004057	-0.346058282	6.66794652	22.18468459	2.48E-06	1.80E-05
ENSRNOG00000007235	-0.392255879	6.332315745	22.18279873	2.48E-06	1.80E-05
ENSRNOG00000011400	-0.422691794	5.952331983	22.17329608	2.49E-06	1.81E-05
ENSRNOG00000033625	-1.598070705	-0.664607078	22.16412183	2.50E-06	1.82E-05
ENSRNOG00000010090	-0.462571653	3.839809083	22.14641253	2.53E-06	1.83E-05
ENSRNOG00000061146	-0.467242685	3.115758233	22.12804008	2.55E-06	1.85E-05
ENSRNOG00000010636	-0.443566729	3.304977876	22.11357959	2.57E-06	1.86E-05
ENSRNOG00000025716	-0.448652675	3.050352267	22.0460959	2.66E-06	1.93E-05
ENSRNOG00000005662	-0.331935077	5.269417228	22.03693665	2.67E-06	1.93E-05
ENSRNOG00000012844	-0.261677207	6.503800663	22.03196198	2.68E-06	1.94E-05
ENSRNOG00000001680	-0.430406647	3.133946645	22.03032232	2.68E-06	1.94E-05
ENSRNOG00000013768	-2.288361	-0.425302326	22.02878028	2.69E-06	1.94E-05
ENSRNOG00000019892	-0.533391797	4.412323288	21.96416028	2.78E-06	2.00E-05
ENSRNOG00000057291	-1.015122529	0.694858109	21.95005903	2.80E-06	2.01E-05
ENSRNOG00000023356	-0.343852767	6.276604949	21.92778737	2.83E-06	2.03E-05
ENSRNOG00000010540	-0.369370913	5.086755798	21.92498894	2.84E-06	2.04E-05
ENSRNOG00000022393	-0.67124143	1.580344356	21.91685022	2.85E-06	2.04E-05
ENSRNOG00000014493	-0.26791673	5.341912269	21.91146913	2.86E-06	2.05E-05
ENSRNOG00000058484	-0.755205395	5.448309903	21.90197693	2.87E-06	2.06E-05
ENSRNOG00000056954	-0.334428678	4.800479941	21.87307166	2.91E-06	2.08E-05
ENSRNOG00000011237	-0.343889218	6.20807509	21.8614687	2.93E-06	2.10E-05
ENSRNOG00000018993	-0.336132539	6.295275032	21.85284749	2.94E-06	2.10E-05
ENSRNOG00000011830	-0.297709826	5.21803121	21.83952449	2.96E-06	2.12E-05
ENSRNOG00000004554	-0.874583804	3.562032214	21.82547266	2.99E-06	2.13E-05
ENSRNOG00000006983	-0.304774133	4.649134058	21.81994309	2.99E-06	2.14E-05
ENSRNOG00000028908	-0.671884467	3.421768254	21.81770266	3.00E-06	2.14E-05
ENSRNOG00000001478	-0.602341742	2.100246305	21.81499337	3.00E-06	2.14E-05
ENSRNOG00000027761	-0.33415547	6.160057772	21.80668262	3.02E-06	2.15E-05
ENSRNOG00000055330	-0.495986979	2.520655413	21.80253421	3.02E-06	2.15E-05
ENSRNOG00000037673	-0.613610506	4.208831851	21.78771062	3.05E-06	2.17E-05
ENSRNOG00000019940	-0.309914752	5.602970139	21.78678964	3.05E-06	2.17E-05
ENSRNOG00000025768	-0.412407056	7.124442548	21.76013053	3.09E-06	2.19E-05
ENSRNOG00000009891	-0.380632204	5.549205744	21.74316008	3.12E-06	2.21E-05
ENSRNOG00000026324	-0.573666643	2.039539972	21.7443705	3.12E-06	2.21E-05
ENSRNOG00000028611	-0.507977063	2.450918296	21.72587994	3.15E-06	2.23E-05
ENSRNOG00000014618	-0.369929786	4.202698562	21.71927357	3.16E-06	2.23E-05
ENSRNOG00000026091	-0.64983626	4.336784974	21.71768277	3.16E-06	2.23E-05
ENSRNOG00000050015	-1.077113673	0.410459801	21.70004021	3.19E-06	2.25E-05
ENSRNOG00000005565	-0.593004059	2.799172379	21.70045483	3.19E-06	2.25E-05
ENSRNOG00000013919	-0.463834573	5.353366767	21.67728639	3.23E-06	2.28E-05
ENSRNOG00000005513	-0.36730043	7.527885317	21.66814053	3.24E-06	2.29E-05
ENSRNOG00000002536	-0.368292468	5.17784714	21.66031359	3.25E-06	2.29E-05
ENSRNOG00000005589	-0.497321663	5.480709575	21.63466943	3.30E-06	2.32E-05
ENSRNOG00000021881	-0.339574601	6.354895849	21.63499239	3.30E-06	2.32E-05
ENSRNOG00000055860	-0.315388441	8.233182996	21.62786597	3.31E-06	2.33E-05
ENSRNOG00000005101	-0.386197188	5.107956085	21.6265369	3.31E-06	2.33E-05
ENSRNOG00000020456	-0.403016606	5.070615633	21.61726917	3.33E-06	2.34E-05
ENSRNOG00000037227	-0.418087258	3.718710332	21.5763198	3.40E-06	2.38E-05
ENSRNOG00000008734	-0.330331958	6.550782726	21.56661307	3.42E-06	2.39E-05
ENSRNOG00000048961	-0.810198683	4.985769116	21.51419412	3.51E-06	2.45E-05
ENSRNOG00000017945	-0.509598044	2.723279959	21.47287958	3.59E-06	2.50E-05

ENSRNOG00000006103	-0.370977526	4.309657354	21.46660168	3.60E-06	2.50E-05
ENSRNOG00000005809	-0.678816337	4.031567491	21.46391704	3.61E-06	2.51E-05
ENSRNOG00000011758	-0.578609354	2.992586985	21.45940445	3.61E-06	2.51E-05
ENSRNOG00000014139	-0.384766529	3.914198526	21.45010333	3.63E-06	2.52E-05
ENSRNOG00000024924	-0.410077155	5.997574237	21.42330173	3.68E-06	2.56E-05
ENSRNOG00000061630	-0.418469871	4.270240578	21.42038079	3.69E-06	2.56E-05
ENSRNOG00000000957	-0.342725907	8.419748725	21.39695417	3.73E-06	2.59E-05
ENSRNOG00000000493	-0.672291039	3.498969304	21.38877537	3.75E-06	2.60E-05
ENSRNOG00000026267	-0.390721642	4.870004066	21.38328742	3.76E-06	2.60E-05
ENSRNOG00000011519	-0.351960633	4.655858159	21.33221106	3.86E-06	2.67E-05
ENSRNOG00000017963	-0.381927915	3.663316745	21.32222544	3.88E-06	2.68E-05
ENSRNOG00000012292	-0.438935261	3.080130901	21.30584156	3.92E-06	2.70E-05
ENSRNOG00000021269	-0.357627704	8.545083304	21.30341818	3.92E-06	2.70E-05
ENSRNOG00000005264	-0.329206796	4.388759018	21.30366905	3.92E-06	2.70E-05
ENSRNOG00000018333	-0.311844487	5.848641955	21.29543059	3.94E-06	2.71E-05
ENSRNOG00000005689	-0.352510368	4.462272249	21.22168429	4.09E-06	2.81E-05
ENSRNOG00000014419	-0.257500399	6.076237506	21.20923229	4.12E-06	2.82E-05
ENSRNOG00000010550	-0.28785638	5.873033486	21.20559296	4.13E-06	2.83E-05
ENSRNOG00000003260	-0.489216941	4.423736755	21.20544096	4.13E-06	2.83E-05
ENSRNOG00000007446	-0.513382471	3.110857924	21.18680039	4.17E-06	2.85E-05
ENSRNOG00000027006	-0.558792648	4.445855902	21.14853088	4.25E-06	2.90E-05
ENSRNOG00000055777	-1.464349682	-0.215814924	21.09257299	4.38E-06	2.98E-05
ENSRNOG00000017170	-0.316644814	4.177057747	21.08830835	4.39E-06	2.99E-05
ENSRNOG00000015078	-0.727824656	3.673945471	21.07386901	4.42E-06	3.01E-05
ENSRNOG00000011296	-0.427104693	3.07768646	21.06309398	4.44E-06	3.02E-05
ENSRNOG00000018417	-0.308881875	5.703197064	21.04873123	4.48E-06	3.04E-05
ENSRNOG00000053757	-0.907284106	-0.374046878	21.02349045	4.54E-06	3.08E-05
ENSRNOG00000008155	-0.58859098	1.953161232	21.02326927	4.54E-06	3.08E-05
ENSRNOG00000047984	-0.295124804	4.635428036	20.99914524	4.59E-06	3.11E-05
ENSRNOG00000030628	-0.319700317	8.046699537	20.99728292	4.60E-06	3.12E-05
ENSRNOG00000010141	-0.312174576	4.919489162	20.98227807	4.64E-06	3.14E-05
ENSRNOG00000049689	-0.608750394	3.299473199	20.98228074	4.64E-06	3.14E-05
ENSRNOG00000058789	-0.625292989	1.322726271	20.94794264	4.72E-06	3.19E-05
ENSRNOG00000033623	-0.305609001	3.959892003	20.94193147	4.73E-06	3.20E-05
ENSRNOG00000029456	-0.708529699	2.270565166	20.90303214	4.83E-06	3.26E-05
ENSRNOG00000003825	-0.320132245	5.029357451	20.88397037	4.88E-06	3.29E-05
ENSRNOG00000043348	-0.49290872	5.553224567	20.87733048	4.90E-06	3.30E-05
ENSRNOG00000016356	-0.33957876	8.391818462	20.86174885	4.94E-06	3.32E-05
ENSRNOG00000018590	-0.319080937	5.218889448	20.86229081	4.94E-06	3.32E-05
ENSRNOG00000011740	-0.382401403	4.22121463	20.84314633	4.98E-06	3.35E-05
ENSRNOG00000061415	-0.400712569	3.189244937	20.83950718	4.99E-06	3.35E-05
ENSRNOG00000021047	-0.326730504	4.790744665	20.81438744	5.06E-06	3.40E-05
ENSRNOG00000012664	-0.388674091	3.211050169	20.7911315	5.12E-06	3.43E-05
ENSRNOG00000003279	-0.44243471	2.883499605	20.76955845	5.18E-06	3.47E-05
ENSRNOG00000004161	-0.346408734	4.546756576	20.76814898	5.18E-06	3.47E-05
ENSRNOG00000018467	-0.509672991	3.075332977	20.76253799	5.20E-06	3.48E-05
ENSRNOG00000046705	-0.36621977	7.416730804	20.73050578	5.29E-06	3.53E-05
ENSRNOG00000001884	-0.542811375	5.765630874	20.709765	5.34E-06	3.57E-05
ENSRNOG00000016121	-0.373754937	4.836886962	20.68705631	5.41E-06	3.61E-05
ENSRNOG00000005993	-0.431530374	3.858289047	20.64810609	5.52E-06	3.68E-05
ENSRNOG00000061039	-0.409294089	4.738547176	20.62592742	5.58E-06	3.72E-05
ENSRNOG00000028651	-0.374838152	4.537129148	20.61625736	5.61E-06	3.73E-05
ENSRNOG00000024497	-0.300802089	4.866269896	20.60588661	5.64E-06	3.75E-05
ENSRNOG00000057615	-1.025600624	-0.709637835	20.57190804	5.74E-06	3.80E-05
ENSRNOG00000002094	-0.275043611	5.698078807	20.57233126	5.74E-06	3.80E-05
ENSRNOG00000047578	-0.347690281	3.667620781	20.56044416	5.78E-06	3.83E-05
ENSRNOG00000058105	-0.837090838	7.266802832	20.53560873	5.85E-06	3.87E-05
ENSRNOG00000018692	-0.739961649	2.762089996	20.52913894	5.87E-06	3.88E-05
ENSRNOG00000055222	-0.599683734	1.274254216	20.46204178	6.08E-06	4.01E-05
ENSRNOG00000004424	-0.432342398	3.710677288	20.45793488	6.10E-06	4.02E-05
ENSRNOG00000009568	-0.292101328	4.972982097	20.45155282	6.12E-06	4.03E-05
ENSRNOG00000001345	-0.369735998	5.201778379	20.43961834	6.15E-06	4.05E-05
ENSRNOG00000057622	-0.420385221	3.470574537	20.43980535	6.15E-06	4.05E-05
ENSRNOG00000047138	-0.56895456	1.744452682	20.426968	6.20E-06	4.08E-05
ENSRNOG00000052506	-0.369061165	5.051233587	20.41627839	6.23E-06	4.09E-05
ENSRNOG00000027596	-0.319905236	5.937956087	20.41604077	6.23E-06	4.09E-05
ENSRNOG00000010230	-0.301152413	5.464890314	20.40550764	6.26E-06	4.11E-05
ENSRNOG00000009451	-0.405266426	4.749066742	20.37327748	6.37E-06	4.17E-05
ENSRNOG00000013802	-0.345412335	5.241380575	20.32936928	6.52E-06	4.27E-05
ENSRNOG00000012763	-0.331174321	4.965985716	20.32198581	6.54E-06	4.28E-05
ENSRNOG00000009745	-0.403948987	4.424539454	20.31689737	6.56E-06	4.29E-05
ENSRNOG00000017905	-0.551518123	7.441683005	20.30556781	6.60E-06	4.31E-05
ENSRNOG00000020753	-0.32058743	3.83700688	20.30252482	6.61E-06	4.32E-05
ENSRNOG00000052870	-0.979270696	-0.439409604	20.30026962	6.62E-06	4.32E-05
ENSRNOG00000005539	-0.456138316	2.742669584	20.24796717	6.80E-06	4.43E-05
ENSRNOG00000010861	-0.277652815	7.159238383	20.24215747	6.82E-06	4.44E-05
ENSRNOG00000007126	-0.381429619	3.402242011	20.24218931	6.82E-06	4.44E-05
ENSRNOG00000019091	-0.313264411	6.313046072	20.22808337	6.87E-06	4.47E-05
ENSRNOG00000000368	-0.341056268	6.520431122	20.17202135	7.08E-06	4.60E-05
ENSRNOG00000060229	-0.305224081	7.887369454	20.11792541	7.28E-06	4.72E-05
ENSRNOG00000017826	-0.316209569	4.639869235	20.09329678	7.38E-06	4.78E-05
ENSRNOG00000059715	-0.483990778	5.793006112	20.07714141	7.44E-06	4.81E-05
ENSRNOG00000014565	-0.255341437	5.408484655	20.0497644	7.55E-06	4.87E-05
ENSRNOG00000014119	-0.303715075	5.558383412	20.0463753	7.56E-06	4.88E-05
ENSRNOG00000012505	-0.368606954	3.932949681	20.02462808	7.65E-06	4.93E-05
ENSRNOG00000004610	-0.705965745	2.671852495	20.01413514	7.69E-06	4.95E-05
ENSRNOG00000042333	-0.440044075	6.314251237	19.99342252	7.77E-06	5.00E-05
ENSRNOG00000005183	-0.295355613	6.484934372	19.96041591	7.91E-06	5.08E-05
ENSRNOG00000029244	-0.414732823	2.569300706	19.96095761	7.90E-06	5.08E-05
ENSRNOG00000019943	-0.350474195	5.371540458	19.92643862	8.05E-06	5.17E-05
ENSRNOG00000014160	-0.359848072	6.835498197	19.88127364	8.24E-06	5.28E-05
ENSRNOG00000013381	-0.285636448	6.518681366	19.87958181	8.25E-06	5.28E-05
ENSRNOG00000009378	-0.310126485	8.885642077	19.87374107	8.27E-06	5.30E-05
ENSRNOG00000009799	-0.445604191	5.234177912	19.84787555	8.39E-06	5.36E-05
ENSRNOG00000031335	-0.771396861	2.496829567	19.84677436	8.39E-06	5.36E-05
ENSRNOG00000008455	-0.337405074	4.825076416	19.76360458	8.76E-06	5.59E-05
ENSRNOG00000010208	-0.734484927	1.384637953	19.75917537	8.78E-06	5.59E-05
ENSRNOG00000060337	-0.390668212	3.606113838	19.75812146	8.79E-06	5.59E-05
ENSRNOG00000058050	-0.359598693	7.173075283	19.72639553	8.94E-06	5.68E-05
ENSRNOG00000038868	-0.346322141	5.240974302	19.66888863	9.21E-06	5.85E-05

ENSRNOG00000010748	-0.368234804	6.450230794	19.66488753	9.23E-06	5.86E-05
ENSRNOG00000057372	-0.45382235	3.979216288	19.65289393	9.29E-06	5.90E-05
ENSRNOG00000001611	-0.336040862	7.272269734	19.64676188	9.32E-06	5.91E-05
ENSRNOG00000004891	-0.291137829	4.947377617	19.64194407	9.34E-06	5.92E-05
ENSRNOG00000052990	-0.245257474	5.780371071	19.63030487	9.40E-06	5.96E-05
ENSRNOG00000055908	-0.81815571	1.827466282	19.62555388	9.42E-06	5.97E-05
ENSRNOG00000002177	-0.348455269	4.74015817	19.62242851	9.44E-06	5.98E-05
ENSRNOG00000051682	-0.349080837	5.321324902	19.61968902	9.45E-06	5.98E-05
ENSRNOG00000016265	-0.329412416	5.436056049	19.59307743	9.58E-06	6.05E-05
ENSRNOG00000027408	-0.33822706	6.020425126	19.54893586	9.81E-06	6.19E-05
ENSRNOG00000013631	-0.406126369	2.600080403	19.50570527	1.00E-05	6.31E-05
ENSRNOG00000033617	-0.448770972	4.462668242	19.48018071	1.02E-05	6.39E-05
ENSRNOG00000049505	-0.541634973	3.260777136	19.47556678	1.02E-05	6.40E-05
ENSRNOG00000026742	-0.274007366	7.080493558	19.47341876	1.02E-05	6.41E-05
ENSRNOG00000020927	-0.847244226	0.591793904	19.45300586	1.03E-05	6.47E-05
ENSRNOG00000007099	-0.318129462	5.669721498	19.45375684	1.03E-05	6.47E-05
ENSRNOG00000033226	-1.077406244	0.053946847	19.44005577	1.04E-05	6.51E-05
ENSRNOG00000054795	-0.549845864	3.476866464	19.41809858	1.05E-05	6.58E-05
ENSRNOG00000033065	-0.375336413	5.156813833	19.39105255	1.07E-05	6.66E-05
ENSRNOG00000000858	-0.704018412	1.196144737	19.38220112	1.07E-05	6.69E-05
ENSRNOG00000049819	-0.321458882	4.988934931	19.37665296	1.07E-05	6.70E-05
ENSRNOG00000020179	-0.269342141	6.110369769	19.37448804	1.07E-05	6.71E-05
ENSRNOG00000057759	-0.602161223	1.531530808	19.32848624	1.10E-05	6.86E-05
ENSRNOG00000013520	-0.343839553	7.983810691	19.32632988	1.10E-05	6.87E-05
ENSRNOG00000042139	-0.8407525	0.832645299	19.28250404	1.13E-05	7.01E-05
ENSRNOG00000011618	-0.359283797	3.923969603	19.2718368	1.13E-05	7.04E-05
ENSRNOG00000010887	-0.343805498	4.401348522	19.26119505	1.14E-05	7.08E-05
ENSRNOG00000021660	-0.291567285	4.637162449	19.26103991	1.14E-05	7.08E-05
ENSRNOG00000059148	-0.933381979	-0.255196514	19.24908342	1.15E-05	7.11E-05
ENSRNOG00000000780	-0.322763309	6.477234315	19.24951437	1.15E-05	7.11E-05
ENSRNOG00000000804	-0.324166918	4.752370842	19.23377094	1.16E-05	7.16E-05
ENSRNOG00000062147	-0.902582751	-0.357644373	19.23043913	1.16E-05	7.17E-05
ENSRNOG00000011471	-0.587210118	3.684260057	19.23035874	1.16E-05	7.17E-05
ENSRNOG00000017838	-0.257895892	5.966311797	19.22816336	1.16E-05	7.17E-05
ENSRNOG00000016825	-0.487393015	2.98149991	19.22697844	1.16E-05	7.17E-05
ENSRNOG00000010164	-0.372213745	5.684795694	19.20271543	1.18E-05	7.25E-05
ENSRNOG00000051248	-0.404549555	3.691928442	19.20195115	1.18E-05	7.25E-05
ENSRNOG00000021748	-0.371351817	6.051357658	19.19478377	1.18E-05	7.28E-05
ENSRNOG00000014354	-0.407435959	4.419842634	19.19119341	1.18E-05	7.29E-05
ENSRNOG00000013078	-0.366115554	4.972428405	19.18144006	1.19E-05	7.32E-05
ENSRNOG00000055553	-1.081848667	-0.734629845	19.1789711	1.19E-05	7.33E-05
ENSRNOG00000018211	-0.288336499	5.00419025	19.17488796	1.19E-05	7.34E-05
ENSRNOG00000010934	-0.90413835	2.957934066	19.16768161	1.20E-05	7.36E-05
ENSRNOG00000054118	-0.363647841	6.084302229	19.16459313	1.20E-05	7.37E-05
ENSRNOG00000003025	-0.373196077	6.307630138	19.11471697	1.23E-05	7.55E-05
ENSRNOG00000002884	-0.378200633	3.814794519	19.10127711	1.24E-05	7.60E-05
ENSRNOG00000050553	-0.670902233	2.32111546	19.04203768	1.28E-05	7.82E-05
ENSRNOG00000023923	-0.355033598	3.541835358	19.03391727	1.28E-05	7.85E-05
ENSRNOG00000006652	-0.291709364	7.077872992	19.03150282	1.29E-05	7.86E-05
ENSRNOG00000013135	-0.30097375	5.518978626	19.01812165	1.29E-05	7.91E-05
ENSRNOG00000022455	-0.509084654	4.955955639	18.97829397	1.32E-05	8.06E-05
ENSRNOG00000004710	-0.424304521	3.180261898	18.94988345	1.34E-05	8.17E-05
ENSRNOG00000009848	-0.640718337	2.097095581	18.91622513	1.37E-05	8.31E-05
ENSRNOG00000021637	-0.281628576	6.46345924	18.89613598	1.38E-05	8.39E-05
ENSRNOG00000013111	-0.285445877	5.360361054	18.84489033	1.42E-05	8.60E-05
ENSRNOG00000015332	-0.378778063	4.953396403	18.80686166	1.45E-05	8.76E-05
ENSRNOG00000058412	-0.340450126	8.23876328	18.74793703	1.49E-05	9.02E-05
ENSRNOG00000009406	-0.303028218	5.676128616	18.74012219	1.50E-05	9.05E-05
ENSRNOG00000051812	-0.849149251	1.267521584	18.73826792	1.50E-05	9.06E-05
ENSRNOG00000016462	-0.359892105	3.567332081	18.73499397	1.50E-05	9.07E-05
ENSRNOG00000037595	-0.404118752	4.685208311	18.73460816	1.50E-05	9.07E-05
ENSRNOG00000013930	-0.347393834	5.540396037	18.72507091	1.51E-05	9.11E-05
ENSRNOG00000045698	-0.456840778	4.989252703	18.71184153	1.52E-05	9.16E-05
ENSRNOG00000049006	-0.488268762	3.88583608	18.69526022	1.53E-05	9.24E-05
ENSRNOG00000039350	-0.635488042	3.394680093	18.66220246	1.56E-05	9.39E-05
ENSRNOG00000009176	-0.267170675	5.580384215	18.6592942	1.56E-05	9.40E-05
ENSRNOG00000019551	-0.627103051	6.367770121	18.65135798	1.57E-05	9.43E-05
ENSRNOG00000022884	-0.899323628	2.51238813	18.62464877	1.59E-05	9.55E-05
ENSRNOG00000016686	-0.424798267	3.990459781	18.59855239	1.61E-05	9.68E-05
ENSRNOG00000051570	-0.510903505	2.102704625	18.56266629	1.64E-05	9.83E-05
ENSRNOG00000032471	-0.286250563	4.617031084	18.53887727	1.66E-05	9.94E-05
ENSRNOG00000056770	-0.89871393	0.588687319	18.52154396	1.68E-05	0.000100152
ENSRNOG00000029549	-0.306602588	5.181443762	18.51635371	1.68E-05	0.000100349
ENSRNOG00000015137	-0.530650913	1.993553352	18.50773355	1.69E-05	0.000100766
ENSRNOG00000013579	-0.392953316	4.528429611	18.4984922	1.70E-05	0.000101142
ENSRNOG00000037710	-0.633096798	2.323072788	18.49679509	1.70E-05	0.000101194
ENSRNOG00000056647	-0.399016618	3.952801984	18.49499995	1.70E-05	0.000101251
ENSRNOG00000014588	-0.379132885	3.532923574	18.47029907	1.73E-05	0.000102457
ENSRNOG00000046109	-0.575921755	4.419173331	18.45107918	1.74E-05	0.000103418
ENSRNOG00000014071	-0.384117154	2.964952754	18.42233524	1.77E-05	0.000104871
ENSRNOG00000009432	-0.335179041	5.654826641	18.40565432	1.79E-05	0.000105754
ENSRNOG00000004083	-0.290427007	6.186724709	18.39626472	1.79E-05	0.000106157
ENSRNOG00000020495	-0.722459481	6.307627701	18.3881991	1.80E-05	0.000106528
ENSRNOG00000027797	-0.340508625	4.984875485	18.38075754	1.81E-05	0.000106905
ENSRNOG00000021099	-0.300572246	4.505842371	18.37512102	1.81E-05	0.000107182
ENSRNOG00000007851	-0.302474332	6.860665435	18.34756227	1.84E-05	0.000108662
ENSRNOG00000031328	-0.363177596	5.071286869	18.33387632	1.85E-05	0.000109405
ENSRNOG00000011020	-0.244489084	5.670529052	18.29938368	1.89E-05	0.000111279
ENSRNOG00000006432	-0.265606229	5.750347773	18.27340885	1.91E-05	0.000112555
ENSRNOG00000022101	-0.773181411	2.321101386	18.27011233	1.92E-05	0.000112677
ENSRNOG00000055672	-0.705777099	0.929728555	18.26563927	1.92E-05	0.000112819
ENSRNOG00000007581	-0.57604304	3.678359406	18.25902239	1.93E-05	0.000113156
ENSRNOG00000010147	-0.334350493	5.819453423	18.25679648	1.93E-05	0.000113243
ENSRNOG00000001524	-0.386145797	4.557811722	18.25615799	1.93E-05	0.000113243
ENSRNOG00000015250	-0.252434526	5.058812538	18.24280245	1.94E-05	0.000113913
ENSRNOG00000051256	-0.345774245	2.836570617	18.23054393	1.96E-05	0.000114606
ENSRNOG00000006375	-0.294707547	7.959382677	18.21915297	1.97E-05	0.000115251
ENSRNOG00000009411	-0.559556375	6.592772231	18.21647457	1.97E-05	0.000115328
ENSRNOG00000030683	-0.302963143	4.864862161	18.21698941	1.97E-05	0.000115328
ENSRNOG00000028919	-0.319492337	4.010742911	18.21420175	1.97E-05	0.000115423

ENSRNOG00000000853	-0.56233835	4.209862602	18.20649078	1.98E-05	0.000115806
ENSRNOG00000007392	-0.463923653	6.482724593	18.20019296	1.99E-05	0.000116146
ENSRNOG00000016134	-0.355233321	5.394524752	18.1992819	1.99E-05	0.000116159
ENSRNOG00000008374	-0.273420608	6.700435004	18.19250544	2.00E-05	0.00011653
ENSRNOG00000050224	-0.322025342	4.582524002	18.18222668	2.01E-05	0.000117032
ENSRNOG00000027430	-0.577212238	1.494313357	18.18162092	2.01E-05	0.000117032
ENSRNOG00000007766	-0.411517934	4.954780754	18.18020002	2.01E-05	0.00011707
ENSRNOG00000043596	-0.635330331	6.587810341	18.17884778	2.01E-05	0.00011711
ENSRNOG00000023650	-0.393631856	6.098814639	18.17631058	2.01E-05	0.000117187
ENSRNOG00000047439	-0.428345005	3.830732814	18.16267752	2.03E-05	0.000117935
ENSRNOG00000007372	-0.693480778	1.788481544	18.1528362	2.04E-05	0.000118459
ENSRNOG00000008689	-0.340519609	4.341026885	18.15290879	2.04E-05	0.000118459
ENSRNOG00000050476	-0.54038344	2.253826804	18.14449218	2.05E-05	0.000118936
ENSRNOG00000017647	-0.380941604	4.098852232	18.14133338	2.05E-05	0.000119046
ENSRNOG00000022742	-0.330554774	3.486584369	18.13739326	2.06E-05	0.000119118
ENSRNOG00000012806	-0.284702678	6.306715071	18.11767392	2.08E-05	0.000120225
ENSRNOG00000011621	-0.36637131	5.092916458	18.11248547	2.08E-05	0.000120465
ENSRNOG00000054517	-1.026896989	-0.875003556	18.11023502	2.08E-05	0.000120564
ENSRNOG00000056728	-0.439314441	6.780070389	18.09502768	2.10E-05	0.000121442
ENSRNOG00000021164	-0.366061657	7.708222114	18.08176886	2.12E-05	0.000122112
ENSRNOG00000026323	-0.619274098	1.506214809	18.07881685	2.12E-05	0.000122257
ENSRNOG00000006094	-0.491544559	3.457853303	18.03457526	2.17E-05	0.000124949
ENSRNOG00000009965	-0.708896843	2.702419492	18.03273038	2.17E-05	0.000125025
ENSRNOG00000055319	-0.333541326	8.397559891	18.02175765	2.18E-05	0.000125656
ENSRNOG00000007330	-0.291812662	7.243360527	17.96631805	2.25E-05	0.000129229
ENSRNOG00000011461	-0.318900448	5.715390004	17.93678149	2.28E-05	0.000131154
ENSRNOG00000023593	-0.296561023	6.210345458	17.91655275	2.31E-05	0.000132412
ENSRNOG00000052149	-1.002182719	-0.558955916	17.90852687	2.32E-05	0.000132875
ENSRNOG00000057116	-0.472073268	3.128698279	17.9068241	2.32E-05	0.000132946
ENSRNOG00000016227	-0.298769649	6.271993893	17.90496533	2.32E-05	0.000133028
ENSRNOG00000017123	-0.579802878	8.725142417	17.89427214	2.34E-05	0.000133729
ENSRNOG00000051384	-0.736391738	3.039109319	17.88838053	2.34E-05	0.000134046
ENSRNOG00000007174	-0.308143503	4.75359891	17.88539297	2.35E-05	0.000134209
ENSRNOG00000030750	-0.364933281	4.621992581	17.86165548	2.38E-05	0.000135648
ENSRNOG00000019975	-0.264448764	6.060885533	17.8587017	2.38E-05	0.00013581
ENSRNOG00000031203	-0.311394876	5.548376627	17.85508195	2.38E-05	0.000136003
ENSRNOG00000019659	-0.571470592	5.391231192	17.85317748	2.39E-05	0.000136058
ENSRNOG00000004813	-0.247612282	6.134156584	17.84551137	2.40E-05	0.000136508
ENSRNOG00000019819	-0.349420798	3.813551724	17.84557352	2.40E-05	0.000136508
ENSRNOG00000059163	-0.465022406	2.428731201	17.79699604	2.46E-05	0.000139832
ENSRNOG00000005195	-0.368027413	10.3707641	17.77364618	2.49E-05	0.000141255
ENSRNOG00000043182	-0.24811024	6.028536295	17.77414657	2.49E-05	0.000141255
ENSRNOG00000047611	-0.398680549	6.051004509	17.77002107	2.49E-05	0.000141473
ENSRNOG00000056694	-0.635397342	0.8700148	17.74000847	2.53E-05	0.000143568
ENSRNOG00000062038	-0.659114176	0.557876182	17.73283012	2.54E-05	0.00014406
ENSRNOG00000014459	-0.268433192	5.028603345	17.71611141	2.56E-05	0.000145279
ENSRNOG00000045752	-0.289419308	5.15302252	17.70148269	2.58E-05	0.000146244
ENSRNOG00000010827	-0.314083747	6.223943643	17.68994562	2.60E-05	0.000147011
ENSRNOG00000013594	-0.348875158	5.711091963	17.65702647	2.65E-05	0.000149488
ENSRNOG00000014777	-0.287579856	4.592376928	17.64733999	2.66E-05	0.000150198
ENSRNOG00000061622	-0.914900391	-0.601422847	17.62836512	2.69E-05	0.000151542
ENSRNOG00000018945	-0.254876612	5.772471935	17.62263793	2.69E-05	0.000151891
ENSRNOG00000009974	-0.336446871	4.749657605	17.6186951	2.70E-05	0.000152152
ENSRNOG00000012410	-0.433227521	4.924004767	17.61565644	2.70E-05	0.000152341
ENSRNOG00000009513	-0.312914637	5.891196553	17.60557137	2.72E-05	0.000153042
ENSRNOG00000000618	-0.318053073	4.534778482	17.60116959	2.72E-05	0.000153342
ENSRNOG00000056323	-1.097708239	-0.648718691	17.57696781	2.76E-05	0.000155196
ENSRNOG00000018153	-0.310674515	7.448691101	17.55924315	2.78E-05	0.000156538
ENSRNOG00000009310	-0.312006101	4.525722088	17.55809116	2.79E-05	0.000156577
ENSRNOG00000037898	-1.057516751	-0.668105307	17.54928026	2.80E-05	0.000157193
ENSRNOG00000014936	-0.688495846	2.765472944	17.53937249	2.81E-05	0.000157958
ENSRNOG00000061138	-0.612131432	0.641619616	17.53751594	2.82E-05	0.000158056
ENSRNOG00000014289	-0.345195339	7.423241102	17.53532108	2.82E-05	0.000158183
ENSRNOG00000019079	-0.272552289	4.343041246	17.50334584	2.87E-05	0.000160752
ENSRNOG00000061579	-1.322879373	-0.983459996	17.4964806	2.88E-05	0.000161276
ENSRNOG00000058522	-0.293559362	6.116892035	17.49245071	2.88E-05	0.000161504
ENSRNOG00000056458	-0.323836174	5.479053976	17.47604511	2.91E-05	0.000162846
ENSRNOG00000059480	-0.288477529	5.456469056	17.4310518	2.98E-05	0.000166335
ENSRNOG00000034233	-0.317136542	5.512550722	17.42304903	2.99E-05	0.000166978
ENSRNOG00000048818	-0.541279118	1.451003327	17.41137117	3.01E-05	0.000167947
ENSRNOG00000028638	-0.460736452	2.900818151	17.38303473	3.06E-05	0.00017023
ENSRNOG00000031459	-0.404550083	3.905249673	17.37584429	3.07E-05	0.000170815
ENSRNOG00000010018	-0.65077564	1.512159231	17.36711668	3.08E-05	0.000171481
ENSRNOG00000005223	-0.44436339	3.741448185	17.36370847	3.09E-05	0.000171668
ENSRNOG00000003964	-0.445026192	3.563844817	17.35593266	3.10E-05	0.000172311
ENSRNOG00000052062	-0.301966504	4.895643603	17.33924301	3.13E-05	0.000173648
ENSRNOG00000018023	-0.337275803	3.784393668	17.33365535	3.14E-05	0.000173999
ENSRNOG00000017233	-0.365099644	3.543355709	17.31823964	3.16E-05	0.000175332
ENSRNOG00000048288	-0.289764013	4.158012742	17.28796906	3.21E-05	0.000177898
ENSRNOG00000007512	-0.35035244	6.396559146	17.28501519	3.22E-05	0.00017805
ENSRNOG00000005623	-0.259410631	6.485348265	17.2667218	3.25E-05	0.000179647
ENSRNOG00000005374	-0.381788344	2.964127204	17.25361963	3.27E-05	0.000180827
ENSRNOG00000006545	-0.34285921	8.132472651	17.24834467	3.28E-05	0.000181203
ENSRNOG00000017380	-0.311623461	4.203132284	17.23785524	3.30E-05	0.000182143
ENSRNOG00000022597	-0.401372483	3.459942308	17.23485472	3.30E-05	0.000182367
ENSRNOG00000000541	-0.268410358	6.547050028	17.21900039	3.33E-05	0.000183831
ENSRNOG00000019273	-0.280135237	5.578873218	17.20596156	3.35E-05	0.000184968
ENSRNOG00000056243	-0.320856805	3.941356309	17.20191135	3.36E-05	0.000185234
ENSRNOG00000016620	-0.508612591	2.744818413	17.19640492	3.37E-05	0.000185707
ENSRNOG00000003599	-0.336534065	4.679427991	17.19489713	3.37E-05	0.00018579
ENSRNOG00000001416	-0.436640339	4.4423644	17.18007217	3.40E-05	0.000187115
ENSRNOG00000021355	-0.858039397	3.915668769	17.16915366	3.42E-05	0.000188063
ENSRNOG00000003545	-0.324827645	4.676770863	17.1386609	3.47E-05	0.000190908
ENSRNOG00000012980	-0.463698038	2.566591545	17.1140992	3.52E-05	0.000193191
ENSRNOG00000018272	-0.440437767	2.854832072	17.06084639	3.62E-05	0.000198067
ENSRNOG00000010353	-0.273726959	6.433377995	17.02902883	3.68E-05	0.000201275
ENSRNOG00000008643	-0.357539873	3.276360663	17.0132175	3.71E-05	0.000202748
ENSRNOG00000003975	-0.68667346	6.073301891	17.01088979	3.72E-05	0.000202919
ENSRNOG00000014935	-0.22948281	5.481068877	16.99998957	3.74E-05	0.000203884
ENSRNOG00000031053	-0.673628917	11.41394337	16.97531429	3.79E-05	0.00020648

ENSRNOG00000010126	-0.349685366	3.436228019	16.94175201	3.85E-05	0.000210018
ENSRNOG00000013841	-0.262942108	5.617301203	16.92798984	3.88E-05	0.000211324
ENSRNOG00000005496	-0.321703921	4.225521178	16.90203815	3.94E-05	0.000213796
ENSRNOG00000054352	-0.25014906	8.453320207	16.89169595	3.96E-05	0.00021489
ENSRNOG00000034191	-0.498771364	2.647955053	16.86757011	4.01E-05	0.000217415
ENSRNOG00000006355	-0.381559102	6.889052843	16.86688451	4.01E-05	0.000217417
ENSRNOG00000010993	-0.315179879	5.12846269	16.8625558	4.02E-05	0.000217618
ENSRNOG00000038761	-0.477570849	4.185776816	16.86061791	4.02E-05	0.000217766
ENSRNOG00000020975	-0.283913536	4.806344873	16.84681213	4.05E-05	0.000219281
ENSRNOG00000051996	-0.879309884	1.123688379	16.83544674	4.08E-05	0.000220522
ENSRNOG00000029061	-0.339367381	5.118457942	16.82555842	4.10E-05	0.000221599
ENSRNOG00000010438	-0.341508962	3.649872003	16.81919311	4.11E-05	0.000222267
ENSRNOG00000028688	-0.37271959	3.695044123	16.81177312	4.13E-05	0.000223062
ENSRNOG00000028381	-0.512091727	1.992462772	16.79116403	4.17E-05	0.000225344
ENSRNOG00000031599	-0.693662228	0.34189385	16.78986262	4.18E-05	0.000225421
ENSRNOG00000029682	-0.414985343	3.498613703	16.76769308	4.22E-05	0.000227915
ENSRNOG00000002960	-0.659110749	1.690872244	16.7665109	4.23E-05	0.000227979
ENSRNOG00000057941	-0.44770821	2.34589686	16.75322829	4.26E-05	0.000229425
ENSRNOG00000042878	-0.374032407	3.868489777	16.74728144	4.27E-05	0.000229988
ENSRNOG00000005141	-0.247834317	4.952907762	16.7340594	4.30E-05	0.000231439
ENSRNOG00000018021	-0.377881946	5.143827315	16.71982845	4.33E-05	0.000233023
ENSRNOG00000061442	-0.78910574	0.311962309	16.67687582	4.43E-05	0.000237875
ENSRNOG00000005765	-0.409883419	4.175827553	16.66330349	4.46E-05	0.000239445
ENSRNOG00000009484	-0.254749778	5.892696189	16.65266205	4.49E-05	0.000240686
ENSRNOG00000011821	-0.727099644	1.061214821	16.64926013	4.50E-05	0.000240955
ENSRNOG00000032376	-0.676813214	2.060359525	16.63284331	4.54E-05	0.000242967
ENSRNOG00000018053	-0.265500452	5.092868042	16.61604931	4.58E-05	0.000244838
ENSRNOG00000001271	-0.454071303	2.804079838	16.6066119	4.60E-05	0.000245935
ENSRNOG00000006285	-0.256003078	5.64433402	16.60242878	4.61E-05	0.000246394
ENSRNOG00000009851	-0.349858943	3.997699523	16.5788112	4.67E-05	0.000249061
ENSRNOG00000020777	-0.281996052	4.819126853	16.57640852	4.67E-05	0.000249293
ENSRNOG00000012469	-0.266145436	4.877368808	16.55305989	4.73E-05	0.000252297
ENSRNOG00000004901	-0.249661664	5.287084527	16.51862743	4.82E-05	0.000256574
ENSRNOG00000001424	-0.405776818	5.595633627	16.49006431	4.89E-05	0.000260293
ENSRNOG00000017758	-0.234179448	6.216355603	16.48562286	4.90E-05	0.000260816
ENSRNOG00000002450	-0.394106754	3.905425943	16.463685	4.96E-05	0.000263586
ENSRNOG00000000473	-0.380233585	5.45661801	16.45773198	4.97E-05	0.000264326
ENSRNOG00000060376	-0.660174473	0.666032641	16.44994814	5.00E-05	0.000265325
ENSRNOG00000031591	-0.504499501	2.010887367	16.44776045	5.00E-05	0.000265542
ENSRNOG00000054347	-0.817970843	-0.154662694	16.43868151	5.02E-05	0.000266727
ENSRNOG00000016330	-0.647011222	1.428042287	16.43095229	5.05E-05	0.000267638
ENSRNOG00000054607	-0.751043853	1.496562089	16.42432526	5.06E-05	0.000268485
ENSRNOG00000016511	-0.291667612	5.983837683	16.38155208	5.18E-05	0.000274245
ENSRNOG00000013234	-0.262679687	5.317874029	16.37994585	5.18E-05	0.000274294
ENSRNOG00000054904	-1.036219499	-1.094730209	16.36167421	5.23E-05	0.000276766
ENSRNOG00000045742	-0.440067686	2.322841747	16.32648602	5.33E-05	0.000281389
ENSRNOG00000021984	-0.329124717	5.892577679	16.32217623	5.34E-05	0.000281842
ENSRNOG00000052327	-1.125364097	-1.207786031	16.32074514	5.35E-05	0.000281961
ENSRNOG00000019246	-0.509006594	5.046027271	16.31990423	5.35E-05	0.000281992
ENSRNOG00000036841	-0.325074146	5.55355771	16.31823674	5.35E-05	0.000282146
ENSRNOG00000032414	-0.379421922	4.52701595	16.3165673	5.36E-05	0.000282301
ENSRNOG00000010031	-0.639311704	4.379299879	16.31344033	5.37E-05	0.000282579
ENSRNOG00000018630	-0.55941521	10.29322459	16.30724716	5.39E-05	0.00028341
ENSRNOG00000000096	-0.461660333	4.277259317	16.3060383	5.39E-05	0.000283497
ENSRNOG00000050903	-1.280663981	-0.67689835	16.29975959	5.41E-05	0.000284232
ENSRNOG00000012716	-0.235559265	6.771188649	16.27767412	5.47E-05	0.000287295
ENSRNOG00000019007	-0.400304484	7.348073036	16.27629153	5.47E-05	0.000287409
ENSRNOG00000037356	-0.49452517	2.424398452	16.27132996	5.49E-05	0.000288067
ENSRNOG00000056410	-1.031564744	-0.836469303	16.23335096	5.60E-05	0.000293414
ENSRNOG00000008103	-0.303207434	9.244157414	16.22993058	5.61E-05	0.000293847
ENSRNOG00000023130	-0.439695816	3.216768653	16.19969733	5.70E-05	0.000298179
ENSRNOG00000056942	-0.955799467	-0.301402044	16.16411908	5.81E-05	0.000303432
ENSRNOG00000016383	-0.262035147	5.6731135	16.15698364	5.83E-05	0.000304376
ENSRNOG00000031667	-1.12651835	-0.189467023	16.15613277	5.83E-05	0.000304413
ENSRNOG00000059402	-0.412254426	5.450851361	16.15045185	5.85E-05	0.000305226
ENSRNOG00000015696	-0.305582779	4.450144304	16.14435916	5.87E-05	0.000306109
ENSRNOG00000016217	-0.316490527	4.163150772	16.07011015	6.10E-05	0.000317303
ENSRNOG00000030319	-0.558724861	1.503416256	16.05931068	6.14E-05	0.000319013
ENSRNOG00000046585	-0.364242074	4.926671525	16.05610455	6.15E-05	0.000319449
ENSRNOG00000014758	-0.469776904	7.248328024	16.045949	6.18E-05	0.000320956
ENSRNOG00000001489	-0.299177809	3.971744985	16.03359276	6.22E-05	0.000322846
ENSRNOG00000006485	-0.27249204	6.05380779	16.02939745	6.24E-05	0.00032335
ENSRNOG00000006806	-0.56076616	2.10448226	16.02549611	6.25E-05	0.000323911
ENSRNOG00000054871	-0.59475882	3.013381929	15.98925073	6.37E-05	0.00032974
ENSRNOG00000009233	-0.470944579	3.862809003	15.98567007	6.38E-05	0.000330257
ENSRNOG00000001931	-0.338278073	6.002922885	15.95749393	6.48E-05	0.00033499
ENSRNOG00000017165	-0.438993334	3.91996528	15.95647363	6.48E-05	0.000334995
ENSRNOG00000043441	-0.371394462	3.298980859	15.95553221	6.48E-05	0.000335009
ENSRNOG00000006392	-0.319089477	4.61275074	15.95408871	6.49E-05	0.000335155
ENSRNOG00000005344	-0.288202356	4.499995196	15.94212334	6.53E-05	0.000336841
ENSRNOG00000004207	-0.4008801	5.604305631	15.93036755	6.57E-05	0.000338719
ENSRNOG00000014765	-0.263815926	7.460047299	15.92054812	6.61E-05	0.000340259
ENSRNOG00000029220	-0.310861036	5.167786227	15.90704614	6.65E-05	0.000342514
ENSRNOG00000021433	-0.394991209	5.546191112	15.87735897	6.76E-05	0.000347322
ENSRNOG00000016133	-0.277515331	6.64586072	15.87023167	6.78E-05	0.000348407
ENSRNOG00000010795	-0.391820128	4.987054927	15.85865609	6.83E-05	0.000350317
ENSRNOG00000004449	-0.329118349	5.588232368	15.85504445	6.84E-05	0.000350759
ENSRNOG00000042951	-0.328998194	5.021204673	15.85518699	6.84E-05	0.000350759
ENSRNOG00000059110	-0.729288733	4.829864328	15.81000493	7.00E-05	0.000358743
ENSRNOG00000006705	-0.372988787	5.684900838	15.78613375	7.09E-05	0.00036318
ENSRNOG00000000881	-0.265329909	6.095254928	15.77579172	7.13E-05	0.000364935
ENSRNOG00000002212	-1.00647921	0.140145265	15.75422216	7.21E-05	0.000368925
ENSRNOG00000042372	-0.746715545	0.470610615	15.73290658	7.29E-05	0.000372941
ENSRNOG00000058681	-0.33251585	4.087816732	15.69599344	7.44E-05	0.000379555
ENSRNOG00000003983	-0.486667249	6.43724783	15.69385283	7.45E-05	0.000379827
ENSRNOG00000061248	-0.787637377	-0.206518292	15.6923814	7.45E-05	0.000379913
ENSRNOG00000015676	-0.301880538	5.567227224	15.68453159	7.48E-05	0.00038137
ENSRNOG00000057250	-0.529948523	1.711154648	15.67911533	7.50E-05	0.000382341
ENSRNOG00000006244	-0.384672609	4.662931022	15.64813036	7.63E-05	0.000388406
ENSRNOG00000002320	-0.259915132	5.603520575	15.64553956	7.64E-05	0.000388814
ENSRNOG00000008794	-0.303737238	6.09066352	15.63860579	7.67E-05	0.000389991

ENSRNOG00000022234	-0.309338296	4.941799453	15.61963908	7.74E-05	0.00039367
ENSRNOG00000001037	-0.403286681	4.197694667	15.60915283	7.79E-05	0.000395605
ENSRNOG00000046719	-0.98613605	-1.108606724	15.59681496	7.84E-05	0.000397939
ENSRNOG00000012828	-0.218254594	5.130148011	15.59055001	7.86E-05	0.000399132
ENSRNOG00000003643	-0.371946091	3.14219104	15.58094043	7.90E-05	0.000400909
ENSRNOG00000001816	-0.478420761	3.039237646	15.57077193	7.95E-05	0.000402684
ENSRNOG00000038239	-0.388484209	3.380103997	15.56280115	7.98E-05	0.000404126
ENSRNOG00000011553	-0.242281055	4.811643893	15.55132132	8.03E-05	0.000406458
ENSRNOG00000013516	-0.309164585	8.535411271	15.53917104	8.08E-05	0.000408903
ENSRNOG00000015577	-0.528783542	1.990519937	15.52344309	8.15E-05	0.000412101
ENSRNOG00000033133	-0.315235519	4.948890203	15.49989378	8.25E-05	0.000417
ENSRNOG00000057682	-0.983645255	-1.138358862	15.49761093	8.26E-05	0.000417371
ENSRNOG00000059043	-0.255022302	5.9114768	15.47999112	8.34E-05	0.000420877
ENSRNOG00000054669	-0.29666349	5.343072187	15.46646286	8.40E-05	0.000423765
ENSRNOG00000061479	-1.1866508	1.307288219	15.44152783	8.51E-05	0.000429119
ENSRNOG00000013902	-0.528937435	5.146390768	15.42189536	8.60E-05	0.000432911
ENSRNOG00000017561	-0.309670521	3.386949681	15.41657271	8.62E-05	0.000433856
ENSRNOG00000001862	-0.406775043	7.698841483	15.40103472	8.69E-05	0.000437161
ENSRNOG00000061433	-1.061118207	-1.157074236	15.39684436	8.71E-05	0.000437992
ENSRNOG00000014975	-0.342578795	6.593888277	15.3912791	8.74E-05	0.000439005
ENSRNOG00000000164	-0.297039174	7.065079159	15.38970585	8.75E-05	0.000439231
ENSRNOG00000048486	-0.950972324	-0.922394713	15.35470191	8.91E-05	0.000446877
ENSRNOG00000004973	-0.375965886	7.156682683	15.34926569	8.94E-05	0.000448023
ENSRNOG00000048577	-0.328581129	5.953144949	15.34794215	8.94E-05	0.000448195
ENSRNOG00000011976	-0.366018014	2.718339656	15.3275682	9.04E-05	0.000452482
ENSRNOG00000037911	-0.884778236	0.377023048	15.32669338	9.04E-05	0.000452548
ENSRNOG00000017595	-0.627789431	4.058510739	15.32528957	9.05E-05	0.000452742
ENSRNOG00000008507	-0.543860297	1.027400455	15.30248071	9.16E-05	0.000457952
ENSRNOG00000057054	-1.231715756	-0.140512229	15.29324842	9.20E-05	0.00046005
ENSRNOG00000060262	-0.26743042	6.765086457	15.28416947	9.25E-05	0.000461829
ENSRNOG00000029043	-0.280080351	5.216856192	15.28074036	9.27E-05	0.000462523
ENSRNOG00000003163	-0.325966663	7.314570757	15.27247772	9.31E-05	0.000464258
ENSRNOG00000004908	-0.286903118	6.109751338	15.26190795	9.36E-05	0.000466716
ENSRNOG00000016912	-0.268365255	6.77636869	15.25142182	9.41E-05	0.000469166
ENSRNOG00000012556	-0.437312758	3.845931561	15.2449358	9.44E-05	0.000470632
ENSRNOG00000022732	-0.296598083	4.801218989	15.22945129	9.52E-05	0.000474208
ENSRNOG00000007291	-0.341439023	3.494663731	15.21393348	9.60E-05	0.00047797
ENSRNOG00000027860	-0.266409506	6.644537061	15.20484187	9.65E-05	0.000480126
ENSRNOG00000004061	-0.299615294	6.561402045	15.1967316	9.69E-05	0.000482041
ENSRNOG00000013451	-0.293247735	6.672702523	15.13181247	0.000100259	0.00049812
ENSRNOG00000016886	-0.39945676	5.079570305	15.12867572	0.000100426	0.000498792
ENSRNOG00000006391	-0.268985903	5.526269575	15.11889657	0.000100948	0.000501068
ENSRNOG00000004517	-0.524426639	1.97129383	15.11753228	0.000101021	0.000501273
ENSRNOG00000017577	-0.331233481	3.892127619	15.10358666	0.00010177	0.000504673
ENSRNOG00000002615	-0.286111091	5.003487956	15.09449524	0.000102261	0.000506951
ENSRNOG00000032902	-0.415807191	2.893049003	15.08532386	0.000102759	0.000509101
ENSRNOG00000061795	-0.281613697	4.582417176	15.08458808	0.000102799	0.00050914
ENSRNOG00000011632	-0.271886765	7.584811668	15.07102063	0.000103541	0.000512493
ENSRNOG00000003291	-0.330346753	4.58388156	15.06648691	0.00010379	0.000513564
ENSRNOG00000014675	-0.238217537	5.177574758	15.03685399	0.000105432	0.000520715
ENSRNOG00000040406	-0.949445961	-1.172384634	15.03044188	0.000105791	0.000522324
ENSRNOG00000003269	-0.318226492	4.645402872	15.0181862	0.00010648	0.000525399
ENSRNOG00000052622	-0.720020018	3.301879803	15.00319296	0.000107329	0.000529095
ENSRNOG00000020317	-0.379645986	5.214706449	14.99696286	0.000107684	0.000530492
ENSRNOG00000000432	-0.539505314	3.617406805	14.98820282	0.000108185	0.000532486
ENSRNOG00000057756	-0.230854287	6.332960183	14.98368117	0.000108445	0.000533532
ENSRNOG00000010452	-0.329743927	6.890432662	14.98131951	0.000108581	0.000533934
ENSRNOG00000002394	-0.270145458	7.207326051	14.97855193	0.00010874	0.00053422
ENSRNOG00000011595	-0.475166164	2.473081454	14.96887855	0.000109299	0.000536633
ENSRNOG00000024309	-0.391925967	6.485549054	14.94510025	0.000110685	0.00054327
ENSRNOG00000055290	-1.020206085	-0.883222017	14.93380212	0.00011135	0.000546194
ENSRNOG00000006873	-0.715672674	1.255335123	14.9343171	0.00011132	0.000546194
ENSRNOG00000006783	-0.692676209	2.150770238	14.91359258	0.000112549	0.000551735
ENSRNOG00000014712	-0.440148	3.302829743	14.90670878	0.000112961	0.000553238
ENSRNOG00000000954	-0.28006474	4.501309836	14.88242843	0.000114424	0.000560058
ENSRNOG00000049681	-0.390728433	7.773011835	14.88036332	0.000114549	0.000560498
ENSRNOG00000004273	-1.299327139	-0.30110447	14.86374138	0.000115563	0.000564935
ENSRNOG00000004316	-0.796526327	0.417411819	14.85013904	0.000116399	0.000568673
ENSRNOG00000048267	-0.237797566	5.196691175	14.82898968	0.000117712	0.000574377
ENSRNOG00000020624	-0.227277826	5.627813995	14.82461557	0.000117985	0.000575533
ENSRNOG00000043171	-0.229960366	5.781843797	14.82360274	0.000118049	0.000575665
ENSRNOG00000024793	-0.324426217	4.049272139	14.81262256	0.000118738	0.000578343
ENSRNOG00000006500	-0.27072282	5.696336682	14.81252706	0.000118744	0.000578343
ENSRNOG00000011647	-0.876856001	1.462737921	14.80991548	0.000118909	0.000578967
ENSRNOG00000005310	-0.25515041	7.336873599	14.80354457	0.000119311	0.00058039
ENSRNOG00000046940	-0.770853169	1.092345927	14.80366583	0.000119303	0.00058039
ENSRNOG00000002032	-0.225162348	5.092599849	14.79064981	0.00012013	0.000584014
ENSRNOG00000021644	-0.634603754	4.006320469	14.76790225	0.000121588	0.000590196
ENSRNOG00000004572	-0.411316698	2.653335059	14.76286545	0.000121913	0.000591593
ENSRNOG00000015526	-1.132906811	-1.107501994	14.75289937	0.000122559	0.000594182
ENSRNOG00000018886	-0.37155815	2.406805992	14.72758007	0.000124216	0.000601662
ENSRNOG00000001597	-0.285985315	6.289868699	14.72227293	0.000124566	0.000602989
ENSRNOG00000007838	-0.233691943	5.499103898	14.70571423	0.000125665	0.000607937
ENSRNOG00000006027	-0.296519358	6.802117527	14.70081531	0.000125992	0.000609147
ENSRNOG00000007241	-0.278188692	5.587057402	14.69050214	0.000126683	0.000612302
ENSRNOG00000019890	-0.697506295	-0.007162945	14.66817466	0.000128193	0.000618525
ENSRNOG00000013386	-0.290844976	5.97495298	14.66799044	0.000128205	0.000618525
ENSRNOG00000034272	-0.395703605	5.014574571	14.66671722	0.000128292	0.000618755
ENSRNOG00000047447	-0.946356751	0.141204334	14.65868483	0.00012884	0.000621208
ENSRNOG00000015560	-0.367281953	5.35314769	14.64265623	0.00012994	0.000625941
ENSRNOG00000027026	-0.356276792	3.907914799	14.63407913	0.000130533	0.000628223
ENSRNOG00000008222	-0.323638894	4.600850973	14.62401265	0.000131232	0.000631204
ENSRNOG00000055496	-0.315266359	3.501854406	14.61960798	0.000131539	0.000632489
ENSRNOG00000019450	-0.267517066	6.178172915	14.61306994	0.000131996	0.000634301
ENSRNOG00000061312	-0.27717764	5.120524398	14.59250804	0.000133444	0.000640677
ENSRNOG00000012788	-0.252934702	5.81264731	14.59265411	0.000133434	0.000640677
ENSRNOG00000006197	-0.243034078	6.538327554	14.5888812	0.000133701	0.000641522
ENSRNOG00000058461	-0.293560562	7.188600363	14.5784857	0.000134441	0.000644485
ENSRNOG00000011158	-0.422262856	6.924192824	14.57776859	0.000134492	0.000644535

ENSRNOG00000018885	-0.6522862	1.363224304	14.56433619	0.000135454	0.000648754
ENSRNOG00000021005	-0.284070441	4.098586045	14.56155283	0.000135654	0.000649516
ENSRNOG00000053813	-0.316972311	3.845673829	14.52727894	0.000138145	0.00066124
ENSRNOG00000037214	-0.462152569	4.245258347	14.49889078	0.000140242	0.000671077
ENSRNOG00000029686	-0.64709667	-0.082237808	14.49540022	0.000140502	0.000671915
ENSRNOG00000049308	-0.744226919	-0.179739459	14.49117959	0.000140817	0.000673219
ENSRNOG00000006796	-0.345708643	4.758843832	14.4868324	0.000141143	0.000674571
ENSRNOG00000040201	-0.851948713	5.14922824	14.48303345	0.000141428	0.000675729
ENSRNOG00000020668	-0.24429552	6.517994039	14.47702125	0.00014188	0.00067748
ENSRNOG00000042283	-0.307694427	4.066946845	14.46924402	0.000142467	0.000679873
ENSRNOG00000013252	-0.36981178	3.666047852	14.44366241	0.000144415	0.000688548
ENSRNOG00000008277	-0.279890024	5.308035148	14.44192462	0.000144548	0.000688976
ENSRNOG00000009430	-0.322626236	5.370793836	14.43650607	0.000144965	0.000690337
ENSRNOG00000002391	-0.727611808	1.367495177	14.43706893	0.000144921	0.000690337
ENSRNOG00000009977	-0.251179609	7.197839977	14.43157729	0.000145345	0.000691938
ENSRNOG00000007114	-0.354681304	6.873149459	14.42388124	0.00014594	0.000694354
ENSRNOG00000015230	-0.283015435	4.380414958	14.40119783	0.000147708	0.000702557
ENSRNOG00000058962	-0.661791315	1.756475392	14.39672656	0.000148059	0.000704016
ENSRNOG00000000634	-0.429489971	4.000519775	14.38210891	0.000149213	0.000709077
ENSRNOG00000010572	-0.348842818	3.581610437	14.3756138	0.000149729	0.000710923
ENSRNOG00000017829	-0.28740211	6.062028443	14.3681855	0.000150321	0.000713269
ENSRNOG00000026845	-0.661156845	0.978985986	14.3573432	0.000151189	0.000716959
ENSRNOG00000012734	-0.299012895	4.264217219	14.34901143	0.000151859	0.000719707
ENSRNOG00000052142	-0.285965158	4.569793633	14.34060878	0.000152539	0.00072271
ENSRNOG00000028380	-0.245615733	4.42438797	14.33346432	0.000153119	0.000725241
ENSRNOG00000029875	-0.394375822	5.149689224	14.33135111	0.000153291	0.000725838
ENSRNOG00000001811	-0.300293982	5.636965752	14.28381975	0.00015721	0.000742724
ENSRNOG00000000303	-0.392272264	3.054131941	14.27300285	0.000158116	0.000746234
ENSRNOG00000010839	-0.348003866	4.715847799	14.22113065	0.000162535	0.000766403
ENSRNOG00000039777	-0.313465511	4.097679444	14.21932118	0.000162691	0.000766912
ENSRNOG00000058890	-0.767993176	1.389861169	14.21818334	0.00016279	0.000767147
ENSRNOG00000013370	-0.358908837	3.346942981	14.20309562	0.0001641	0.000773093
ENSRNOG00000004628	-0.357572613	6.459909377	14.19716333	0.000164618	0.000775303
ENSRNOG00000028092	-0.809119656	1.947045828	14.18432942	0.000165745	0.000780377
ENSRNOG00000012153	-0.224391156	6.263068782	14.18163033	0.000165983	0.000781265
ENSRNOG00000010125	-0.271453235	5.98344587	14.17528468	0.000166544	0.000783671
ENSRNOG00000021323	-0.537950695	0.928782679	14.1516782	0.000168647	0.000792859
ENSRNOG00000049662	-0.463007609	2.399354199	14.14423117	0.000169315	0.000795768
ENSRNOG00000013217	-0.287463276	5.460333193	14.13523447	0.000170127	0.000798448
ENSRNOG00000020678	-0.42201658	2.466531245	14.13455849	0.000170188	0.000798448
ENSRNOG00000004143	-0.229599949	6.18611194	14.12849273	0.000170738	0.000800789
ENSRNOG00000049259	-0.271881508	3.894338515	14.12430614	0.000171118	0.00080199
ENSRNOG00000007827	-0.742998288	0.265858368	14.12400355	0.000171146	0.00080199
ENSRNOG00000027850	-0.624902075	0.907633954	14.12044315	0.00017147	0.000803271
ENSRNOG00000004214	-0.441160514	7.78913757	14.10588746	0.000172802	0.000808554
ENSRNOG00000018898	-0.382275023	5.051073751	14.09655442	0.000173662	0.000811617
ENSRNOG00000056870	-0.251560508	9.447040751	14.091925	0.00017409	0.000813377
ENSRNOG00000016919	-0.290387682	6.370896113	14.08732807	0.000174516	0.000814974
ENSRNOG00000047517	-0.448536491	2.530916648	14.08712619	0.000174534	0.000814974
ENSRNOG00000059538	-0.453068181	4.216159036	14.0861944	0.000174621	0.000815137
ENSRNOG00000054385	-0.453466077	2.443596058	14.08107114	0.000175097	0.00081712
ENSRNOG00000042195	-0.311802401	7.028387479	14.05844947	0.000177216	0.00082642
ENSRNOG00000005009	-0.344961858	3.110135425	14.05513264	0.000177529	0.000827248
ENSRNOG00000039504	-0.838140121	1.055079887	14.04812579	0.000178191	0.000829604
ENSRNOG00000010800	-0.267295514	7.159509856	14.01535248	0.000181324	0.000843445
ENSRNOG00000055845	-0.7117995	0.797597818	14.01231272	0.000181617	0.000844065
ENSRNOG00000061005	-0.686625618	2.550995594	14.01016605	0.000181825	0.000844782
ENSRNOG00000019463	-0.387236192	2.581414815	14.00039839	0.000182772	0.000848683
ENSRNOG00000004923	-0.251251528	5.779589124	13.99954599	0.000182855	0.000848819
ENSRNOG00000010930	-0.272406437	4.757306993	13.99620108	0.00018318	0.000849821
ENSRNOG00000011222	-0.410011918	7.537505649	13.99567464	0.000183232	0.000849821
ENSRNOG00000051251	-0.642440638	1.552438751	13.9733531	0.00018542	0.000859467
ENSRNOG00000020076	-0.229858993	5.814348135	13.9692868	0.000185821	0.000860824
ENSRNOG00000029608	-0.5686132	5.344269883	13.95399318	0.000187339	0.000867348
ENSRNOG00000011528	-0.322195667	3.572341421	13.95239302	0.000187499	0.000867833
ENSRNOG00000021027	-0.37890687	6.445399803	13.93262418	0.000189481	0.000876495
ENSRNOG00000014605	-0.302524681	4.365781777	13.92491449	0.00019026	0.000879583
ENSRNOG00000009894	-0.362130906	5.368326185	13.91285797	0.000191484	0.000884727
ENSRNOG00000008767	-0.258853229	4.336449653	13.90855385	0.000191923	0.000886496
ENSRNOG00000033351	-0.32076472	4.239895898	13.89157213	0.000193665	0.000894021
ENSRNOG00000028411	-0.317193096	6.160932653	13.88853946	0.000193978	0.000894943
ENSRNOG00000020721	-0.534723936	1.902734947	13.88623237	0.000194216	0.000895781
ENSRNOG00000013872	-0.572382413	1.490781668	13.87341873	0.000195545	0.000901384
ENSRNOG00000050602	-0.738142477	1.856408033	13.85847883	0.000197105	0.000906994
ENSRNOG00000011756	-0.296376069	6.555441117	13.85782558	0.000197174	0.000907046
ENSRNOG00000012392	-0.404828225	5.363901775	13.85501941	0.000197469	0.000908138
ENSRNOG00000047620	-0.251907053	5.111189099	13.84774729	0.000198234	0.000911129
ENSRNOG00000004510	-0.237000686	5.57211337	13.84322414	0.000198712	0.000912531
ENSRNOG00000060103	-0.447820355	4.28057292	13.83134788	0.000199972	0.000917784
ENSRNOG00000017730	-0.233911105	7.575612536	13.82973884	0.000200143	0.000918304
ENSRNOG00000019070	-0.263982132	6.912302191	13.82653142	0.000200485	0.000919606
ENSRNOG00000053570	-0.683572887	-0.083615346	13.80009989	0.000203325	0.000931016
ENSRNOG00000053070	-0.533614084	1.256173843	13.79834561	0.000203515	0.000931617
ENSRNOG00000027332	-0.392362219	2.57120885	13.75283114	0.000208506	0.000953361
ENSRNOG00000052051	-0.538675549	4.194507478	13.74694936	0.00020916	0.000955523
ENSRNOG00000021705	-0.412108843	3.961547429	13.74156467	0.000209761	0.00095748
ENSRNOG00000046037	-0.446290372	1.197011591	13.73961842	0.000209978	0.000958154
ENSRNOG00000005981	-0.250007708	5.218738169	13.73792424	0.000210168	0.000958466
ENSRNOG00000061993	-0.629303497	1.604653934	13.73201488	0.00021083	0.000961209
ENSRNOG00000017874	-0.472832106	3.619287467	13.71923582	0.000212269	0.000967493
ENSRNOG00000032777	-0.919502513	0.901330262	13.71331795	0.000212939	0.000970267
ENSRNOG00000018698	-0.230303025	6.637991481	13.7075932	0.000213589	0.000972949
ENSRNOG00000052004	-0.293953899	4.792901982	13.70625957	0.000213741	0.00097336
ENSRNOG00000038372	-0.228578652	7.641786133	13.70272517	0.000214144	0.000974913
ENSRNOG00000008672	-0.280824021	6.762305081	13.69510048	0.000215015	0.000978598
ENSRNOG00000053270	-0.472576125	3.346428335	13.68446728	0.000216236	0.000983872
ENSRNOG00000010543	-0.271340095	6.560535516	13.66816418	0.000218121	0.000991311
ENSRNOG00000030021	-0.837971753	2.594790537	13.66053605	0.000219009	0.000994623
ENSRNOG00000024545	-0.55937503	0.801657776	13.64818784	0.000220454	0.001000192
ENSRNOG00000002991	-0.232547396	5.652395019	13.64015807	0.000221399	0.001003328

ENSRNOG00000004962	-0.265809135	5.012492053	13.63724358	0.000221743	0.001004599
ENSRNOG00000032723	-0.283538498	3.9641497	13.62082504	0.00022369	0.001012267
ENSRNOG00000021793	-0.272412353	4.720588614	13.62084248	0.000223688	0.001012267
ENSRNOG00000020554	-0.503051287	3.346619745	13.61921244	0.000223883	0.001012845
ENSRNOG00000000585	-0.284196097	7.569267367	13.61559922	0.000224314	0.001014216
ENSRNOG00000054302	-0.604662504	0.859498308	13.60017618	0.000226164	0.001022291
ENSRNOG00000007023	-0.338578676	3.513163904	13.59258223	0.000227081	0.001025849
ENSRNOG00000005841	-0.273382419	5.060544195	13.57443511	0.000229287	0.001034929
ENSRNOG00000017832	-0.230456113	6.08519354	13.55654057	0.000231483	0.00104395
ENSRNOG00000037661	-0.529189923	3.959318022	13.54736641	0.000232618	0.001048468
ENSRNOG00000040213	-0.436449952	1.499881523	13.51352139	0.000236851	0.00106603
ENSRNOG00000012166	-0.424260153	3.126381689	13.49551773	0.000239134	0.001075695
ENSRNOG00000026085	-0.423933517	3.358573912	13.49298316	0.000239457	0.001076843
ENSRNOG00000016754	-0.269292957	3.798282542	13.48982062	0.000239861	0.001078354
ENSRNOG00000017744	-0.313622506	3.616195427	13.46606126	0.000242918	0.001090547
ENSRNOG00000010668	-0.316641438	6.454444162	13.46231066	0.000243404	0.001092419
ENSRNOG00000010232	-0.268357243	8.252033041	13.44590047	0.000245542	0.001100457
ENSRNOG00000014564	-0.2350288	5.834224283	13.43930696	0.000246407	0.001103707
ENSRNOG00000018741	-0.252455648	5.723336812	13.43423765	0.000247073	0.001106067
ENSRNOG00000014521	-0.274323681	5.838488008	13.42041435	0.000248901	0.001113933
ENSRNOG00000011613	-0.273503537	5.988290176	13.41554198	0.000249548	0.001116514
ENSRNOG00000009808	-0.307668854	5.423097324	13.40386975	0.000251106	0.001122215
ENSRNOG00000000611	-0.264726772	4.870395555	13.39963774	0.000251673	0.001124432
ENSRNOG00000032552	-0.315577737	3.621184922	13.39068262	0.000252877	0.001128858
ENSRNOG00000038740	-0.707771491	-0.219340933	13.38114288	0.000254167	0.001133975
ENSRNOG00000013280	-0.638461102	1.217178381	13.35844361	0.000257262	0.001146168
ENSRNOG00000031590	-0.832448805	-0.718764214	13.35199091	0.000258148	0.001149533
ENSRNOG00000027088	-0.261277959	7.062256023	13.35189082	0.000258162	0.001149533
ENSRNOG00000047303	-0.479575917	2.676037023	13.35009194	0.00025841	0.001150313
ENSRNOG00000002054	-0.260013337	5.011407931	13.34724954	0.000258802	0.001151734
ENSRNOG00000016942	-0.223832949	6.42500648	13.34083104	0.000259689	0.001155194
ENSRNOG00000062248	-0.508776661	1.56150996	13.34056984	0.000259725	0.001155194
ENSRNOG00000059746	-0.347127043	2.948082103	13.32921891	0.000261302	0.001161556
ENSRNOG00000010461	-0.431022787	3.758685117	13.32851347	0.000261401	0.001161667
ENSRNOG00000013223	-0.521925151	5.072857652	13.32691157	0.000261624	0.001162333
ENSRNOG00000023220	-0.249708478	4.014136849	13.32490155	0.000261905	0.001163253
ENSRNOG00000027888	-0.391723394	3.505020718	13.32039618	0.000262535	0.001165725
ENSRNOG00000005470	-0.459498152	4.711167152	13.31880011	0.000262758	0.00116639
ENSRNOG00000005176	-0.267684731	7.215157262	13.31606911	0.000263141	0.001167763
ENSRNOG00000009845	-0.280921111	5.043564505	13.29983892	0.000265429	0.001176926
ENSRNOG00000056026	-0.595216089	0.705050413	13.29465291	0.000266164	0.001179525
ENSRNOG00000012650	-0.268370912	5.530378119	13.29127944	0.000266643	0.001181246
ENSRNOG00000021442	-0.331415305	3.173835032	13.28918916	0.000266941	0.001181411
ENSRNOG00000017273	-0.56526429	0.841602604	13.28983221	0.000266849	0.001181411
ENSRNOG00000013798	-0.235721878	6.661175454	13.27414173	0.000269092	0.001189818
ENSRNOG00000004737	-0.533652951	4.57667834	13.2578427	0.000271442	0.001199038
ENSRNOG00000047957	-0.566155193	0.20073561	13.25147521	0.000272365	0.001202025
ENSRNOG00000002210	-0.403722286	5.007549271	13.24987466	0.000272598	0.001202317
ENSRNOG00000021823	-0.586937458	1.148333046	13.23684556	0.000274499	0.001210029
ENSRNOG00000001609	-0.337646871	4.110405573	13.21929639	0.000277082	0.001219714
ENSRNOG00000038102	-0.296165033	3.9396275	13.21662876	0.000277476	0.001221111
ENSRNOG00000012899	-0.336102277	4.17216939	13.21454343	0.000277785	0.001222131
ENSRNOG00000013336	-0.290902413	4.203492875	13.15799399	0.000286295	0.001254691
ENSRNOG00000002940	-0.250912428	7.648940926	13.15839419	0.000286234	0.001254691
ENSRNOG00000019108	-0.312116618	4.693598828	13.15596427	0.000286606	0.001255703
ENSRNOG00000002275	-0.257134952	5.565499624	13.11608124	0.000292772	0.001280947
ENSRNOG00000027864	-0.293934696	9.201571572	13.10497561	0.000294513	0.001287496
ENSRNOG00000018645	-0.45573973	8.833847268	13.08506909	0.000297659	0.00130031
ENSRNOG00000008613	-0.309235563	4.838905981	13.07118063	0.000299874	0.001309489
ENSRNOG00000035576	-1.072082709	-0.666719047	13.0685902	0.000300289	0.001310939
ENSRNOG00000018531	-0.637077299	4.506283697	13.06562047	0.000300766	0.001311951
ENSRNOG00000029756	-0.689525649	3.07573968	13.06613424	0.000300683	0.001311951
ENSRNOG00000025764	-0.778660733	2.493827619	13.06559664	0.000300769	0.001311951
ENSRNOG00000057925	-0.663537546	-0.195581362	13.06081344	0.000301538	0.00131422
ENSRNOG00000014589	-0.288606901	3.656015345	13.05367771	0.000302689	0.001318148
ENSRNOG00000027628	-0.243148919	4.545976019	13.05415049	0.000302613	0.001318148
ENSRNOG00000006541	-0.253822582	4.17540597	13.04689906	0.000303787	0.001322563
ENSRNOG00000038616	-0.702956786	0.317118735	13.04490619	0.00030411	0.001323607
ENSRNOG00000002100	-0.24100845	6.049106266	13.04417367	0.000304229	0.001323761
ENSRNOG00000004755	-0.285875324	4.996189153	13.03349307	0.000305969	0.001329871
ENSRNOG00000050413	-0.436178271	1.466574025	13.02157586	0.000307923	0.001337924
ENSRNOG00000027422	-0.249105254	4.185034378	13.01067189	0.000309721	0.001344699
ENSRNOG00000026644	-0.491319158	2.458230808	13.00310435	0.000310975	0.001349775
ENSRNOG00000054866	-0.520186342	3.136387372	12.99786895	0.000311846	0.001352812
ENSRNOG00000011904	-0.275264992	4.751706978	12.97782265	0.000315202	0.001366625
ENSRNOG00000056558	-0.253490947	6.215633104	12.97165531	0.000316242	0.001370383
ENSRNOG00000017294	-0.282130133	5.038078271	12.94286674	0.000321143	0.001390858
ENSRNOG00000014173	-0.284150336	6.430716708	12.93949393	0.000321722	0.001392223
ENSRNOG00000010282	-0.878569484	3.100074759	12.93975331	0.000321677	0.001392223
ENSRNOG00000001269	-0.492335991	8.035400256	12.94007758	0.000321622	0.001392223
ENSRNOG00000003958	-0.297427118	4.90103542	12.93002529	0.000323353	0.001398519
ENSRNOG00000007247	-0.846391739	0.018604291	12.92513257	0.0003242	0.001401779
ENSRNOG00000002256	-0.847097219	2.910064139	12.92098031	0.00032492	0.001404142
ENSRNOG00000022162	-0.444439802	5.907066542	12.91920575	0.000325228	0.001405091
ENSRNOG00000018701	-0.55588293	1.665711433	12.9092295	0.000326966	0.001412214
ENSRNOG00000048309	-0.270620563	6.011492486	12.90165564	0.000328292	0.001417554
ENSRNOG00000011865	-0.302441234	4.318261807	12.90108063	0.000328392	0.001417603
ENSRNOG00000027317	-0.281923879	6.99761987	12.89114541	0.00033014	0.001424759
ENSRNOG00000001789	-0.375765175	5.717319866	12.87781761	0.0003325	0.001433769
ENSRNOG00000061748	-0.359694522	2.469397369	12.87365491	0.00033324	0.001436571
ENSRNOG00000021718	-0.287316313	5.115720021	12.86857863	0.000334145	0.001439689
ENSRNOG00000053852	-0.589854408	1.141217036	12.86558586	0.00033468	0.001441601
ENSRNOG00000004577	-0.228387316	4.604637577	12.85475459	0.000336623	0.001448787
ENSRNOG00000014323	-0.218327218	5.823446251	12.85334056	0.000336877	0.001449094
ENSRNOG00000007315	-0.314253639	5.46405569	12.84646972	0.000338117	0.001452846
ENSRNOG00000054891	-0.373304639	5.755183833	12.8452125	0.000338344	0.001453428
ENSRNOG00000021317	-0.282828594	7.178342536	12.8365236	0.000339919	0.001459797
ENSRNOG00000018351	-0.31112696	4.529775618	12.83290193	0.000340577	0.001462228
ENSRNOG00000025580	-0.420926718	5.507015614	12.81948905	0.000343027	0.001471551
ENSRNOG00000003029	-0.3024811	8.56814982	12.81777823	0.000343341	0.001472499

ENSRNOG00000051278	-0.385631195	1.578483934	12.81710558	0.000343465	0.001472629
ENSRNOG00000001783	-0.312832251	6.489044294	12.81499058	0.000343853	0.001473497
ENSRNOG00000008912	-0.247242818	5.87496981	12.81185589	0.00034443	0.001475568
ENSRNOG00000013852	-0.239816582	5.031934822	12.80166698	0.000346311	0.001483225
ENSRNOG00000010753	-0.240372659	5.400328871	12.78623005	0.00034918	0.001493898
ENSRNOG00000000812	-0.48371134	5.197820107	12.77841771	0.000350641	0.001499745
ENSRNOG00000029038	-0.264126018	3.95220844	12.77439294	0.000351397	0.001502569
ENSRNOG00000010760	-0.383993278	3.018840914	12.76824595	0.000352553	0.001506701
ENSRNOG00000046357	-0.223256104	5.679297053	12.74899555	0.0003562	0.001521056
ENSRNOG00000005006	-0.258994655	4.088630107	12.74333798	0.000357279	0.001525253
ENSRNOG00000002934	-0.251488788	10.26972397	12.71932743	0.000361896	0.001542466
ENSRNOG00000060912	-0.207823562	6.12751609	12.71673028	0.000362399	0.001544194
ENSRNOG00000007069	-0.295180385	5.786147189	12.70698284	0.000364293	0.001551846
ENSRNOG00000009178	-0.726103474	-0.015826392	12.70297489	0.000365074	0.001554757
ENSRNOG00000025728	-0.854593059	0.927461792	12.69666548	0.000366308	0.001559173
ENSRNOG00000011308	-0.240469032	6.170589451	12.69319808	0.000366988	0.001561647
ENSRNOG00000007899	-0.376109263	3.005393546	12.67970943	0.000369645	0.001571686
ENSRNOG00000015619	-0.25674686	5.601212468	12.67871452	0.000369842	0.0015721
ENSRNOG00000003967	-0.297909154	6.682964252	12.66607131	0.000372351	0.001582342
ENSRNOG00000023484	-0.245850284	4.415971284	12.65634229	0.000374294	0.001588892
ENSRNOG00000017907	-0.282302694	5.379815689	12.65008576	0.000375548	0.001592937
ENSRNOG00000002205	-0.247520605	7.896808127	12.63950181	0.000377681	0.001601123
ENSRNOG00000056189	-0.327268553	3.46622097	12.629878	0.00037963	0.001608526
ENSRNOG00000017235	-0.241977911	8.052469799	12.62513784	0.000380594	0.001611316
ENSRNOG00000007874	-0.320683234	3.344574922	12.62160149	0.000381315	0.001613936
ENSRNOG00000030416	-0.346198424	3.268472442	12.61084783	0.000383515	0.00162238
ENSRNOG00000007098	-0.360149709	3.078796989	12.60698652	0.000384308	0.001625301
ENSRNOG00000011047	-0.22464262	4.790180725	12.60560583	0.000384592	0.001626068
ENSRNOG00000005100	-0.924070616	0.872508707	12.60277269	0.000385175	0.001628099
ENSRNOG00000043033	-0.353957952	2.713050994	12.60038888	0.000385667	0.001629742
ENSRNOG00000029209	-0.359667486	5.882270481	12.58637104	0.00038857	0.001641135
ENSRNOG00000054457	-0.596026852	0.45338822	12.58388021	0.000389088	0.001642886
ENSRNOG00000004874	-0.425815821	5.433091533	12.57798971	0.000390316	0.001646316
ENSRNOG00000011448	-0.325253643	4.559259038	12.57646673	0.000390635	0.001647219
ENSRNOG00000029966	-0.334549267	3.312287326	12.57516075	0.000390908	0.001647932
ENSRNOG00000018783	-0.216303781	5.141057254	12.56852552	0.000392298	0.001653353
ENSRNOG00000011555	-0.249281516	5.23549952	12.56651253	0.000392721	0.001654054
ENSRNOG00000051442	-0.375538965	3.282840317	12.55980193	0.000394134	0.001659322
ENSRNOG00000016396	-0.249274214	7.062529535	12.55328284	0.000395511	0.001664678
ENSRNOG00000013459	-0.383055586	3.370651642	12.55251765	0.000395673	0.001664917
ENSRNOG00000002751	-0.317141419	3.213692204	12.52921539	0.000400638	0.00168402
ENSRNOG00000034184	-0.308174804	2.697452482	12.52548479	0.000401439	0.00168649
ENSRNOG00000060950	-0.811766854	-0.284986022	12.51919636	0.000402792	0.001691727
ENSRNOG00000007817	-0.346613706	3.14592911	12.51004374	0.00040477	0.001698904
ENSRNOG00000017725	-0.573721524	2.532826828	12.49868734	0.000407238	0.001708587
ENSRNOG00000059886	-0.58696696	0.260891759	12.49451306	0.000408149	0.001711955
ENSRNOG00000056350	-0.666952441	0.247745708	12.47076988	0.00041337	0.001732935
ENSRNOG00000011535	-0.267703761	5.774190557	12.44542194	0.000419018	0.001755683
ENSRNOG00000060194	-0.689189649	1.034376186	12.44278632	0.000419609	0.001757697
ENSRNOG00000014064	-0.285621165	5.744198153	12.4283866	0.000422857	0.001770364
ENSRNOG00000051232	-0.297732151	4.786865645	12.42076819	0.000424586	0.001776662
ENSRNOG00000049489	-0.52122755	1.079006985	12.41884118	0.000425024	0.001778026
ENSRNOG00000058053	-0.267016809	5.690672699	12.40732367	0.000427653	0.001787609
ENSRNOG00000004521	-0.274280297	5.461062488	12.40333249	0.000428568	0.001790961
ENSRNOG00000048152	-0.362510578	4.57541088	12.39044775	0.000431536	0.001802886
ENSRNOG00000005486	-0.297338839	4.722081209	12.35767976	0.000439176	0.001833743
ENSRNOG00000019974	-0.502310307	7.567158272	12.35728415	0.000439269	0.001833743
ENSRNOG00000009533	-0.462874514	5.287076654	12.3379558	0.000443841	0.001851851
ENSRNOG00000011509	-0.416079724	4.773141716	12.33798229	0.000443834	0.001851851
ENSRNOG00000003515	-0.346396347	4.484125996	12.33126613	0.000445434	0.00185801
ENSRNOG00000057221	-0.351302223	8.023814958	12.32120843	0.000447841	0.001867064
ENSRNOG00000005021	-0.26533567	5.154511969	12.31818285	0.000448567	0.001869601
ENSRNOG00000030517	-0.389655615	2.453739536	12.30331673	0.000452154	0.001882568
ENSRNOG00000003223	-0.441659636	6.179446783	12.29528541	0.000454104	0.001889196
ENSRNOG00000013288	-0.235970941	7.137006689	12.29083288	0.000455189	0.001893211
ENSRNOG00000003346	-0.475962327	1.545010853	12.27072763	0.000460119	0.001911207
ENSRNOG00000023226	-0.597107129	3.658757583	12.26441186	0.000461679	0.001916681
ENSRNOG00000020929	-0.438372408	3.838452806	12.25816073	0.000463228	0.001922104
ENSRNOG00000059169	-0.777642697	-0.622852643	12.24277519	0.000467063	0.001936496
ENSRNOG00000014501	-0.300063256	7.283944967	12.23872904	0.000468077	0.001939684
ENSRNOG00000012512	-1.039816122	5.044206721	12.22812425	0.000470745	0.001949209
ENSRNOG00000012738	-0.263018375	6.364796705	12.21520555	0.000474016	0.001961727
ENSRNOG00000011478	-0.65036996	0.43863348	12.19244581	0.000479834	0.001983733
ENSRNOG00000016855	-0.315703337	5.012440064	12.163922	0.000487228	0.002010102
ENSRNOG00000009886	-0.444333178	3.138665801	12.16201965	0.000487725	0.002011629
ENSRNOG00000000778	-0.393581487	2.395700367	12.15732098	0.000488956	0.002016178
ENSRNOG00000009156	-0.259826701	6.141824848	12.1559835	0.000489306	0.002017099
ENSRNOG00000007831	-0.25419105	5.379379774	12.14493973	0.000492212	0.002028022
ENSRNOG00000032735	-0.333384218	5.734561222	12.13869023	0.000493864	0.0020343
ENSRNOG00000002540	-0.866637845	-0.534388548	12.1357988	0.000494631	0.002036397
ENSRNOG00000011669	-0.440373758	1.471770641	12.1330022	0.000495373	0.002038923
ENSRNOG00000012733	-0.322164538	6.396891737	12.12336855	0.000497939	0.002048418
ENSRNOG00000008539	-0.916214686	-0.839303773	12.10926032	0.00050172	0.002062368
ENSRNOG00000007136	-0.221379045	5.661043287	12.109585	0.000501633	0.002062368
ENSRNOG00000011908	-0.338490461	4.639542584	12.10142394	0.000503833	0.002069978
ENSRNOG00000016360	-0.387918761	4.024220441	12.09745867	0.000504906	0.002073309
ENSRNOG00000059276	-0.385068143	2.198310274	12.07868643	0.000510015	0.002092661
ENSRNOG00000030410	-0.539745725	3.093241877	12.07807489	0.000510183	0.002092806
ENSRNOG00000013228	-0.429393827	5.054998963	12.07134915	0.000512026	0.002099825
ENSRNOG00000018403	-0.228672106	5.33700306	12.05159393	0.000517481	0.002118669
ENSRNOG00000024874	-0.44827395	4.949761141	12.04733625	0.000518664	0.00212265
ENSRNOG00000022347	-0.293131871	3.978748236	12.03485049	0.00052215	0.00213526
ENSRNOG00000050771	-0.328463417	3.595223404	12.03148046	0.000523095	0.002137468
ENSRNOG00000031032	-0.280977486	5.651346986	12.02456659	0.000525039	0.002144858
ENSRNOG00000001296	-0.734659137	3.610543627	12.02377073	0.000525263	0.002145062
ENSRNOG00000014529	-0.2635074	5.325785214	12.01868514	0.000526698	0.002149973
ENSRNOG00000012021	-0.198874422	4.860182913	12.01814304	0.000526852	0.002150044
ENSRNOG00000028176	-0.383550318	2.21403771	12.0114329	0.000528752	0.002157242
ENSRNOG00000022431	-0.399568587	2.29130212	11.9971299	0.000532825	0.002172183
ENSRNOG00000007206	-0.717594407	6.029923738	11.98992321	0.00053489	0.002179477

ENSRNOG00000003359	-0.314621836	8.310278029	11.98733737	0.000535633	0.00218082
ENSRNOG00000007753	-0.228934484	5.333885081	11.9790702	0.000538014	0.002189578
ENSRNOG00000030443	-0.280687502	4.511595807	11.97740966	0.000538494	0.002190295
ENSRNOG00000025011	-0.262551428	6.870664867	11.96523382	0.000542024	0.002203443
ENSRNOG00000019980	-0.476355455	7.351487981	11.96210417	0.000542935	0.00220658
ENSRNOG00000017222	-0.25018975	5.051485551	11.94025223	0.00054934	0.00222975
ENSRNOG00000023533	-0.256261209	5.376625877	11.93580739	0.000550653	0.002234006
ENSRNOG00000023187	-0.299204152	4.334008685	11.93574471	0.000550671	0.002234006
ENSRNOG00000006941	-0.271722786	5.162527366	11.93269683	0.000551573	0.002237091
ENSRNOG00000061412	-0.746856582	-0.501263933	11.92933275	0.00055257	0.00224056
ENSRNOG00000054155	-0.454907949	3.188033133	11.91571552	0.000556624	0.002255843
ENSRNOG00000017745	-0.198268363	5.96889121	11.87031589	0.000570357	0.002307958
ENSRNOG00000029844	-1.490029452	-1.073039966	11.86142834	0.000573086	0.002317813
ENSRNOG00000020915	-0.23937652	6.61134061	11.84231269	0.000578999	0.002338145
ENSRNOG00000003954	-0.557360221	0.384672645	11.84155739	0.000579234	0.002338497
ENSRNOG00000015967	-0.280288596	5.585629068	11.83761067	0.000580463	0.002341667
ENSRNOG00000049128	-0.2882443	6.574088104	11.83440615	0.000581462	0.002345103
ENSRNOG00000038819	-0.63335107	1.269677795	11.8271647	0.000583728	0.002353642
ENSRNOG00000009208	-0.317107582	3.598845593	11.82117843	0.000585608	0.002359248
ENSRNOG00000020782	-0.279209497	5.596840634	11.81800785	0.000586606	0.002361637
ENSRNOG00000006412	-0.290228205	6.667510533	11.7980489	0.000592928	0.002386484
ENSRNOG00000025489	-0.274960681	4.818071248	11.78318459	0.000597682	0.002403782
ENSRNOG00000059556	-0.841099102	-1.030448095	11.76532265	0.000603444	0.00242388
ENSRNOG00000001323	-0.288995139	4.804389425	11.76375699	0.000603952	0.002425305
ENSRNOG00000011979	-0.360525984	3.603711833	11.76094498	0.000604865	0.002428355
ENSRNOG00000017951	-0.27174504	5.603097483	11.67127441	0.000634725	0.002535378
ENSRNOG00000051364	-0.786346897	-0.840693124	11.66683143	0.000636243	0.002540799
ENSRNOG00000047205	-0.539402665	3.7128925	11.66059405	0.00063838	0.002547405
ENSRNOG00000046002	-0.308480168	6.09878808	11.6503523	0.000641904	0.002558888
ENSRNOG00000011879	-0.230810411	6.83631997	11.65039904	0.000641888	0.002558888
ENSRNOG00000055990	-0.504421891	1.895780953	11.64371325	0.000644199	0.002566099
ENSRNOG00000019971	-0.257379332	4.648924157	11.63627404	0.00064678	0.002575086
ENSRNOG00000046132	-0.61343549	1.737480959	11.63490188	0.000647258	0.002576339
ENSRNOG00000038025	-0.404825484	2.352537005	11.62566008	0.000650482	0.00258722
ENSRNOG00000030049	-0.283250494	5.047963251	11.60860652	0.000656473	0.002607381
ENSRNOG00000012472	-0.28797434	3.368192108	11.608995	0.000656336	0.002607381
ENSRNOG00000050180	-0.291482116	3.932918466	11.59456534	0.000661448	0.002626222
ENSRNOG00000013572	-0.443285906	4.781532223	11.58314228	0.000665524	0.002641079
ENSRNOG00000039971	-0.681904975	0.242030793	11.57436831	0.000668671	0.002652905
ENSRNOG00000014240	-0.38065053	1.814047108	11.57240782	0.000669377	0.002655038
ENSRNOG00000020435	-0.318280796	4.481758563	11.56871635	0.000670707	0.002658982
ENSRNOG00000052699	-0.663064523	0.28963659	11.55746232	0.000674779	0.002670445
ENSRNOG00000047796	-0.418700409	2.990316294	11.55430077	0.000675927	0.002673653
ENSRNOG00000003786	-0.315199754	5.35329728	11.55435721	0.000675907	0.002673653
ENSRNOG00000058588	-0.257461094	4.356443588	11.55194347	0.000676785	0.002676376
ENSRNOG00000021475	-0.29254468	5.460654776	11.54657321	0.000678742	0.002683448
ENSRNOG00000025523	-0.274443184	4.090839941	11.53609659	0.000682578	0.002696592
ENSRNOG00000022953	-0.422600322	2.144357299	11.52531099	0.00068655	0.002710929
ENSRNOG00000027694	-0.46545113	2.297627119	11.52004587	0.000688497	0.002717941
ENSRNOG00000029893	-0.37573543	2.690481232	11.5146683	0.000690492	0.002723099
ENSRNOG00000010902	-0.223172921	5.240196428	11.51475417	0.00069046	0.002723099
ENSRNOG00000018345	-0.209082087	6.617608764	11.50904033	0.000692585	0.002729317
ENSRNOG00000050910	-0.911038266	-0.967473521	11.50635126	0.000693588	0.002732588
ENSRNOG00000021009	-0.380949838	2.447122243	11.49786944	0.00069676	0.002742356
ENSRNOG00000046889	-0.377781859	7.372322309	11.49791705	0.000696742	0.002742356
ENSRNOG00000000900	-0.257628686	4.963227189	11.49736386	0.00069695	0.002742421
ENSRNOG00000017453	-0.319513075	3.533502832	11.48930084	0.00069998	0.002753659
ENSRNOG00000017403	-0.443802326	1.644831345	11.48678069	0.000700929	0.002756026
ENSRNOG00000009303	-0.352165668	2.831930295	11.48078356	0.000703195	0.002764247
ENSRNOG00000035452	-1.071864231	-0.052846467	11.48022781	0.000703405	0.002764387
ENSRNOG00000041608	-0.432761768	2.00095057	11.47739566	0.000704478	0.002767593
ENSRNOG00000060175	-0.269665438	3.677534193	11.47715159	0.00070457	0.002767593
ENSRNOG00000016454	-0.308658343	4.533474725	11.47350035	0.000705956	0.002772348
ENSRNOG00000024230	-0.340184624	3.542648677	11.47053293	0.000707084	0.002776089
ENSRNOG00000001331	-0.189468218	5.989188032	11.44467049	0.000716993	0.002812206
ENSRNOG00000002814	-0.435019949	1.481049736	11.42660324	0.000723999	0.002838278
ENSRNOG00000043151	-0.37176046	2.721957363	11.42442461	0.000724849	0.002840905
ENSRNOG00000033099	-0.286509459	4.636679036	11.4185937	0.000727127	0.00284772
ENSRNOG00000026616	-0.332885564	5.347848329	11.41722598	0.000727663	0.002849112
ENSRNOG00000014272	-0.445206724	7.525010671	11.40020206	0.000734361	0.002872499
ENSRNOG00000043390	-0.255440374	5.581639458	11.39339088	0.000737059	0.002880934
ENSRNOG00000042833	-0.372056778	4.819194062	11.39337845	0.000737064	0.002880934
ENSRNOG00000001889	-0.243697225	4.81430668	11.3923834	0.000737459	0.002881056
ENSRNOG00000031325	-0.27397134	6.933105021	11.3885889	0.000738966	0.002884766
ENSRNOG00000019995	-0.230131254	5.623386484	11.38815999	0.000739137	0.002884766
ENSRNOG00000018410	-0.237560829	4.138700265	11.3737222	0.000744905	0.002903697
ENSRNOG00000008035	-0.301277761	5.089912323	11.36297684	0.000749226	0.002919107
ENSRNOG00000006646	-0.301566499	6.745193884	11.35991723	0.000750462	0.002923201
ENSRNOG00000032443	-1.082051454	-0.981832869	11.33061307	0.000762397	0.002967502
ENSRNOG00000061443	-0.49600986	0.640712173	11.31893868	0.000767206	0.002985483
ENSRNOG00000015989	-0.280133248	3.654810947	11.31167713	0.000770212	0.002996445
ENSRNOG00000031453	-0.251612836	5.805673015	11.3007521	0.000774757	0.003013388
ENSRNOG00000021528	-0.233357868	5.835530681	11.2871714	0.000780445	0.003034021
ENSRNOG00000011245	-0.35889709	4.485922529	11.28365743	0.000781924	0.003039023
ENSRNOG00000004425	-0.353234969	3.772524451	11.27599191	0.000785159	0.003050848
ENSRNOG00000011675	-0.393842869	4.486414977	11.27407035	0.000785972	0.00305251
ENSRNOG00000000729	-0.307359716	3.110087363	11.24101848	0.000800092	0.003103543
ENSRNOG00000036865	-0.459552047	3.538518436	11.2241558	0.000807395	0.003128804
ENSRNOG00000023577	-0.198255275	6.385920709	11.22186343	0.000808393	0.003131904
ENSRNOG00000057316	-0.762017844	-0.044319503	11.20904149	0.000813998	0.003151305
ENSRNOG00000032426	-0.576279069	0.525076001	11.19947218	0.000818206	0.003166049
ENSRNOG00000024213	-0.279253324	4.65571751	11.19727872	0.000819174	0.00316902
ENSRNOG00000020376	-0.243812037	3.689317759	11.19478987	0.000820273	0.003171723
ENSRNOG00000012136	-0.312802643	4.665037312	11.19492717	0.000820213	0.003171723
ENSRNOG00000009639	-0.383201422	5.841351803	11.18663876	0.000823885	0.003183355
ENSRNOG00000010957	-0.338578861	5.129055441	11.18614599	0.000824103	0.003183424
ENSRNOG00000048109	-0.395420979	2.635722557	11.18184305	0.000826017	0.003190037
ENSRNOG00000006583	-0.714679918	-0.358322988	11.16798757	0.000832208	0.003210816
ENSRNOG00000011782	-0.242909467	7.715632612	11.16673604	0.00083277	0.0032122
ENSRNOG00000061067	-0.703646522	-0.593872093	11.16127439	0.000835225	0.003220102

ENSRNOG00000051102	-0.590075671	1.135441243	11.14812808	0.000841165	0.003242212
ENSRNOG00000053061	-0.25369124	5.587228506	11.13694614	0.000846251	0.003260228
ENSRNOG00000026239	-0.297055766	5.705266075	11.13572995	0.000846806	0.003261573
ENSRNOG00000004145	-0.413331602	1.732954278	11.13371746	0.000847725	0.003264319
ENSRNOG00000012500	-0.299154798	4.92590209	11.12676989	0.000850906	0.003274506
ENSRNOG00000002585	-0.248464695	6.02696908	11.12516199	0.000851644	0.003276224
ENSRNOG00000062209	-0.27190976	3.641714153	11.11105719	0.000858146	0.00329963
ENSRNOG00000024425	-0.83702281	0.117538749	11.11138113	0.000857996	0.00329963
ENSRNOG00000012552	-0.199723287	5.341736384	11.09399948	0.000866075	0.003328503
ENSRNOG00000018305	-0.366788142	5.624646354	11.09236699	0.000866838	0.003329818
ENSRNOG00000000095	-0.238573052	5.21224676	11.07744041	0.000873843	0.003354288
ENSRNOG00000018179	-0.242530592	4.655606215	11.06904783	0.000877807	0.003368687
ENSRNOG00000014917	-0.229360697	4.679349966	11.0576108	0.000883239	0.003387889
ENSRNOG00000054920	-0.329840338	2.323371872	11.05014818	0.000886801	0.003400729
ENSRNOG00000020340	-0.196729103	5.964710925	11.04771795	0.000887964	0.003404365
ENSRNOG00000034206	-0.903423517	-0.926841128	11.01898223	0.000901836	0.003452531
ENSRNOG00000015231	-0.218765393	4.447777891	11.01429717	0.000904118	0.003458131
ENSRNOG00000002612	-0.28759511	3.830121777	11.01501307	0.000903769	0.003458131
ENSRNOG00000061488	-0.91454597	-0.923837238	11.00892278	0.000906743	0.00346629
ENSRNOG00000047006	-0.520407598	5.274878683	11.00813048	0.000907131	0.003466935
ENSRNOG00000011178	-0.876866146	-0.837488162	10.99607691	0.000913049	0.00348703
ENSRNOG00000015925	-0.292620873	5.071780623	10.98854712	0.000916767	0.003497925
ENSRNOG00000024899	-1.299792171	1.028381476	10.98687518	0.000917594	0.003500164
ENSRNOG00000004512	-0.401284798	3.076307036	10.9577376	0.000932136	0.003551356
ENSRNOG00000027502	-0.432622647	2.186709679	10.94230688	0.000939931	0.003579332
ENSRNOG00000019982	-0.484351943	3.263527522	10.93119945	0.000945583	0.00359862
ENSRNOG00000026833	-0.206286329	6.965919554	10.93101357	0.000945677	0.00359862
ENSRNOG00000050996	-0.326462905	4.469503324	10.93052307	0.000945928	0.003598708
ENSRNOG00000015235	-0.390916874	4.333750605	10.92651916	0.000947974	0.003605627
ENSRNOG00000006919	-0.389286914	4.296352801	10.89214862	0.000965727	0.003667865
ENSRNOG00000061781	-0.854250343	0.20669851	10.89144031	0.000966097	0.003668388
ENSRNOG00000014882	-0.40610833	4.978019129	10.8836133	0.000970188	0.003683039
ENSRNOG00000016629	-0.2774972	5.611595037	10.88044519	0.000971849	0.003686975
ENSRNOG00000005558	-0.340910465	3.120149891	10.8784249	0.00097291	0.003689832
ENSRNOG00000017502	-0.232693282	5.330728464	10.87569532	0.000974344	0.003691992
ENSRNOG00000047922	-0.587749213	1.319700778	10.87512439	0.000974645	0.003691992
ENSRNOG00000004668	-0.518294504	1.768464019	10.87617853	0.00097409	0.003691992
ENSRNOG00000010037	-0.350315548	4.457037324	10.86678818	0.000979042	0.003707761
ENSRNOG00000004903	-0.370012717	3.123896115	10.86344255	0.000980812	0.003712689
ENSRNOG00000009576	-0.255823682	3.392942848	10.85207334	0.000986852	0.003732875
ENSRNOG00000057740	-0.356891563	2.162804726	10.84114277	0.000992694	0.003753182
ENSRNOG00000007506	-0.252308327	4.964193767	10.83978903	0.000993421	0.00375503
ENSRNOG00000019976	-0.479287944	1.304934355	10.82802382	0.000999753	0.003776261
ENSRNOG00000027161	-0.308915204	3.604093465	10.82257166	0.001002701	0.003786494
ENSRNOG00000006261	-0.241680635	4.481901924	10.81144624	0.001008745	0.0038075
ENSRNOG00000020333	-0.292424992	4.314715652	10.78925481	0.00102091	0.003851581
ENSRNOG00000034161	-0.311517359	4.528947854	10.77269083	0.001030086	0.003882041
ENSRNOG00000003833	-0.53867038	4.885096907	10.76696041	0.001033281	0.003891761
ENSRNOG00000020922	-0.458110797	4.336516821	10.75945249	0.001037481	0.003903866
ENSRNOG00000011480	-0.216762174	5.414851672	10.75876602	0.001037866	0.003904387
ENSRNOG00000011962	-0.25807311	3.488575566	10.7372043	0.001050028	0.003948267
ENSRNOG00000006949	-0.219927807	5.666219304	10.72570395	0.001056575	0.003970995
ENSRNOG00000026162	-0.502950443	1.593526824	10.72382611	0.001057647	0.003974084
ENSRNOG00000011498	-0.275850542	7.87845971	10.72149345	0.001058982	0.003978153
ENSRNOG00000001971	-0.258335679	3.925004202	10.71473729	0.001062855	0.003991758
ENSRNOG00000051701	-0.387016621	1.915639067	10.7140953	0.001063224	0.003992197
ENSRNOG00000061205	-0.736595672	-0.144037694	10.71174497	0.001064576	0.003996324
ENSRNOG00000009278	-0.263939333	4.734559735	10.70808862	0.001066682	0.00400328
ENSRNOG00000060634	-0.649786625	0.668784466	10.70716031	0.001067217	0.00400434
ENSRNOG00000059245	-0.853674055	-0.825490172	10.70217553	0.001070096	0.004013241
ENSRNOG00000015597	-0.233812397	4.489945962	10.6891398	0.001077663	0.004038749
ENSRNOG00000056950	-0.666722656	1.827855867	10.68754912	0.00107859	0.004041033
ENSRNOG00000031665	-0.3845586	2.057445989	10.68721852	0.001078783	0.004041033
ENSRNOG00000040024	-0.481598216	1.141616466	10.68675413	0.001079054	0.004041092
ENSRNOG00000056249	-0.767914648	-1.068255283	10.68031566	0.001082816	0.004051348
ENSRNOG00000061129	-0.229767709	6.067487779	10.6750586	0.001085897	0.0040599
ENSRNOG00000030629	-0.242621935	3.432898233	10.67423072	0.001086384	0.0040599
ENSRNOG00000020059	-0.36508625	2.900816668	10.66134156	0.00109398	0.004085397
ENSRNOG00000024335	-0.241133479	4.251927588	10.64116379	0.001105981	0.004125818
ENSRNOG00000047506	-0.334583003	4.180033324	10.6409526	0.001106107	0.004125818
ENSRNOG00000029416	-0.317098943	2.976983926	10.63043007	0.001112418	0.00414643
ENSRNOG00000000662	-0.329417464	3.219533272	10.62286602	0.001116978	0.004161466
ENSRNOG00000010587	-0.281995959	3.955837508	10.61475542	0.001121888	0.00417681
ENSRNOG00000003853	-0.286363908	7.956373732	10.61494266	0.001121774	0.00417681
ENSRNOG00000019986	-0.289739745	4.290747957	10.60545038	0.001127548	0.004195909
ENSRNOG00000053687	-0.489509077	1.792276403	10.60479377	0.001127948	0.004196413
ENSRNOG00000008610	-0.320941628	3.319682564	10.59162291	0.001136012	0.004223437
ENSRNOG00000022701	-0.463315335	0.87024428	10.57824838	0.00114426	0.004251107
ENSRNOG00000002478	-0.216534091	4.503426377	10.57714293	0.001144945	0.00425184
ENSRNOG00000025892	-0.220242069	6.792894042	10.57374995	0.001147048	0.004258071
ENSRNOG00000020004	-0.231435849	5.113731164	10.56662125	0.00115148	0.004271915
ENSRNOG00000025619	-0.387227435	4.623142577	10.55594661	0.001158149	0.004293638
ENSRNOG00000057057	-0.98049578	-0.955787283	10.55390234	0.00115943	0.004297383
ENSRNOG00000007515	-0.248778301	7.120896839	10.5482929	0.001162954	0.00430742
ENSRNOG00000017904	-0.300854349	5.271592903	10.5364637	0.001170422	0.004332037
ENSRNOG00000003205	-0.412419045	5.535483406	10.53376746	0.00117213	0.004337348
ENSRNOG00000020700	-0.504521742	2.319316783	10.5196667	0.001181108	0.004369547
ENSRNOG00000011040	-0.581940358	5.701484404	10.51494098	0.001184132	0.004379712
ENSRNOG00000039819	-0.232511599	4.898773911	10.51061707	0.001186906	0.004388947
ENSRNOG00000006655	-0.233464731	5.360050443	10.49346463	0.001197975	0.004428844
ENSRNOG00000001808	-0.2390562	4.712529986	10.49168449	0.00119913	0.004432078
ENSRNOG00000010105	-0.815781128	1.625953774	10.48977072	0.001200373	0.004435636
ENSRNOG00000012966	-0.282661502	5.084190209	10.48512482	0.001203395	0.004445766
ENSRNOG00000042347	-0.365654518	2.29006061	10.47429865	0.001210467	0.004470851
ENSRNOG00000007351	-0.408122577	4.791831866	10.46406812	0.001217189	0.004491488
ENSRNOG00000051298	-0.609197786	0.468977898	10.4534612	0.001224199	0.004511045
ENSRNOG00000019342	-0.334228632	4.290745801	10.4504688	0.001226183	0.004516256
ENSRNOG00000020336	-0.228526718	6.391520474	10.44449197	0.001230157	0.004528787
ENSRNOG00000011910	-0.282569546	5.427163659	10.43821745	0.001234343	0.004542598
ENSRNOG00000010784	-0.349721726	7.801155502	10.43798081	0.001234502	0.004542598

ENSRNOG00000051406	-0.337087383	3.240929157	10.43716036	0.00123505	0.004542598
ENSRNOG00000052122	-0.588885999	1.627612975	10.43380374	0.001237297	0.00454978
ENSRNOG00000007066	-0.282989403	3.382861668	10.42134392	0.001245672	0.00457739
ENSRNOG00000005931	-0.383863597	4.693097905	10.40972414	0.001253535	0.004599877
ENSRNOG00000059492	-0.305167564	4.532765833	10.40426447	0.001257246	0.004610291
ENSRNOG00000007924	-0.325307064	4.384687498	10.39767875	0.001261738	0.004624621
ENSRNOG00000018829	-0.398450008	6.994538125	10.39432099	0.001264035	0.004631966
ENSRNOG00000024245	-0.287385927	3.856843663	10.37165694	0.001279646	0.004684838
ENSRNOG00000012574	-0.266568143	4.668223497	10.36795812	0.001282213	0.004692716
ENSRNOG00000018475	-0.274943148	5.031258986	10.36770128	0.001282391	0.004692716
ENSRNOG00000015161	-0.498034572	2.075391462	10.36415368	0.001284858	0.004700656
ENSRNOG00000027730	-0.505575252	2.160745073	10.35714117	0.001289748	0.004717456
ENSRNOG00000018405	-0.949734525	-0.515170433	10.35610431	0.001290472	0.004719016
ENSRNOG00000056849	-0.59260038	2.768643021	10.35228864	0.001293143	0.004726597
ENSRNOG00000018381	-0.336460062	3.056844291	10.34754242	0.001296472	0.004736579
ENSRNOG00000027447	-0.280514116	4.197902012	10.34513148	0.001298166	0.004741676
ENSRNOG00000015585	-0.365410647	3.248962339	10.34371278	0.001299164	0.004744227
ENSRNOG00000007829	-0.247535318	4.516402756	10.32944262	0.001309247	0.004775541
ENSRNOG00000025496	-0.231259455	7.286441576	10.32055638	0.001315566	0.004796379
ENSRNOG00000029799	-0.248322626	5.897108615	10.3176618	0.001317631	0.004802802
ENSRNOG00000021900	-0.379360872	2.158297089	10.31178948	0.00132183	0.004815891
ENSRNOG00000020452	-0.246485907	5.194585142	10.30946778	0.001323494	0.004819736
ENSRNOG00000021199	-0.594438981	1.744444225	10.30957802	0.001323415	0.004819736
ENSRNOG00000016810	-0.395387685	8.504442127	10.2940075	0.001334629	0.004854702
ENSRNOG00000046969	-0.327230262	3.167083279	10.29406457	0.001334588	0.004854702
ENSRNOG00000045649	-0.333194212	4.830706272	10.29289945	0.001335431	0.004856503
ENSRNOG00000012447	-0.307669989	2.880882326	10.28277756	0.001342777	0.004879854
ENSRNOG00000042727	-0.603410144	-0.028256573	10.28013777	0.001344699	0.004884599
ENSRNOG00000017272	-0.209776653	4.884524423	10.27727829	0.001346785	0.004891052
ENSRNOG00000053054	-0.571777031	1.583099127	10.27129073	0.001351162	0.004903794
ENSRNOG00000034093	-0.697097684	0.090594669	10.2574741	0.001361319	0.004937043
ENSRNOG00000014481	-0.560260546	1.378958641	10.25649932	0.001362039	0.004938522
ENSRNOG00000038319	-0.694174977	2.137929635	10.24369657	0.001371525	0.004966341
ENSRNOG00000021143	-0.233418295	5.795671574	10.2423749	0.001372508	0.00496738
ENSRNOG00000020250	-0.302047965	3.585476329	10.23790525	0.001375837	0.004977156
ENSRNOG00000027295	-0.381036029	2.779275464	10.23645943	0.001376916	0.004979921
ENSRNOG00000020485	-0.46108176	4.457843426	10.23583443	0.001377383	0.004980471
ENSRNOG00000000312	-0.199367431	5.710146154	10.20697389	0.001399106	0.005054401
ENSRNOG00000018322	-0.214731308	7.743584345	10.19255259	0.00141009	0.005090598
ENSRNOG00000018082	-0.400273446	2.309731356	10.19161758	0.001410805	0.005092019
ENSRNOG00000018674	-0.247853071	5.735778854	10.18170901	0.001418407	0.005118288
ENSRNOG00000059705	-0.290520206	6.603301693	10.15702345	0.001437525	0.005182554
ENSRNOG00000020467	-0.442948022	4.208038587	10.15313051	0.001440564	0.005192327
ENSRNOG00000043341	-0.271387913	2.939828211	10.14342406	0.001448169	0.00521628
ENSRNOG00000007665	-0.176351342	6.104313707	10.14332311	0.001448248	0.00521628
ENSRNOG00000007875	-0.28758721	4.819280335	10.14233399	0.001449026	0.00521628
ENSRNOG00000023381	-0.282472577	3.340743185	10.14212984	0.001449186	0.00521628
ENSRNOG00000025484	-0.303870539	3.099214847	10.14265235	0.001448776	0.00521628
ENSRNOG00000018650	-0.233332787	5.855305159	10.13282195	0.001456522	0.005240304
ENSRNOG00000055641	-0.262797313	3.127415758	10.1251352	0.001462609	0.005259813
ENSRNOG00000003890	-0.345049948	7.818151923	10.1228783	0.001464401	0.005265061
ENSRNOG00000011078	-0.723765675	-0.279263554	10.1202304	0.001466507	0.005271434
ENSRNOG00000053928	-0.301327554	2.82639107	10.10447513	0.001479097	0.005311868
ENSRNOG00000005042	-0.292486625	3.071562964	10.10006238	0.001482643	0.005323395
ENSRNOG00000043037	-0.32179985	3.853102025	10.08722209	0.00149301	0.005352122
ENSRNOG00000042467	-0.188977229	5.554148403	10.08737413	0.001492887	0.005352122
ENSRNOG00000046599	-0.640722668	2.164387998	10.08772508	0.001492603	0.005352122
ENSRNOG00000052419	-0.902240697	-1.232089491	10.08220461	0.001497081	0.005363073
ENSRNOG00000014434	-0.265300627	4.913352882	10.08102257	0.001498042	0.0053653
ENSRNOG00000030930	-0.879755726	-1.033985049	10.06535073	0.001510838	0.005402577
ENSRNOG00000012748	-0.318093794	3.430527124	10.06035285	0.001514942	0.005413163
ENSRNOG00000017491	-0.339321818	3.764808697	10.06041999	0.001514887	0.005413163
ENSRNOG00000003003	-0.229762939	4.627810169	10.06008053	0.001515166	0.005413163
ENSRNOG00000055024	-0.873110781	-0.874077504	10.05472146	0.00151958	0.00542526
ENSRNOG00000052389	-0.78623748	-0.98511954	10.05214668	0.001521705	0.005431623
ENSRNOG00000049137	-0.222588328	4.446948643	10.04707206	0.001525903	0.005445378
ENSRNOG00000047884	-0.480720532	2.434850093	10.03820909	0.001533262	0.005469176
ENSRNOG00000001815	-0.228628764	9.405179441	10.02965076	0.001540402	0.005493408
ENSRNOG00000021839	-0.373598815	4.455506223	10.02154356	0.001547198	0.005512674
ENSRNOG00000027955	-0.228063897	3.890979157	10.01088868	0.001556174	0.005539672
ENSRNOG00000014532	-1.03421116	-1.101926902	10.00911813	0.001557671	0.005543754
ENSRNOG00000058609	-0.410292787	3.394787156	10.00458616	0.001561509	0.005556164
ENSRNOG00000045668	-0.27391131	3.663644933	10.00036234	0.001565094	0.005567671
ENSRNOG00000006619	-0.278212147	4.938833598	9.998899295	0.001566338	0.005570844
ENSRNOG00000004287	-0.263736671	4.01216692	9.994129161	0.001570401	0.005581532
ENSRNOG00000052418	-0.2070528	5.180919479	9.992229842	0.001572021	0.00558377
ENSRNOG00000058291	-0.773274598	-0.935354162	9.98577312	0.001577543	0.005601888
ENSRNOG00000018567	-0.204515462	6.926552062	9.977718308	0.001584459	0.005621404
ENSRNOG00000019211	-0.355715679	3.023579808	9.974303768	0.0015874	0.005630576
ENSRNOG00000027360	-0.203290554	6.099421233	9.973457789	0.00158813	0.005631902
ENSRNOG00000017213	-0.211764549	6.442688475	9.972468353	0.001588983	0.005633668
ENSRNOG00000022245	-0.227663699	6.183733891	9.967832946	0.001592989	0.005645341
ENSRNOG00000001341	-0.344196388	2.506662564	9.951491065	0.001607192	0.005693126
ENSRNOG00000031138	-0.652115437	2.820330133	9.949506417	0.001608925	0.005696718
ENSRNOG00000050437	-0.244079505	6.618394623	9.946601664	0.001611466	0.005704438
ENSRNOG00000052826	-0.784295224	-0.390231692	9.936800749	0.001620069	0.005733609
ENSRNOG00000019174	-0.440348543	3.564410729	9.930169416	0.001625916	0.0057513
ENSRNOG00000021395	-0.521129602	1.307400956	9.921058526	0.001633984	0.005776401
ENSRNOG00000017077	-0.359789604	4.070217492	9.919428294	0.001635432	0.005780229
ENSRNOG00000000158	-0.267979979	5.145848385	9.915932645	0.001638541	0.005789925
ENSRNOG00000019456	-0.198559486	6.245564072	9.915441194	0.001638978	0.005790179
ENSRNOG00000051179	-0.242425869	5.49998912	9.879955218	0.00167089	0.005892399
ENSRNOG00000006978	-0.307951511	3.345097895	9.871180574	0.001678877	0.005916614
ENSRNOG00000055795	-0.605149743	3.352103	9.866600523	0.001683062	0.005930041
ENSRNOG00000021776	-0.239929365	4.596176169	9.841459842	0.00170622	0.006003984
ENSRNOG00000027690	-0.251161318	6.009252263	9.833366657	0.001713744	0.006027415
ENSRNOG00000056624	-0.680018507	-0.149661758	9.827640923	0.001719087	0.006044864
ENSRNOG00000023079	-0.287063468	4.7822094	9.822186536	0.001724192	0.006060125
ENSRNOG00000006828	-0.554535263	3.339789835	9.795464734	0.001749428	0.006142005
ENSRNOG00000011992	-0.570098385	1.635582443	9.78242164	0.001761881	0.006181614

ENSRNOG00000030131	-0.285776148	3.253039639	9.774836975	0.001769164	0.006203043
ENSRNOG00000007733	-0.385270508	8.866422	9.771751996	0.001772135	0.006212085
ENSRNOG00000053060	-0.215036685	6.318426848	9.770069357	0.001773758	0.006216396
ENSRNOG00000011653	-0.223562923	7.379960362	9.762731232	0.001780851	0.006239877
ENSRNOG00000050701	-0.559483616	2.678968457	9.762199476	0.001781367	0.006240301
ENSRNOG00000004534	-0.403633019	2.307230172	9.754619585	0.001788727	0.006264697
ENSRNOG00000059866	-0.739950591	2.664396346	9.748138193	0.001795044	0.006285434
ENSRNOG00000017493	-0.263829086	5.594099275	9.746131082	0.001797005	0.006289518
ENSRNOG00000003215	-0.293194696	3.38407526	9.73847346	0.001804507	0.006312983
ENSRNOG00000006302	-0.234188085	6.134762342	9.729833965	0.001813009	0.006341325
ENSRNOG00000046741	-0.190544705	6.321594068	9.72824106	0.001814581	0.006345422
ENSRNOG00000062220	-0.543252415	0.845942099	9.722854923	0.001819906	0.006361234
ENSRNOG00000003875	-0.263422621	6.020829673	9.662744432	0.001880425	0.006556848
ENSRNOG00000031483	-0.995239476	-0.98424714	9.655147213	0.001888218	0.006582571
ENSRNOG00000003831	-0.34898303	2.186488408	9.654375909	0.001889011	0.006583886
ENSRNOG00000033824	-0.221270065	5.417841679	9.651691933	0.001891773	0.006592062
ENSRNOG00000042602	-0.498964941	2.846873278	9.64627911	0.001897355	0.00661006
ENSRNOG00000007253	-0.24669553	5.008766175	9.630334047	0.001913898	0.006663294
ENSRNOG00000023579	-0.255006224	5.443118833	9.620742542	0.001923919	0.006696711
ENSRNOG00000014665	-0.171202615	5.713518126	9.600977628	0.001944738	0.006758776
ENSRNOG00000056036	-0.372049208	2.495935587	9.599297048	0.001946519	0.00676348
ENSRNOG00000013529	-0.224854787	4.954996741	9.588881547	0.001957592	0.006799512
ENSRNOG00000006810	-0.201344267	5.624670881	9.588735244	0.001957748	0.006799512
ENSRNOG00000010098	-0.406305041	2.005348874	9.583480893	0.001963358	0.006814514
ENSRNOG00000032668	-0.290701947	2.678490097	9.570725798	0.001977046	0.006860519
ENSRNOG00000019776	-0.214189392	5.748949362	9.568203481	0.001979764	0.006868446
ENSRNOG00000014125	-0.700821022	1.496871574	9.566734147	0.001981349	0.00687244
ENSRNOG00000013251	-0.242160909	4.458334815	9.562385648	0.001986048	0.00688723
ENSRNOG00000015733	-0.379534615	7.168028578	9.558725567	0.001990012	0.006899464
ENSRNOG00000029678	-0.377088214	2.057257023	9.552785203	0.001996463	0.006912746
ENSRNOG00000019150	-0.201782588	4.799705978	9.552857919	0.001996383	0.006912746
ENSRNOG00000009696	-0.220154887	4.919930173	9.547421174	0.002002305	0.006929946
ENSRNOG00000000145	-0.257446897	4.684630039	9.531379547	0.002019882	0.006983148
ENSRNOG00000014483	-0.219972636	4.953315926	9.52924847	0.002022229	0.006989735
ENSRNOG00000014739	-0.45819094	2.703667282	9.527535996	0.002024117	0.006993207
ENSRNOG00000002154	-0.563274703	1.379316352	9.514441519	0.002038611	0.007041748
ENSRNOG00000006231	-0.233784588	6.17628781	9.503952885	0.002050297	0.007075939
ENSRNOG00000012880	-0.197149036	5.301454859	9.490876802	0.002064961	0.007123441
ENSRNOG00000055687	-0.329228604	2.444384532	9.487230543	0.002069069	0.007134552
ENSRNOG00000018520	-0.416108537	2.208952025	9.487218125	0.002069083	0.007134552
ENSRNOG00000000520	-0.249087555	7.343587177	9.481768465	0.002075238	0.007152661
ENSRNOG00000029466	-0.290854984	3.116846498	9.480177598	0.002077039	0.007157308
ENSRNOG00000007587	-0.246642615	4.645113311	9.476741796	0.002080932	0.007167605
ENSRNOG00000015630	-0.179937786	7.637909825	9.47357637	0.002084526	0.007178422
ENSRNOG00000015523	-0.356297774	4.817342398	9.467471758	0.002091474	0.007200784
ENSRNOG00000007732	-0.256765683	4.58222708	9.454077133	0.002106803	0.007251981
ENSRNOG00000008892	-0.268088273	3.735111739	9.452811905	0.002108256	0.007255408
ENSRNOG00000014436	-0.185451847	7.029376935	9.452355677	0.002108781	0.007255636
ENSRNOG00000011678	-0.225572198	5.331250199	9.444019584	0.002118387	0.007285522
ENSRNOG00000038176	-0.236776052	4.925094782	9.442932204	0.002119643	0.007288259
ENSRNOG00000007398	-0.295502081	2.615911687	9.44057302	0.002122372	0.007296056
ENSRNOG00000057042	-0.184311113	4.675749679	9.434395851	0.002129532	0.007315907
ENSRNOG00000062253	-1.008729724	-1.098550928	9.425428971	0.00213997	0.007348577
ENSRNOG00000017253	-0.311888504	4.766328109	9.423513238	0.002142207	0.007354663
ENSRNOG00000033792	-0.445925935	2.234582642	9.407376366	0.002161142	0.007406824
ENSRNOG00000007504	-0.212267391	6.260038473	9.402733288	0.002166622	0.007423998
ENSRNOG00000039500	-0.786246139	-1.040355015	9.402199736	0.002167253	0.007424552
ENSRNOG00000021911	-0.233034182	4.226168563	9.380644384	0.002192885	0.007505866
ENSRNOG00000056372	-0.745484047	-0.708886976	9.377231744	0.002196971	0.007514978
ENSRNOG00000056907	-0.237179978	6.479288819	9.374218527	0.002200585	0.007525716
ENSRNOG00000028856	-0.440027802	5.101658173	9.370653849	0.002204869	0.007532853
ENSRNOG00000010919	-0.426419051	2.84036008	9.366573452	0.002209782	0.007545761
ENSRNOG00000010369	-0.217969615	4.973308658	9.348418587	0.00223178	0.007611027
ENSRNOG00000001851	-0.29353933	4.963369316	9.345240443	0.002235653	0.007619314
ENSRNOG00000013916	-0.561285881	7.715023484	9.345566614	0.002235255	0.007619314
ENSRNOG00000042478	-0.277276095	5.619491262	9.317207926	0.002270117	0.007723469
ENSRNOG00000007316	-0.197562622	7.194946249	9.314293245	0.002273731	0.007734103
ENSRNOG00000023492	-0.409724056	3.184402882	9.309882802	0.002279211	0.007751078
ENSRNOG00000006368	-0.350768651	6.752211769	9.307889477	0.002281692	0.00775785
ENSRNOG00000014710	-0.718706429	-0.3409411	9.306093444	0.00228393	0.007763791
ENSRNOG00000002091	-0.194782299	4.560358119	9.300391571	0.002291049	0.007786321
ENSRNOG00000004971	-0.23707965	3.975527302	9.296555573	0.002295851	0.007799592
ENSRNOG00000056377	-0.785317332	-1.010425557	9.294270291	0.002298717	0.007807354
ENSRNOG00000015271	-0.294358101	2.438366787	9.291019346	0.0023028	0.007817867
ENSRNOG00000016991	-0.210889413	7.793171079	9.290043326	0.002304027	0.00781871
ENSRNOG00000013877	-0.353828224	2.105618027	9.284726061	0.002310725	0.007836371
ENSRNOG00000009771	-0.231085319	5.463353888	9.277432158	0.002319944	0.007865953
ENSRNOG00000012266	-0.214020747	5.874907778	9.27615665	0.00232156	0.007869747
ENSRNOG00000004863	-0.56129283	5.662314432	9.271913524	0.002326945	0.007886311
ENSRNOG00000051344	-0.252719478	6.648563327	9.268801537	0.002330902	0.007898031
ENSRNOG00000034198	-0.227329728	6.422604922	9.267825501	0.002332144	0.00790055
ENSRNOG00000053621	-0.753643573	-0.310894364	9.258951333	0.002343472	0.007933832
ENSRNOG00000025071	-0.213252942	5.596375685	9.258167444	0.002344475	0.007935532
ENSRNOG00000019618	-0.171828716	5.794148605	9.256666314	0.002346397	0.007938801
ENSRNOG00000011785	-0.193869291	6.009905678	9.251200144	0.002353411	0.007960674
ENSRNOG00000003971	-0.178666349	5.79953863	9.247780643	0.00235781	0.007968744
ENSRNOG00000049075	-0.272545977	5.859861371	9.246858586	0.002358997	0.007969355
ENSRNOG00000002580	-0.222949537	4.511609415	9.242151551	0.002365069	0.007988162
ENSRNOG00000023065	-0.227149983	5.429212871	9.238674975	0.002369564	0.008001636
ENSRNOG00000047250	-0.301377243	5.821865998	9.234967526	0.002374366	0.008014435
ENSRNOG00000011421	-0.215905017	7.402542538	9.228289099	0.002383042	0.008038579
ENSRNOG00000011997	-0.251391424	3.815864219	9.19446001	0.002427487	0.008179789
ENSRNOG00000011987	-0.271252386	4.189630544	9.18724147	0.002437079	0.008210364
ENSRNOG00000042427	-0.214771033	4.812015355	9.18003914	0.002446688	0.008239229
ENSRNOG00000015902	-0.812896208	1.175080838	9.173326157	0.002455679	0.008264903
ENSRNOG00000023162	-0.543202229	4.188578558	9.166042086	0.002465472	0.008291001
ENSRNOG00000029956	-0.289097499	3.820180223	9.163512391	0.002468882	0.008299848
ENSRNOG00000032803	-1.115908487	0.496750398	9.150440379	0.002486582	0.008357576
ENSRNOG00000028254	-0.231182783	3.81586109	9.14467597	0.002494428	0.008380389
ENSRNOG00000009313	-0.185368097	7.745248277	9.140527932	0.00250009	0.008397628

ENSRNOG00000047647	-0.762754705	-0.572066929	9.139886086	0.002500967	0.008398793
ENSRNOG00000006591	-0.519183904	6.542934848	9.138229343	0.002503232	0.008404619
ENSRNOG00000019145	-0.210536355	6.70012129	9.129605464	0.00251506	0.008437172
ENSRNOG00000007177	-0.269626417	4.931127782	9.129882178	0.002514679	0.008437172
ENSRNOG00000018350	-0.290290989	5.477641782	9.126738314	0.002519004	0.008445038
ENSRNOG00000038449	-0.246038334	3.795158592	9.104754331	0.00254946	0.008539907
ENSRNOG00000007947	-0.242061748	4.349731359	9.102314588	0.002552862	0.008549497
ENSRNOG00000048765	-0.685188518	-0.925627396	9.09836585	0.00255838	0.008565384
ENSRNOG00000033176	-0.249217995	5.308733883	9.098145444	0.002558688	0.008565384
ENSRNOG00000021157	-0.25569664	7.520434697	9.09627887	0.002561301	0.008572317
ENSRNOG00000050600	-0.36245836	2.479843176	9.093292371	0.002565486	0.008584512
ENSRNOG00000004398	-0.985540602	-0.969624194	9.071316306	0.002596501	0.008680954
ENSRNOG00000002292	-0.219948406	6.708089311	9.066036429	0.002604009	0.008702944
ENSRNOG00000005606	-0.262153404	5.650278285	9.043144706	0.002636817	0.008803391
ENSRNOG00000014029	-0.250679173	7.611744779	9.041464346	0.002639242	0.008806876
ENSRNOG00000058694	-0.222392542	3.840677104	9.025372472	0.002662577	0.008875766
ENSRNOG00000006604	-0.416833178	8.921422291	9.025344307	0.002662618	0.008875766
ENSRNOG00000017035	-0.178900811	5.763960306	9.025529609	0.002662348	0.008875766
ENSRNOG00000032885	-0.722624363	-0.494721201	9.022360293	0.002666968	0.008882787
ENSRNOG00000042504	-0.426078264	2.338214178	9.016652037	0.00267531	0.008906824
ENSRNOG00000006644	-0.261840809	4.527784853	9.008369726	0.00268746	0.008941636
ENSRNOG00000050510	-0.221107622	5.120835147	9.00380957	0.002694174	0.008962091
ENSRNOG00000002980	-0.18517356	6.273742119	9.001672781	0.002697326	0.008970691
ENSRNOG00000017258	-0.233168734	3.75843655	8.998097119	0.002702609	0.008986372
ENSRNOG00000006576	-0.200762621	5.26809613	8.997344522	0.002703722	0.008988186
ENSRNOG00000060356	-0.512006563	2.207446626	8.996025605	0.002705674	0.008992788
ENSRNOG00000049661	-0.382663647	1.919260706	8.988528927	0.002716796	0.009024072
ENSRNOG00000060626	-0.297525304	3.769327482	8.980827808	0.00272827	0.009058383
ENSRNOG00000031731	-0.198652998	5.462916586	8.979398271	0.002730405	0.009063572
ENSRNOG00000024990	-0.663430835	-0.675773627	8.976361882	0.002734947	0.009076744
ENSRNOG00000014275	-0.364023633	3.875519929	8.969102992	0.002745834	0.009107149
ENSRNOG00000042340	-0.281373613	3.961527529	8.9679598	0.002747552	0.009110941
ENSRNOG00000062221	-0.400815103	1.080675022	8.966340652	0.002749988	0.00911711
ENSRNOG00000051450	-0.468500259	2.939710578	8.96315737	0.002754784	0.009129186
ENSRNOG00000033583	-0.365239766	1.89371308	8.963429502	0.002754374	0.009129186
ENSRNOG00000052341	-1.023067617	-1.178896599	8.960253657	0.002759166	0.009141383
ENSRNOG00000009196	-0.25129355	5.214929146	8.95360782	0.002769221	0.009171272
ENSRNOG00000031346	-0.257327338	4.928722145	8.950332196	0.002774191	0.00918389
ENSRNOG00000026204	-0.336839034	4.034178761	8.942697879	0.00278581	0.009216571
ENSRNOG00000011348	-0.251925306	5.894586855	8.934510249	0.002798325	0.009256043
ENSRNOG00000008011	-0.543026727	1.846485999	8.931763225	0.002802537	0.009268038
ENSRNOG00000042094	-0.593918701	4.432538826	8.929403664	0.00280616	0.009278081
ENSRNOG00000051563	-0.336674289	3.109243034	8.916514476	0.002826034	0.009337941
ENSRNOG00000021100	-0.365854864	2.284441578	8.910839582	0.002834829	0.00936505
ENSRNOG00000048659	-0.820802691	-1.051562697	8.910310756	0.00283565	0.009365808
ENSRNOG00000010352	-0.266125006	4.443813481	8.90926259	0.002837278	0.009369231
ENSRNOG00000013673	-0.296988132	3.750136306	8.897878321	0.002855022	0.009416042
ENSRNOG00000011199	-0.327169421	6.929922914	8.892706746	0.00286312	0.009440783
ENSRNOG00000001049	-0.22283259	9.067119016	8.891583767	0.002864881	0.009442658
ENSRNOG00000018773	-0.240461297	3.335861888	8.879855954	0.002883343	0.009497577
ENSRNOG00000018657	-0.256999546	5.268747142	8.871210778	0.002897029	0.009540674
ENSRNOG00000004076	-0.28937954	3.40503536	8.864373645	0.0029079	0.009574483
ENSRNOG00000056575	-0.851975174	-0.213635834	8.846486814	0.002936536	0.009660733
ENSRNOG00000052124	-0.844772798	-1.186673925	8.839020457	0.002948574	0.00969832
ENSRNOG00000002810	-0.378889107	2.495281224	8.834482573	0.002955914	0.009718428
ENSRNOG00000000277	-0.33049133	2.864419909	8.834790527	0.002955416	0.009718428
ENSRNOG00000003400	-0.402828888	1.340122806	8.819755923	0.002979865	0.009791074
ENSRNOG00000057097	-0.369665738	2.788106249	8.803999083	0.00300571	0.009871896
ENSRNOG00000012609	-1.077613697	-0.668510271	8.802161265	0.003008739	0.009879796
ENSRNOG00000005804	-0.168559477	5.768587022	8.799241936	0.003013557	0.009893566
ENSRNOG00000029859	-0.635647056	0.03611911	8.796921595	0.003017393	0.009904103
ENSRNOG00000014835	-0.420502658	0.55330988	8.793314223	0.003023365	0.00992165
ENSRNOG00000002610	-0.267566659	6.220716137	8.78989622	0.003029035	0.009938197
ENSRNOG00000058784	-0.360839759	4.098727298	8.787009129	0.003033832	0.009951875
ENSRNOG00000011492	-0.336196986	2.990074368	8.782714775	0.003040983	0.0099712
ENSRNOG00000004437	-0.377315571	4.082892181	8.781991176	0.003042189	0.00997309
ENSRNOG00000046621	-0.37728801	2.274629079	8.78046651	0.003044733	0.009979364
ENSRNOG00000012138	-0.191528337	4.913679891	8.778291742	0.003048365	0.009987134
ENSRNOG00000021155	-0.593308541	2.387751506	8.75816425	0.003082191	0.010079599
ENSRNOG00000024450	-0.239369712	3.196609706	8.757712798	0.003082954	0.010079599
ENSRNOG00000052003	-0.468984798	1.012798685	8.751591235	0.00309332	0.010109315
ENSRNOG00000012810	-0.265984075	3.441631063	8.745906374	0.003102978	0.010138786
ENSRNOG00000012255	-0.20199122	6.898247824	8.742766255	0.003108326	0.010154165
ENSRNOG00000003803	-0.261950893	6.566912696	8.741871842	0.003109851	0.010154957
ENSRNOG00000018962	-0.422873344	1.234748553	8.739066541	0.003114639	0.010168494
ENSRNOG00000007472	-0.210396807	7.08239105	8.73819754	0.003116124	0.010171244
ENSRNOG00000003187	-0.249750073	4.541610443	8.728604037	0.003132562	0.010214373
ENSRNOG00000010150	-0.219989303	6.49885539	8.727349677	0.003134718	0.010217194
ENSRNOG00000003171	-0.742112775	1.879118396	8.725499759	0.0031379	0.010225461
ENSRNOG00000020054	-0.358030365	2.258566047	8.724594599	0.003139459	0.010228434
ENSRNOG00000008390	-0.252427602	5.017850308	8.721902355	0.003144098	0.010239335
ENSRNOG00000033719	-0.288879951	2.475518394	8.700906031	0.003180519	0.010347307
ENSRNOG00000058446	-0.468517637	1.679313019	8.699706094	0.003182614	0.010351993
ENSRNOG00000010815	-0.194959442	4.80454064	8.691069886	0.003197729	0.010399022
ENSRNOG00000018996	-0.280532707	3.058513271	8.677083682	0.003222362	0.010474827
ENSRNOG00000012362	-0.519053948	5.753346368	8.674543286	0.003226857	0.010487286
ENSRNOG00000037310	-0.750711161	1.234694287	8.671271484	0.003232656	0.010503975
ENSRNOG00000001050	-0.170488109	5.956485664	8.669772287	0.003235316	0.010507332
ENSRNOG00000039571	-0.661437726	-0.480692643	8.670308876	0.003234364	0.010507332
ENSRNOG00000027923	-0.215825568	4.179791783	8.662823153	0.003247677	0.01054413
ENSRNOG00000022971	-0.270584844	3.123007421	8.659481123	0.003253639	0.010561321
ENSRNOG00000006995	-0.267980996	4.75378471	8.640164538	0.003288317	0.010667326
ENSRNOG00000003714	-0.269715707	5.253875374	8.628121631	0.003310127	0.010731481
ENSRNOG00000005228	-0.203018251	6.013819414	8.622034589	0.003321207	0.010765197
ENSRNOG00000045920	-0.407814302	4.390315189	8.620463577	0.003324072	0.01077228
ENSRNOG00000003198	-0.232694708	7.711950113	8.616449383	0.003331406	0.010791629
ENSRNOG00000005564	-0.243034905	4.816062755	8.61197713	0.003339596	0.010811523
ENSRNOG00000003472	-0.315211707	3.901768465	8.6099558	0.003343304	0.010819104
ENSRNOG00000042777	-0.272685514	3.130561912	8.597726885	0.003365829	0.010883098
ENSRNOG00000042668	-0.367244368	2.516011583	8.597117604	0.003366955	0.010884516

ENSRNOG00000008980	-0.263082277	3.230344164	8.588044389	0.003383772	0.010929956
ENSRNOG00000043358	-0.930210157	-1.063066374	8.588332965	0.003383236	0.010929956
ENSRNOG00000017723	-0.239277665	5.642243707	8.586577312	0.003386499	0.010936535
ENSRNOG00000012004	-0.190027862	5.456874897	8.584931948	0.003389561	0.010944189
ENSRNOG00000011588	-0.227418522	5.689267043	8.579575863	0.003399545	0.010971953
ENSRNOG00000017787	-0.242986681	3.884598576	8.572778634	0.00341226	0.011006524
ENSRNOG00000012947	-0.195948765	5.633457232	8.566619989	0.003423822	0.011039054
ENSRNOG00000034052	-0.941909094	-0.965678982	8.56601838	0.003424953	0.011040455
ENSRNOG00000055853	-0.251653061	3.589384032	8.56047679	0.003435394	0.011070142
ENSRNOG00000057623	-0.175478842	6.532058227	8.556974049	0.003442011	0.011088666
ENSRNOG00000051623	-0.447614804	4.098045061	8.530387003	0.003492654	0.011238099
ENSRNOG00000056571	-0.515702811	1.00550916	8.51569772	0.003520959	0.011315378
ENSRNOG00000042152	-0.502881471	1.283001213	8.49524631	0.003560756	0.01143399
ENSRNOG00000059589	-0.218790314	5.00926261	8.491754649	0.003567596	0.011453632
ENSRNOG00000022202	-0.216581146	5.874042837	8.490804558	0.003569459	0.011454969
ENSRNOG00000003329	-0.312120222	4.385592042	8.482576121	0.00358564	0.011502233
ENSRNOG00000043419	-0.339508898	3.340187205	8.478919473	0.003592854	0.011520708
ENSRNOG00000009751	-0.305148814	1.852760419	8.476099824	0.003598428	0.011536243
ENSRNOG00000028185	-0.284365145	5.250652466	8.475701929	0.003599215	0.01153643
ENSRNOG00000018946	-0.201643319	5.240492505	8.47451131	0.003601571	0.011539312
ENSRNOG00000002541	-0.231112508	5.640645611	8.47050503	0.003609512	0.011560076
ENSRNOG00000011280	-0.304248574	3.916232427	8.468684116	0.003613127	0.011569313
ENSRNOG00000021013	-0.219851118	4.208330855	8.456466156	0.003637479	0.011635524
ENSRNOG00000002361	-0.26823175	4.093101379	8.456742014	0.003636927	0.011635524
ENSRNOG00000012325	-0.225912202	5.67849705	8.456896849	0.003636618	0.011635524
ENSRNOG00000055171	-0.72718428	0.314729392	8.452923289	0.003644572	0.011653503
ENSRNOG00000051024	-0.229671046	5.553419948	8.449937847	0.003650559	0.011665644
ENSRNOG00000004807	-0.188390048	5.042418345	8.450288968	0.003649854	0.011665644
ENSRNOG00000039379	-0.44130203	0.730766393	8.449927956	0.003650579	0.011665644
ENSRNOG00000005528	-0.191258652	5.253923132	8.446640737	0.003657183	0.011682034
ENSRNOG00000018091	-0.209345301	7.900058343	8.442397316	0.003665727	0.011706962
ENSRNOG00000017166	-0.411034219	3.069979908	8.435703303	0.003679246	0.011747766
ENSRNOG00000053223	-0.321913161	2.36964578	8.433926616	0.003682842	0.011755545
ENSRNOG00000033837	-0.482224867	3.935127545	8.432243133	0.003686253	0.011760656
ENSRNOG00000004059	-0.391137818	1.985042949	8.431513665	0.003687732	0.011763004
ENSRNOG00000051321	-0.493937857	0.470105335	8.429867248	0.003691073	0.011771289
ENSRNOG00000003689	-0.172518802	7.229753862	8.428258748	0.003694339	0.011779334
ENSRNOG00000006076	-0.424284278	4.923875768	8.423412399	0.003704199	0.011806017
ENSRNOG00000056421	-0.797219305	0.896657304	8.412156687	0.003727202	0.011876698
ENSRNOG00000008049	-0.157628924	5.885179055	8.411828109	0.003727876	0.011876698
ENSRNOG00000015477	-0.824465573	-0.612407728	8.409339655	0.003732982	0.011888182
ENSRNOG00000010480	-0.356376281	3.009517286	8.408440794	0.003734828	0.01189167
ENSRNOG00000016034	-0.193003717	5.225979572	8.40109819	0.003749944	0.011934998
ENSRNOG00000006112	-0.261576931	3.006643097	8.399024363	0.003754224	0.01194622
ENSRNOG00000052787	-0.219370996	5.630224194	8.37337912	0.00380757	0.012101377
ENSRNOG00000049281	-0.22129382	6.494086559	8.367546785	0.003819809	0.012135405
ENSRNOG00000024372	-0.285529824	5.238874716	8.366922181	0.003821122	0.012137142
ENSRNOG00000057249	-0.639628994	-0.557229878	8.363623358	0.003828065	0.012154318
ENSRNOG00000042224	-0.293644414	4.119412391	8.361497494	0.003832546	0.012165478
ENSRNOG00000018715	-0.481407765	0.551883479	8.361226902	0.003833117	0.012165478
ENSRNOG00000020660	-0.213854176	9.234627747	8.346671439	0.003863945	0.012253497
ENSRNOG00000005908	-0.307209701	6.462518708	8.336517025	0.003885602	0.012314776
ENSRNOG00000026807	-0.318256547	2.051888895	8.336700431	0.00388521	0.012314776
ENSRNOG00000006328	-0.245270923	5.640499811	8.32697	0.003906075	0.01237471
ENSRNOG00000000942	-0.224277233	4.601954389	8.321350751	0.003918177	0.012408084
ENSRNOG00000060203	-0.912141213	1.074041716	8.310436957	0.003941791	0.012472884
ENSRNOG00000061011	-0.776794365	-0.977598653	8.296310999	0.003972569	0.012552716
ENSRNOG00000005326	-0.25212356	5.833933647	8.296466865	0.003972228	0.012552716
ENSRNOG00000018164	-0.198548955	6.682357799	8.288801155	0.003989032	0.012599706
ENSRNOG00000010756	-0.315099227	4.334009335	8.285111169	0.003997146	0.012617784
ENSRNOG00000027921	-0.257434017	5.3089355	8.284179058	0.003999198	0.012621747
ENSRNOG00000022492	-0.811559707	-1.118364464	8.275557927	0.004018231	0.012676763
ENSRNOG00000030572	-0.242213177	5.802736428	8.255693328	0.004062439	0.012811125
ENSRNOG00000014798	-0.168613127	6.556122351	8.252251751	0.004070148	0.01283288
ENSRNOG00000059741	-0.686761303	0.025149868	8.248127359	0.004079406	0.01285951
ENSRNOG00000050849	-0.240261778	5.176089588	8.246896952	0.004082173	0.012865669
ENSRNOG00000020073	-0.367532737	3.068743437	8.228771063	0.004123144	0.012992211
ENSRNOG00000048708	-0.244077812	3.532456434	8.227545686	0.004125929	0.012997508
ENSRNOG00000047800	-0.613699758	0.569162513	8.226817762	0.004127584	0.012998442
ENSRNOG00000061741	-0.794990433	-0.537413007	8.224128159	0.004133706	0.013012545
ENSRNOG00000022320	-0.479274041	1.3410729	8.222339469	0.004137782	0.013022788
ENSRNOG00000024056	-0.196852752	4.315652013	8.218491476	0.004146566	0.013044735
ENSRNOG00000004198	-0.359190188	4.699684905	8.217841541	0.004148051	0.013044735
ENSRNOG00000018760	-0.469219216	3.090816058	8.216568653	0.004150962	0.013051296
ENSRNOG00000047606	-0.574985336	0.690658531	8.210787977	0.004164206	0.013087742
ENSRNOG00000004040	-0.239713069	3.595657788	8.209024881	0.004168255	0.013097865
ENSRNOG00000011603	-0.232015127	4.951434488	8.201537078	0.004185492	0.01314942
ENSRNOG00000033906	-0.380465393	5.13924461	8.196042021	0.004198188	0.013184549
ENSRNOG00000023605	-0.211785169	6.133438695	8.195976689	0.004198339	0.013184549
ENSRNOG00000013756	-0.235715018	3.744297402	8.194193119	0.004202468	0.0131949
ENSRNOG00000054315	-0.561110222	-0.246041028	8.193014779	0.004205198	0.013198836
ENSRNOG00000031478	-0.619697217	1.754917988	8.19293258	0.004205389	0.013198836
ENSRNOG00000004091	-0.2238841	3.951451655	8.181810168	0.004231252	0.013272115
ENSRNOG00000018623	-0.23473994	5.578934822	8.176580799	0.004243467	0.013302526
ENSRNOG00000018881	-0.363027452	6.850852444	8.164249798	0.004272414	0.013377379
ENSRNOG00000059491	-0.268791112	5.012195506	8.161098451	0.004279844	0.013392698
ENSRNOG00000013438	-0.184971452	5.069896043	8.161228522	0.004279537	0.013392698
ENSRNOG00000002420	-0.193464457	5.062626062	8.158354289	0.004286325	0.013410328
ENSRNOG00000018297	-0.332686861	4.079449943	8.141052816	0.004327415	0.013525521
ENSRNOG00000040266	-0.411336916	2.83059226	8.134135573	0.004343955	0.01357186
ENSRNOG00000011513	-0.250771969	3.927604279	8.132106825	0.004348818	0.013584374
ENSRNOG00000002342	-0.194543499	6.053542915	8.131014022	0.00435144	0.013589883
ENSRNOG00000021719	-0.355912771	5.111146988	8.117098197	0.004384969	0.013686079
ENSRNOG00000014691	-0.203568485	5.937986453	8.107075265	0.004409281	0.013754247
ENSRNOG00000020925	-0.318039062	3.757366445	8.092715665	0.004444352	0.013855457
ENSRNOG00000053362	-0.207374946	9.193754641	8.074399264	0.004489499	0.013985191
ENSRNOG00000036484	-0.91702591	0.248935835	8.071880331	0.004495744	0.013999137
ENSRNOG00000051152	-0.463388951	0.930267344	8.068704657	0.00450363	0.014020936
ENSRNOG00000026797	-0.51877455	0.153702463	8.067851567	0.004505751	0.014024778
ENSRNOG00000007128	-0.269839289	6.240390748	8.056728736	0.004533496	0.014097286

ENSRNOG00000060168	-0.207090927	4.154149566	8.045628336	0.004561359	0.014172796
ENSRNOG00000047939	-0.936872911	0.127831615	8.040663992	0.004573877	0.014206113
ENSRNOG00000039649	-0.815958896	1.035141493	8.037018587	0.004583091	0.014222809
ENSRNOG00000042492	-0.251260596	6.102265311	8.036760631	0.004583744	0.014222809
ENSRNOG00000011937	-0.27677857	5.795887299	8.021654861	0.004622132	0.014336304
ENSRNOG00000020244	-0.500180291	0.684325342	8.020970513	0.004623879	0.014338914
ENSRNOG00000005506	-0.3772119	1.385235982	8.017839212	0.00463188	0.014360914
ENSRNOG00000048725	-0.334404222	2.685542005	8.013964446	0.004641801	0.014386039
ENSRNOG00000011254	-0.195871019	3.831923776	8.010284681	0.004651243	0.01440966
ENSRNOG00000025189	-0.333336243	1.985866547	8.007337041	0.00465882	0.014427489
ENSRNOG00000009447	-0.35407777	1.176042007	8.001661584	0.004673444	0.014469949
ENSRNOG00000003732	-0.394106548	4.430077426	8.00126599	0.004674466	0.014470281
ENSRNOG00000003755	-0.454211862	1.685111091	7.999931429	0.004677912	0.01447812
ENSRNOG00000003563	-0.195764581	5.604415718	7.991511896	0.004699715	0.014539917
ENSRNOG00000060574	-0.462701988	0.861608724	7.991862693	0.004698805	0.014539917
ENSRNOG00000010799	-0.276855703	4.932450477	7.988080204	0.004708632	0.014561811
ENSRNOG00000053366	-0.287560085	4.189830844	7.981367205	0.004726124	0.014607347
ENSRNOG00000022323	-0.22966647	6.059982655	7.977888325	0.004735214	0.014632589
ENSRNOG00000012616	-0.264310886	6.980667735	7.977500508	0.004736229	0.014632868
ENSRNOG00000018681	-0.410388863	1.462153623	7.975238636	0.00474215	0.01464566
ENSRNOG00000000137	-0.418999039	1.607543763	7.975212325	0.004742219	0.01464566
ENSRNOG00000005256	-0.235075888	6.280746466	7.972863785	0.004748376	0.014661814
ENSRNOG00000050995	-0.458882345	1.126349178	7.970288886	0.004755135	0.01467696
ENSRNOG00000006471	-0.904190608	4.701364224	7.966576384	0.004764898	0.014701362
ENSRNOG00000011953	-0.195220008	6.684214854	7.959681391	0.004783083	0.014751722
ENSRNOG00000041411	-0.3159821	5.346491792	7.950527589	0.004807336	0.014809214
ENSRNOG00000018884	-0.182674299	4.699314954	7.950738541	0.004806776	0.014809214
ENSRNOG00000003887	-0.424853671	6.696663871	7.950068388	0.004808556	0.01481009
ENSRNOG00000000098	-0.183491594	5.916615608	7.946922585	0.004816922	0.014830087
ENSRNOG00000016221	-0.3057306	6.441539548	7.944067977	0.004824526	0.014844839
ENSRNOG00000007937	-0.207777897	5.915120727	7.94328136	0.004826623	0.014848408
ENSRNOG00000057398	-0.659269184	-0.570340827	7.939295831	0.004837265	0.014878256
ENSRNOG00000036664	-0.185414491	5.802686418	7.937368729	0.00484242	0.014891216
ENSRNOG00000052300	-0.56766882	0.001816276	7.935769089	0.004846702	0.014900119
ENSRNOG00000011402	-0.266957025	2.853454215	7.935584472	0.004847197	0.014900119
ENSRNOG00000021270	-0.276528364	3.990682997	7.93418162	0.004850956	0.014905888
ENSRNOG00000012375	-0.248991705	3.921615197	7.934439195	0.004850266	0.014905888
ENSRNOG00000000150	-0.239544024	3.184446231	7.929191693	0.004864352	0.014941252
ENSRNOG00000007461	-0.52654195	0.249419468	7.926571191	0.004871402	0.014960005
ENSRNOG00000003597	-0.255494116	8.098818002	7.925839266	0.004873373	0.014963156
ENSRNOG00000002874	-0.231738836	4.688561318	7.925181521	0.004875145	0.014965356
ENSRNOG00000045837	-0.497558857	0.17764937	7.921249088	0.004885753	0.014989539
ENSRNOG00000008652	-0.261799798	5.520326226	7.915074221	0.004902457	0.015034961
ENSRNOG00000053349	-0.632634809	-0.387804523	7.909532174	0.004917498	0.01507817
ENSRNOG00000055756	-0.253149758	6.065564296	7.893318452	0.004961775	0.015205097
ENSRNOG00000006418	-0.300353562	3.151312673	7.890595399	0.00496925	0.01522506
ENSRNOG00000021110	-0.224122702	7.476252533	7.877354244	0.005005766	0.015331006
ENSRNOG00000039473	-1.062159323	6.054154871	7.874412702	0.005013915	0.015350588
ENSRNOG00000010363	-0.218293175	4.492369401	7.87434645	0.005014099	0.015350588
ENSRNOG00000000869	-0.268319248	3.453260194	7.870064874	0.005025985	0.015381029
ENSRNOG00000001982	-0.215494923	4.470155576	7.864530522	0.005041391	0.015422216
ENSRNOG00000016877	-0.456472317	7.410105572	7.862020815	0.005048394	0.015437672
ENSRNOG00000010008	-0.17533971	5.068728113	7.833437679	0.005128846	0.015668561
ENSRNOG00000059779	-0.442755133	1.140494861	7.829492049	0.005140054	0.015699772
ENSRNOG00000002735	-0.207122721	7.322093343	7.820420765	0.005165917	0.015763563
ENSRNOG00000014422	-0.247764143	4.247633103	7.813106994	0.005186865	0.01581834
ENSRNOG00000060075	-0.410214994	0.976146814	7.810873145	0.005193281	0.015834856
ENSRNOG00000010034	-0.222285716	6.364611955	7.80756457	0.005202798	0.015860819
ENSRNOG00000015375	-0.220227311	3.638829278	7.79744017	0.00523203	0.015937662
ENSRNOG00000010103	-0.158668443	7.650415652	7.796346371	0.005235199	0.015944246
ENSRNOG00000008079	-0.217866283	6.40040539	7.777915898	0.005288878	0.016095347
ENSRNOG00000019232	-0.237762252	4.182608453	7.778555897	0.005287004	0.016095347
ENSRNOG00000001823	-0.217812296	4.589650147	7.777121408	0.005291204	0.016097791
ENSRNOG00000011263	-0.856685261	1.201017664	7.776757171	0.005292271	0.016097791
ENSRNOG00000026171	-0.23300139	5.852884518	7.774644513	0.005298464	0.01610904
ENSRNOG00000005691	-0.287209733	2.623314288	7.773144526	0.005302866	0.016116721
ENSRNOG00000003779	-0.239664311	4.204934869	7.773089828	0.005303026	0.016116721
ENSRNOG00000018647	-0.21211055	5.325870285	7.769776998	0.005312761	0.01614011
ENSRNOG00000024918	-0.306530011	2.515873113	7.76978859	0.005312727	0.01614011
ENSRNOG00000019243	-0.244050097	3.500813621	7.762642625	0.005333786	0.01619156
ENSRNOG00000010532	-0.297953481	1.970456088	7.753756111	0.005360095	0.016262069
ENSRNOG00000018033	-0.205968566	5.860084039	7.753261516	0.005361563	0.016263407
ENSRNOG00000007053	-0.282334095	3.280540913	7.751484453	0.005366841	0.016276299
ENSRNOG00000011606	-0.524163064	-0.23369626	7.742940566	0.005392292	0.016340967
ENSRNOG00000014616	-0.227390651	5.689592232	7.739600207	0.005402276	0.01636809
ENSRNOG00000005196	-0.227675492	6.926966833	7.738641666	0.005405144	0.016373648
ENSRNOG00000024101	-0.168567208	5.927907293	7.738171024	0.005406553	0.016374783
ENSRNOG00000061167	-0.653967267	-0.642579449	7.731099136	0.00542777	0.016435897
ENSRNOG00000009968	-0.220232938	4.872191288	7.727952184	0.005437238	0.016461421
ENSRNOG00000020394	-0.232056891	4.692651833	7.727091791	0.00543983	0.01646545
ENSRNOG00000010914	-0.316255503	5.300929932	7.726820033	0.005440649	0.01646545
ENSRNOG00000001414	-0.473985142	2.043875156	7.726349267	0.005442068	0.016466596
ENSRNOG00000057851	-0.41480021	2.171906054	7.724943438	0.005446307	0.016476274
ENSRNOG00000011105	-0.64815273	3.509418817	7.707484143	0.005499234	0.016609684
ENSRNOG00000058688	-0.657925958	-0.211724841	7.706166747	0.005503249	0.016612798
ENSRNOG00000014089	-0.261236658	2.873628684	7.704981524	0.005506864	0.01661503
ENSRNOG00000013596	-0.253549561	3.842575135	7.704450676	0.005508483	0.016616751
ENSRNOG00000002595	-0.281161273	6.197168066	7.695452671	0.005536012	0.016690255
ENSRNOG00000018119	-0.277498166	5.490122374	7.694492603	0.005538958	0.016691619
ENSRNOG00000007859	-0.22822743	5.636739175	7.694274544	0.005539627	0.016691619
ENSRNOG00000061917	-0.593439495	-0.384823061	7.693075206	0.005543309	0.016699536
ENSRNOG00000060414	-0.905271103	-0.689766718	7.685728547	0.005565921	0.016758088
ENSRNOG00000017372	-0.259702632	4.839468344	7.676898976	0.00559322	0.016833881
ENSRNOG00000038789	-0.301358356	2.236252569	7.674071188	0.005601992	0.016857078
ENSRNOG00000016137	-0.226018943	8.00616031	7.670736517	0.005612354	0.016881843
ENSRNOG00000024557	-0.337270041	1.740785985	7.670868212	0.005611945	0.016881843
ENSRNOG00000002207	-0.257442317	5.129291749	7.667977603	0.005620942	0.016904464
ENSRNOG00000006792	-0.236101234	4.877156493	7.667536786	0.005622316	0.016905383
ENSRNOG00000042912	-0.390446112	2.552093136	7.666716159	0.005624873	0.016909862
ENSRNOG00000022640	-0.785237898	-1.085966084	7.659952617	0.005645999	0.016970149

ENSRNOG00000003122	-0.205422786	3.585698586	7.656848838	0.00565572	0.016992917
ENSRNOG00000011279	-0.389174136	2.765794978	7.651966897	0.005671046	0.017035731
ENSRNOG00000010316	-0.380141919	1.925829273	7.640318349	0.005707784	0.01713634
ENSRNOG00000019542	-0.93199005	0.765536073	7.636298558	0.005720519	0.017164808
ENSRNOG00000005963	-0.177753852	6.495784457	7.621136319	0.005768813	0.017296607
ENSRNOG00000021392	-0.179375355	6.044740628	7.618606838	0.00577691	0.017314327
ENSRNOG00000023172	-0.505159961	0.526070615	7.617061807	0.005781861	0.017325888
ENSRNOG00000010945	-0.179969031	9.398802019	7.610681814	0.005802354	0.017380718
ENSRNOG00000018273	-0.203626314	8.384771501	7.604705597	0.005821617	0.017428271
ENSRNOG00000049083	-0.225439153	6.454446797	7.596618217	0.005847788	0.017493649
ENSRNOG00000036913	-0.240199343	4.674283927	7.593125373	0.005859129	0.017524262
ENSRNOG00000032576	-0.218821856	6.468274385	7.591613371	0.005864045	0.017535653
ENSRNOG00000051082	-0.491766127	0.868172484	7.589058869	0.00587236	0.017555667
ENSRNOG00000051521	-0.759861976	-0.986605954	7.587078172	0.005878815	0.017569868
ENSRNOG00000008741	-0.186866588	7.959064104	7.585367402	0.005884397	0.017583231
ENSRNOG00000019724	-0.169447521	7.360438134	7.583750176	0.005889678	0.017595692
ENSRNOG00000008755	-0.170361579	7.350552263	7.580738798	0.005899526	0.017621786
ENSRNOG00000052407	-0.459530524	0.926531567	7.578261628	0.005907639	0.017642691
ENSRNOG00000030101	-0.536075992	3.370585484	7.57186079	0.005928655	0.017692106
ENSRNOG00000059057	-0.227341187	3.720448511	7.572168133	0.005927644	0.017692106
ENSRNOG00000052319	-0.396439672	4.175614724	7.567182735	0.005944062	0.017734743
ENSRNOG00000032919	-0.220742281	3.30148807	7.559155705	0.005970596	0.017807198
ENSRNOG00000005923	-0.292745988	6.311942983	7.557360595	0.005976546	0.017818232
ENSRNOG00000045728	-0.415431101	3.608135576	7.555580039	0.005982454	0.017832488
ENSRNOG00000060475	-0.667832823	-0.806088446	7.545104131	0.006017334	0.017922963
ENSRNOG00000000274	-0.277507232	7.190827533	7.541222795	0.00603031	0.017958235
ENSRNOG00000008846	-0.416013024	3.028214406	7.538849027	0.00603826	0.017978528
ENSRNOG00000055661	-0.318685424	3.737795106	7.527450911	0.006076583	0.018081516
ENSRNOG00000001099	-0.239040078	3.071548717	7.526864937	0.00607856	0.018081516
ENSRNOG00000028196	-0.331757495	1.776144519	7.523773428	0.006089	0.018106003
ENSRNOG00000023304	-0.500086108	1.349582743	7.513321758	0.006124432	0.018196772
ENSRNOG00000020315	-0.308372521	5.546528401	7.50830152	0.006141526	0.018241403
ENSRNOG00000035596	-0.610336456	0.203357317	7.506654928	0.006147143	0.018250541
ENSRNOG00000059957	-0.443959966	1.281333164	7.506386266	0.00614806	0.018250541
ENSRNOG00000054479	-0.189701314	3.876326243	7.503619884	0.006157511	0.018275168
ENSRNOG00000010537	-0.183995721	4.493677765	7.492026662	0.006197277	0.01837253
ENSRNOG00000010117	-0.196729891	7.840731588	7.484875367	0.006221938	0.018435284
ENSRNOG00000000244	-0.209329663	3.568696636	7.485210993	0.006220778	0.018435284
ENSRNOG00000003687	-0.387495496	5.514354791	7.483717084	0.006225941	0.018443696
ENSRNOG00000013035	-0.201408914	4.554036983	7.482474751	0.006230238	0.018452401
ENSRNOG00000015969	-0.235585298	3.924808722	7.481857015	0.006232376	0.018452401
ENSRNOG00000022764	-0.382210838	5.959813335	7.480157941	0.00623826	0.018466369
ENSRNOG00000060479	-0.252165577	4.77920608	7.477380693	0.00624789	0.018487962
ENSRNOG00000051029	-0.69771362	-0.821671632	7.477674899	0.006246869	0.018487962
ENSRNOG00000006001	-0.206552887	6.57746886	7.468519088	0.006278718	0.018565307
ENSRNOG00000016610	-0.270729119	6.085454234	7.466499906	0.006285764	0.018582671
ENSRNOG00000019384	-0.272749784	2.821235356	7.465324921	0.006289868	0.018591332
ENSRNOG00000006067	-0.213877399	4.757202937	7.453701906	0.006330612	0.018704778
ENSRNOG00000056371	-0.194792954	6.516637037	7.446994553	0.006354246	0.018771108
ENSRNOG00000049052	-0.341975181	3.491742622	7.433418999	0.006402359	0.018902656
ENSRNOG00000001032	-0.210890587	4.998677818	7.43351839	0.006402005	0.018902656
ENSRNOG00000013320	-0.487789777	3.216186968	7.432046026	0.006407245	0.018913557
ENSRNOG00000033747	-0.358242317	2.431940422	7.427403782	0.006423795	0.018958878
ENSRNOG00000036917	-0.243227761	4.365117246	7.421354579	0.006445427	0.01901209
ENSRNOG00000053943	-0.173761777	6.147190158	7.421616944	0.006444488	0.01901209
ENSRNOG00000043342	-0.732398187	-0.767565824	7.420623159	0.006448048	0.019016277
ENSRNOG00000017254	-0.177295603	5.507664581	7.41561457	0.006466022	0.019065735
ENSRNOG00000012419	-0.278152818	2.586995393	7.414352272	0.006470561	0.019075564
ENSRNOG00000001758	-0.235131367	3.983668741	7.399042226	0.006525861	0.019227854
ENSRNOG00000055825	-0.738779636	-1.143686717	7.397755691	0.00653053	0.019236788
ENSRNOG00000023920	-0.333755984	1.889260199	7.397537338	0.006531323	0.019236788
ENSRNOG00000022845	-0.32827385	4.098140123	7.396383945	0.006535512	0.019245546
ENSRNOG00000061508	-0.329229837	4.686752327	7.391914497	0.006551772	0.019286252
ENSRNOG00000004688	-0.269310331	5.75726991	7.375390121	0.006612245	0.019453415
ENSRNOG00000007283	-0.279151582	6.845710103	7.371673846	0.006625923	0.019490036
ENSRNOG00000059509	-0.399951546	0.995036929	7.365759867	0.00664775	0.019546978
ENSRNOG00000007430	-0.266645139	3.681708055	7.363742595	0.006655212	0.019562322
ENSRNOG00000023100	-0.415946715	1.524132645	7.362465896	0.006659939	0.019571915
ENSRNOG00000003542	-0.610333636	6.333758416	7.349777128	0.006707107	0.019699561
ENSRNOG00000011584	-0.204773887	4.319381674	7.349395871	0.00670853	0.019700085
ENSRNOG00000022555	-0.487473364	0.940419014	7.346578136	0.006719052	0.019727327
ENSRNOG00000015339	-0.171983584	5.72148405	7.342932313	0.006732693	0.019763711
ENSRNOG00000022748	-0.314634472	4.549350115	7.334643989	0.006763807	0.019851367
ENSRNOG00000019706	-0.81015035	-0.671828552	7.32220871	0.006810766	0.01998178
ENSRNOG00000048088	-0.296483827	4.655539622	7.320147033	0.006818583	0.020001009
ENSRNOG00000002389	-0.210134916	8.362910804	7.318457456	0.006824997	0.020012407
ENSRNOG00000030328	-0.223165737	5.155109666	7.316828261	0.006831187	0.020023142
ENSRNOG00000010608	-0.247003921	3.498602441	7.309125777	0.00686053	0.02009427
ENSRNOG00000028627	-0.302588232	2.932307899	7.304210277	0.006879323	0.020138139
ENSRNOG00000011284	-0.403982662	1.589782771	7.288583935	0.006939415	0.020304767
ENSRNOG00000008244	-0.242825067	3.958886518	7.288296796	0.006940524	0.020304767
ENSRNOG00000020946	-0.225974278	4.327659881	7.288077078	0.006941373	0.020304767
ENSRNOG00000021735	-0.315931223	3.159804053	7.286592311	0.006947113	0.02031405
ENSRNOG00000006779	-0.271923218	5.128041917	7.274502265	0.006994027	0.020431163
ENSRNOG00000017765	-0.236233842	4.452881166	7.264245024	0.007034083	0.020526663
ENSRNOG00000000808	-0.231999772	5.281284856	7.262127569	0.007042381	0.020547091
ENSRNOG00000059702	-0.326984002	3.607940498	7.26160522	0.00704443	0.020549282
ENSRNOG00000036942	-0.836948319	-0.8239842	7.260270396	0.007049668	0.020560773
ENSRNOG00000016849	-0.257394769	6.205249199	7.256582503	0.007064159	0.020599245
ENSRNOG00000003354	-0.601841871	-0.439173508	7.254333337	0.007073012	0.020621263
ENSRNOG00000055471	-0.241794443	9.488382629	7.25206697	0.007081945	0.020643504
ENSRNOG00000048932	-0.51351079	-0.142303748	7.25001262	0.007090051	0.02066333
ENSRNOG00000036918	-0.377060935	2.35586699	7.249441609	0.007092306	0.020666098
ENSRNOG00000025317	-0.166308358	5.337133511	7.24804444	0.007097827	0.020678379
ENSRNOG00000006989	-0.184251018	9.715507095	7.244352878	0.007112434	0.020717124
ENSRNOG00000007748	-0.525070637	2.59085822	7.239928753	0.007129981	0.020753704
ENSRNOG00000035546	-0.461852467	0.11521231	7.23986466	0.007130235	0.020753704
ENSRNOG00000024934	-0.174192546	5.198582874	7.235869732	0.007146118	0.020792289
ENSRNOG00000046862	-0.322401101	2.618262782	7.23330638	0.007156329	0.020818172
ENSRNOG00000015695	-0.351263085	1.765108622	7.231445657	0.00716375	0.020835932

ENSRNOG00000012228	-0.269555906	3.909708605	7.230915203	0.007165867	0.020838261
ENSRNOG00000007076	-0.278479499	2.853464088	7.22809622	0.007177128	0.020867176
ENSRNOG00000021614	-0.215300829	7.165000264	7.224027676	0.007193412	0.020903008
ENSRNOG00000041989	-0.326894038	4.16667865	7.218648976	0.007214999	0.020961889
ENSRNOG00000030954	-0.284251509	7.561852933	7.217376555	0.007220115	0.020972906
ENSRNOG00000015957	-0.41955257	1.331701837	7.214074928	0.007233408	0.021004675
ENSRNOG00000059656	-0.480874821	2.500019847	7.214001219	0.007233706	0.021004675
ENSRNOG00000006503	-0.415194163	2.42161424	7.205076846	0.007269764	0.021093905
ENSRNOG00000020897	-0.204886506	7.83228349	7.192068254	0.007322653	0.021235694
ENSRNOG00000012219	-0.178245702	4.391133906	7.191100358	0.007326604	0.02124326
ENSRNOG00000017952	-0.238438203	7.476570347	7.188252758	0.00733824	0.02126921
ENSRNOG00000027808	-0.65788146	-1.019572367	7.184566594	0.007353331	0.02130905
ENSRNOG00000011543	-0.261203811	4.124377663	7.178850214	0.007376797	0.021369228
ENSRNOG00000018288	-0.264518226	6.052548765	7.178479183	0.007378322	0.021369738
ENSRNOG00000048719	-0.247506415	4.27758957	7.177595982	0.007381956	0.02137635
ENSRNOG00000007719	-0.209368249	5.585522591	7.172874314	0.007401409	0.021420931
ENSRNOG00000037251	-0.235295799	3.92047087	7.170382353	0.007411698	0.021446787
ENSRNOG00000058643	-0.245572032	4.924150601	7.166313294	0.007428529	0.021483712
ENSRNOG00000024629	-0.205608943	7.092816039	7.16530298	0.007432714	0.021491889
ENSRNOG00000001652	-0.330292422	2.673463698	7.164788632	0.007434845	0.021494128
ENSRNOG00000060153	-0.316516601	2.908833153	7.160309921	0.007453432	0.021539996
ENSRNOG00000042717	-0.341003506	3.194466303	7.156694138	0.007468472	0.021574827
ENSRNOG00000056214	-0.555581735	0.609547126	7.151865823	0.007488604	0.021617968
ENSRNOG00000007049	-0.449366778	4.884462297	7.151370533	0.007490672	0.021619998
ENSRNOG00000024870	-0.329367296	6.771263434	7.147480237	0.007506938	0.021662996
ENSRNOG00000056847	-0.671054076	0.651480979	7.126545218	0.007595093	0.02190541
ENSRNOG00000061999	-0.56238939	-0.171893452	7.122172454	0.007613639	0.021942913
ENSRNOG00000033809	-0.225918361	3.169597934	7.114741945	0.007645261	0.02202603
ENSRNOG00000018069	-0.201098813	4.876170222	7.111614249	0.007658611	0.022056466
ENSRNOG00000009163	-0.231537301	4.259608917	7.108883674	0.007670286	0.022078096
ENSRNOG00000052721	-0.211484125	5.18453826	7.108879345	0.007670305	0.022078096
ENSRNOG00000006082	-0.314191743	1.905126593	7.108210969	0.007673165	0.022079712
ENSRNOG00000038597	-0.445680804	8.000703697	7.108096504	0.007673655	0.022079712
ENSRNOG00000011414	-0.206728453	6.608516471	7.100215867	0.007707467	0.022153405
ENSRNOG00000004477	-0.199899912	6.367453576	7.097784061	0.007717931	0.022178897
ENSRNOG00000009097	-0.175246979	6.888683963	7.095417169	0.00772813	0.022204176
ENSRNOG00000016063	-0.209874886	5.747704691	7.091818617	0.007743663	0.022240732
ENSRNOG00000004602	-0.145372371	6.449017885	7.087737111	0.007761319	0.022279318
ENSRNOG00000004284	-0.421796447	6.035090122	7.080144106	0.007794274	0.02236581
ENSRNOG00000008587	-0.219310665	4.969972328	7.078661636	0.007800725	0.022380265
ENSRNOG00000010552	-0.18450133	4.921732013	7.074197962	0.007820182	0.022419837
ENSRNOG00000008978	-0.229825075	2.945931056	7.07328236	0.007824179	0.022425095
ENSRNOG00000002021	-0.198586135	8.344891136	7.073129088	0.007824848	0.022425095
ENSRNOG00000007327	-0.285131028	2.803680931	7.044127572	0.007952555	0.022754022
ENSRNOG00000005334	-0.317305177	6.535278234	7.042323358	0.00796057	0.022772839
ENSRNOG00000015932	-0.22655182	2.934887758	7.041082285	0.007966088	0.022784508
ENSRNOG00000028996	-0.466265223	1.096649942	7.03994843	0.007971133	0.022794819
ENSRNOG00000013165	-0.178763005	4.026327522	7.028531695	0.00802211	0.022924038
ENSRNOG00000057307	-0.58842414	-0.258983456	7.027743823	0.00802564	0.022929987
ENSRNOG00000020906	-0.446240068	3.395594307	7.027197645	0.008028089	0.022932844
ENSRNOG00000004904	-0.15137278	6.02441197	7.016817181	0.008074764	0.023053699
ENSRNOG00000010038	-0.285942655	7.654993454	7.015298337	0.008081617	0.023069103
ENSRNOG00000018046	-0.289480032	4.839785801	7.009351993	0.008108502	0.023141677
ENSRNOG00000015479	-0.231875032	4.138309234	7.008563945	0.008112072	0.023147694
ENSRNOG00000021015	-0.343495339	2.171212112	6.994767201	0.008174834	0.023318379
ENSRNOG00000019298	-0.166125678	5.836948867	6.99366408	0.008179873	0.023328551
ENSRNOG00000047329	-0.19873493	3.990835384	6.991552873	0.008189527	0.023347673
ENSRNOG00000021302	-0.287830891	5.320188477	6.989706513	0.008197979	0.023367562
ENSRNOG00000011357	-0.42201819	2.299738385	6.983850084	0.008224848	0.023439929
ENSRNOG00000050535	-0.511140884	3.17698585	6.978232094	0.008250707	0.023505164
ENSRNOG00000021469	-0.305308727	2.794923327	6.976363918	0.008259325	0.023524221
ENSRNOG00000004789	-0.238572411	4.484150922	6.976137886	0.008260368	0.023524221
ENSRNOG00000046343	-0.372431909	2.75421588	6.973693556	0.008271659	0.023552139
ENSRNOG00000056648	-0.59428782	-0.07486541	6.967362743	0.008300975	0.023627114
ENSRNOG00000030547	-0.250591798	8.44070863	6.96049332	0.008332905	0.0236967
ENSRNOG00000014525	-0.195393801	7.85383833	6.959631323	0.008336921	0.023699606
ENSRNOG00000026542	-0.219868436	5.838640519	6.959824096	0.008336023	0.023699606
ENSRNOG00000019936	-0.209364166	3.892662779	6.956549377	0.008351294	0.023731945
ENSRNOG00000023085	-0.3001038	3.231457757	6.944737567	0.008406617	0.023864401
ENSRNOG00000010958	-0.201812977	6.03399283	6.941810133	0.008420386	0.02389041
ENSRNOG00000000062	-0.274606195	4.090439275	6.940221387	0.008427868	0.02390664
ENSRNOG00000052695	-0.531778051	0.286542277	6.935887746	0.008448312	0.023956048
ENSRNOG00000030294	-0.185591858	5.703576601	6.932192075	0.008465787	0.023992709
ENSRNOG00000004179	-0.451279904	5.821075295	6.932633832	0.008463696	0.023992709
ENSRNOG00000051222	-0.705629817	1.615108155	6.932305319	0.008465251	0.023992709
ENSRNOG00000008554	-0.227145825	3.721492804	6.925439607	0.00849781	0.024070837
ENSRNOG00000048910	-0.416507463	0.335853254	6.918206626	0.00853225	0.024163047
ENSRNOG00000019697	-0.259393418	6.344250297	6.917940463	0.00853352	0.024163047
ENSRNOG00000008865	-0.468985777	1.949890668	6.91345459	0.008554955	0.024217221
ENSRNOG00000025459	-0.144227402	6.347920588	6.91072209	0.008568038	0.024247778
ENSRNOG00000054999	-0.318775077	1.44730884	6.909506192	0.008573867	0.024257052
ENSRNOG00000058912	-0.214628284	4.489239025	6.909399295	0.008574379	0.024257052
ENSRNOG00000002898	-0.282556779	6.471580764	6.907059585	0.008585607	0.024280139
ENSRNOG00000001573	-0.198998423	4.277628698	6.90237893	0.008608113	0.024335094
ENSRNOG00000006426	-0.231134311	10.00159261	6.901615931	0.008611788	0.024337228
ENSRNOG00000058486	-0.617768012	-0.813859748	6.901583883	0.008611942	0.024337228
ENSRNOG00000007678	-0.231857315	3.714646086	6.899598641	0.00862151	0.024355574
ENSRNOG00000003160	-0.510195838	3.178583035	6.894710994	0.008645114	0.024417897
ENSRNOG00000028993	-0.260905054	5.099749703	6.881783015	0.008707865	0.024586366
ENSRNOG00000053309	-0.351549065	1.827438236	6.878528328	0.008723737	0.024626786
ENSRNOG00000009112	-0.267518607	6.695651879	6.877726295	0.008727652	0.024633448
ENSRNOG00000006126	-0.149165821	5.050473069	6.873964256	0.008746043	0.024672162
ENSRNOG00000057749	-0.329168624	2.655373178	6.87171725	0.008757046	0.024698802
ENSRNOG00000015004	-0.4824393	0.271169719	6.866418522	0.00878305	0.024763321
ENSRNOG00000007701	-0.215753028	5.519745261	6.86605607	0.008784832	0.024763935
ENSRNOG00000000249	-0.486117306	2.714862616	6.865383158	0.00878814	0.024768852
ENSRNOG00000000580	-0.530775049	0.399081829	6.860490238	0.008812237	0.024832347
ENSRNOG00000003201	-0.185048144	7.750527685	6.859847981	0.008815405	0.024836854
ENSRNOG00000042482	-0.168814104	5.32703883	6.854254375	0.008843045	0.024905866
ENSRNOG00000009538	-0.198652619	5.149435063	6.852103739	0.008853696	0.024922564

ENSRNOG00000006617	-0.191167353	6.720227943	6.851302733	0.008857666	0.024929308
ENSRNOG00000005165	-0.206809196	4.951378567	6.848992575	0.008869127	0.024957127
ENSRNOG00000024194	-0.232335644	5.263815842	6.840692036	0.008910431	0.025059995
ENSRNOG00000017282	-0.350144742	2.977760829	6.834109056	0.008943329	0.025139122
ENSRNOG00000014779	-0.201519898	6.541701293	6.830447929	0.008961679	0.02518623
ENSRNOG00000005916	-0.160232162	6.321971285	6.82083433	0.009010047	0.025308689
ENSRNOG00000061432	-0.587255893	0.649572141	6.812716269	0.009051098	0.025410476
ENSRNOG00000036576	-0.189141161	4.631315141	6.811289813	0.009058331	0.025426275
ENSRNOG00000005457	-0.80762915	6.626277996	6.80929855	0.009068438	0.025446179
ENSRNOG00000004016	-0.214292959	5.134300739	6.8091178	0.009069356	0.025446179
ENSRNOG00000032396	-0.630788023	-0.585419267	6.807246154	0.009078867	0.025465861
ENSRNOG00000027962	-0.203358724	6.719015365	6.805685654	0.009086805	0.025476446
ENSRNOG00000029556	-0.309169234	3.344339565	6.804186432	0.009094438	0.025491474
ENSRNOG00000028082	-0.721224375	-0.994817955	6.799924567	0.009116172	0.025543351
ENSRNOG00000017852	-0.200388808	7.487249049	6.798979175	0.009121001	0.025552358
ENSRNOG00000048617	-0.177642443	7.6010274	6.798031773	0.009125842	0.025561398
ENSRNOG00000007416	-0.283661791	2.919280827	6.795765505	0.009137434	0.025589339
ENSRNOG00000006865	-0.329560496	8.151290149	6.794709567	0.00914284	0.025599951
ENSRNOG00000016643	-0.214591719	3.788681057	6.794191344	0.009145494	0.025602855
ENSRNOG00000007699	-0.197319108	5.038558571	6.792698853	0.009153143	0.025619739
ENSRNOG00000055439	-0.397582499	2.021140953	6.78924549	0.009170866	0.025660275
ENSRNOG00000033560	-0.198112743	4.558971074	6.787314989	0.009180789	0.025674429
ENSRNOG00000007532	-0.174974298	5.071671721	6.787399495	0.009180355	0.025674429
ENSRNOG00000020271	-0.232531199	6.212536364	6.781763496	0.009209386	0.025736219
ENSRNOG00000009821	-0.269859957	5.070962591	6.779386304	0.009221659	0.02576597
ENSRNOG00000017195	-0.151180749	6.240757538	6.776875035	0.009234643	0.025797694
ENSRNOG00000012369	-0.308167217	2.335154137	6.772418675	0.009257729	0.025853065
ENSRNOG00000027355	-0.207120907	4.415068005	6.767517081	0.00928319	0.025905891
ENSRNOG00000002253	-0.331636509	1.942345561	6.765665699	0.009292825	0.025928211
ENSRNOG00000007548	-0.206761056	5.250711823	6.765308793	0.009294684	0.025928828
ENSRNOG00000014534	-0.716651451	-0.905824323	6.763092538	0.009306234	0.025951904
ENSRNOG00000035543	-0.489624312	0.578562493	6.757847732	0.009333627	0.02600735
ENSRNOG00000010284	-0.207669084	6.438395582	6.755934716	0.009343639	0.026028708
ENSRNOG00000059202	-0.186405267	6.851191748	6.749885528	0.00937537	0.026098736
ENSRNOG00000016206	-0.202858672	4.896201528	6.748613181	0.009382058	0.026103586
ENSRNOG00000018426	-0.472054187	2.380977929	6.747365136	0.009388623	0.026112675
ENSRNOG00000012236	-0.210282884	4.632888262	6.737911385	0.009438507	0.026242197
ENSRNOG00000037799	-0.429738443	0.602553667	6.735231147	0.009452698	0.026277041
ENSRNOG00000005556	-0.252069191	3.873984351	6.734836774	0.009454788	0.026278237
ENSRNOG00000033790	-0.213678383	8.706249032	6.733859738	0.009459968	0.02628802
ENSRNOG00000055401	-0.48066654	6.268465091	6.733329881	0.009462779	0.026291215
ENSRNOG00000012940	-0.210585058	7.487981468	6.725990681	0.009501794	0.026381099
ENSRNOG00000001270	-0.269905631	3.638691812	6.722973542	0.009517881	0.02642113
ENSRNOG00000001335	-0.19986791	5.000158713	6.710134539	0.009586649	0.026598038
ENSRNOG00000049020	-0.410259357	5.333209377	6.708766451	0.009594007	0.026613788
ENSRNOG00000010495	-0.16672612	5.941585173	6.707483546	0.009600912	0.026628277
ENSRNOG00000055085	-0.728821996	-1.215069624	6.70042653	0.009638985	0.026724512
ENSRNOG00000002713	-0.165195168	4.175138126	6.700096951	0.009640767	0.026724773
ENSRNOG00000039469	-0.377990664	3.473064726	6.697495257	0.009654845	0.02675443
ENSRNOG00000054391	-0.165139357	9.360519799	6.692008915	0.009684601	0.026818114
ENSRNOG00000015274	-0.235646747	4.153752268	6.681869467	0.009739842	0.026947521
ENSRNOG00000011871	-0.325499942	2.71155291	6.678657853	0.009757407	0.026991401
ENSRNOG00000047213	-0.211791858	4.778363397	6.672034532	0.009793733	0.027072968
ENSRNOG00000062277	-0.476443976	0.590500201	6.657902085	0.009871707	0.027278988
ENSRNOG00000016243	-0.474512653	1.90671236	6.655017038	0.009887703	0.027318423
ENSRNOG00000005362	-0.209347161	3.845009973	6.645122652	0.009942763	0.027460965
ENSRNOG00000026942	-0.191695799	5.909361428	6.644129281	0.009948309	0.027471489
ENSRNOG00000006858	-0.231712611	4.854694763	6.64179566	0.009961348	0.027497905
ENSRNOG00000006534	-0.179093243	6.251459155	6.638184309	0.009981561	0.0275441
ENSRNOG00000018936	-0.241093146	3.491969035	6.634422332	0.010002663	0.0275831
ENSRNOG00000004282	-0.404559248	4.503645806	6.635280693	0.009997844	0.0275831
ENSRNOG00000012812	-0.24419407	5.066868303	6.634470131	0.010002394	0.0275831
ENSRNOG00000048990	-0.56925304	0.782892295	6.634593738	0.0100017	0.0275831
ENSRNOG00000020081	-0.27422516	3.73129112	6.629459385	0.01003057	0.027640802
ENSRNOG00000050650	-0.293251613	2.566177928	6.628108792	0.010038179	0.027656206
ENSRNOG00000023021	-0.178618393	4.709446351	6.627847139	0.010039653	0.027656206
ENSRNOG00000058320	-0.459085873	0.29286947	6.625827664	0.010051043	0.027673137
ENSRNOG00000060729	-0.272730484	3.372597982	6.625504727	0.010052865	0.027673343
ENSRNOG00000001490	-0.248678825	3.824253723	6.624027142	0.010061208	0.027691495
ENSRNOG00000007221	-0.208235677	4.201521304	6.61491731	0.010112803	0.027828664
ENSRNOG00000051680	-0.173596737	5.886823362	6.614510132	0.010115116	0.02783019
ENSRNOG00000037406	-0.404606284	1.919921602	6.613018281	0.010123593	0.027848675
ENSRNOG00000001295	-0.227652645	10.63762264	6.607261095	0.010156376	0.027934002
ENSRNOG00000014628	-0.221573459	5.591743328	6.598880299	0.010204292	0.028051173
ENSRNOG00000020411	-0.16742831	6.012163197	6.596202364	0.010219652	0.02808852
ENSRNOG00000039754	-0.314879575	2.469244805	6.586379647	0.010276194	0.028234125
ENSRNOG00000020360	-0.194696038	5.668986351	6.579788765	0.010314313	0.028333941
ENSRNOG00000003955	-0.209046813	4.277753666	6.576513134	0.010333312	0.028371365
ENSRNOG00000033056	-0.280208232	2.67137313	6.576729555	0.010332056	0.028371365
ENSRNOG00000056951	-0.845617342	0.631775447	6.57579622	0.010337475	0.028377874
ENSRNOG00000042821	-0.39596662	7.049080701	6.573188263	0.010352633	0.028414559
ENSRNOG00000011341	-0.174968242	5.584334633	6.572287383	0.010357875	0.028424018
ENSRNOG00000031266	-0.197706824	4.572094999	6.567823728	0.010383885	0.028485522
ENSRNOG00000002312	-0.268234763	3.307091257	6.568036214	0.010382645	0.028485522
ENSRNOG00000022822	-0.430072001	1.227293673	6.545838174	0.010512979	0.02880971
ENSRNOG00000003480	-0.595038836	0.285377503	6.544935198	0.010518316	0.028819348
ENSRNOG00000016717	-0.236692096	3.302432738	6.54343019	0.010527218	0.028833759
ENSRNOG00000026963	-0.192278778	8.859222596	6.541458016	0.010538894	0.028850769
ENSRNOG00000012271	-0.179047364	6.898151281	6.541612036	0.010537982	0.028850769
ENSRNOG00000054047	-0.256605224	4.285696015	6.536413854	0.010568819	0.028927689
ENSRNOG00000009468	-0.364303739	1.297449461	6.534833477	0.010578213	0.028948396
ENSRNOG00000019751	-0.479974644	2.821209202	6.533839414	0.010584126	0.028954569
ENSRNOG00000014613	-0.315158971	7.054570673	6.532137447	0.010594257	0.02897728
ENSRNOG00000037580	-0.147237625	6.090294666	6.528754717	0.010614424	0.029022413
ENSRNOG00000038951	-0.214714503	4.656314479	6.526066408	0.010630479	0.029061293
ENSRNOG00000013328	-0.471395722	0.832253083	6.524879108	0.010637578	0.029070661
ENSRNOG00000059764	-0.163225209	9.358674994	6.522218726	0.010653502	0.029109153
ENSRNOG00000002199	-0.185066616	4.482970913	6.519463066	0.010670021	0.029144232
ENSRNOG00000011849	-0.173166062	7.783947869	6.517469807	0.010681987	0.029161971
ENSRNOG00000018177	-0.78535771	-1.182094655	6.517460841	0.010682041	0.029161971
ENSRNOG00000003048	-0.210482203	5.475995379	6.516431112	0.010688228	0.029173831

ENSRNOG00000033038	-0.471774226	0.292538491	6.505111433	0.010756482	0.029350016
ENSRNOG00000006868	-0.19583229	4.768927356	6.493017975	0.010829897	0.029540153
ENSRNOG00000002569	-0.205600225	5.056218421	6.492314236	0.010834185	0.029546759
ENSRNOG00000012103	-0.577297328	0.208893421	6.491221446	0.010840847	0.029559836
ENSRNOG00000004740	-0.168277276	5.058040313	6.489459446	0.010851597	0.029584054
ENSRNOG00000001043	-0.192916956	3.75725961	6.484737339	0.010880462	0.029647432
ENSRNOG00000002270	-0.181103716	6.789228348	6.484739948	0.010880446	0.029647432
ENSRNOG00000059582	-0.597317168	0.912547757	6.478872566	0.010916421	0.02973518
ENSRNOG00000002632	-0.184204165	7.805726114	6.475752441	0.010935601	0.02977718
ENSRNOG00000003553	-0.390457853	3.095981524	6.461265455	0.01102511	0.029979666
ENSRNOG00000003881	-0.169847889	5.675167923	6.454893326	0.011064719	0.03007704
ENSRNOG00000010750	-0.248896526	3.791323411	6.447906838	0.011108314	0.03018
ENSRNOG00000027259	-0.319128157	3.500889133	6.444891828	0.011127182	0.030226076
ENSRNOG00000019165	-0.162458386	5.563514511	6.439885935	0.011158581	0.030295777
ENSRNOG00000014953	-0.867787161	0.961034396	6.438038909	0.01117019	0.030322095
ENSRNOG00000010706	-0.193338425	4.000352122	6.432958891	0.011202181	0.030398515
ENSRNOG00000051432	-0.777153198	-1.298167336	6.431727551	0.011209949	0.030414383
ENSRNOG00000053200	-0.261208857	6.611616861	6.429101609	0.011226535	0.030448947
ENSRNOG00000010624	-0.191628226	5.023844884	6.410097574	0.011347318	0.030734422
ENSRNOG00000046602	-0.605330075	0.923795715	6.40834195	0.011358543	0.030759564
ENSRNOG00000017243	-0.180659503	6.518024664	6.40167583	0.011401269	0.030854913
ENSRNOG00000033411	-0.278384764	3.82307589	6.401632562	0.011401547	0.030854913
ENSRNOG00000016848	-0.29155296	4.437345632	6.398973391	0.011418637	0.030890604
ENSRNOG00000049150	-0.305916568	3.42431932	6.394542746	0.011447171	0.030962506
ENSRNOG00000018125	-0.16853802	5.116707351	6.393164065	0.011456065	0.030981269
ENSRNOG00000018481	-0.164150895	5.105425092	6.387963895	0.011489675	0.031066856
ENSRNOG00000058386	-0.178363553	5.804758289	6.384483623	0.011512225	0.031122515
ENSRNOG00000020255	-0.275437339	6.932596157	6.380920769	0.011535357	0.031179728
ENSRNOG00000055716	-0.62055215	-0.722795837	6.37944466	0.011544955	0.031200344
ENSRNOG00000016456	-0.334762112	5.789715957	6.377617316	0.011556848	0.031227155
ENSRNOG00000020644	-0.259296073	9.797975345	6.375124025	0.011573095	0.031265721
ENSRNOG00000009110	-0.176557246	5.136709272	6.371829086	0.011594603	0.031318482
ENSRNOG00000021202	-0.166828563	9.930958491	6.360872302	0.011666417	0.031490974
ENSRNOG00000015749	-0.19371186	4.307929617	6.35470296	0.011707054	0.031568375
ENSRNOG00000030058	-0.555426327	0.32278002	6.342119085	0.011790394	0.031766054
ENSRNOG00000043204	-0.212376876	3.221072184	6.341358556	0.01179545	0.031774271
ENSRNOG00000019622	-0.238133023	3.624543184	6.340506134	0.01180112	0.031784137
ENSRNOG00000007743	-0.284466199	4.105549664	6.332179555	0.011856652	0.03192284
ENSRNOG00000046338	-0.29619039	3.061785924	6.331699447	0.011859862	0.031926054
ENSRNOG00000020438	-0.20354213	5.989042927	6.31645901	0.011962227	0.032168802
ENSRNOG00000008908	-0.224338913	4.524593726	6.316479247	0.011962091	0.032168802
ENSRNOG00000033192	-0.488391232	0.778777919	6.316096435	0.011964674	0.032169917
ENSRNOG00000016186	-0.216594831	3.854133405	6.314358974	0.011976404	0.032195989
ENSRNOG00000013636	-0.257257626	3.060995733	6.308564785	0.012015607	0.032295895
ENSRNOG00000019134	-0.492028497	2.41824414	6.308222053	0.01201793	0.032296657
ENSRNOG00000011839	-0.19503861	4.22455707	6.307078112	0.012025687	0.032312018
ENSRNOG00000053480	-0.367181755	0.401001549	6.306285188	0.012031067	0.032320989
ENSRNOG00000004249	-0.329144143	3.313736314	6.301354893	0.012064573	0.032394513
ENSRNOG00000060833	-0.406326431	0.183812809	6.300769772	0.012068555	0.032399713
ENSRNOG00000021938	-0.2389533	4.969186097	6.295339412	0.012105583	0.032493611
ENSRNOG00000005276	-0.18597144	6.389215341	6.291177652	0.01213404	0.032564474
ENSRNOG00000002818	-0.181485248	6.461478776	6.28463093	0.012178944	0.032668371
ENSRNOG00000015029	-0.154463527	5.339824572	6.279406961	0.012214897	0.032748168
ENSRNOG00000020175	-0.165004341	4.145843498	6.267949694	0.012294132	0.032932717
ENSRNOG00000009332	-0.881212223	2.714023502	6.266314745	0.012305482	0.032957545
ENSRNOG00000045781	-0.351498536	0.900864547	6.265551271	0.012310786	0.032966174
ENSRNOG00000033579	-0.45626515	2.259656511	6.263223904	0.012326968	0.033003925
ENSRNOG00000003399	-0.197269788	7.934260462	6.260853292	0.012343474	0.033031361
ENSRNOG00000009740	-0.247307893	6.268643896	6.259399981	0.012353604	0.033047299
ENSRNOG00000039759	-0.2629797	4.50274289	6.256361526	0.01237481	0.033092846
ENSRNOG00000005861	-0.409348435	5.192872814	6.245243225	0.012452728	0.033284351
ENSRNOG00000012681	-0.4990646	3.78209171	6.243201343	0.012467092	0.033311498
ENSRNOG00000012360	-0.212725744	5.213335487	6.241665816	0.012477906	0.033334766
ENSRNOG00000011562	-0.276445658	4.550350144	6.238916447	0.012497291	0.033375293
ENSRNOG00000019341	-0.280725958	2.348828408	6.226479912	0.012585368	0.033593512
ENSRNOG00000020347	-0.179660475	5.887430868	6.224953333	0.012596223	0.03361682
ENSRNOG00000009275	-0.151666647	7.862705736	6.223148857	0.012609067	0.033645426
ENSRNOG00000039593	-0.517267286	-0.452180472	6.212851446	0.012682618	0.033818891
ENSRNOG00000042628	-0.395554559	0.246053855	6.21098312	0.01269601	0.033843203
ENSRNOG00000021678	-0.183982433	4.241417756	6.195084698	0.012810559	0.034117017
ENSRNOG00000026319	-0.206622431	7.470454251	6.194933008	0.012811657	0.034117017
ENSRNOG00000004339	-0.199744508	4.517238002	6.195044911	0.012810847	0.034117017
ENSRNOG00000058034	-0.339075698	5.937072246	6.195768558	0.01280561	0.034117017
ENSRNOG00000018509	-0.420461061	4.678167704	6.185851952	0.012877569	0.034269483
ENSRNOG00000011116	-0.200277386	6.125346203	6.183288894	0.012896235	0.034307625
ENSRNOG00000054929	-0.665063966	-0.149885143	6.175735418	0.012951407	0.03443704
ENSRNOG00000013502	-0.242926663	5.348349603	6.173328861	0.012969035	0.034478124
ENSRNOG00000057389	-0.261227996	2.857154511	6.171192774	0.012984703	0.03450819
ENSRNOG00000011631	-0.561317306	0.565699519	6.161529752	0.013055825	0.03467974
ENSRNOG00000011847	-0.242093265	2.333119245	6.15980127	0.013068589	0.034707823
ENSRNOG00000059446	-0.447527427	0.334415111	6.156466426	0.013093252	0.034761661
ENSRNOG00000052444	-0.34038366	5.021515662	6.151207584	0.01313224	0.034847644
ENSRNOG00000012513	-0.406422632	5.117054338	6.15122289	0.013132127	0.034847644
ENSRNOG00000051483	-0.301786891	7.735722446	6.150227127	0.013139523	0.034861126
ENSRNOG00000058615	-0.643135482	0.068903348	6.149244132	0.013146828	0.034874664
ENSRNOG00000004382	-0.441782495	7.572799994	6.144918277	0.013179026	0.034948366
ENSRNOG00000021729	-0.38399594	4.797336348	6.143061473	0.013192871	0.034976943
ENSRNOG00000006063	-0.187851511	4.67958069	6.139648601	0.013218358	0.035035065
ENSRNOG00000008218	-0.171443771	7.181986555	6.139192468	0.013221768	0.035038239
ENSRNOG00000018166	-0.24846115	5.087737528	6.137721166	0.013232774	0.035061537
ENSRNOG00000059897	-0.649899487	-0.666334202	6.136764531	0.013239935	0.035074642
ENSRNOG00000053599	-0.174264415	6.245473518	6.134070379	0.013260125	0.035116376
ENSRNOG00000018766	-0.213364097	4.674885272	6.124811796	0.013329748	0.035270791
ENSRNOG00000004060	-0.351252347	10.46775405	6.124539942	0.013331798	0.035270791
ENSRNOG00000003549	-0.192293525	5.421792747	6.124166242	0.013334616	0.035272354
ENSRNOG00000054488	-0.313813362	3.625579354	6.121224967	0.013356821	0.035325189
ENSRNOG00000005904	-0.166632391	5.599728703	6.120597583	0.013361562	0.035331827
ENSRNOG00000028557	-0.172899236	4.554268811	6.120268122	0.013364053	0.035332512
ENSRNOG00000017712	-0.730259135	5.591954815	6.115952043	0.013396724	0.035412976

ENSRNOG00000030689	-0.480381976	0.281511344	6.115120968	0.013403024	0.035423716
ENSRNOG00000011339	-0.362903936	6.070955161	6.113991711	0.01341159	0.035440439
ENSRNOG00000038955	-0.539984721	-0.386236434	6.112447663	0.013423311	0.035465494
ENSRNOG00000007917	-0.354509903	1.271434072	6.099730355	0.013520251	0.03568589
ENSRNOG00000010463	-0.488616626	-0.540480487	6.090290974	0.01359267	0.035841188
ENSRNOG00000009346	-0.235585263	3.315164028	6.08652744	0.013621655	0.035911636
ENSRNOG00000017201	-0.166307794	7.088829322	6.084355189	0.013638414	0.035943848
ENSRNOG00000012873	-0.19578029	5.367365873	6.083610924	0.01364416	0.03595301
ENSRNOG00000018294	-0.250681954	8.664732043	6.083220791	0.013647174	0.035954966
ENSRNOG00000018081	-0.242395427	3.319006487	6.081648228	0.013659327	0.035975014
ENSRNOG00000000167	-0.463371778	1.735098766	6.078048631	0.013687188	0.036024424
ENSRNOG00000037645	-0.582224473	-0.366699022	6.077209535	0.013693691	0.03603555
ENSRNOG00000010079	-0.217645068	5.955956393	6.074904423	0.013711572	0.036076609
ENSRNOG00000053450	-0.263826985	5.346122498	6.073072284	0.013725801	0.036096053
ENSRNOG00000009552	-0.180632677	8.106508839	6.073141873	0.01372526	0.036096053
ENSRNOG00000006661	-0.324694726	0.959949207	6.069709758	0.013751956	0.036152825
ENSRNOG00000017645	-0.386913464	1.202374572	6.064743393	0.013790679	0.036248607
ENSRNOG00000012693	-0.147693128	5.745464124	6.062500556	0.013808203	0.036282624
ENSRNOG00000059292	-0.706072791	1.532501138	6.059976374	0.013827953	0.036322464
ENSRNOG00000002653	-0.337838814	6.268497705	6.059281919	0.013833392	0.036330723
ENSRNOG00000001053	-0.158848751	6.766458787	6.055901589	0.013859897	0.036388263
ENSRNOG00000037508	-0.256535022	2.676196324	6.053253901	0.013880694	0.03641871
ENSRNOG00000017899	-0.496772406	1.958912054	6.052176934	0.013889163	0.036434891
ENSRNOG00000013428	-0.341800506	1.976484795	6.049776332	0.013908059	0.036478416
ENSRNOG00000005753	-0.205537566	4.385697549	6.045369537	0.013942814	0.036557464
ENSRNOG00000003453	-0.354023559	7.014387981	6.044890877	0.013946595	0.036561322
ENSRNOG00000004783	-0.250416014	7.32713932	6.038017358	0.014000999	0.036697869
ENSRNOG00000008098	-0.204642206	5.910723549	6.037470135	0.01400534	0.03670317
ENSRNOG00000018967	-0.215913567	4.890004283	6.035287843	0.014022664	0.03674249
ENSRNOG00000060048	-0.170088441	5.989892881	6.034609763	0.014028052	0.036750524
ENSRNOG00000025274	-0.188282662	6.491968938	6.024622405	0.01410765	0.036940718
ENSRNOG00000056474	-0.322402172	3.099714775	6.017691669	0.01416316	0.037067681
ENSRNOG00000013703	-0.292584833	3.143818244	6.010123687	0.01422403	0.037208541
ENSRNOG00000012597	-0.164458822	5.463731707	6.005914799	0.014258	0.03729124
ENSRNOG00000017986	-0.234021599	2.875992304	6.003989992	0.014273562	0.037325778
ENSRNOG00000038489	-0.144527889	6.687596638	5.995192184	0.014344918	0.037493799
ENSRNOG00000053126	-0.560645495	-0.846769325	5.993732244	0.014356795	0.037518648
ENSRNOG00000001599	-0.262389898	4.404569887	5.993265866	0.014360591	0.037519045
ENSRNOG00000002778	-0.25170138	3.295193577	5.992342412	0.01436811	0.037529637
ENSRNOG00000017939	-0.488848819	8.961279321	5.991613096	0.014374052	0.037538964
ENSRNOG00000014478	-0.248381962	6.005758019	5.980072533	0.014468407	0.037766693
ENSRNOG00000037331	-0.376567262	2.395964496	5.977536041	0.014489231	0.037814815
ENSRNOG00000060542	-0.165957874	5.768853233	5.971355876	0.014540097	0.037928812
ENSRNOG00000010280	-0.456329173	6.773457352	5.970664286	0.0145458	0.037931193
ENSRNOG00000051744	-0.273720085	4.522504307	5.970837048	0.014544375	0.037931193
ENSRNOG00000008055	-0.220144936	4.088349491	5.968894671	0.014560405	0.037963025
ENSRNOG00000003248	-0.210563993	5.337550769	5.966550407	0.014579775	0.03800727
ENSRNOG00000051578	-0.316475217	1.608824107	5.963956479	0.014601239	0.038050695
ENSRNOG00000016088	-0.187628443	5.932531045	5.961080046	0.014625079	0.038100281
ENSRNOG00000010475	-0.278417202	4.258281375	5.959844723	0.01463533	0.038108176
ENSRNOG00000026913	-0.224526184	3.881326091	5.957141883	0.014657784	0.038150766
ENSRNOG00000029184	-0.330672806	4.384250572	5.957005853	0.014658915	0.038150766
ENSRNOG00000010154	-0.167367367	4.181415342	5.953523857	0.014687896	0.03821991
ENSRNOG00000053606	-0.414045648	0.790249286	5.949899189	0.014718128	0.038285992
ENSRNOG00000060544	-0.226708971	6.372992254	5.948317575	0.014731339	0.038314065
ENSRNOG00000028219	-0.201167608	3.565492443	5.943672802	0.014770209	0.038406316
ENSRNOG00000031058	-0.468284415	0.745003502	5.943499617	0.01477166	0.038406316
ENSRNOG00000012109	-0.259776809	2.353690921	5.94284667	0.014777133	0.038407932
ENSRNOG00000014668	-0.152832581	4.649214379	5.942867975	0.014776954	0.038407932
ENSRNOG00000006490	-0.243116088	6.629799794	5.942224927	0.014782346	0.038415177
ENSRNOG00000020264	-0.19620841	4.654663055	5.939540345	0.014804879	0.038461109
ENSRNOG00000010985	-0.17166052	4.679482739	5.938830355	0.014810844	0.038463984
ENSRNOG00000047809	-0.549303455	0.505353419	5.938449463	0.014814046	0.038465988
ENSRNOG00000008586	-0.373195209	0.584560646	5.935079525	0.014842399	0.038533292
ENSRNOG00000006986	-0.284860708	1.841836051	5.932593189	0.014863354	0.038581368
ENSRNOG00000024798	-0.332041836	2.444438889	5.928748861	0.014895814	0.038652953
ENSRNOG00000017291	-0.18006637	5.17327954	5.921621488	0.014956188	0.038777842
ENSRNOG00000009609	-0.237875215	2.552550863	5.90684346	0.015082173	0.039085292
ENSRNOG00000021209	-0.203363159	3.578456538	5.905609779	0.01509274	0.039102521
ENSRNOG00000023026	-0.570220832	-0.809895502	5.905490665	0.015093761	0.039102521
ENSRNOG00000003936	-0.209237351	4.267182223	5.903475111	0.015111042	0.039140886
ENSRNOG00000039567	-0.211502751	4.40823572	5.902658182	0.015118052	0.039149729
ENSRNOG00000010735	-0.214237071	5.407038417	5.902126981	0.015122612	0.039151641
ENSRNOG00000042179	-0.361228523	5.231383451	5.90150489	0.015127954	0.039159068
ENSRNOG00000015277	-0.26081923	6.00282455	5.897831414	0.015159539	0.039228
ENSRNOG00000040301	-1.27206193	-1.095524831	5.894875341	0.015185004	0.039268896
ENSRNOG00000024886	-0.223364553	4.51204423	5.894939521	0.015184451	0.039268896
ENSRNOG00000061403	-0.380938311	2.190056933	5.895564426	0.015179064	0.039268896
ENSRNOG00000057245	-0.508642867	-0.230694804	5.893112074	0.015200215	0.03928857
ENSRNOG00000032776	-0.17805656	6.115226509	5.893100583	0.015200315	0.03928857
ENSRNOG00000031222	-0.302869393	5.980273702	5.892434633	0.015206064	0.039297015
ENSRNOG00000012655	-0.498162599	2.670724048	5.888618498	0.015239051	0.039375837
ENSRNOG00000018378	-0.24854807	5.343272345	5.886359506	0.015258613	0.039413519
ENSRNOG00000012162	-0.187783331	5.515645277	5.879720589	0.015316252	0.0395495
ENSRNOG00000014052	-0.311836371	2.410143842	5.877391368	0.015336528	0.039595399
ENSRNOG00000011619	-0.19658257	6.430488731	5.875910482	0.015349433	0.039611772
ENSRNOG00000012594	-0.178323893	5.655615119	5.874359265	0.015362963	0.039637797
ENSRNOG00000003495	-0.173472168	8.217687677	5.873710762	0.015368623	0.03964594
ENSRNOG00000004000	-0.234159803	2.760389588	5.867733945	0.01542089	0.039767812
ENSRNOG00000010155	-0.176978993	7.458150897	5.862393292	0.015467748	0.03987724
ENSRNOG00000009957	-0.32902886	3.015193668	5.856446166	0.0155201	0.039991092
ENSRNOG00000008927	-0.172338812	5.527317945	5.855580926	0.015527732	0.040004248
ENSRNOG00000014582	-0.195620767	4.242159845	5.850208982	0.015575202	0.040113491
ENSRNOG00000010274	-0.340517405	3.693682211	5.849651363	0.015580138	0.040119678
ENSRNOG00000029592	-0.168488254	6.600780357	5.847527099	0.015598956	0.040155076
ENSRNOG00000000007	-0.218467344	9.232979595	5.845431445	0.015617544	0.040189858
ENSRNOG00000001527	-0.466564595	0.616656886	5.845712421	0.015615051	0.040189858
ENSRNOG00000059362	-0.44284717	3.127395044	5.840442241	0.01566189	0.040277791
ENSRNOG00000014834	-0.177195946	4.879090002	5.831912373	0.015738006	0.040466968
ENSRNOG00000020248	-0.365149375	7.621951461	5.830005186	0.015755077	0.040497709

ENSRNOG00000023633	-0.419881447	2.748536373	5.830246303	0.015752918	0.040497709
ENSRNOG00000008516	-0.601236594	-1.167126062	5.829272919	0.015761637	0.040507994
ENSRNOG00000019352	-0.281356448	3.779030181	5.815708095	0.015883657	0.040801724
ENSRNOG00000008027	-0.30979063	1.604731825	5.813904445	0.015899955	0.040823721
ENSRNOG00000045785	-0.377304947	1.458306197	5.814160119	0.015897644	0.040823721
ENSRNOG00000026518	-0.546083113	-0.624701806	5.814317351	0.015896222	0.040823721
ENSRNOG00000033693	-0.32312242	1.785401623	5.810686521	0.015929075	0.040885227
ENSRNOG00000052343	-0.203729812	6.898048679	5.810820307	0.015927863	0.040885227
ENSRNOG00000003600	-0.204691029	4.986503007	5.80797403	0.015953663	0.040941703
ENSRNOG00000020189	-0.19002254	4.007388333	5.807188746	0.015960789	0.040953352
ENSRNOG00000014031	-0.214893884	4.65344122	5.80650934	0.015966957	0.04096254
ENSRNOG00000051868	-0.169328185	4.328708636	5.804198843	0.01598795	0.041009753
ENSRNOG00000061774	-0.453174554	4.347595559	5.799160678	0.016033827	0.041087492
ENSRNOG00000011194	-0.192508722	5.634033204	5.794369038	0.016077584	0.041172968
ENSRNOG00000010601	-0.169082742	7.621810515	5.791127416	0.016107256	0.041228951
ENSRNOG00000011990	-0.652529162	-1.028619093	5.791289781	0.016105769	0.041228951
ENSRNOG00000042726	-0.396565708	0.544954126	5.791314036	0.016105546	0.041228951
ENSRNOG00000002860	-0.183361114	5.62271861	5.790339854	0.016114474	0.041234094
ENSRNOG00000013956	-0.219666579	4.626586022	5.787982784	0.016136095	0.041276257
ENSRNOG00000017841	-0.141515468	5.346797407	5.787975133	0.016136165	0.041276257
ENSRNOG00000019267	-0.169598107	4.803376408	5.78748282	0.016140684	0.04128115
ENSRNOG00000026415	-0.378657291	2.66361876	5.786348498	0.016151103	0.041301125
ENSRNOG00000012454	-0.196975951	3.404209565	5.784766855	0.016165642	0.041324955
ENSRNOG00000009054	-0.395847204	8.065068272	5.784091679	0.016171852	0.041334158
ENSRNOG00000011559	-0.175296817	6.619895516	5.780760627	0.016202528	0.041399199
ENSRNOG00000006553	-0.596106541	-0.107732486	5.779518515	0.016213982	0.041415099
ENSRNOG00000034026	-0.191851774	3.693566221	5.776645228	0.01624051	0.041462793
ENSRNOG00000057476	-0.476881488	-0.12976588	5.773847179	0.016266387	0.041522161
ENSRNOG00000009085	-0.162660071	5.773314641	5.773153406	0.016272809	0.04153186
ENSRNOG00000034269	-0.196075293	3.897022	5.771177828	0.016291112	0.041571873
ENSRNOG00000000576	-0.266643392	3.67707015	5.770863844	0.016294023	0.041572601
ENSRNOG00000003880	-0.50093942	0.322164225	5.769894798	0.01630301	0.04158883
ENSRNOG00000001786	-0.170811295	5.47157574	5.769294565	0.01630858	0.041596336
ENSRNOG00000010160	-0.202494539	3.450802044	5.763278206	0.016364512	0.041725553
ENSRNOG00000011193	-0.1870118	4.920691001	5.760025014	0.016394838	0.041787236
ENSRNOG00000001426	-0.144888385	5.097513869	5.760263926	0.016392609	0.041787236
ENSRNOG00000001182	-0.262751902	5.171766738	5.759833977	0.016396621	0.041787236
ENSRNOG00000009548	-0.194428504	4.170309776	5.758923495	0.01640512	0.041795442
ENSRNOG00000007043	-0.206152304	3.804739654	5.752832323	0.016462095	0.041920361
ENSRNOG00000055298	-0.550961936	-0.319557739	5.750340512	0.016485461	0.041973113
ENSRNOG00000012315	-0.660466575	0.405956931	5.747943683	0.016507969	0.042023662
ENSRNOG00000028014	-0.170893295	5.895278479	5.746445403	0.016522055	0.042040608
ENSRNOG00000022837	-0.212889891	2.840323659	5.745377231	0.016532105	0.042058057
ENSRNOG00000005538	-0.148417841	6.370844873	5.740297084	0.016579988	0.042165473
ENSRNOG00000018770	-0.267091268	3.61731885	5.740075343	0.016582081	0.042165473
ENSRNOG00000002890	-0.460571855	3.926910075	5.739402688	0.016588433	0.042174253
ENSRNOG00000028941	-0.364084102	3.328036446	5.737604252	0.016605427	0.042203906
ENSRNOG00000013445	-0.216798621	4.100197903	5.733994922	0.016639588	0.042277156
ENSRNOG00000002044	-0.292277979	2.644340544	5.730477852	0.016672945	0.042344133
ENSRNOG00000014785	-0.156061138	5.619077592	5.730369716	0.016673972	0.042344133
ENSRNOG00000001072	-0.20110106	5.918891809	5.722094108	0.016752738	0.04251293
ENSRNOG00000051346	-0.274406456	3.570913481	5.721976109	0.016753864	0.04251293
ENSRNOG00000017568	-0.222975263	7.179487669	5.720555291	0.016767427	0.042540528
ENSRNOG00000061299	-0.677140622	4.906869277	5.717710124	0.01679462	0.042602693
ENSRNOG00000057754	-0.214874225	4.979286278	5.712486397	0.016844665	0.042695442
ENSRNOG00000000888	-0.15538447	6.14288454	5.712706278	0.016842555	0.042695442
ENSRNOG00000051257	-0.783922969	-1.024072646	5.709833888	0.016870136	0.042739476
ENSRNOG00000004239	-0.566632488	0.164639811	5.705640842	0.016910481	0.042827983
ENSRNOG00000016451	-0.364174916	1.302155581	5.699940538	0.016965488	0.042960424
ENSRNOG00000008480	-0.535625245	4.043086313	5.699409965	0.016970617	0.042966541
ENSRNOG00000057806	-0.162939356	5.947795771	5.677675263	0.017182119	0.043446451
ENSRNOG00000038786	-0.642981716	-1.132716291	5.676276656	0.017195822	0.043474157
ENSRNOG00000023216	-0.265707366	1.983472527	5.675692117	0.017201552	0.043481702
ENSRNOG00000009643	-0.217143065	6.717151538	5.674137067	0.017216806	0.043513314
ENSRNOG00000008081	-0.147753373	4.838186594	5.670452662	0.017253004	0.04358495
ENSRNOG00000028699	-0.197464608	3.676084025	5.670411493	0.017253408	0.04358495
ENSRNOG00000014630	-0.212238333	4.707844782	5.670082724	0.017256642	0.043586166
ENSRNOG00000010169	-0.203351004	6.180055904	5.668722529	0.017270028	0.043613019
ENSRNOG00000015140	-0.224166669	3.767593766	5.667634844	0.01728074	0.043633111
ENSRNOG00000002052	-0.249599997	3.57676554	5.655311245	0.017402586	0.043919755
ENSRNOG00000018236	-1.364236461	-0.848516913	5.65477344	0.017407923	0.04392438
ENSRNOG00000019147	-0.320694142	3.189312704	5.654547615	0.017410165	0.04392438
ENSRNOG00000048320	-0.370590665	5.783455056	5.65371691	0.017418414	0.043931693
ENSRNOG00000031769	-0.384566	2.262214384	5.645713666	0.017498093	0.044118599
ENSRNOG00000029212	-0.211806248	5.886020754	5.645363444	0.017501588	0.044120386
ENSRNOG00000032813	-0.288217058	3.9635364	5.641025529	0.017544942	0.044215599
ENSRNOG00000031162	-0.574392508	-1.025427368	5.635194959	0.017603388	0.044341718
ENSRNOG00000001681	-0.173488796	4.558219478	5.631113038	0.017644425	0.044438018
ENSRNOG00000019746	-0.167827593	5.823422679	5.624697897	0.017709118	0.044589179
ENSRNOG00000019630	-0.26346234	4.594612563	5.624219126	0.017713956	0.044591856
ENSRNOG00000020991	-0.394016945	0.851307245	5.623667813	0.017719528	0.044598794
ENSRNOG00000057318	-0.597242391	-0.905437344	5.618764692	0.017769169	0.044716628
ENSRNOG00000002757	-0.163955366	4.521621479	5.614829081	0.017809118	0.044810039
ENSRNOG00000017695	-0.479517188	0.081737335	5.609487247	0.017863489	0.044925428
ENSRNOG00000010891	-0.219759436	4.184547014	5.603149749	0.017928218	0.04506674
ENSRNOG00000038835	-0.516587544	0.680479087	5.603332419	0.017926348	0.04506674
ENSRNOG00000043370	-0.147495181	6.287461891	5.602670809	0.017933119	0.045071906
ENSRNOG00000054973	-0.50085275	-0.617260302	5.597781168	0.017983239	0.045176361
ENSRNOG00000014149	-0.412530164	4.196536826	5.595400872	0.01800769	0.045230609
ENSRNOG00000053086	-0.263069781	8.445515454	5.59425925	0.018019429	0.045252917
ENSRNOG00000010730	-0.648158696	0.662914453	5.590658355	0.018056508	0.045338845
ENSRNOG00000007926	-0.178034715	4.869908137	5.589774772	0.018065619	0.045354528
ENSRNOG00000008725	-0.146837759	5.70007299	5.587736474	0.018086654	0.045400139
ENSRNOG00000021680	-0.186706153	3.07522182	5.579217936	0.018174837	0.045607032
ENSRNOG00000002278	-0.414707178	3.436161929	5.578500388	0.018182285	0.045618492
ENSRNOG00000019163	-0.470780621	5.892394673	5.573816108	0.018230986	0.045718948
ENSRNOG00000003288	-0.262506165	4.523890646	5.571301999	0.01825718	0.045777386
ENSRNOG00000051069	-0.232183617	4.02804818	5.561982277	0.01835462	0.04599257
ENSRNOG00000037850	-0.230973542	4.495472247	5.562218614	0.018352142	0.04599257
ENSRNOG00000049686	-0.173368035	3.811160862	5.561471589	0.018359975	0.045998709

ENSRNOG00000012692	-0.192206257	4.107461224	5.559215193	0.018383654	0.046041509
ENSRNOG00000014060	-0.265941835	7.428046138	5.556628448	0.018410838	0.046089678
ENSRNOG00000056820	-0.471011097	-0.594094637	5.55606128	0.018416804	0.046097325
ENSRNOG00000018847	-0.135001638	5.069950709	5.545022482	0.018533319	0.046345004
ENSRNOG00000008841	-0.375599046	1.098578062	5.542740075	0.018557504	0.046398156
ENSRNOG00000014693	-0.149783592	6.013329285	5.542459157	0.018560483	0.046398278
ENSRNOG00000018011	-0.189473663	5.119645115	5.541786967	0.018567614	0.046408776
ENSRNOG00000008297	-0.190403714	4.232003873	5.539396638	0.018592992	0.046464873
ENSRNOG00000043069	-0.227545155	3.99774661	5.535914915	0.018630022	0.046550066
ENSRNOG00000042528	-0.712822953	-0.793670023	5.531889878	0.018672926	0.04663519
ENSRNOG00000005135	-0.274804725	2.663284747	5.52932066	0.018700365	0.04668899
ENSRNOG00000056722	-0.676390266	-0.869123268	5.528280863	0.018711481	0.04670938
ENSRNOG00000017298	-0.257855944	2.994164854	5.527609195	0.018718666	0.046719949
ENSRNOG00000009837	-0.289157493	4.467884377	5.526950869	0.018725711	0.046730166
ENSRNOG00000019840	-0.186099105	4.099394735	5.518595413	0.01881536	0.046946488
ENSRNOG00000050563	-0.298701334	7.323933647	5.516282081	0.018840259	0.047001208
ENSRNOG00000039050	-0.280043969	2.327428127	5.510673069	0.018900772	0.047122473
ENSRNOG00000011404	-0.178306815	4.583817124	5.509848814	0.018909681	0.047137263
ENSRNOG00000002855	-0.155732876	5.865069908	5.505558817	0.018956122	0.047223291
ENSRNOG00000011422	-0.208848927	5.480406538	5.501404151	0.01900121	0.047320724
ENSRNOG00000045779	-0.25161192	6.223757601	5.500795598	0.019007824	0.04732975
ENSRNOG00000031965	-0.154950346	5.506525884	5.497586313	0.019042741	0.047405332
ENSRNOG00000011861	-0.487778637	2.97706639	5.497455461	0.019044166	0.047405332
ENSRNOG00000027617	-0.183102996	3.717132937	5.494467341	0.019076738	0.047471488
ENSRNOG00000056599	-0.446935863	-0.18760084	5.49411999	0.019080528	0.047473458
ENSRNOG00000062009	-0.246930752	2.83646401	5.489377698	0.019132352	0.047587442
ENSRNOG00000000795	-0.261858722	3.535124489	5.485939035	0.01917002	0.047673645
ENSRNOG00000024553	-0.265654165	2.663703924	5.484992141	0.019180406	0.047691984
ENSRNOG00000014661	-0.311531122	4.028670162	5.484476636	0.019186063	0.047698559
ENSRNOG00000021088	-0.385200923	1.161595465	5.482016756	0.01921308	0.047737613
ENSRNOG00000006130	-0.136887973	8.020555022	5.479849557	0.019236915	0.047787465
ENSRNOG00000056643	-0.234876826	5.862683332	5.474333946	0.019297713	0.047908434
ENSRNOG00000006048	-0.204432611	3.499320266	5.469821946	0.019347597	0.048024744
ENSRNOG00000028236	-0.18574109	5.183369749	5.468952372	0.019357226	0.048041115
ENSRNOG00000005346	-0.183802575	4.656847051	5.465737063	0.019392873	0.048099428
ENSRNOG00000016591	-0.181209621	4.353345545	5.465119357	0.019399729	0.048108897
ENSRNOG00000009739	-0.146587602	5.797902776	5.46476854	0.019403624	0.048111021
ENSRNOG00000004359	-0.198384099	6.06442331	5.448352954	0.019586784	0.048519578
ENSRNOG00000021831	-0.361175391	1.404444603	5.447970126	0.019591077	0.048522621
ENSRNOG00000016239	-0.231334562	4.085446627	5.437668013	0.019706963	0.0487715
ENSRNOG00000017864	-0.201258895	5.444584704	5.436968444	0.019714858	0.048775993
ENSRNOG00000024863	-0.185009997	4.227872823	5.43696124	0.01971494	0.048775993
ENSRNOG00000016420	-0.341931361	0.764635136	5.433372571	0.019755491	0.04886105
ENSRNOG00000060946	-0.182469757	5.19899955	5.427276346	0.019824575	0.049001299
ENSRNOG00000025644	-0.224974846	4.266517659	5.420030979	0.019907007	0.049171776
ENSRNOG00000006925	-0.22556549	3.135049177	5.419665664	0.019911172	0.049171776
ENSRNOG00000030447	-0.562866547	-0.579557787	5.419909671	0.01990839	0.049171776
ENSRNOG00000024170	-0.165023684	4.220229372	5.405777213	0.02007021	0.049546571
ENSRNOG00000001294	-0.278567449	4.910265733	5.402913919	0.020103161	0.049612445
ENSRNOG00000023541	-0.238274401	6.320719859	5.398636323	0.020152492	0.049726438
ENSRNOG00000049615	-0.492028523	-0.449991208	5.396803167	0.020173671	0.049770941
ENSRNOG00000005178	-0.600652113	-0.915678237	5.396316003	0.020179303	0.04977708
ENSRNOG00000008234	-0.167394504	4.576844699	5.395798751	0.020185285	0.04978408
ENSRNOG00000000130	-0.434511183	6.581255646	5.39518453	0.02019239	0.049793849
ENSRNOG00000000157	-0.209959419	6.584646923	5.393536694	0.020211466	0.049817614
ENSRNOG00000017310	-0.179808094	3.827888231	5.393047685	0.020217131	0.04982382
ENSRNOG00000045765	-0.240199833	4.558070448	5.392749027	0.020220591	0.049824593
ENSRNOG00000027271	-0.204380876	4.80931217	5.392304133	0.020225747	0.049829543
ENSRNOG00000009239	-0.514429424	-0.243404794	5.389325551	0.0202603	0.049906906

Supplementary Table 9

Worksheet1: GSEA result for Gene Ontology (GO) terms

ONTOLOGY	ID	Description	setSize	enrichmentScore	NES	pvalue	p.adjust	qvalue	rank	leading_edge
BP	GO:0045165	cell fate commitment	175	0.5124	1.9418	1.163E-04	1.581E-02	1.494E-02	3363	tags=35%, list=21%, signal=28%
BP	GO:0048562	embryonic organ morphogenesis	227	0.4906	1.9058	1.123E-04	1.581E-02	1.494E-02	3758	tags=37%, list=24%, signal=29%
BP	GO:0010001	glial cell differentiation	271	0.4585	1.8083	1.098E-04	1.581E-02	1.494E-02	3774	tags=34%, list=24%, signal=27%
BP	GO:0051963	regulation of synapse assembly	188	0.4736	1.8069	1.153E-04	1.581E-02	1.494E-02	2822	tags=29%, list=18%, signal=24%
BP	GO:0021953	central nervous system neuron differentiation	201	0.4698	1.8049	1.139E-04	1.581E-02	1.494E-02	4234	tags=40%, list=27%, signal=30%
BP	GO:0030198	extracellular matrix organization	249	0.4559	1.7847	1.113E-04	1.581E-02	1.494E-02	3776	tags=37%, list=24%, signal=29%
BP	GO:0043062	extracellular structure organization	249	0.4559	1.7847	1.113E-04	1.581E-02	1.494E-02	3776	tags=37%, list=24%, signal=29%
BP	GO:0045229	external encapsulating structure organization	249	0.4559	1.7847	1.113E-04	1.581E-02	1.494E-02	3776	tags=37%, list=24%, signal=29%
BP	GO:0050808	synapse organization	609	0.4271	1.7595	1.020E-04	1.581E-02	1.494E-02	3748	tags=33%, list=24%, signal=26%
BP	GO:0050803	regulation of synapse structure or activity	370	0.4363	1.7546	1.062E-04	1.581E-02	1.494E-02	3748	tags=34%, list=24%, signal=26%
BP	GO:0050807	regulation of synapse organization	364	0.4363	1.7529	1.064E-04	1.581E-02	1.494E-02	3748	tags=33%, list=24%, signal=26%
BP	GO:0034330	cell junction organization	815	0.4202	1.7463	1.009E-04	1.581E-02	1.494E-02	3802	tags=33%, list=24%, signal=27%
BP	GO:0048598	embryonic morphogenesis	506	0.4272	1.7457	1.035E-04	1.581E-02	1.494E-02	3784	tags=32%, list=24%, signal=25%
BP	GO:0007416	synapse assembly	272	0.4425	1.7450	1.099E-04	1.581E-02	1.494E-02	2974	tags=29%, list=19%, signal=24%
BP	GO:0007411	axon guidance	205	0.4464	1.7181	1.137E-04	1.581E-02	1.494E-02	3714	tags=34%, list=23%, signal=26%
BP	GO:0097485	neuron projection guidance	205	0.4464	1.7181	1.137E-04	1.581E-02	1.494E-02	3714	tags=34%, list=23%, signal=26%
BP	GO:0099173	postsynapse organization	300	0.4305	1.7101	1.085E-04	1.581E-02	1.494E-02	3748	tags=35%, list=24%, signal=27%
BP	GO:0051216	cartilage development	169	0.4506	1.7020	1.168E-04	1.581E-02	1.494E-02	4189	tags=41%, list=26%, signal=31%
BP	GO:0007389	pattern specification process	328	0.4250	1.6983	1.074E-04	1.581E-02	1.494E-02	3962	tags=35%, list=25%, signal=27%
BP	GO:0048705	skeletal system morphogenesis	175	0.4480	1.6978	1.163E-04	1.581E-02	1.494E-02	4498	tags=41%, list=28%, signal=30%
BP	GO:1901888	regulation of cell junction assembly	272	0.4292	1.6925	1.099E-04	1.581E-02	1.494E-02	2925	tags=26%, list=18%, signal=22%
BP	GO:0061448	connective tissue development	251	0.4313	1.6897	1.110E-04	1.581E-02	1.494E-02	4216	tags=37%, list=27%, signal=28%
MF	GO:0001228	DNA-binding transcription activator activity, RNA polymerase II-specific	309	0.4244	1.6893	1.082E-04	1.581E-02	1.494E-02	3165	tags=28%, list=20%, signal=23%
MF	GO:0001216	DNA-binding transcription activator activity	314	0.4233	1.6874	1.079E-04	1.581E-02	1.494E-02	3165	tags=29%, list=20%, signal=23%
MF	GO:0001217	DNA-binding transcription repressor activity	190	0.4418	1.6872	1.150E-04	1.581E-02	1.494E-02	3513	tags=36%, list=22%, signal=29%
BP	GO:0048568	embryonic organ development	382	0.4182	1.6856	1.057E-04	1.581E-02	1.494E-02	4090	tags=36%, list=26%, signal=27%
BP	GO:0030902	hindbrain development	180	0.4432	1.6841	1.160E-04	1.581E-02	1.494E-02	3784	tags=35%, list=24%, signal=27%
MF	GO:0001227	DNA-binding transcription repressor activity, RNA polymerase II-specific	185	0.4419	1.6838	1.155E-04	1.581E-02	1.494E-02	3513	tags=36%, list=22%, signal=29%
BP	GO:0048469	cell maturation	182	0.4421	1.6822	1.158E-04	1.581E-02	1.494E-02	3734	tags=35%, list=24%, signal=27%
BP	GO:0048839	inner ear development	188	0.4401	1.6790	1.153E-04	1.581E-02	1.494E-02	3684	tags=34%, list=23%, signal=26%
BP	GO:0003002	regionalization	300	0.4211	1.6726	1.085E-04	1.581E-02	1.494E-02	3962	tags=36%, list=25%, signal=28%
BP	GO:0061138	morphogenesis of a branching epithelium	167	0.4412	1.6650	1.167E-04	1.581E-02	1.494E-02	4266	tags=45%, list=27%, signal=33%
BP	GO:0021700	developmental maturation	315	0.4176	1.6646	1.079E-04	1.581E-02	1.494E-02	3734	tags=34%, list=24%, signal=26%
BP	GO:0071695	anatomical structure maturation	237	0.4247	1.6556	1.116E-04	1.581E-02	1.494E-02	3672	tags=33%, list=23%, signal=26%
BP	GO:0001501	skeletal system development	410	0.4089	1.6552	1.051E-04	1.581E-02	1.494E-02	4232	tags=37%, list=27%, signal=28%
BP	GO:0043583	ear development	208	0.4283	1.6510	1.133E-04	1.581E-02	1.494E-02	3684	tags=32%, list=23%, signal=25%
BP	GO:0002064	epithelial cell development	196	0.4303	1.6496	1.143E-04	1.581E-02	1.494E-02	3807	tags=36%, list=24%, signal=27%
BP	GO:0001649	osteoblast differentiation	184	0.4324	1.6468	1.157E-04	1.581E-02	1.494E-02	3363	tags=35%, list=21%, signal=28%
BP	GO:0099175	regulation of postsynapse organization	187	0.4311	1.6446	1.153E-04	1.581E-02	1.494E-02	3748	tags=33%, list=24%, signal=26%
BP	GO:0045664	regulation of neuron differentiation	175	0.4334	1.6425	1.163E-04	1.581E-02	1.494E-02	3481	tags=35%, list=22%, signal=27%
BP	GO:0009887	animal organ morphogenesis	820	0.3949	1.6418	1.009E-04	1.581E-02	1.494E-02	4154	tags=35%, list=26%, signal=27%
BP	GO:0042063	gliogenesis	366	0.4078	1.6387	1.063E-04	1.581E-02	1.494E-02	3794	tags=32%, list=24%, signal=25%
BP	GO:0034329	cell junction assembly	464	0.4016	1.6355	1.041E-04	1.581E-02	1.494E-02	3883	tags=32%, list=25%, signal=25%
BP	GO:0002790	peptide secretion	266	0.4147	1.6333	1.101E-04	1.581E-02	1.494E-02	4828	tags=42%, list=30%, signal=30%
MF	GO:0004930	G protein-coupled receptor activity	251	0.4147	1.6248	1.110E-04	1.581E-02	1.494E-02	3797	tags=35%, list=24%, signal=27%
BP	GO:0030072	peptide hormone secretion	261	0.4134	1.6244	1.106E-04	1.581E-02	1.494E-02	4828	tags=43%, list=30%, signal=30%
BP	GO:0030900	forebrain development	459	0.3967	1.6144	1.043E-04	1.581E-02	1.494E-02	3894	tags=31%, list=25%, signal=24%
BP	GO:0016358	dendrite development	302	0.4063	1.6140	1.086E-04	1.581E-02	1.494E-02	3780	tags=33%, list=24%, signal=26%
MF	GO:0003700	DNA-binding transcription factor activity	735	0.3894	1.6137	1.013E-04	1.581E-02	1.494E-02	3177	tags=28%, list=20%, signal=23%
BP	GO:0015833	peptide transport	289	0.4063	1.6103	1.087E-04	1.581E-02	1.494E-02	4112	tags=35%, list=26%, signal=26%
BP	GO:0090596	sensory organ morphogenesis	239	0.4127	1.6093	1.117E-04	1.581E-02	1.494E-02	3752	tags=33%, list=24%, signal=26%
BP	GO:0021537	telencephalon development	326	0.3996	1.5959	1.076E-04	1.581E-02	1.494E-02	3873	tags=30%, list=24%, signal=23%
BP	GO:0007507	heart development	593	0.3855	1.5865	1.022E-04	1.581E-02	1.494E-02	4090	tags=33%, list=26%, signal=25%
MF	GO:0000981	DNA-binding transcription factor activity, RNA polymerase II-specific	695	0.3825	1.5826	1.015E-04	1.581E-02	1.494E-02	3177	tags=27%, list=20%, signal=23%
BP	GO:0007423	sensory organ development	540	0.3832	1.5719	1.028E-04	1.581E-02	1.494E-02	4090	tags=35%, list=26%, signal=27%
BP	GO:0048667	cell morphogenesis involved in neuron differentiation	592	0.3815	1.5695	1.022E-04	1.581E-02	1.494E-02	3758	tags=30%, list=24%, signal=24%
BP	GO:0051960	regulation of nervous system development	494	0.3843	1.5693	1.035E-04	1.581E-02	1.494E-02	3991	tags=36%, list=25%, signal=28%
MF	GO:0000978	RNA polymerase II cis-regulatory region sequence-specific DNA binding	653	0.3778	1.5607	1.016E-04	1.581E-02	1.494E-02	3177	tags=27%, list=20%, signal=23%
MF	GO:0000987	cis-regulatory region sequence-specific DNA binding	676	0.3769	1.5583	1.015E-04	1.581E-02	1.494E-02	3177	tags=27%, list=20%, signal=23%
BP	GO:0007417	central nervous system development	989	0.3734	1.5580	1.005E-04	1.581E-02	1.494E-02	3808	tags=30%, list=24%, signal=24%
BP	GO:0000122	negative regulation of transcription by RNA polymerase II	690	0.3751	1.5514	1.014E-04	1.581E-02	1.494E-02	3931	tags=34%, list=25%, signal=27%
BP	GO:0048812	neuron projection morphogenesis	649	0.3728	1.5396	1.016E-04	1.581E-02	1.494E-02	3758	tags=30%, list=24%, signal=24%
BP	GO:0030855	epithelial cell differentiation	533	0.3746	1.5354	1.030E-04	1.581E-02	1.494E-02	3807	tags=32%, list=24%, signal=25%
MF	GO:0038023	signaling receptor activity	647	0.3715	1.5342	1.016E-04	1.581E-02	1.494E-02	3144	tags=28%, list=20%, signal=23%
MF	GO:0060089	molecular transducer activity	647	0.3715	1.5342	1.016E-04	1.581E-02	1.494E-02	3144	tags=28%, list=20%, signal=23%
BP	GO:0120039	plasma membrane bounded cell projection morphogenesis	665	0.3705	1.5317	1.014E-04	1.581E-02	1.494E-02	3758	tags=30%, list=24%, signal=24%
BP	GO:0048858	cell projection morphogenesis	671	0.3702	1.5308	1.014E-04	1.581E-02	1.494E-02	3762	tags=30%, list=24%, signal=24%
BP	GO:0061564	axon development	498	0.3736	1.5258	1.036E-04	1.581E-02	1.494E-02	3762	tags=30%, list=24%, signal=24%
MF	GO:0004888	transmembrane signaling receptor activity	515	0.3728	1.5258	1.033E-04	1.581E-02	1.494E-02	3797	tags=32%, list=24%, signal=25%
BP	GO:0000902	cell morphogenesis	936	0.3655	1.5242	1.006E-04	1.581E-02	1.494E-02	3873	tags=30%, list=24%, signal=24%
BP	GO:0045596	negative regulation of cell differentiation	531	0.3719	1.5241	1.030E-04	1.581E-02	1.494E-02	3711	tags=32%, list=23%, signal=25%
BP	GO:0060429	epithelium development	980	0.3628	1.5138	1.005E-04	1.581E-02	1.494E-02	3810	tags=31%, list=24%, signal=25%
BP	GO:0007420	brain development	735	0.3631	1.5047	1.013E-04	1.581E-02	1.494E-02	3808	tags=29%, list=24%, signal=23%
BP	GO:0060322	head development	782	0.3621	1.5037	1.011E-04	1.581E-02	1.494E-02	3808	tags=28%, list=24%, signal=23%
BP	GO:0007167	enzyme-linked receptor protein signaling pathway	754	0.3612	1.4984	1.012E-04	1.581E-02	1.494E-02	3973	tags=32%, list=25%, signal=25%
BP	GO:0045892	negative regulation of DNA-templated transcription	937	0.3591	1.4975	1.006E-04	1.581E-02	1.494E-02	4218	tags=35%, list=27%, signal=27%
BP	GO:0023061	signal release	512	0.3660	1.4970	1.034E-04	1.581E-02	1.494E-02	4112	tags=33%, list=26%, signal=25%
BP	GO:1902679	negative regulation of RNA biosynthetic process	939	0.3583	1.4939	1.006E-04	1.581E-02	1.494E-02	4218	tags=35%, list=27%, signal=27%
BP	GO:0048729	tissue morphogenesis	520	0.3645	1.4922	1.033E-04	1.581E-02	1.494E-02	4234	tags=35%, list=27%, signal=26%
BP	GO:1903530	regulation of secretion by cell	566	0.3630	1.4912	1.025E-04	1.581E-02	1.494E-02	3520	tags=28%, list=22%, signal=23%
MF	GO:0000977	RNA polymerase II transcription regulatory region sequence-specific DNA binding	756	0.3555	1.4748	1.012E-04	1.581E-02	1.494E-02	3177	tags=26%, list=20%, signal=21%
BP	GO:0003013	circulatory system process	449	0.3627	1.4745	1.044E-04	1.581E-02	1.494E-02	3358	tags=27%, list=21%, signal=22%
BP	GO:0022603	regulation of anatomical structure morphogenesis	763	0.3547	1.4718	1.011E-04	1.581E-02	1.494E-02	4245	tags=35%, list=27%, signal=27%
BP	GO:0032940	secretion by cell	795	0.3540	1.4702	1.010E-04	1.581E-02	1.494E-02	4112	tags=32%, list=26%, signal=25%
BP	GO:0051046	regulation of secretion	600	0.3563	1.4671	1.021E-04	1.581E-02	1.494E-02	3419	tags=27%, list=22%, signal=22%
BP	GO:0048589	developmental growth	689	0.3530	1.4602	1.014E-04	1.581E-02	1.494E-02	4235	tags=34%, list=27%, signal=26%
BP	GO:0051093	negative regulation of developmental process	748	0.3511	1.4565	1.012E-04	1.581E-02	1.494E-02	3596	tags=30%, list=23%, signal=24%
BP	GO:0009790	embryo development	1000	0.3483	1.4540	1.005E-04	1.581E-02	1.494E-02	4097	tags=30%, list=26%, signal=24%

BP	GO:0140352	export from cell	853	0.3478	1.4476	1.008E-04	1.581E-02	1.494E-02	4137	tags=31%, list=26%, signal=25%
BP	GO:0046903	secretion	910	0.3467	1.4456	1.006E-04	1.581E-02	1.494E-02	4112	tags=31%, list=26%, signal=25%
MF	GO:0043565	sequence-specific DNA binding	967	0.3455	1.4411	1.006E-04	1.581E-02	1.494E-02	3177	tags=25%, list=20%, signal=21%
MF	GO:1990837	sequence-specific double-stranded DNA binding	883	0.3459	1.4410	1.007E-04	1.581E-02	1.494E-02	3177	tags=25%, list=20%, signal=21%
MF	GO:0000976	transcription cis-regulatory region binding	844	0.3462	1.4403	1.008E-04	1.581E-02	1.494E-02	3177	tags=25%, list=20%, signal=21%
MF	GO:0001067	transcription regulatory region nucleic acid binding	849	0.3455	1.4377	1.008E-04	1.581E-02	1.494E-02	3177	tags=25%, list=20%, signal=21%
BP	GO:0048646	anatomical structure formation involved in morphogenesis	982	0.3418	1.4265	1.005E-04	1.581E-02	1.494E-02	4090	tags=32%, list=26%, signal=25%
BP	GO:0035295	tube development	975	0.3395	1.4168	1.005E-04	1.581E-02	1.494E-02	4223	tags=33%, list=27%, signal=26%
BP	GO:0050877	nervous system process	891	0.3398	1.4161	1.006E-04	1.581E-02	1.494E-02	4205	tags=33%, list=27%, signal=26%
BP	GO:0040007	growth	875	0.3331	1.3872	1.007E-04	1.581E-02	1.494E-02	4235	tags=32%, list=27%, signal=25%
CC	GO:0045211	postsynaptic membrane	355	0.4005	1.6063	1.069E-04	1.581E-02	1.494E-02	3897	tags=34%, list=25%, signal=26%
CC	GO:0097060	synaptic membrane	511	0.3889	1.5908	1.034E-04	1.581E-02	1.494E-02	3943	tags=33%, list=25%, signal=26%
CC	GO:0030425	dendrite	735	0.3595	1.4898	1.013E-04	1.581E-02	1.494E-02	3956	tags=32%, list=25%, signal=25%
CC	GO:0097447	dendritic tree	737	0.3576	1.4826	1.012E-04	1.581E-02	1.494E-02	3956	tags=32%, list=25%, signal=25%
CC	GO:0098978	glutamatergic synapse	825	0.3476	1.4452	1.009E-04	1.581E-02	1.494E-02	3883	tags=29%, list=25%, signal=23%
CC	GO:0070161	anchoring junction	584	0.3505	1.4414	1.023E-04	1.581E-02	1.494E-02	4220	tags=31%, list=27%, signal=23%
CC	GO:0098794	postsynapse	932	0.3412	1.4231	1.006E-04	1.581E-02	1.494E-02	3943	tags=30%, list=25%, signal=24%
BP	GO:0021782	glial cell development	136	0.5600	2.0670	1.203E-04	1.588E-02	1.501E-02	3762	tags=43%, list=24%, signal=33%
BP	GO:0007219	Notch signaling pathway	143	0.5059	1.8743	1.201E-04	1.588E-02	1.501E-02	3945	tags=46%, list=25%, signal=35%
BP	GO:0071772	response to BMP	126	0.4905	1.7928	1.218E-04	1.588E-02	1.501E-02	2708	tags=28%, list=17%, signal=23%
BP	GO:0071773	cellular response to BMP stimulus	126	0.4905	1.7928	1.218E-04	1.588E-02	1.501E-02	2708	tags=28%, list=17%, signal=23%
BP	GO:0099068	postsynapse assembly	100	0.5443	1.9334	1.262E-04	1.631E-02	1.542E-02	2764	tags=37%, list=17%, signal=31%
BP	GO:0008593	regulation of Notch signaling pathway	78	0.5825	1.9990	1.307E-04	1.674E-02	1.582E-02	3945	tags=53%, list=25%, signal=40%
BP	GO:1902893	regulation of miRNA transcription	64	0.5806	1.9327	1.342E-04	1.704E-02	1.610E-02	3758	tags=50%, list=24%, signal=38%
BP	GO:0048663	neuron fate commitment	53	0.6842	2.2040	1.381E-04	1.713E-02	1.619E-02	2987	tags=57%, list=19%, signal=46%
BP	GO:2000630	positive regulation of miRNA metabolic process	52	0.6087	1.9561	1.382E-04	1.713E-02	1.619E-02	3758	tags=52%, list=24%, signal=40%
BP	GO:0001708	cell fate specification	50	0.6025	1.9233	1.385E-04	1.713E-02	1.619E-02	3363	tags=50%, list=21%, signal=40%
BP	GO:1902895	positive regulation of miRNA transcription	46	0.5937	1.8630	1.404E-04	1.722E-02	1.627E-02	3758	tags=50%, list=24%, signal=38%
MF	GO:0005201	extracellular matrix structural constituent	42	0.6383	1.9703	1.430E-04	1.738E-02	1.643E-02	3046	tags=55%, list=19%, signal=44%
MF	GO:0030020	extracellular matrix structural constituent conferring tensile strength	27	0.7226	2.0450	1.501E-04	1.809E-02	1.710E-02	3046	tags=70%, list=19%, signal=57%
BP	GO:0002125	maternal aggressive behavior	4	0.9628	1.7099	1.836E-04	2.194E-02	2.073E-02	30	tags=50%, list=0%, signal=50%
MF	GO:0031894	V1A vasopressin receptor binding	3	0.9932	1.6421	1.860E-04	2.204E-02	2.083E-02	30	tags=67%, list=0%, signal=67%
BP	GO:0071692	protein localization to extracellular region	348	0.4040	1.6188	2.140E-04	2.323E-02	2.196E-02	4459	tags=38%, list=28%, signal=28%
BP	GO:0035592	establishment of protein localization to extracellular region	346	0.4012	1.6071	2.140E-04	2.323E-02	2.196E-02	4459	tags=37%, list=28%, signal=27%
BP	GO:0009306	protein secretion	343	0.3996	1.6005	2.140E-04	2.323E-02	2.196E-02	4432	tags=37%, list=28%, signal=27%
BP	GO:0001503	ossification	351	0.3870	1.5518	2.137E-04	2.323E-02	2.196E-02	3380	tags=29%, list=21%, signal=23%
BP	GO:0007409	axonogenesis	435	0.3705	1.5043	2.093E-04	2.323E-02	2.196E-02	3714	tags=30%, list=23%, signal=24%
BP	GO:0007169	cell surface receptor protein tyrosine kinase signaling pathway	503	0.3597	1.4694	2.071E-04	2.323E-02	2.196E-02	4283	tags=35%, list=27%, signal=26%
BP	GO:0071363	cellular response to growth factor stimulus	667	0.3451	1.4269	2.028E-04	2.323E-02	2.196E-02	3977	tags=31%, list=25%, signal=24%
BP	GO:0007610	behavior	674	0.3440	1.4223	2.029E-04	2.323E-02	2.196E-02	3808	tags=28%, list=24%, signal=22%
BP	GO:0050804	modulation of chemical synaptic transmission	565	0.3456	1.4196	2.050E-04	2.323E-02	2.196E-02	4238	tags=34%, list=27%, signal=26%
BP	GO:0099177	regulation of trans-synaptic signaling	565	0.3456	1.4196	2.050E-04	2.323E-02	2.196E-02	4238	tags=34%, list=27%, signal=26%
CC	GO:0005911	cell-cell junction	428	0.3714	1.5068	2.098E-04	2.323E-02	2.196E-02	3830	tags=29%, list=24%, signal=23%
CC	GO:0031012	extracellular matrix	291	0.3943	1.5625	2.175E-04	2.326E-02	2.198E-02	3724	tags=38%, list=24%, signal=30%
CC	GO:0030312	external encapsulating structure	292	0.3883	1.5396	2.174E-04	2.326E-02	2.198E-02	3724	tags=38%, list=24%, signal=30%
BP	GO:0002791	regulation of peptide secretion	221	0.4190	1.6228	2.257E-04	2.337E-02	2.209E-02	4654	tags=42%, list=29%, signal=30%
BP	GO:0030073	insulin secretion	214	0.4195	1.6198	2.268E-04	2.337E-02	2.209E-02	4112	tags=36%, list=26%, signal=27%
BP	GO:0090087	regulation of peptide transport	223	0.4166	1.6144	2.256E-04	2.337E-02	2.209E-02	4654	tags=42%, list=29%, signal=30%
BP	GO:0090276	regulation of peptide hormone secretion	218	0.4162	1.6093	2.263E-04	2.337E-02	2.209E-02	4654	tags=42%, list=29%, signal=30%
BP	GO:0003007	heart morphogenesis	224	0.4141	1.6053	2.257E-04	2.337E-02	2.209E-02	3985	tags=36%, list=25%, signal=27%
BP	GO:0007188	adenylate cyclase-modulating G protein-coupled receptor signaling pathway	180	0.4238	1.6104	2.321E-04	2.357E-02	2.228E-02	3877	tags=37%, list=24%, signal=28%
BP	GO:0050796	regulation of insulin secretion	183	0.4221	1.6065	2.316E-04	2.357E-02	2.228E-02	4432	tags=40%, list=28%, signal=29%
BP	GO:0051961	negative regulation of nervous system development	151	0.4553	1.6996	2.374E-04	2.394E-02	2.262E-02	3711	tags=38%, list=23%, signal=30%
BP	GO:0031644	regulation of nervous system process	144	0.4691	1.7399	2.395E-04	2.399E-02	2.267E-02	4199	tags=44%, list=27%, signal=32%
BP	GO:0048709	oligodendrocyte differentiation	132	0.4699	1.7270	2.421E-04	2.408E-02	2.275E-02	3711	tags=35%, list=23%, signal=27%
BP	GO:0030509	BMP signaling pathway	116	0.4825	1.7477	2.462E-04	2.414E-02	2.282E-02	2708	tags=27%, list=17%, signal=22%
BP	GO:0072073	kidney epithelium development	121	0.4804	1.7473	2.448E-04	2.414E-02	2.282E-02	3423	tags=38%, list=22%, signal=30%
MF	GO:0046933	proton-transporting ATP synthase activity, rotational mechanism	13	-0.7878	-2.0878	2.534E-04	2.451E-02	2.317E-02	2095	tags=69%, list=13%, signal=60%
CC	GO:0045259	proton-transporting ATP synthase complex	13	-0.8112	-2.1499	2.534E-04	2.451E-02	2.317E-02	2095	tags=77%, list=13%, signal=67%
BP	GO:2000628	regulation of miRNA metabolic process	76	0.5635	1.9270	2.617E-04	2.481E-02	2.344E-02	3758	tags=49%, list=24%, signal=37%
BP	GO:0030510	regulation of BMP signaling pathway	77	0.5367	1.8378	2.617E-04	2.481E-02	2.344E-02	3340	tags=36%, list=21%, signal=29%
CC	GO:0045277	respiratory chain complex IV	15	-0.7795	-2.1560	2.602E-04	2.481E-02	2.344E-02	1098	tags=67%, list=7%, signal=62%
MF	GO:0008137	NADH dehydrogenase (ubiquinone) activity	17	-0.7703	-2.1987	2.703E-04	2.501E-02	2.363E-02	782	tags=53%, list=5%, signal=50%
MF	GO:0015252	proton channel activity	17	-0.7074	-2.0193	2.703E-04	2.501E-02	2.363E-02	2095	tags=53%, list=13%, signal=46%
BP	GO:0061614	miRNA transcription	66	0.5683	1.9008	2.674E-04	2.501E-02	2.363E-02	3758	tags=48%, list=24%, signal=37%
BP	GO:0014003	oligodendrocyte development	59	0.5554	1.8273	2.709E-04	2.501E-02	2.363E-02	4103	tags=44%, list=26%, signal=33%
BP	GO:0014014	negative regulation of gliogenesis	48	0.6000	1.8978	2.795E-04	2.564E-02	2.423E-02	3596	tags=50%, list=23%, signal=39%
MF	GO:0003954	NADH dehydrogenase activity	21	-0.7345	-2.2154	2.830E-04	2.579E-02	2.437E-02	782	tags=43%, list=5%, signal=41%
CC	GO:0098556	cytoplasmic side of rough endoplasmic reticulum membrane	23	-0.7659	-2.3783	2.867E-04	2.597E-02	2.454E-02	1506	tags=65%, list=10%, signal=59%
MF	GO:0015453	oxidoreduction-driven active transmembrane transporter activity	31	-0.7953	-2.6626	3.082E-04	2.607E-02	2.464E-02	782	tags=48%, list=5%, signal=46%
MF	GO:0016655	oxidoreductase activity, acting on NAD(P)H, quinone or similar compound as acceptor	28	-0.7326	-2.3879	3.021E-04	2.607E-02	2.464E-02	782	tags=36%, list=5%, signal=34%
MF	GO:0018455	alcohol dehydrogenase [NAD(P)+] activity	32	-0.6098	-2.0593	3.131E-04	2.607E-02	2.464E-02	2844	tags=50%, list=18%, signal=41%
MF	GO:0004033	aldo-keto reductase (NADPH) activity	25	-0.6381	-2.0205	2.933E-04	2.607E-02	2.464E-02	2651	tags=52%, list=17%, signal=43%
BP	GO:0045686	negative regulation of glial cell differentiation	31	0.6666	1.9390	2.960E-04	2.607E-02	2.464E-02	3473	tags=58%, list=22%, signal=45%
BP	GO:0002009	morphogenesis of an epithelium	439	0.3595	1.4600	3.135E-04	2.607E-02	2.464E-02	4223	tags=35%, list=27%, signal=26%
BP	GO:0060284	regulation of cell development	729	0.3386	1.4032	3.036E-04	2.607E-02	2.464E-02	3991	tags=33%, list=25%, signal=26%
MF	GO:0003690	double-stranded DNA binding	946	0.3250	1.3556	3.017E-04	2.607E-02	2.464E-02	3177	tags=24%, list=20%, signal=20%
BP	GO:0044087	regulation of cellular component biogenesis	939	0.3207	1.3370	3.018E-04	2.607E-02	2.464E-02	4061	tags=29%, list=26%, signal=23%
CC	GO:0070069	cytochrome complex	26	-0.7366	-2.3533	2.964E-04	2.607E-02	2.464E-02	488	tags=54%, list=3%, signal=52%
CC	GO:0046540	U4/U6 x U5 tri-snRNP complex	31	-0.6171	-2.0658	3.082E-04	2.607E-02	2.464E-02	2512	tags=48%, list=16%, signal=41%
CC	GO:0097526	spliceosomal tri-snRNP complex	32	-0.6075	-2.0515	3.131E-04	2.607E-02	2.464E-02	1736	tags=47%, list=11%, signal=42%
CC	GO:0098576	lumenal side of membrane	21	0.7251	1.9506	3.092E-04	2.607E-02	2.464E-02	922	tags=29%, list=6%, signal=27%
CC	GO:0030424	axon	757	0.3352	1.3906	3.036E-04	2.607E-02	2.464E-02	4086	tags=31%, list=26%, signal=24%
BP	GO:0050767	regulation of neurogenesis	404	0.3672	1.4846	3.159E-04	2.611E-02	2.468E-02	4282	tags=38%, list=27%, signal=29%
CC	GO:0016469	proton-transporting two-sector ATPase complex	34	-0.6607	-2.2596	3.186E-04	2.618E-02	2.475E-02	1580	tags=50%, list=10%, signal=45%
BP	GO:0042886	amide transport	317	0.3975	1.5846	3.240E-04	2.633E-02	2.488E-02	4112	tags=34%, list=26%, signal=26%
BP	GO:0090287	regulation of cellular response to growth factor stimulus	248	0.4040	1.5817	3.333E-04	2.633E-02	2.488E-02	3628	tags=33%, list=23%, signal=26%
BP	GO:0060562	epithelial tube morphogenesis	302	0.3873	1.5386	3.257E-04	2.633E-02	2.488E-02	4223	tags=37%, list=27%, signal=28%
CC	GO:0030867	rough endoplasmic reticulum membrane	42	-0.6568	-2.3637	3.321E-04	2.633E-02	2.488E-02	1582	tags=43%, list=10%, signal=39%
CC	GO:0030964	NADH dehydrogenase complex	42	-0.6553	-2.3585	3.321E-04	2.633E-02	2.488E-02	1072	tags=36%, list=7%, signal=33%

CC	GO:0045271	respiratory chain complex I	42	-0.6553	-2.3585	3.321E-04	2.633E-02	2.488E-02	1072	tags=36%, list=7%, signal=33%
CC	GO:0098553	lumenal side of endoplasmic reticulum membrane	12	0.8495	1.9958	3.333E-04	2.633E-02	2.488E-02	922	tags=42%, list=6%, signal=39%
CC	GO:0022627	cytosolic small ribosomal subunit	43	-0.6094	-2.2007	3.357E-04	2.637E-02	2.492E-02	1436	tags=44%, list=9%, signal=40%
CC	GO:0071005	U2-type precatalytic spliceosome	44	-0.5506	-1.9950	3.423E-04	2.675E-02	2.528E-02	2936	tags=50%, list=19%, signal=41%
MF	GO:0009055	electron transfer activity	47	-0.6876	-2.5278	3.512E-04	2.714E-02	2.565E-02	782	tags=34%, list=5%, signal=32%
CC	GO:0071011	precatalytic spliceosome	47	-0.5485	-2.0164	3.512E-04	2.714E-02	2.565E-02	2936	tags=49%, list=19%, signal=40%
CC	GO:0022625	cytosolic large ribosomal subunit	51	-0.5654	-2.1172	3.578E-04	2.750E-02	2.599E-02	1534	tags=41%, list=10%, signal=37%
BP	GO:0019646	aerobic electron transport chain	57	-0.5979	-2.2812	3.785E-04	2.840E-02	2.684E-02	1214	tags=30%, list=8%, signal=28%
BP	GO:0042773	ATP synthesis coupled electron transport	68	-0.5706	-2.2556	4.014E-04	2.840E-02	2.684E-02	1214	tags=28%, list=8%, signal=26%
BP	GO:0042775	mitochondrial ATP synthesis coupled electron transport	66	-0.5458	-2.1467	3.964E-04	2.840E-02	2.684E-02	1214	tags=36%, list=8%, signal=34%
BP	GO:0010257	NADH dehydrogenase complex assembly	56	-0.5420	-2.0649	3.747E-04	2.840E-02	2.684E-02	2268	tags=41%, list=14%, signal=35%
BP	GO:0032981	mitochondrial respiratory chain complex I assembly	56	-0.5420	-2.0649	3.747E-04	2.840E-02	2.684E-02	2268	tags=41%, list=14%, signal=35%
MF	GO:0019843	rRNA binding	67	-0.5063	-1.9938	4.006E-04	2.840E-02	2.684E-02	1322	tags=27%, list=8%, signal=25%
BP	GO:0045665	negative regulation of neuron differentiation	71	0.5431	1.8385	3.966E-04	2.840E-02	2.684E-02	3481	tags=44%, list=22%, signal=34%
BP	GO:0042472	inner ear morphogenesis	87	0.5259	1.8322	3.844E-04	2.840E-02	2.684E-02	3102	tags=36%, list=20%, signal=29%
BP	GO:0048708	astrocyte differentiation	86	0.5230	1.8200	3.853E-04	2.840E-02	2.684E-02	3734	tags=41%, list=24%, signal=31%
BP	GO:0042471	ear morphogenesis	102	0.4961	1.7669	3.777E-04	2.840E-02	2.684E-02	3635	tags=36%, list=23%, signal=28%
BP	GO:0099084	postsynaptic specialization organization	75	0.5178	1.7668	3.932E-04	2.840E-02	2.684E-02	3147	tags=39%, list=20%, signal=31%
MF	GO:0019900	kinase binding	743	0.3360	1.3934	4.048E-04	2.840E-02	2.684E-02	4486	tags=35%, list=28%, signal=26%
BP	GO:0070848	response to growth factor	697	0.3360	1.3908	4.054E-04	2.840E-02	2.684E-02	3977	tags=30%, list=25%, signal=24%
BP	GO:0035239	tube morphogenesis	752	0.3323	1.3786	4.046E-04	2.840E-02	2.684E-02	4270	tags=34%, list=27%, signal=26%
BP	GO:0099536	synaptic signaling	778	0.3310	1.3742	4.043E-04	2.840E-02	2.684E-02	4205	tags=33%, list=27%, signal=25%
BP	GO:0099537	trans-synaptic signaling	750	0.3286	1.3630	4.047E-04	2.840E-02	2.684E-02	4205	tags=33%, list=27%, signal=25%
CC	GO:0000313	organellar ribosome	70	-0.4882	-1.9438	4.045E-04	2.840E-02	2.684E-02	3182	tags=53%, list=20%, signal=42%
CC	GO:0005761	mitochondrial ribosome	70	-0.4882	-1.9438	4.045E-04	2.840E-02	2.684E-02	3182	tags=53%, list=20%, signal=42%
CC	GO:0015935	small ribosomal subunit	71	-0.5991	-2.3899	4.103E-04	2.860E-02	2.703E-02	1436	tags=38%, list=9%, signal=35%
CC	GO:0005791	rough endoplasmic reticulum	73	-0.5276	-2.1148	4.134E-04	2.867E-02	2.710E-02	1608	tags=30%, list=10%, signal=27%
MF	GO:0005179	hormone activity	48	0.5763	1.8228	4.192E-04	2.894E-02	2.735E-02	2158	tags=42%, list=14%, signal=36%
MF	GO:0022853	active monoatomic ion transmembrane transporter activity	78	-0.4807	-1.9518	4.255E-04	2.920E-02	2.760E-02	1364	tags=26%, list=9%, signal=24%
BP	GO:0045777	positive regulation of blood pressure	43	0.5843	1.8124	4.272E-04	2.920E-02	2.760E-02	2184	tags=33%, list=14%, signal=28%
BP	GO:0050708	regulation of protein secretion	265	0.3940	1.5510	4.408E-04	2.978E-02	2.815E-02	4432	tags=38%, list=28%, signal=28%
BP	GO:0007517	muscle organ development	278	0.3909	1.5441	4.383E-04	2.978E-02	2.815E-02	3927	tags=31%, list=25%, signal=23%
CC	GO:0098803	respiratory chain complex	82	-0.6956	-2.8443	4.427E-04	2.978E-02	2.815E-02	1185	tags=41%, list=7%, signal=39%
CC	GO:0055029	nuclear DNA-directed RNA polymerase complex	83	-0.4841	-1.9877	4.440E-04	2.978E-02	2.815E-02	4938	tags=58%, list=31%, signal=40%
BP	GO:0022904	respiratory electron transport chain	84	-0.5251	-2.1591	4.496E-04	2.989E-02	2.825E-02	1214	tags=25%, list=8%, signal=23%
CC	GO:0005684	U2-type spliceosomal complex	88	-0.4913	-2.0359	4.562E-04	2.989E-02	2.825E-02	4149	tags=52%, list=26%, signal=39%
CC	GO:0071013	catalytic step 2 spliceosome	88	-0.4871	-2.0186	4.562E-04	2.989E-02	2.825E-02	4744	tags=58%, list=30%, signal=41%
CC	GO:0000428	DNA-directed RNA polymerase complex	86	-0.4476	-1.8498	4.513E-04	2.989E-02	2.825E-02	3476	tags=42%, list=22%, signal=33%
CC	GO:0030880	RNA polymerase complex	88	-0.4333	-1.7958	4.562E-04	2.989E-02	2.825E-02	3476	tags=41%, list=22%, signal=32%
MF	GO:0004129	cytochrome-c oxidase activity	6	-0.9124	-1.8965	4.639E-04	2.998E-02	2.834E-02	488	tags=67%, list=3%, signal=65%
BP	GO:0048813	dendrite morphogenesis	184	0.4152	1.5813	4.627E-04	2.998E-02	2.834E-02	3748	tags=34%, list=24%, signal=26%
MF	GO:0051015	actin filament binding	183	0.4151	1.5798	4.632E-04	2.998E-02	2.834E-02	3507	tags=30%, list=22%, signal=23%
BP	GO:0022900	electron transport chain	93	-0.5481	-2.2907	4.695E-04	3.007E-02	2.842E-02	1214	tags=27%, list=8%, signal=25%
BP	GO:0033108	mitochondrial respiratory chain complex assembly	93	-0.4427	-1.8500	4.695E-04	3.007E-02	2.842E-02	3457	tags=40%, list=22%, signal=31%
CC	GO:0015934	large ribosomal subunit	100	-0.4908	-2.0840	4.810E-04	3.067E-02	2.898E-02	1937	tags=34%, list=12%, signal=30%
BP	GO:0007204	positive regulation of cytosolic calcium ion concentration	132	0.4607	1.6931	4.843E-04	3.074E-02	2.905E-02	4115	tags=40%, list=26%, signal=30%
BP	GO:0006119	oxidative phosphorylation	112	-0.5322	-2.2908	5.139E-04	3.218E-02	3.041E-02	1214	tags=26%, list=8%, signal=24%
MF	GO:0015078	proton transmembrane transporter activity	111	-0.4945	-2.1246	5.120E-04	3.218E-02	3.041E-02	1364	tags=26%, list=9%, signal=24%
BP	GO:0044057	regulation of system process	529	0.3495	1.4326	5.148E-04	3.218E-02	3.041E-02	3402	tags=27%, list=21%, signal=22%
CC	GO:0022626	cytosolic ribosome	113	-0.5454	-2.3484	5.181E-04	3.218E-02	3.041E-02	1534	tags=38%, list=10%, signal=35%
CC	GO:0098984	neuron to neuron synapse	499	0.3436	1.4032	5.182E-04	3.218E-02	3.041E-02	3924	tags=30%, list=25%, signal=23%
BP	GO:0048880	sensory system development	380	0.3668	1.4776	5.294E-04	3.267E-02	3.087E-02	4090	tags=35%, list=26%, signal=27%
BP	GO:0150063	visual system development	375	0.3658	1.4721	5.307E-04	3.267E-02	3.087E-02	4090	tags=35%, list=26%, signal=26%
BP	GO:0009914	hormone transport	328	0.3743	1.4956	5.369E-04	3.291E-02	3.110E-02	4432	tags=36%, list=28%, signal=27%
CC	GO:1990204	oxidoreductase complex	120	-0.5264	-2.2902	5.420E-04	3.297E-02	3.116E-02	1098	tags=23%, list=7%, signal=22%
CC	GO:0043235	receptor complex	306	0.3838	1.5258	5.427E-04	3.297E-02	3.116E-02	4132	tags=35%, list=26%, signal=27%
BP	GO:1902600	proton transmembrane transport	124	-0.4513	-1.9697	5.562E-04	3.365E-02	3.180E-02	1364	tags=23%, list=9%, signal=22%
CC	GO:0031332	RNAi effector complex	22	-0.6549	-2.0072	5.647E-04	3.402E-02	3.215E-02	1221	tags=32%, list=8%, signal=29%
BP	GO:0001763	morphogenesis of a branching structure	185	0.4131	1.5741	5.774E-04	3.464E-02	3.274E-02	4266	tags=43%, list=27%, signal=32%
CC	GO:0098798	mitochondrial protein-containing complex	135	-0.3643	-1.6165	5.882E-04	3.514E-02	3.321E-02	3201	tags=44%, list=20%, signal=36%
BP	GO:0006120	mitochondrial electron transport, NADH to ubiquinone	28	-0.6019	-1.9618	6.042E-04	3.595E-02	3.397E-02	1214	tags=29%, list=8%, signal=26%
BP	GO:0007189	adenylate cyclase-activating G protein-coupled receptor signaling pathway	120	0.4672	1.6975	6.130E-04	3.619E-02	3.420E-02	3848	tags=38%, list=24%, signal=29%
CC	GO:0071007	U2-type catalytic step 2 spliceosome	29	-0.5890	-1.9350	6.133E-04	3.619E-02	3.420E-02	4744	tags=72%, list=30%, signal=51%
BP	GO:0000460	maturation of 5.8S rRNA	31	-0.5726	-1.9168	6.163E-04	3.621E-02	3.422E-02	3140	tags=48%, list=20%, signal=39%
MF	GO:0003735	structural constituent of ribosome	152	-0.5288	-2.3835	6.394E-04	3.659E-02	3.458E-02	1603	tags=39%, list=10%, signal=35%
BP	GO:0009060	aerobic respiration	152	-0.4767	-2.1487	6.394E-04	3.659E-02	3.458E-02	1214	tags=21%, list=8%, signal=20%
MF	GO:0016251	RNA polymerase II general transcription initiation factor activity	33	-0.5722	-1.9434	6.333E-04	3.659E-02	3.458E-02	1618	tags=24%, list=10%, signal=22%
BP	GO:1903532	positive regulation of secretion by cell	305	0.3809	1.5144	6.510E-04	3.659E-02	3.458E-02	3520	tags=29%, list=22%, signal=23%
BP	GO:0046879	hormone secretion	320	0.3778	1.5071	6.466E-04	3.659E-02	3.458E-02	4432	tags=37%, list=28%, signal=27%
BP	GO:0043010	camera-type eye development	332	0.3750	1.4990	6.438E-04	3.659E-02	3.458E-02	4090	tags=35%, list=26%, signal=26%
BP	GO:0051047	positive regulation of secretion	322	0.3733	1.4895	6.468E-04	3.659E-02	3.458E-02	3520	tags=29%, list=22%, signal=23%
BP	GO:0001654	eye development	372	0.3681	1.4811	6.367E-04	3.659E-02	3.458E-02	4090	tags=35%, list=26%, signal=26%
BP	GO:0014706	striated muscle tissue development	374	0.3648	1.4682	6.367E-04	3.659E-02	3.458E-02	3908	tags=30%, list=25%, signal=23%
CC	GO:0098554	cytoplasmic side of endoplasmic reticulum membrane	35	-0.5791	-1.9869	6.485E-04	3.659E-02	3.458E-02	1506	tags=43%, list=10%, signal=39%
CC	GO:0005669	transcription factor TFIID complex	32	-0.5659	-1.9109	6.262E-04	3.659E-02	3.458E-02	3792	tags=44%, list=24%, signal=33%
MF	GO:0030971	receptor tyrosine kinase binding	65	0.5300	1.7694	6.685E-04	3.742E-02	3.537E-02	2852	tags=38%, list=18%, signal=32%
BP	GO:0006364	rRNA processing	174	-0.3855	-1.7697	7.062E-04	3.830E-02	3.619E-02	2993	tags=35%, list=19%, signal=29%
BP	GO:0008217	regulation of blood pressure	168	0.4226	1.5960	6.995E-04	3.830E-02	3.619E-02	3856	tags=36%, list=24%, signal=27%
BP	GO:0021915	neural tube development	163	0.4231	1.5930	7.017E-04	3.830E-02	3.619E-02	4956	tags=44%, list=31%, signal=30%
BP	GO:0001764	neuron migration	181	0.4128	1.5703	6.941E-04	3.830E-02	3.619E-02	4013	tags=34%, list=25%, signal=25%
BP	GO:0045597	positive regulation of cell differentiation	769	0.3263	1.3546	7.074E-04	3.830E-02	3.619E-02	3991	tags=31%, list=25%, signal=25%
BP	GO:0007268	chemical synaptic transmission	743	0.3253	1.3490	7.084E-04	3.830E-02	3.619E-02	4205	tags=33%, list=27%, signal=25%
BP	GO:0098916	anterograde trans-synaptic signaling	743	0.3253	1.3490	7.084E-04	3.830E-02	3.619E-02	4205	tags=33%, list=27%, signal=25%
CC	GO:0044391	ribosomal subunit	166	-0.5289	-2.4113	6.916E-04	3.830E-02	3.619E-02	1856	tags=38%, list=12%, signal=34%
CC	GO:0099081	supramolecular polymer	760	0.3236	1.3426	7.080E-04	3.830E-02	3.619E-02	3606	tags=29%, list=23%, signal=24%
BP	GO:0045747	positive regulation of Notch signaling pathway	36	0.6130	1.8393	7.205E-04	3.866E-02	3.653E-02	3132	tags=47%, list=20%, signal=38%
BP	GO:0050768	negative regulation of neurogenesis	143	0.4429	1.6409	7.204E-04	3.866E-02	3.653E-02	3711	tags=37%, list=23%, signal=29%
BP	GO:0010975	regulation of neuron projection development	509	0.3412	1.3948	7.245E-04	3.873E-02	3.660E-02	3991	tags=31%, list=25%, signal=24%
CC	GO:0005681	spliceosomal complex	181	-0.4556	-2.0999	7.364E-04	3.922E-02	3.706E-02	4165	tags=51%, list=26%, signal=38%
MF	GO:0070990	snRNP binding	13	-0.7415	-1.9652	7.603E-04	4.034E-02	3.812E-02	1736	tags=62%, list=11%, signal=55%

BP	GO:0045333	cellular respiration	191	-0.4239	-1.9724	7.746E-04	4.094E-02	3.869E-02	1214	tags=18%, list=8%, signal=17%
BP	GO:0060746	parental behavior	17	0.7297	1.8654	7.933E-04	4.178E-02	3.948E-02	905	tags=29%, list=6%, signal=28%
BP	GO:0048715	negative regulation of oligodendrocyte differentiation	16	0.7462	1.8813	8.026E-04	4.205E-02	3.974E-02	2579	tags=50%, list=16%, signal=42%
BP	GO:0045944	positive regulation of transcription by RNA polymerase II	956	0.3155	1.3159	8.044E-04	4.205E-02	3.974E-02	3963	tags=30%, list=25%, signal=24%
BP	GO:0016072	rRNA metabolic process	204	-0.3575	-1.6749	8.244E-04	4.294E-02	4.058E-02	2993	tags=34%, list=19%, signal=28%
BP	GO:0003188	heart valve formation	12	0.7838	1.8413	8.332E-04	4.308E-02	4.071E-02	1278	tags=42%, list=8%, signal=38%
BP	GO:0016441	post-transcriptional gene silencing	74	-0.4188	-1.6822	8.309E-04	4.308E-02	4.071E-02	1532	tags=19%, list=10%, signal=17%
BP	GO:0035107	appendage morphogenesis	134	0.4392	1.6178	8.437E-04	4.331E-02	4.093E-02	4234	tags=36%, list=27%, signal=26%
BP	GO:0035108	limb morphogenesis	134	0.4392	1.6178	8.437E-04	4.331E-02	4.093E-02	4234	tags=36%, list=27%, signal=26%
CC	GO:0120114	Sm-like protein family complex	76	-0.4200	-1.6942	8.478E-04	4.336E-02	4.098E-02	3407	tags=42%, list=22%, signal=33%
BP	GO:0090277	positive regulation of peptide hormone secretion	121	0.4625	1.6822	8.568E-04	4.355E-02	4.116E-02	2163	tags=25%, list=14%, signal=22%
CC	GO:0005840	ribosome	218	-0.4720	-2.2332	8.576E-04	4.355E-02	4.116E-02	1856	tags=30%, list=12%, signal=27%
BP	GO:0046883	regulation of hormone secretion	272	0.3874	1.5278	8.793E-04	4.449E-02	4.205E-02	4503	tags=39%, list=28%, signal=28%
BP	GO:0010721	negative regulation of cell development	245	0.3903	1.5262	8.905E-04	4.490E-02	4.243E-02	3711	tags=34%, list=23%, signal=26%
BP	GO:0048878	chemical homeostasis	893	0.3176	1.3232	9.060E-04	4.552E-02	4.302E-02	4115	tags=29%, list=26%, signal=23%
MF	GO:0031893	vasopressin receptor binding	4	0.9493	1.6859	9.179E-04	4.580E-02	4.328E-02	30	tags=50%, list=0%, signal=50%
BP	GO:0043009	chordate embryonic development	634	0.3331	1.3741	9.162E-04	4.580E-02	4.328E-02	3990	tags=29%, list=25%, signal=23%
BP	GO:0060041	retina development in camera-type eye	185	0.4093	1.5595	9.239E-04	4.593E-02	4.341E-02	3908	tags=37%, list=25%, signal=28%
BP	GO:0008015	blood circulation	425	0.3499	1.4187	9.445E-04	4.679E-02	4.422E-02	3872	tags=30%, list=24%, signal=23%
BP	GO:0060537	muscle tissue development	393	0.3548	1.4330	9.486E-04	4.683E-02	4.426E-02	3927	tags=30%, list=25%, signal=23%
CC	GO:0030684	preribosome	99	-0.3847	-1.6315	9.556E-04	4.701E-02	4.443E-02	2928	tags=32%, list=18%, signal=27%
MF	GO:0003777	microtubule motor activity	52	0.5542	1.7810	9.675E-04	4.728E-02	4.468E-02	2517	tags=37%, list=16%, signal=31%
BP	GO:0042274	ribosomal small subunit biogenesis	101	-0.3995	-1.6979	9.657E-04	4.728E-02	4.468E-02	3162	tags=34%, list=20%, signal=27%
BP	GO:0003179	heart valve morphogenesis	49	0.5534	1.7581	9.733E-04	4.738E-02	4.478E-02	3927	tags=45%, list=25%, signal=34%
BP	GO:0002793	positive regulation of peptide secretion	123	0.4552	1.6589	9.763E-04	4.738E-02	4.478E-02	2163	tags=24%, list=14%, signal=21%
BP	GO:0098698	postsynaptic specialization assembly	42	0.5853	1.8067	1.001E-03	4.794E-02	4.531E-02	1918	tags=36%, list=12%, signal=31%
BP	GO:0051647	nucleus localization	39	0.5902	1.7962	1.005E-03	4.794E-02	4.531E-02	3093	tags=38%, list=20%, signal=31%
BP	GO:0000375	RNA splicing, via transesterification reactions	252	-0.3624	-1.7390	1.008E-03	4.794E-02	4.531E-02	4172	tags=44%, list=26%, signal=33%
BP	GO:0000377	RNA splicing, via transesterification reactions with bulged adenosine as nucleophile	252	-0.3624	-1.7390	1.008E-03	4.794E-02	4.531E-02	4172	tags=44%, list=26%, signal=33%
BP	GO:0000398	mRNA splicing, via spliceosome	252	-0.3624	-1.7390	1.008E-03	4.794E-02	4.531E-02	4172	tags=44%, list=26%, signal=33%
BP	GO:0001578	microtubule bundle formation	112	0.4603	1.6589	9.930E-04	4.794E-02	4.531E-02	3006	tags=35%, list=19%, signal=28%
BP	GO:0001944	vasculature development	650	0.3329	1.3750	1.016E-03	4.814E-02	4.549E-02	4199	tags=32%, list=27%, signal=25%
BP	GO:0006123	mitochondrial electron transport, cytochrome c to oxygen	14	-0.7147	-1.9368	1.032E-03	4.874E-02	4.606E-02	3006	tags=64%, list=19%, signal=52%
BP	GO:0060538	skeletal muscle organ development	179	0.4084	1.5512	1.044E-03	4.914E-02	4.644E-02	2663	tags=23%, list=17%, signal=20%
BP	GO:0006749	glutathione metabolic process	49	-0.4848	-1.7996	1.068E-03	4.997E-02	4.723E-02	2244	tags=37%, list=14%, signal=32%
MF	GO:0015399	primary active transmembrane transporter activity	117	-0.3597	-1.5600	1.068E-03	4.997E-02	4.723E-02	1364	tags=18%, list=9%, signal=17%

Worksheet2: GSEA result for Kyoto Encyclopedia of Genes and Genomes (KEGG) pathways

ID	Description	setSize	enrichmentScore	NES	pvalue	p.adjust	qvalue	rank	leading_edge
rno04934	Cushing syndrome	119	0.5285	1.9626	1.209E-04	7.032E-03	5.346E-03	3416	tags=47%, list=26%, signal=35%
rno04330	Notch signaling pathway	55	0.5733	1.9072	2.681E-04	7.032E-03	5.346E-03	1957	tags=42%, list=15%, signal=36%
rno05217	Basal cell carcinoma	49	0.5764	1.8757	2.735E-04	7.032E-03	5.346E-03	2269	tags=41%, list=17%, signal=34%
rno04081	Hormone signaling	145	0.4928	1.8745	2.355E-04	7.032E-03	5.346E-03	2460	tags=34%, list=19%, signal=28%
rno05224	Breast cancer	110	0.4988	1.8347	1.222E-04	7.032E-03	5.346E-03	3740	tags=51%, list=29%, signal=37%
rno04820	Cytoskeleton in muscle cells	179	0.4615	1.7970	1.136E-04	7.032E-03	5.346E-03	2619	tags=34%, list=20%, signal=28%
rno04360	Axon guidance	163	0.4643	1.7878	1.156E-04	7.032E-03	5.346E-03	2978	tags=37%, list=23%, signal=29%
rno04270	Vascular smooth muscle contraction	104	0.4895	1.7870	2.469E-04	7.032E-03	5.346E-03	1184	tags=20%, list=9%, signal=19%
rno04550	Signaling pathways regulating pluripotency of stem cells	101	0.4912	1.7860	2.476E-04	7.032E-03	5.346E-03	2657	tags=32%, list=20%, signal=25%
rno05165	Human papillomavirus infection	269	0.4357	1.7542	1.082E-04	7.032E-03	5.346E-03	2650	tags=31%, list=20%, signal=25%
rno04080	Neuroactive ligand-receptor interaction	224	0.4404	1.7473	1.107E-04	7.032E-03	5.346E-03	2451	tags=29%, list=19%, signal=24%
rno04928	Parathyroid hormone synthesis, secretion and action	91	0.4858	1.7444	2.509E-04	7.032E-03	5.346E-03	2749	tags=35%, list=21%, signal=28%
rno04390	Hippo signaling pathway	131	0.4569	1.7165	2.394E-04	7.032E-03	5.346E-03	3740	tags=41%, list=29%, signal=30%
rno05200	Pathways in cancer	410	0.3658	1.5094	2.077E-04	7.032E-03	5.346E-03	2856	tags=30%, list=22%, signal=24%
rno03022	Basal transcription factors	38	-0.5781	-2.0864	3.414E-04	7.911E-03	6.015E-03	1094	tags=34%, list=8%, signal=31%
rno03050	Proteasome	41	-0.5165	-1.8903	3.516E-04	7.911E-03	6.015E-03	3437	tags=61%, list=26%, signal=45%
rno03420	Nucleotide excision repair	52	-0.4851	-1.8772	3.827E-04	8.104E-03	6.161E-03	1596	tags=38%, list=12%, signal=34%
rno04260	Cardiac muscle contraction	68	-0.5178	-2.1045	4.243E-04	8.485E-03	6.451E-03	967	tags=28%, list=7%, signal=26%
rno00190	Oxidative phosphorylation	105	-0.6689	-2.9396	5.299E-04	9.693E-03	7.369E-03	1789	tags=47%, list=14%, signal=41%
rno03010	Ribosome	165	-0.5603	-2.6348	7.570E-04	9.693E-03	7.369E-03	1025	tags=35%, list=8%, signal=33%
rno03040	Spliceosome	136	-0.5169	-2.3607	6.341E-04	9.693E-03	7.369E-03	2231	tags=44%, list=17%, signal=37%
rno05415	Diabetic cardiomyopathy	171	-0.4855	-2.2855	7.874E-04	9.693E-03	7.369E-03	1060	tags=26%, list=8%, signal=24%
rno05208	Chemical carcinogenesis - reactive oxygen species	186	-0.4785	-2.2803	8.482E-04	9.693E-03	7.369E-03	874	tags=22%, list=7%, signal=21%
rno05171	Coronavirus disease - COVID-19	185	-0.4669	-2.2241	8.375E-04	9.693E-03	7.369E-03	1025	tags=25%, list=8%, signal=24%
rno04714	Thermogenesis	184	-0.4455	-2.1181	8.375E-04	9.693E-03	7.369E-03	1789	tags=28%, list=14%, signal=24%
rno04061	Viral protein interaction with cytokine and cytokine receptor	30	-0.5754	-1.9576	6.317E-04	9.693E-03	7.369E-03	1118	tags=33%, list=9%, signal=31%
rno04932	Non-alcoholic fatty liver disease	125	-0.4228	-1.9008	5.981E-04	9.693E-03	7.369E-03	1789	tags=26%, list=14%, signal=23%
rno00480	Glutathione metabolism	48	-0.4881	-1.8524	7.429E-04	9.693E-03	7.369E-03	1065	tags=44%, list=8%, signal=40%
rno04512	ECM-receptor interaction	70	0.5004	1.7251	6.525E-04	9.693E-03	7.369E-03	2752	tags=43%, list=21%, signal=34%
rno04723	Retrograde endocannabinoid signaling	126	-0.3814	-1.7194	5.935E-04	9.693E-03	7.369E-03	684	tags=14%, list=5%, signal=14%
rno01522	Endocrine resistance	83	0.4778	1.6923	8.886E-04	9.693E-03	7.369E-03	3444	tags=46%, list=26%, signal=34%
rno04310	Wnt signaling pathway	135	0.4317	1.6271	8.332E-04	9.693E-03	7.369E-03	3416	tags=36%, list=26%, signal=27%
rno04151	PI3K-Akt signaling pathway	261	0.3758	1.5100	8.669E-04	9.693E-03	7.369E-03	2803	tags=30%, list=21%, signal=24%
rno05012	Parkinson disease	225	-0.4684	-2.2855	1.041E-03	1.070E-02	8.137E-03	1789	tags=30%, list=14%, signal=26%
rno05020	Prion disease	223	-0.4266	-2.0804	1.019E-03	1.070E-02	8.137E-03	1789	tags=28%, list=14%, signal=24%
rno05016	Huntington disease	258	-0.4060	-2.0104	1.267E-03	1.267E-02	9.635E-03	1065	tags=22%, list=8%, signal=20%
rno04927	Cortisol synthesis and secretion	48	0.5351	1.7362	1.505E-03	1.426E-02	1.084E-02	3324	tags=48%, list=25%, signal=36%
rno05166	Human T-cell leukemia virus 1 infection	179	0.4001	1.5582	1.477E-03	1.426E-02	1.084E-02	3002	tags=33%, list=23%, signal=26%
rno05014	Amyotrophic lateral sclerosis	311	-0.3830	-1.9353	1.626E-03	1.438E-02	1.094E-02	1501	tags=24%, list=11%, signal=22%
rno00980	Metabolism of xenobiotics by cytochrome P450	33	-0.5197	-1.8108	1.638E-03	1.438E-02	1.094E-02	2430	tags=52%, list=19%, signal=42%
rno05010	Alzheimer disease	318	-0.3308	-1.6767	1.600E-03	1.438E-02	1.094E-02	1789	tags=26%, list=14%, signal=23%
rno04510	Focal adhesion	173	0.4020	1.5597	1.713E-03	1.468E-02	1.116E-02	3495	tags=38%, list=27%, signal=28%
rno05226	Gastric cancer	113	0.4369	1.6114	2.071E-03	1.734E-02	1.318E-02	3416	tags=39%, list=26%, signal=29%
rno04974	Protein digestion and absorption	65	0.4898	1.6736	2.228E-03	1.823E-02	1.386E-02	3106	tags=46%, list=24%, signal=35%
rno04658	Th1 and Th2 cell differentiation	58	0.4997	1.6753	2.534E-03	1.941E-02	1.476E-02	2488	tags=38%, list=19%, signal=31%
rno04916	Melanogenesis	76	0.4642	1.6211	2.436E-03	1.941E-02	1.476E-02	3740	tags=45%, list=29%, signal=32%
rno05022	Pathways of neurodegeneration - multiple diseases	398	-0.2837	-1.4658	2.532E-03	1.941E-02	1.476E-02	1789	tags=21%, list=14%, signal=19%
rno04022	cGMP-PKG signaling pathway	130	0.4171	1.5647	2.756E-03	2.067E-02	1.571E-02	3344	tags=36%, list=26%, signal=27%
rno04024	cAMP signaling pathway	180	0.3864	1.5048	3.068E-03	2.254E-02	1.714E-02	3550	tags=37%, list=27%, signal=27%
rno04020	Calcium signaling pathway	203	0.3788	1.4920	3.245E-03	2.336E-02	1.776E-02	2779	tags=30%, list=21%, signal=24%
rno05204	Chemical carcinogenesis - DNA adducts	26	-0.5302	-1.7400	3.922E-03	2.716E-02	2.064E-02	3697	tags=73%, list=28%, signal=53%
rno04514	Cell adhesion molecule (CAM) interaction	109	0.4244	1.5579	3.923E-03	2.716E-02	2.064E-02	2752	tags=30%, list=21%, signal=24%
rno04911	Insulin secretion	69	0.4679	1.6115	4.171E-03	2.805E-02	2.133E-02	3952	tags=49%, list=30%, signal=35%
rno04518	Integrin signaling	126	0.4101	1.5340	4.208E-03	2.805E-02	2.133E-02	3347	tags=41%, list=26%, signal=31%
rno04810	Regulation of actin cytoskeleton	180	0.3771	1.4686	5.114E-03	3.347E-02	2.545E-02	3292	tags=33%, list=25%, signal=25%
rno05332	Graft-versus-host disease	17	0.6561	1.7107	5.651E-03	3.569E-02	2.714E-02	1240	tags=47%, list=9%, signal=43%
rno04814	Motor proteins	151	0.3884	1.4848	5.611E-03	3.569E-02	2.714E-02	2691	tags=30%, list=21%, signal=24%
rno00982	Drug metabolism - cytochrome P450	31	-0.4901	-1.6789	6.049E-03	3.755E-02	2.854E-02	2430	tags=48%, list=19%, signal=39%
rno04517	IgSF CAM signaling	243	0.3505	1.4008	6.679E-03	4.075E-02	3.098E-02	2657	tags=25%, list=20%, signal=20%
rno04744	Phototransduction	7	-0.7994	-1.7857	7.114E-03	4.268E-02	3.245E-02	1580	tags=57%, list=12%, signal=50%
rno04015	Rap1 signaling pathway	171	0.3722	1.4424	8.016E-03	4.731E-02	3.597E-02	3313	tags=34%, list=25%, signal=26%

Supplementary Table 10

Worksheet 1: Neural cell type-specific pathway enrichments

Cell_type	ID	Description	setSize	enrichmentScore	NES	pvalue	p.adjust	qvalue	rank	leading_edge
Microglia	GO:0002250	adaptive immune response	671	0.560293485	1.724480958	1.28719853683218e-05	0.000154464	4.0648374847332e-05	4545	tags=16%, list=12%, signal=14%
DRD2MSNs	GO:0002250	adaptive immune response	671	0.444494214	1.483879167	4.84427612578748e-05	0.000581313	0.000509924	5722	tags=16%, list=16%, signal=13%
Astrocytes	GO:0002250	adaptive immune response	671	0.440757729	1.418662224	0.000334853	0.001339412	0.000469969	5389	tags=14%, list=15%, signal=12%
DRD1MSNs	GO:0002250	adaptive immune response	671	0.451381038	1.441545173	0.000146311	0.001755729	0.001540114	4513	tags=12%, list=12%, signal=11%
FastSpikingInterneuron(PV)	GO:0002250	adaptive immune response	671	0.309447888	1.22793909	0.002112094	0.012672561	0.008893025	7565	tags=18%, list=21%, signal=14%
OPCs	GO:0002250	adaptive immune response	671	0.357488944	1.217599909	0.028444027	0.037925369	0.006653574	6031	tags=14%, list=16%, signal=12%
Endothelial	GO:0002250	adaptive immune response	671	0.451525668	1.325055004	0.019580036	0.117480215	0.092747538	2776	tags=10%, list=8%, signal=9%
DRD1DRD2MSNs	GO:0002250	adaptive immune response	671	0.342947055	1.277716196	0.010847145	0.130165741	0.125598522	6877	tags=17%, list=19%, signal=14%
CRInterneurons	GO:0002250	adaptive immune response	671	0.350763269	1.126078992	0.129523810	0.17522245	0.107592733	5276	tags=13%, list=14%, signal=11%
EpendymalCells	GO:0002250	adaptive immune response	671	0.315695111	1.093068999	0.144067797	0.246973366	0.129985982	4967	tags=12%, list=14%, signal=10%
LowThresholdSpikeNeurons(SSTNOS)	GO:0002250	adaptive immune response	671	0.331165461	1.105823718	0.108734403	0.652406417	0.652406417	5288	tags=12%, list=14%, signal=10%
CCKInterneurons	GO:0002250	adaptive immune response	671	-0.318030931	-0.865167739	0.703313253	0.767250821	0.74032974	5046	tags=11%, list=14%, signal=10%
Oligodendrocytes	GO:0002250	adaptive immune response	671	0.34074107	0.888547025	0.906093906	0.906093906	0.874301137	7702	tags=15%, list=21%, signal=12%
CRInterneurons	GO:0034340	response to type I interferon	81	0.840941516	2.22832514	0.000453422	0.00272053	0.001670501	702	tags=6%, list=2%, signal=6%
EpendymalCells	GO:0034340	response to type I interferon	81	0.62697893	1.787601071	0.002767708	0.01107083	0.005826753	5892	tags=41%, list=16%, signal=34%
Microglia	GO:0034340	response to type I interferon	81	0.690668594	1.828167191	0.011551504	0.018181615	0.004784635	4346	tags=35%, list=12%, signal=31%
OPCs	GO:0034340	response to type I interferon	81	0.554189418	1.539850054	0.011634890	0.019945525	0.003499215	4767	tags=28%, list=13%, signal=25%
Astrocytes	GO:0034340	response to type I interferon	81	0.558634033	1.51220031	0.016934726	0.025402088	0.008913013	4715	tags=27%, list=13%, signal=24%
Endothelial	GO:0034340	response to type I interferon	81	0.594709205	1.410867897	0.079545455	0.162754304	0.12849024	2006	tags=12%, list=5%, signal=12%
FastSpikingInterneuron(PV)	GO:0034340	response to type I interferon	81	0.357719167	1.136676383	0.194895592	0.292343387	0.205153254	6205	tags=26%, list=17%, signal=22%
Oligodendrocytes	GO:0034340	response to type I interferon	81	0.48645476	1.197222644	0.166315789	0.402956705	0.388817874	6820	tags=22%, list=19%, signal=18%
CCKInterneurons	GO:0034340	response to type I interferon	81	-0.405091047	-0.994460432	0.415913201	0.554550934	0.535093007	4808	tags=23%, list=13%, signal=20%
DRD1DRD2MSNs	GO:0034340	response to type I interferon	81	0.323062379	0.959829669	0.505859375	0.758789063	0.732164885	8428	tags=33%, list=23%, signal=26%
DRD1MSNs	GO:0034340	response to type I interferon	81	0.286356654	0.781741542	0.802746567	0.873737374	0.766436293	6941	tags=21%, list=19%, signal=17%
LowThresholdSpikeNeurons(SSTNOS)	GO:0034340	response to type I interferon	81	0.292941943	0.799768183	0.908914729	0.911067194	0.911067194	7000	tags=26%, list=19%, signal=21%
DRD2MSNs	GO:0034340	response to type I interferon	81	0.231035916	0.660848615	0.964797914	0.964797914	0.84631396	9210	tags=27%, list=25%, signal=20%
Astrocytes	GO:0034341	response to type II interferon	136	0.677419938	1.967069129	5.51417764944816e-07	6.61701317933779e-06	2.32175901029396e-06	4337	tags=26%, list=12%, signal=23%
OPCs	GO:0034341	response to type II interferon	136	0.578949123	1.71003435	0.000513468	0.002053871	0.000360328	5222	tags=29%, list=14%, signal=25%
Microglia	GO:0034341	response to type II interferon	136	0.671288321	1.85140208	0.001354163	0.00406249	0.001069076	4766	tags=28%, list=13%, signal=24%
CRInterneurons	GO:0034341	response to type II interferon	136	0.767211284	2.174203774	0.003210405	0.012810765	0.007866259	1124	tags=9%, list=3%, signal=9%
Endothelial	GO:0034341	response to type II interferon	136	0.643331328	1.634893693	0.013276033	0.117480215	0.092747538	3141	tags=26%, list=9%, signal=24%
FastSpikingInterneuron(PV)	GO:0034341	response to type II interferon	136	0.36211938	1.241680532	0.064587973	0.142180095	0.099775505	7429	tags=30%, list=20%, signal=24%
EpendymalCells	GO:0034341	response to type II interferon	136	0.372783697	1.12735378	0.177914110	0.254620123	0.134010591	6244	tags=25%, list=17%, signal=21%
CCKInterneurons	GO:0034341	response to type II interferon	136	-0.468995279	-1.203203879	0.117850953	0.393617021	0.379805898	2892	tags=15%, list=8%, signal=14%
LowThresholdSpikeNeurons(SSTNOS)	GO:0034341	response to type II interferon	136	-0.372551588	-1.098754469	0.218142549	0.716094033	0.716094033	8418	tags=33%, list=23%, signal=26%
DRD1MSNs	GO:0034341	response to type II interferon	136	0.392762061	1.147303964	0.238958097	0.716874292	0.628837098	6690	tags=26%, list=18%, signal=21%
DRD1DRD2MSNs	GO:0034341	response to type II interferon	136	0.328829844	1.053414175	0.328273245	0.758789063	0.732164885	6377	tags=24%, list=17%, signal=20%
Oligodendrocytes	GO:0034341	response to type II interferon	136	0.390492075	0.982498627	0.539245668	0.808868502	0.780487151	6992	tags=24%, list=19%, signal=19%
DRD2MSNs	GO:0034341	response to type II interferon	136	0.286061743	0.867845097	0.697560976	0.964797914	0.84631396	7752	tags=30%, list=21%, signal=24%
Astrocytes	GO:0038061	non-canonical NF-kappaB sign	122	0.608579365	1.745199175	0.000216493	0.001298955	0.000455774	4617	tags=26%, list=13%, signal=23%
OPCs	GO:0038061	non-canonical NF-kappaB sign	122	0.568190515	1.661226151	0.001241335	0.003724006	0.000653334	4264	tags=26%, list=12%, signal=23%
EpendymalCells	GO:0038061	non-canonical NF-kappaB sign	122	0.487919305	1.46986349	0.016235419	0.032470839	0.017089915	3048	tags=25%, list=8%, signal=23%
FastSpikingInterneuron(PV)	GO:0038061	non-canonical NF-kappaB sign	122	0.382869419	1.287223769	0.027217325	0.108869301	0.076399509	5732	tags=32%, list=16%, signal=27%
Microglia	GO:0038061	non-canonical NF-kappaB sign	122	0.469698674	1.279476992	0.133144476	0.145248519	0.038223295	6232	tags=39%, list=17%, signal=33%
CRInterneurons	GO:0038061	non-canonical NF-kappaB sign	122	0.431657164	1.202740956	0.131416838	0.17522245	0.107592733	4487	tags=26%, list=12%, signal=23%
Endothelial	GO:0038061	non-canonical NF-kappaB sign	122	0.452051383	1.127138925	0.235119048	0.352678571	0.278430451	3311	tags=21%, list=9%, signal=19%
CCKInterneurons	GO:0038061	non-canonical NF-kappaB sign	122	-0.464957273	-1.172546144	0.131205674	0.393617021	0.379805898	3898	tags=21%, list=11%, signal=19%
DRD2MSNs	GO:0038061	non-canonical NF-kappaB sign	122	0.423985767	1.27875737	0.117283951	0.469135802	0.411522634	8420	tags=37%, list=23%, signal=28%
LowThresholdSpikeNeurons(SSTNOS)	GO:0038061	non-canonical NF-kappaB sign	122	0.33002432	0.949883955	0.576779026	0.818061089	0.818061089	6431	tags=25%, list=18%, signal=21%
Oligodendrocytes	GO:0038061	non-canonical NF-kappaB sign	122	0.350876589	0.877943765	0.737166324	0.830426716	0.801288937	7068	tags=22%, list=19%, signal=18%
DRD1DRD2MSNs	GO:0038061	non-canonical NF-kappaB sign	122	0.267207639	0.843352918	0.808712121	0.860411899	0.830222008	6238	tags=26%, list=17%, signal=22%
DRD1MSNs	GO:0038061	non-canonical NF-kappaB sign	122	0.365007525	1.049776482	0.382217090	0.873737374	0.766436293	9573	tags=35%, list=26%, signal=26%
OPCs	GO:0043065	positive regulation of apoptot	494	0.505696514	1.685103716	4.03688914375949e-07	4.84426697251139e-06	8.49871398686209e-07	5919	tags=36%, list=16%, signal=31%
Microglia	GO:0043065	positive regulation of apoptot	494	0.527276338	1.597877794	0.000822503	0.003290013	0.000865793	4457	tags=23%, list=12%, signal=21%
Astrocytes	GO:0043065	positive regulation of apoptot	494	0.44485898	1.421924811	0.001693619	0.004064687	0.001426206	6790	tags=29%, list=19%, signal=24%
FastSpikingInterneuron(PV)	GO:0043065	positive regulation of apoptot	494	0.336218167	1.310522937	0.001398761	0.012672561	0.008893025	5786	tags=26%, list=16%, signal=22%
EpendymalCells	GO:0043065	positive regulation of apoptot	494	0.38882586	1.313168541	0.009698820	0.023277168	0.012251141	4339	tags=24%, list=12%, signal=21%
CRInterneurons	GO:0043065	positive regulation of apoptot	494	0.479935026	1.508410087	0.013899788	0.033359491	0.020483898	4889	tags=24%, list=13%, signal=22%
CCKInterneurons	GO:0043065	positive regulation of apoptot	494	-0.414354854	-1.140292956	0.058551618	0.351309707	0.338983051	4542	tags=25%, list=12%, signal=22%
Oligodendrocytes	GO:0043065	positive regulation of apoptot	494	0.44998053	1.171326208	0.043956044	0.402956705	0.388817874	8066	tags=30%, list=22%, signal=24%
DRD2MSNs	GO:0043065	positive regulation of apoptot	494	0.339848115	1.116963023	0.211640212	0.634920635	0.556947925	8022	tags=30%, list=22%, signal=24%
DRD1MSNs	GO:0043065	positive regulation of apoptot	494	0.356935202	1.131949023	0.163412127	0.65364851	0.573375886	7402	tags=26%, list=20%, signal=21%
LowThresholdSpikeNeurons(SSTNOS)	GO:0043065	positive regulation of apoptot	494	0.317605982	1.040671939	0.298372514	0.716094033	0.716094033	5310	tags=25%, list=15%, signal=22%
Endothelial	GO:0043065	positive regulation of apoptot	494	-0.328310742	-0.859759723	0.792011019	0.792011019	0.625271857	2704	tags=15%, list=7%, signal=14%
DRD1DRD2MSNs	GO:0043065	positive regulation of apoptot	494	-0.242736982	-0.910440443	0.860411899	0.860411899	0.830222008	7404	tags=27%, list=20%, signal=21%
OPCs	GO:0043331	response to dsRNA	54	0.63744607	1.654833832	0.009089832	0.018179665	0.003189415	3631	tags=24%, list=10%, signal=22%
EpendymalCells	GO:0043331	response to dsRNA	54	0.423310267	1.1208096	0.255319149	0.278529981	0.146594727	5109	tags=26%, list=14%, signal=22%
Astrocytes	GO:0043331	response to dsRNA	54	0.449708849	1.157816591	0.242816092	0.323754789	0.113598172	6319	tags=24%, list=17%, signal=20%
Microglia	GO:0043331	response to dsRNA	54	0.412681215	1.027516725	0.386837881	0.386837881	0.101799442	5556	tags=30%, list=15%, signal=25%
CRInterneurons	GO:0043331	response to dsRNA	54	0.384938797	0.960643632	0.511061947	0.511061947	0.313809967	6138	tags=22%, list=17%, signal=19%
Endothelial	GO:0043331	response to dsRNA	54	-0.468959905	-1.012554195	0.430645161	0.56744186	0.447980416	5277	tags=26%, list=14%, signal=22%
Oligodendrocytes	GO:0043331	response to dsRNA	54	-0.323196581	-1.015435381	0.393939394	0.675324675	0.651629073	497	tags=7%, list=1%, signal=7%
LowThresholdSpikeNeurons(SSTNOS)	GO:0043331	response to dsRNA	54	-0.424925971	-1.107876178	0.249484536	0.716094033	0.716094033	5593	tags=30%, list=15%, signal=25%
DRD1DRD2MSNs	GO:0043331	response to dsRNA	54	-0.342750657	-0.987503357	0.457317073	0.758789063	0.732164885	9776	tags=37%, list=27%, signal=27%
CCKInterneurons	GO:0043331	response to dsRNA	54	0.334731443	0.789289879	0.869848156	0.869848156	0.839327168	5967	tags=31%, list=16%, signal=26%
DRD1MSNs	GO:0043331	response to dsRNA	54	0.297802947	0.775086841	0.796833773	0.873737374	0.766436293	6726	tags=17%, list=18%, signal=14%
FastSpikingInterneuron(PV)	GO:0043331	response to dsRNA	54	0.282317612	0.838354568	0.815217391	0.889328063	0.624089869	1468	tags=9%, list=4%, signal=9%
DRD2MSNs	GO:0043331	response to dsRNA	54	0.302686392	0.826846149	0.721835883	0.964797914	0.84631396	7160	tags=22%, list=20%, signal=18%
CRInterneurons	GO:0060337	type I interferon-mediated sig	74	0.850955543	2.225712073	0.000447616	0.00272053	0.001670501	702	tags=7%, list=2%, signal=7%
EpendymalCells	GO:0060337	type I interferon-mediated sig	74	0.640841798	1.7776571	0.004084798	0.012254394	0.006449681	5892	tags=41%, list=16%, signal=34%
Microglia	GO:0060337	type I interferon-mediated sig	74	0.713327817	1.850007133	0.007746506	0.015493013	0.004077109	4346	tags=35%, list=12%, signal=31%
Astrocytes	GO:0060337	type I interferon-mediated sig	74	0.584983977	1.575522119	0.011085958	0.022171916	0.00777962	4715	tags=30%, list=13%, signal=26%
OPCs	GO:0060337	type I interferon-mediated sig	74	0.553820774	1.517565894	0.033940261	0.040728313	0.007145318	4767	tags=27%, list=13%, signal=24%
FastSpikingInterneuron(PV)	GO:0060337	type I interferon-mediated sig	74	0.431019132	1.344801189	0.039974665	0.119923996	0.08415719	6205	tags=27%, list=17%, signal=22%
Endothelial	GO:0060337	type I interferon-mediated sig	74	0.658226492	1.543238078	0.037298768	0.149195073	0.117785584	2006	tags=14%, list=5%, signal=13%
Oligodendrocytes	GO:0060337	type I interferon-mediated sig	74	0.500154603	1.220549561	0.157338965	0.402956705	0.388817874	6820	tags=23%, list=19%, signal=19%
CCKInterneurons	GO:0060337	type I interferon-mediated sig	74	-0.437370553	-1.054435708	0.328996283	0.554550934	0.535093007	4808	tags=23%, list=13%, signal=20%
DRD1DRD2MSNs	GO:0060337	type I interferon-mediated sig	74	0.352543212	1.03340677	0.377733598	0.758789063	0.732164885	8428	tags=34%, list=23%, signal=26%
LowThresholdSpikeNeurons(SSTNOS)	GO:0060337	type I interferon-mediated sig	74	0.353542822	0.949181405	0.564612326	0.818061089	0.818061089	7537	tags=30%, list=21%, signal=24%

DRD1MSNs	GO:0060337	type I interferon-mediated sig	74	0.3263225	0.881532246	0.652284264	0.873737374	0.766436293	6941	tags=23%, list=19%, signal=19%
DRD2MSNs	GO:0060337	type I interferon-mediated sig	74	0.270418746	0.764557094	0.840369393	0.964797914	0.84631396	9210	tags=28%, list=25%, signal=21%
EpendymalCells	GO:0071357	cellular response to type I inte	75	0.636276643	1.772562602	0.002399307	0.01107083	0.005826753	6605	tags=44%, list=18%, signal=36%
CRInterneurons	GO:0071357	cellular response to type I inte	75	0.85092739	2.220525595	0.004270255	0.012810765	0.007866259	702	tags=7%, list=2%, signal=7%
Microglia	GO:0071357	cellular response to type I inte	75	0.710303299	1.848245065	0.012121076	0.018181615	0.004784635	4346	tags=35%, list=12%, signal=31%
Astrocytes	GO:0071357	cellular response to type I inte	75	0.577169034	1.556833036	0.014461388	0.024790951	0.008698579	4715	tags=29%, list=13%, signal=26%
OPCs	GO:0071357	cellular response to type I inte	75	0.548416845	1.504780633	0.025579786	0.037925369	0.006653574	4767	tags=27%, list=13%, signal=23%
FastSpikingInterneuron(PV)	GO:0071357	cellular response to type I inte	75	0.409262921	1.278957027	0.071090047	0.142180095	0.099775505	6205	tags=27%, list=17%, signal=22%
Endothelial	GO:0071357	cellular response to type I inte	75	0.621742907	1.454450024	0.051655495	0.154966484	0.122341961	2006	tags=13%, list=5%, signal=13%
Oligodendrocytes	GO:0071357	cellular response to type I inte	75	0.494253988	1.208438961	0.167898627	0.402956705	0.388817874	6820	tags=23%, list=19%, signal=18%
CCKInterneurons	GO:0071357	cellular response to type I inte	75	-0.446883426	-1.079958231	0.290740741	0.554550934	0.535093007	4808	tags=24%, list=13%, signal=21%
DRD1DRD2MSNs	GO:0071357	cellular response to type I inte	75	0.349283562	1.030708	0.376725838	0.758789063	0.732164885	8428	tags=33%, list=23%, signal=26%
LowThresholdSpikeNeurons(SSTNOS)	GO:0071357	cellular response to type I inte	75	0.343616542	0.92442492	0.613545817	0.818061089	0.818061089	7537	tags=29%, list=21%, signal=23%
DRD1MSNs	GO:0071357	cellular response to type I inte	75	0.319987291	0.865815109	0.675538657	0.873737374	0.766436293	6941	tags=23%, list=19%, signal=18%
DRD2MSNs	GO:0071357	cellular response to type I inte	75	0.25897649	0.733364485	0.889473684	0.964797914	0.84631396	9210	tags=28%, list=25%, signal=21%
OPCs	GO:0097190	apoptotic signaling pathway	588	0.461049009	1.556071336	1.36200069104998e-05	8.17200414629987e-05	1.43368493794735e-05	5764	tags=35%, list=16%, signal=30%
Microglia	GO:0097190	apoptotic signaling pathway	588	0.549832321	1.680162588	5.63689536982038e-05	0.000338214	8.90036111024271e-05	5155	tags=31%, list=14%, signal=27%
EpendymalCells	GO:0097190	apoptotic signaling pathway	588	0.434107226	1.48496994	6.82648100099738e-05	0.000819178	0.000431146	5149	tags=31%, list=14%, signal=27%
Astrocytes	GO:0097190	apoptotic signaling pathway	588	0.44201055	1.418960083	0.001291587	0.003874762	0.001359566	7858	tags=34%, list=21%, signal=27%
CRInterneurons	GO:0097190	apoptotic signaling pathway	588	0.400072963	1.266628117	0.092747522	0.158995752	0.09762897	5316	tags=27%, list=15%, signal=23%
FastSpikingInterneuron(PV)	GO:0097190	apoptotic signaling pathway	588	0.279484705	1.104040714	0.103975535	0.178243775	0.125083351	7185	tags=31%, list=20%, signal=25%
DRD2MSNs	GO:0097190	apoptotic signaling pathway	588	0.37410986	1.240110697	0.036231884	0.217391304	0.190694127	9256	tags=36%, list=25%, signal=28%
DRD1MSNs	GO:0097190	apoptotic signaling pathway	588	0.380208816	1.209175642	0.047764228	0.286585366	0.251390672	9165	tags=34%, list=25%, signal=26%
CCKInterneurons	GO:0097190	apoptotic signaling pathway	588	-0.455204428	-1.239004946	0.048780488	0.351309707	0.338983051	4150	tags=27%, list=11%, signal=24%
Oligodendrocytes	GO:0097190	apoptotic signaling pathway	588	0.421365875	1.097262934	0.125874126	0.402956705	0.388817874	7999	tags=30%, list=22%, signal=24%
Endothelial	GO:0097190	apoptotic signaling pathway	588	0.33934858	0.984872616	0.472868217	0.56744186	0.447980416	4007	tags=27%, list=11%, signal=24%
LowThresholdSpikeNeurons(SSTNOS)	GO:0097190	apoptotic signaling pathway	588	-0.324998234	-1.100133262	0.090090090	0.652406417	0.652406417	6705	tags=31%, list=18%, signal=26%
DRD1DRD2MSNs	GO:0097190	apoptotic signaling pathway	588	-0.288106229	-1.089948459	0.145199063	0.758789063	0.732164885	7521	tags=30%, list=21%, signal=24%
Microglia	GO:0150076	neuroinflammatory response	69	0.584084383	1.509154107	0.042121685	0.056162246	0.014779539	4580	tags=28%, list=13%, signal=24%
Endothelial	GO:0150076	neuroinflammatory response	69	-0.639816894	-1.416608136	0.081377152	0.162754304	0.12849024	2021	tags=19%, list=6%, signal=18%
OPCs	GO:0150076	neuroinflammatory response	69	0.419360856	1.130568487	0.255769231	0.279020979	0.048951049	5567	tags=20%, list=15%, signal=17%
CRInterneurons	GO:0150076	neuroinflammatory response	69	0.400478808	1.030418397	0.368763557	0.442516269	0.271720516	7158	tags=38%, list=20%, signal=30%
EpendymalCells	GO:0150076	neuroinflammatory response	69	-0.354023206	-0.963960957	0.499009901	0.499009901	0.26263679	7502	tags=38%, list=20%, signal=30%
Astrocytes	GO:0150076	neuroinflammatory response	69	0.370778872	0.995570296	0.466206897	0.508589342	0.178452401	5389	tags=16%, list=15%, signal=14%
FastSpikingInterneuron(PV)	GO:0150076	neuroinflammatory response	69	0.331628605	1.01795967	0.413551402	0.551401869	0.38694868	7666	tags=33%, list=21%, signal=26%
DRD1DRD2MSNs	GO:0150076	neuroinflammatory response	69	0.342836672	0.990788969	0.458585859	0.758789063	0.732164885	8041	tags=33%, list=22%, signal=26%
CCKInterneurons	GO:0150076	neuroinflammatory response	69	0.374034558	0.916212187	0.637554585	0.765065502	0.738221099	2026	tags=14%, list=6%, signal=14%
Oligodendrocytes	GO:0150076	neuroinflammatory response	69	0.356834708	0.861738194	0.744385027	0.830426716	0.801288937	5540	tags=23%, list=15%, signal=20%
DRD1MSNs	GO:0150076	neuroinflammatory response	69	0.269323138	0.724062994	0.873737374	0.873737374	0.766436293	4222	tags=13%, list=12%, signal=12%
LowThresholdSpikeNeurons(SSTNOS)	GO:0150076	neuroinflammatory response	69	0.319017895	0.848136372	0.792899408	0.911067194	0.911067194	4210	tags=23%, list=12%, signal=21%
DRD2MSNs	GO:0150076	neuroinflammatory response	69	0.239900877	0.669473933	0.957994580	0.964797914	0.84631396	9646	tags=35%, list=26%, signal=26%
Microglia	GO:0150077	regulation of neuroinflammat	34	0.611490768	1.431027244	0.084033613	0.100840336	0.026536931	5237	tags=32%, list=14%, signal=28%
EpendymalCells	GO:0150077	regulation of neuroinflammat	34	-0.476804695	-1.150047085	0.236734694	0.278529981	0.146594727	2583	tags=18%, list=7%, signal=16%
Endothelial	GO:0150077	regulation of neuroinflammat	34	-0.607975542	-1.233793682	0.202551834	0.347231716	0.274130302	2021	tags=18%, list=6%, signal=17%
CCKInterneurons	GO:0150077	regulation of neuroinflammat	34	-0.55836716	-1.194207481	0.205607477	0.493457944	0.47614363	1072	tags=15%, list=3%, signal=14%
OPCs	GO:0150077	regulation of neuroinflammat	34	0.411053606	0.975645781	0.507812500	0.5078125	0.089089912	4991	tags=21%, list=14%, signal=18%
CRInterneurons	GO:0150077	regulation of neuroinflammat	34	0.419002763	0.972677023	0.468879668	0.511061947	0.313809967	4956	tags=26%, list=14%, signal=23%
FastSpikingInterneuron(PV)	GO:0150077	regulation of neuroinflammat	34	-0.364628381	-0.975724487	0.461538462	0.553846154	0.388663968	3186	tags=15%, list=9%, signal=13%
Oligodendrocytes	GO:0150077	regulation of neuroinflammat	34	0.509034584	1.130639265	0.307873090	0.615746181	0.594141052	8530	tags=44%, list=23%, signal=34%
DRD1DRD2MSNs	GO:0150077	regulation of neuroinflammat	34	0.355386967	0.910425098	0.571142285	0.761523046	0.734802939	2204	tags=12%, list=6%, signal=11%
DRD1MSNs	GO:0150077	regulation of neuroinflammat	34	-0.286889765	-0.846260395	0.666666667	0.873737374	0.766436293	3652	tags=18%, list=10%, signal=16%
LowThresholdSpikeNeurons(SSTNOS)	GO:0150077	regulation of neuroinflammat	34	0.319815626	0.757454831	0.911067194	0.911067194	0.911067194	5293	tags=21%, list=14%, signal=18%
Astrocytes	GO:0150077	regulation of neuroinflammat	34	-0.252008552	-0.680519547	0.961077844	0.961077844	0.337220296	5156	tags=32%, list=14%, signal=28%
DRD2MSNs	GO:0150077	regulation of neuroinflammat	34	-0.276544111	-0.778384526	0.793413174	0.964797914	0.84631396	4069	tags=18%, list=11%, signal=16%
Microglia	GO:2001235	positive regulation of apoptot	131	0.669193968	1.853589643	0.003153554	0.007568529	0.001991718	3398	tags=22%, list=9%, signal=20%
OPCs	GO:2001235	positive regulation of apoptot	131	0.526781689	1.548144631	0.004366750	0.0104802	0.001838631	5414	tags=42%, list=15%, signal=36%
CRInterneurons	GO:2001235	positive regulation of apoptot	131	0.454624921	1.282327932	0.078787879	0.157575758	0.096757044	5459	tags=31%, list=15%, signal=26%
EpendymalCells	GO:2001235	positive regulation of apoptot	131	0.377972193	1.135976499	0.190965092	0.254620123	0.134010591	5149	tags=31%, list=14%, signal=27%
Astrocytes	GO:2001235	positive regulation of apoptot	131	0.360185519	1.041222626	0.384905660	0.461886792	0.162065541	7858	tags=31%, list=21%, signal=24%
CCKInterneurons	GO:2001235	positive regulation of apoptot	131	-0.396566837	-1.009138982	0.395061728	0.554550934	0.535093007	4323	tags=26%, list=12%, signal=23%
Endothelial	GO:2001235	positive regulation of apoptot	131	-0.396120405	-0.941825223	0.532041729	0.580409159	0.458217757	3441	tags=21%, list=9%, signal=19%
LowThresholdSpikeNeurons(SSTNOS)	GO:2001235	positive regulation of apoptot	131	-0.321996838	-0.948065049	0.594882729	0.818061089	0.818061089	5237	tags=24%, list=14%, signal=21%
Oligodendrocytes	GO:2001235	positive regulation of apoptot	131	0.348372944	0.875770516	0.761224490	0.830426716	0.801288937	9249	tags=32%, list=25%, signal=24%
DRD1DRD2MSNs	GO:2001235	positive regulation of apoptot	131	-0.272017109	-0.890678485	0.745798319	0.860411899	0.830222008	7827	tags=35%, list=21%, signal=28%
DRD1MSNs	GO:2001235	positive regulation of apoptot	131	0.303729463	0.880778294	0.690366972	0.873737374	0.766436293	8999	tags=29%, list=25%, signal=22%
FastSpikingInterneuron(PV)	GO:2001235	positive regulation of apoptot	131	-0.255024456	-0.841494404	0.891651865	0.891651865	0.625720607	7997	tags=32%, list=22%, signal=25%
DRD2MSNs	GO:2001235	positive regulation of apoptot	131	0.296625461	0.89561915	0.641975309	0.964797914	0.84631396	9866	tags=34%, list=27%, signal=25%

Worksheet 2: Neural cell-type specific DEGs

Gene	Alias	Description	Cell_type	Log2_Fold-change	Pvalue	FDR
NFKB2	P52	Critical NF-κB gene, multifaceted function	Astrocytes	0.352530283	0.000104483	0.005784578
			CCKInterneurons	-1.6552E-05	0.946977863	0.999938679
			CRInterneurons	0.000633782	0.750774546	NA
			DRD1DRD2MSNs	-0.013387533	0.498775056	NA
			DRD1MSNs	-0.053874654	0.0392995	0.263111629
			DRD2MSNs	-0.026518329	0.195019404	0.540777194
			Endothelial	0.007342257	0.281869993	0.999964992
			EpendymalCells	0.015873974	0.253726655	0.978514813
			FastSpikingInterneuron(PV)	-0.018011347	0.265826266	NA
			LowThresholdSpikeNeurons(SSTNOS)	0.004740474	0.581088605	0.999909403
			Microglia	0.010846517	0.291552508	0.722902953
			OPCs	-0.008613862	0.512367275	0.828301536
			Oligodendrocytes	0.012255493	0.72891996	0.851424327
OAS1	OIAS	dsRNA sensor	Astrocytes	-0.000947569	0.974036674	0.98995779
			CCKInterneurons	0.000123336	0.80971296	0.999938679
			CRInterneurons	-0.005782181	0.148979733	0.781505383
			DRD1DRD2MSNs	-0.002175671	0.916689379	NA
			DRD1MSNs	0.020789001	0.279152869	0.638338664
			DRD2MSNs	0.021766445	0.281750025	0.630471895
			Endothelial	-0.016962766	0.065341251	0.916989974
			EpendymalCells	0.008225695	0.471524243	0.999745691
			FastSpikingInterneuron(PV)	-0.033042269	0.222573646	0.494027235
			LowThresholdSpikeNeurons(SSTNOS)	-0.04599552	0.038848949	0.645438359
			Microglia	-0.016289482	0.236349875	0.677683469
			OPCs	-0.010930431	0.459029212	0.800886275
			Oligodendrocytes	0.244281134	0.007581168	0.047451999
REL	C-Rel	Rel homology domain protein, NF-κB subunit, core transcriptional regulator in:<br>• apoptosis,<br>• inflammation,<br>• immune response,<br>• (also) oncogenic processes	Astrocytes	0.017771296	0.594642024	0.815923412
			CCKInterneurons	0.001471074	0.552327434	0.999938679
			CRInterneurons	0.007158562	0.355803288	0.90442329
			DRD1DRD2MSNs	0.065439298	0.028260348	0.184423052
			DRD1MSNs	0.041492436	0.087433615	0.382075821
			DRD2MSNs	0.056523845	0.032064614	0.235716346
			Endothelial	-0.010944318	0.175264371	0.999964992
			EpendymalCells	-0.004538935	0.814579204	0.999745691
			FastSpikingInterneuron(PV)	0.028937153	0.378752312	0.636869991
			LowThresholdSpikeNeurons(SSTNOS)	-0.038195107	0.384337033	0.999909403
			Microglia	0.019227363	0.14932016	0.581276007
			OPCs	0.016494998	0.407358608	0.766191426
			Oligodendrocytes	0.104976383	0.001329869	0.014898398
RELB	REL-B	• Critical NF-κB subunit	Astrocytes	0.277268391	0.001140382	0.025764429
			CCKInterneurons	0.000208307	0.755680489	0.999938679
			CRInterneurons	0.007059134	0.038025676	0.591956182
			DRD1DRD2MSNs	0.023723794	0.369561206	0.637005286
			DRD1MSNs	0.126723825	0.003442397	0.075594527
			DRD2MSNs	0.201160649	0.000807406	0.033372388
			Endothelial	0.004225822	0.496522271	0.999964992
			EpendymalCells	0.015458098	0.160558538	0.91648916
			FastSpikingInterneuron(PV)	0.061810697	0.007466304	0.106046723
			LowThresholdSpikeNeurons(SSTNOS)	-0.01425276	0.497116216	0.999909403
			Microglia	0.030119306	0.04572863	0.382843983
			OPCs	0.033122247	0.069070893	0.400074707
			Oligodendrocytes	0.02837268	0.435691296	0.637093328
TICAM1	TICAM-1, TRIF	• TLR3-specific direct downstream target.<br>• Activated when TLR3 binds to dsRNA.<br>• TICAM1 is known to activates NF-κB and I-IFN signaling pathways to initiate the TLR3-mediated, pro-inflammatory dsRNA-sensing signaling cascade.	Astrocytes	0.007016875	0.832827506	0.93752819
			CCKInterneurons	0.000387273	0.759884056	0.999938679
			CRInterneurons	-0.002752	0.604166608	0.96140676
			DRD1DRD2MSNs	-0.009321033	0.77039235	0.896210307
			DRD1MSNs	0.065229235	0.013022114	0.151227333
			DRD2MSNs	0.034727941	0.17597786	0.514741864
			Endothelial	0.00078941	0.902429417	0.999964992
			EpendymalCells	0.015173048	0.299350299	0.994484315
			FastSpikingInterneuron(PV)	0.093488602	0.010168696	0.117546396
			LowThresholdSpikeNeurons(SSTNOS)	0.024768592	0.45984288	0.999909403
			Microglia	-0.005056673	0.760127246	0.938638842
			OPCs	0.045894775	0.038955007	0.314654917
			Oligodendrocytes	0.330851885	0.000602542	0.00887805
